# Targeting AKT and CK2 represents a novel therapeutic strategy for SMO constitutive activation‐driven medulloblastoma

**DOI:** 10.1111/cns.13835

**Published:** 2022-04-14

**Authors:** Yue‐Liang Yao, Yan‐Xia Wang, Fei‐Cheng Yang, Chuan Wang, Min Mao, Qu‐Jing Gai, Jiang He, Yan Qin, Xiao‐Xue Yao, Xi Lan, Jiang Zhu, Hui‐Min Lu, Hui Zeng, Xiao‐Hong Yao, Xiu‐Wu Bian, Yan Wang

**Affiliations:** ^1^ Institute of Pathology and Southwest Cancer Center Southwest Hospital Army Medical University (Third Military Medical University) Chongqing China; ^2^ Fuzhou Medical College of Nanchang University Fuzhou China

**Keywords:** CK2, constitutive activation, medulloblastoma, SMO, W535L mutation

## Abstract

**Aims:**

Sonic hedgehog subtype medulloblastoma is featured with overactivation of hedgehog pathway and can be targeted by SMO‐specific inhibitors. However, the resistance is frequently developed leading to treatment failure of SMO inhibitors. W535L mutation of SMO (SMO^W535L^) is thought to be an oncogenic driver for Sonic hedgehog subtype MB and confer resistance to SMO inhibitors. The regulation network of SMO^W535L^ remains to be explored in comparison with wild‐type SMO (SMO^WT^).

**Methods:**

In this study, we profiled transcriptomes, methylomes, and interactomes of MB cells expression SMOWT or SMOW535L in the treatment of DMSO or SMO inhibitor, respectively.

**Results:**

Analysis of transcriptomic data indicated that SMO inhibitor disrupted processes of endocytosis and cilium organization in MB cells with SMO^WT^, which are necessary for SMO activation. In MB cells with SMO^W535L^, however, SMO inhibitor did not affect the two processes‐related genes, implying resistance of SMO^W535L^ toward SMO inhibitor. Moreover, we noticed that SMO inhibitor significantly inhibited metabolism‐related pathways. Our metabolic analysis indicated that nicotinate and nicotinamide metabolism, glycerolipid metabolism, beta‐alanine metabolism, and synthesis and degradation of ketone bodies might be involved in SMO^W535L^ function maintenance. Interactomic analysis revealed casein kinase II (CK2) as an important SMO‐associated protein. Finally, we linked CK2 and AKT together and found combination of inhibitors targeting CK2 and AKT showed synergetic effects to inhibit the growth of MB cells with SMO constitutive activation mutation.

**Conclusions:**

Taken together, our work described SMO‐related transcriptomes, metabolomes, and interactomes under different SMO status and treatment conditions, identifying CK2 and AKT as therapeutic targets for SHH‐subtype MB cells with SMO inhibitor resistance.

## Graphical abstract

This work profiled transcriptomes, metabolomes, and interactomes of wild‐type and constitutively activated SMO and revealed CK2 and AKT as therapeutic targets for SHH‐subtype MB cells with SMO inhibitor resistance.

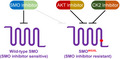

## 1 INTRODUCTION

Medulloblastoma (MB) is the most common malignant brain tumor in children and accounts for approximately 20% of all pediatric central nervous system (CNS) tumors.^1^ Current multimodal treatment has led to a 70–90% 5‐year overall survival rate but the prognosis for patients with tumor dissemination and recurrent MB remains poor with long‐term neurocognitive and neuroendocrine complications.^2^, ^3^, ^4^ MB has been undergoing extensive investigation through next‐generation sequencing studies, which have tremendously advanced our understanding of driver genes, pathways, and molecular processes in MB.^5^, ^6^, ^7^ Four major molecular subtypes of MB have been profiled with various clinical relevance and distinctive transcriptional and epigenetic signatures.^8^, ^9^ WNT‐ and SHH‐subtype MBs are featured with constitutive activation of the Wingless and Sonic hedgehog signaling (SHH) pathways, respectively. By contrast, the genetic features of Group 3‐ and Group 4‐subtype MB subgroups remain unclear.^8^, ^9^, ^10^ The SHH‐subtype MB mainly affects patients of less than 5 years old or more than 16 years old and account for 30% of all MBs with a 5‐year overall survival rate of about 70%.^11^, ^12^, ^13^

The canonical SHH signaling is initiated by hedgehog ligand binding to the pathway suppressor PTCH1 followed by activation of key G protein‐coupled receptor smoothened (SMO). Generally, SMO goes through degradation without activation but endocytosis and cilium transport with activation.^14^, ^15^, ^16^ The activated SMO further leads to activation of Gli transcription factors for biological functions and aberrant SHH signaling pathway plays driver roles in several types of cancers.^17^, ^18^ Mutation of SMO 535 Tryptophan (W) into Leucine (L) (SMO^W535L^) leads to constitutive activation of SMO and has been found as oncogenic driver mutations in medulloblastoma and basal cell carcinoma.^5^, ^19^, ^20^, ^21^

Inhibition on SHH pathway through targeting SMO has been reported by using two novel SMO inhibitors in MB, Sonidegib (NVP‐LDE225, LDE) and Vismodegib (GDC‐0449, GDC), which have been approved by FDA in basal cell carcinoma and being examined in MB in clinical trials.^22^, ^23^, ^24^, ^25^, ^26^, ^27^, ^28^ Although SMO inhibitors show effectiveness at the beginning for MB, resistance is frequently developed and results in treatment failure. Drug resistance mutation of SMO has been noticed and both 535 Tryptophan (W) mutation and 473 Aspartic acid (D) mutation confer resistance to SMO inhibitor in MB cells.^29^ In addition, SMO inhibitor resistance also accompanies with sustaining activation of PI3K‐AKT‐mTOR axis and blocking this axis partially reverses the resistance toward SMO inhibitors.^30^, ^31^, ^32^, ^33^

Because wild‐type SMO (SMO^WT^) and SMO with oncogenic mutation (SMO^W535L^) show distinct functions during tumor development and drug response, we hypothesized that both mutants should produce different downstream transcriptomes, which might provide new insight on SMO function and regulation. In this study, thereby, we performed RNA‐sequencing on MB cells with SMO^WT^ or SMO^W535L^ followed by profiling metabolome and interactome, and revealed casein kinase II (CK2) as an important mediator for SMO^W535L^.

## 2 MATERIALS AND METHODS

### 2.1 Plasmids, antibodies, and reagents

SMO^WT^ was purchased from Sino Biological (https://www.sinobiological.com/) and SmoM2 (W535L)‐pcw107‐V5 plasmids were purchased from Addgene (http://www.addgene.org/). The SMO expression frame was subcloned into pCDH‐CMV‐MCS‐FE1a‐Puro vector (System Biosciences) with a Flag‐tag at the C‐terminal of SMO. The constructed plasmids were used to package lentivirus in 293T cells according to Kutner's protocol.^34^ Anti‐Flag antibody (#F3165) and anti‐β‐actin antibody (#A5441) were from Sigma Aldrich. MTT kit (#C0009S) was purchased from Beyotime (https://beyotime.com/). SAG (SMO agonist) (#S7779) (0.1 µM), GDC‐0449 (#S1082) (1 µM), NVP‐LDE225 (#S2151) (1 µM), CX4945 (#S0707), and MK2206 (#S1078) were purchased from Selleck (https://www.selleck.cn/).

### 2.2 Cell culture

293T cell, DAOY SHH MB cell, and ONS76 SHH MB cell were purchased from American Type Culture Collection (ATCC, USA). MB726 cell lines derived from freshly resected SHH MB tumors respectively were established in our institute. All cells were cultured in Dulbecco's modification of Eagle's medium (DMEM, Gibico, USA) supplemented with 10% (v/v) fetal bovine serum (FBS, HyClone, USA), 2 mM L‐glutamine, 2 mM sodium pyruvate, 100 U/mL penicillin and 100 µg/mL streptomycin, and maintained in humidified atmosphere containing 5% CO_2_ at 37°C. Cells were treated with SAG, CX4945 (1 μM), MK2206 (0.5 μM), or as indicated in the text.

### 2.3 Western blotting

MB cells were harvested in cold Radio‐Immunoprecipitation Assay (RIPA) buffer (Beyotime Biotech, China) with the protease inhibitor phenylmethanesulfonylfluoride (PMSF, Beyotime Biotech, China) and incubated on ice for 20 min. After centrifugation at 13000 x*g* at 4°C for 20 min, supernatant was collected and measured for protein concentration (BCA protein Assay, Pierce, USA). Equal amounts of proteins (20 µg/well) were separated by 10% sodium dodecyl sulfate–polyacrylamide gel electrophoresis (SDS‐PAGE) and transferred onto polyvinylidene difluoride (PVDF) membranes (Millipore, USA) at 4°C. After block with PBST‐5% skimmed milk block, the membranes were incubated with primary antibodies overnight at 4°C, then were washed and incubated with corresponding secondary HRP‐conjugated antibodies (Beyotime Biotech, China) for 2 h at room temperature. Proteins were visualized with SuperSignal West Femto Maximum Sensitivity Substrate (ECL, Thermo Fisher Scientific) and detected by a ChemiDocXRS system (Bio‐Rad, USA). Primary antibodies used in the study included: anti‐Flag antibody (Sigma Aldrich, #F3165, 1:5000 dilution), anti‐β‐actin antibody (Sigma Aldrich, #A5441, 1:5000 dilution).

### 2.4 RNA sequencing and data processing

#### 2.4.1 Library preparation for Transcriptome sequencing

Total RNA of cells was extracted using TRIzol reagent (15596026 ThermoFisher, USA) according to the manufacturer's instructions. A total amount of 2 μg RNA per sample was used as input material for the RNA sample preparations. Sequencing libraries were generated using NEBNext^®^ UltraTM RNA Library Prep Kit for Illumina^®^ (NEB, USA) following the manufacturer's recommendations and index codes were added to attribute sequences to each sample. Briefly, mRNA was purified from total RNA using poly‐T oligo‐attached magnetic beads. Fragmentation was carried out using divalent cations under elevated temperature in NEBNext First Strand Synthesis Reaction Buffer (5X). First strand cDNA was synthesized using random hexamer primer and M‐MuLV Reverse Transcriptase (RNase H‐). Second strand cDNA synthesis was subsequently performed using DNA Polymerase I and RNase H. Remaining overhangs were converted into blunt ends via exonuclease/polymerase activities. After adenylation of 3′ ends of DNA fragments, NEBNext Adaptor with hairpin loop structure was ligated to prepare for hybridization. In order to select cDNA fragments of preferentially 150–200 bp in length, the library fragments were purified with AMPure XP system (Beckman Coulter, Beverly, USA). Then adaptor‐ligated cDNA at 37°C for 15 min followed by 5 min at 95°C before PCR. Then PCR was performed with Phusion High‐Fidelity DNA polymerase, Universal PCR primers, and Index (X) Primer. At last, PCR products were purified (AMPure XP system) and library quality was assessed on the Agilent Bioanalyzer 2100 system.

#### 2.4.2 Clustering and sequencing

The clustering of the index‐coded samples was performed on a cBot Cluster Generation System using TruSeq PE Cluster Kit v3‐cBot‐HS (Illumia) according to the manufacturer's instructions. After cluster generation, the library preparations were sequenced on an Illumina Hiseq platform and 125 bp/150 bp paired‐end reads were generated.

#### 2.4.3 Quality control

Raw data (raw reads) of fastq format were firstly processed through in‐house perl scripts. In this step, clean data (clean reads) were obtained by removing reads containing adapter, reads containing ploy‐N and low‐quality reads from raw data. At the same time, Q20, Q30, and GC content of the clean data were calculated. All the downstream analyses were based on clean data with high quality.

#### 2.4.4 Reads mapping to the reference genome

Reference genome and gene model annotation files were downloaded from genome website directly. Index of the reference genome was built using Bowtie v2.2.3 and paired‐end clean reads were aligned to the reference genome using Hisat2 v2.0.5. We selected Hisat2 as the mapping tool for that Hisat2 can generate a database of splice sets based on the gene model annotation file and thus a better mapping result than other non‐splice mapping tools.

#### 2.4.5 Quantification of gene expression level

HTSeq was used to count the reads numbers mapped to each gene. And then FPKM of each gene was calculated based on the length of the gene and reads count mapped to this gene. FPKM, expected number of Fragments Per Kilobase of transcript sequence per million base pairs sequenced, considers the effect of sequencing depth and gene length for the reads count at the same time, and is currently the most used method for estimating gene expression levels.^35^

#### 2.4.6 Differential expression analysis

Differential expression analysis was performed using the edgeR package. edgeR is one of the most popular Bioconductor packages for assessing differential expression in RNA‐seq data. It is based on the negative binomial (NB) distribution and it models the variation between biological replicates through the NB dispersion parameter. This method is immediately able to handle complex experimental designs. Genes with an adjusted *p*‐value <0.05 found by DESeq were assigned as differentially expressed.^36^ Differential expression analysis of two conditions was performed using the DEGSeq R package. The *p* values were adjusted using the Benjamini & Hochberg method. Corrected *p*‐value of 0.005 and log_2_(Fold change) of 1 were set as the threshold for significantly differential expression.

### 2.5 Metabolites identification for metabolome

#### 2.5.1 Metabolite extractions

The culture medium from the cultured cells (1 × 10^7^ cells per sample) was removed using pipette. Then the cells were washed with PBS under 37°C and the PBS was removed. 800 μl of cold methanol/acetonitrile (1:1, v/v) was added to remove the protein and extract the metabolites. The mixture was collected into a new centrifuge tube, and centrifuged at 14,000 ***g*** for 5 min at 4°C to collect the supernatant. The supernatant was dried in a vacuum centrifuge. For LC‐MS analysis, the samples were re‐dissolved in 100 μl acetonitrile/water (1:1, v/v) solvent.

#### 2.5.2 LC‐MS analysis

For untargeted metabolomics of polar metabolites, extracts were analyzed using a quadrupole time‐of‐flight mass spectrometer (Sciex TripleTOF 6600) coupled to hydrophilic interaction chromatography via electrospray ionization in Shanghai Applied Protein Technology Co., Ltd. LC separation was on a ACQUIY UPLC BEH Amide column (2.1 mm × 100 mm, 1.7 μm particle size (waters, Ireland) using a gradient of solvent A (25 mM ammonium acetate and 25 mM ammonium hydroxide in water) solvent B (acetonitrile). The gradient was 85% B for 1 min and was linearly reduced to 65% in 11 min, and then was reduced to 40% in 0.1 min and kept for 4 min, and then increased to 85% in 0.1 min, with a 5 min re‐equilibration period employed. Flow rate was 0.4 ml/min, column temperature was 25°C, auto sampler temperature was 5°C, and injection volume was 2 μl. The mass spectrometer was operated in both negative ion and positive ionizations mode. The ESI source conditions were set as follows: Ion Source Gas1 (Gas1) as 60, Ion Source Gas2 (Gas2) as 60, curtain gas (CUR) as 30, source temperature: 600°C, IonSpray Voltage Floating (ISVF) ±5500 V. In MS acquisition, the instrument was set to acquire over the *m/z* range 60–1000 Da, and the accumulation time for TOF MS scan was set at 0.20 s/spectra. In auto MS/MS acquisition, the instrument was set to acquire over the *m/z* range 25–1000 Da, and the accumulation time for product ion scan was set at 0.05 s/spectra. The product ion scan is acquired using information‐dependent acquisition (IDA) with high sensitivity mode selected. The parameters were set as follows: the collision energy (CE) was fixed at 35 V with ±15 eV; declustering potential (DP), 60 V (+) and −60 V (−); exclude isotopes within 4 Da, candidate ions to monitor per cycle: 10.

#### 2.5.3 Data analysis

The raw MS data were converted to MzXML files using ProteoWizard MSConvert before importing them into freely available XCMS software. For peak picking, the following parameters were used: centWave *m/z* = 25 ppm, peakwidth = c (10, 60), prefilter = c (10, 100). For peak grouping, bw = 5, mzwid = 0.025, minfrac = 0.5 were used. In the extracted ion features, only the variables having more than 50% of the nonzero measurement values in at least one group were kept. Compound identification of metabolites by MS/MS spectra with an in‐house database established with available authentic standards. After normalized to total peak intensity, the processed data were uploaded before importing into SIMCA‐P (version 14.1, Umetrics, Umea, Sweden), where it was subjected to multivariate data analysis, including Pareto‐scaled principal component analysis (PCA) and orthogonal partial least‐squares discriminant analysis (OPLS‐DA). The 7‐fold cross‐validation and response permutation testing were used to evaluate the robustness of the model. The variable importance in the projection (VIP) value of each variable in the OPLS‐DA model was calculated to indicate its contribution to the classification. Significance was determined using an unpaired Student's *t* test. VIP value >1 and *p *< 0.05 were considered as statistically significant.

### 2.6 Co‐immunoprecipitation‐Mass‐spectrum (MS) for interactome

Immunoprecipitation (IP) was performed using IP kit (#26149, Thermo Scientific, USA) following the manufacturer's protocol. Briefly, 10 µg Flag antibody and IgG were immobilized on AminoLink Plus coupling resin for 2 h, respectively. 250 µg MB cell lysate was added and incubated at 4°C overnight. Then the resin was washed and eluted using an elution buffer. The eluted proteins were sent to The Central Laboratory of the Army Medical University for MS.

### 2.7 MTT assay

5000 cells were seeded in a 96‐well plate for overnight growth and then inhibitors with series concentrations were added into the wells with the presence of SAG plus DMSO or SAG plus SMO inhibitors. After 72 h growth, the cell viability was measured through MTT assay as per the manufacturer's instruction and the IC_50_ was calculated using GraphPad Prism 5 software.

### 2.8 Mouse orthotopic models and drug treatments

Mice were purchased from the Laboratory Animal Center, Army Medical University (Chongqing, China). To establish mouse orthotopic models, ONS76‐SMO^W535L^ cells were injected into the cerebellum of 6‐week‐old NOD‐SCID mice at 1 × 10^5^ cells per mouse (*n* = 8). The mouse survival was observed for up to all the animal dead. On the 7th day after implantation, animals were intraperitoneally injected with CX4945 (20 mg/kg), MK2206 (10 mg/kg), or both inhibitors once per week, for 4 weeks. Control groups were given identical volume of vehicle instead of inhibitors. Xenograft growth was monitored through bioluminescent imaging on Day 16, Day 23, Day 30, and Day 37 using In Vivo Imaging System (IVIS) Spectrum (Perkin‐Elmer). The animal experiments were approved by the Institutional Animal Care and Use Committee of Army Medical University. All animal data reporting in this study followed the ARRIVE 2.0 guidelines.^37^

### 2.9 Statistics

All experiments were conducted at least three times and each treatment was set up in triplicate, unless specially indicated otherwise. Results were presented as the mean ± standard deviation (SD). The statistical significance between testing and control groups was analyzed with SPSS20.0 statistical software and GraphPad Prism 9.2.0. The significance was determined by a two‐tailed unpaired Student's *t* test. Kaplan–Meier survival plots and log‐rank statistics were used to evaluate the survival of mouse model. A *p* value <0.05 was considered statistically significant.

## 3 RESULTS

### 3.1 Transcriptomic features of wild‐type and mutant SMO with or without SMO inhibitors

**FIGURE 1:**
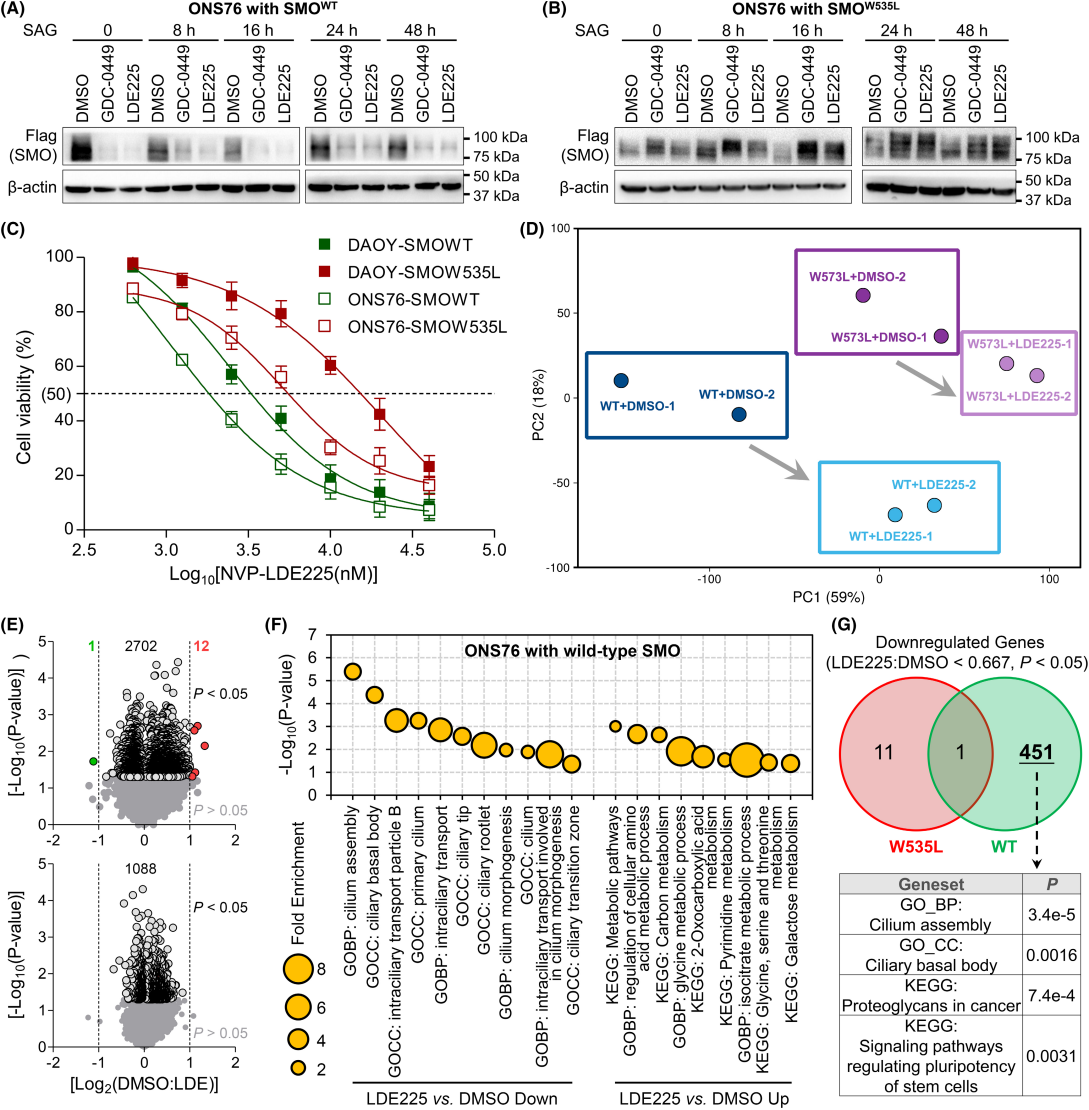
Transcriptomic analysis on MB cells with SMO^WT^ or SMO^W535L^. (A) Western blotting of MB cells expressing flag tagged SMO^WT^ treated with indicated reagents. (B) Western blotting of MB cells expressing flag tagged SMO^W535L^ treated with indicated reagents. (C) IC_50_ of LDE225 in MB cells with SMO^WT^ or SMO^W535L^. (D) PCA on transcriptomes of SMO^WT^ or SMO^W535L^ MB cells treated with indicated reagents. (E) Volcano graph of altered genes by LDE225 *vs*. DMSO in MB cells with SMO^WT^ (Upper panel) or SMO^W535L^ (Lower panel). (F) Geneset enrichment in the context of GO and KEGG terms for genes altered by LDE225 *vs*. DMSO in MB cells with SMO^WT^. (G) Venn diagram of significantly downregulated genes by LDE225 *vs*. DMSO and gene enrichment in the context of GO and KEGG terms

We first checked the influence of SMO inhibitors on SMO protein level. For this purpose, we established MB cells with stable expression of wild‐type SMO (SMO^WT^) or W535L mutant SMO (SMO^W535L^). Since SMO^W535L^ mutation is an activating mutation of SMO and it behaves in a dominant manner, the endogenously expressed WT form of SMO protein did not affect the results of SMO^W535L^ in our model. Interestingly, we noticed that protein level of SMO^WT^ was significantly decreased with GDC or LDE (Figure 1A). However, protein level of SMO^W535L^ was slightly increased with the treatment of SMO inhibitors (Figure 1B). Although the mechanisms of SMO^WT^ activation remain confusing, it has been reported that the activation usually requires endocytosis and recycling but undergoes ubiquitination‐mediated proteasome degradation without activation.^14^, ^15^, ^16^ Consistently, we observed that NVP‐LDE225 and GDC‐0449 obviously decreased protein level of SMO^WT^, but protein level of SMO^W535L^ was not inhibited by the two inhibitors. Thus, our result implied that SMO^WT^ was sensitive but SMO^W535L^ was resistant to SMO inhibitors. MTT assays further showed that IC_50_ of LDE was lower in SMO^WT^ MB cells than in SMO^W535L^ MB cells (Figure 1C). Then, we profile transcriptomes of the stable cells with or without SMO inhibitor through RNA‐sequencing (Dataset S1). The result confirmed that MB cells with SMO^WT^ were more sensitive than SMO^W535L^ to SMO inhibitor. PCA indicated that SMO inhibitor changed transcriptomic profile of SMO^WT^ much more than that of SMO^W535L^ (Figure 1D). Transcriptomic analysis further showed that 2715 genes in SMO^WT^ were altered with statistical significance (*p* < 0.05) and 1088 genes in SMO^W535L^ were altered with statistical significance (*p* < 0.05; Figure 1E). Additionally, 12 genes were upregulated and 1 downregulated gene more than 2 folds of DMSO group *vs*. SMO inhibitor group in SMO^WT^ cells, but no genes were changed more than 2 folds of DMSO group *vs*. SMO inhibitor group in SMO^W535L^ cells (Figure 1E). It was noted that PIK3C2A, a key regulator of PI3K/AKT, was among the top‐ranked downregulated genes by LDE225 in SMO^WT^ cells but not changed by LDE225 in SMO^W535L^ cells (Dataset S1), suggesting the tight correlation of Hedgehog signaling pathway and PI3K/AKT signaling pathway. Moreover, downregulated genes by LDE225 *vs*. DMSO in MB cells with SMO^WT^ enriched cilium‐related genesets (Figure 1F and Table S1) but not in MB cells with SMO^W535L^ (Table S2). However, upregulated genes by LDE225 *vs*. DMSO in MB cells with SMO^WT^ enriched metabolism‐related genesets (Figure 1F and Table S1), which was not detected in MB cells with SMO^W535L^ (Table S2). Venn diagram analysis showed that most of downregulated genes by SMO inhibitor in SMO^WT^ MB cells were not observed in SMO^W535L^ MB cells and enriched genesets related to cilia and cancer pathways (Figure 1G and Table S3). In addition, LDE225 could effectively downregulate PI3K‐AKT signaling pathway and mTOR signaling pathway (Table S1), two well‐known pathways involved in non‐canonical HH pathway,^30^, ^31^, ^32^, ^33^ which did not change in SMO^W535L^ MB cells treated by LDE225 (Table S2). Thus, SMO inhibitor effectively suppressed MB cells with SMO^WT^ but not those with SMO^W535L^.

### 3.2 Metabolomic features of SMO^WT^ and SMO^W535L^ with or without SMO inhibitors

**FIGURE 2:**
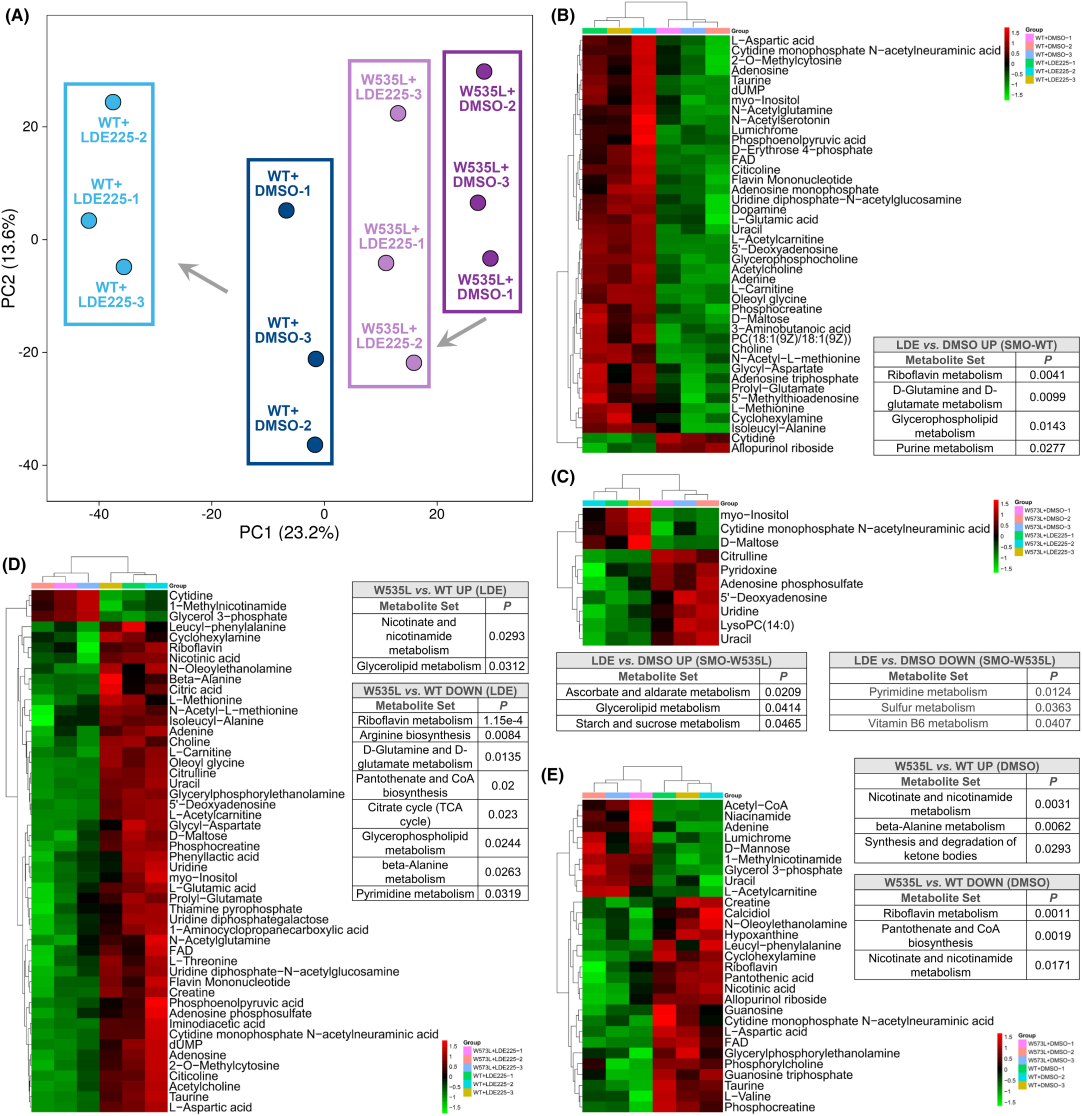
Metabolomic analysis on MB cells with SMO^WT^ or SMO^W535L^. (A) PCA on metabolomes of SMO^WT^ or SMO^W535L^ MB cells treated with indicated reagents. (B)–(E) KEGG analysis on significantly changed metabolites in different comparison groups

Transcriptomic analysis revealed that SMO inhibitor led to alteration of a series of metabolism pathways and metabolism reprogramming has been observed and druggable in MB,^38^, ^39^, ^40^ which promoted us to investigate the metabolome of MB cells with or without SMO inhibitor (Table S4). PCA result consistently showed that SMO^WT^ was more sensitive to SMO inhibitor than SMO^W535L^ in metabolite variations (Figure 2A). In SMO^WT^ cells, 42 metabolites were significantly affected by LDE225 (Figure 2B), but only 10 metabolites were changed by LDE225, in comparison with DMSO (Figure 2C). Metabolite analysis through www.metaboanalyst.ca indicated that Riboflavin metabolism, D‐Glutamine and D‐glutamate metabolism, glycerophospholipid metabolism, and purine metabolism were enriched by LDE225‐upregulated metabolites in SMO^WT^ cells (Figure 2B). In SMO^W535L^ cells, LDE225 increased ascorbate and aldarate metabolism, glycerolipid metabolism, and starch and sucrose metabolism, but decreased pyrimidine metabolism, sulfur metabolism, and vitamin B6 metabolism (Figure 2C). Then, we combined transcriptomic data with metabolic data for analysis using KEGG reference metabolic pathways. Interestingly, in LDE225‐treated SMO^WT^ cells, glycine, serine, and threonine metabolism (www.kegg.jp/pathway/map00260) (identified in transcriptome data) was directly associated with D‐glutamine and D‐glutamate metabolism or D‐amino acid metabolism, glycerophospholipid metabolism, and purine metabolism (identified in metabolism data). Purine metabolism (www.kegg.jp/pathway/map00230) was further linked to riboflavin metabolism. Hence, glycine, serine, and threonine metabolism might play a central role for metabolite regulation in LDE225‐treated SMO^WT^ cells. In SMO^W535L^ cells, however, both transcriptomic and metabolic data revealed pyrimidine metabolism was downregulated by LDE225 treatment. We also compared the metabolome of SMO^W535L^ cells to that of SMO^WT^ cells with LDE225 or DMSO (Figure 2D and E). Only nicotinate and nicotinamide metabolism and glycerolipid metabolism were increased in SMO^W535L^ cells treated with LDE225. Arginine biosynthesis, D‐glutamine and D‐glutamate metabolism, citrate cycle (TCA cycle), glycerophospholipid metabolism, beta‐alanine metabolism, and pyrimidine metabolism were decreased by LDE225 in SMO^W535L^ cells compared with SMO^WT^ cells (Figure 2D). Moreover, we noticed that glycerolipid metabolism was enhanced in SMO^W535L^ cells treated with LDE225 *vs*. DMSO and also enhanced by LDE225 in SMO^W535L^ cells *vs*. SMO^WT^ (Figure 2C and D), implying that this metabolism pathway was critical for SMO^W535L^ cells. Thus, the metabolism of SMO^WT^ cells was more vulnerable to SMO inhibitor treatment than SMO^W535L^ cells, and both glycine, serine and threonine metabolism and glycerolipid metabolism were associated with SMO^W535L^ cells.

### 3.3 Interactomic features of SMO^WT^ and SMO^W535L^ with or without SMO inhibitors

**FIGURE 3:**
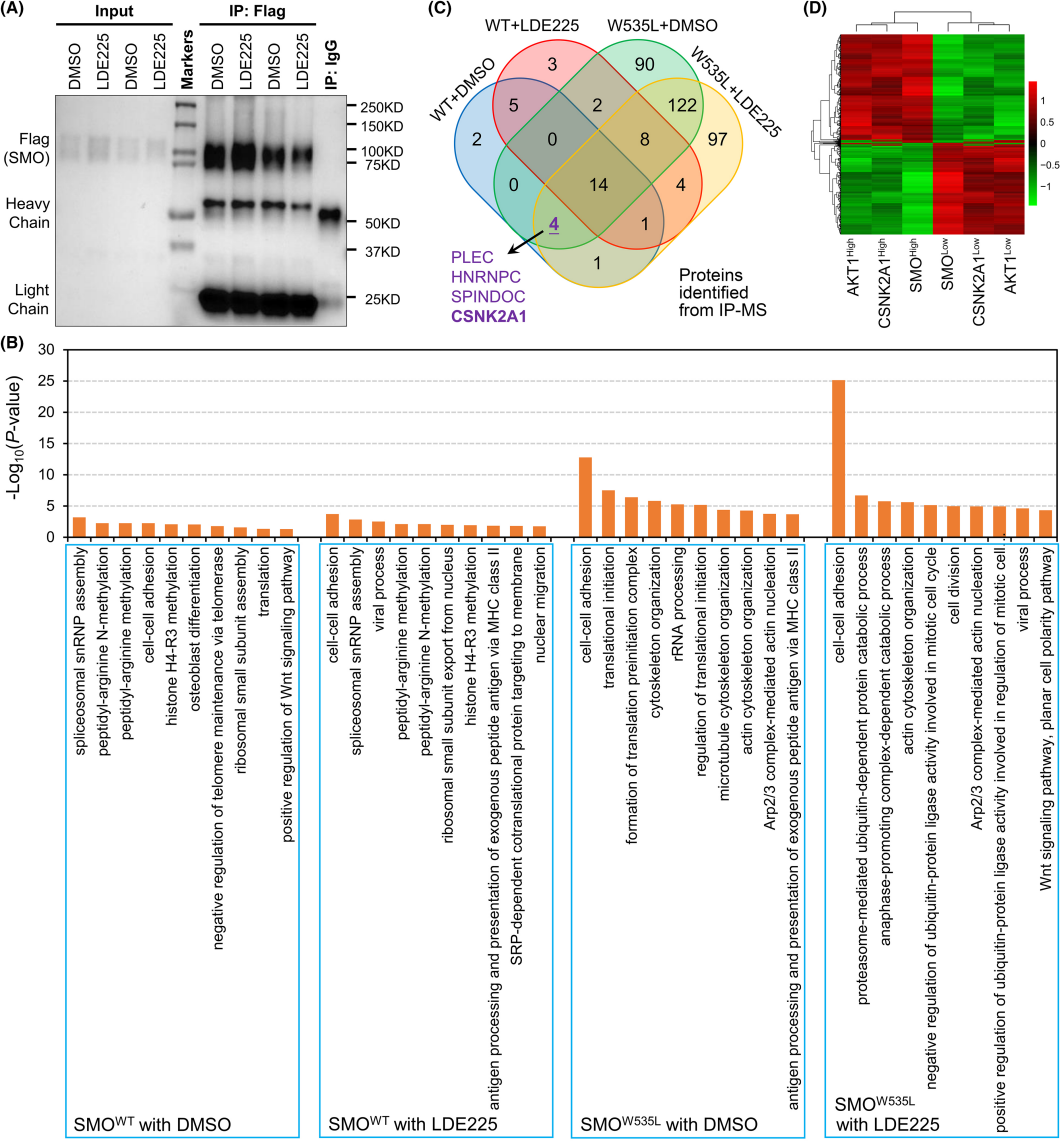
Interactomic analysis on MB cells with SMO^WT^ or SMO^W535L^. (A) IP‐western confirmation of SMO expression subjected to MASS‐Spectrum. (B) Geneset enrichment in the context of KEGG term for proteins interacted with SMO^WT^ or SMO^W535L^ in the indicated treatment condition. (C) Venn diagram of proteins interacted with SMO^WT^ or SMO^W535L^ in the indicated treatment condition. (D) Heatmap cluster analysis on transcriptomes of high and low expression of SMO, AKT, and CK2, respectively, using SHH‐subtype MB from GSE85217 dataset

According to transcriptomic and metabolomic analyses, we found that SMO^W535L^ remained activated in the presence of LDE225. Thus, we investigated the binding proteins with SMO. ONS76 cells with SMO^WT^‐Flag or SMO^W535L^‐Flag were pretreated with SAG followed by treatment of DMSO or LDE225. Then, the cells were subjected to immunoprecipitation using anti‐Flag antibody (Figure 3A) and MS identification. The MS result showed that more interacting proteins were identified for SMO^W535L^ than SMO^WT^ in both LDE225 treatment and DMSO treatment (Table S5). GO_BP (Biological Process) analysis through David software (Table S6) showed that SMO^WT^‐interacting complex highly included methylation‐related proteins but SMO^W525L^‐interacting complex highly included cytoskeleton proteins (Figure 3B). We speculated that critical SMO function mediators should interact with SMO^WT^ without LDE225 treatment but fail to do so with LDE225 treatment. However, these mediators could interact with SMO^W535L^ in spite of LDE225 treatment. According to this criterion, we found PLEC, HNRNPC, SPINDOC, and CSNK2A1 as potential SMO function mediators through Venn diagram analysis (Figure 3C). Interestingly, CSNK2A1, a gene encoding CK2, has been known to play critical roles in the regulation of DNA damage repair and metabolism processes and promote glycerolipid metabolism,^41^ which were significantly enriched by SMO^W535L^. since AKT has been known related with SMO constitutive mutation and our data further showed CK2 was also related with SMO^W535L^. We further analyzed the relationships among SMO, AKT, and CK2 using SHH‐subtype MB from GSE85217 dataset.^42^ Heatmap cluster analysis showed that transcriptomes of high expression of SMO, AKT, and CK2 could be clustered together and those of low expression of the three genes also fell into one cluster (Figure 3D). Thus, CK2 might be pivotally involved in SMO^W535L^ signaling pathways.

### 3.4 Synergetic effects of CK2 inhibitor and AKT inhibitor on the growth of MB cells with SMO^W535L^

**FIGURE 4:**
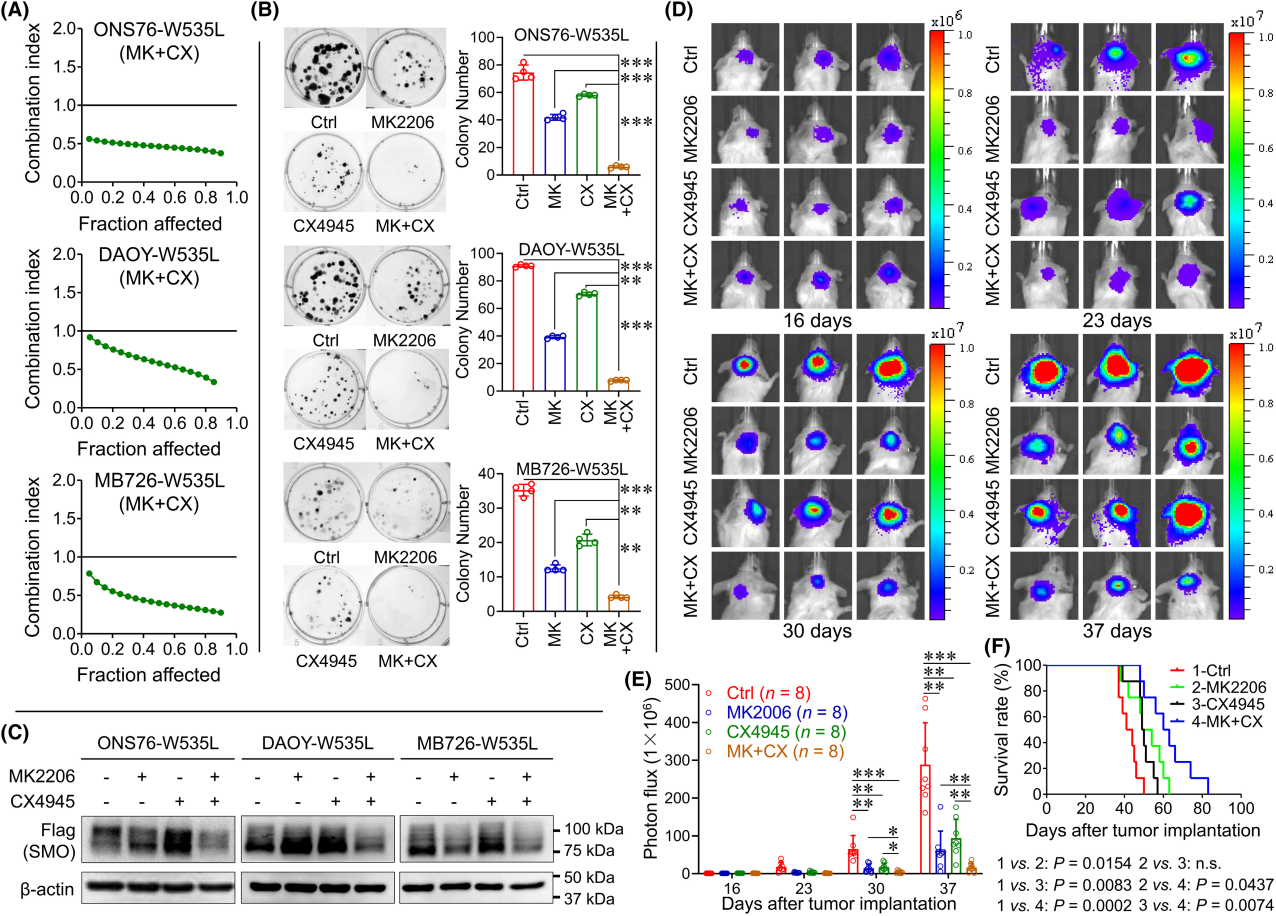
Synergetic effects of CX4945 and MK2206 for MB cells with SMO^W535L^. (A) Measurement of combination index of CX4945 plusMK2206 in three MB cells with SMO^W535L^. (B) Colony formation of three MB cells with SMO^W535L^ in treatment of CX4945 or/and MK2206. The data are shown as mean ± SD; ***p < 0.001; **p < 0.01; *n* = 4 per group. (C) Western blotting of MB cells expressing flag tagged SMO^W535L^ treated with indicated reagents. (D) Representative images of orthotopic growth of ONS76 cells with SMO^W535L^ treated with vehicle, CX4945, MK2206, or MK+CX). (E) Statistic graph of tumor size using bioluminescence signal intensity. The data are shown as mean ± SD; ***p < 0.001; **p < 0.01; *n* = 8 per group. (F) Kaplan–Meier survival analysis of mice treated with indicated conditions. *n* = 8 per group

Our current study revealed CK2 as a potential mediator for SMO signaling and AKT pathway has been known to mediate HH pathway in SMO inhibitor‐resistant cells. Therefore, we evaluated the combining effects of CK2 inhibitor (CX4945) and AKT inhibitor (MK2206) on MB cells with SMO^W535L^. CX‐4945 is the first selective orally bioavailable CK2 inhibitor to advance into human clinical trials.^43^, ^44^ We evaluated the inhibitory effects of different chemical combinations through MTT assay according to CompuSyn software.^45^ In three MB cell lines with SMO^W535L^, we consistently observed synergetic effects of CX4945 and MK2206 (Figure 4A). Colony formation assay indicated that individual application of CX4945 or MK2206 inhibited the growth of the three SMO^W535L^ MB cells but the combination of the two inhibitors showed a more potent inhibitory effect on these cells than each inhibitor (Figure 4B). Western blotting suggested that a combination of the two inhibitors could remarkably decrease protein levels of SMO^W535L^ (Figure 4C). To further examine the therapeutic effects of the two inhibitors, we orthotopically inoculated ONS76‐SMO^W535L^ cells into mouse cerebellum (Figure 4D). At 16, 23, 30, and 37 days after inoculation, we calculated tumor size using bioluminescent reads. The data showed that combination of the two inhibitors effectively suppressed tumor growth *in vivo* in comparison with individual application of the inhibitors (Figure 4E). Kaplan‐Meier survival plots and log‐rank statistics also suggested that the combination of CX4945 and MK2206 significantly improved the survival of mice with xenografts of ONS76‐SMO^W535L^ cells (Figure 4F). Together, targeting CK2 and AKT represented a promising therapeutic strategy for SMO inhibitor‐resistant MB.

## 4 DISCUSSION

In this study, we, for the first time, profiled transcriptomes, metabolomes, and interactomes of MB cells with SMO^WT^ or constitutively activated SMO^W535L^ in the context of DMSO or FDA‐approved SMO inhibitor, respectively. Our findings revealed that PI3K‐AKT‐mTOR axis and metabolic pathways were regulated by SMO^W535L^ and concurrently targeting CK2 and AKT potently repressed in vitro and in vivo growth of MB with SMO^W535L^.

Analysis on transcriptomic data indicated that SMO inhibitor disrupted processes of endocytosis and cilium organization in MB cells with SMO^WT^, which are necessary for SMO activation. In MB cells with SMO^W535L^; however, SMO inhibitor did not affect the two processes‐related genes, implying resistance of SMO^W535L^ toward SMO inhibitor. Interestingly, it was noted that SMO inhibitor significantly inhibited metabolism‐related pathways. SHH/SMO axis has been found to play important role in the metabolism of lipid, cholesterol, amino acids, and glucose.^46^, ^47^, ^48^, ^49^, ^50^, ^51^, ^52^, ^53^ Our metabolic analysis further supported the alterations of metabolites due to SMO inhibition in MB cells with SMO^WT^ but not in MB cells with SMO^W535L^. Through integrating transcriptomic and metabolic findings, we found that SMO inhibitor mainly affected amino acid metabolisms, such as glycine, serine and threonine metabolism and D‐glutamine and D‐glutamate metabolism in SMO^WT^ cells. Thus, we speculated that SMO inhibitor might function through interfering with amino acid metabolism in sensitive MB cells, which implied a potential monitor target to predict SMO inhibitor resistance for MB. Moreover, we found that glycerolipid metabolism was stronger in SMO^W535L^ cells than SMO^WT^ cells with both DMSO and SMO inhibitor treatment. KEGG reference pathway analysis showed that glycine, serine, and threonine (www.kegg.jp/pathway/map00260)–glycerophospholipid (www.kegg.jp/pathway/map00564) metabolism axis is associated with glycolysis in SMO^WT^ cells but glycerolipid metabolism (www.kegg.jp/pathway/map00561) was correlated with glycolysis in SMO^W535L^ cells. The glycolysis has been known to be related with the activation of hedgehog pathway.^38^, ^51^, ^54^ Thus, switching from glycine, serine and threonine‐related glycolysis to glycerolipid‐mediated glycolysis might be involved in the development of SMO inhibitor resistance of MB cells. Additionally, our findings newly indicated that nicotinate and nicotinamide metabolism, glycerolipid metabolism, beta‐alanine metabolism, and synthesis and degradation of ketone bodies might be involved in SMO^W535L^ function maintenance.

Interactomic analysis revealed CK2 as an important SMO‐associated protein. CK2 composes of two catalytic (CK2α) and two regulatory subunits (CK2β) and functions as protein kinase, critically involved in maintaining cell survival through pro‐proliferative, anti‐apoptotic, and pro‐angiogenic signaling.^55^, ^56^, ^57^ Aberrant activation and expression of CK2 has been demonstrated in many types of cancers.^57^ Upregulation of CK2 correlates with poor cancer prognosis in numerous cancers including cervical, gastric, liver, head and neck cancers, and adult gliomas.^58^, ^59^, ^60^ CK2 inhibition is also found to sensitize medulloblastoma to temozolomide.^61^ CK2 has been known to phosphorylate and activate SMO^WT^ and is a potential therapeutic target for MB.^62^, ^63^

Because most of the patients with MB are children, regular surgery and radio‐chemotherapy have to be cautious to avoid severe brain damage and long‐term side effects. Targeted therapy is a hopeful strategy for young patients due to its specificity. In this study, we linked CK2 and AKT together and found combination of inhibitors targeting CK2 and AKT showed synergetic effects to inhibit the growth of MB cells with SMO constitutive activation mutation.

## 5 CONCLUSION

Altogether, our work described SMO‐related transcriptomes, metabolomes, and interactomes under different SMO status and treatment conditions and revealed CK2 and AKT as therapeutic targets for SHH‐subtype MB cells with SMO inhibitor resistance.

## CONFLICTS OF INTEREST

The authors declare that they have no conflict of interest.

## AUTHOR CONTRIBUTION

Designing research studies: Y.W., Y.‐X.W., and Y.‐L.Y.; Conducting experiments: Y.‐L.Y., Y.‐X.W., F.‐C.Y., C.W., M.M., Q.‐J.G., J.Z., and J.H.; Acquiring data: Y.Q., X.‐X.Y., X.L., H.Z., and H.‐M.L.; Analyzing data: Y.W., X.‐W.B., and X.‐H.Y.; Writing the manuscript: Y.W.. All authors have read and approved the article.

## Supporting information

Table S1‐S6

Supplementary Material

## Data Availability

All data are available in the manuscript and its supplemental materials.
