# Supplementary material for: Targeting AKT and CK2 represents a novel therapeutic strategy for SMO constitutive activation‐driven medulloblastoma
Source: CNS Neurosci Ther. 2022 Apr 14;28(7):1033–44. doi: 10.1111/cns.13835 (PMC9160449; doi:10.1111/cns.13835)
Supplement: Supplementary file 1 — Table S1‐S6 [file CNS-28-1033-s002.pdf]

Table S1. Geneset enrichment analysis using genes altered by LDE225 *vs.* DMSO in ONS76 with SMO^{WT}.

Genesets enriched by downregulated genes from LDE225 *vs.* DMSO							Genesets enriched by upregulated genes from LDE225 *vs.* DMSO					
Category	Term	Count	%	*P* Value	Fold Enrichment		Category	Term	Count	%	*P* Value	Fold Enrichment
GO_BP	regulation of transcription, DNA-templated	215	13.2226	0.0000	1.6857		GO_BP	rRNA processing	44	4.0930	0.0000	3.6002
GO_BP	transcription, DNA-templated	248	15.2522	0.0000	1.4959		GO_BP	translation	48	4.4651	0.0000	3.3220
GO_BP	cilium assembly	28	1.7220	0.0000	2.6627		GO_BP	nuclear-transcribed mRNA catabolic process, nonsense-mediated decay	28	2.6047	0.0000	4.1200
GO_BP	RNA splicing	31	1.9065	0.0001	2.2021		GO_BP	translational initiation	29	2.6977	0.0000	3.7065
GO_BP	cellular response to DNA damage stimulus	36	2.2140	0.0001	2.0409		GO_BP	mitochondrial translational elongation	22	2.0465	0.0000	4.5320
GO_BP	ubiquitin-dependent protein catabolic process	32	1.9680	0.0001	2.0733		GO_BP	SRP-dependent cotranslational protein targeting to membrane	23	2.1395	0.0000	4.2843
GO_BP	regulation of transcription from RNA polymerase II promoter	61	3.7515	0.0002	1.6311		GO_BP	mRNA splicing, via spliceosome	35	3.2558	0.0000	2.7606
GO_BP	cell-cell adhesion	42	2.5830	0.0002	1.8276		GO_BP	mitochondrial translational termination	20	1.8605	0.0000	4.0721
GO_BP	protein ubiquitination involved in ubiquitin-dependent protein catabolic process	28	1.7220	0.0002	2.1580		GO_BP	viral transcription	23	2.1395	0.0000	3.5958
GO_BP	protein ubiquitination	51	3.1365	0.0003	1.6752		GO_BP	ribosomal small subunit assembly	9	0.8372	0.0000	8.2942
GO_BP	fat cell differentiation	17	1.0455	0.0003	2.7461		GO_BP	cell-cell adhesion	34	3.1628	0.0000	2.1968
GO_BP	pre-miRNA processing	7	0.4305	0.0004	6.3496		GO_BP	NIK/NF-kappaB signaling	14	1.3023	0.0001	3.7142
GO_BP	mitotic nuclear division	38	2.3370	0.0005	1.8069		GO_BP	cell division	38	3.5349	0.0002	1.9011
GO_BP	mRNA splicing, via spliceosome	35	2.1525	0.0005	1.8591		GO_BP	mitochondrial electron transport, NADH to ubiquinone	11	1.0233	0.0004	3.9308
GO_BP	protein K48-linked deubiquitination	9	0.5535	0.0006	4.4221		GO_BP	mitochondrion organization	14	1.3023	0.0004	3.1836
GO_BP	negative regulation of transcription, DNA-templated	64	3.9360	0.0009	1.5124		GO_BP	anaphase-promoting complex-dependent catabolic process	14	1.3023	0.0005	3.1030
GO_BP	ER to Golgi vesicle-mediated transport	27	1.6605	0.0009	1.9899		GO_BP	protein targeting to mitochondrion	9	0.8372	0.0005	4.6350
GO_BP	endosome organization	10	0.6150	0.0010	3.6850		GO_BP	positive regulation of t-circle formation	4	0.3721	0.0007	17.5099
GO_BP	in utero embryonic development	30	1.8450	0.0011	1.8918		GO_BP	antigen processing and presentation of exogenous peptide antigen via MHC class I, TAP-dependent	12	1.1163	0.0008	3.3352
GO_BP	cell division	48	2.9520	0.0011	1.6172		GO_BP	intrinsic apoptotic signaling pathway in response to DNA damage by p53 class mediator	8	0.7442	0.0015	4.5187
GO_BP	Golgi to plasma membrane protein transport	9	0.5535	0.0013	3.9307		GO_BP	negative regulation of fibroblast proliferation	8	0.7442	0.0015	4.5187

GO_BP	intraciliary transport	7	0.4305	0.0014	5.1591		GO_BP	t-circle formation	4	0.3721	0.0017	14.0079
GO_BP	endocytosis	24	1.4760	0.0014	2.0361		GO_BP	regulation of mRNA stability	15	1.3953	0.0021	2.5500
GO_BP	COPII vesicle coating	14	0.8610	0.0015	2.7064		GO_BP	regulation of cellular amino acid metabolic process	10	0.9302	0.0022	3.4333
GO_BP	protein localization to plasma membrane	14	0.8610	0.0021	2.6205		GO_BP	negative regulation of ubiquitin-protein ligase activity involved in mitotic cell cycle	12	1.1163	0.0022	2.9594
GO_BP	pexophagy	4	0.2460	0.0023	11.7921		GO_BP	mitochondrial respiratory chain complex I assembly	11	1.0233	0.0028	3.0573
GO_BP	mRNA processing	28	1.7220	0.0024	1.8446		GO_BP	canonical glycolysis	7	0.6512	0.0029	4.7142
GO_BP	positive regulation of transcription from RNA polymerase II promoter	109	6.7036	0.0026	1.3102		GO_BP	positive regulation of ubiquitin-protein ligase activity involved in regulation of mitotic cell cycle transition	12	1.1163	0.0037	2.7647
GO_BP	protein transport	51	3.1365	0.0027	1.5225		GO_BP	regulation of mitochondrial membrane potential	7	0.6512	0.0043	4.3775
GO_BP	post-embryonic development	15	0.9225	0.0030	2.4230		GO_BP	protein stabilization	17	1.5814	0.0047	2.1887
GO_BP	visual learning	11	0.6765	0.0038	2.8825		GO_BP	exonucleolytic nuclear-transcribed mRNA catabolic process involved in deadenylation-dependent decay	7	0.6512	0.0052	4.2265
GO_BP	heart morphogenesis	9	0.5535	0.0043	3.3165		GO_BP	RNA catabolic process	6	0.5581	0.0057	5.0028
GO_BP	protein retention in Golgi apparatus	4	0.2460	0.0053	9.4337		GO_BP	proteasome-mediated ubiquitin-dependent protein catabolic process	22	2.0465	0.0061	1.8976
GO_BP	establishment of protein localization	10	0.6150	0.0054	2.9480		GO_BP	intrinsic apoptotic signaling pathway	7	0.6512	0.0062	4.0856
GO_BP	chromatin remodeling	16	0.9840	0.0055	2.1939		GO_BP	antigen processing and presentation of peptide antigen via MHC class I	7	0.6512	0.0062	4.0856
GO_BP	hippo signaling	8	0.4920	0.0061	3.4940		GO_BP	platelet aggregation	8	0.7442	0.0079	3.4166
GO_BP	epidermal growth factor receptor signaling pathway	12	0.7380	0.0066	2.5269		GO_BP	negative regulation of endoplasmic reticulum calcium ion concentration	4	0.3721	0.0083	8.7550
GO_BP	positive regulation of proteasomal ubiquitin-dependent protein catabolic process	13	0.7995	0.0069	2.3953		GO_BP	positive regulation of IRE1-mediated unfolded protein response	4	0.3721	0.0083	8.7550
GO_BP	positive regulation of transcription, DNA-templated	61	3.7515	0.0071	1.3967		GO_BP	positive regulation of canonical Wnt signaling pathway	15	1.3953	0.0084	2.1887
GO_BP	RNA processing	17	1.0455	0.0073	2.0667		GO_BP	release of cytochrome c from mitochondria	6	0.5581	0.0085	4.5678
GO_BP	vesicle organization	8	0.4920	0.0075	3.3692		GO_BP	protein polyubiquitination	20	1.8605	0.0090	1.9033
GO_BP	definitive hemopoiesis	6	0.3690	0.0086	4.4221		GO_BP	extrinsic apoptotic signaling pathway	8	0.7442	0.0090	3.3352
GO_BP	protein K63-linked deubiquitination	7	0.4305	0.0105	3.5889		GO_BP	transcription-coupled nucleotide-excision repair	11	1.0233	0.0091	2.6028
GO_BP	cilium morphogenesis	21	1.2915	0.0106	1.8208		GO_BP	antigen processing and presentation of endogenous peptide antigen via MHC class I via ER pathway, TAP-	3	0.2791	0.0094	17.5099

								independent				
GO_BP	Golgi to plasma membrane transport	6	0.3690	0.0113	4.1619		GO_BP	termination of RNA polymerase II transcription	10	0.9302	0.0101	2.7359
GO_BP	protein phosphorylation	54	3.3210	0.0116	1.3964		GO_BP	ribosomal small subunit biogenesis	5	0.4651	0.0110	5.4718
GO_BP	protein polyubiquitination	26	1.5990	0.0126	1.6663		GO_BP	fatty acid beta-oxidation	8	0.7442	0.0116	3.1836
GO_BP	snRNA transcription from RNA polymerase II promoter	13	0.7995	0.0139	2.1900		GO_BP	antigen processing and presentation of exogenous peptide antigen via MHC class I, TAP-independent	4	0.3721	0.0120	7.7822
GO_BP	covalent chromatin modification	18	1.1070	0.0142	1.8784		GO_BP	glycine metabolic process	4	0.3721	0.0120	7.7822
GO_BP	positive regulation of GTPase activity	64	3.9360	0.0146	1.3357		GO_BP	RNA phosphodiester bond hydrolysis, endonucleolytic	9	0.8372	0.0122	2.8653
GO_BP	cytoskeleton-dependent intracellular transport	6	0.3690	0.0146	3.9307		GO_BP	viral process	28	2.6047	0.0124	1.6397
GO_BP	protein targeting	9	0.5535	0.0150	2.7213		GO_BP	ribosome biogenesis	7	0.6512	0.0152	3.4047
GO_BP	microtubule cytoskeleton organization	13	0.7995	0.0155	2.1591		GO_BP	regulation of translational initiation	7	0.6512	0.0152	3.4047
GO_BP	regulation of cell morphogenesis	7	0.4305	0.0160	3.3018		GO_BP	mitochondrial translation	7	0.6512	0.0152	3.4047
GO_BP	RNA 3'-end processing	4	0.2460	0.0164	6.7384		GO_BP	antigen processing and presentation of exogenous peptide antigen via MHC class II	12	1.1163	0.0155	2.2839
GO_BP	miRNA loading onto RISC involved in gene silencing by miRNA	4	0.2460	0.0164	6.7384		GO_BP	Wnt signaling pathway, planar cell polarity pathway	12	1.1163	0.0155	2.2839
GO_BP	intraciliary transport involved in cilium morphogenesis	4	0.2460	0.0164	6.7384		GO_BP	DNA repair	23	2.1395	0.0155	1.7137
GO_BP	posttranscriptional gene silencing by RNA	4	0.2460	0.0164	6.7384		GO_BP	negative regulation of hydrogen peroxide-induced cell death	4	0.3721	0.0164	7.0040
GO_BP	miRNA metabolic process	4	0.2460	0.0164	6.7384		GO_BP	RNA methylation	4	0.3721	0.0164	7.0040
GO_BP	intracellular protein transport	31	1.9065	0.0165	1.5490		GO_BP	tumor necrosis factor-mediated signaling pathway	14	1.3023	0.0170	2.0774
GO_BP	cytoskeleton organization	23	1.4145	0.0173	1.6846		GO_BP	retrograde vesicle-mediated transport, Golgi to ER	11	1.0233	0.0180	2.3489
GO_BP	protein N-linked glycosylation via asparagine	9	0.5535	0.0174	2.6532		GO_BP	extrinsic apoptotic signaling pathway via death domain receptors	7	0.6512	0.0196	3.2255
GO_BP	termination of RNA polymerase II transcription	12	0.7380	0.0178	2.2110		GO_BP	regulation of defense response to virus by virus	6	0.5581	0.0197	3.7521
GO_BP	negative regulation of myeloid cell differentiation	6	0.3690	0.0185	3.7238		GO_BP	I-kappaB kinase/NF-kappaB signaling	9	0.8372	0.0201	2.6265
GO_BP	spliceosomal complex assembly	7	0.4305	0.0193	3.1748		GO_BP	protein homotetramerization	9	0.8372	0.0201	2.6265
GO_BP	protein targeting to lysosome	5	0.3075	0.0197	4.5354		GO_BP	positive regulation of proteolysis	5	0.4651	0.0205	4.6079
GO_BP	PMA-inducible membrane protein	3	0.1845	0.0203	11.7921		GO_BP	mRNA 3'-end processing	8	0.7442	0.0225	2.8016

	ectodomain proteolysis											
GO_BP	Golgi organization	13	0.7995	0.0211	2.0716		GO_BP	positive regulation of viral transcription	6	0.5581	0.0227	3.6227
GO_BP	phosphatidylinositol biosynthetic process	11	0.6765	0.0228	2.2364		GO_BP	negative regulation of type I interferon production	6	0.5581	0.0260	3.5020
GO_BP	histone H3-K4 methylation	6	0.3690	0.0229	3.5376		GO_BP	mitotic nuclear division	23	2.1395	0.0267	1.6239
GO_BP	mRNA 3'-end processing	10	0.6150	0.0232	2.3584		GO_BP	type I interferon signaling pathway	9	0.8372	0.0285	2.4623
GO_BP	production of miRNAs involved in gene silencing by miRNA	4	0.2460	0.0246	5.8961		GO_BP	cellular response to oxidative stress	9	0.8372	0.0285	2.4623
GO_BP	protein targeting to vacuole	4	0.2460	0.0246	5.8961		GO_BP	protection from natural killer cell mediated cytotoxicity	3	0.2791	0.0290	10.5059
GO_BP	regulation of protein kinase B signaling	4	0.2460	0.0246	5.8961		GO_BP	isocitrate metabolic process	3	0.2791	0.0290	10.5059
GO_BP	negative regulation of transcription from RNA polymerase II promoter	77	4.7355	0.0258	1.2611		GO_BP	regulation of growth	8	0.7442	0.0301	2.6430
GO_BP	regulation of cell motility	7	0.4305	0.0273	2.9480		GO_BP	positive regulation of translation	8	0.7442	0.0301	2.6430
GO_BP	cellular component disassembly involved in execution phase of apoptosis	6	0.3690	0.0281	3.3692		GO_BP	negative regulation of protein catabolic process	6	0.5581	0.0334	3.2831
GO_BP	ER-associated ubiquitin-dependent protein catabolic process	11	0.6765	0.0283	2.1619		GO_BP	positive regulation of neuron apoptotic process	7	0.6512	0.0341	2.8504
GO_BP	lysosome organization	8	0.4920	0.0292	2.6205		GO_BP	positive regulation of T cell mediated cytotoxicity	4	0.3721	0.0344	5.3877
GO_BP	spleen development	8	0.4920	0.0292	2.6205		GO_BP	RNA export from nucleus	8	0.7442	0.0359	2.5469
GO_BP	cellular response to UV	9	0.5535	0.0297	2.4120		GO_BP	stimulatory C-type lectin receptor signaling pathway	12	1.1163	0.0370	2.0011
GO_BP	mitotic cytokinesis	7	0.4305	0.0321	2.8464		GO_BP	intrinsic apoptotic signaling pathway in response to endoplasmic reticulum stress	6	0.5581	0.0376	3.1836
GO_BP	positive regulation of microtubule polymerization	5	0.3075	0.0329	3.9307		GO_BP	formation of translation preinitiation complex	5	0.4651	0.0392	3.8065
GO_BP	negative regulation of cell-matrix adhesion	5	0.3075	0.0329	3.9307		GO_BP	establishment of protein localization to telomere	3	0.2791	0.0418	8.7550
GO_BP	cell adhesion mediated by integrin	5	0.3075	0.0329	3.9307		GO_BP	DNA double-strand break processing involved in repair via single-strand annealing	3	0.2791	0.0418	8.7550
GO_BP	phospholipid translocation	6	0.3690	0.0338	3.2160		GO_BP	positive regulation of exit from mitosis	3	0.2791	0.0418	8.7550
GO_BP	cell migration	23	1.4145	0.0339	1.5769		GO_BP	histone H4-R3 methylation	3	0.2791	0.0418	8.7550
GO_BP	protein complex assembly	17	1.0455	0.0355	1.7282		GO_BP	mitotic sister chromatid cohesion	4	0.3721	0.0420	5.0028
GO_BP	face morphogenesis	7	0.4305	0.0373	2.7515		GO_BP	maturation of SSU-rRNA	4	0.3721	0.0420	5.0028
GO_BP	proteasome-mediated ubiquitin-dependent protein catabolic process	26	1.5990	0.0374	1.5103		GO_BP	positive regulation of extrinsic apoptotic signaling pathway in absence of ligand	4	0.3721	0.0420	5.0028
GO_BP	regulation of alternative mRNA splicing, via	8	0.4920	0.0382	2.4826		GO_BP	negative regulation of protein binding	8	0.7442	0.0425	2.4575

	spliceosome										
GO_BP	mRNA cleavage involved in gene silencing by siRNA	3	0.1845	0.0384	8.8441		GO_BP	cellular response to interleukin-4	5	0.4651	0.0450	3.6479
GO_BP	positive regulation of transcription from RNA polymerase II promoter in response to hypoxia	3	0.1845	0.0384	8.8441		GO_BP	Ras protein signal transduction	9	0.8372	0.0452	2.2513
GO_BP	negative regulation of sodium ion transport	3	0.1845	0.0384	8.8441		GO_BP	negative regulation of canonical Wnt signaling pathway	16	1.4884	0.0460	1.7188
GO_BP	T follicular helper cell differentiation	3	0.1845	0.0384	8.8441		GO_BP	organ regeneration	7	0.6512	0.0496	2.6079
GO_BP	microtubule-based movement	13	0.7995	0.0396	1.8926		GO_CC	nucleoplasm	289	26.8837	0.0000	1.8657
GO_BP	RNA export from nucleus	10	0.6150	0.0403	2.1440		GO_CC	mitochondrion	160	14.8837	0.0000	2.1605
GO_BP	regulation of JNK cascade	5	0.3075	0.0410	3.6850		GO_CC	cytosol	299	27.8140	0.0000	1.6210
GO_BP	mRNA export from nucleus	15	0.9225	0.0423	1.7688		GO_CC	cytoplasm	417	38.7907	0.0000	1.4352
GO_BP	regulation of endocytosis	7	0.4305	0.0430	2.6627		GO_CC	mitochondrial inner membrane	72	6.6977	0.0000	2.9343
GO_BP	cellular response to nerve growth factor stimulus	7	0.4305	0.0430	2.6627		GO_CC	extracellular exosome	249	23.1628	0.0000	1.5920
GO_BP	Wnt signaling pathway, calcium modulating pathway	8	0.4920	0.0433	2.4189		GO_CC	ribosome	38	3.5349	0.0000	4.1142
GO_BP	cerebral cortex development	10	0.6150	0.0446	2.1057		GO_CC	nucleus	387	36.0000	0.0000	1.2845
GO_BP	Wnt signaling pathway	24	1.4760	0.0449	1.5134		GO_CC	membrane	180	16.7442	0.0000	1.4705
GO_BP	vacuolar proton-transporting V-type ATPase complex assembly	4	0.2460	0.0464	4.7169		GO_CC	small ribosomal subunit	11	1.0233	0.0000	7.3221
GO_BP	protein K11-linked deubiquitination	4	0.2460	0.0464	4.7169		GO_CC	cytosolic small ribosomal subunit	14	1.3023	0.0000	5.2419
GO_BP	peripheral nervous system neuron development	4	0.2460	0.0464	4.7169		GO_CC	mitochondrial large ribosomal subunit	14	1.3023	0.0000	5.2419
GO_BP	endoplasmic reticulum mannose trimming	4	0.2460	0.0464	4.7169		GO_CC	mitochondrial matrix	41	3.8140	0.0000	2.2534
GO_BP	negative regulation of osteoclast differentiation	6	0.3690	0.0475	2.9480		GO_CC	catalytic step 2 spliceosome	19	1.7674	0.0000	3.7117
GO_BP	regulation of cell shape	19	1.1685	0.0491	1.6004		GO_CC	cell-cell adherens junction	38	3.5349	0.0000	2.1144
GO_CC	nucleoplasm	366	22.5092	0.0000	1.5877		GO_CC	focal adhesion	43	4.0000	0.0000	1.9765
GO_CC	nucleus	608	37.3924	0.0000	1.3560		GO_CC	nucleolus	77	7.1628	0.0000	1.6148
GO_CC	cytoplasm	546	33.5793	0.0000	1.2627		GO_CC	mitochondrial respiratory chain complex I	12	1.1163	0.0001	4.4014
GO_CC	nuclear speck	44	2.7060	0.0000	2.6437		GO_CC	preribosome, large subunit precursor	9	0.8372	0.0001	5.5776
GO_CC	centrosome	72	4.4280	0.0000	2.0412		GO_CC	myelin sheath	21	1.9535	0.0003	2.4830

GO_CC	intracellular membrane-bounded organelle	82	5.0431	0.0000	1.7747		GO_CC	mitochondrial nucleoid	10	0.9302	0.0007	3.9939
GO_CC	nucleolus	108	6.6421	0.0000	1.5219		GO_CC	lysosome	25	2.3256	0.0018	1.9881
GO_CC	Golgi apparatus	107	6.5806	0.0000	1.4974		GO_CC	vesicle	17	1.5814	0.0020	2.3870
GO_CC	cytosol	334	20.5412	0.0000	1.2168		GO_CC	mitochondrial intermembrane space	12	1.1163	0.0025	2.9144
GO_CC	ciliary basal body	22	1.3530	0.0000	2.7111		GO_CC	U12-type spliceosomal complex	7	0.6512	0.0026	4.8387
GO_CC	postsynaptic density	33	2.0295	0.0000	2.1660		GO_CC	intercellular bridge	9	0.8372	0.0026	3.6762
GO_CC	cell-cell adherens junction	48	2.9520	0.0001	1.7947		GO_CC	spindle	16	1.4884	0.0029	2.3765
GO_CC	ruffle	20	1.2300	0.0001	2.6837		GO_CC	intracellular ribonucleoprotein complex	17	1.5814	0.0037	2.2465
GO_CC	membrane	228	14.0221	0.0002	1.2516		GO_CC	intracellular membrane-bounded organelle	47	4.3721	0.0049	1.5138
GO_CC	trans-Golgi network	25	1.5375	0.0003	2.2200		GO_CC	microtubule organizing center	18	1.6744	0.0050	2.1144
GO_CC	intraciliary transport particle B	8	0.4920	0.0005	5.0850		GO_CC	cell body	10	0.9302	0.0077	2.8528
GO_CC	primary cilium	17	1.0455	0.0006	2.6321		GO_CC	extracellular matrix	28	2.6047	0.0080	1.7001
GO_CC	lamellipodium	27	1.6605	0.0007	2.0380		GO_CC	nuclear speck	21	1.9535	0.0085	1.8777
GO_CC	COPII vesicle coat	6	0.3690	0.0007	7.2461		GO_CC	Slx1-Slx4 complex	3	0.2791	0.0089	17.9724
GO_CC	Golgi membrane	72	4.4280	0.0009	1.4713		GO_CC	MCM complex	4	0.3721	0.0112	7.9877
GO_CC	nuclear matrix	19	1.1685	0.0009	2.3656		GO_CC	peroxisome	13	1.2093	0.0127	2.2465
GO_CC	intracellular	142	8.7331	0.0012	1.2875		GO_CC	spliceosomal complex	12	1.1163	0.0151	2.2943
GO_CC	nuclear membrane	34	2.0910	0.0012	1.7931		GO_CC	MHC class I protein complex	4	0.3721	0.0202	6.5354
GO_CC	perinuclear region of cytoplasm	73	4.4895	0.0022	1.4197		GO_CC	MLL1 complex	6	0.5581	0.0205	3.7184
GO_CC	Cajal body	12	0.7380	0.0026	2.8416		GO_CC	cytoplasmic microtubule	8	0.7442	0.0220	2.8192
GO_CC	ciliary tip	11	0.6765	0.0027	3.0192		GO_CC	lipid particle	9	0.8372	0.0293	2.4508
GO_CC	microtubule organizing center	24	1.4760	0.0037	1.8944		GO_CC	exon-exon junction complex	5	0.4651	0.0312	4.0846
GO_CC	chromosome, telomeric region	11	0.6765	0.0038	2.8879		GO_CC	small nucleolar ribonucleoprotein complex	4	0.3721	0.0322	5.5300
GO_CC	midbody	21	1.2915	0.0046	1.9660		GO_CC	PRC1 complex	4	0.3721	0.0322	5.5300
GO_CC	transcriptional repressor complex	12	0.7380	0.0048	2.6350		GO_CC	cytoplasmic exosome (RNase complex)	4	0.3721	0.0322	5.5300
GO_CC	chromosome	18	1.1070	0.0055	2.0703		GO_CC	lysosomal membrane	24	2.2326	0.0323	1.5742
GO_CC	cell cortex	20	1.2300	0.0059	1.9637		GO_CC	centrosome	34	3.1628	0.0341	1.4344
GO_CC	PML body	17	1.0455	0.0065	2.0950		GO_CC	cytosolic large ribosomal subunit	9	0.8372	0.0343	2.3787
GO_CC	cis-Golgi network	10	0.6150	0.0065	2.8754		GO_CC	U4/U6 x U5 tri-snRNP complex	5	0.4651	0.0361	3.9070
GO_CC	ciliary rootlet	5	0.3075	0.0065	6.0384		GO_CC	anaphase-promoting complex	5	0.4651	0.0361	3.9070
GO_CC	spindle pole	18	1.1070	0.0081	1.9943		GO_CC	COPI vesicle coat	4	0.3721	0.0394	5.1350
GO_CC	endoplasmic reticulum-Golgi intermediate compartment	13	0.7995	0.0093	2.3088		GO_CC	microtubule cytoskeleton	14	1.3023	0.0416	1.8366

GO_CC	micro-ribonucleoprotein complex	4	0.2460	0.0093	8.0512		GO_CC	integral component of peroxisomal membrane	4	0.3721	0.0472	4.7926
GO_CC	Golgi cisterna	5	0.3075	0.0096	5.4895		GO_CC	eukaryotic 43S preinitiation complex	4	0.3721	0.0472	4.7926
GO_CC	RISC complex	5	0.3075	0.0096	5.4895		GO_CC	eukaryotic 48S preinitiation complex	4	0.3721	0.0472	4.7926
GO_CC	focal adhesion	47	2.8905	0.0098	1.4517		GO_CC	precatalytic spliceosome	5	0.4651	0.0473	3.5945
GO_CC	cilium	22	1.3530	0.0129	1.7595		GO_CC	proteasome complex	8	0.7442	0.0479	2.3963
GO_CC	kinetochore	14	0.8610	0.0152	2.0874		KEGG	Ribosome	36	3.3488	0.0000	4.0919
GO_CC	RISC-loading complex	4	0.2460	0.0154	6.9011		KEGG	Parkinson's disease	30	2.7907	0.0000	3.2659
GO_CC	endosome membrane	25	1.5375	0.0185	1.6320		KEGG	Biosynthesis of amino acids	19	1.7674	0.0000	4.0793
GO_CC	microtubule associated complex	8	0.4920	0.0194	2.8416		KEGG	Huntington's disease	33	3.0698	0.0000	2.6569
GO_CC	actin cytoskeleton	28	1.7220	0.0227	1.5512		KEGG	Alzheimer's disease	29	2.6977	0.0000	2.6684
GO_CC	phosphatidylinositol 3-kinase complex	5	0.3075	0.0238	4.3132		KEGG	Oxidative phosphorylation	25	2.3256	0.0000	2.9057
GO_CC	ER to Golgi transport vesicle membrane	10	0.6150	0.0256	2.3225		KEGG	Non-alcoholic fatty liver disease (NAFLD)	25	2.3256	0.0000	2.5593
GO_CC	cytoplasmic mRNA processing body	13	0.7995	0.0261	2.0128		KEGG	Spliceosome	21	1.9535	0.0003	2.4408
GO_CC	nuclear pore	12	0.7380	0.0338	2.0128		KEGG	Metabolic pathways	105	9.7674	0.0010	1.3315
GO_CC	neuromuscular junction	10	0.6150	0.0354	2.1958		KEGG	Carbon metabolism	17	1.5814	0.0023	2.3256
GO_CC	transcriptionally active chromatin	6	0.3690	0.0369	3.1505		KEGG	Biosynthesis of antibiotics	26	2.4186	0.0023	1.8958
GO_CC	spindle	17	1.0455	0.0413	1.6968		KEGG	Epstein-Barr virus infection	17	1.5814	0.0050	2.1540
GO_CC	ciliary transition zone	6	0.3690	0.0436	3.0192		KEGG	2-Oxocarboxylic acid metabolism	5	0.4651	0.0208	4.5466
GO_CC	oligosaccharyltransferase complex	4	0.2460	0.0436	4.8307		KEGG	Proteasome	8	0.7442	0.0213	2.8106
GO_CC	axon	27	1.6605	0.0454	1.4688		KEGG	Sulfur relay system	4	0.3721	0.0228	6.1834
GO_CC	endoplasmic reticulum membrane	86	5.2891	0.0499	1.2049		KEGG	Viral carcinogenesis	22	2.0465	0.0232	1.6590
KEGG	Thyroid hormone signaling pathway	23	1.4145	0.0000	2.8544		KEGG	RNA degradation	11	1.0233	0.0254	2.2083
KEGG	Ubiquitin mediated proteolysis	24	1.4760	0.0001	2.5002		KEGG	Herpes simplex infection	20	1.8605	0.0264	1.6894
KEGG	Proteoglycans in cancer	30	1.8450	0.0001	2.1408		KEGG	Pyrimidine metabolism	13	1.2093	0.0286	1.9897
KEGG	Prostate cancer	17	1.0455	0.0003	2.7570		KEGG	Glycine, serine and threonine metabolism	7	0.6512	0.0372	2.7746
KEGG	Pathways in cancer	46	2.8290	0.0006	1.6705		KEGG	Galactose metabolism	6	0.5581	0.0409	3.0917
KEGG	Small cell lung cancer	16	0.9840	0.0007	2.6865		KEGG	Lysosome	14	1.3023	0.0475	1.7886
KEGG	Endometrial cancer	12	0.7380	0.0008	3.2935							
KEGG	Spliceosome	21	1.2915	0.0009	2.2534							
KEGG	FoxO signaling pathway	20	1.2300	0.0023	2.1301							
KEGG	Protein processing in endoplasmic reticulum	23	1.4145	0.0033	1.9423							
KEGG	Chronic myeloid leukemia	13	0.7995	0.0039	2.5768							

KEGG	Non-small cell lung cancer	11	0.6765	0.0049	2.8034							
KEGG	Cell cycle	18	1.1070	0.0055	2.0717							
KEGG	Signaling pathways regulating pluripotency of stem cells	19	1.1685	0.0085	1.9369							
KEGG	Estrogen signaling pathway	15	0.9225	0.0086	2.1624							
KEGG	Focal adhesion	25	1.5375	0.0090	1.7320							
KEGG	Colorectal cancer	11	0.6765	0.0101	2.5321							
KEGG	mRNA surveillance pathway	14	0.8610	0.0102	2.1957							
KEGG	Glioma	11	0.6765	0.0140	2.4152							
KEGG	PI3K-Akt signaling pathway	36	2.2140	0.0154	1.4892							
KEGG	Notch signaling pathway	9	0.5535	0.0170	2.6760							
KEGG	RNA degradation	12	0.7380	0.0174	2.2242							
KEGG	Sphingolipid signaling pathway	16	0.9840	0.0196	1.9029							
KEGG	Epstein-Barr virus infection	16	0.9840	0.0224	1.8717							
KEGG	Melanoma	11	0.6765	0.0249	2.2111							
KEGG	Lysine degradation	9	0.5535	0.0266	2.4701							
KEGG	Endocytosis	26	1.5990	0.0296	1.5397							
KEGG	HIF-1 signaling pathway	13	0.7995	0.0346	1.9326							
KEGG	Pancreatic cancer	10	0.6150	0.0358	2.1957							
KEGG	Platelet activation	16	0.9840	0.0371	1.7565							
KEGG	Renal cell carcinoma	10	0.6150	0.0390	2.1624							
KEGG	Progesterone-mediated oocyte maturation	12	0.7380	0.0391	1.9685							
KEGG	ErbB signaling pathway	12	0.7380	0.0391	1.9685							
KEGG	Phosphatidylinositol signaling system	13	0.7995	0.0397	1.8932							
KEGG	Neurotrophin signaling pathway	15	0.9225	0.0397	1.7840							
KEGG	p53 signaling pathway	10	0.6150	0.0423	2.1301							
KEGG	Hepatitis B	17	1.0455	0.0455	1.6732							
KEGG	mTOR signaling pathway	9	0.5535	0.0472	2.2146							

Table S2. Geneset enrichment analysis using genes altered by LDE225 *vs.* DMSO in ONS76 with SMO^{W535L}.

Genesets enriched by downregulated genes from LDE225 *vs.* DMSO							Genesets enriched by upregulated genes from LDE225 *vs.* DMSO					
Category	Term	Count	%	*P* Value	Fold Enrichment		Category	Term	Count	%	*P* Value	Fold Enrichment

GO_BP	positive regulation of axonogenesis	6	1.4085	0.0001	12.4940		GO_BP	protein transport	37	5.6402	0.0000	2.6888
GO_BP	autophagy	12	2.8169	0.0002	3.9455		GO_BP	negative regulation of translation	9	1.3720	0.0008	4.4541
GO_BP	cell-cell adhesion	17	3.9906	0.0005	2.7432		GO_BP	vesicle-mediated transport	15	2.2866	0.0009	2.8327
GO_BP	retinoic acid receptor signaling pathway	5	1.1737	0.0006	12.1470		GO_BP	NLS-bearing protein import into nucleus	6	0.9146	0.0010	7.4881
GO_BP	viral transcription	10	2.3474	0.0011	3.9044		GO_BP	cell-cell adhesion	21	3.2012	0.0013	2.2243
GO_BP	vascular endothelial growth factor receptor signaling pathway	8	1.8779	0.0013	4.8588		GO_BP	tRNA methylation	6	0.9146	0.0018	6.6241
GO_BP	branching involved in mammary gland duct morphogenesis	4	0.9390	0.0022	14.5764		GO_BP	protein polyubiquitination	16	2.4390	0.0020	2.4960
GO_BP	mitochondrial respiratory chain complex I assembly	7	1.6432	0.0031	4.8588		GO_BP	transcription, DNA-templated	92	14.0244	0.0022	1.3508
GO_BP	translational initiation	10	2.3474	0.0042	3.1919		GO_BP	cilium morphogenesis	13	1.9817	0.0028	2.7438
GO_BP	mitochondrial electron transport, NADH to ubiquinone	6	1.4085	0.0050	5.3546		GO_BP	negative regulation of endothelial cell proliferation	6	0.9146	0.0030	5.9388
GO_BP	neurotrophin TRK receptor signaling pathway	4	0.9390	0.0053	10.9323		GO_BP	Golgi to endosome transport	5	0.7622	0.0037	7.5538
GO_BP	SRP-dependent cotranslational protein targeting to membrane	8	1.8779	0.0057	3.7216		GO_BP	cilium assembly	12	1.8293	0.0040	2.7778
GO_BP	protein destabilization	5	1.1737	0.0080	6.2470		GO_BP	negative regulation of canonical Wnt signaling pathway	14	2.1341	0.0046	2.4654
GO_BP	cellular triglyceride homeostasis	3	0.7042	0.0101	18.7411		GO_BP	regulation of filopodium assembly	4	0.6098	0.0056	10.4379
GO_BP	rRNA processing	12	2.8169	0.0102	2.4521		GO_BP	small GTPase mediated signal transduction	18	2.7439	0.0056	2.1003
GO_BP	ER-associated ubiquitin-dependent protein catabolic process	6	1.4085	0.0118	4.3729		GO_BP	cell division	23	3.5061	0.0056	1.8863
GO_BP	positive regulation of protein catabolic process	6	1.4085	0.0118	4.3729		GO_BP	mitotic nuclear division	18	2.7439	0.0062	2.0834
GO_BP	response to retinoic acid	5	1.1737	0.0139	5.3328		GO_BP	regulation of transcription, DNA-templated	71	10.8232	0.0072	1.3551
GO_BP	response to hypoxia	10	2.3474	0.0173	2.5424		GO_BP	endocytosis	12	1.8293	0.0093	2.4781
GO_BP	adipose tissue development	4	0.9390	0.0187	6.9967		GO_BP	snRNA transcription from RNA polymerase II promoter	8	1.2195	0.0107	3.2805
GO_BP	endoplasmic reticulum unfolded protein response	5	1.1737	0.0190	4.8588		GO_BP	positive regulation of xenophagy	3	0.4573	0.0113	17.2226
GO_BP	nuclear-transcribed mRNA catabolic process, nonsense-mediated decay	8	1.8779	0.0193	2.9398		GO_BP	tRNA modification	5	0.7622	0.0135	5.3156
GO_BP	glucose 6-phosphate metabolic process	3	0.7042	0.0207	13.1188		GO_BP	Golgi to plasma membrane protein transport	5	0.7622	0.0135	5.3156
GO_BP	UDP-N-acetylglucosamine biosynthetic process	3	0.7042	0.0249	11.9261		GO_BP	negative regulation of protein binding	7	1.0671	0.0139	3.5251
GO_BP	regulation of mitochondrial membrane potential	4	0.9390	0.0253	6.2470		GO_BP	double-strand break repair via homologous recombination	8	1.2195	0.0143	3.1032

GO_BP	positive regulation of viral transcription	4	0.9390	0.0277	6.0316		GO_BP	transforming growth factor beta receptor signaling pathway	9	1.3720	0.0147	2.8080
GO_BP	cellular response to glucose starvation	4	0.9390	0.0277	6.0316		GO_BP	vesicle organization	5	0.7622	0.0153	5.1258
GO_BP	response to endoplasmic reticulum stress	6	1.4085	0.0283	3.4983		GO_BP	mesoderm formation	5	0.7622	0.0153	5.1258
GO_BP	lateral ventricle development	3	0.7042	0.0294	10.9323		GO_BP	regulation of translational termination	3	0.4573	0.0165	14.3521
GO_BP	translation	12	2.8169	0.0312	2.0741		GO_BP	SNARE complex assembly	3	0.4573	0.0165	14.3521
GO_BP	positive regulation of epithelial cell migration	4	0.9390	0.0388	5.3005		GO_BP	heart looping	7	1.0671	0.0190	3.2939
GO_BP	retrograde protein transport, ER to cytosol	3	0.7042	0.0394	9.3705		GO_BP	negative regulation of BMP signaling pathway	6	0.9146	0.0194	3.8272
GO_BP	endocytosis	8	1.8779	0.0402	2.5168		GO_BP	negative regulation of protein import into nucleus	3	0.4573	0.0226	12.3018
GO_BP	activation of cysteine-type endopeptidase activity involved in apoptotic process	6	1.4085	0.0412	3.1611		GO_BP	mRNA transport	6	0.9146	0.0231	3.6644
GO_BP	protein autophosphorylation	9	2.1127	0.0438	2.2882		GO_BP	protein K48-linked ubiquitination	6	0.9146	0.0231	3.6644
GO_BP	ERAD pathway	3	0.7042	0.0448	8.7458		GO_BP	mRNA catabolic process	4	0.6098	0.0231	6.3787
GO_BP	G-protein coupled acetylcholine receptor signaling pathway	3	0.7042	0.0448	8.7458		GO_BP	regulation of Wnt signaling pathway	4	0.6098	0.0268	6.0430
GO_BP	positive regulation of protein serine/threonine kinase activity	4	0.9390	0.0450	4.9976		GO_BP	regulation of signal transduction by p53 class mediator	10	1.5244	0.0291	2.3149
GO_BP	regulation of glucose import in response to insulin stimulus	2	0.4695	0.0451	43.7292		GO_BP	protein desumoylation	3	0.4573	0.0294	10.7641
GO_BP	IRE1-mediated unfolded protein response	5	1.1737	0.0455	3.7059		GO_BP	myoblast migration	3	0.4573	0.0294	10.7641
GO_CC	nucleoplasm	98	23.0047	0.0000	1.6078		GO_BP	regulation of transforming growth factor beta receptor signaling pathway	4	0.6098	0.0307	5.7409
GO_CC	membrane	80	18.7793	0.0000	1.6609		GO_BP	retrograde transport, endosome to Golgi	7	1.0671	0.0326	2.9120
GO_CC	cytosol	108	25.3521	0.0000	1.4880		GO_BP	regulation of protein localization	6	0.9146	0.0340	3.3120
GO_CC	cytoplasm	153	35.9155	0.0000	1.3382		GO_BP	positive regulation of protein autophosphorylation	4	0.6098	0.0349	5.4675
GO_CC	cell-cell adherens junction	19	4.4601	0.0003	2.6867		GO_BP	melanosome transport	4	0.6098	0.0349	5.4675
GO_CC	ribosome	13	3.0516	0.0003	3.5769		GO_BP	cell cycle checkpoint	3	0.4573	0.0370	9.5681
GO_CC	extracellular exosome	84	19.7183	0.0021	1.3649		GO_BP	embryo development	5	0.7622	0.0420	3.7769
GO_CC	focal adhesion	19	4.4601	0.0025	2.2195		GO_BP	ureteric bud development	5	0.7622	0.0420	3.7769
GO_CC	endoplasmic reticulum membrane	32	7.5117	0.0046	1.6956		GO_BP	endosome to lysosome transport	5	0.7622	0.0456	3.6800
GO_CC	perinuclear region of cytoplasm	25	5.8685	0.0052	1.8387		GO_BP	endocytic recycling	4	0.6098	0.0493	4.7840
GO_CC	endoplasmic reticulum	30	7.0423	0.0085	1.6549		GO_CC	cytosol	178	27.1341	0.0000	1.5885
GO_CC	mitochondrion	43	10.0939	0.0103	1.4756		GO_CC	nucleoplasm	156	23.7805	0.0000	1.6577

GO_CC	cytoskeleton	16	3.7559	0.0169	1.9698		GO_CC	cytoplasm	231	35.2134	0.0000	1.3087
GO_CC	mitochondrial inner membrane	18	4.2254	0.0173	1.8643		GO_CC	membrane	112	17.0732	0.0000	1.5061
GO_CC	nucleus	139	32.6291	0.0174	1.1724		GO_CC	intracellular	71	10.8232	0.0001	1.5769
GO_CC	mitochondrial respiratory chain complex I	5	1.1737	0.0219	4.6606		GO_CC	nucleus	224	34.1463	0.0002	1.2238
GO_CC	midbody	8	1.8779	0.0232	2.8325		GO_CC	early endosome	19	2.8963	0.0008	2.4546
GO_MF	protein binding	251	58.9202	0.0000	1.2626		GO_CC	centrosome	28	4.2683	0.0013	1.9445
GO_MF	cadherin binding involved in cell-cell adhesion	18	4.2254	0.0003	2.7429		GO_CC	cell-cell adherens junction	23	3.5061	0.0015	2.1066
GO_MF	protein complex binding	14	3.2864	0.0008	3.0033		GO_CC	endosome membrane	16	2.4390	0.0016	2.5587
GO_MF	NADH dehydrogenase (ubiquinone) activity	6	1.4085	0.0044	5.5239		GO_CC	endosome	18	2.7439	0.0017	2.3668
GO_MF	translation initiation factor binding	4	0.9390	0.0112	8.4174		GO_CC	chromosome, centromeric region	8	1.2195	0.0029	4.1522
GO_MF	ferrous iron binding	4	0.9390	0.0128	8.0347		GO_CC	retromer complex	5	0.7622	0.0058	6.7237
GO_MF	polyubiquitin binding	4	0.9390	0.0145	7.6854		GO_CC	transcription factor complex	15	2.2866	0.0060	2.2993
GO_MF	ubiquitin protein ligase activity involved in ERAD pathway	3	0.7042	0.0165	14.7304		GO_CC	nucleolus	43	6.5549	0.0100	1.4844
GO_MF	enzyme binding	15	3.5211	0.0191	1.9906		GO_CC	Golgi apparatus	43	6.5549	0.0112	1.4741
GO_MF	intermediate filament binding	3	0.7042	0.0203	13.2573		GO_CC	clathrin-coated vesicle	7	1.0671	0.0132	3.5705
GO_MF	kinase activity	12	2.8169	0.0212	2.2004		GO_CC	cilium	12	1.8293	0.0135	2.3511
GO_MF	poly(A) RNA binding	37	8.6854	0.0231	1.4482		GO_CC	nuclear chromatin	14	2.1341	0.0141	2.1460
GO_MF	protein serine/threonine/tyrosine kinase activity	4	0.9390	0.0270	6.0953		GO_CC	primary cilium	8	1.2195	0.0161	3.0343
GO_MF	DNA-(apurinic or apyrimidinic site) lyase activity	3	0.7042	0.0289	11.0478		GO_CC	ciliary basal body	9	1.3720	0.0178	2.7169
GO_MF	structural constituent of ribosome	11	2.5822	0.0295	2.1896		GO_CC	peroxisome	9	1.3720	0.0244	2.5602
GO_MF	methylated histone binding	5	1.1737	0.0314	4.1690		GO_CC	intraciliary transport particle B	4	0.6098	0.0248	6.2283
GO_MF	identical protein binding	26	6.1033	0.0333	1.5340		GO_CC	SMAD protein complex	3	0.4573	0.0278	11.0942
GO_MF	phosphoprotein binding	4	0.9390	0.0407	5.1990		GO_CC	protein phosphatase type 2A complex	4	0.6098	0.0284	5.9169
GO_MF	GTP-Rho binding	3	0.7042	0.0495	8.2858		GO_CC	cell-cell junction	12	1.8293	0.0318	2.0640
GO_MF	phosphatidic acid binding	3	0.7042	0.0495	8.2858		GO_CC	SNARE complex	6	0.9146	0.0327	3.3492
GO_MF	transforming growth factor beta binding	3	0.7042	0.0495	8.2858		GO_CC	recycling endosome	9	1.3720	0.0327	2.4205
KEGG	Non-alcoholic fatty liver disease (NAFLD)	11	2.5822	0.0046	2.8800		GO_CC	VCP-NPL4-UFD1 AAA ATPase complex	3	0.4573	0.0350	9.8615
KEGG	Ribosome	10	2.3474	0.0071	2.9069		GO_CC	phagocytic vesicle	5	0.7622	0.0416	3.7929
KEGG	Biosynthesis of antibiotics	12	2.8169	0.0176	2.2378		GO_CC	lysosomal membrane	16	2.4390	0.0457	1.7276
KEGG	Oxidative phosphorylation	9	2.1127	0.0186	2.6753		GO_CC	cytoskeleton	20	3.0488	0.0476	1.5948
KEGG	Central carbon metabolism in cancer	6	1.4085	0.0219	3.7064		KEGG	SNARE interactions in vesicular transport	6	0.9146	0.0054	5.1657
KEGG	Protein processing in endoplasmic reticulum	10	2.3474	0.0263	2.3393		KEGG	RNA transport	14	2.1341	0.0056	2.3826

KEGG	Parkinson's disease	9	2.1127	0.0264	2.5057		KEGG	Endocytosis	16	2.4390	0.0169	1.9434
KEGG	Adipocytokine signaling pathway	6	1.4085	0.0309	3.3887		KEGG	mRNA surveillance pathway	8	1.2195	0.0348	2.5734
KEGG	Amino sugar and nucleotide sugar metabolism	5	1.1737	0.0319	4.1182		KEGG	Terpenoid backbone biosynthesis	4	0.6098	0.0371	5.3222
KEGG	Pyrimidine metabolism	7	1.6432	0.0413	2.7400		KEGG	Ubiquitin mediated proteolysis	10	1.5244	0.0435	2.1367

Table S3. Geneset enrichment analysis using genes significantly upregulated by LDE225 *vs.* DMSO in ONS76 with SMO^{WT}.

Category	Term	Count	%	*P* Value	Fold Enrichment
GOTERM_BP_DIRECT	GO:0006355~regulation of transcription, DNA-templated	77	17.11111111	7.36544E-11	2.187523686
GOTERM_BP_DIRECT	GO:0006351~transcription, DNA-templated	83	18.44444444	7.49403E-08	1.814016387
GOTERM_BP_DIRECT	GO:0042384~cilium assembly	13	2.888888889	3.35395E-05	4.479520644
GOTERM_BP_DIRECT	GO:2000114~regulation of establishment of cell polarity	5	1.111111111	0.000546905	12.56698099
GOTERM_BP_DIRECT	GO:0071108~protein K48-linked deubiquitination	5	1.111111111	0.002146184	8.901611535
GOTERM_BP_DIRECT	GO:2000145~regulation of cell motility	5	1.111111111	0.003842757	7.629952744
GOTERM_BP_DIRECT	GO:0006338~chromatin remodeling	8	1.777777778	0.003957901	3.974673058
GOTERM_BP_DIRECT	GO:0008380~RNA splicing	11	2.444444444	0.005682507	2.831355958
GOTERM_BP_DIRECT	GO:0007062~sister chromatid cohesion	8	1.777777778	0.010444004	3.318659058
GOTERM_BP_DIRECT	GO:0051057~positive regulation of small GTPase mediated signal transduction	3	0.666666667	0.010561987	18.31188659
GOTERM_BP_DIRECT	GO:0031123~RNA 3'-end processing	3	0.666666667	0.010561987	18.31188659
GOTERM_BP_DIRECT	GO:0035735~intraciliary transport involved in cilium morphogenesis	3	0.666666667	0.010561987	18.31188659
GOTERM_BP_DIRECT	GO:0006921~cellular component disassembly involved in execution phase of apoptosis	4	0.888888889	0.0122877	8.138616261
GOTERM_BP_DIRECT	GO:0045184~establishment of protein localization	5	1.111111111	0.013782579	5.340966921
GOTERM_BP_DIRECT	GO:0006357~regulation of transcription from RNA polymerase II promoter	19	4.222222222	0.015820674	1.840877488
GOTERM_BP_DIRECT	GO:0051301~cell division	16	3.555555556	0.017847049	1.953267903
GOTERM_BP_DIRECT	GO:0022604~regulation of cell morphogenesis	4	0.888888889	0.0198426	6.836437659
GOTERM_BP_DIRECT	GO:0015031~protein transport	17	3.777777778	0.023664078	1.838915193
GOTERM_BP_DIRECT	GO:0033169~histone H3-K9 demethylation	3	0.666666667	0.026008912	11.65301874
GOTERM_BP_DIRECT	GO:0016050~vesicle organization	4	0.888888889	0.026853618	6.103962196
GOTERM_BP_DIRECT	GO:0000281~mitotic cytokinesis	4	0.888888889	0.029447107	5.893480741
GOTERM_BP_DIRECT	GO:0006406~mRNA export from nucleus	7	1.555555556	0.029684369	2.990941476
GOTERM_BP_DIRECT	GO:0007010~cytoskeleton organization	9	2	0.035426332	2.388506946
GOTERM_BP_DIRECT	GO:0031054~pre-miRNA processing	3	0.666666667	0.035772669	9.860246624
GOTERM_BP_DIRECT	GO:0006622~protein targeting to lysosome	3	0.666666667	0.035772669	9.860246624
GOTERM_BP_DIRECT	GO:0051492~regulation of stress fiber assembly	3	0.666666667	0.035772669	9.860246624

GOTERM_BP_DIRECT	GO:0007032~endosome organization	4	0.888888889	0.037989116	5.340966921
GOTERM_BP_DIRECT	GO:0006405~RNA export from nucleus	5	1.111111111	0.039259768	3.884339579
GOTERM_BP_DIRECT	GO:0006468~protein phosphorylation	18	4	0.039399981	1.686621133
GOTERM_BP_DIRECT	GO:0060271~cilium morphogenesis	8	1.777777778	0.040464789	2.513396198
GOTERM_BP_DIRECT	GO:0030833~regulation of actin filament polymerization	3	0.666666667	0.041102389	9.155943293
GOTERM_BP_DIRECT	GO:0071044~histone mRNA catabolic process	3	0.666666667	0.041102389	9.155943293
GOTERM_BP_DIRECT	GO:0021591~ventricular system development	3	0.666666667	0.041102389	9.155943293
GOTERM_BP_DIRECT	GO:0007173~epidermal growth factor receptor signaling pathway	5	1.111111111	0.041530807	3.814976372
GOTERM_BP_DIRECT	GO:0043547~positive regulation of GTPase activity	21	4.666666667	0.043400453	1.588110518
GOTERM_BP_DIRECT	GO:0045892~negative regulation of transcription, DNA-templated	19	4.222222222	0.045558087	1.62690776
GOTERM_BP_DIRECT	GO:0070895~negative regulation of transposon integration	2	0.444444444	0.046145294	42.72773537
GOTERM_CC_DIRECT	GO:0005634~nucleus	180	40	5.12805E-10	1.484764543
GOTERM_CC_DIRECT	GO:0005813~centrosome	30	6.666666667	1.13492E-07	3.145539906
GOTERM_CC_DIRECT	GO:0005654~nucleoplasm	102	22.66666667	2.78517E-07	1.636494253
GOTERM_CC_DIRECT	GO:0005737~cytoplasm	156	34.66666667	2.11031E-05	1.334354653
GOTERM_CC_DIRECT	GO:0005815~microtubule organizing center	13	2.888888889	0.000170187	3.795206972
GOTERM_CC_DIRECT	GO:0005622~intracellular	51	11.33333333	0.000209322	1.71021021
GOTERM_CC_DIRECT	GO:0017053~transcriptional repressor complex	8	1.777777778	0.000211956	6.496969697
GOTERM_CC_DIRECT	GO:0043231~intracellular membrane-bounded organelle	27	6	0.000355352	2.161290323
GOTERM_CC_DIRECT	GO:0001726~ruffle	9	2	0.000907361	4.466666667
GOTERM_CC_DIRECT	GO:0016363~nuclear matrix	9	2	0.00147832	4.144329897
GOTERM_CC_DIRECT	GO:0036064~ciliary basal body	9	2	0.001579055	4.102040816
GOTERM_CC_DIRECT	GO:0031252~cell leading edge	6	1.333333333	0.001670396	6.871794872
GOTERM_CC_DIRECT	GO:0030027~lamellipodium	11	2.444444444	0.00323323	3.070833333
GOTERM_CC_DIRECT	GO:0016605~PML body	8	1.777777778	0.006386797	3.646258503
GOTERM_CC_DIRECT	GO:0005874~microtubule	15	3.333333333	0.01058307	2.154340836
GOTERM_CC_DIRECT	GO:0000922~spindle pole	8	1.777777778	0.011186817	3.278287462
GOTERM_CC_DIRECT	GO:0005794~Golgi apparatus	31	6.888888889	0.011268113	1.604480494
GOTERM_CC_DIRECT	GO:0015630~microtubule cytoskeleton	9	2	0.011898821	2.934306569
GOTERM_CC_DIRECT	GO:0005814~centriole	8	1.777777778	0.013452541	3.162241888
GOTERM_CC_DIRECT	GO:0031965~nuclear membrane	12	2.666666667	0.014117951	2.340611354
GOTERM_CC_DIRECT	GO:0000785~chromatin	7	1.555555556	0.014687161	3.513108614
GOTERM_CC_DIRECT	GO:0030496~midbody	8	1.777777778	0.025830512	2.77002584

GOTERM_CC_DIRECT	GO:0005925~focal adhesion	16	3.555555556	0.030007704	1.827791986
GOTERM_CC_DIRECT	GO:0072372~primary cilium	6	1.333333333	0.030338508	3.435897436
GOTERM_CC_DIRECT	GO:0005802~trans-Golgi network	8	1.777777778	0.0331219	2.62745098
GOTERM_CC_DIRECT	GO:0019898~extrinsic component of membrane	6	1.333333333	0.036522508	3.268292683
GOTERM_CC_DIRECT	GO:0016607~nuclear speck	10	2.222222222	0.036854181	2.222222222
GOTERM_CC_DIRECT	GO:0048471~perinuclear region of cytoplasm	22	4.888888889	0.039798788	1.582393988
GOTERM_CC_DIRECT	GO:0005829~cytosol	89	19.77777778	0.042240669	1.199195576
KEGG_PATHWAY	hsa05205:Proteoglycans in cancer	12	2.666666667	0.000743233	3.383114754
KEGG_PATHWAY	hsa04550:Signaling pathways regulating pluripotency of stem cells	9	2	0.003137074	3.624765808
KEGG_PATHWAY	hsa05214:Glioma	6	1.333333333	0.005519918	5.204791929
KEGG_PATHWAY	hsa05213:Endometrial cancer	5	1.111111111	0.01287443	5.42165826
KEGG_PATHWAY	hsa05200:Pathways in cancer	14	3.111111111	0.020196639	2.008634714
KEGG_PATHWAY	hsa05206:MicroRNAs in cancer	11	2.444444444	0.028646804	2.168663304
KEGG_PATHWAY	hsa04115:p53 signaling pathway	5	1.111111111	0.029848991	4.207854172
KEGG_PATHWAY	hsa04915:Estrogen signaling pathway	6	1.333333333	0.029907007	3.41728763
KEGG_PATHWAY	hsa04917:Prolactin signaling pathway	5	1.111111111	0.035886007	3.970791965
KEGG_PATHWAY	hsa05220:Chronic myeloid leukemia	5	1.111111111	0.037497812	3.915642077

Table S4. Metabolite examination of ONS76 cells expressing SMO^{WT} or SMO^{W535L} with treatment of LDE225 or DMSO.

name	adduct	description	m/z	rt(s)	W573L+DMSO-1	W573L+DMSO-2	W573L+DMSO-3	W573L+LDE225-1	W573L+LDE225-2	W573L+LDE225-3	WT+DMSO-1	WT+DMSO-2	WT+DMSO-3	WT+LDE225-1	WT+LDE225-2	WT+LDE225-3
M102T765	(M+H)+	1-Aminocyclopropanecarboxylic acid	102.05	765.37	25922.48	28159.85	26573.67	27134.47	25707.56	29051.78	29784.38	25408.79	29517.64	32508.34	33312.81	30469.84
M137T557	M+	1-Methylnicotinamide	137.07	556.86	33092.53	37164.38	34030.88	32428.46	28571.99	36725.32	17837.48	14272.80	9334.94	20816.03	22671.97	14625.31
M833T254	(M-H+2Na)+	1-Stearoyl-2-oleoyl-sn-glycerol 3-phosphocholine (SOPC)	832.58	254.25	7126.57	1312.72	2582.38	582.24	2084.34	1265.43	641.03	1180.01	871.36	981.38	2339.65	1683.26
M359T57	(M+H)+	1-Stearoyl-sn-glycerol	359.32	57.25	118301.07	18910.71	37096.08	20520.77	75060.49	47580.57	23619.63	59436.92	24692.42	24123.68	38692.59	19994.25
M524T341	(M+H)+	1-Stearoyl-sn-glycerol 3-phosphocholine	524.37	341.10	20043.19	21038.92	15988.74	12732.47	17188.92	14072.98	14931.13	21675.11	19491.71	17157.82	17351.55	18148.08
M383T56	(M+H-H2O)+	25-hydroxyvitamin D3	383.33	55.51	13711.45	16818.27	17764.65	16049.01	18984.61	18657.42	19757.46	25025.20	20662.96	15258.76	18418.25	16187.23

M151T115	(M+CH3COO+2H)+	2-Ethoxyethanol	151.10	114.91	44860.61	9208.93	39249.76	15726.48	9488.92	22227.07	5738.59	24279.34	8929.97	9267.52	72618.56	12289.19
M299T141	(M+NH4)+	2'-O-methyladenosine	299.15	141.37	2126.74	1291.49	24419.63	1367.54	1285.91	1239.60	1804.62	1413.23	1411.51	2100.72	1605.22	1281.61
M324T855	(M-2H+3Na)+	2'-O-methylcytidine	324.06	854.50	1370.62	1659.30	1702.74	1664.83	1519.17	1796.72	1988.25	1616.08	1860.48	2278.67	2485.43	2142.47
M130T118	(M+H)+	2-Pyrrolidineacetic acid	130.09	118.46	60388.59	49521.44	47879.72	50069.92	48431.42	57784.02	57807.12	39671.88	58367.11	54837.36	61237.79	47374.21
M104T80	(M+H)+	3-Aminobutanoic acid	104.07	80.23	6772.43	4998.56	4697.12	5011.58	3862.80	6964.39	4458.60	4909.50	5304.03	7842.74	7787.30	6655.99
M320T146	(M+K)+	3'-O-methyladenosine	320.08	145.59	5107.57	6335.81	6344.38	6397.03	5541.11	5230.41	6304.50	5404.16	5252.03	6945.21	5869.58	5433.64
M187T889	(M+H)+	3-Phospho-D-glycerate	187.00	888.92	899.61	964.98	952.47	841.23	822.07	986.73	906.17	652.16	902.29	1137.35	1035.86	885.65
M146T678	(M+H)+	4-Guanidinobutyric acid	146.09	677.95	2001.12	1448.93	1682.70	1998.69	1602.20	1479.93	2016.30	2124.31	1628.46	1812.58	2177.90	1906.33
M252T262	(M+H)+	5'-Deoxyadenosine	252.11	261.59	1212.45	1541.43	1644.14	844.57	967.76	953.90	1143.90	1150.22	1120.49	3289.42	3362.73	2900.81
M810T820	(M+H)+	Acetyl coenzyme A (Acetyl-CoA)	810.13	820.01	1525.17	1153.85	1292.55	1319.97	1601.46	1338.92	867.89	874.74	848.95	1376.59	1046.84	1048.33
M204T580	(M+H)+	Acetylcarnitine	204.12	579.87	2583.15	3066.09	3083.45	3079.57	3087.20	3251.36	2425.73	2209.13	2429.61	6243.99	6433.51	5875.09
M146T718	(M+H)+	Acetylcholine	146.12	718.45	50676.99	48951.19	48209.91	45100.33	42956.15	53847.70	48678.92	37089.26	39759.21	77990.23	86164.86	73337.79
M136T294	(M+H)+	Adenine	136.06	293.62	71680.65	48252.42	48454.47	51013.89	28291.58	35902.04	36562.04	18336.06	17876.60	78069.65	85006.62	69767.85
M268T315	(M+H)+	Adenosine	268.10	314.84	34664.66	41175.29	33538.94	31712.79	27937.99	38811.88	51578.78	36187.92	44867.73	62373.20	70994.30	54329.85
M428T967	(M+H)+	Adenosine 5'-diphosphate (ADP)	428.04	967.50	19373.30	6039.48	3539.80	4432.99	2693.80	4317.75	25579.78	3683.76	4142.40	4008.61	4928.45	3139.24
M450T888	(M+Na)+	Adenosine 5'-phosphosulfate (APS)	450.02	888.43	2125.11	2246.42	1997.71	1521.84	1400.49	1778.04	1639.05	1746.19	2045.31	2130.35	2660.58	1986.16

M508T940	(M+H)+	Adenosine 5'-triphosphate (ATP)	508.00	939.72	217354.72	274574.84	199507.46	247113.38	238891.40	284193.13	251135.93	249917.82	236926.97	287446.38	281795.33	260616.46
M348T765	(M+H)+	Adenosine monophosphate (AMP)	348.07	764.79	811.44	848.88	918.78	1412.90	783.89	1607.31	975.58	681.99	951.24	1405.19	2042.41	2018.19
M560T815	(M+H)+	ADP-ribose	560.08	815.49	662.48	757.69	670.08	744.86	770.51	773.45	641.66	742.13	797.51	739.57	834.70	748.21
M243T69	(M+H-2H2O)+	all cis-(6,9,12)-Linolenic acid	243.21	68.62	19828.33	25999.58	14901.40	14861.91	14704.31	23443.53	19852.73	17093.49	17897.25	15054.52	13685.76	13927.30
M269T415	(M+H)+	Allopurinol riboside	269.09	415.33	4064.05	1614.55	1929.21	4201.77	4143.08	4190.91	5939.55	6154.22	5880.80	4003.03	4463.85	4601.25
M323T973	(M+H-H2O)+	Alpha-D-Glucose 1,6-bisphosphate	322.99	972.72	3005.69	3608.95	3031.40	4393.60	3687.49	3882.02	3573.47	2851.18	3038.71	4845.43	4712.83	3473.81
M243T915	(M+H-H2O)+	alpha-D-Glucose 1-phosphate	243.03	914.56	3925.01	4871.23	4030.52	4497.47	3716.03	5000.37	5369.05	4559.61	5989.56	4448.26	5742.71	4874.53
M114T80	(M+H-H2O)+	Aminocaproic acid	114.09	79.79	21003.86	15587.04	31864.03	16358.07	18693.53	40896.83	14312.70	26084.45	16504.16	18118.74	32528.18	31117.82
M90T696	(M+H)+	Beta-Alanine	90.05	696.31	1195.34	2517.32	1226.49	1447.38	1360.90	1423.75	3142.83	1377.33	1844.27	2021.76	2269.05	3350.27
M118T515	(M+H)+	Betaine	118.09	514.69	110534.00	46460.58	42083.87	55322.60	49825.15	107624.40	107965.39	60380.27	105945.07	53887.24	65476.46	59092.52
M104T507_2	M+	Choline	104.11	507.06	98092.43	64599.97	68164.62	111143.84	85593.99	94277.53	102686.77	113341.49	106195.39	189778.23	161735.42	185704.65
M210T915	(M+NH4)+	Citrate	210.06	915.17	1305.44	288.51	1349.96	1730.91	1459.92	1557.70	3363.01	1415.68	564.74	2763.24	3614.32	4723.25
M132T679	(M+H)+	Creatine	132.08	678.85	6289.17	6526.94	4363.35	6189.62	5224.33	6168.92	8048.24	13173.25	14002.33	8549.60	9805.23	9385.63
M114T312	(M+H)+	Creatinine	114.07	311.70	3705.25	7611.79	4527.30	4007.05	5126.84	4994.25	3864.38	4842.01	5455.47	6699.32	4319.41	5714.08
M100T414	(M+H)+	Cyclohexylamine	100.11	413.91	1705.33	1418.37	1541.02	2116.00	2269.51	1644.62	2435.44	2278.67	2103.32	2836.81	2513.55	3051.16
M244T455	(M+H)+	Cytidine	244.09	454.87	7625.78	8262.71	6876.35	7411.32	6690.78	8217.98	9732.58	8049.39	8814.27	4435.81	5221.27	4034.54

M404T934	(M+H)+	Cytidine 5'-diphosphate (CDP)	404.02	934.27	4282.59	4184.40	4220.31	4193.92	4502.03	6841.46	4777.63	5318.81	4338.47	5100.32	5980.46	5616.70
M489T854	(M+H)+	Cytidine 5'-diphosphocholine (CDP-choline)	489.11	854.24	674.58	838.30	773.98	671.26	680.98	758.85	736.03	658.97	719.94	980.30	1098.87	976.96
M615T855	(M+H)+	Cytidine monophosphate N-acetylneuraminic acid	615.15	854.53	3879.55	4006.06	4184.56	4435.16	4286.64	4567.34	5190.02	4351.73	4719.29	5679.09	6368.54	5664.05
M484T971	(M+H)+	Cytidine triphosphate (CTP)	483.99	971.08	23580.79	26837.84	21175.32	27248.72	24042.19	27580.18	26911.15	21743.47	22521.41	26072.73	30375.59	21760.92
M201T763	(M+H)+	D-Erythrose 4-phosphate	201.02	763.49	1730.80	1993.47	2446.71	3486.77	2591.21	4128.25	1823.01	1622.13	1527.15	3357.76	4928.27	3982.57
M341T974	(M+H)+	D-Fructose 1,6-bisphosphate	341.00	974.19	5427.84	6500.61	7170.57	7407.15	6265.74	7371.37	7781.15	6778.99	6038.19	8372.68	8532.62	6987.03
M278T880	(M+NH4)+	D-Glucose 6-phosphate	278.06	879.70	843.40	887.30	1130.86	1025.54	876.36	1247.74	1109.25	1705.67	1026.62	957.68	913.92	793.32
M70T599	(M+H-2H2O)+	Diethanolamine	70.06	599.03	34204.99	38074.92	35386.94	37249.63	35342.77	43212.99	36083.84	30552.19	30922.40	34648.97	41247.29	34212.23
M162T787	(M+H)+	DL-2-Aminoadipic acid	162.08	787.05	641.46	621.85	687.09	592.56	620.90	1069.64	1253.33	981.10	795.53	1028.30	1152.04	1076.10
M131T488	(M+H-2H2O)+	DL-3-Phenyllactic acid	131.05	488.47	2884.54	3021.59	2638.66	2665.64	2461.86	2736.42	2933.01	2231.59	2617.59	3142.53	3176.70	2794.51
M207T426	(M+CH3CN+H)+	DL-Phenylalanine	207.11	425.94	3941.15	3896.42	4195.81	5363.32	3841.25	3349.35	6888.67	7331.31	4985.91	10186.25	6142.63	7457.14
M360T769	(M+NH4)+	D-Maltose	360.15	768.84	1024.32	1099.14	1108.00	1381.13	1586.63	1992.91	1968.78	1813.93	1974.16	3108.54	2845.19	2628.80
M198T589	(M+NH4)+	D-Mannose	198.10	588.90	31273.07	32797.23	26474.58	27238.28	19061.30	67396.24	25269.22	23433.77	22906.30	25244.24	53651.43	15826.47
M136T570	(M+H-H2O)+	Dopamine	136.08	570.38	5706.92	6918.51	7635.31	6392.96	6180.24	7926.73	6941.37	6488.77	6970.13	7555.38	7726.69	7783.95
M116T632	(M+H)+	D-Proline	116.07	631.74	43785.12	45839.76	54656.11	62508.78	42261.46	58466.77	42547.13	39738.07	27837.03	44759.63	38964.66	36291.03
M338T55	(M+H)+	Erucamide	338.34	55.29	57679.37	16729.27	59630.88	17065.20	20077.95	29587.73	12597.54	43926.93	52866.56	51398.30	77302.75	67431.28

M786T766	(M+H)+	Flavin adenine dinucleotide (FAD)	786.16	765.92	1858.84	1821.69	1862.08	2268.40	1958.96	2556.21	2205.46	2098.21	2241.34	2709.57	3297.76	2948.49
M457T781	(M+H)+	Flavin mononucleotide (FMN)	457.11	780.96	1124.65	1372.05	1247.54	1301.88	1021.46	1299.09	1473.77	1142.72	1404.23	1766.15	2276.57	2018.89
M442T831	(M+H)+	Folate	442.15	830.77	861.42	926.53	867.21	896.91	641.64	769.84	828.95	574.40	744.16	519.21	621.57	647.54
M277T890	(M+H)+	gamma-L-Glutamyl-L-glutamic acid	277.10	890.23	2296.74	2254.20	2362.44	2199.04	1908.05	2661.36	3378.67	2011.93	2451.70	3329.96	2981.50	2490.33
M606T911	(M+H)+	GDP-glucose	606.08	911.25	1229.14	1386.37	1374.05	1479.98	1328.77	1650.62	1716.65	1357.63	1343.14	1540.43	2198.59	1712.39
M590T878	(M+H)+	GDP-L-Fucose	590.09	878.49	1290.37	1532.00	1415.23	1430.52	1383.98	1672.12	1390.12	1267.20	1583.12	1364.94	1753.35	1525.45
M308T823	(M+H)+	Glutathione	308.09	823.43	16351.46	26704.94	26537.81	33429.67	29520.36	42541.62	75168.67	36271.72	30158.37	11116.28	21123.70	32485.34
M613T964	(M+H)+	Glutathione disulfide	613.16	964.23	190552.74	169335.18	163074.96	122300.58	185961.87	161289.09	142965.74	140023.52	175341.53	269109.46	222608.09	135578.99
M341T57	(M+H-H2O)+	Glycerol 1-octadecanoate	341.30	56.57	39762.35	9109.01	17933.87	9447.60	29552.50	16956.28	9823.13	24155.31	12841.92	11453.60	19409.95	13995.46
M173T835	(M+H)+	Glycerol 3-phosphate	173.02	834.67	4839.75	6119.96	5269.35	8279.17	7403.29	9527.31	3176.82	2605.29	2748.74	1829.09	2956.25	2673.97
M258T743	M+	Glycerophosphocholine	258.11	742.60	38152.16	39421.92	35744.13	38775.25	38031.76	40302.71	47270.26	42189.02	37685.01	81030.33	84139.40	80976.41
M231T599	M+	Glycyl-Arginine	231.13	599.04	1759.92	2302.48	1832.10	1978.78	1555.92	2699.84	1648.70	1079.55	1655.09	1496.25	2322.03	1356.43
M191T828	(M+H)+	Glycyl-Aspartate	191.07	828.29	1336.37	1421.12	1291.82	1327.39	1298.13	1441.84	1533.96	1336.56	1290.73	1992.95	1887.48	1591.03
M204T684	(M+H)+	Glycyl-Glutamine	204.10	683.58	2345.62	1579.07	823.19	789.21	2345.58	767.81	346.49	1171.73	1150.70	820.24	740.51	264.06
M284T494	(M+H)+	Guanosine	284.10	494.47	3107.64	2614.84	3071.72	2599.65	2680.00	3907.27	4785.69	3494.86	4011.72	2605.86	3363.11	2803.56
M444T878	(M+H)+	Guanosine 5'-diphosphate (GDP)	444.03	878.40	810.71	952.26	867.33	823.94	855.00	948.15	953.16	824.78	994.89	836.34	1083.80	954.39

M524T976	(M+H)+	Guanosine 5'-triphosphate (GTP)	524.00	976.32	23876.61	26490.03	23820.65	24170.02	22540.63	29340.86	29536.07	29319.95	27652.42	30638.23	33970.71	24521.78
M137T413	(M+H)+	Hypoxanthine	137.04	412.90	4147.00	4445.88	5653.91	6173.15	5614.08	4131.80	6615.69	8112.55	8523.89	7059.09	6190.77	6373.25
M69T111	(M+H)+	Imidazole	69.04	111.25	3669.86	2045.24	1515.87	2023.12	2202.58	762.07	3552.24	1491.11	1412.54	2034.03	2629.79	1934.35
M98T133	(M+H-2H2O)+	Iminodiacetic acid	98.02	133.07	1267.67	1740.43	1275.59	1373.35	1244.90	1359.55	2436.56	1784.69	1380.50	2699.82	3545.97	2707.25
M188T485	(M+H-H2O)+	Indolelactic acid	188.07	485.42	31919.90	35131.65	34310.20	36398.95	27951.82	36177.44	37989.80	35662.71	28562.15	35211.83	38843.26	37264.56
M203T75	(M+H)+	Isoleucyl-Alanine	203.14	75.43	3557.22	3482.15	3299.87	3946.68	2417.32	3970.90	4427.06	3658.03	3632.91	4864.54	4911.67	5054.47
M133T725	(M+H)+	L-Asparagine	133.06	724.89	5373.11	4469.11	5864.96	6318.58	7251.83	4936.30	7781.17	5466.02	5811.67	6252.05	3915.65	7520.57
M134T776	(M+H)+	L-Aspartate	134.04	776.24	26063.31	27479.13	24816.72	23129.05	22324.87	27675.14	43394.95	35587.11	41171.49	53342.24	60099.03	50574.54
M162T676	(M+H)+	L-Carnitine	162.11	675.88	26528.44	21497.62	23046.40	29351.12	26638.28	23757.87	21088.42	24113.46	21239.18	49665.54	54878.47	55794.56
M176T750	(M+H)+	L-Citrulline	176.10	750.17	10581.09	9263.85	10257.72	6374.17	5928.19	6502.45	10013.69	6982.59	11850.03	12120.59	13057.49	12407.37
M170T704	(M+H)+	L-Cysteic acid	170.01	703.55	1501.82	1102.41	1218.33	1411.38	1359.63	1468.56	1100.01	1360.51	1304.10	805.86	1123.55	1251.50
M148T765_2	(M+H)+	L-Glutamate	148.06	764.91	349222.94	394227.51	351536.75	372380.09	340065.98	403245.55	398340.90	348643.65	402357.27	453391.04	472112.97	445782.35
M147T714	(M+H)+	L-Glutamine	147.08	713.92	395714.35	377521.96	400717.20	377860.19	352596.17	455331.28	410425.78	400006.92	339215.33	464912.83	453822.80	420486.14
M196T199	(M+NH4)+	L-Gulonic gamma-lactone	196.08	198.80	81557.48	94687.63	36879.23	18289.18	56489.80	62713.97	43976.64	29388.17	47154.94	38170.01	35509.91	37164.18
M156T839	(M+H)+	L-Histidine	156.08	838.66	2944.09	2755.07	5137.78	2484.87	2469.72	4860.30	2879.54	2824.22	3300.92	3106.62	976.45	3174.24
M132T522	(M+H)+	L-Isoleucine	132.10	522.26	207551.72	185534.48	185514.91	215094.89	189087.48	191836.97	250287.07	169329.92	195803.35	211319.63	218257.83	209435.24

M132T501	(M+H)+	L-Leucine	132.10	501.40	187523.46	194799.69	176353.07	165571.43	150539.81	189690.29	204257.09	168850.28	170958.01	180832.49	193117.40	173880.13
M150T549	(M+H)+	L-Methionine	150.06	549.16	25526.30	26812.62	25388.45	21569.49	18739.84	24329.02	26833.57	18203.10	18992.19	33265.91	27083.49	35058.64
M176T522	(M-H+2Na)+	L-Norleucine	176.07	521.52	2267.72	1710.86	2043.38	2729.19	1557.69	1856.67	3598.70	2317.84	2512.40	2496.76	3668.31	4553.39
M166T488_2	(M+H)+	L-Phenylalanine	166.09	488.00	202646.96	220910.07	200682.32	200915.66	186052.85	225040.94	233326.60	192492.17	187747.57	223735.08	240257.24	218751.42
M157T618	(M+CH3CN+H)+	L-Proline	157.10	618.05	9670.16	9238.17	9193.33	9589.63	7245.20	11008.92	11370.79	11044.32	8925.84	19964.93	13089.58	13081.79
M147T836	(M+NH4)+	L-Pyroglutamic acid	147.08	836.26	10068.20	5113.45	4211.74	5706.35	9117.81	5163.91	5525.02	4328.57	4103.59	4901.31	4378.81	5601.49
M106T757	(M+H)+	L-Serine	106.05	756.77	2792.83	2835.81	2179.42	2491.54	3318.91	1345.57	1730.32	2166.66	3234.41	2435.65	3308.82	2876.04
M189T513	(M+H)+	L-Targinine	189.13	513.14	4026.33	2935.77	3794.94	3960.28	3138.89	4187.41	3198.22	3375.61	3217.57	4412.21	5705.96	3165.03
M120T684	(M+H)+	L-Threonine	120.06	684.31	26368.42	37017.21	25934.79	27317.69	24051.50	28467.57	30477.43	37465.85	28288.84	31516.62	35064.97	34332.14
M227T484	(M+Na)+	L-Tryptophan	227.08	484.12	4036.60	3548.62	3247.72	4064.33	3055.57	3332.48	4514.56	3796.07	4121.15	3301.64	3944.34	3834.97
M182T630	(M+H)+	L-Tyrosine	182.08	629.61	3484.45	1987.75	1778.97	2604.24	2262.05	2635.62	1959.21	7287.74	2272.29	2159.07	2602.55	8315.06
M243T82_1	(M+H)+	Lumichrome	243.09	82.37	7514.44	7395.21	5348.89	6329.18	5804.44	8128.73	3782.90	4138.56	4811.49	6994.69	9891.28	6960.88
M159T502_2	(M+CH3CN+H)+	L-Valine	159.11	501.70	1915.09	2328.43	2312.52	2003.48	1728.52	1987.40	2955.33	2709.37	2757.91	3965.89	2415.32	2685.12
M468T357	(M+H)+	LysoPC(14:0)	468.31	357.23	26734.39	36513.77	37816.17	18865.09	15884.05	20631.75	19418.50	36810.35	17918.33	14223.09	31351.76	29644.58
M496T333_4	(M+H)+	LysoPC(16:0)	496.34	332.57	23084.19	21016.27	24341.87	25496.66	25299.44	19903.66	20110.50	29229.62	24682.63	25884.31	23833.80	23555.58
M523T347	M+	LysoPC(18:0)	523.36	347.33	46001.21	12740.56	11348.09	29869.93	29723.08	32416.22	11604.77	13206.62	12387.77	12459.41	13264.00	32761.97

M522T344	(M+H)+	LysoPC(18:1(9Z))	522.35	344.02	184298.09	88788.05	91856.32	88773.68	88471.81	96529.23	97968.90	111728.55	103743.87	97663.93	97933.73	95027.59
M134T208	(M+H)+	Mandelonitrile	134.06	207.78	21530.22	21988.44	21755.51	22089.22	18820.91	25169.78	25555.53	23092.73	20736.24	22185.65	17563.89	23690.95
M303T59_1	(M+H)+	MG(14:0)	303.25	58.90	9656.01	19948.48	14851.98	24381.82	16018.07	14976.02	18766.76	13679.44	11383.98	19793.16	12150.28	15079.28
M331T58_2	(M+H)+	MG(16:0)	331.28	57.72	279429.11	176470.70	187457.78	151208.74	268859.23	200378.40	129611.98	168059.50	182309.59	232357.58	210251.95	188793.92
M198T758	(M+NH4)+	myo-Inositol	198.10	758.39	55104.21	55362.41	56394.33	64988.67	60586.24	67994.38	69030.19	60242.77	56709.54	81352.75	87757.43	71969.57
M189T606	(M+H)+	N-Acetylglutamine	189.09	605.54	1865.95	1773.84	1616.80	1633.87	1668.00	1829.16	1759.44	1604.65	1617.46	1895.46	2123.93	1918.51
M192T399	(M+H)+	N-Acetyl-L-methionine	192.07	398.64	533.54	352.75	640.37	589.07	336.32	616.87	501.76	595.58	507.46	815.06	752.43	839.22
M131T629	(M+H)+	N-Acetylputrescine	131.12	629.45	1881.27	1383.32	1742.43	2270.80	3460.13	2043.33	2284.01	2224.56	3509.03	2509.62	1236.49	3738.75
M219T68	(M+H)+	N-Acetylserotonin	219.11	68.28	16584.74	12416.76	11823.45	13249.82	8961.75	11070.27	12202.23	10697.64	10362.22	14063.64	18701.79	14266.60
M192T211	(M+H-H2O)+	N-Benzyloxycarbonylglycine	192.06	211.23	405.37	4286.63	1621.45	4088.10	1943.61	2526.45	2812.31	3366.15	3693.08	1221.50	2624.25	5438.62
M123T115	(M+H)+	Nicotinamide	123.05	114.52	46979.48	36107.10	30467.09	24309.71	28847.18	27526.35	21204.82	21614.69	22583.88	22388.11	24961.12	21323.01
M664T920	(M+H)+	Nicotinamide adenine dinucleotide (NAD)	664.12	920.17	1197.71	1728.64	1807.66	2105.84	2114.38	1948.44	1748.55	1117.72	2109.17	2462.75	1903.89	4348.83
M744T957	(M+H)+	Nicotinamide adenine dinucleotide phosphate (NADP)	744.08	956.96	5304.76	5987.91	5105.43	5488.82	5655.88	6683.78	6570.95	5565.92	6462.92	7926.18	8190.75	6098.93
M124T418	(M+H)+	Nicotinate	124.04	417.52	19536.66	11286.43	12609.50	20251.68	19382.13	14524.00	23996.72	29705.88	27795.74	27918.42	27904.65	26652.47
M326T60	(M+H)+	N-Oleoylethanolamine	326.30	59.51	5063.33	5256.67	5980.96	5238.01	6554.76	5122.76	6385.22	8436.20	7653.21	7723.93	10658.86	10612.07
M340T81	(M+H)+	Oleoyl glycine	340.28	81.14	16034.90	13318.48	11811.86	10720.96	9978.47	11574.27	12283.48	12994.74	10828.77	25602.97	29693.61	30053.87

M220T527	(M+H)+	Pantothenate	220.12	527.43	124364.62	65103.54	99208.68	154027.63	167508.32	86114.63	167586.25	188925.07	189380.20	205580.74	187860.53	212417.60
M717T266	(M+H-H2O)+	PC(16:0/16:0)	716.56	266.25	7201.15	947.77	1847.88	600.04	1380.34	887.35	755.81	744.89	1472.13	721.92	981.42	1264.72
M787T150	(M+H)+	PC(18:1(9Z)/18:1(9Z))	786.60	149.64	68549.59	5815.48	32665.88	3887.75	12461.12	2228.07	3202.92	6219.96	5375.00	14361.41	14816.61	10141.83
M212T851	(M+H)+	Phosphocreatine	212.04	851.27	70491.74	78519.54	79389.35	78534.06	74964.54	90348.06	103700.94	92480.87	98878.49	125894.58	124143.34	115299.85
M169T885	(M+H)+	Phosphoenolpyruvate	168.99	885.48	839.26	843.38	868.56	859.22	782.50	947.74	795.68	910.21	954.75	1402.29	2011.96	1252.66
M184T921	(M+H)+	Phosphorylcholine	184.07	921.16	475559.77	512088.03	468740.93	573924.38	502579.54	610365.63	527557.43	518514.69	527643.52	490903.14	512154.80	457884.00
M124T623	(M+H)+	Picolinic acid	124.04	622.90	1029.39	1641.63	1652.47	1709.16	1281.24	1328.27	1560.51	1670.35	1611.48	1999.02	1919.07	1696.97
M245T801	(M+H)+	Prolyl-Glutamate	245.11	801.40	885.28	922.99	791.59	979.90	820.42	1223.62	1148.67	1004.59	902.00	1534.11	1440.84	1328.34
M263T874	(M+CH3COO+2H)+	Prolyl-Serine	263.12	873.63	1647.50	2584.92	2278.85	2197.38	2278.36	2915.64	2971.69	2386.30	2414.87	3796.22	2845.38	3193.72
M170T207_2	(M+H)+	Pyridoxine	170.08	206.90	567964.73	577336.37	545575.60	507324.46	429249.40	494316.07	534557.77	431222.91	437320.47	583427.48	480168.50	561801.65
M666T776	(M+H)+	Reduced nicotinamide adenine dinucleotide (NADH)	666.13	776.12	6384.44	8042.91	7697.22	9577.48	7109.86	8221.04	8223.76	5393.95	6006.98	5608.76	8228.34	9034.90
M377T399	(M+H)+	Riboflavin	377.15	398.81	1614.82	875.16	1359.09	1779.89	1650.73	1149.66	1854.91	2147.26	2025.53	2100.44	2280.52	2024.84
M298T144	(M+H)+	S-Methyl-5'-thioadenosine	298.10	144.25	349664.80	422808.85	353893.09	425091.17	359275.47	394746.20	384424.42	366818.41	327139.82	464682.16	416429.00	407785.96
M216T756	(M+H)+	sn-Glycerol 3-phosphoethanolamine	216.06	755.98	1810.43	1953.99	1929.89	1966.76	1781.67	2082.25	2064.65	2017.36	2161.88	4310.07	4175.07	4008.01
M126T565	(M+H)+	Taurine	126.02	564.65	47961.89	51868.58	49892.35	53743.15	51890.40	56363.43	63830.29	60170.32	59196.15	82263.31	89382.59	76330.60
M265T672	M+	Thiamine	265.11	672.41	18314.34	16516.44	17011.32	20117.37	15290.08	16142.95	16557.23	19670.53	15188.44	19501.78	21932.99	22307.68

M425T977	M+	Thiamine pyrophosphate (TPP)	425.04	977.41	3792.63	4272.44	3832.75	2743.04	666.19	4012.29	669.21	4217.76	4302.91	6605.69	6883.07	4400.47
M165T570	(M+H)+	trans-2-Hydroxycinnamic acid	165.05	569.90	9791.65	11867.74	12058.68	10307.15	10167.36	13005.51	12717.04	11326.45	11558.08	12813.86	13941.26	12450.86
M149T488	(M+H)+	trans-cinnamate	149.06	488.42	4619.08	6174.39	5346.11	4675.20	5781.87	6270.03	5669.65	5120.57	5984.89	5902.66	6220.26	5909.88
M150T277	(M+H)+	Triethanolamine	150.11	276.55	4805.82	11536.15	8166.07	32179.68	60883.59	8972.42	12587.27	118338.33	12918.87	6070.37	16777.46	6809.92
M120T488	(M+H-H2O)+	Tyramine	120.08	487.98	109705.89	122529.86	106235.54	107415.11	100838.24	120836.90	121949.36	104691.90	103318.89	127270.17	134630.61	120292.58
M567T859	(M+H)+	UDP-D-Galactose	567.06	858.90	6431.44	9115.10	6848.89	8401.41	7798.47	8991.60	9829.66	8010.66	9289.52	10571.26	10578.60	9487.73
M625T836	(M+NH4)+	UDP-N-acetylglucosamine	625.11	836.39	26360.41	28355.55	28117.34	29100.85	26321.49	33156.02	34480.02	25809.40	31949.58	41342.32	45820.25	44289.70
M113T150	(M+H)+	Uracil	113.03	149.84	6113.52	7186.84	6812.64	5020.35	4365.89	4844.46	2627.15	1055.95	4221.26	9713.34	10589.06	9639.30
M61T183	(M+H)+	Urea	61.04	182.89	2876.64	4664.71	3919.51	3531.76	3274.28	1631.21	4309.39	2477.72	2503.03	4891.16	2454.89	3615.04
M245T299	(M+H)+	Uridine	245.08	298.56	3184.58	3611.12	3473.81	2696.26	2478.28	2943.33	3348.41	2984.75	3234.81	3832.96	4046.81	3079.29
M405T912	(M+H)+	Uridine 5'-diphosphate (UDP)	405.01	911.51	4181.69	5868.22	5877.23	5147.74	4767.31	6818.11	6931.53	4556.90	5612.55	6552.85	7845.85	6838.71
M581T927	(M+H)+	Uridine 5'-diphosphoglucuronic acid (UDP-D-glucuronate)	581.04	927.24	4973.23	6764.94	5972.91	5903.00	5193.42	6958.08	7722.73	5896.69	6123.17	7517.20	9073.06	7327.66
M325T851	(M+H)+	Uridine 5'-monophosphate (UMP)	325.04	850.68	2244.58	2447.73	2677.55	2652.09	2661.09	2743.85	2830.55	2716.12	2742.98	3339.68	3469.11	3087.57
M485T959	(M+H)+	Uridine 5'-triphosphate (UTP)	484.98	959.45	22983.93	24440.51	23684.13	27332.97	24675.52	28433.52	30106.11	27133.32	23533.69	31423.40	28768.79	24400.68
M139T554	(M+H)+	Urocanic acid	139.05	554.37	3825.61	1141.92	1724.12	817.58	1902.96	1323.84	1795.29	2335.89	860.51	2234.28	5968.03	840.60

Table S5. Binding proteins of SMO in ONS76 cells expressing SMO^{WT} or SMO^{W535L} with treatment of LDE225 or DMSO.

SMO^{WT}+DMSO-1	SMO^{WT}+DMSO-1	SMO^{WT}+LDE225-1	SMO^{WT}+LDE225-1

Accession	Description	Accession	Description	Accession	Description	Accession	Description
4502891	methylosome subunit pICln [Homo sapiens]	4502891	methylosome subunit pICln [Homo sapiens]	4502891	methylosome subunit pICln [Homo sapiens]	4502891	methylosome subunit pICln [Homo sapiens]
4506687	40S ribosomal protein S15 [Homo sapiens]	4506687	40S ribosomal protein S15 [Homo sapiens]	4505821	prolactin-inducible protein precursor [Homo sapiens]	4505821	prolactin-inducible protein precursor [Homo sapiens]
4885377	histone H1.3 [Homo sapiens]	4885377	histone H1.3 [Homo sapiens]	4506613	60S ribosomal protein L22 proprotein [Homo sapiens]	4506613	60S ribosomal protein L22 proprotein [Homo sapiens]
5032099	smoothened homolog precursor [Homo sapiens]	5032099	smoothened homolog precursor [Homo sapiens]	4506687	40S ribosomal protein S15 [Homo sapiens]	4506687	40S ribosomal protein S15 [Homo sapiens]
5174449	histone H1x [Homo sapiens]	5174449	histone H1x [Homo sapiens]	4758638	peroxiredoxin-6 [Homo sapiens]	4758638	peroxiredoxin-6 [Homo sapiens]
7661958	bcl-2-associated transcription factor 1 isoform 1 [Homo sapiens]	7661958	bcl-2-associated transcription factor 1 isoform 1 [Homo sapiens]	4885165	cystatin-A [Homo sapiens]	4885165	cystatin-A [Homo sapiens]
9506651	BTB/POZ domain-containing protein KCTD5 [Homo sapiens]	9506651	BTB/POZ domain-containing protein KCTD5 [Homo sapiens]	4885377	histone H1.3 [Homo sapiens]	4885377	histone H1.3 [Homo sapiens]
13129110	methylosome protein 50 [Homo sapiens]	13129110	methylosome protein 50 [Homo sapiens]	5032099	smoothened homolog precursor [Homo sapiens]	5032099	smoothened homolog precursor [Homo sapiens]
13699824	kinesin-like protein KIF11 [Homo sapiens]	13699824	kinesin-like protein KIF11 [Homo sapiens]	5174449	histone H1x [Homo sapiens]	5174449	histone H1x [Homo sapiens]
14043070	heterogeneous nuclear ribonucleoprotein A1 isoform b [Homo sapiens]	14043070	heterogeneous nuclear ribonucleoprotein A1 isoform b [Homo sapiens]	5453555	GTP-binding nuclear protein Ran [Homo sapiens]	5453555	GTP-binding nuclear protein Ran [Homo sapiens]
20070220	protein arginine N-methyltransferase 5 isoform a [Homo sapiens]	20070220	protein arginine N-methyltransferase 5 isoform a [Homo sapiens]	5803225	14-3-3 protein epsilon [Homo sapiens]	5803225	14-3-3 protein epsilon [Homo sapiens]
28173564	keratin, type II cytoskeletal 73 [Homo sapiens]	28173564	keratin, type II cytoskeletal 73 [Homo sapiens]	7661958	bcl-2-associated transcription factor 1 isoform 1 [Homo sapiens]	7661958	bcl-2-associated transcription factor 1 isoform 1 [Homo sapiens]
29570791	casein kinase II subunit alpha isoform a [Homo sapiens]	29570791	casein kinase II subunit alpha isoform a [Homo sapiens]	7705696	thioredoxin domain-containing protein 12 precursor [Homo sapiens]	7705696	thioredoxin domain-containing protein 12 precursor [Homo sapiens]
39930523	uncharacterized protein C11orf84 [Homo sapiens]	39930523	uncharacterized protein C11orf84 [Homo sapiens]	9506651	BTB/POZ domain-containing protein KCTD5 [Homo sapiens]	9506651	BTB/POZ domain-containing protein KCTD5 [Homo sapiens]
41322916	plectin isoform 1 [Homo sapiens]	41322916	plectin isoform 1 [Homo sapiens]	13129110	methylosome protein 50 [Homo sapiens]	13129110	methylosome protein 50 [Homo sapiens]
53829365	BTB/POZ domain-containing protein KCTD2 [Homo sapiens]	53829365	BTB/POZ domain-containing protein KCTD2 [Homo sapiens]	13699824	kinesin-like protein KIF11 [Homo sapiens]	13699824	kinesin-like protein KIF11 [Homo sapiens]

57013276	tubulin alpha-1B chain [Homo sapiens]	57013276	tubulin alpha-1B chain [Homo sapiens]	14043070	heterogeneous nuclear ribonucleoprotein A1 isoform b [Homo sapiens]	14043070	heterogeneous nuclear ribonucleoprotein A1 isoform b [Homo sapiens]
1.12E+08	spindlin-1 [Homo sapiens]	1.12E+08	spindlin-1 [Homo sapiens]	14210488	dynactin subunit 5 isoform 1 [Homo sapiens]	14210488	dynactin subunit 5 isoform 1 [Homo sapiens]
1.17E+08	heterogeneous nuclear ribonucleoproteins C1/C2 isoform a [Homo sapiens]	1.17E+08	heterogeneous nuclear ribonucleoproteins C1/C2 isoform a [Homo sapiens]	14249398	PHD finger-like domain-containing protein 5A [Homo sapiens]	14249398	PHD finger-like domain-containing protein 5A [Homo sapiens]
1.24E+08	unconventional myosin-Ic isoform a [Homo sapiens]	1.24E+08	unconventional myosin-Ic isoform a [Homo sapiens]	14589951	DNA-directed RNA polymerases I, II, and III subunit RPABC1 [Homo sapiens]	14589951	DNA-directed RNA polymerases I, II, and III subunit RPABC1 [Homo sapiens]
1.26E+08	keratin, type II cytoskeletal 80 isoform K80 [Homo sapiens]	1.26E+08	keratin, type II cytoskeletal 80 isoform K80 [Homo sapiens]	20070220	protein arginine N-methyltransferase 5 isoform a [Homo sapiens]	20070220	protein arginine N-methyltransferase 5 isoform a [Homo sapiens]
1.34E+08	arginine and glutamate-rich protein 1 [Homo sapiens]	1.34E+08	arginine and glutamate-rich protein 1 [Homo sapiens]	20270371	carboxymethylenebutenolidase homolog [Homo sapiens]	20270371	carboxymethylenebutenolidase homolog [Homo sapiens]
1.55E+08	protein arginine N-methyltransferase 1 isoform 1 [Homo sapiens]	1.55E+08	protein arginine N-methyltransferase 1 isoform 1 [Homo sapiens]	28173564	keratin, type II cytoskeletal 73 [Homo sapiens]	28173564	keratin, type II cytoskeletal 73 [Homo sapiens]
1.95E+08	spectrin alpha chain, non-erythrocytic 1 isoform 1 [Homo sapiens]	1.95E+08	spectrin alpha chain, non-erythrocytic 1 isoform 1 [Homo sapiens]	33286418	pyruvate kinase isozymes M1/M2 isoform a [Homo sapiens]	33286418	pyruvate kinase isozymes M1/M2 isoform a [Homo sapiens]
3.21E+08	RPS10-NUDT3 protein [Homo sapiens]	3.21E+08	RPS10-NUDT3 protein [Homo sapiens]	53829365	BTB/POZ domain-containing protein KCTD2 [Homo sapiens]	53829365	BTB/POZ domain-containing protein KCTD2 [Homo sapiens]
3.87E+08	40S ribosomal protein S3 isoform 2 [Homo sapiens]	3.87E+08	40S ribosomal protein S3 isoform 2 [Homo sapiens]	57013276	tubulin alpha-1B chain [Homo sapiens]	57013276	tubulin alpha-1B chain [Homo sapiens]
4.01E+08	mitochondrial dicarboxylate carrier isoform 3 [Homo sapiens]	4.01E+08	mitochondrial dicarboxylate carrier isoform 3 [Homo sapiens]	67782356	corneodesmosin precursor [Homo sapiens]	67782356	corneodesmosin precursor [Homo sapiens]
				1.12E+08	spindlin-1 [Homo sapiens]	1.12E+08	spindlin-1 [Homo sapiens]
				1.26E+08	keratin, type II cytoskeletal 80 isoform K80 [Homo sapiens]	1.26E+08	keratin, type II cytoskeletal 80 isoform K80 [Homo sapiens]
				1.55E+08	protein arginine N-methyltransferase 1 isoform 1 [Homo sapiens]	1.55E+08	protein arginine N-methyltransferase 1 isoform 1 [Homo sapiens]
				1.95E+08	spectrin alpha chain, non-erythrocytic 1 isoform 1 [Homo sapiens]	1.95E+08	spectrin alpha chain, non-erythrocytic 1 isoform 1 [Homo sapiens]

				2.08E+08	dynactin subunit 4 isoform a [Homo sapiens]	2.08E+08	dynactin subunit 4 isoform a [Homo sapiens]
				3.08E+08	taperin [Homo sapiens]	3.08E+08	taperin [Homo sapiens]
				3.21E+08	RPS10-NUDT3 protein [Homo sapiens]	3.21E+08	RPS10-NUDT3 protein [Homo sapiens]
				3.67E+08	myosin-10 isoform 2 [Homo sapiens]	3.67E+08	myosin-10 isoform 2 [Homo sapiens]
				3.87E+08	40S ribosomal protein S3 isoform 2 [Homo sapiens]	3.87E+08	40S ribosomal protein S3 isoform 2 [Homo sapiens]
				4.32E+08	dedicator of cytokinesis protein 7 isoform 1 [Homo sapiens]	4.32E+08	dedicator of cytokinesis protein 7 isoform 1 [Homo sapiens]
**SMO^{W535L}+DMSO-1**		**SMO^{W535L}+DMSO-1**		**SMO^{W535L}+LDE225-1**		**SMO^{W535L}+LDE225-1**	
Accession	Description	Accession	Description	Accession	Description	Accession	Description
4502211	ADP-ribosylation factor 6 [Homo sapiens]	4502211	ADP-ribosylation factor 6 [Homo sapiens]	4502211	ADP-ribosylation factor 6 [Homo sapiens]	4502211	ADP-ribosylation factor 6 [Homo sapiens]
4502891	methylosome subunit pICln [Homo sapiens]	4502891	methylosome subunit pICln [Homo sapiens]	4502709	cyclin-dependent kinase 1 isoform 1 [Homo sapiens]	4502709	cyclin-dependent kinase 1 isoform 1 [Homo sapiens]
4503507	eukaryotic translation initiation factor 2 subunit 3 [Homo sapiens]	4503507	eukaryotic translation initiation factor 2 subunit 3 [Homo sapiens]	4502891	methylosome subunit pICln [Homo sapiens]	4502891	methylosome subunit pICln [Homo sapiens]
4503509	eukaryotic translation initiation factor 3 subunit A [Homo sapiens]	4503509	eukaryotic translation initiation factor 3 subunit A [Homo sapiens]	4502899	clathrin light chain A isoform a [Homo sapiens]	4502899	clathrin light chain A isoform a [Homo sapiens]
4503515	eukaryotic translation initiation factor 3 subunit H [Homo sapiens]	4503515	eukaryotic translation initiation factor 3 subunit H [Homo sapiens]	4503507	eukaryotic translation initiation factor 2 subunit 3 [Homo sapiens]	4503507	eukaryotic translation initiation factor 2 subunit 3 [Homo sapiens]
4503519	eukaryotic translation initiation factor 3 subunit F [Homo sapiens]	4503519	eukaryotic translation initiation factor 3 subunit F [Homo sapiens]	4503521	eukaryotic translation initiation factor 3 subunit E [Homo sapiens]	4503521	eukaryotic translation initiation factor 3 subunit E [Homo sapiens]
4503521	eukaryotic translation initiation factor 3 subunit E [Homo sapiens]	4503521	eukaryotic translation initiation factor 3 subunit E [Homo sapiens]	4505753	phosphoglycerate mutase 1 [Homo sapiens]	4505753	phosphoglycerate mutase 1 [Homo sapiens]
4503971	rab GDP dissociation inhibitor alpha [Homo sapiens]	4503971	rab GDP dissociation inhibitor alpha [Homo sapiens]	4505821	prolactin-inducible protein precursor [Homo sapiens]	4505821	prolactin-inducible protein precursor [Homo sapiens]
4504897	importin subunit alpha-2 [Homo sapiens]	4504897	importin subunit alpha-2 [Homo sapiens]	4506031	palmitoyl-protein thioesterase 1 isoform 1 precursor [Homo sapiens]	4506031	palmitoyl-protein thioesterase 1 isoform 1 precursor [Homo sapiens]
4506031	palmitoyl-protein thioesterase 1 isoform 1 precursor [Homo sapiens]	4506031	palmitoyl-protein thioesterase 1 isoform 1 precursor [Homo sapiens]	4506189	proteasome subunit alpha type-7 [Homo sapiens]	4506189	proteasome subunit alpha type-7 [Homo sapiens]

4506341	ATP-binding cassette sub-family D member 3 isoform a [Homo sapiens]	4506341	ATP-binding cassette sub-family D member 3 isoform a [Homo sapiens]	4506201	proteasome subunit beta type-5 isoform 1 [Homo sapiens]	4506201	proteasome subunit beta type-5 isoform 1 [Homo sapiens]
4506613	60S ribosomal protein L22 proprotein [Homo sapiens]	4506613	60S ribosomal protein L22 proprotein [Homo sapiens]	4506341	ATP-binding cassette sub-family D member 3 isoform a [Homo sapiens]	4506341	ATP-binding cassette sub-family D member 3 isoform a [Homo sapiens]
4506631	60S ribosomal protein L30 [Homo sapiens]	4506631	60S ribosomal protein L30 [Homo sapiens]	4506407	ran-specific GTPase-activating protein [Homo sapiens]	4506407	ran-specific GTPase-activating protein [Homo sapiens]
4506643	60S ribosomal protein L37a [Homo sapiens]	4506643	60S ribosomal protein L37a [Homo sapiens]	4506613	60S ribosomal protein L22 proprotein [Homo sapiens]	4506613	60S ribosomal protein L22 proprotein [Homo sapiens]
4506687	40S ribosomal protein S15 [Homo sapiens]	4506687	40S ribosomal protein S15 [Homo sapiens]	4506643	60S ribosomal protein L37a [Homo sapiens]	4506643	60S ribosomal protein L37a [Homo sapiens]
4506901	serine/arginine-rich splicing factor 3 [Homo sapiens]	4506901	serine/arginine-rich splicing factor 3 [Homo sapiens]	4506687	40S ribosomal protein S15 [Homo sapiens]	4506687	40S ribosomal protein S15 [Homo sapiens]
4506975	solute carrier family 12 member 2 isoform 1 [Homo sapiens]	4506975	solute carrier family 12 member 2 isoform 1 [Homo sapiens]	4506753	ruvB-like 1 [Homo sapiens]	4506753	ruvB-like 1 [Homo sapiens]
4507115	fascin [Homo sapiens]	4507115	fascin [Homo sapiens]	4507115	fascin [Homo sapiens]	4507115	fascin [Homo sapiens]
4557511	death-associated protein kinase 3 [Homo sapiens]	4557511	death-associated protein kinase 3 [Homo sapiens]	4507475	protein-glutamine gamma-glutamyltransferase K [Homo sapiens]	4507475	protein-glutamine gamma-glutamyltransferase K [Homo sapiens]
4557795	merlin isoform 1 [Homo sapiens]	4557795	merlin isoform 1 [Homo sapiens]	4507555	thymopoietin isoform alpha [Homo sapiens]	4507555	thymopoietin isoform alpha [Homo sapiens]
4757766	rho GTPase-activating protein 1 [Homo sapiens]	4757766	rho GTPase-activating protein 1 [Homo sapiens]	4557795	merlin isoform 1 [Homo sapiens]	4557795	merlin isoform 1 [Homo sapiens]
4758638	peroxiredoxin-6 [Homo sapiens]	4758638	peroxiredoxin-6 [Homo sapiens]	4757766	rho GTPase-activating protein 1 [Homo sapiens]	4757766	rho GTPase-activating protein 1 [Homo sapiens]
4758648	kinesin-1 heavy chain [Homo sapiens]	4758648	kinesin-1 heavy chain [Homo sapiens]	4757880	mitotic checkpoint protein BUB3 isoform a [Homo sapiens]	4757880	mitotic checkpoint protein BUB3 isoform a [Homo sapiens]
4758876	polyadenylate-binding protein 2 [Homo sapiens]	4758876	polyadenylate-binding protein 2 [Homo sapiens]	4758304	protein disulfide-isomerase A4 precursor [Homo sapiens]	4758304	protein disulfide-isomerase A4 precursor [Homo sapiens]
5031571	actin-related protein 2 isoform b [Homo sapiens]	5031571	actin-related protein 2 isoform b [Homo sapiens]	4758638	peroxiredoxin-6 [Homo sapiens]	4758638	peroxiredoxin-6 [Homo sapiens]
5031595	actin-related protein 2/3 complex subunit 4 isoform a [Homo sapiens]	5031595	actin-related protein 2/3 complex subunit 4 isoform a [Homo sapiens]	4826730	serine/threonine-protein kinase mTOR [Homo sapiens]	4826730	serine/threonine-protein kinase mTOR [Homo sapiens]

5031875	lamin isoform C [Homo sapiens]	5031875	lamin isoform C [Homo sapiens]	4826898	profilin-1 [Homo sapiens]	4826898	profilin-1 [Homo sapiens]
5032099	smoothened homolog precursor [Homo sapiens]	5032099	smoothened homolog precursor [Homo sapiens]	4885165	cystatin-A [Homo sapiens]	4885165	cystatin-A [Homo sapiens]
5174449	histone H1x [Homo sapiens]	5174449	histone H1x [Homo sapiens]	4885661	tyrosine-protein kinase Yes [Homo sapiens]	4885661	tyrosine-protein kinase Yes [Homo sapiens]
5453607	T-complex protein 1 subunit eta isoform a [Homo sapiens]	5453607	T-complex protein 1 subunit eta isoform a [Homo sapiens]	5031571	actin-related protein 2 isoform b [Homo sapiens]	5031571	actin-related protein 2 isoform b [Homo sapiens]
5453998	importin-7 [Homo sapiens]	5453998	importin-7 [Homo sapiens]	5031595	actin-related protein 2/3 complex subunit 4 isoform a [Homo sapiens]	5031595	actin-related protein 2/3 complex subunit 4 isoform a [Homo sapiens]
5454158	valine--tRNA ligase [Homo sapiens]	5454158	valine--tRNA ligase [Homo sapiens]	5031635	cofilin-1 [Homo sapiens]	5031635	cofilin-1 [Homo sapiens]
5730009	zinc finger protein RFP [Homo sapiens]	5730009	zinc finger protein RFP [Homo sapiens]	5032099	smoothened homolog precursor [Homo sapiens]	5032099	smoothened homolog precursor [Homo sapiens]
5730023	ruvB-like 2 [Homo sapiens]	5730023	ruvB-like 2 [Homo sapiens]	5032137	vesicle-associated membrane protein 7 isoform 1 [Homo sapiens]	5032137	vesicle-associated membrane protein 7 isoform 1 [Homo sapiens]
5803225	14-3-3 protein epsilon [Homo sapiens]	5803225	14-3-3 protein epsilon [Homo sapiens]	5174449	histone H1x [Homo sapiens]	5174449	histone H1x [Homo sapiens]
5902122	spectrin beta chain, non-erythrocytic 2 [Homo sapiens]	5902122	spectrin beta chain, non-erythrocytic 2 [Homo sapiens]	5453555	GTP-binding nuclear protein Ran [Homo sapiens]	5453555	GTP-binding nuclear protein Ran [Homo sapiens]
6005757	FACT complex subunit SPT16 [Homo sapiens]	6005757	FACT complex subunit SPT16 [Homo sapiens]	5453607	T-complex protein 1 subunit eta isoform a [Homo sapiens]	5453607	T-complex protein 1 subunit eta isoform a [Homo sapiens]
6005830	plakophilin-3 [Homo sapiens]	6005830	plakophilin-3 [Homo sapiens]	5453629	dynactin subunit 2 isoform 1 [Homo sapiens]	5453629	dynactin subunit 2 isoform 1 [Homo sapiens]
6005942	transitional endoplasmic reticulum ATPase [Homo sapiens]	6005942	transitional endoplasmic reticulum ATPase [Homo sapiens]	5453998	importin-7 [Homo sapiens]	5453998	importin-7 [Homo sapiens]
6912280	activator of 90 kDa heat shock protein ATPase homolog 1 [Homo sapiens]	6912280	activator of 90 kDa heat shock protein ATPase homolog 1 [Homo sapiens]	5454052	14-3-3 protein sigma [Homo sapiens]	5454052	14-3-3 protein sigma [Homo sapiens]
7656952	calcyclin-binding protein isoform 1 [Homo sapiens]	7656952	calcyclin-binding protein isoform 1 [Homo sapiens]	5729953	nuclear migration protein nudC [Homo sapiens]	5729953	nuclear migration protein nudC [Homo sapiens]
7661858	rho GTPase-activating protein 11A isoform 1 [Homo sapiens]	7661858	rho GTPase-activating protein 11A isoform 1 [Homo sapiens]	5730009	zinc finger protein RFP [Homo sapiens]	5730009	zinc finger protein RFP [Homo sapiens]
7661878	kinesin-like protein KIF14 [Homo sapiens]	7661878	kinesin-like protein KIF14 [Homo sapiens]	5730023	ruvB-like 2 [Homo sapiens]	5730023	ruvB-like 2 [Homo sapiens]
7661958	bcl-2-associated transcription factor 1 isoform 1 [Homo sapiens]	7661958	bcl-2-associated transcription factor 1 isoform 1 [Homo sapiens]	5803225	14-3-3 protein epsilon [Homo sapiens]	5803225	14-3-3 protein epsilon [Homo sapiens]

7662126	signal-induced proliferation-associated 1-like protein 1 [Homo sapiens]	7662126	signal-induced proliferation-associated 1-like protein 1 [Homo sapiens]	5902122	spectrin beta chain, non-erythrocytic 2 [Homo sapiens]	5902122	spectrin beta chain, non-erythrocytic 2 [Homo sapiens]
7705696	thioredoxin domain-containing protein 12 precursor [Homo sapiens]	7705696	thioredoxin domain-containing protein 12 precursor [Homo sapiens]	6005757	FACT complex subunit SPT16 [Homo sapiens]	6005757	FACT complex subunit SPT16 [Homo sapiens]
7705706	40S ribosomal protein S27-like [Homo sapiens]	7705706	40S ribosomal protein S27-like [Homo sapiens]	6005830	plakophilin-3 [Homo sapiens]	6005830	plakophilin-3 [Homo sapiens]
8392875	UPF0468 protein C16orf80 [Homo sapiens]	8392875	UPF0468 protein C16orf80 [Homo sapiens]	6005942	transitional endoplasmic reticulum ATPase [Homo sapiens]	6005942	transitional endoplasmic reticulum ATPase [Homo sapiens]
8923448	39S ribosomal protein L16, mitochondrial [Homo sapiens]	8923448	39S ribosomal protein L16, mitochondrial [Homo sapiens]	6041669	NADH dehydrogenase [ubiquinone] 1 beta subcomplex subunit 4 isoform 1 [Homo sapiens]	6041669	NADH dehydrogenase [ubiquinone] 1 beta subcomplex subunit 4 isoform 1 [Homo sapiens]
8923712	actin-related protein 10 [Homo sapiens]	8923712	actin-related protein 10 [Homo sapiens]	6912280	activator of 90 kDa heat shock protein ATPase homolog 1 [Homo sapiens]	6912280	activator of 90 kDa heat shock protein ATPase homolog 1 [Homo sapiens]
9506651	BTB/POZ domain-containing protein KCTD5 [Homo sapiens]	9506651	BTB/POZ domain-containing protein KCTD5 [Homo sapiens]	7305053	myoferlin isoform a [Homo sapiens]	7305053	myoferlin isoform a [Homo sapiens]
9966881	nuclear pore complex protein Nup107 [Homo sapiens]	9966881	nuclear pore complex protein Nup107 [Homo sapiens]	7657198	probable dimethyladenosine transferase [Homo sapiens]	7657198	probable dimethyladenosine transferase [Homo sapiens]
9966913	actin-related protein 3B isoform 1 [Homo sapiens]	9966913	actin-related protein 3B isoform 1 [Homo sapiens]	7661844	coiled-coil domain-containing protein 22 [Homo sapiens]	7661844	coiled-coil domain-containing protein 22 [Homo sapiens]
10863889	U4/U6.U5 tri-snRNP-associated protein 1 [Homo sapiens]	10863889	U4/U6.U5 tri-snRNP-associated protein 1 [Homo sapiens]	7661858	rho GTPase-activating protein 11A isoform 1 [Homo sapiens]	7661858	rho GTPase-activating protein 11A isoform 1 [Homo sapiens]
10863927	peptidyl-prolyl cis-trans isomerase A [Homo sapiens]	10863927	peptidyl-prolyl cis-trans isomerase A [Homo sapiens]	7661878	kinesin-like protein KIF14 [Homo sapiens]	7661878	kinesin-like protein KIF14 [Homo sapiens]
11125770	aminoacyl tRNA synthase complex-interacting multifunctional protein 2 [Homo sapiens]	11125770	aminoacyl tRNA synthase complex-interacting multifunctional protein 2 [Homo sapiens]	7661958	bcl-2-associated transcription factor 1 isoform 1 [Homo sapiens]	7661958	bcl-2-associated transcription factor 1 isoform 1 [Homo sapiens]
11545731	gigaxonin [Homo sapiens]	11545731	gigaxonin [Homo sapiens]	7662126	signal-induced proliferation-associated 1-like protein 1 [Homo sapiens]	7662126	signal-induced proliferation-associated 1-like protein 1 [Homo sapiens]
13129110	methylosome protein 50 [Homo sapiens]	13129110	methylosome protein 50 [Homo sapiens]	7705622	WASH complex subunit CCDC53 [Homo sapiens]	7705622	WASH complex subunit CCDC53 [Homo sapiens]
13259510	dynactin subunit 1 isoform 1 [Homo sapiens]	13259510	dynactin subunit 1 isoform 1 [Homo sapiens]	7705696	thioredoxin domain-containing protein 12 precursor [Homo sapiens]	7705696	thioredoxin domain-containing protein 12 precursor [Homo sapiens]

13569956	actin-related protein 2/3 complex subunit 5-like protein [Homo sapiens]	13569956	actin-related protein 2/3 complex subunit 5-like protein [Homo sapiens]	8922331	protein mago nashi homolog 2 [Homo sapiens]	8922331	protein mago nashi homolog 2 [Homo sapiens]
13654270	ribosomal biogenesis protein LAS1L isoform 1 [Homo sapiens]	13654270	ribosomal biogenesis protein LAS1L isoform 1 [Homo sapiens]	8922601	ADP-ribosylation factor-like protein 8B [Homo sapiens]	8922601	ADP-ribosylation factor-like protein 8B [Homo sapiens]
13699824	kinesin-like protein KIF11 [Homo sapiens]	13699824	kinesin-like protein KIF11 [Homo sapiens]	8923448	39S ribosomal protein L16, mitochondrial [Homo sapiens]	8923448	39S ribosomal protein L16, mitochondrial [Homo sapiens]
13775198	SH3 domain-binding glutamic acid-rich-like protein 3 [Homo sapiens]	13775198	SH3 domain-binding glutamic acid-rich-like protein 3 [Homo sapiens]	8923579	ragulator complex protein LAMTOR1 [Homo sapiens]	8923579	ragulator complex protein LAMTOR1 [Homo sapiens]
13899317	SH3 domain-binding glutamic acid-rich-like protein 2 [Homo sapiens]	13899317	SH3 domain-binding glutamic acid-rich-like protein 2 [Homo sapiens]	8923712	actin-related protein 10 [Homo sapiens]	8923712	actin-related protein 10 [Homo sapiens]
14043070	heterogeneous nuclear ribonucleoprotein A1 isoform b [Homo sapiens]	14043070	heterogeneous nuclear ribonucleoprotein A1 isoform b [Homo sapiens]	9506651	BTB/POZ domain-containing protein KCTD5 [Homo sapiens]	9506651	BTB/POZ domain-containing protein KCTD5 [Homo sapiens]
14210488	dynactin subunit 5 isoform 1 [Homo sapiens]	14210488	dynactin subunit 5 isoform 1 [Homo sapiens]	9506689	exosome complex component RRP41 [Homo sapiens]	9506689	exosome complex component RRP41 [Homo sapiens]
14249342	alpha-internexin [Homo sapiens]	14249342	alpha-internexin [Homo sapiens]	9943848	gamma-adducin isoform a [Homo sapiens]	9943848	gamma-adducin isoform a [Homo sapiens]
14589847	protein transport protein Sec61 subunit alpha isoform 2 isoform a [Homo sapiens]	14589847	protein transport protein Sec61 subunit alpha isoform 2 isoform a [Homo sapiens]	9966913	actin-related protein 3B isoform 1 [Homo sapiens]	9966913	actin-related protein 3B isoform 1 [Homo sapiens]
14589889	cadherin-2 preproprotein [Homo sapiens]	14589889	cadherin-2 preproprotein [Homo sapiens]	10863927	peptidyl-prolyl cis-trans isomerase A [Homo sapiens]	10863927	peptidyl-prolyl cis-trans isomerase A [Homo sapiens]
14589951	DNA-directed RNA polymerases I, II, and III subunit RPABC1 [Homo sapiens]	14589951	DNA-directed RNA polymerases I, II, and III subunit RPABC1 [Homo sapiens]	11125770	aminoacyl tRNA synthase complex-interacting multifunctional protein 2 [Homo sapiens]	11125770	aminoacyl tRNA synthase complex-interacting multifunctional protein 2 [Homo sapiens]
15147337	E3 ubiquitin-protein ligase UBR5 [Homo sapiens]	15147337	E3 ubiquitin-protein ligase UBR5 [Homo sapiens]	11596859	39S ribosomal protein L17, mitochondrial [Homo sapiens]	11596859	39S ribosomal protein L17, mitochondrial [Homo sapiens]
15431316	keratin, type II cuticular Hb4 [Homo sapiens]	15431316	keratin, type II cuticular Hb4 [Homo sapiens]	11641247	Golgi-associated plant pathogenesis-related protein 1 [Homo sapiens]	11641247	Golgi-associated plant pathogenesis-related protein 1 [Homo sapiens]
16117789	60S ribosomal protein L34 [Homo sapiens]	16117789	60S ribosomal protein L34 [Homo sapiens]	12383056	microtubule-associated proteins 1A/1B light chain 3B [Homo sapiens]	12383056	microtubule-associated proteins 1A/1B light chain 3B [Homo sapiens]
16262452	cingulin [Homo sapiens]	16262452	cingulin [Homo sapiens]	13129110	methylosome protein 50 [Homo sapiens]	13129110	methylosome protein 50 [Homo sapiens]

16554629	WD repeat-containing protein 5 [Homo sapiens]	16554629	WD repeat-containing protein 5 [Homo sapiens]	13569956	actin-related protein 2/3 complex subunit 5-like protein [Homo sapiens]	13569956	actin-related protein 2/3 complex subunit 5-like protein [Homo sapiens]
16950593	28S ribosomal protein S21, mitochondrial [Homo sapiens]	16950593	28S ribosomal protein S21, mitochondrial [Homo sapiens]	13699824	kinesin-like protein KIF11 [Homo sapiens]	13699824	kinesin-like protein KIF11 [Homo sapiens]
17158023	protein FAM207A [Homo sapiens]	17158023	protein FAM207A [Homo sapiens]	13775198	SH3 domain-binding glutamic acid-rich-like protein 3 [Homo sapiens]	13775198	SH3 domain-binding glutamic acid-rich-like protein 3 [Homo sapiens]
17388799	dnaJ homolog subfamily B member 6 isoform a [Homo sapiens]	17388799	dnaJ homolog subfamily B member 6 isoform a [Homo sapiens]	13899317	SH3 domain-binding glutamic acid-rich-like protein 2 [Homo sapiens]	13899317	SH3 domain-binding glutamic acid-rich-like protein 2 [Homo sapiens]
18644728	nucleolar protein 6 alpha isoform [Homo sapiens]	18644728	nucleolar protein 6 alpha isoform [Homo sapiens]	13994151	PDZ and LIM domain protein 1 [Homo sapiens]	13994151	PDZ and LIM domain protein 1 [Homo sapiens]
19115964	1-phosphatidylinositol 4,5-bisphosphate phosphodiesterase delta-3 [Homo sapiens]	19115964	1-phosphatidylinositol 4,5-bisphosphate phosphodiesterase delta-3 [Homo sapiens]	14043070	heterogeneous nuclear ribonucleoprotein A1 isoform b [Homo sapiens]	14043070	heterogeneous nuclear ribonucleoprotein A1 isoform b [Homo sapiens]
19913369	transducin beta-like protein 3 [Homo sapiens]	19913369	transducin beta-like protein 3 [Homo sapiens]	14149607	H(+)/Cl(-) exchange transporter 7 isoform a [Homo sapiens]	14149607	H(+)/Cl(-) exchange transporter 7 isoform a [Homo sapiens]
20070220	protein arginine N-methyltransferase 5 isoform a [Homo sapiens]	20070220	protein arginine N-methyltransferase 5 isoform a [Homo sapiens]	14210488	dynactin subunit 5 isoform 1 [Homo sapiens]	14210488	dynactin subunit 5 isoform 1 [Homo sapiens]
21264616	epidermal growth factor receptor kinase substrate 8-like protein 2 [Homo sapiens]	21264616	epidermal growth factor receptor kinase substrate 8-like protein 2 [Homo sapiens]	14251209	chloride intracellular channel protein 1 [Homo sapiens]	14251209	chloride intracellular channel protein 1 [Homo sapiens]
21361282	serine/arginine-rich splicing factor 4 [Homo sapiens]	21361282	serine/arginine-rich splicing factor 4 [Homo sapiens]	14589889	cadherin-2 preproprotein [Homo sapiens]	14589889	cadherin-2 preproprotein [Homo sapiens]
21361576	nucleolar protein of 40 kDa [Homo sapiens]	21361576	nucleolar protein of 40 kDa [Homo sapiens]	15431316	keratin, type II cuticular Hb4 [Homo sapiens]	15431316	keratin, type II cuticular Hb4 [Homo sapiens]
21361794	cullin-associated NEDD8-dissociated protein 1 [Homo sapiens]	21361794	cullin-associated NEDD8-dissociated protein 1 [Homo sapiens]	16117789	60S ribosomal protein L34 [Homo sapiens]	16117789	60S ribosomal protein L34 [Homo sapiens]
22027646	angiomotin-like protein 1 [Homo sapiens]	22027646	angiomotin-like protein 1 [Homo sapiens]	16262452	cingulin [Homo sapiens]	16262452	cingulin [Homo sapiens]
22325356	PALM2-AKAP2 protein isoform 2 [Homo sapiens]	22325356	PALM2-AKAP2 protein isoform 2 [Homo sapiens]	17158023	protein FAM207A [Homo sapiens]	17158023	protein FAM207A [Homo sapiens]
24233517	inhibitor of nuclear factor kappa-B kinase-interacting protein isoform 1 [Homo sapiens]	24233517	inhibitor of nuclear factor kappa-B kinase-interacting protein isoform 1 [Homo sapiens]	17388799	dnaJ homolog subfamily B member 6 isoform a [Homo sapiens]	17388799	dnaJ homolog subfamily B member 6 isoform a [Homo sapiens]

24797086	importin-5 [Homo sapiens]	24797086	importin-5 [Homo sapiens]	19115964	1-phosphatidylinositol 4,5-bisphosphate phosphodiesterase delta-3 [Homo sapiens]	19115964	1-phosphatidylinositol 4,5-bisphosphate phosphodiesterase delta-3 [Homo sapiens]
25777602	26S proteasome non-ATPase regulatory subunit 2 [Homo sapiens]	25777602	26S proteasome non-ATPase regulatory subunit 2 [Homo sapiens]	19913406	DNA topoisomerase 2-alpha [Homo sapiens]	19913406	DNA topoisomerase 2-alpha [Homo sapiens]
26667203	calcium/calmodulin-dependent protein kinase type II subunit gamma isoform 1 [Homo sapiens]	26667203	calcium/calmodulin-dependent protein kinase type II subunit gamma isoform 1 [Homo sapiens]	20070220	protein arginine N-methyltransferase 5 isoform a [Homo sapiens]	20070220	protein arginine N-methyltransferase 5 isoform a [Homo sapiens]
29570791	casein kinase II subunit alpha isoform a [Homo sapiens]	29570791	casein kinase II subunit alpha isoform a [Homo sapiens]	21071032	probable ATP-dependent RNA helicase DDX41 [Homo sapiens]	21071032	probable ATP-dependent RNA helicase DDX41 [Homo sapiens]
29725607	protein unc-45 homolog A isoform 2 [Homo sapiens]	29725607	protein unc-45 homolog A isoform 2 [Homo sapiens]	21264315	EH domain-containing protein 4 [Homo sapiens]	21264315	EH domain-containing protein 4 [Homo sapiens]
29826321	alpha-adducin isoform b [Homo sapiens]	29826321	alpha-adducin isoform b [Homo sapiens]	21328448	14-3-3 protein beta/alpha [Homo sapiens]	21328448	14-3-3 protein beta/alpha [Homo sapiens]
29837655	polymerase delta-interacting protein 3 isoform 1 [Homo sapiens]	29837655	polymerase delta-interacting protein 3 isoform 1 [Homo sapiens]	21361091	ubiquitin carboxyl-terminal hydrolase isozyme L1 [Homo sapiens]	21361091	ubiquitin carboxyl-terminal hydrolase isozyme L1 [Homo sapiens]
30089962	serine/threonine-protein kinase MRCK alpha isoform B [Homo sapiens]	30089962	serine/threonine-protein kinase MRCK alpha isoform B [Homo sapiens]	21361282	serine/arginine-rich splicing factor 4 [Homo sapiens]	21361282	serine/arginine-rich splicing factor 4 [Homo sapiens]
30348954	E3 ubiquitin-protein ligase MIB1 [Homo sapiens]	30348954	E3 ubiquitin-protein ligase MIB1 [Homo sapiens]	21361659	importin-9 [Homo sapiens]	21361659	importin-9 [Homo sapiens]
31541941	heat shock 70 kDa protein 4L [Homo sapiens]	31541941	heat shock 70 kDa protein 4L [Homo sapiens]	21464101	14-3-3 protein gamma [Homo sapiens]	21464101	14-3-3 protein gamma [Homo sapiens]
32967311	ephrin type-A receptor 2 precursor [Homo sapiens]	32967311	ephrin type-A receptor 2 precursor [Homo sapiens]	21618340	signal transducer and activator of transcription 3 isoform 1 [Homo sapiens]	21618340	signal transducer and activator of transcription 3 isoform 1 [Homo sapiens]
33188445	microtubule-actin cross-linking factor 1 [Homo sapiens]	33188445	microtubule-actin cross-linking factor 1 [Homo sapiens]	21624607	coactosin-like protein [Homo sapiens]	21624607	coactosin-like protein [Homo sapiens]
33356552	G patch domain-containing protein 4 isoform 1 [Homo sapiens]	33356552	G patch domain-containing protein 4 isoform 1 [Homo sapiens]	22027646	angiomotin-like protein 1 [Homo sapiens]	22027646	angiomotin-like protein 1 [Homo sapiens]
34328899	tyrosine-protein phosphatase non-receptor type 14 [Homo sapiens]	34328899	tyrosine-protein phosphatase non-receptor type 14 [Homo sapiens]	22094987	regulatory-associated protein of mTOR isoform 1 [Homo sapiens]	22094987	regulatory-associated protein of mTOR isoform 1 [Homo sapiens]
35493811	RNA-binding protein 39 isoform a [Homo sapiens]	35493811	RNA-binding protein 39 isoform a [Homo sapiens]	22325356	PALM2-AKAP2 protein isoform 2 [Homo sapiens]	22325356	PALM2-AKAP2 protein isoform 2 [Homo sapiens]

38026934	rho guanine nucleotide exchange factor 11 isoform 2 [Homo sapiens]	38026934	rho guanine nucleotide exchange factor 11 isoform 2 [Homo sapiens]	23110942	proteasome subunit alpha type-5 isoform 1 [Homo sapiens]	23110942	proteasome subunit alpha type-5 isoform 1 [Homo sapiens]
38045910	laminin subunit alpha-3 isoform 1 precursor [Homo sapiens]	38045910	laminin subunit alpha-3 isoform 1 precursor [Homo sapiens]	23111028	sorting nexin-11 [Homo sapiens]	23111028	sorting nexin-11 [Homo sapiens]
38045919	huntingtin-interacting protein 1 isoform 1 [Homo sapiens]	38045919	huntingtin-interacting protein 1 isoform 1 [Homo sapiens]	23503295	casein kinase II subunit beta [Homo sapiens]	23503295	casein kinase II subunit beta [Homo sapiens]
38176300	nestin [Homo sapiens]	38176300	nestin [Homo sapiens]	23503301	protein FAM71C [Homo sapiens]	23503301	protein FAM71C [Homo sapiens]
38327039	heat shock 70 kDa protein 4 [Homo sapiens]	38327039	heat shock 70 kDa protein 4 [Homo sapiens]	23510340	ubiquitin-like modifier-activating enzyme 1 [Homo sapiens]	23510340	ubiquitin-like modifier-activating enzyme 1 [Homo sapiens]
39930523	uncharacterized protein C11orf84 [Homo sapiens]	39930523	uncharacterized protein C11orf84 [Homo sapiens]	23510448	DNA replication licensing factor MCM5 [Homo sapiens]	23510448	DNA replication licensing factor MCM5 [Homo sapiens]
39995082	tRNA (cytosine(34)-C(5))-methyltransferase isoform 1 [Homo sapiens]	39995082	tRNA (cytosine(34)-C(5))-methyltransferase isoform 1 [Homo sapiens]	25777600	26S proteasome non-ATPase regulatory subunit 1 isoform 1 [Homo sapiens]	25777600	26S proteasome non-ATPase regulatory subunit 1 isoform 1 [Homo sapiens]
40217825	SLIT and NTRK-like protein 6 precursor [Homo sapiens]	40217825	SLIT and NTRK-like protein 6 precursor [Homo sapiens]	25777602	26S proteasome non-ATPase regulatory subunit 2 [Homo sapiens]	25777602	26S proteasome non-ATPase regulatory subunit 2 [Homo sapiens]
40255047	pleckstrin homology domain-containing family H member 3 precursor [Homo sapiens]	40255047	pleckstrin homology domain-containing family H member 3 precursor [Homo sapiens]	26667180	calcium/calmodulin-dependent protein kinase type II subunit delta isoform 3 [Homo sapiens]	26667180	calcium/calmodulin-dependent protein kinase type II subunit delta isoform 3 [Homo sapiens]
40353727	synaptopodin isoform A [Homo sapiens]	40353727	synaptopodin isoform A [Homo sapiens]	29570791	casein kinase II subunit alpha isoform a [Homo sapiens]	29570791	casein kinase II subunit alpha isoform a [Homo sapiens]
41322912	plectin isoform 1f [Homo sapiens]	41322912	plectin isoform 1f [Homo sapiens]	29725607	protein unc-45 homolog A isoform 2 [Homo sapiens]	29725607	protein unc-45 homolog A isoform 2 [Homo sapiens]
41322916	plectin isoform 1 [Homo sapiens]	41322916	plectin isoform 1 [Homo sapiens]	29826321	alpha-adducin isoform b [Homo sapiens]	29826321	alpha-adducin isoform b [Homo sapiens]
42544159	heat shock protein 105 kDa [Homo sapiens]	42544159	heat shock protein 105 kDa [Homo sapiens]	30089962	serine/threonine-protein kinase MRCK alpha isoform B [Homo sapiens]	30089962	serine/threonine-protein kinase MRCK alpha isoform B [Homo sapiens]
42544193	galectin-8 isoform a [Homo sapiens]	42544193	galectin-8 isoform a [Homo sapiens]	31881740	solute carrier family 12 member 9 isoform 1 [Homo sapiens]	31881740	solute carrier family 12 member 9 isoform 1 [Homo sapiens]
42716280	vigilin isoform a [Homo sapiens]	42716280	vigilin isoform a [Homo sapiens]	32484979	AP-3 complex subunit beta-1 isoform 1 [Homo sapiens]	32484979	AP-3 complex subunit beta-1 isoform 1 [Homo sapiens]

42822874	ribonuclease inhibitor [Homo sapiens]	42822874	ribonuclease inhibitor [Homo sapiens]	32528282	cytosolic acyl coenzyme A thioester hydrolase isoform hBACHb [Homo sapiens]	32528282	cytosolic acyl coenzyme A thioester hydrolase isoform hBACHb [Homo sapiens]
44680105	caldesmon isoform 1 [Homo sapiens]	44680105	caldesmon isoform 1 [Homo sapiens]	32967311	ephrin type-A receptor 2 precursor [Homo sapiens]	32967311	ephrin type-A receptor 2 precursor [Homo sapiens]
45439306	aspartate--tRNA ligase, cytoplasmic [Homo sapiens]	45439306	aspartate--tRNA ligase, cytoplasmic [Homo sapiens]	33188445	microtubule-actin cross-linking factor 1 [Homo sapiens]	33188445	microtubule-actin cross-linking factor 1 [Homo sapiens]
45439327	periplakin [Homo sapiens]	45439327	periplakin [Homo sapiens]	34328899	tyrosine-protein phosphatase non-receptor type 14 [Homo sapiens]	34328899	tyrosine-protein phosphatase non-receptor type 14 [Homo sapiens]
45827706	protein quaking isoform HQK-5 [Homo sapiens]	45827706	protein quaking isoform HQK-5 [Homo sapiens]	35493811	RNA-binding protein 39 isoform a [Homo sapiens]	35493811	RNA-binding protein 39 isoform a [Homo sapiens]
46249397	ras-related GTP-binding protein B long isoform [Homo sapiens]	46249397	ras-related GTP-binding protein B long isoform [Homo sapiens]	38045919	huntingtin-interacting protein 1 isoform 1 [Homo sapiens]	38045919	huntingtin-interacting protein 1 isoform 1 [Homo sapiens]
47132587	protein kinase C delta-binding protein [Homo sapiens]	47132587	protein kinase C delta-binding protein [Homo sapiens]	38176300	nestin [Homo sapiens]	38176300	nestin [Homo sapiens]
49472822	eukaryotic translation initiation factor 3 subunit G [Homo sapiens]	49472822	eukaryotic translation initiation factor 3 subunit G [Homo sapiens]	38327039	heat shock 70 kDa protein 4 [Homo sapiens]	38327039	heat shock 70 kDa protein 4 [Homo sapiens]
49472841	A-kinase anchor protein 8-like [Homo sapiens]	49472841	A-kinase anchor protein 8-like [Homo sapiens]	38569421	ATP-citrate synthase isoform 1 [Homo sapiens]	38569421	ATP-citrate synthase isoform 1 [Homo sapiens]
50658065	structural maintenance of chromosomes protein 4 [Homo sapiens]	50658065	structural maintenance of chromosomes protein 4 [Homo sapiens]	39930523	uncharacterized protein C11orf84 [Homo sapiens]	39930523	uncharacterized protein C11orf84 [Homo sapiens]
51093861	caspase recruitment domain-containing protein 10 [Homo sapiens]	51093861	caspase recruitment domain-containing protein 10 [Homo sapiens]	40018629	WASH complex subunit 7 [Homo sapiens]	40018629	WASH complex subunit 7 [Homo sapiens]
51173717	actin-binding LIM protein 1 isoform s [Homo sapiens]	51173717	actin-binding LIM protein 1 isoform s [Homo sapiens]	40217825	SLIT and NTRK-like protein 6 precursor [Homo sapiens]	40217825	SLIT and NTRK-like protein 6 precursor [Homo sapiens]
53759151	acyl-CoA desaturase [Homo sapiens]	53759151	acyl-CoA desaturase [Homo sapiens]	40353727	synaptopodin isoform A [Homo sapiens]	40353727	synaptopodin isoform A [Homo sapiens]
53829374	plakophilin-4 isoform a [Homo sapiens]	53829374	plakophilin-4 isoform a [Homo sapiens]	41322912	plectin isoform 1f [Homo sapiens]	41322912	plectin isoform 1f [Homo sapiens]
55749621	pleckstrin homology domain-containing family G member 3 [Homo sapiens]	55749621	pleckstrin homology domain-containing family G member 3 [Homo sapiens]	41322916	plectin isoform 1 [Homo sapiens]	41322916	plectin isoform 1 [Homo sapiens]
55953087	nucleolar GTP-binding protein 1 [Homo sapiens]	55953087	nucleolar GTP-binding protein 1 [Homo sapiens]	42544159	heat shock protein 105 kDa [Homo sapiens]	42544159	heat shock protein 105 kDa [Homo sapiens]

56549121	dynamin-2 isoform 1 [Homo sapiens]	56549121	dynamin-2 isoform 1 [Homo sapiens]	42822874	ribonuclease inhibitor [Homo sapiens]	42822874	ribonuclease inhibitor [Homo sapiens]
57013276	tubulin alpha-1B chain [Homo sapiens]	57013276	tubulin alpha-1B chain [Homo sapiens]	45439306	aspartate--tRNA ligase, cytoplasmic [Homo sapiens]	45439306	aspartate--tRNA ligase, cytoplasmic [Homo sapiens]
58219792	ras-related protein Rap-1b isoform 1 precursor [Homo sapiens]	58219792	ras-related protein Rap-1b isoform 1 precursor [Homo sapiens]	45439327	periplakin [Homo sapiens]	45439327	periplakin [Homo sapiens]
58331242	FH1/FH2 domain-containing protein 3 [Homo sapiens]	58331242	FH1/FH2 domain-containing protein 3 [Homo sapiens]	46249397	ras-related GTP-binding protein B long isoform [Homo sapiens]	46249397	ras-related GTP-binding protein B long isoform [Homo sapiens]
59850762	uveal autoantigen with coiled-coil domains and ankyrin repeats isoform 1 [Homo sapiens]	59850762	uveal autoantigen with coiled-coil domains and ankyrin repeats isoform 1 [Homo sapiens]	46275835	protein FAM45A [Homo sapiens]	46275835	protein FAM45A [Homo sapiens]
61676188	E3 ubiquitin-protein ligase HUWE1 [Homo sapiens]	61676188	E3 ubiquitin-protein ligase HUWE1 [Homo sapiens]	47132587	protein kinase C delta-binding protein [Homo sapiens]	47132587	protein kinase C delta-binding protein [Homo sapiens]
61835148	fragile X mental retardation syndrome-related protein 1 isoform a [Homo sapiens]	61835148	fragile X mental retardation syndrome-related protein 1 isoform a [Homo sapiens]	48255929	periphilin-1 isoform 1 [Homo sapiens]	48255929	periphilin-1 isoform 1 [Homo sapiens]
63176611	SAFB-like transcription modulator isoform a [Homo sapiens]	63176611	SAFB-like transcription modulator isoform a [Homo sapiens]	50658065	structural maintenance of chromosomes protein 4 [Homo sapiens]	50658065	structural maintenance of chromosomes protein 4 [Homo sapiens]
66933016	inosine-5'-monophosphate dehydrogenase 2 [Homo sapiens]	66933016	inosine-5'-monophosphate dehydrogenase 2 [Homo sapiens]	51173717	actin-binding LIM protein 1 isoform s [Homo sapiens]	51173717	actin-binding LIM protein 1 isoform s [Homo sapiens]
67944630	60S ribosomal protein L9 [Homo sapiens]	67944630	60S ribosomal protein L9 [Homo sapiens]	53759151	acyl-CoA desaturase [Homo sapiens]	53759151	acyl-CoA desaturase [Homo sapiens]
68303572	casein kinase I isoform alpha isoform 2 [Homo sapiens]	68303572	casein kinase I isoform alpha isoform 2 [Homo sapiens]	53829365	BTB/POZ domain-containing protein KCTD2 [Homo sapiens]	53829365	BTB/POZ domain-containing protein KCTD2 [Homo sapiens]
70906430	AP-2 complex subunit sigma isoform AP17 [Homo sapiens]	70906430	AP-2 complex subunit sigma isoform AP17 [Homo sapiens]	53829374	plakophilin-4 isoform a [Homo sapiens]	53829374	plakophilin-4 isoform a [Homo sapiens]
71143119	signal-induced proliferation-associated 1-like protein 3 [Homo sapiens]	71143119	signal-induced proliferation-associated 1-like protein 3 [Homo sapiens]	55749621	pleckstrin homology domain-containing family G member 3 [Homo sapiens]	55749621	pleckstrin homology domain-containing family G member 3 [Homo sapiens]
71361682	nuclear mitotic apparatus protein 1 [Homo sapiens]	71361682	nuclear mitotic apparatus protein 1 [Homo sapiens]	56549121	dynamin-2 isoform 1 [Homo sapiens]	56549121	dynamin-2 isoform 1 [Homo sapiens]
73695475	HEAT repeat-containing protein 1 [Homo sapiens]	73695475	HEAT repeat-containing protein 1 [Homo sapiens]	56549640	septin-2 [Homo sapiens]	56549640	septin-2 [Homo sapiens]

74272284	membrane-associated guanylate kinase, WW and PDZ domain-containing protein 1 isoform c [Homo sapiens]	74272284	membrane-associated guanylate kinase, WW and PDZ domain-containing protein 1 isoform c [Homo sapiens]	57013276	tubulin alpha-1B chain [Homo sapiens]	57013276	tubulin alpha-1B chain [Homo sapiens]
83367072	eukaryotic translation initiation factor 3 subunit B [Homo sapiens]	83367072	eukaryotic translation initiation factor 3 subunit B [Homo sapiens]	58331242	FH1/FH2 domain-containing protein 3 [Homo sapiens]	58331242	FH1/FH2 domain-containing protein 3 [Homo sapiens]
83776600	aurora kinase B isoform 1 [Homo sapiens]	83776600	aurora kinase B isoform 1 [Homo sapiens]	59850762	uveal autoantigen with coiled-coil domains and ankyrin repeats isoform 1 [Homo sapiens]	59850762	uveal autoantigen with coiled-coil domains and ankyrin repeats isoform 1 [Homo sapiens]
91208426	pre-mRNA-processing-splicing factor 8 [Homo sapiens]	91208426	pre-mRNA-processing-splicing factor 8 [Homo sapiens]	61835148	fragile X mental retardation syndrome-related protein 1 isoform a [Homo sapiens]	61835148	fragile X mental retardation syndrome-related protein 1 isoform a [Homo sapiens]
92110027	glutaminyl-peptide cyclotransferase-like protein isoform 1 [Homo sapiens]	92110027	glutaminyl-peptide cyclotransferase-like protein isoform 1 [Homo sapiens]	62420875	integrin-linked protein kinase isoform 1 [Homo sapiens]	62420875	integrin-linked protein kinase isoform 1 [Homo sapiens]
94557305	39S ribosomal protein L19, mitochondrial [Homo sapiens]	94557305	39S ribosomal protein L19, mitochondrial [Homo sapiens]	66933016	inosine-5'-monophosphate dehydrogenase 2 [Homo sapiens]	66933016	inosine-5'-monophosphate dehydrogenase 2 [Homo sapiens]
94721241	isoleucine--tRNA ligase, cytoplasmic [Homo sapiens]	94721241	isoleucine--tRNA ligase, cytoplasmic [Homo sapiens]	67782356	corneodesmosin precursor [Homo sapiens]	67782356	corneodesmosin precursor [Homo sapiens]
94721261	2',3'-cyclic-nucleotide 3'-phosphodiesterase [Homo sapiens]	94721261	2',3'-cyclic-nucleotide 3'-phosphodiesterase [Homo sapiens]	67944630	60S ribosomal protein L9 [Homo sapiens]	67944630	60S ribosomal protein L9 [Homo sapiens]
1.09E+08	centrosomal protein of 170 kDa isoform alpha [Homo sapiens]	1.09E+08	centrosomal protein of 170 kDa isoform alpha [Homo sapiens]	68303572	casein kinase I isoform alpha isoform 2 [Homo sapiens]	68303572	casein kinase I isoform alpha isoform 2 [Homo sapiens]
1.1E+08	WD40 repeat-containing protein SMU1 [Homo sapiens]	1.1E+08	WD40 repeat-containing protein SMU1 [Homo sapiens]	68989256	ras and Rab interactor 1 [Homo sapiens]	68989256	ras and Rab interactor 1 [Homo sapiens]
1.11E+08	collagen alpha-1(XVIII) chain isoform 1 precursor [Homo sapiens]	1.11E+08	collagen alpha-1(XVIII) chain isoform 1 precursor [Homo sapiens]	70906430	AP-2 complex subunit sigma isoform AP17 [Homo sapiens]	70906430	AP-2 complex subunit sigma isoform AP17 [Homo sapiens]
1.12E+08	spindlin-1 [Homo sapiens]	1.12E+08	spindlin-1 [Homo sapiens]	71361682	nuclear mitotic apparatus protein 1 [Homo sapiens]	71361682	nuclear mitotic apparatus protein 1 [Homo sapiens]
1.12E+08	SLIT-ROBO Rho GTPase-activating protein 2 isoform a [Homo sapiens]	1.12E+08	SLIT-ROBO Rho GTPase-activating protein 2 isoform a [Homo sapiens]	77404397	staphylococcal nuclease domain-containing protein 1 [Homo sapiens]	77404397	staphylococcal nuclease domain-containing protein 1 [Homo sapiens]
1.12E+08	synemin isoform A [Homo sapiens]	1.12E+08	synemin isoform A [Homo sapiens]	81295407	acyl-coenzyme A thioesterase 9, mitochondrial isoform a precursor [Homo sapiens]	81295407	acyl-coenzyme A thioesterase 9, mitochondrial isoform a precursor [Homo sapiens]
1.13E+08	casein kinase I isoform gamma-3 isoform 4 [Homo sapiens]	1.13E+08	casein kinase I isoform gamma-3 isoform 4 [Homo sapiens]	82775371	coiled-coil domain-containing protein 93 [Homo sapiens]	82775371	coiled-coil domain-containing protein 93 [Homo sapiens]

1.17E+08	cytoplasmic FMR1-interacting protein 2 [Homo sapiens]	1.17E+08	cytoplasmic FMR1-interacting protein 2 [Homo sapiens]	83367072	eukaryotic translation initiation factor 3 subunit B [Homo sapiens]	83367072	eukaryotic translation initiation factor 3 subunit B [Homo sapiens]
1.17E+08	heterogeneous nuclear ribonucleoproteins C1/C2 isoform a [Homo sapiens]	1.17E+08	heterogeneous nuclear ribonucleoproteins C1/C2 isoform a [Homo sapiens]	83776600	aurora kinase B isoform 1 [Homo sapiens]	83776600	aurora kinase B isoform 1 [Homo sapiens]
1.19E+08	formin-like protein 3 isoform 1 [Homo sapiens]	1.19E+08	formin-like protein 3 isoform 1 [Homo sapiens]	94557305	39S ribosomal protein L19, mitochondrial [Homo sapiens]	94557305	39S ribosomal protein L19, mitochondrial [Homo sapiens]
1.19E+08	protein diaphanous homolog 1 isoform 1 [Homo sapiens]	1.19E+08	protein diaphanous homolog 1 isoform 1 [Homo sapiens]	94721241	isoleucine--tRNA ligase, cytoplasmic [Homo sapiens]	94721241	isoleucine--tRNA ligase, cytoplasmic [Homo sapiens]
1.23E+08	USP6 N-terminal-like protein isoform 2 [Homo sapiens]	1.23E+08	USP6 N-terminal-like protein isoform 2 [Homo sapiens]	1.11E+08	182 kDa tankyrase-1-binding protein [Homo sapiens]	1.11E+08	182 kDa tankyrase-1-binding protein [Homo sapiens]
1.24E+08	protein transport protein Sec16A isoform 1 [Homo sapiens]	1.24E+08	protein transport protein Sec16A isoform 1 [Homo sapiens]	1.11E+08	collagen alpha-1(XVIII) chain isoform 1 precursor [Homo sapiens]	1.11E+08	collagen alpha-1(XVIII) chain isoform 1 precursor [Homo sapiens]
1.26E+08	keratin, type II cytoskeletal 80 isoform K80 [Homo sapiens]	1.26E+08	keratin, type II cytoskeletal 80 isoform K80 [Homo sapiens]	1.12E+08	spindlin-1 [Homo sapiens]	1.12E+08	spindlin-1 [Homo sapiens]
1.26E+08	E3 ubiquitin-protein ligase HERC2 [Homo sapiens]	1.26E+08	E3 ubiquitin-protein ligase HERC2 [Homo sapiens]	1.12E+08	synemin isoform A [Homo sapiens]	1.12E+08	synemin isoform A [Homo sapiens]
1.26E+08	abnormal spindle-like microcephaly-associated protein isoform 1 [Homo sapiens]	1.26E+08	abnormal spindle-like microcephaly-associated protein isoform 1 [Homo sapiens]	1.17E+08	heterogeneous nuclear ribonucleoproteins C1/C2 isoform a [Homo sapiens]	1.17E+08	heterogeneous nuclear ribonucleoproteins C1/C2 isoform a [Homo sapiens]
1.34E+08	pericentriolar material 1 protein [Homo sapiens]	1.34E+08	pericentriolar material 1 protein [Homo sapiens]	1.19E+08	lysosomal protective protein isoform a precursor [Homo sapiens]	1.19E+08	lysosomal protective protein isoform a precursor [Homo sapiens]
1.34E+08	melanotransferrin isoform 1 precursor [Homo sapiens]	1.34E+08	melanotransferrin isoform 1 precursor [Homo sapiens]	1.19E+08	protein diaphanous homolog 1 isoform 1 [Homo sapiens]	1.19E+08	protein diaphanous homolog 1 isoform 1 [Homo sapiens]
1.49E+08	coatomer subunit alpha isoform 1 [Homo sapiens]	1.49E+08	coatomer subunit alpha isoform 1 [Homo sapiens]	1.21E+08	WASH complex subunit strumpellin [Homo sapiens]	1.21E+08	WASH complex subunit strumpellin [Homo sapiens]
1.49E+08	nexilin isoform 1 [Homo sapiens]	1.49E+08	nexilin isoform 1 [Homo sapiens]	1.23E+08	gap junction gamma-1 protein [Homo sapiens]	1.23E+08	gap junction gamma-1 protein [Homo sapiens]
1.54E+08	unconventional myosin-Vc [Homo sapiens]	1.54E+08	unconventional myosin-Vc [Homo sapiens]	1.26E+08	keratin, type II cytoskeletal 80 isoform K80 [Homo sapiens]	1.26E+08	keratin, type II cytoskeletal 80 isoform K80 [Homo sapiens]
1.54E+08	microtubule-associated protein 1B [Homo sapiens]	1.54E+08	microtubule-associated protein 1B [Homo sapiens]	1.26E+08	abnormal spindle-like microcephaly-associated protein isoform 1 [Homo sapiens]	1.26E+08	abnormal spindle-like microcephaly-associated protein isoform 1 [Homo sapiens]

1.54E+08	tenascin precursor [Homo sapiens]	1.54E+08	tenascin precursor [Homo sapiens]	1.34E+08	arginine and glutamate-rich protein 1 [Homo sapiens]	1.34E+08	arginine and glutamate-rich protein 1 [Homo sapiens]
1.54E+08	unconventional myosin-X [Homo sapiens]	1.54E+08	unconventional myosin-X [Homo sapiens]	1.49E+08	coatomer subunit alpha isoform 1 [Homo sapiens]	1.49E+08	coatomer subunit alpha isoform 1 [Homo sapiens]
1.55E+08	protein arginine N-methyltransferase 1 isoform 1 [Homo sapiens]	1.55E+08	protein arginine N-methyltransferase 1 isoform 1 [Homo sapiens]	1.49E+08	interferon-induced transmembrane protein 3 [Homo sapiens]	1.49E+08	interferon-induced transmembrane protein 3 [Homo sapiens]
1.56E+08	probable U3 small nucleolar RNA-associated protein 11 [Homo sapiens]	1.56E+08	probable U3 small nucleolar RNA-associated protein 11 [Homo sapiens]	1.49E+08	nexilin isoform 1 [Homo sapiens]	1.49E+08	nexilin isoform 1 [Homo sapiens]
1.57E+08	poly [ADP-ribose] polymerase 1 [Homo sapiens]	1.57E+08	poly [ADP-ribose] polymerase 1 [Homo sapiens]	1.5E+08	FK506-binding protein 15 [Homo sapiens]	1.5E+08	FK506-binding protein 15 [Homo sapiens]
1.57E+08	RNA 3'-terminal phosphate cyclase-like protein [Homo sapiens]	1.57E+08	RNA 3'-terminal phosphate cyclase-like protein [Homo sapiens]	1.53E+08	mitochondrial fission regulator 1-like isoform a [Homo sapiens]	1.53E+08	mitochondrial fission regulator 1-like isoform a [Homo sapiens]
1.57E+08	dedicator of cytokinesis protein 6 [Homo sapiens]	1.57E+08	dedicator of cytokinesis protein 6 [Homo sapiens]	1.54E+08	eukaryotic translation initiation factor 3 subunit C [Homo sapiens]	1.54E+08	eukaryotic translation initiation factor 3 subunit C [Homo sapiens]
1.58E+08	adenylyl cyclase-associated protein 1 [Homo sapiens]	1.58E+08	adenylyl cyclase-associated protein 1 [Homo sapiens]	1.54E+08	unconventional myosin-Vc [Homo sapiens]	1.54E+08	unconventional myosin-Vc [Homo sapiens]
1.58E+08	phosphatidylinositol 4-phosphate 3-kinase C2 domain-containing subunit alpha [Homo sapiens]	1.58E+08	phosphatidylinositol 4-phosphate 3-kinase C2 domain-containing subunit alpha [Homo sapiens]	1.54E+08	microtubule-associated protein 1B [Homo sapiens]	1.54E+08	microtubule-associated protein 1B [Homo sapiens]
1.58E+08	tensin-4 precursor [Homo sapiens]	1.58E+08	tensin-4 precursor [Homo sapiens]	1.54E+08	unconventional myosin-X [Homo sapiens]	1.54E+08	unconventional myosin-X [Homo sapiens]
1.58E+08	chromodomain-helicase-DNA-binding protein 3 isoform 3 [Homo sapiens]	1.58E+08	chromodomain-helicase-DNA-binding protein 3 isoform 3 [Homo sapiens]	1.55E+08	protein arginine N-methyltransferase 1 isoform 1 [Homo sapiens]	1.55E+08	protein arginine N-methyltransferase 1 isoform 1 [Homo sapiens]
1.66E+08	kin of IRRE-like protein 1 precursor [Homo sapiens]	1.66E+08	kin of IRRE-like protein 1 precursor [Homo sapiens]	1.57E+08	poly [ADP-ribose] polymerase 1 [Homo sapiens]	1.57E+08	poly [ADP-ribose] polymerase 1 [Homo sapiens]
1.67E+08	glypican-1 precursor [Homo sapiens]	1.67E+08	glypican-1 precursor [Homo sapiens]	1.57E+08	calpain-2 catalytic subunit isoform 1 [Homo sapiens]	1.57E+08	calpain-2 catalytic subunit isoform 1 [Homo sapiens]
1.68E+08	lactadherin isoform a preproprotein [Homo sapiens]	1.68E+08	lactadherin isoform a preproprotein [Homo sapiens]	1.57E+08	dedicator of cytokinesis protein 6 [Homo sapiens]	1.57E+08	dedicator of cytokinesis protein 6 [Homo sapiens]
1.88E+08	CD59 glycoprotein preproprotein [Homo sapiens]	1.88E+08	CD59 glycoprotein preproprotein [Homo sapiens]	1.58E+08	26S proteasome non-ATPase regulatory subunit 13 isoform 2 [Homo sapiens]	1.58E+08	26S proteasome non-ATPase regulatory subunit 13 isoform 2 [Homo sapiens]

1.89E+08	gelsolin isoform b [Homo sapiens]	1.89E+08	gelsolin isoform b [Homo sapiens]	1.58E+08	adenylyl cyclase-associated protein 1 [Homo sapiens]	1.58E+08	adenylyl cyclase-associated protein 1 [Homo sapiens]
1.93E+08	MAP/microtubule affinity-regulating kinase 3 isoform a [Homo sapiens]	1.93E+08	MAP/microtubule affinity-regulating kinase 3 isoform a [Homo sapiens]	1.58E+08	tensin-4 precursor [Homo sapiens]	1.58E+08	tensin-4 precursor [Homo sapiens]
1.94E+08	lysine--tRNA ligase isoform 1 [Homo sapiens]	1.94E+08	lysine--tRNA ligase isoform 1 [Homo sapiens]	1.58E+08	FYVE and coiled-coil domain-containing protein 1 [Homo sapiens]	1.58E+08	FYVE and coiled-coil domain-containing protein 1 [Homo sapiens]
1.96E+08	SUN domain-containing protein 1 isoform a [Homo sapiens]	1.96E+08	SUN domain-containing protein 1 isoform a [Homo sapiens]	1.66E+08	kin of IRRE-like protein 1 precursor [Homo sapiens]	1.66E+08	kin of IRRE-like protein 1 precursor [Homo sapiens]
1.97E+08	pleckstrin homology-like domain family B member 2 isoform a [Homo sapiens]	1.97E+08	pleckstrin homology-like domain family B member 2 isoform a [Homo sapiens]	1.67E+08	glypican-1 precursor [Homo sapiens]	1.67E+08	glypican-1 precursor [Homo sapiens]
1.97E+08	actin filament-associated protein 1 isoform A [Homo sapiens]	1.97E+08	actin filament-associated protein 1 isoform A [Homo sapiens]	1.68E+08	lactadherin isoform a preproprotein [Homo sapiens]	1.68E+08	lactadherin isoform a preproprotein [Homo sapiens]
2.03E+08	rho GTPase-activating protein 21 [Homo sapiens]	2.03E+08	rho GTPase-activating protein 21 [Homo sapiens]	1.69E+08	BTB/POZ domain-containing protein KCTD17 [Homo sapiens]	1.69E+08	BTB/POZ domain-containing protein KCTD17 [Homo sapiens]
2.08E+08	dynactin subunit 4 isoform a [Homo sapiens]	2.08E+08	dynactin subunit 4 isoform a [Homo sapiens]	1.72E+08	drebrin-like protein isoform c [Homo sapiens]	1.72E+08	drebrin-like protein isoform c [Homo sapiens]
2.09E+08	target of Myb protein 1 isoform 2 [Homo sapiens]	2.09E+08	target of Myb protein 1 isoform 2 [Homo sapiens]	1.88E+08	CD59 glycoprotein preproprotein [Homo sapiens]	1.88E+08	CD59 glycoprotein preproprotein [Homo sapiens]
2.1E+08	trifunctional purine biosynthetic protein adenosine-3 isoform 1 [Homo sapiens]	2.1E+08	trifunctional purine biosynthetic protein adenosine-3 isoform 1 [Homo sapiens]	1.88E+08	RNA-binding protein 28 isoform 1 [Homo sapiens]	1.88E+08	RNA-binding protein 28 isoform 1 [Homo sapiens]
2.14E+08	actin, aortic smooth muscle [Homo sapiens]	2.14E+08	actin, aortic smooth muscle [Homo sapiens]	1.89E+08	gelsolin isoform b [Homo sapiens]	1.89E+08	gelsolin isoform b [Homo sapiens]
2.17E+08	leucine zipper protein 1 [Homo sapiens]	2.17E+08	leucine zipper protein 1 [Homo sapiens]	1.89E+08	maspardin isoform a [Homo sapiens]	1.89E+08	maspardin isoform a [Homo sapiens]
2.21E+08	bullous pemphigoid antigen 1 isoform 2 [Homo sapiens]	2.21E+08	bullous pemphigoid antigen 1 isoform 2 [Homo sapiens]	1.94E+08	V-type proton ATPase 116 kDa subunit a isoform 1 isoform a [Homo sapiens]	1.94E+08	V-type proton ATPase 116 kDa subunit a isoform 1 isoform a [Homo sapiens]
2.23E+08	talin-1 [Homo sapiens]	2.23E+08	talin-1 [Homo sapiens]	1.94E+08	lysine--tRNA ligase isoform 1 [Homo sapiens]	1.94E+08	lysine--tRNA ligase isoform 1 [Homo sapiens]
2.24E+08	tropomyosin alpha-4 chain isoform 1 [Homo sapiens]	2.24E+08	tropomyosin alpha-4 chain isoform 1 [Homo sapiens]	1.96E+08	SUN domain-containing protein 1 isoform a [Homo sapiens]	1.96E+08	SUN domain-containing protein 1 isoform a [Homo sapiens]
2.24E+08	uncharacterized protein KIAA1671 [Homo sapiens]	2.24E+08	uncharacterized protein KIAA1671 [Homo sapiens]	1.97E+08	pleckstrin homology-like domain family B member 2 isoform a [Homo sapiens]	1.97E+08	pleckstrin homology-like domain family B member 2 isoform a [Homo sapiens]

2.38E+08	probable rRNA-processing protein EBP2 isoform 1 [Homo sapiens]	2.38E+08	probable rRNA-processing protein EBP2 isoform 1 [Homo sapiens]	1.97E+08	actin filament-associated protein 1 isoform A [Homo sapiens]	1.97E+08	actin filament-associated protein 1 isoform A [Homo sapiens]
2.39E+08	protein phosphatase Slingshot homolog 1 isoform 1 [Homo sapiens]	2.39E+08	protein phosphatase Slingshot homolog 1 isoform 1 [Homo sapiens]	2.08E+08	dynactin subunit 4 isoform a [Homo sapiens]	2.08E+08	dynactin subunit 4 isoform a [Homo sapiens]
2.42E+08	programmed cell death 6-interacting protein isoform 2 [Homo sapiens]	2.42E+08	programmed cell death 6-interacting protein isoform 2 [Homo sapiens]	2.1E+08	plastin-3 isoform 1 [Homo sapiens]	2.1E+08	plastin-3 isoform 1 [Homo sapiens]
2.54E+08	rho guanine nucleotide exchange factor 2 isoform 1 [Homo sapiens]	2.54E+08	rho guanine nucleotide exchange factor 2 isoform 1 [Homo sapiens]	2.1E+08	trifunctional purine biosynthetic protein adenosine-3 isoform 1 [Homo sapiens]	2.1E+08	trifunctional purine biosynthetic protein adenosine-3 isoform 1 [Homo sapiens]
2.54E+08	serine/threonine-protein kinase MARK2 isoform d [Homo sapiens]	2.54E+08	serine/threonine-protein kinase MARK2 isoform d [Homo sapiens]	2.14E+08	actin, aortic smooth muscle [Homo sapiens]	2.14E+08	actin, aortic smooth muscle [Homo sapiens]
2.55E+08	unconventional myosin-XIX isoform 2 [Homo sapiens]	2.55E+08	unconventional myosin-XIX isoform 2 [Homo sapiens]	2.17E+08	leucine zipper protein 1 [Homo sapiens]	2.17E+08	leucine zipper protein 1 [Homo sapiens]
2.56E+08	leucine-rich repeat and calponin homology domain-containing protein 1 isoform 1 [Homo sapiens]	2.56E+08	leucine-rich repeat and calponin homology domain-containing protein 1 isoform 1 [Homo sapiens]	2.2E+08	eukaryotic translation initiation factor 5A-1 isoform A [Homo sapiens]	2.2E+08	eukaryotic translation initiation factor 5A-1 isoform A [Homo sapiens]
2.97E+08	rho GDP-dissociation inhibitor 1 isoform a [Homo sapiens]	2.97E+08	rho GDP-dissociation inhibitor 1 isoform a [Homo sapiens]	2.21E+08	bullous pemphigoid antigen 1 isoform 2 [Homo sapiens]	2.21E+08	bullous pemphigoid antigen 1 isoform 2 [Homo sapiens]
3.07E+08	spectrin alpha chain, non-erythrocytic 1 isoform 3 [Homo sapiens]	3.07E+08	spectrin alpha chain, non-erythrocytic 1 isoform 3 [Homo sapiens]	2.22E+08	C-1-tetrahydrofolate synthase, cytoplasmic [Homo sapiens]	2.22E+08	C-1-tetrahydrofolate synthase, cytoplasmic [Homo sapiens]
3.07E+08	clathrin interactor 1 isoform 1 [Homo sapiens]	3.07E+08	clathrin interactor 1 isoform 1 [Homo sapiens]	2.23E+08	talin-1 [Homo sapiens]	2.23E+08	talin-1 [Homo sapiens]
3.08E+08	taperin [Homo sapiens]	3.08E+08	taperin [Homo sapiens]	2.24E+08	tropomyosin alpha-4 chain isoform 1 [Homo sapiens]	2.24E+08	tropomyosin alpha-4 chain isoform 1 [Homo sapiens]
3.12E+08	eukaryotic translation initiation factor 4 gamma 3 isoform 1 [Homo sapiens]	3.12E+08	eukaryotic translation initiation factor 4 gamma 3 isoform 1 [Homo sapiens]	2.24E+08	uncharacterized protein KIAA1671 [Homo sapiens]	2.24E+08	uncharacterized protein KIAA1671 [Homo sapiens]
3.24E+08	protein FAM101A [Homo sapiens]	3.24E+08	protein FAM101A [Homo sapiens]	2.25E+08	mitochondrial fission regulator 1 isoform 1 [Homo sapiens]	2.25E+08	mitochondrial fission regulator 1 isoform 1 [Homo sapiens]
3.26E+08	SWI/SNF-related matrix-associated actin-dependent regulator of chromatin subfamily A member 5 [Homo sapiens]	3.26E+08	SWI/SNF-related matrix-associated actin-dependent regulator of chromatin subfamily A member 5 [Homo sapiens]	2.39E+08	protein phosphatase Slingshot homolog 1 isoform 1 [Homo sapiens]	2.39E+08	protein phosphatase Slingshot homolog 1 isoform 1 [Homo sapiens]
3.32E+08	keratin, type II cytoskeletal 4 [Homo sapiens]	3.32E+08	keratin, type II cytoskeletal 4 [Homo sapiens]	2.42E+08	programmed cell death 6-interacting protein isoform 2 [Homo sapiens]	2.42E+08	programmed cell death 6-interacting protein isoform 2 [Homo sapiens]

3.33E+08	smoothelin isoform d [Homo sapiens]	3.33E+08	smoothelin isoform d [Homo sapiens]	2.54E+08	rho guanine nucleotide exchange factor 2 isoform 1 [Homo sapiens]	2.54E+08	rho guanine nucleotide exchange factor 2 isoform 1 [Homo sapiens]
3.39E+08	AP-2 complex subunit alpha-2 isoform 1 [Homo sapiens]	3.39E+08	AP-2 complex subunit alpha-2 isoform 1 [Homo sapiens]	2.55E+08	unconventional myosin-XIX isoform 2 [Homo sapiens]	2.55E+08	unconventional myosin-XIX isoform 2 [Homo sapiens]
3.43E+08	cytospin-B isoform 1 [Homo sapiens]	3.43E+08	cytospin-B isoform 1 [Homo sapiens]	2.56E+08	WD repeat-containing protein 81 isoform 1 [Homo sapiens]	2.56E+08	WD repeat-containing protein 81 isoform 1 [Homo sapiens]
3.55E+08	ras-related protein Rab-5C isoform b [Homo sapiens]	3.55E+08	ras-related protein Rab-5C isoform b [Homo sapiens]	2.6E+08	L-lactate dehydrogenase A chain isoform 3 [Homo sapiens]	2.6E+08	L-lactate dehydrogenase A chain isoform 3 [Homo sapiens]
3.55E+08	protein scribble homolog isoform a [Homo sapiens]	3.55E+08	protein scribble homolog isoform a [Homo sapiens]	2.82E+08	serine/threonine-protein phosphatase PGAM5, mitochondrial isoform 2 [Homo sapiens]	2.82E+08	serine/threonine-protein phosphatase PGAM5, mitochondrial isoform 2 [Homo sapiens]
3.59E+08	protein LAP2 isoform 8 [Homo sapiens]	3.59E+08	protein LAP2 isoform 8 [Homo sapiens]	2.9E+08	eukaryotic translation initiation factor 4 gamma 2 isoform 1 [Homo sapiens]	2.9E+08	eukaryotic translation initiation factor 4 gamma 2 isoform 1 [Homo sapiens]
3.67E+08	myosin-10 isoform 2 [Homo sapiens]	3.67E+08	myosin-10 isoform 2 [Homo sapiens]	2.97E+08	rho GDP-dissociation inhibitor 1 isoform a [Homo sapiens]	2.97E+08	rho GDP-dissociation inhibitor 1 isoform a [Homo sapiens]
3.74E+08	pleckstrin homology domain-containing family A member 5 isoform 4 [Homo sapiens]	3.74E+08	pleckstrin homology domain-containing family A member 5 isoform 4 [Homo sapiens]	3.05E+08	UPF0505 protein C16orf62 [Homo sapiens]	3.05E+08	UPF0505 protein C16orf62 [Homo sapiens]
3.88E+08	lamin-B2 [Homo sapiens]	3.88E+08	lamin-B2 [Homo sapiens]	3.07E+08	clathrin interactor 1 isoform 1 [Homo sapiens]	3.07E+08	clathrin interactor 1 isoform 1 [Homo sapiens]
3.9E+08	sentrin-specific protease 1 [Homo sapiens]	3.9E+08	sentrin-specific protease 1 [Homo sapiens]	3.08E+08	taperin [Homo sapiens]	3.08E+08	taperin [Homo sapiens]
3.95E+08	26S proteasome non-ATPase regulatory subunit 11 [Homo sapiens]	3.95E+08	26S proteasome non-ATPase regulatory subunit 11 [Homo sapiens]	3.24E+08	protein FAM101A [Homo sapiens]	3.24E+08	protein FAM101A [Homo sapiens]
4.29E+08	CUE domain-containing protein 1 [Homo sapiens]	4.29E+08	CUE domain-containing protein 1 [Homo sapiens]	3.32E+08	keratin, type II cytoskeletal 4 [Homo sapiens]	3.32E+08	keratin, type II cytoskeletal 4 [Homo sapiens]
4.32E+08	dedicator of cytokinesis protein 7 isoform 1 [Homo sapiens]	4.32E+08	dedicator of cytokinesis protein 7 isoform 1 [Homo sapiens]	3.43E+08	cytospin-B isoform 1 [Homo sapiens]	3.43E+08	cytospin-B isoform 1 [Homo sapiens]
4.76E+08	POTE ankyrin domain family member I [Homo sapiens]	4.76E+08	POTE ankyrin domain family member I [Homo sapiens]	3.55E+08	ras-related protein Rab-5C isoform b [Homo sapiens]	3.55E+08	ras-related protein Rab-5C isoform b [Homo sapiens]
				3.55E+08	protein scribble homolog isoform a [Homo sapiens]	3.55E+08	protein scribble homolog isoform a [Homo sapiens]
				3.59E+08	protein LAP2 isoform 8 [Homo sapiens]	3.59E+08	protein LAP2 isoform 8 [Homo sapiens]

				3.67E+08	myosin-10 isoform 2 [Homo sapiens]	3.67E+08	myosin-10 isoform 2 [Homo sapiens]
				3.84E+08	transketolase isoform 2 [Homo sapiens]	3.84E+08	transketolase isoform 2 [Homo sapiens]
				3.87E+08	radixin isoform 1 [Homo sapiens]	3.87E+08	radixin isoform 1 [Homo sapiens]
				3.88E+08	structural maintenance of chromosomes protein 2 [Homo sapiens]	3.88E+08	structural maintenance of chromosomes protein 2 [Homo sapiens]
				3.94E+08	actin-related protein 2/3 complex subunit 5 isoform 2 [Homo sapiens]	3.94E+08	actin-related protein 2/3 complex subunit 5 isoform 2 [Homo sapiens]
				3.95E+08	26S proteasome non-ATPase regulatory subunit 11 [Homo sapiens]	3.95E+08	26S proteasome non-ATPase regulatory subunit 11 [Homo sapiens]
				4.1E+08	PREDICTED: nucleoside diphosphate kinase B-like [Homo sapiens]	4.1E+08	PREDICTED: nucleoside diphosphate kinase B-like [Homo sapiens]
				4.29E+08	CUE domain-containing protein 1 [Homo sapiens]	4.29E+08	CUE domain-containing protein 1 [Homo sapiens]
				4.7E+08	transgelin-2 isoform a [Homo sapiens]	4.7E+08	transgelin-2 isoform a [Homo sapiens]

Table S6. Geneset enrichment of SMO binding proteins in ONS76 cells expressing SMO^{WT} or SMO^{W535L} with treatment of LDE225 or DMSO.

SMO^{WT}+DMSO							SMO^{WT}+LDE225					
Category	Term	Count	%	PValue	Fold Enrichment		Category	Term	Count	%	PValue	Fold Enrichment
GOTERM_BP_DIRECT	GO:0000387~spliceosomal snRNP assembly	3	11.11111111	0.000663821	74.96428571		GOTERM_BP_DIRECT	GO:0098609~cell-cell adhesion	6	16.21621622	2.00E-04	10.62224565
GOTERM_BP_DIRECT	GO:0035246~peptidyl-arginine N-methylation	2	7.407407407	0.005468041	349.8333333		GOTERM_BP_DIRECT	GO:0000387~spliceosomal snRNP assembly	3	8.108108108	0.001455359	51.40408163
GOTERM_BP_DIRECT	GO:0018216~peptidyl-arginine methylation	2	7.407407407	0.005468041	349.8333333		GOTERM_BP_DIRECT	GO:0016032~viral process	5	13.51351351	0.002999415	8.022933588

GOTERM_BP_DIRECT	GO:0098609~cell-cell adhesion	4	14.81481481	0.00579739	10.32718327		GOTERM_BP_DIRECT	GO:0018216~peptidyl-arginine methylation	2	5.405405405	0.008075249	239.8857143
GOTERM_BP_DIRECT	GO:0043985~histone H4-R3 methylation	2	7.407407407	0.008191325	233.2222222		GOTERM_BP_DIRECT	GO:0035246~peptidyl-arginine N-methylation	2	5.405405405	0.008075249	239.8857143
GOTERM_BP_DIRECT	GO:0001649~osteoblast differentiation	3	11.11111111	0.008830255	20.18269231		GOTERM_BP_DIRECT	GO:0000056~ribosomal small subunit export from nucleus	2	5.405405405	0.010084151	191.9085714
GOTERM_BP_DIRECT	GO:0032211~negative regulation of telomere maintenance via telomerase	2	7.407407407	0.016318446	116.6111111		GOTERM_BP_DIRECT	GO:0043985~histone H4-R3 methylation	2	5.405405405	0.012089103	159.9238095
GOTERM_BP_DIRECT	GO:0000028~ribosomal small subunit assembly	2	7.407407407	0.025719583	73.64912281		GOTERM_BP_DIRECT	GO:0019886~antigen processing and presentation of exogenous peptide antigen via MHC class II	3	8.108108108	0.014865378	15.6447205
GOTERM_BP_DIRECT	GO:0006412~translation	3	11.11111111	0.046477503	8.296442688		GOTERM_BP_DIRECT	GO:0006614~SRP-dependent cotranslational protein targeting to membrane	3	8.108108108	0.015483351	15.3118541
GOTERM_BP_DIRECT	GO:0030177~positive regulation of Wnt signaling pathway	2	7.407407407	0.048194457	38.87037037		GOTERM_BP_DIRECT	GO:0007097~nuclear migration	2	5.405405405	0.018080341	106.615873
							GOTERM_BP_DIRECT	GO:0019083~viral transcription	3	8.108108108	0.02152653	12.85102041
							GOTERM_BP_DIRECT	GO:0000184~nuclear-transcribed mRNA catabolic process, nonsense-mediated decay	3	8.108108108	0.024101749	12.09507803
							GOTERM_BP_DIRECT	GO:0006413~translational initiation	3	8.108108108	0.031267153	10.50594369
							GOTERM_BP_DIRECT	GO:0051301~cell division	4	10.81081081	0.033358876	5.483102041
							GOTERM_BP_DIRECT	GO:0035904~aorta development	2	5.405405405	0.03584323	53.30793651
							GOTERM_BP_DIRECT	GO:0000028~ribosomal small subunit assembly	2	5.405405405	0.037797524	50.50225564
							GOTERM_BP_DIRECT	GO:0006888~ER to Golgi vesicle-mediated transport	3	8.108108108	0.04148278	8.995714286
							GOTERM_BP_DIRECT	GO:0060976~coronary vasculature development	2	5.405405405	0.049442801	38.38171429
SMOW535L+DMSO							SMOWT+LDE225					

Category	Term	Count	%	PValue	Fold Enrichment		Category	Term	Count	%	PValue	Fold Enrichment
GOTERM_BP_DIRECT	GO:0098609~cell-cell adhesion	25	10.68376068	1.69E-13	6.977826535		GOTERM_BP_DIRECT	GO:0098609~cell-cell adhesion	38	15.32258065	7.35E-26	9.851873581
GOTERM_BP_DIRECT	GO:0006413~translational initiation	14	5.982905983	3.17E-08	7.729598211		GOTERM_BP_DIRECT	GO:0043161~proteasome-mediated ubiquitin-dependent protein catabolic process	16	6.451612903	0.000000208	5.537687821
GOTERM_BP_DIRECT	GO:0001731~formation of translation preinitiation complex	7	2.991452991	0.000000407	23.02075989		GOTERM_BP_DIRECT	GO:0031145~anaphase-promoting complex-dependent catabolic process	10	4.032258065	0.00000172	8.893596737
GOTERM_BP_DIRECT	GO:0007010~cytoskeleton organization	13	5.555555556	0.00000149	6.107548542		GOTERM_BP_DIRECT	GO:0030036~actin cytoskeleton organization	12	4.838709677	0.00000243	6.48548439
GOTERM_BP_DIRECT	GO:0006364~rRNA processing	14	5.982905983	0.00000533	4.94838764		GOTERM_BP_DIRECT	GO:0051436~negative regulation of ubiquitin-protein ligase activity involved in mitotic cell cycle	9	3.629032258	0.00000714	8.90612293
GOTERM_BP_DIRECT	GO:0006446~regulation of translational initiation	7	2.991452991	0.0000068	14.70770771		GOTERM_BP_DIRECT	GO:0051301~cell division	18	7.258064516	0.0000109	3.613341303
GOTERM_BP_DIRECT	GO:0000226~microtubule cytoskeleton organization	8	3.418803419	0.0000405	8.522776297		GOTERM_BP_DIRECT	GO:0034314~Arp2/3 complex-mediated actin nucleation	6	2.419354839	0.0000119	19.16165843
GOTERM_BP_DIRECT	GO:0030036~actin cytoskeleton organization	10	4.273504274	0.0000559	5.818433818		GOTERM_BP_DIRECT	GO:0051437~positive regulation of ubiquitin-protein ligase activity involved in regulation of mitotic cell cycle transition	9	3.629032258	0.0000119	8.32019379
GOTERM_BP_DIRECT	GO:0034314~Arp2/3 complex-mediated actin nucleation	5	2.136752137	0.000177	17.19082719		GOTERM_BP_DIRECT	GO:0016032~viral process	16	6.451612903	0.0000249	3.759701096
GOTERM_BP_DIRECT	GO:0019886~antigen processing and presentation of exogenous peptide antigen via MHC class II	8	3.418803419	0.000211	6.577359969		GOTERM_BP_DIRECT	GO:0060071~Wnt signaling pathway, planar cell polarity pathway	9	3.629032258	0.0000484	6.873203566
GOTERM_BP_DIRECT	GO:0051056~regulation of small GTPase mediated signal transduction	9	3.846153846	0.000399	5.080274304		GOTERM_BP_DIRECT	GO:0006521~regulation of cellular amino acid metabolic process	7	2.822580645	0.0000804	9.643449011
GOTERM_BP_DIRECT	GO:0001649~osteoblast differentiation	8	3.418803419	0.000448	5.818433818		GOTERM_BP_DIRECT	GO:0007010~cytoskeleton organization	11	4.435483871	0.000101	4.800332649
GOTERM_BP_DIRECT	GO:0008360~regulation of cell shape	9	3.846153846	0.000535	4.862548263		GOTERM_BP_DIRECT	GO:0043488~regulation of mRNA stability	9	3.629032258	0.000108	6.139172117
GOTERM_BP_DIRECT	GO:0048268~clathrin coat assembly	4	1.709401709	0.000583	23.27373527		GOTERM_BP_DIRECT	GO:0006413~translational initiation	10	4.032258065	0.000147	5.128424396
GOTERM_BP_DIRECT	GO:0016032~viral process	13	5.555555556	0.000626	3.288679984		GOTERM_BP_DIRECT	GO:0007067~mitotic nuclear division	13	5.241935484	0.000228	3.682953165

GOTERM_BP_DIRECT	GO:0021987~cerebral cortex development	6	2.564102564	0.000835	8.104247104		GOTERM_BP_DIRECT	GO:0002479~antigen processing and presentation of exogenous peptide antigen via MHC class I, TAP-dependent	7	2.822580645	0.000263	7.806601581
GOTERM_BP_DIRECT	GO:0045104~intermediate filament cytoskeleton organization	4	1.709401709	0.00091	20.17057057		GOTERM_BP_DIRECT	GO:0090263~positive regulation of canonical Wnt signaling pathway	9	3.629032258	0.000311	5.269456067
GOTERM_BP_DIRECT	GO:0006928~movement of cell or subcellular component	7	2.991452991	0.000951	6.156714854		GOTERM_BP_DIRECT	GO:0019886~antigen processing and presentation of exogenous peptide antigen via MHC class II	8	3.225806452	0.000331	6.109514281
GOTERM_BP_DIRECT	GO:0007051~spindle organization	4	1.709401709	0.00110961	18.90990991		GOTERM_BP_DIRECT	GO:0038061~NIK/NF-kappaB signaling	7	2.822580645	0.00034	7.451756054
GOTERM_BP_DIRECT	GO:0030705~cytoskeleton-dependent intracellular transport	4	1.709401709	0.001585782	16.80880881		GOTERM_BP_DIRECT	GO:0045104~intermediate filament cytoskeleton organization	4	1.612903226	0.001127774	18.73584379
GOTERM_BP_DIRECT	GO:0075525~viral translational termination-reinitiation	3	1.282051282	0.001679844	45.38378378		GOTERM_BP_DIRECT	GO:0021987~cerebral cortex development	6	2.419354839	0.001162949	7.527794381
GOTERM_BP_DIRECT	GO:0006418~tRNA aminoacylation for protein translation	5	2.136752137	0.001840702	9.454954955		GOTERM_BP_DIRECT	GO:0006606~protein import into nucleus	6	2.419354839	0.00136345	7.268215265
GOTERM_BP_DIRECT	GO:0006412~translation	11	4.700854701	0.001941856	3.288679984		GOTERM_BP_DIRECT	GO:0007051~spindle organization	4	1.612903226	0.001373575	17.56485356
GOTERM_BP_DIRECT	GO:0043547~positive regulation of GTPase activity	17	7.264957265	0.003387928	2.275882963		GOTERM_BP_DIRECT	GO:0048013~ephrin receptor signaling pathway	7	2.822580645	0.001391139	5.71878953
GOTERM_BP_DIRECT	GO:0075522~IRES-dependent viral translational initiation	3	1.282051282	0.003466813	32.41698842		GOTERM_BP_DIRECT	GO:0006928~movement of cell or subcellular component	7	2.822580645	0.001391139	5.71878953
GOTERM_BP_DIRECT	GO:0019083~viral transcription	7	2.991452991	0.003650706	4.727477477		GOTERM_BP_DIRECT	GO:0006893~Golgi to plasma membrane transport	4	1.612903226	0.001650555	16.53162688
GOTERM_BP_DIRECT	GO:0007018~microtubule-based movement	6	2.564102564	0.004299625	5.60293627		GOTERM_BP_DIRECT	GO:0007043~cell-cell junction assembly	4	1.612903226	0.001960069	15.61320316
GOTERM_BP_DIRECT	GO:0000184~nuclear-transcribed mRNA catabolic process, nonsense-mediated decay	7	2.991452991	0.004910192	4.449390567		GOTERM_BP_DIRECT	GO:0090090~negative regulation of canonical Wnt signaling pathway	9	3.629032258	0.002270892	3.87935416
GOTERM_BP_DIRECT	GO:0006888~ER to Golgi vesicle-mediated transport	8	3.418803419	0.005312433	3.781981982		GOTERM_BP_DIRECT	GO:0035338~long-chain fatty-acyl-CoA biosynthetic process	5	2.016129032	0.002886946	8.364215979
GOTERM_BP_DIRECT	GO:0048013~ephrin receptor signaling pathway	6	2.564102564	0.005544378	5.277184161		GOTERM_BP_DIRECT	GO:0000226~microtubule cytoskeleton organization	6	2.419354839	0.003339681	5.937415287
GOTERM_BP_DIRECT	GO:0000387~spliceosomal snRNP assembly	4	1.709401709	0.005779831	10.80566281		GOTERM_BP_DIRECT	GO:0001649~osteoblast differentiation	7	2.822580645	0.003638676	4.728999034

GOTERM_BP_DIRECT	GO:0036498~IRE1-mediated unfolded protein response	5	2.136752137	0.007551568	6.410138953		GOTERM_BP_DIRECT	GO:0002223~stimulatory C-type lectin receptor signaling pathway	7	2.822580645	0.003814514	4.683960948
GOTERM_BP_DIRECT	GO:0006614~SRP-dependent cotranslational protein targeting to membrane	6	2.564102564	0.008033775	4.828062105		GOTERM_BP_DIRECT	GO:0001731~formation of translation preinitiation complex	4	1.612903226	0.004037824	12.21902856
GOTERM_BP_DIRECT	GO:0000902~cell morphogenesis	5	2.136752137	0.008978287	6.099970939		GOTERM_BP_DIRECT	GO:0006457~protein folding	9	3.629032258	0.004158233	3.512970711
GOTERM_BP_DIRECT	GO:0016055~Wnt signaling pathway	8	3.418803419	0.012016478	3.235920412		GOTERM_BP_DIRECT	GO:1900034~regulation of cellular response to heat	6	2.419354839	0.004230021	5.620753138
GOTERM_BP_DIRECT	GO:0051693~actin filament capping	3	1.282051282	0.012224453	17.45530146		GOTERM_BP_DIRECT	GO:0008283~cell proliferation	13	5.241935484	0.006124553	2.495552964
GOTERM_BP_DIRECT	GO:0006886~intracellular protein transport	9	3.846153846	0.013079755	2.884562529		GOTERM_BP_DIRECT	GO:0033209~tumor necrosis factor-mediated signaling pathway	7	2.822580645	0.006714821	4.167931352
GOTERM_BP_DIRECT	GO:0007015~actin filament organization	5	2.136752137	0.014964251	5.252752753		GOTERM_BP_DIRECT	GO:0007097~nuclear migration	3	1.209677419	0.006744126	23.41980474
GOTERM_BP_DIRECT	GO:1900034~regulation of cellular response to heat	5	2.136752137	0.017152224	5.042642643		GOTERM_BP_DIRECT	GO:0000184~nuclear-transcribed mRNA catabolic process, nonsense-mediated decay	7	2.822580645	0.006989795	4.132906719
GOTERM_BP_DIRECT	GO:0016925~protein sumoylation	6	2.564102564	0.019280061	3.878955879		GOTERM_BP_DIRECT	GO:0006888~ER to Golgi vesicle-mediated transport	8	3.225806452	0.0077945	3.512970711
GOTERM_BP_DIRECT	GO:0090630~activation of GTPase activity	5	2.136752137	0.020365783	4.787318965		GOTERM_BP_DIRECT	GO:0045792~negative regulation of cell size	3	1.209677419	0.008351794	21.07782427
GOTERM_BP_DIRECT	GO:0007043~cell-cell junction assembly	3	1.282051282	0.022968096	12.60660661		GOTERM_BP_DIRECT	GO:0051639~actin filament network formation	3	1.209677419	0.008351794	21.07782427
GOTERM_BP_DIRECT	GO:0035904~aorta development	3	1.282051282	0.022968096	12.60660661		GOTERM_BP_DIRECT	GO:1900740~positive regulation of protein insertion into mitochondrial membrane involved in apoptotic signaling pathway	4	1.612903226	0.008607483	9.367921897
GOTERM_BP_DIRECT	GO:0051493~regulation of cytoskeleton organization	3	1.282051282	0.025450819	11.943101		GOTERM_BP_DIRECT	GO:0045773~positive regulation of axon extension	4	1.612903226	0.008607483	9.367921897
GOTERM_BP_DIRECT	GO:0016477~cell migration	7	2.991452991	0.026434582	3.078357427		GOTERM_BP_DIRECT	GO:0030100~regulation of endocytosis	4	1.612903226	0.009431518	9.065730868
GOTERM_BP_DIRECT	GO:0021762~substantia nigra development	4	1.709401709	0.026588795	6.17466446		GOTERM_BP_DIRECT	GO:0061024~membrane organization	4	1.612903226	0.010300032	8.782426778
GOTERM_BP_DIRECT	GO:0000398~mRNA splicing, via spliceosome	8	3.418803419	0.0278548	2.72575278		GOTERM_BP_DIRECT	GO:0016477~cell migration	8	3.225806452	0.011326397	3.267879731

GOTERM_BP_DIRECT	GO:0031124~mRNA 3'-end processing	4	1.709401709	0.028018646	6.051171171		GOTERM_BP_DIRECT	GO:0000902~cell morphogenesis	5	2.016129032	0.01155321	5.666081792
GOTERM_BP_DIRECT	GO:0007266~Rho protein signal transduction	4	1.709401709	0.028018646	6.051171171		GOTERM_BP_DIRECT	GO:0000165~MAPK cascade	10	4.032258065	0.012631406	2.681657032
GOTERM_BP_DIRECT	GO:0072583~clathrin-mediated endocytosis	3	1.282051282	0.028037254	11.34594595		GOTERM_BP_DIRECT	GO:0048268~clathrin coat assembly	3	1.209677419	0.01407741	16.21371098
GOTERM_BP_DIRECT	GO:0097150~neuronal stem cell population maintenance	3	1.282051282	0.030724268	10.80566281		GOTERM_BP_DIRECT	GO:0006446~regulation of translational initiation	4	1.612903226	0.014226823	7.806601581
GOTERM_BP_DIRECT	GO:0006405~RNA export from nucleus	4	1.709401709	0.035768246	5.501064701		GOTERM_BP_DIRECT	GO:0016337~single organismal cell-cell adhesion	6	2.419354839	0.014461836	4.173826588
GOTERM_BP_DIRECT	GO:0050770~regulation of axonogenesis	3	1.282051282	0.036387814	9.866039953		GOTERM_BP_DIRECT	GO:0008360~regulation of cell shape	7	2.822580645	0.01482289	3.512970711
GOTERM_BP_DIRECT	GO:0006897~endocytosis	6	2.564102564	0.036827728	3.265020416		GOTERM_BP_DIRECT	GO:0051016~barbed-end actin filament capping	3	1.209677419	0.016271691	15.05558876
GOTERM_BP_DIRECT	GO:0048870~cell motility	3	1.282051282	0.039358394	9.454954955		GOTERM_BP_DIRECT	GO:0006605~protein targeting	4	1.612903226	0.017655611	7.206093767
GOTERM_BP_DIRECT	GO:0030866~cortical actin cytoskeleton organization	3	1.282051282	0.039358394	9.454954955		GOTERM_BP_DIRECT	GO:1904874~positive regulation of telomerase RNA localization to Cajal body	3	1.209677419	0.018601525	14.05188285
GOTERM_BP_DIRECT	GO:0060976~coronary vasculature development	3	1.282051282	0.042417645	9.076756757		GOTERM_BP_DIRECT	GO:0006418~tRNA aminoacylation for protein translation	4	1.612903226	0.018891761	7.025941423
GOTERM_BP_DIRECT	GO:0030041~actin filament polymerization	3	1.282051282	0.045562742	8.727650728		GOTERM_BP_DIRECT	GO:0045184~establishment of protein localization	4	1.612903226	0.018891761	7.025941423
GOTERM_BP_DIRECT	GO:0007067~mitotic nuclear division	8	3.418803419	0.046641714	2.439988375		GOTERM_BP_DIRECT	GO:0050852~T cell receptor signaling pathway	7	2.822580645	0.018980671	3.323080403
GOTERM_BP_DIRECT	GO:0001764~neuron migration	5	2.136752137	0.049963326	3.601887602		GOTERM_BP_DIRECT	GO:0007015~actin filament organization	5	2.016129032	0.019108396	4.879125988
							GOTERM_BP_DIRECT	GO:0006886~intracellular protein transport	9	3.629032258	0.019298806	2.679384441
							GOTERM_BP_DIRECT	GO:0010592~positive regulation of lamellipodium assembly	3	1.209677419	0.021062661	13.17364017
							GOTERM_BP_DIRECT	GO:0016192~vesicle-mediated transport	7	2.822580645	0.021331689	3.235630918

							GOTERM_BP_DIRECT	GO:0016236~macroautophagy	5	2.016129032	0.022817514	4.622329883
							GOTERM_BP_DIRECT	GO:1901216~positive regulation of neuron death	3	1.209677419	0.023650941	12.39872016
							GOTERM_BP_DIRECT	GO:0030838~positive regulation of actin filament polymerization	4	1.612903226	0.0257754	6.245281265
							GOTERM_BP_DIRECT	GO:0030705~cytoskeleton-dependent intracellular transport	3	1.209677419	0.0263623	11.70990237
							GOTERM_BP_DIRECT	GO:0035904~aorta development	3	1.209677419	0.0263623	11.70990237
							GOTERM_BP_DIRECT	GO:0035264~multicellular organism growth	5	2.016129032	0.026936065	4.391213389
							GOTERM_BP_DIRECT	GO:0006412~translation	9	3.629032258	0.02764802	2.499346751
							GOTERM_BP_DIRECT	GO:0007018~microtubule-based movement	5	2.016129032	0.028030639	4.337000878
							GOTERM_BP_DIRECT	GO:0032956~regulation of actin cytoskeleton organization	4	1.612903226	0.028856697	5.979524615
							GOTERM_BP_DIRECT	GO:0071230~cellular response to amino acid stimulus	4	1.612903226	0.028856697	5.979524615
							GOTERM_BP_DIRECT	GO:0000266~mitochondrial fission	3	1.209677419	0.02919276	11.09359172
							GOTERM_BP_DIRECT	GO:0051493~regulation of cytoskeleton organization	3	1.209677419	0.02919276	11.09359172
							GOTERM_BP_DIRECT	GO:0051603~proteolysis involved in cellular protein catabolic process	4	1.612903226	0.030467285	5.854951185
							GOTERM_BP_DIRECT	GO:0031424~keratinization	4	1.612903226	0.030467285	5.854951185
							GOTERM_BP_DIRECT	GO:0030307~positive regulation of cell growth	5	2.016129032	0.031471795	4.18210799
							GOTERM_BP_DIRECT	GO:0072583~clathrin-mediated endocytosis	3	1.209677419	0.03213843	10.53891213

							GOTERM_BP_DIRECT	GO:0000132~establishment of mitotic spindle orientation	3	1.209677419	0.035195504	10.03705918
							GOTERM_BP_DIRECT	GO:0038095~Fc-epsilon receptor signaling pathway	7	2.822580645	0.040964101	2.763010672
							GOTERM_BP_DIRECT	GO:0032487~regulation of Rap protein signal transduction	2	0.806451613	0.041922899	46.83960948
							GOTERM_BP_DIRECT	GO:0090306~spindle assembly involved in meiosis	2	0.806451613	0.041922899	46.83960948
							GOTERM_BP_DIRECT	GO:0051383~kinetochore organization	2	0.806451613	0.041922899	46.83960948
							GOTERM_BP_DIRECT	GO:0006614~SRP-dependent cotranslational protein targeting to membrane	5	2.016129032	0.044665252	3.737202884
							GOTERM_BP_DIRECT	GO:0048870~cell motility	3	1.209677419	0.044998357	8.782426778
							GOTERM_BP_DIRECT	GO:0030866~cortical actin cytoskeleton organization	3	1.209677419	0.044998357	8.782426778
							GOTERM_BP_DIRECT	GO:0016049~cell growth	4	1.612903226	0.045006179	5.018529588
							GOTERM_BP_DIRECT	GO:0000086~G2/M transition of mitotic cell cycle	6	2.419354839	0.045594478	3.077054638
							GOTERM_BP_DIRECT	GO:0000209~protein polyubiquitination	7	2.822580645	0.046792751	2.672912498
							GOTERM_BP_DIRECT	GO:0006897~endocytosis	6	2.419354839	0.047996532	3.03278047
							GOTERM_BP_DIRECT	GO:0060976~coronary vasculature development	3	1.209677419	0.048464657	8.431129707
							GOTERM_BP_DIRECT	GO:0007569~cell aging	3	1.209677419	0.048464657	8.431129707
							GOTERM_BP_DIRECT	GO:0016055~Wnt signaling pathway	7	2.822580645	0.049890899	2.630031549
