# Supplementary material for: Targeting AKT and CK2 represents a novel therapeutic strategy for SMO constitutive activation‐driven medulloblastoma
Source: CNS Neurosci Ther. 2022 Apr 14;28(7):1033–44. doi: 10.1111/cns.13835 (PMC9160449; doi:10.1111/cns.13835)
Supplement: Supplementary file 2 — Supplementary Material [file CNS-28-1033-s001.pdf]

Dataset 1. FPKM value (>=1) of RNA-sequencing result using ONS76 cells stably expressing wild-type SMO or W535L mutant SMO with treatment of

Gene Symbol	W535L/LDE-1	W535L/LDE-2	W535L/DMSO-	W535L/DMSO-	WT/LDE-1	WT/LDE-2	WT/DMSO-1	WT/DMSO-2
CDKL3	1.19	1.32	1.61	1.36	1.27	1	1.62	1.66
CCT6B	2	1.65	1.71	1.68	1.5	1.01	1.65	1.12
C11orf45	1.33	1.08	1.52	1.27	1.34	1.01	1.32	1.26
C14orf132	1.22	1.32	1.23	1.09	1.15	1.02	1.28	1.12
ZNF836	1.01	1.14	1.11	1.17	1.16	1.02	1.45	1.31
CFHR3	1.28	1.39	1.76	1.54	1.41	1.02	2.36	1.55
ZNF470	1.07	1.06	1.25	1.54	1.02	1.04	1.86	1.64
DUSP10	1.27	1.09	1.4	1.84	1.61	1.04	1.37	1.66
ONECUT2	1.13	1.22	1.01	1.46	1.11	1.05	1.27	1.35
APOL6	1.19	1.28	1.39	1.54	1.13	1.05	1.53	1.35
METTL10	1.41	1.4	1.51	1.32	1.2	1.05	1.54	1.37
LOC107984974	1.44	1.33	1.36	1.22	1.5	1.07	1.48	1.23
ZNF813	1.09	1.11	1.11	1.29	1.27	1.07	2.05	1.75
LIN54	1.18	1	1.38	1.58	1.27	1.07	2.03	1.87
GALNT4	1.12	1.31	1.03	1.72	1.32	1.08	2.02	1.51
LOC107985246	1.6	1.65	1.75	1.45	1.09	1.09	1.35	1.6
KCTD6	2.38	1.99	2.12	1.83	1.09	1.09	1.35	1.71
DNAJC6	1.19	1.26	1.64	1.47	1.12	1.1	1.48	1.41
FHDC1	1.33	1.18	1.15	1.29	1.16	1.11	1.16	1.08
MTUS1	1.13	1.09	1.22	1.79	1.2	1.11	1.89	1.31
APOL4	1.29	1.15	1.24	1.29	1.29	1.11	1.79	1.74
PRSS27	1.65	1.45	1.48	1.49	1.1	1.12	1.48	1.16
ZIC1	1.27	1.2	1.25	1.74	1.21	1.12	1.45	1.17
PSTPIP1	1.4	1.32	1.03	1.04	1.23	1.12	1.35	1.25
ZNF484	1.2	1.17	1.54	1.59	1.23	1.12	1.82	1.38
PPP1R3E	1.33	1.06	1.37	1.21	1.31	1.12	1.78	1.42
FGF5	1.08	1.18	1.07	1.68	1.24	1.12	1.86	1.48
DDX60L	1.72	1.74	2.34	2.72	1.09	1.12	2.39	1.79
KCNE5	1.21	1.15	1.58	1.24	1.34	1.13	1.13	1.1
ASIC2	1.48	1.44	1.64	1.47	1.14	1.13	1.08	1.24
CSRNP1	1.91	1.97	1.83	1.81	1.33	1.14	1.18	1.37
RNF125	1.2	1.09	1.41	1.44	1	1.14	1.48	1.53
ZNF614	1	1.41	1.21	1.27	1.09	1.14	1.73	1.61
LOC100996574	1.39	1.13	1.05	1.33	1.53	1.15	1.18	1.23
KDM7A	1.2	1.05	1.17	1.55	1.27	1.15	2.01	1.79
NEDD4	1.2	1.39	1.61	1.7	1.39	1.15	2.04	1.85
LCORL	1.42	1.14	1.24	1.73	1.51	1.15	2.3	2.1
PPP1R1C	1.07	1.14	2.01	1.3	1.16	1.16	2.02	1.07
SLC46A3	1.44	1.36	1.46	1.36	1.46	1.17	1.15	1.45
ZNF184	1.43	1.34	1.37	1.63	1.32	1.18	1.76	1.52
ZNF577	1.24	1.28	1.61	1.72	1.72	1.18	2.64	1.89
ITGB2	1.69	1.48	1.49	1.66	1.04	1.19	1.01	1
MDH1B	1.16	1.47	1.7	1.77	1.05	1.19	1.69	1.56
CADM3	1.41	1.78	1.73	1.72	1.07	1.2	1.03	1.03
PCDHB7	1.78	1.66	1.88	2.04	1.16	1.2	1.23	1.31
GDNF	1.33	1.32	1.25	1.42	1.31	1.2	1.83	1.4
TRAM1L1	1.78	1.35	1.25	1.82	1.22	1.2	2.01	1.69
MAMSTR	1.72	1.5	1.76	1.81	1.24	1.2	1.8	1.71
PDE4D	1.2	1.07	1.36	1.55	1.44	1.2	2.39	1.74
OR51B4	2.25	1.5	1.88	2.63	1.47	1.2	2.36	1.75
LOC107987397	1.05	1.14	1.54	1.27	1.22	1.2	2.09	1.96
ODF3L1	1.66	1.79	1.51	1.42	1.77	1.21	1.3	1.34
CDYL2	1.26	1.16	1.38	1.47	1.58	1.21	1.61	1.64
TET2	1.29	1.18	1.4	1.89	1.33	1.21	2.08	1.83
SENP8	1.49	1.85	1.6	1.57	1.25	1.21	1.87	1.97
CNTNAP2	1.16	1.12	1.42	1.53	1.33	1.22	1.7	1.37
GSTM3	1.26	1.4	1.29	1.4	1.01	1.22	1.35	1.38
PGBD2	1.31	1.2	1.13	1.24	1.04	1.22	1.77	1.41
THRB	1.22	1.35	1.45	1.43	1.09	1.22	1.51	1.54
SFMBT2	1.06	1.16	1.21	1.52	1.21	1.22	1.74	1.55
EPB41L4A	1.07	1.13	1.41	1.37	1.44	1.22	2.19	1.62
FGD6	1.55	1.23	1.57	2.01	1.43	1.22	2.17	1.98
FGD3	1.21	1.62	1.53	1.2	1.25	1.23	1.12	1.12
CALML4	1.12	1.05	1.17	1.33	1.47	1.23	1.75	1.46
ZFHX4	1.64	1.44	1.74	2.05	1.36	1.23	1.66	1.56
LCA5	1.11	1.28	1.2	1.45	1.39	1.23	1.79	1.56
RBM47	1.22	1.28	1.38	1.22	1.45	1.23	1.38	1.63
MTCP1	1.01	1.23	1	1.1	1.11	1.23	1.36	1.65
TBC1D19	1.26	1.37	1.55	1.64	1.19	1.23	1.75	1.8
ZNF665	1.28	1.16	1.18	1.44	1.25	1.23	1.54	1.83
LOC100996716	1.25	1.21	1.19	1.5	1.16	1.24	1.5	1
RCBTB2	1.29	1.22	1.43	1.63	1.22	1.24	1.58	1.48
EMID1	2.32	2.16	2.06	1.54	1.02	1.25	1.11	1.11
SPINK2	1.55	2.41	1.58	2.24	1.53	1.25	1.42	1.26
RAB27A	1.16	1.37	1.44	1.37	1.27	1.25	1.88	1.38
STARD9	1.18	1.17	1.24	1.37	1.24	1.25	1.62	1.5
LZTFL1	1.51	1.23	1.51	1.69	1.4	1.25	2.73	1.94
CAMK1D	1.21	1.35	1.06	1.12	1.26	1.26	1.53	1.16
TIMP4	2.09	1.09	1.27	1.36	1.38	1.26	1.2	1.27
THEMIS2	2.07	3.13	2.28	2.06	1.42	1.26	1.51	1.34
RBMS3	1.29	1.11	1.27	1.5	1.76	1.26	1.97	1.63
PDE3B	1.01	1.25	1.28	1.99	1.56	1.26	1.99	1.88

STBD1	1.29	1.59	1.45	1.67	1.33	1.27	1.61	1.3
ZNF70	1.67	1.61	1.69	1.83	1.03	1.27	1.05	1.35
MGC57346-CRHR1	1.5	1.4	1.5	1.29	1.15	1.27	1.22	1.35
PPIL6	1.45	1.29	1.33	1.01	1.34	1.27	1.63	1.4
IL18BP	1.14	1.15	1.37	1.61	1.45	1.27	2.36	1.62
ZNF701	1.39	1.29	1.65	1.73	1.72	1.27	2.3	1.75
MYEF2	1.44	1.27	1.47	1.9	1.44	1.27	1.97	1.92
ZNF75D	1.29	1.24	1.5	1.73	1.65	1.27	2.45	2.03
LIN28B	1.16	1.05	1.24	1.56	1.48	1.27	2.33	2.08
LIG4	1.31	1.45	1.24	1.68	1.31	1.27	2.8	2.37
C1orf115	1.61	1.84	1.6	1.95	1.19	1.28	1.16	1.37
RORB	1.19	1.27	1.42	1.43	1.26	1.28	1.85	1.46
C10orf12	1.08	1.16	1.39	1.66	1.53	1.28	2.1	1.56
OR1F1	1.02	1.12	1.25	1.49	1.26	1.28	2.61	1.72
HOXA13	1.24	1.22	1.17	1.41	1.52	1.29	1.34	1.31
LOC107985856	1.36	1.41	1.07	1.65	1.22	1.29	2.46	1.43
KLHL4	1.19	1.11	1.32	1.41	1.31	1.29	1.74	1.65
RC3H1	1.02	1.11	1.1	1.61	1.17	1.29	1.85	1.7
TRIM46	1.43	1.42	1.32	1.64	1.31	1.29	1.85	1.78
BEND4	1.12	1.14	1.38	1.45	1.28	1.29	1.8	1.9
C4B	2.02	3.16	3.47	1.98	1.78	1.29	1.86	2.24
SPACA6	1.2	1.48	1.68	1.53	1.23	1.3	1.44	1.31
NEO1	1.5	1.57	1.58	1.67	1.27	1.3	1.49	1.32
ITGAE	1.26	1.06	1.25	1.32	1.52	1.3	1.48	1.36
SOSTDC1	2.94	2.76	3.44	3.46	1.64	1.3	2.18	1.56
SMIM8	1.64	1.32	1.58	1.76	1.4	1.3	2.35	1.85
MTHFD2L	1.13	1.42	1.69	1.63	1.89	1.3	2.11	1.93
MEF2C	1.54	1.55	1.72	1.96	1.48	1.3	1.65	1.97
REL	1.27	1.18	1.43	1.77	1.35	1.3	2.72	2.1
C5	1.14	1.19	1.32	1.62	1.38	1.31	1.99	1.17
GRAMD1B	1.26	1.23	1.06	1.41	1.22	1.31	1.47	1.26
PRDM10	1.16	1.07	1.24	1.36	1.29	1.31	1.8	1.47
FAM13A	1.27	1.2	1.47	1.54	1.35	1.31	1.87	1.57
METTL25	1.43	1.21	1.04	1.18	1.62	1.31	1.95	1.63
BARD1	1.54	1.94	1.9	2.04	1.4	1.31	2.49	1.95
KIF27	1.45	1.1	1.72	2.03	1.6	1.31	3.01	2.09
C17orf100	1.14	1.55	1.17	1.04	1.68	1.32	1.06	1.53
SOGA3	1.01	1.06	1.12	1.52	1.32	1.32	2.15	1.8
ZNF652	1.3	1.21	1.38	1.76	1.47	1.32	1.81	1.89
ZNF224	1.36	1.07	1.37	2.03	1.4	1.32	2.68	2.34
ZNF543	1.02	1.37	1.27	1.64	1.31	1.33	1.49	1.44
HCFC2	1.43	1.21	1.39	1.55	1.52	1.33	1.88	2.05
TARSL2	1.22	1.25	1.38	1.84	1.38	1.33	2.01	2.08
TYW5	1.25	1.24	1.41	1.47	1.52	1.33	2.44	2.15
PRX	1.58	1.76	1.61	1.53	1.41	1.34	1.1	1.29
VASH1	1.38	1.42	1.3	1.26	1.24	1.34	1.18	1.38
RFTN1	1.22	1.35	1.22	1.01	1.64	1.34	1.43	1.46
DLC1	1.44	1.43	1.68	1.54	1.45	1.34	1.67	1.46
N4BP2L1	1.51	1.47	2.04	1.89	1.66	1.34	1.77	1.89
MKRN3	1.24	1.21	1.58	1.53	1.59	1.34	2.34	2.05
MGAT5B	1.15	1.31	1.44	1	1.36	1.35	1.04	1.04
HTRA3	1.6	1.77	1.49	1.21	1.34	1.35	1.14	1.07
LOC105371763	1.84	2.07	1.68	1.64	1.11	1.35	1.62	1.24
ARHGAP10	1.3	1.52	1.37	1.24	1.38	1.35	1.17	1.25
ARPIN	1.49	1.24	1.57	1.52	1.07	1.35	1.2	1.36
GBP2	1.55	1.53	1.36	1.5	1.5	1.35	1.56	1.45
RGCC	1.1	1.1	1.46	1.35	1.84	1.35	1.25	1.48
TBCEL	1.03	1.08	1.15	1.31	1.21	1.35	1.56	1.49
PDGFRL	1.63	1.89	1.99	1.62	1.4	1.35	1.33	1.53
RBM20	1.19	1.25	1.27	1.36	1.38	1.35	1.58	1.53
ZNF718	1.41	1.4	1.49	1.54	1.05	1.35	2.02	1.63
ZNF30	1.28	1.15	1.76	1.93	1.37	1.35	2.16	1.63
AP4E1	1.34	1.18	1.51	2.05	1.95	1.35	2.38	2.25
VWA5A	1.96	1.96	1.86	1.58	1.27	1.36	1.38	1.42
ZNF611	1	1.23	1.28	1.42	1.44	1.36	2.03	1.84
VSIG1	1.17	1.22	1.47	1.8	1.48	1.36	1.31	1.85
SEPSECS	1.24	1.27	1.51	1.54	1.48	1.36	2.25	1.9
PDIK1L	1.37	1.26	1.5	1.78	1.47	1.36	2.25	2.08
RASL11A	1	1.39	1.03	1.57	1.53	1.37	1.27	1.24
NR2F2	1.63	1.55	1.52	1.5	1.39	1.37	1.63	1.36
FBXO36	1.4	1.51	1.74	1.48	1.14	1.37	1.61	1.5
CDADC1	1.19	1.05	1.07	1.4	1.52	1.37	1.81	1.61
LOC107986810	1.13	1.25	1.62	1.44	1.24	1.37	2.25	1.61
ZNF649	1.38	1.33	1.31	1.49	1.53	1.37	1.94	1.69
IKZF4	1.29	1.15	1.2	1.49	1.22	1.37	1.37	1.72
JAK2	1.24	1.11	1.37	1.92	1.34	1.37	2.93	1.95
INTS6L	1.33	1.36	1.43	1.85	1.54	1.37	2.59	2.22
KCNG1	1.47	1.48	1.4	1.59	1.42	1.38	1.03	1.32
SHC4	1.34	1.35	1.63	2.02	1.49	1.38	2.2	1.62
TMEM81	1.21	1.43	1.17	1.8	1.82	1.38	1.72	1.65
CPT1B	1.7	1.41	1.46	1.76	1.81	1.38	2.41	1.81

LOC101929322	2.32	2.06	2.28	3.28	1.77	1.38	1.7	1.95
PLAG1	1.4	1.29	1.75	2.05	1.51	1.38	2.62	2.08
HIST1H3E	1.58	1.81	1.92	1.85	1.72	1.38	2.36	2.49
LOC107987276	1.47	1.44	1.78	1.77	1.42	1.39	1.58	1.62
ZNF816	1.54	1.41	1.78	1.87	1.26	1.39	2.14	1.72
ABHD13	1.28	1.62	1.62	1.75	1.59	1.39	2.02	2.06
GAS2L3	1.09	1.1	1.38	1.39	1.56	1.39	2.07	2.19
KDM8	1.74	1.66	1.45	1.47	1.3	1.4	1.43	1
CDNF	1.7	1.49	1.63	1.65	1.4	1.4	1.54	1.04
TRANK1	2.01	2	1.92	1.99	1.4	1.4	1.67	1.33
GBP1	1.63	1.66	1.56	2.31	1.41	1.4	2.37	1.71
ZBTB10	1.21	1.17	1.4	1.47	1.61	1.4	2.22	1.84
ZNF25	1.45	1.24	1.66	1.74	1.35	1.4	2.45	1.96
RFESD	1.24	1.26	1.85	1.35	1.7	1.4	1.96	2.03
DMXL2	1.03	1.01	1.31	1.53	1.27	1.4	2.45	2.15
RBP5	1.75	1.21	1.67	2.09	1.84	1.4	2.25	2.46
ZIK1	1.2	1.39	1.29	1.42	1.24	1.41	1.42	1.22
GFOD1	1.49	1.3	1.37	1.49	1.31	1.41	1.69	1.54
ADAMTS15	1.45	1.54	1.31	1.24	1.44	1.41	1.28	1.55
ZNF234	1.16	1.06	1.09	1.35	1.76	1.41	2.49	1.71
SDPR	1.49	1.25	1.49	1.48	1.51	1.41	1.96	1.75
ABHD5	1.18	1.28	1.55	1.85	1.66	1.41	2.37	1.77
LURAP1	2.03	1.5	1.51	1.61	1.77	1.41	1.54	1.91
ESR1	1.26	1	1.17	1.64	1.32	1.41	2.23	2.06
TTLL7	1.05	1.04	1.51	1.47	1.24	1.41	3.07	2.44
ABCA13	1.13	1.26	1.46	1.97	1.34	1.41	2.63	2.48
FAM167B	2.37	1.86	1.65	1.95	1.68	1.42	1.83	1.35
AGER	1.71	1.55	1.29	1.43	1.67	1.42	1.43	1.78
ARHGEF9	1.22	1.35	1.52	1.81	1.51	1.42	2.17	1.9
MCOLN3	1.26	1.44	1.72	1.46	1.48	1.42	2.99	2.13
DAAM1	1.5	1.4	1.63	2.16	1.52	1.42	2.3	2.15
CRYBG3	1.44	1.37	1.67	2.47	1.43	1.42	2.99	2.52
AHRR	1.38	1.23	1.38	1.3	1.35	1.43	1.64	1.23
LYRM9	1.38	1.3	1.32	1.31	1.14	1.43	1.18	1.46
ZNF443	1.28	1.2	1.46	1.65	1.21	1.43	2.16	1.52
POU3F2	1.25	1.13	1.32	1.3	1.45	1.43	1.65	1.53
PCDHGB7	1.33	1.86	1.66	1.66	1.47	1.43	1.58	1.56
KIAA1456	1.61	1.59	1.8	1.91	1.5	1.43	1.93	1.62
SLC44A3	1.7	2.13	1.87	1.99	1.71	1.43	2.04	1.76
ZNF2	1.86	1.48	1.54	1.63	1.6	1.43	1.72	1.79
ST3GAL6	1.59	1.56	1.53	1.7	1.79	1.43	2.48	2.35
CXCL10	3.19	3.92	2.99	3.14	1.64	1.44	2.53	1.02
FAM189A2	1.47	1.71	1.79	1.79	1.56	1.44	1.51	1.32
SCUBE3	1.94	2.1	1.78	2.04	1.23	1.44	1.33	1.48
SLC29A4	1.9	2.26	2.37	1.53	1.17	1.44	1	1.53
FBXL13	1.62	1.25	1.43	1.82	1.76	1.44	2.7	1.54
RNF152	1.33	1.12	1.68	1.77	1.72	1.44	2.3	1.66
ZDHHC23	1.72	1.3	1.65	1.95	1.58	1.44	2.23	1.96
KLHL8	1.63	1.33	1.56	1.98	1.74	1.44	2.19	2.04
ENO3	1.32	1.07	1.12	1.36	1.02	1.45	1.44	1.09
ANKRD37	1.89	1.87	1.96	1.8	1.82	1.45	1.36	1.49
LOXL3	1.69	2.44	1.93	1.86	1.23	1.45	1.49	1.49
LOC728485	1.19	1.93	1.26	1.32	1.33	1.45	1.38	1.52
RARB	1.46	1.31	1.3	1.7	1.37	1.45	1.67	1.67
PPP1R9A	1.33	1.47	1.75	2.1	1.49	1.45	2.46	2.1
ZNF354B	1.46	1.33	1.26	1.95	1.31	1.45	2.79	2.46
QRFPR	1.25	1.41	1.4	1.25	1.35	1.46	1.57	1.26
ZNF557	1.13	1.13	1.07	1.19	1.18	1.46	1.41	1.47
ZNF367	1.58	1.41	1.66	2.21	1.4	1.46	2.21	1.67
SATB2	1.21	1.12	1.46	1.74	1.52	1.46	1.7	1.73
POMK	1.15	1.56	1.17	1.81	1.75	1.46	1.08	1.75
INCA1	1.56	1.98	1.63	1.85	2.2	1.46	1.87	1.84
ZNF749	1.96	1.69	2.23	2.06	1.85	1.46	1.94	2.05
OTUD3	1.38	1.16	1.31	1.81	1.41	1.46	2.21	2.06
HIST2H2BF	2.74	1.17	1.83	1.6	1.03	1.46	1.43	2.82
MX2	4.25	4.23	4.91	4.79	1.12	1.47	1.86	1.15
LRRC73	1.63	1.93	1.63	1.58	1.52	1.47	1.31	1.31
VWA5B2	1.45	1.36	1.4	1.45	1.74	1.47	1.58	1.31
ARHGAP24	1.76	2.07	1.83	1.96	1.37	1.47	2.24	1.69
DIXDC1	1.16	1.37	1.39	1.36	1.65	1.47	1.62	1.7
ZNF778	1.47	1.67	1.62	1.7	1.41	1.47	1.87	1.78
ZNF45	1.63	1.55	1.57	2.01	1.7	1.47	1.86	1.83
ZNF347	1.31	1.29	1.45	2.02	1.21	1.47	1.97	2.04
CENPC	1.09	1.19	1.58	1.37	1.5	1.47	2.54	2.1
RAB30	1.56	1.37	1.76	2.06	1.58	1.47	2.16	2.17
MAP3K2	1.43	1.29	1.67	1.99	1.71	1.47	2.89	2.43
NPIPA2	1.19	1.98	2.35	2.49	3.72	1.47	3.79	3.63
SOWAHB	1.92	1.67	1.8	1.62	1.71	1.48	1.19	1.19
NAGS	2.25	1.68	2.13	1.7	1.77	1.48	1.32	1.51
BMP2K	1.44	1	1.44	1.68	1.5	1.48	2.14	1.63
OXCT2	1.59	2.83	1.83	1.79	1.59	1.48	1.52	1.67
NPR3	1.89	2.07	2.13	2.13	1.39	1.48	1.85	1.69

ANKRD45	1.51	1.67	2.06	2.18	1.67	1.48	1.6	1.84
TRPS1	1.25	1.19	1.59	1.6	1.56	1.48	2.23	1.84
ZFP82	1.27	1.54	1.64	1.89	1.5	1.48	2.33	1.89
KLHL11	1.38	1.31	1.54	1.81	1.69	1.48	1.75	1.99
SLC35A1	1.68	1.3	1.76	1.7	1.51	1.48	2.48	1.99
ZNF41	1.61	1.31	1.7	1.84	1.7	1.48	2.2	2.2
ALG10B	1.16	1.13	1.15	1.53	1.38	1.48	2.62	2.24
ZNF26	1.29	1.43	1.88	2.17	1.62	1.48	2.53	2.48
USP53	1.25	1.04	1.51	1.93	1.48	1.48	2.67	2.53
HPSE	1.96	1.64	2.26	1.76	1.52	1.49	2.18	1.5
NRIP3	1.55	1.69	1.43	1.46	1.64	1.49	1.52	1.73
LCOR	1.24	1.07	1.44	1.91	1.52	1.49	2.53	2.11
TRIM59	1.39	1.32	1.35	1.92	1.54	1.49	2.87	2.39
HHIPL2	1.51	1.32	1.9	2.35	1.32	1.49	2.68	2.71
GYPC	2.1	1.98	1.83	1.41	1.19	1.5	1.17	1.25
CASZ1	1.43	1.47	1.49	1.29	1.47	1.5	1.1	1.59
SETBP1	1.31	1.49	1.81	1.87	1.25	1.5	1.58	1.78
ZNF596	1.3	1.21	1.33	1.76	1.49	1.5	1.71	1.79
PDGFD	1.47	1.46	1.43	1.74	1.21	1.5	2.26	1.84
RAB42	1.68	1.13	1.42	2.08	1.47	1.5	1.66	2.23
SLFN12	1.29	1.17	1.51	1.77	1.57	1.5	2.73	2.33
CPNE5	1.87	2.21	1.66	1.79	2.14	1.51	1.57	1.3
ZNF792	1.7	1.92	1.72	1.95	1.55	1.51	1.35	1.33
ZNF132	1.18	1.21	1.37	1.14	1.06	1.51	1.75	1.34
SRGAP1	1.11	1.2	1.26	1.33	1.56	1.51	1.63	1.46
SAMD13	1.18	1.41	1.58	1.07	1.27	1.51	2.34	1.46
GK	1.39	1.26	1.6	1.67	1.64	1.51	2.16	1.69
LOC107986211	1.7	1.79	1.81	1.87	1.54	1.51	1.89	1.87
TLE2	2.1	2.27	2.3	1.88	1.22	1.52	1.28	1.04
DHRS4L2	1.76	1.57	1.78	1.49	1.37	1.52	1.01	1.19
NUPR2	1.94	1.49	1.42	2.06	1.59	1.52	1.17	1.49
LHFPL4	1.81	1.66	1.63	1.41	1.61	1.52	1.29	1.57
RARRES3	2.07	2.59	2.33	2.58	1.58	1.52	2.37	1.57
ZBTB34	1.38	1.32	1.59	1.78	1.52	1.52	2.03	1.65
CCL24	1.15	1.29	1.52	1.66	1.79	1.52	2.37	1.85
CCNE2	1.67	1.97	2	2.19	2.1	1.52	2.42	1.85
ZNF14	1.66	1.37	2.1	2.42	1.4	1.52	3.21	2.5
FAM200B	1.78	1.52	1.84	2.08	1.86	1.52	2.73	2.53
HCAR3	2.04	1.73	1.83	1.72	1.89	1.53	1.1	1.45
NRG2	1.47	1.35	1.23	1.39	1.45	1.53	1.13	1.56
CCDC40	1.18	1.46	1.73	1.7	1.13	1.53	1.5	1.64
IFIH1	2.74	2.3	2.88	3.53	1.58	1.53	2.56	1.79
SETDB2	1.2	1.01	1.76	1.82	1.71	1.53	2.39	1.8
GIN1	1.55	1.44	1.24	1.67	1.66	1.53	2.22	1.84
PRRX1	1.75	1.7	1.92	2.42	1.81	1.53	2.58	2.1
LOC100506127	1.63	1.31	1.74	2.07	1.74	1.53	2.35	2.12
FOXD4	1.34	1.27	1.57	1.95	1.47	1.54	1.47	1.4
STXBP5	1.87	1.35	1.49	2.08	1.58	1.54	2.04	1.94
MCOLN2	1.9	1.66	1.84	2.14	2.15	1.54	3.06	2.52
ZNF273	1.77	1.36	2.14	1.83	2.25	1.54	2.93	3.17
RNF39	1.98	2.1	1.63	1.78	1.46	1.55	1.26	1.16
ZIC4	1.48	1.48	1.73	2.53	1.68	1.55	2.04	2.05
PIM1	1.84	2.13	2.14	1.76	1.77	1.55	1.63	2.2
XAF1	1.45	1.65	2.08	2.56	1.48	1.55	2.98	2.37
ZNF780B	1.24	1.21	1.73	2.19	1.55	1.55	3.04	2.51
ZNF780A	1.58	1.69	1.7	2.13	1.43	1.55	2.95	3.02
KIAA1328	1.41	1.48	1.28	1.66	1.73	1.56	1.87	1.55
PTPRCAP	2.35	1.53	2.08	1.56	1.72	1.56	1.2	1.66
TFCP2L1	1.76	1.93	1.76	1.68	1.4	1.56	1.42	1.66
PRKCE	1.43	1.38	1.39	1.77	1.46	1.56	1.67	1.69
FAM184A	1.25	1.32	1.54	1.73	1.26	1.56	3.07	2.04
AQP3	1.48	1.77	1.17	1.01	1.43	1.57	1.23	1.51
FUT1	1.57	1.6	1.69	1.49	1.5	1.57	1.44	1.55
SEMA4D	1.52	1.59	1.76	1.56	1.36	1.57	1.39	1.57
NUTM2A	2.33	1.81	2.01	2.07	1.78	1.57	1.88	1.7
XDH	1.47	1.54	1.52	1.35	1.85	1.57	2.06	1.73
ZNF835	1.75	1.88	1.79	1.66	1.62	1.57	1.43	1.81
MCM9	1.33	1.25	1.78	1.61	1.86	1.57	2.12	1.83
MFSD8	1.37	1.37	1.48	1.85	1.57	1.57	2.56	2.27
COL8A2	1.77	1.33	1.53	1.49	1.19	1.58	1.28	1.31
LOX	2.14	1.81	2.25	2.48	1.75	1.58	2.35	1.74
FAM129A	2.96	2.92	2.81	2.92	1.81	1.58	1.81	1.96
PPM1K	1.67	1.37	1.85	2.07	1.63	1.58	2.33	1.99
METTL4	1.45	1.11	1.81	1.63	1.89	1.58	2.66	2.32
ATP8A1	1.14	1.18	1.21	1.65	1.33	1.58	2.98	2.72
CCDC126	2.49	2.04	2.38	2.55	2.11	1.58	2.41	3.05
TSACC	1.16	1.61	1.19	1.4	1.51	1.59	1.46	1.5
ZNF35	1.77	1.35	1.45	1.79	1.36	1.59	1.63	1.5
PCDH18	1.62	2.13	2.28	2.34	1.58	1.59	1.82	1.54
LOC101927789	2.4	1.97	2.64	1.91	1.52	1.59	2.41	1.6
KIAA1614	1.61	1.7	1.69	1.58	1.43	1.59	1.54	1.79
CCDC171	1.39	1.6	1.56	1.97	1.44	1.59	2.43	1.86
RAET1G	1.94	1.87	1.94	1.68	1.98	1.59	1.77	1.89
ZNF264	1.92	1.75	1.64	2.17	2	1.59	1.91	2
CEP162	1.37	1.78	1.71	1.8	1.44	1.59	3.13	2.19
FMN1	1.34	1.3	1.49	2.13	1.67	1.59	2.61	2.24

AGAP5	1.98	1.29	1.88	1.58	1.7	1.59	2.41	2.26
P2RY1	1.78	1.39	1.71	2.34	1.35	1.59	2.22	2.27
NBEAL1	1.27	1.34	1.39	1.9	1.42	1.59	3.01	2.62
ZNF605	1.66	1.54	2	2.48	1.82	1.59	3.61	3.08
GXYLT2	1.81	2.17	2.04	1.96	1.28	1.6	1.53	1.17
ICA1L	1.3	1.63	1.21	2.03	1.52	1.6	1.91	1.59
CCDC187	1.72	1.58	1.55	1.41	1.53	1.6	1.61	1.6
P2RX6	1.79	1.79	1.56	1.45	1.62	1.6	1.52	1.77
ARHGAP32	1.53	1.48	1.66	1.83	1.64	1.6	1.94	1.92
GPM6B	1.4	1.39	1.72	1.74	1.59	1.6	2.5	2.08
CLOCK	1.33	1.32	1.51	1.99	1.74	1.6	2.56	2.34
APC2	1.56	1.63	1.51	1.32	1.37	1.61	1.08	1.38
TMIGD2	1.08	1.79	1.68	2.48	2.03	1.61	2.48	1.76
ARID3A	2.1	2.21	2	2.61	1.58	1.61	1.79	1.89
PBX4	1.5	1.69	1.53	1.54	1.66	1.61	1.71	1.95
ZNF181	1.56	1.47	1.36	2.08	1.69	1.61	2.74	2.08
ENTPD7	1.59	1.43	1.54	1.89	1.46	1.61	2.3	2.14
GPSM3	2.03	2.33	1.74	1.81	1.64	1.61	1.56	2.25
MYO5C	1.61	1.26	1.85	1.8	1.59	1.61	2.5	2.48
LOC653602	1.71	1.33	1.72	1.92	1.68	1.61	3.11	2.92
DAPL1	2.9	1.5	2.81	2.2	1.14	1.62	1.86	1.38
HECW1	1.16	1.21	1.34	1.21	1.65	1.62	1.76	1.7
CARD6	1.24	1.4	1.25	1.53	1.38	1.62	1.55	1.74
ENOX1	1.57	1.21	1.42	1.59	1.58	1.62	1.71	1.75
RGL3	1.64	1.22	1.41	1.47	1.22	1.62	1.92	1.93
PCDHB11	1.84	2.12	2.07	1.81	1.69	1.62	1.82	1.94
NPIPB11	1.41	1.04	1.16	1.97	1.01	1.62	1.45	2.06
CEP44	1.45	1.14	1.55	2	1.95	1.62	3.46	2.34
KLHL28	1.65	1.82	1.94	1.96	1.25	1.62	2.9	2.53
KANK3	1.65	1.53	1.25	1.26	1.57	1.63	1.31	1.48
TMEM253	1.38	1.85	1.2	1.1	1.84	1.63	1.78	1.53
MN1	1.78	1.85	1.62	1.71	1.38	1.63	1.42	1.55
ZNF432	1.37	1.32	1.33	1.84	1.43	1.63	2.42	1.91
LOC100996598	1.23	1.96	1.5	1.89	1.91	1.63	2.8	1.92
CDH1	1.49	1.67	1.74	1.67	1.75	1.63	2.09	2.06
GPR173	1.62	1.55	1.84	1.75	1.4	1.63	2.22	2.09
FBXL2	1.48	1.87	1.96	1.88	1.86	1.63	2.76	2.43
FAM179B	1.88	1.26	1.98	2.26	1.77	1.63	2.72	2.74
PHOSPHO2	1.7	1.86	1.69	2.22	1.86	1.63	2.57	3.31
TRIM39-RPP21	1.22	2.07	1.89	2.11	2.83	1.64	1.88	1.33
PCDHGB2	1.17	1.46	1.95	2.18	1.32	1.64	1.95	1.36
E2F2	2.21	1.92	1.94	2.35	1.78	1.64	1.43	1.68
C1orf233	2.28	2.33	1.93	2.4	1.73	1.64	1.43	1.69
LOC102724488	1.56	1.51	1.48	1.95	1.22	1.64	1.89	1.81
PLA2R1	1.39	1.13	1.5	1.68	1.42	1.64	2.38	1.96
KIAA1107	1.49	1.56	1.8	1.93	1.49	1.64	2.97	2.16
NETO1	1.49	1.62	2.31	2.02	2.23	1.64	2.54	2.38
CCDC191	1.45	1.29	1.86	2.03	1.44	1.64	3.15	2.44
WDPCP	1.98	1.61	1.81	2.12	2.18	1.64	2.87	2.95
VWA7	1.31	1.31	1.58	1.17	1.09	1.65	1.53	1.47
NAT1	1.22	1.42	1.56	2.01	1.26	1.65	1.94	1.84
HIVEP1	1.7	1.36	1.81	2.29	1.95	1.65	2.74	2.17
NCOA7	1.8	1.62	1.99	2.25	1.44	1.65	3.05	2.36
C15orf38-AP3S2	2.53	2.77	2.84	2.94	1.81	1.65	2.76	2.45
SNX16	1.7	1.79	1.85	2.32	2.21	1.65	3.05	2.62
GSG2	1.43	1.74	1.3	1.38	1.89	1.66	1.22	1.27
ZSCAN16	1.93	1.65	1.28	1.55	1.47	1.66	1.79	1.99
KIAA1958	1.69	1.45	1.73	2.04	1.69	1.66	2.4	2.38
KCNJ8	1.41	1.66	1.36	1.31	1.95	1.67	1.85	1.81
DCUN1D3	1.32	1.35	1.58	1.86	1.42	1.67	2.01	1.82
GAPDHS	1.14	1.21	1.32	1.12	1.49	1.67	1.63	1.85
MUC1	2.04	2.18	2.04	1.92	1.45	1.67	1.4	2
ZFP69B	1.25	1.45	1.51	2.07	1.8	1.67	2.27	2.26
ZNF548	2.02	1.77	2	2.58	2.4	1.67	2.68	2.28
SULT4A1	1.59	1.23	1.68	1.88	1.86	1.67	1.91	2.31
TSPAN2	1.64	1.95	2.04	2.19	1.82	1.67	2.16	2.36
SERAC1	1.45	1.44	1.6	2.03	1.82	1.67	2.63	2.38
LOC105379272	1.75	1.15	1.97	2.22	2.89	1.67	3.21	3.36
MINOS1-NBL1	5.35	7.81	1.63	3.97	1.85	1.67	4.62	4.21
CCDC88B	2.13	1.97	1.56	1.89	1.8	1.68	2.04	1.65
LOC107984648	2.24	1.79	2.33	2.46	2.08	1.68	2.5	1.68
ARMCX1	1.51	2.08	1.6	1.59	2.02	1.68	1.79	1.87
SLC2A3	3.59	4.13	3.82	3.84	1.67	1.68	1.99	1.93
SLC9A9	1.92	1.76	1.85	1.66	1.72	1.68	2.07	1.93
IL1A	1.24	1.36	1.5	2.04	1.92	1.68	2.63	1.98
KLF7	1.61	1.61	1.76	2.01	1.86	1.68	2.53	2.17
VCX3B	1.77	2.1	1.75	2.44	1.59	1.68	2.32	2.31
ENPP1	2.45	1.96	2.28	2.56	1.65	1.68	2.31	2.58
HMOX1	1.29	1.69	1.38	1.53	1.65	1.69	1.47	1.74

MMRN2	2.95	3.1	2.79	2.76	1.46	1.69	1.72	1.75
PRSS8	1.95	2.6	2.36	1.88	1.48	1.69	1.11	1.77
DDO	2.09	2.54	3.07	2.58	2.3	1.69	2.33	2.05
DENND5B	1.52	1.28	1.69	2.05	1.67	1.69	2.48	2.11
FAM228B	1.9	1.98	1.31	3.02	1.25	1.69	2.08	2.18
ZNF518A	1.46	1.31	1.79	2.09	1.6	1.69	2.9	2.34
CCDC66	1.73	1.44	1.54	1.65	1.11	1.69	2.81	2.41
ZNF720	1.54	1.85	1.78	2.22	2.26	1.69	2.51	2.48
TCTEX1D4	1.56	1.31	1.42	1.26	1.57	1.69	2.57	2.58
NPIPB8	1.19	1.55	1.69	1.65	1.32	1.69	3.83	2.91
SORCS1	1.45	1.89	1.62	1.71	1.19	1.7	1.58	1.41
LOC107987017	2.16	1.56	1.38	2.37	2.1	1.7	1.64	1.52
HAP1	2.13	2.39	2.19	2.07	1.79	1.7	1.39	1.55
NPTXR	1.62	1.8	1.6	1.62	1.66	1.7	1.36	1.63
PIPOX	1.33	1.3	1.36	1.38	1.79	1.7	1.56	1.87
UPK3B	1.93	2.11	1.8	2.14	2.72	1.7	2.06	1.9
ZSCAN5A	2.11	1.76	1.64	1.41	1.62	1.7	1.66	2.16
ZNF136	1.61	1.39	1.47	2.13	1.49	1.7	2.65	2.6
MGLL	1.09	1.46	1.28	1.16	1.7	1.71	1.25	1.35
CCNA1	1.45	1.42	1.63	1.81	1.89	1.71	1.51	1.35
WRB-SH3BGR	1.09	1.25	1.8	1.02	2.08	1.71	1.99	1.61
KDM4D	1.61	1.66	1.74	1.95	2.14	1.71	2.06	2.02
TIGD2	1.51	1.49	1.79	2	1.8	1.71	2.19	2.18
ZNF33B	1.71	1.55	1.88	2.52	1.74	1.71	3.6	2.55
HOGA1	2.18	1.61	1.48	1.93	1.43	1.72	1.42	1.6
RCSD1	1.91	1.91	1.94	2.05	2.18	1.72	1.66	1.67
ICAM2	1.41	1.52	2.04	1.37	1.34	1.72	1.32	1.69
LOC101929747	1.49	1.68	1.53	1.1	1.83	1.72	1.03	1.72
PRKAR2B	1.12	1.22	1.28	1.42	1.53	1.72	2.07	1.73
ZNF677	1.26	1.47	1.42	1.72	1.83	1.72	2.16	2.06
PUS7L	1.3	1.3	1.4	1.71	1.68	1.72	2.95	2.23
ZNF607	1.81	1.68	1.82	2.11	1.75	1.72	2.38	2.25
ZNF468	1.12	1.42	1.63	2.16	1.8	1.72	2.54	2.28
CNKSR2	2.24	1.9	2.13	2.27	1.55	1.72	2.52	2.36
STK17B	1.8	1.52	1.98	2.2	1.87	1.72	2.81	2.39
PCDHB13	1.91	1.62	2.28	2.05	1.43	1.72	2.05	2.49
LYZ	2.19	1.44	1.79	1.95	1.72	1.72	2.47	2.59
CPLX1	2.43	2.41	2.41	2.32	1.71	1.73	1.51	1.14
ZNF837	1.31	1.57	1.59	1.77	1.47	1.73	1.18	1.34
IRF5	1.58	1.32	1.25	1.5	1.7	1.73	1.31	1.41
CLCF1	1.64	1.74	1.89	2.02	2.15	1.73	1.77	1.66
GLIPR1	1.26	1.3	1.16	1.26	1.93	1.73	3.06	1.91
C5orf34	1.35	1.07	1.58	1.81	1.4	1.73	2.6	2.05
ZSCAN22	1.69	1.99	1.59	1.42	1.63	1.73	2.08	2.2
TMTC1	1.31	1.25	1.55	1.71	1.81	1.73	2.51	2.27
ZNF182	1.56	1.57	1.85	1.91	1.59	1.73	2.82	2.57
44256	1.27	1.39	1.54	1.23	1.59	1.74	1.81	1.56
PRAME	1.3	1.86	1.67	1.81	1.67	1.74	1.36	1.72
LDAH	1.62	1.46	1.71	1.75	2.09	1.74	2.43	2.1
ECHDC2	1.41	1.25	1.41	1.41	1.82	1.74	2.5	2.38
KIF21A	1.43	1.54	1.66	2.18	1.68	1.74	3.03	2.52
NLRP6	1.55	1.63	1.51	1.29	1.58	1.75	1.31	1.33
BCL2	1.64	1.16	1.33	1.25	1.34	1.75	1.8	1.45
RIBC1	1.58	1.55	1.5	1.94	1.65	1.75	1.55	1.7
SEMA6A	2.64	2.51	2.67	2.84	1.62	1.75	1.96	1.81
LOC107984110	1.61	1.63	1.25	1.8	1.57	1.75	2.36	1.82
SNCAIP	1.88	1.76	2.09	2.74	1.69	1.75	2.18	1.96
ZW10	1.76	1.39	1.91	2.03	1.93	1.75	2.77	2
MAP3K1	2.1	1.73	2.23	2.29	1.83	1.75	2.32	2.09
ZSCAN9	2.72	1.51	2.7	2.96	1.94	1.75	2.62	2.13
KIF9	2.33	1.92	1.72	2.41	2.67	1.75	2.24	2.22
TTC25	1.8	2.59	1.97	1.81	1.86	1.75	1.74	2.34
PRMT9	1.6	1.54	1.69	2.23	1.79	1.75	2.54	2.4
ARL13B	1.86	1.59	1.89	2.31	1.66	1.75	3.36	2.59
SESTD1	1.5	1.5	1.77	2.25	1.94	1.75	2.92	2.61
LYST	1.52	1.43	1.83	2.37	1.63	1.75	3.31	2.92
PKDCC	2.03	2.21	2.09	2	1.54	1.76	1.78	1.61
ZNF445	2.14	1.92	2.09	2.42	1.81	1.76	2.28	1.92
SPICE1	1.65	1.45	2.32	2.42	1.64	1.76	2.81	2.23
SPAG1	1.61	1.66	1.89	1.67	1.52	1.76	2.79	2.27
ZSCAN26	2.54	2.9	3	3.33	1.46	1.76	2.61	2.31
CEP83	1.65	1.78	1.72	1.81	1.78	1.76	3.02	2.33
ATXN3	1.46	1.35	1.6	1.98	1.84	1.76	2.72	2.44
ZNF44	1.91	1.67	2.06	2.49	1.7	1.76	2.96	2.59
CASC5	1.36	1.28	1.67	1.48	1.68	1.76	3.29	2.6
ARHGEF26	2.09	1.39	1.82	1.96	1.64	1.76	2.68	2.65
OSBP2	1.83	2.15	1.95	1.91	1.83	1.77	1.54	1.65
CASKIN1	1.66	1.41	1.43	1.55	1.69	1.77	1.87	1.84
HRH1	1.38	1.27	1.29	1.81	1.86	1.77	2.29	1.94
TREX2	1.97	2.01	2.05	1.37	1.65	1.77	2.35	2.03
ARHGAP33	1.73	1.65	2.04	2.01	1.61	1.77	2.24	2.07
MBD5	1.37	1.38	1.67	1.92	1.73	1.77	2.38	2.1

DTWD2	1.65	1.41	1.54	2.01	1.64	1.77	2.45	2.21
ZNF736	1.75	2.32	2.63	2.04	1.83	1.77	3.01	2.77
RNF32	1.6	1.79	2.11	2.33	1.86	1.77	2.6	2.78
AKR1C2	1.7	1.41	1.42	1	1.34	1.78	1.82	1.76
ZNF155	1.44	1.6	1.58	1.51	1.45	1.78	2.25	1.95
KIAA1211	1.64	1.63	1.74	1.74	1.72	1.78	2.44	2.06
LOC401052	1.39	1.77	1.75	1.48	1.83	1.78	1.82	2.32
TTLL3	1.97	2.12	1.69	2.3	2.24	1.78	2.74	2.61
SAYSD1	1.52	1.28	2.04	1.61	1.98	1.79	1.88	1.43
AK5	1.3	1.02	1.19	1.03	1.76	1.79	1.67	1.61
HID1	3.1	2.88	2.56	2.42	1.96	1.79	1.33	1.75
ATP8B3	1.86	1.55	1.85	1.78	1.55	1.79	2	1.83
GPR39	2.01	1.86	1.94	1.65	2.09	1.79	1.79	1.92
KLLN	1.64	1.4	1.38	1.61	2.06	1.79	2.45	2
FAM149A	2.03	2.13	1.9	1.88	1.82	1.79	1.78	2.08
SIK2	1.88	1.81	2.02	1.76	2.02	1.79	1.9	2.21
SGK494	1.87	1.49	1.59	2.01	1.73	1.79	2.84	2.28
MSANTD2	1.57	1.79	2.07	2.35	1.89	1.79	3.14	2.4
YOD1	1.78	1.34	1.84	2.2	1.83	1.79	3.53	3.16
RPS6KA6	1.4	1.7	2.03	2.12	2.35	1.79	3.36	3.27
DOK6	1.23	1.28	1.55	1.93	1.29	1.8	1.96	1.31
STAT5A	1.66	1.97	1.78	1.79	1.96	1.8	1.52	1.83
SYNM	2.12	1.74	2.31	2.15	1.7	1.8	2.02	2.07
ZSCAN30	1.62	1.63	2.17	2	1.54	1.8	2.5	2.18
ZNF407	1.6	1.28	1.8	1.94	1.96	1.8	2.42	2.24
PLAGL1	1.83	1.87	2.15	2.21	2.07	1.8	2.82	2.28
ZNF772	1.72	1.61	2.1	2.18	1.76	1.8	2.11	2.38
CEP57L1	1.22	1.48	2.18	2.05	1.58	1.8	3.49	2.57
DENND4A	1.41	1.36	1.83	2.2	1.59	1.8	3.03	2.66
PRSS22	1.81	1.95	1.81	2.14	1.48	1.81	1.46	1.12
IL22RA1	2.46	2.42	2.08	2.15	2.04	1.81	1.79	1.64
KSR1	1.53	1.69	1.52	1.46	1.6	1.81	2.16	2.08
CASP10	2.04	2.01	2.02	2.11	2.26	1.81	2.01	2.13
ZNF814	1.63	1.68	1.85	2.1	1.73	1.81	2.4	2.29
SHOX	1.53	1.61	1.58	1.87	1.71	1.81	2.82	2.29
RAPGEF2	1.91	1.54	1.86	2.35	1.86	1.81	2.91	2.65
ZBTB21	1.79	1.71	2.38	2.63	2.11	1.81	3.58	2.78
AUNIP	2.23	2.09	2.19	2.16	2.45	1.82	2.6	1.99
ZNF20	1.53	1.29	1.8	1.97	1.41	1.82	2.02	2.2
ROR1	1.76	1.3	2.03	1.99	1.97	1.82	2.24	2.38
FRS2	1.82	1.47	1.86	2.24	2.18	1.82	3.21	2.76
FNTB	3.98	3.08	2.87	3.73	3.42	1.82	3.34	2.77
FILIP1L	3.12	3.29	3.95	4.32	1.9	1.82	3.38	3
LOC107984155	1.88	1.69	1.81	1.69	2.26	1.83	1.64	1.66
DISP1	1.74	1.7	1.76	1.8	1.65	1.83	1.68	1.75
TMEM182	1.57	1.6	1.39	2.68	2.04	1.83	2.21	1.97
USP28	1.8	1.63	1.9	1.77	2.04	1.83	2.24	2.09
KLF9	1.61	1.6	1.89	2.11	1.69	1.83	1.99	2.1
SERPINB8	1.58	1.83	1.8	1.79	1.77	1.83	2.22	2.12
HELQ	1.46	1.8	2.15	1.95	1.98	1.83	2.65	2.21
ZNF721	1.38	1.21	1.44	2.14	1.46	1.83	2.96	2.36
FAIM	2.08	1.69	2.88	2.13	2.11	1.83	2.34	2.54
PRKCH	2.1	1.85	2	2.24	2.24	1.83	2.4	2.76
C18orf54	1.93	1.69	1.82	2.41	1.99	1.83	3.7	2.84
PHYHIPL	1.46	1.39	1.6	2.08	1.62	1.83	3.59	3.04
ARIH2OS	1.71	1.86	1.66	2.59	1.72	1.84	1.91	1.85
NAP1L2	1.77	1.7	1.85	2.23	2.11	1.84	2.54	1.98
ADGRL3	2.17	1.88	2.32	2.43	1.6	1.84	2.19	2.04
GRAPL	1.92	1.99	1.74	2.04	2.02	1.84	2.53	2.28
MICU3	1.94	1.11	1.25	1.59	1.34	1.84	2.51	2.38
ZFX	1.58	1.6	1.79	2.18	1.61	1.84	2.47	2.52
PLSCR4	2.36	2.08	2.81	3.45	2.26	1.84	3.15	3.29
C5orf66	1.48	1.42	1.94	2.11	1.96	1.85	2.18	1.65
ALX4	2.19	2.2	2.61	2.05	1.87	1.85	1.7	2.22
CCNJ	1.74	1.57	1.69	2.13	1.66	1.85	2.5	2.26
ATAD5	1.39	1.72	1.47	2.09	1.55	1.85	2.94	2.41
ANK3	1.67	1.79	2.07	2.23	2.23	1.85	2.85	2.52
ERO1B	2.06	1.86	2.35	2.71	1.72	1.85	3.49	2.53
LMLN	1.81	1.66	1.98	2.44	1.97	1.85	3.16	2.6
FAM111B	1.89	1.45	2.23	2.36	1.97	1.85	3.29	2.65
ZFP30	1.84	1.55	2.26	2.47	1.75	1.85	2.86	2.71
LRCH2	1.53	1.43	2.05	1.9	1.61	1.85	3.54	2.89
USP45	1.74	1.99	2.48	2.23	2.73	1.85	4.67	4.02
USP27X	2.24	1.99	1.99	1.88	1.69	1.86	1.64	1.94
PDZD7	2.47	2.39	1.94	1.98	1.93	1.86	2.11	2.03
DNMT3B	2.15	2.31	2.52	2.22	2.28	1.86	2.14	2.05
ZNF641	1.82	2.12	1.77	1.77	1.93	1.86	2.47	2.21
NUP62CL	1.99	1.69	1.63	2.6	1.6	1.86	2.6	2.41
SPATA17	2.51	2.45	2.79	2.81	2.57	1.86	3.29	2.46
RBM7	1.93	1.43	1.81	2.13	2.02	1.86	2.89	2.49
ZNF514	2.11	1.85	2.06	2.22	2.2	1.86	3.05	2.68
B4GALT6	1.88	1.51	2.1	2.42	1.89	1.86	2.81	2.85
PRR36	2.79	2.9	2.4	2.59	1.65	1.87	1.23	1.51
VAX2	2.13	2.23	1.42	1.32	1.87	1.87	1.38	1.51
C19orf57	2.14	2.12	2	1.75	1.47	1.87	1.78	1.55

CASC10	1.59	1.86	1.81	1.75	1.76	1.87	1.53	1.59
NKX3-1	1.67	1.73	1.78	1.97	1.78	1.87	2.01	1.84
LOC105370687	2.01	1.84	1.88	1.47	1.97	1.87	2.22	1.93
ZNF606	1.72	1.37	2	2.28	1.8	1.87	2.37	2.03
CARD11	1.73	1.74	1.75	1.85	2.1	1.87	1.65	2.18
ALG10	1.35	1.39	1.75	1.78	1.77	1.87	2.52	2.26
NIPAL2	1.66	1.36	1.88	2	1.95	1.87	2.54	2.33
PFKFB2	1.58	1.44	1.49	1.98	1.69	1.87	2.63	2.36
ZNF793	1.67	1.65	1.74	2.25	1.76	1.87	2.91	2.61
EPHA3	2.22	1.68	2.18	2.66	1.53	1.87	2.09	2.64
SNRNP48	1.68	1.95	1.74	2.67	2.4	1.87	3.99	3.32
CISH	2.19	1.94	1.84	1.74	1.55	1.88	1.24	1.53
DEPTOR	1.9	2.13	2.16	2.01	1.77	1.88	1.96	1.62
C1orf116	1.2	1.62	1.44	1.24	2.15	1.88	1.67	1.68
RNF144A	2.12	2.12	1.96	2.04	1.73	1.88	1.81	1.97
TMEM242	1.96	1.91	2.09	2.24	1.9	1.88	2.75	2.42
ADPRM	1.76	1.59	1.72	1.29	2.26	1.88	2.37	2.47
RPGR	1.31	1.44	1.76	2.28	2	1.88	2.81	2.71
ZNF675	1.89	2.22	2.05	2.62	1.91	1.88	3.6	3.47
CYP2J2	1.68	1.91	1.88	2.24	2.04	1.89	1.83	1.5
PTPRN2	1.66	1.71	1.82	1.66	1.82	1.89	1.78	1.86
FSTL3	2.09	2.42	2.11	1.98	2.3	1.89	1.78	2.17
SERTM1	1.68	1.51	1.69	2.12	2.21	1.89	2.84	2.25
IDI2	1.42	1.24	1.86	2.01	1.96	1.89	1.98	2.29
ZBTB3	1.73	1.73	1.82	1.91	1.97	1.89	2.21	2.3
ATAD3C	2.06	2.27	2.03	2.48	1.86	1.89	2.19	2.43
DYX1C1	1.66	1.54	1.43	2.02	2.16	1.89	2.12	2.47
SMAD9	1.59	1.67	2.11	2.37	2.17	1.89	2.73	2.59
DZIP1L	1.92	1.57	2.28	2.19	1.66	1.89	1.93	2.66
FAM83B	1.65	1.58	1.81	2.15	1.62	1.89	2.83	2.83
ENTPD1	2.47	2.32	2.42	2.43	2.84	1.89	3.47	3.22
SPRN	1.81	1.65	1.66	1.62	1.91	1.9	2.2	1.96
STAT4	1.65	1.49	1.73	1.46	1.62	1.9	2.01	1.98
PXYLP1	3.08	2.69	2.96	3.67	2.3	1.9	2.72	2.35
DSEL	1.84	1.51	1.89	2.11	1.77	1.9	2.62	2.49
CEP97	1.38	1.28	1.56	2.09	1.83	1.9	3.24	2.87
C7orf55-LUC7L2	2.22	3.8	2.86	4.93	4.01	1.9	4.73	4.66
CYP26B1	2.41	2.55	2.49	2.39	1.85	1.91	1.46	1.63
MAML3	2.16	1.94	2.18	2.44	2.25	1.91	2.51	2.19
MTM1	1.91	1.32	1.85	2.26	1.55	1.91	2.99	2.31
SPIN3	1.7	1.64	2.2	2.02	2.27	1.91	3.15	2.53
OGFRL1	1.91	1.77	2.28	2.61	2.2	1.91	3.55	2.91
RAB20	2.46	2.52	2.49	1.83	1.82	1.92	1.6	1.44
C17orf67	1.91	1.98	1.86	1.79	1.41	1.92	1.75	1.52
CALHM2	1.82	1.47	1.3	1.35	2.05	1.92	1.6	1.84
ZNF202	1.73	2.06	1.99	2.01	2.15	1.92	2.68	2.11
C2orf27A	1.89	2.03	2.08	2.39	2.5	1.92	2.26	2.35
THAP10	2.33	2.08	2.36	2.48	2.09	1.92	2.47	2.35
ZNF267	2.12	2.16	1.97	2.18	1.64	1.92	3.28	2.45
STXBP4	1.29	1.22	1.75	1.83	1.69	1.92	3.05	2.59
GBP3	2.47	2.25	2.39	3.05	1.82	1.92	3.25	2.77
SH3BGRL2	2.11	1.79	1.83	2.32	1.94	1.93	2.58	2.36
MOXD1	1.86	1.99	2.23	2.42	1.94	1.93	3.05	2.4
LECT1	2.43	3.49	2.85	2.59	1.66	1.93	1.75	2.46
ABCA5	1.66	1.34	1.94	2.3	1.81	1.93	3.41	2.99
BAIAP3	2.46	2.28	2.14	1.94	1.39	1.94	1.11	1.44
SLC30A4	1.78	1.28	2.19	2.46	2	1.94	2.06	1.71
NPAS1	1.85	2.66	2.26	1.57	1.81	1.94	1.87	1.73
PRDM5	1.56	1.28	1.64	1.49	1.37	1.94	1.81	1.8
ALDH5A1	1.77	1.95	2.03	2.35	1.87	1.94	1.85	1.92
PTPRM	2.3	2	2.2	2.56	2.04	1.94	2.84	2.15
MFSD4B	2.12	2.17	2.17	2.08	1.92	1.94	2.51	2.18
FUT10	1.88	1.63	2.01	2.42	1.65	1.94	2.51	2.41
GAGE2A	3.6	1.45	2.7	1.78	3.53	1.95	1.37	1.13
CA11	1.7	2.15	2.2	1.69	1.28	1.95	1.67	1.63
DENND2A	2.49	2.32	2.5	2.74	1.61	1.95	2.09	1.95
SLAIN1	1.88	2.22	2.41	2.35	2.14	1.95	2.14	1.96
HOXB3	1.78	1.59	1.63	2.2	1.49	1.95	1.96	1.97
CDC14A	2.02	1.81	2.1	2.24	2.16	1.95	2.7	2.12
TAF5	1.41	1.22	1.71	1.81	1.88	1.95	2.14	2.37
ZNF138	1.58	1.2	1.74	2.07	1.9	1.95	2.85	2.49
RBM43	2.22	2.05	2.33	2.48	2.11	1.95	2.63	2.63
ARFGEF3	1.72	1.68	2.22	2.43	2	1.95	2.94	2.76
ZEB1	1.71	1.62	2.21	2.44	1.56	1.95	3.2	2.81
CLGN	2.25	2.67	2.42	3.03	2.44	1.95	3.88	2.86
DOPEY1	2	1.65	2.16	2.74	1.92	1.95	3.6	3.05
ZNF471	1.6	1.45	1.88	2.75	2.19	1.95	3.71	3.21
ZNF680	1.84	1.79	1.97	2.66	1.79	1.95	3.24	3.26
MOCS1	1.62	1.36	1.49	1.72	1.94	1.96	1.48	1.52
CXCL16	2.22	1.99	2.16	1.66	2.14	1.96	1.73	1.84
CDS1	1.88	1.72	1.85	2.56	2.05	1.96	2.81	2.41
CACNA2D1	1.51	1.63	1.79	2.92	2.15	1.96	3.13	2.66
SLC36A4	1.14	1.61	1.67	2.12	2.05	1.96	2.97	2.88
CCDC152	1.69	1.71	2.12	3.07	2.32	1.96	3.77	3.49

RHPN2	2.2	2.04	2.24	1.87	2.37	1.97	2.64	1.82
ARSG	1.54	1.44	1.42	1.63	1.64	1.97	1.5	1.88
ALG1L2	2.77	1.88	1.87	1.7	1.65	1.97	1.77	1.93
PCED1B	2.82	2.28	2.17	3.03	2.36	1.97	2.38	2.28
LSM8	1.75	1.66	1.74	1.96	1.89	1.97	2.76	2.48
ZNF141	1.67	1.78	1.51	2.34	1.69	1.97	2.95	2.61
C16orf52	1.68	1.62	1.91	2.9	2.41	1.97	3.75	3.1
LOC100652777	1.79	1.85	1.92	2.17	1.63	1.97	4.19	3.11
BICDL1	1.52	1.49	1.36	1.58	1.93	1.98	1.86	1.76
RPS6KL1	2.02	1.42	2.15	2.02	1.42	1.98	1.88	1.82
ZNF618	2.24	2.16	1.96	2.13	2.15	1.98	2.15	1.88
CACNG8	1.71	1.63	1.89	1.85	1.63	1.98	2.26	2.33
PCSK4	1.76	2.05	1.58	1.95	1.82	1.98	2.4	2.33
CISD2	1.72	1.74	1.85	2.06	2	1.98	3.1	2.44
PTEN	2.03	1.99	2.12	2.56	1.82	1.98	3.1	2.77
NOCT	2.05	2.04	1.98	1.73	1.98	1.99	1.36	1.71
SPATA25	1.81	1.61	1.68	2.13	1.44	1.99	2.25	1.77
PRSS53	2.21	1.89	1.93	2.04	2.44	1.99	2.11	1.84
TNRC6C	1.74	1.43	1.73	1.82	1.7	1.99	1.78	1.95
LINGO2	2.22	2.38	2.6	2.39	1.93	1.99	2.34	2.33
GDPD1	1.92	1.82	2.23	2.2	1.96	1.99	3.12	2.58
ADAMTS12	1.98	2.35	2.28	2.5	2.05	1.99	2.23	2.75
HIVEP2	2.28	1.98	2.46	2.57	2.04	1.99	3.3	2.81
BBS9	1.95	2.02	2.07	2.49	2.76	1.99	2.42	2.94
FAM161B	1.88	1.65	2.05	1.97	2.18	2	2	1.76
CHAC1	2.15	2.22	2.06	2.04	1.84	2	1.39	1.87
IBA57	2.02	1.88	2.06	1.75	1.92	2	2.23	1.93
NR4A1	1.84	1.69	2.24	1.88	2.46	2	1.91	1.95
EFEMP2	2.22	3.2	2.27	2.58	1.64	2	2.2	2.11
C7orf31	2.44	1.94	2.48	2.56	2.5	2	2.37	2.16
LIPT1	1.48	1.45	1.85	2.16	2.05	2	3.08	2.37
TPK1	1.84	1.58	1.98	2.13	2.22	2	2.84	2.43
IFT46	2.93	3.29	3.14	2.93	1.55	2.01	1.2	1.67
AMER1	1.85	1.93	2.07	1.8	1.75	2.01	2.23	2.13
STARD13	1.86	1.76	2.22	2.09	2.13	2.01	2.28	2.18
GTF2H5	2.12	2.02	2	2.39	1.76	2.01	2.6	2.44
FRMD3	1.8	1.74	2.26	2.01	2.5	2.01	3.1	2.56
AKAP12	2.7	2.13	2.89	2.95	2.02	2.01	3.15	2.56
PRR16	2.05	1.79	2.32	2.16	2.42	2.01	2.77	2.65
KIAA1551	1.65	1.36	1.9	2.32	1.91	2.01	3.8	3.16
BFSP1	2.14	2.05	2.02	2.1	1.98	2.02	1.83	2.13
RMDN2	1.99	1.74	1.4	2.48	1.66	2.02	3.07	2.19
NHSL1	2.16	2.16	2.11	2.38	2.08	2.02	2.37	2.22
CSMD1	1.93	1.88	2.03	2.27	2.07	2.02	2.1	2.3
ZNF430	1.4	1.35	1.97	2.45	1.67	2.02	3.77	3.06
LOC107985103	1.47	1.52	1.5	1.48	1.78	2.03	1.3	1.45
GDPD5	1.75	1.92	2.13	1.67	2.16	2.03	1.7	1.89
ERCC6	1.85	1.56	1.94	2.27	1.61	2.03	2.67	2.02
ESRP1	1.78	2.19	1.95	1.95	1.95	2.03	2.29	2.32
BLM	1.89	1.53	2.38	2.6	2.35	2.03	3.32	2.45
GPR155	2.03	1.87	2.39	2.92	1.97	2.03	3.27	2.63
FEM1C	1.8	1.59	2.12	2.28	2.14	2.03	3.04	2.88
LRRC8C	2	1.92	2.2	2.89	1.98	2.03	3.33	2.95
NOTCH2NL	1.98	2.4	2.18	2.27	2.35	2.04	1.91	2.05
SEMA6B	4.01	4.7	3.98	3.73	2.03	2.04	1.41	2.31
ARL6	1.81	2.18	2.1	2.09	2.03	2.04	3.24	2.41
LNP1	2.57	2.29	2.55	2	2.11	2.04	2.63	2.76
RASA2	1.96	1.77	2.36	2.48	2.34	2.04	3.99	2.89
PANK1	2.07	2.16	2.69	2.63	2.58	2.04	3.32	3.39
HIST2H2BE	2.09	2.65	2.21	2.16	1.69	2.05	1.7	1.61
CELF2	2.26	1.57	2.43	2.39	2.45	2.05	2.76	1.96
ARMCX5-GPRASP2	2.24	1.94	1.76	2.13	2.09	2.05	1.88	2.01
ZNF713	2.43	2.36	2.52	2.53	1.91	2.05	2.41	2.09
TRPV1	1.83	1.98	2.07	2.54	2.31	2.05	2.67	2.25
LOC102724843	2.81	2.61	2.52	3.47	1.87	2.05	2.31	2.56
ZKSCAN3	2.61	2.43	2.67	2.6	1.87	2.05	2.54	2.83
LOC101927345	2.75	1.67	2.97	4.06	3.71	2.05	5.98	4.53
AMIGO1	1.82	2	1.61	1.62	1.69	2.06	1.6	1.52
SNAI1	1.65	2.11	1.94	2.07	2.03	2.06	1.4	1.83
PTK2B	2.71	1.92	2.15	1.98	1.74	2.06	1.76	2.05
SLC2A13	1.46	1.51	1.18	1.65	1.62	2.06	2.49	2.1
CT55	2.38	1.67	1.66	1.38	1.81	2.06	1.83	2.26
TUBE1	2.12	1.88	2.25	2.45	2.17	2.06	3.47	2.49
ZBTB43	1.66	1.82	1.9	2.07	2.01	2.06	2.72	2.71
SPOPL	1.9	1.87	2.01	2.46	1.83	2.06	3.61	2.76
TNFRSF6B	2.45	1.94	2.45	2.55	2.27	2.06	2.73	3.12
ZSCAN20	1.62	1.95	1.92	1.9	1.91	2.07	2.07	2.09
PAEP	1.24	1.73	1.2	1.48	2.04	2.07	1.84	2.1
ASTE1	1.79	1.54	2.4	2.24	2.33	2.07	2.83	2.47
TNFAIP8	1.76	1.78	1.56	2.4	2.13	2.07	3.2	2.59
SLC22A15	1.64	1.75	1.88	2.17	2.21	2.07	3.35	2.62

KLHL2	2.26	2.2	2.03	2.24	2.44	2.07	2.34	2.83
CXorf23	1.95	1.79	2.16	2.24	1.75	2.07	3.47	3
FZD7	3.63	3.37	3.07	3.32	2.14	2.08	2.03	2.09
HSH2D	2.9	3.39	3.18	3.78	1.71	2.08	2.28	2.13
CTC1	1.91	2.05	1.98	1.83	2.04	2.08	2.03	2.16
C16orf86	2.19	2.04	1.81	1.54	1.92	2.08	2.06	2.21
PLEKHH1	1.95	1.73	2.2	2.07	1.8	2.08	2.65	2.27
PLK4	1.59	1.51	1.95	1.85	1.66	2.08	3.65	2.27
ZNF510	1.66	1.58	2.1	2.65	1.83	2.08	3.15	2.47
PROCA1	1.61	1.84	2.23	1.9	2.2	2.08	2.29	2.57
LOC150051	3.6	2.94	3.36	2.94	2.93	2.08	1.94	2.65
TDRP	1.89	2.13	2.03	1.99	2.26	2.08	2.74	2.69
NFKBID	1.43	1.57	1.79	1.93	2.1	2.08	2.22	2.78
ZNF776	2.03	1.94	2.23	2.75	2.2	2.08	3.62	2.83
KANSL1L	1.94	1.95	2.29	3.1	1.99	2.08	3.59	3
TEX9	2.43	2.2	2.52	2.23	1.93	2.08	3.99	3.17
DNAJC25	2.41	1.81	2.74	2.61	2.76	2.08	4.13	3.27
ZNF33A	1.75	1.71	1.89	2.34	1.82	2.08	4.18	3.42
CCDC186	1.68	1.55	1.93	2.36	1.86	2.08	4.27	4.19
DHRS12	2.28	2.65	2.28	2.01	2.12	2.09	2.12	1.73
MX1	5.38	5.76	5.13	5.92	2.15	2.09	2.29	1.92
ZNF530	2.16	1.84	2.06	1.91	2.79	2.09	2.82	1.98
JADE3	1.7	1.7	1.75	2.04	2.21	2.09	2.45	2.11
SMKR1	2.24	1.91	1.63	2.14	1.66	2.09	2.63	2.11
MXRA5	5.07	5	5.27	5.11	2.05	2.09	2.3	2.24
BTBD9	1.93	2.11	2.03	2.08	2.09	2.09	2.37	2.28
FAM46C	1.65	1.67	1.43	1.89	1.94	2.09	2.24	2.46
CENPE	1.19	1.28	1.55	1.37	2.18	2.09	3.45	2.8
KIAA0895	2.26	2.33	2.57	2.86	2.12	2.09	3.12	2.83
CIPC	2.08	1.87	2.16	2.57	2.28	2.09	3.48	2.93
DDIAS	1.88	1.56	2.02	2.33	2.58	2.09	3.6	3.14
FERMT2	2.1	1.97	2.61	2.87	2.07	2.09	3.41	3.16
VPS13C	1.45	1.51	1.86	2.46	1.84	2.09	4.09	3.54
HERC6	3.89	3.48	4.29	4.46	1.9	2.1	2.55	1.86
SMAGP	1.37	1.61	1.65	1.66	1.82	2.1	1.58	1.95
LOC107984156	1.26	1.7	1.76	1.85	1.67	2.1	2.79	2.1
CIDEB	1.78	1.55	1.63	2	1.9	2.1	2.78	2.21
ZNF816-ZNF321P	1.71	1.98	1.82	2.1	1.85	2.1	2.32	2.4
PLEKHA7	2.51	2.32	2.45	2.5	2.11	2.1	2.34	2.6
ZNF490	2.06	1.87	2.33	2.37	2.13	2.1	2.41	2.89
TNFSF12	1.78	1.66	1.86	1.76	1.89	2.11	1.1	1.33
TERT	1.86	1.8	1.9	1.98	2.03	2.11	1.83	1.83
STAR	1.6	1.66	1.64	2	1.77	2.11	1.67	2
DISC1	2.38	2.1	2.08	2.74	2.24	2.11	2.54	2.21
ZNF587B	2.08	1.71	1.97	1.95	2.13	2.11	2.39	2.54
LDB2	1.97	2.19	1.65	2.52	2.11	2.11	2.43	2.56
DOC2A	1.64	1.84	2.06	1.88	2.13	2.11	2.5	2.67
FZD4	2.64	2.52	2.47	2.62	2.47	2.11	2.69	2.69
FAM155A	2	1.7	2.1	2.26	2.36	2.11	2.86	2.71
CCDC18	1.48	1.58	1.55	2.03	1.5	2.11	3	2.74
PELI2	1.95	1.61	2.44	2.85	1.89	2.11	3.31	2.76
VAMP1	2.11	1.76	2.57	2.54	2.55	2.11	2.35	2.87
ATM	1.58	1.69	1.86	2.45	1.96	2.11	3.81	3
TDRD5	2.79	2.43	2.48	2.34	2.74	2.11	3.14	3.01
ADM5	1.83	1.48	1.92	1.73	2	2.12	2.35	1.51
HDAC4	2.25	2.31	2	2.12	2.04	2.12	1.91	1.95
SLC15A2	2.02	1.87	2.12	2.23	1.85	2.12	2.27	2.09
SFMBT1	1.85	1.94	2.09	2.18	2.43	2.12	2.51	2.27
GAB2	1.79	1.93	1.94	1.82	2.13	2.12	2.14	2.34
ZSWIM6	1.98	1.75	2.4	2.3	2.49	2.12	2.92	2.41
BEX1	1.31	1.55	1.63	1.37	2.02	2.12	2.61	2.62
FAM169A	1.62	1.38	1.85	2.26	1.83	2.12	3.23	2.63
FAM71E1	2.9	2.61	2.41	1.97	2.35	2.12	2.18	2.66
TTC30A	2.57	2.34	2.51	2.71	2.18	2.12	2.65	2.81
MMP13	3.76	3.9	3.96	4.08	2.36	2.12	3.74	2.87
NR2F1	2.45	2.08	2.03	2.74	1.86	2.12	1.73	2.97
ARID4A	1.81	1.9	2.06	2.78	2.11	2.12	3.91	3.31
CARF	2.03	1.44	2.1	2.1	2.05	2.12	3.42	3.33
GJC2	3.5	3.38	3.97	2.73	2.27	2.13	1.36	1.81
HOMER2	2.1	1.66	1.91	2	1.81	2.13	1.52	2.15
KLF12	1.27	2.02	2.07	2.26	1.68	2.13	2.93	2.44
MCMDC2	2.39	2.19	2.27	2.08	2.3	2.13	2.82	2.49
KAT6B	2.04	1.8	2.5	2.56	2.29	2.13	3.26	2.79
UHRF1BP1L	1.63	1.68	1.91	2.23	1.98	2.13	3.19	2.92
ZNF483	2.24	1.68	2.37	2.66	1.98	2.13	2.55	3.4
IFITM10	2.48	2.83	2.47	2.63	1.95	2.14	1.76	1.71
DLX2	1.88	2.03	1.86	1.47	2.69	2.14	1.77	1.76
MEX3B	2.87	2.35	2.72	2.74	2.14	2.14	1.85	1.94
ZBTB8A	2.03	2.02	2.06	2.33	1.97	2.14	2.47	2.4
FBXO33	1.84	1.58	2	1.96	2.1	2.14	2.55	2.4
ZNF383	1.71	1.48	1.9	2.23	2	2.14	2.71	2.5
ISL2	2.44	2.61	2.52	1.94	2.44	2.14	2.14	2.51
HSPA12A	1.82	1.98	1.85	2.24	2.27	2.14	2.6	2.51
TIGD7	1.93	1.51	2.58	2.6	2.59	2.14	4.47	2.68
AASDH	1.94	1.91	2.49	2.68	2.35	2.14	2.81	2.81

ZNF429	2.12	1.89	2.33	2.72	1.86	2.14	3.43	2.85
TMEM171	1.71	2.51	2.49	1.54	1.47	2.15	1.55	1.4
PLEKHG4B	1.34	1.85	1.95	1.68	1.8	2.15	1.53	1.87
ARHGEF37	2.47	2.38	2.95	2.54	1.84	2.15	2.01	2.03
HNRNPH2	1.08	1.56	2.56	2.31	2.84	2.15	2.95	2.09
ZBTB39	2.4	2.17	2.41	2.24	2.12	2.15	1.93	2.1
GAREM1	2.4	1.91	2.57	2.9	1.96	2.15	2.71	2.26
RAB17	2.11	2.09	2.4	2.35	3.09	2.15	3.02	2.38
ZNF93	2.08	2.02	2.63	3.21	2.6	2.15	3.63	3.74
NOXO1	1.68	2.14	2.34	1.54	2.04	2.16	1.56	1.92
C19orf71	2.2	2.58	1.82	1.86	1.77	2.16	2.69	2.16
TBC1D12	2.17	1.79	1.7	2.17	1.63	2.16	2.12	2.17
ATXN1	2.04	1.99	2.25	2.49	2.02	2.16	2.59	2.64
NAP1L3	1.98	2.15	2.37	2.4	2.3	2.16	2.92	2.82
LOC100129940	2.31	1.91	2.31	2.62	2.71	2.16	3.13	3.08
TVP23C-CDRT4	1.39	1.74	1.96	2.33	2	2.17	2.51	1.58
SNX25	2.29	1.84	2.27	2.14	2.21	2.17	2.65	2.55
BORA	1.39	1.39	2.11	1.87	2.12	2.17	3.22	2.72
ABHD18	1.73	1.68	2.03	2.05	2.49	2.17	3.49	2.73
EXOC6	2.08	2.21	2.04	2.44	2.44	2.17	3.73	2.74
AUTS2	2.37	2.33	2.29	2.51	2.48	2.17	2.62	3.01
GALNT5	2.26	2.1	2.49	2.9	2.48	2.17	2.99	3.17
SALL4	2.26	2.08	2.3	3.16	1.99	2.17	3.69	3.21
BAZ2B	1.8	1.87	2.04	2.84	2.06	2.17	3.28	3.36
44263	2.36	2.24	1.87	2.13	2.07	2.18	2.44	2.36
PPP1R3C	2.52	2.61	2.61	2.71	2.39	2.18	2.87	2.61
SLCO1A2	2.38	2	2.38	2.42	1.72	2.18	2.59	2.68
PPP2R3A	2.11	1.73	2.23	2.83	2.21	2.18	3.81	3.22
FAM214A	1.91	1.86	2.33	2.52	2.25	2.18	3.73	3.31
PTGR1	2.79	3.23	3.59	3.53	2.58	2.18	2.37	3.46
NCAM2	2.28	2.41	2.28	3.24	2.79	2.18	3.65	3.49
NLRC5	2.3	2.19	2.41	2.98	2.28	2.19	2.1	1.85
ZBTB42	2.82	3.22	2.41	2.59	2.38	2.19	1.85	2.25
RGS16	2.05	1.85	2.03	1.52	2.61	2.19	2.54	2.47
PLEKHM3	2.21	2.06	2.31	2.31	2.52	2.19	2.99	2.71
UBN2	1.66	1.66	1.97	2.68	1.86	2.19	3.34	2.96
PTX3	2.25	2.31	2.31	2.18	2.09	2.2	2.52	2.42
BORCS8-MEF2B	2.55	3.01	2.41	3.05	2.45	2.2	2.22	2.6
PDK4	2.49	2.21	3.2	3.47	1.78	2.2	3.19	2.71
CSPP1	1.93	1.99	2.35	2.72	2.07	2.2	3.91	3.47
LOC101930090	1.45	1.71	1.36	1.7	1.68	2.21	1.25	2.06
GLIPR2	3.16	3.14	2.57	2.75	2.63	2.21	2.12	2.06
DZANK1	1.96	1.8	1.97	2.24	1.76	2.21	2.67	2.16
PITPNC1	2.09	2.46	2.37	2.2	2.13	2.29	2.21	2.04
AGPAT4	1.87	1.9	2.2	2.5	2.28	2.21	2.83	2.48
ADAT2	1.85	1.9	2.46	2.01	2.39	2.21	3.05	2.5
RASSF3	1.8	1.98	1.78	2.15	2.35	2.21	2.49	2.63
LMO7	1.73	1.51	1.76	2.16	2.11	2.21	3.43	2.65
ELAC1	3.22	2.74	2.98	3.45	2.43	2.21	2.61	2.74
C3orf18	2.01	1.85	2.09	1.94	2.13	2.22	1.49	1.94
PPFIA3	1.64	1.95	1.48	1.6	2.05	2.22	1.46	1.97
SLC35G1	1.99	2.02	1.98	2.2	2.23	2.22	3.3	2.36
IRGQ	1.77	1.99	2.01	2.59	2.17	2.22	2.58	2.5
SLC22A4	2.15	2.45	2.7	2.05	2.56	2.22	2.64	2.57
PCDHB16	3.08	2.53	2.48	2.81	2.18	2.22	2.95	2.88
NBPF20	1.66	1.71	2.07	2.32	2.4	2.22	3	2.96
TBC1D8B	1.93	1.82	2.54	2.37	1.64	2.22	3.4	3.14
LOC644249	1.59	1.03	1.21	1.91	1.19	2.23	3.25	2.04
FRMD5	2.1	2.02	2.75	2.96	2.8	2.23	3.53	2.71
TIFA	2.69	2.63	2.46	3.07	2.53	2.23	3.36	3.14
TIGD6	2.14	2.11	2.28	2.39	2.5	2.24	2.5	2.56
PROS1	2.08	2.15	2.29	2.58	2.32	2.24	3.32	2.62
AKNA	2.49	2.74	2.45	2.52	2.46	2.24	2.25	2.63
MAP3K9	2.32	2.25	2.47	2.72	2.64	2.24	2.6	2.69
MITF	1.88	2.02	1.85	2.65	2.46	2.24	3.23	2.73
BORCS7	2.02	1.84	2.15	3.08	1.97	2.24	3.82	2.89
EEA1	1.63	1.5	1.91	2.01	1.89	2.24	3.6	3.04
SERTAD4	3.1	2.98	3.1	3.46	3.1	2.24	2.73	3.1
SC5D	1.49	1.81	1.73	2.29	2.21	2.24	3.74	3.1
SECISBP2L	1.99	1.76	2.27	2.62	2.07	2.24	3.41	3.29
REST	2.26	2.05	2.61	2.98	2.39	2.24	3.75	3.45
LEPR	2.02	2.14	2.14	2.54	2.13	2.24	4.08	3.46
SKIL	2.15	2.23	2.59	3.1	2.16	2.24	4.09	3.62
LIFR	2.11	1.89	2.53	3.07	2.51	2.24	4.5	3.66
SLC52A1	1.95	1.82	1.62	2.15	1.79	2.25	1.89	1.59
ZNF552	2.15	2.32	2.52	1.96	2.45	2.25	2.89	2.36
FAM171B	2.22	1.71	2.15	2.63	2.04	2.25	3.36	2.43
NNAT	2.4	2.64	2.66	2.55	2.65	2.25	3.26	2.51
STRIP2	1.58	1.41	1.97	2.27	2.06	2.25	3.02	2.64
FKBP7	2.26	2.01	2.21	2.33	2.04	2.25	3.26	2.85

ZNF23	2.22	1.58	2.23	2.63	2.25	2.25	3.94	2.97
RNF219	2.44	2.08	2.73	3.55	2.61	2.25	3.7	3.62
C10orf95	2.25	2.16	2.35	2.43	2.02	2.26	2.31	2.05
UBASH3B	1.64	1.73	1.78	1.9	2.38	2.26	2.54	2.39
APOBEC3D	2.31	2.31	2.03	2.48	2.08	2.26	2.92	2.41
OAS2	6.88	7.72	7.15	7.42	2.4	2.26	3.13	2.79
LOC102724250	1.79	1.88	2.07	2.5	2.08	2.26	3.55	2.8
ATG4A	2.4	1.68	2.38	2.44	2.95	2.26	3.02	3.09
ERCC6L	2.21	1.73	2.78	2.68	3.01	2.26	4.01	3.23
CCDC84	1.77	1.58	2.09	2.11	2.52	2.27	3.44	2.65
SAPCD1	1.1	1.31	2.02	1.52	1.74	2.27	3.27	2.77
SLC19A2	2.22	2.11	2.26	2.67	2.65	2.27	3.46	2.92
SOX6	2.71	2.54	2.72	3.17	2.44	2.27	2.83	2.94
LRP1B	1.56	1.32	1.9	2.11	2.4	2.27	3.49	3.08
IKZF5	2.11	1.95	1.95	2.8	2.19	2.27	3.92	3.3
SASS6	1.92	1.47	2.06	2.75	2.11	2.27	4.46	3.56
ANKRD36	1.87	2.29	2.22	3.11	2.53	2.27	5.42	4.01
IL17D	1.35	1.62	1.84	2.14	1.9	2.28	2.26	1.85
PDGFB	3.26	3.03	3.67	3.73	2.02	2.28	2.3	2.05
SOCS1	2.12	2.61	2.45	1.83	2.53	2.28	2.17	2.06
CDK20	2.38	2.3	1.96	2.66	2.91	2.28	1.98	2.16
BTN3A3	2.19	2.47	2.41	2.29	2.06	2.28	2.6	2.22
UCP2	2.27	3.38	2.48	2.41	2.7	2.28	1.81	2.39
PDE4B	2.08	2.03	1.66	2.12	2.4	2.28	3.02	2.51
ZRANB3	2.25	2.11	1.98	2.44	2.44	2.28	3.21	2.56
C4orf33	2.52	2.92	3.04	2.76	2.38	2.28	3.71	2.71
RAB23	2.12	1.97	2.34	2.48	2.3	2.28	3.05	2.74
CNOT6L	2.09	1.94	2.51	2.76	2.36	2.28	3.06	3.02
ZNF708	1.72	2.25	2.37	2.44	1.91	2.28	4.46	3.17
AFF1	2.38	1.93	2.41	3.48	2.37	2.28	3.19	3.25
PIGA	2.65	2.25	2.69	3.51	2.66	2.28	4.31	3.69
LIPT2	2.61	2.31	1.76	1.84	1.55	2.29	1.53	1.63
BEND3	2.09	1.92	2.01	2.21	2.09	2.29	1.87	2
WDFY3	2.04	2.13	2.62	2.77	2.14	2.29	2.98	2.58
CYB5D1	2.42	2.35	2.7	2.7	3.02	2.29	2.76	3.09
ATP8B1	3.13	2.93	3.56	4.03	2.21	2.29	3.27	3.1
C12orf66	2.55	2.34	2.74	2.42	2.71	2.29	2.95	3.21
NRL	2.48	2.41	1.99	1.64	1.87	2.3	1.84	1.51
ANXA9	2.4	2.19	1.74	1.74	1.84	2.3	1.93	2.23
CDKN1C	5.1	4.79	4.42	4.57	2.48	2.3	2.07	2.49
NPL	2.46	2.39	2.28	2.76	2.01	2.3	3.21	2.57
MFSD9	2.28	2.14	2.46	2.42	2.64	2.3	2.79	2.58
DNMBP	2.85	2.71	2.82	3.11	2.62	2.3	2.97	2.73
ZNF101	2.81	2.63	2.84	3.12	2.99	2.3	2.89	2.91
PTBP2	2.08	1.88	2.26	2.18	2.22	2.3	4.41	3.21
MAGIX	1.78	1.89	1.55	1.55	2.22	2.31	1.59	1.69
EPHB3	2.37	2.5	1.83	2.15	2.13	2.31	1.82	2.23
SCLT1	1.54	1.58	1.52	2.1	1.91	2.31	1.99	2.3
ALPK1	1.83	2.02	2.15	2.26	2.07	2.31	2.86	2.53
CAP2	2.39	2.61	2.58	3.17	2.49	2.31	2.73	2.62
CLEC2B	2.12	2.43	2.51	2.49	3.01	2.31	3.54	2.83
RAB8B	2.28	2.43	2.35	3.2	2.61	2.31	3.7	3.11
FRAS1	2.87	2.82	2.7	3.1	2.46	2.32	2.62	2.56
C3orf67	2.2	1.78	2.2	2.05	1.81	2.32	2.56	2.58
ATG4C	1.96	1.79	2.4	2.68	2.47	2.32	4.2	2.68
MAGEE1	3.02	3.34	3.1	2.86	2.54	2.32	3.01	2.93
ZMYM5	1.88	1.49	1.88	2.71	1.79	2.32	3.4	3.07
ZNF107	1.88	1.74	1.98	2.54	2.05	2.32	4.55	3.39
PHACTR2	2	1.83	2.21	2.81	2.53	2.32	4.56	4
IZUMO4	2.38	1.76	2.59	2.26	1.97	2.33	2.61	1.72
DPF1	2.51	2.58	1.89	2.27	1.97	2.33	1.51	1.9
ZNF250	2.69	3.19	3.62	3.09	2.76	2.33	2.7	2.25
BAHCC1	2.54	2.47	2.62	2.47	2.15	2.33	1.95	2.35
ANK1	2.3	2.34	2.11	2.18	2.59	2.33	2.54	2.59
TLCD2	2.16	2.4	2.52	2.76	2.34	2.33	2.47	2.65
MMP16	1.71	2.06	1.83	2.27	1.89	2.33	3.32	3.05
UBL3	1.86	1.99	2.15	2.56	2.77	2.33	3.55	3.14
TTC30B	2.56	2.74	3.3	2.94	2.25	2.33	3.23	3.51
TMEM267	2.34	2.53	2.26	2.71	2.48	2.33	4.14	3.77
CPEB2	2.92	3.14	3.37	3.91	2.84	2.33	3.76	3.93
CCDC61	2.44	2.13	2.07	1.93	1.93	2.34	1.62	1.96
PPP1R1B	2.95	4.18	2.71	2.88	1.7	2.34	1.65	2.25
CARD8	1.63	2.42	2.43	2.31	2.5	2.34	3.65	2.53
DLG4	2.65	2.44	2.7	2.73	1.91	2.34	2.21	2.54
VLDLR	3.72	3.61	4.27	3.63	1.97	2.34	2.78	2.55
WWC1	1.87	2.08	2.1	1.9	2.48	2.34	2.07	2.7
SHPRH	1.99	1.83	2.1	3.01	2.3	2.34	4.27	3.67
CTSV	2.35	1.96	2.51	2.6	2.49	2.35	2.43	2.55
RHOBTB1	1.89	2.31	2.78	2.7	2.41	2.35	2.52	2.67
ZNF227	2.18	1.97	2.73	2.61	2.64	2.35	3.45	2.87
PRRT2	2.39	2.08	2.31	2.47	2.11	2.35	3.61	3.19
TTC33	2.19	1.92	2.55	2.7	2.66	2.35	3.72	3.27
WDR35	1.79	1.36	2.56	2.39	2.36	2.35	3.87	3.43
ZBTB24	2.33	3.19	2.6	3.05	2.55	2.35	3.7	3.64
ZC4H2	2.04	2.49	2.18	2.2	2.21	2.36	1.89	2.08
IL11	2.01	2.18	1.52	1.9	2.83	2.36	2.49	2.64
MED14OS	3.46	2.7	2.47	3.53	2.79	2.36	2.39	2.72

ZNF440	2.22	1.84	2.63	2.89	2.51	2.36	4.09	2.89
PCDH20	3.48	3.08	3.41	3.34	2.12	2.36	3.14	3
ZNF773	2.78	2.65	2.74	3.58	2.73	2.36	3.18	3.15
TMEM67	2.2	1.8	2.47	2.61	2.2	2.36	3.55	3.3
CD302	2.46	2.19	2.41	3.56	2.9	2.36	4.46	3.51
SGTB	1.92	1.78	2.31	2.63	2.49	2.36	4.27	3.75
ZNF550	1.96	1.78	2.49	2.53	2.5	2.36	4.87	3.8
MOB1B	2.15	2.19	2.74	2.92	2.12	2.36	4.34	3.94
LOC100996842	2.32	2.69	2.8	2.23	2.58	2.37	1.74	1.66
LOC107985388	2.4	1.9	2.29	1.91	2.05	2.37	2.14	2.04
ATP6V0A2	1.83	1.82	2.24	2.31	2.35	2.37	2.69	2.56
C4orf46	2.19	1.99	2.08	2.52	2.17	2.37	3.14	2.64
DHFRL1	2.22	1.98	2.21	2.46	2.88	2.37	3.5	2.74
PER2	2.4	2.51	2.75	2.66	2.34	2.37	2.71	2.94
SPOCK3	3.3	3.34	2.98	4.06	2.55	2.37	3.05	3.21
ANGPT1	2.04	2.08	2.61	2.48	2.1	2.37	4.14	3.21
FOXO4	2.45	3.09	2.75	2.51	2.89	2.37	2.46	3.33
MYO9A	1.86	1.69	2.28	2.84	2.37	2.37	3.52	3.36
S100A4	3.22	3.04	2.43	2.38	2.98	2.37	3.91	3.51
ADRB2	2.12	2.24	2.07	1.95	2.2	2.38	2.41	2.58
SLX4IP	2.05	1.95	2.2	3.24	2.6	2.38	3.57	3.13
PCLO	2.22	2.01	2.66	2.87	2.27	2.38	3.61	3.31
ATG2B	2.36	1.9	2.41	2.77	2.54	2.38	4.19	3.58
KIAA1715	2.24	2.13	2.7	3.15	2.73	2.38	4.08	3.64
SP4	2.46	2.09	2.87	3.99	2.82	2.38	4.15	3.82
C2orf69	1.88	1.97	2.4	2.52	2.36	2.38	3.74	3.89
GDPGP1	2.67	2.34	2.19	1.95	2.06	2.39	2.04	2.21
ZNF57	2.01	2.08	2.32	2.44	2.53	2.39	2.82	2.5
PANO1	1.66	1.83	1.77	1.76	1.96	2.39	2.16	2.56
SIX4	2.4	1.89	2.37	2.93	2.03	2.39	3.09	2.81
CEP295	1.81	1.64	2.19	2.58	2.17	2.39	4.05	3.46
ZNF493	2.18	1.95	2.09	2.73	2.12	2.39	4.71	3.51
THAP6	2.27	1.83	2.27	2.86	2.43	2.39	4.23	3.83
LYPD1	2.46	3	3.3	2.67	2.69	2.4	2.09	1.63
SPDYE17	2.45	2.28	2.25	3.1	2.97	2.4	3.21	2.58
ZNF699	2.53	2.18	2.76	2.85	2.94	2.4	4.14	3.24
ANKRD26	1.65	2.03	2.11	2.59	2.39	2.4	4.04	3.27
ZNF268	2.23	2.06	2.69	2.74	2.6	2.4	4.14	3.49
ANKRD46	2.68	2.99	3.26	3.51	2.88	2.4	3.92	3.71
CNIH2	2.37	2.19	2.63	2.16	1.89	2.41	2.06	1.99
PDLIM3	2.28	2.45	2.38	2.37	2.38	2.41	2.06	2.35
ALX3	1.97	2.14	2.24	2.49	2.48	2.41	2.62	2.66
KATNAL1	2.15	1.82	2.36	2.68	2.46	2.41	3.25	3.09
STAMBPL1	3.29	2.23	3.5	3.64	2.07	2.41	3.81	3.15
MAP9	2.1	2.17	2.13	2.79	2.46	2.41	3.34	3.25
ZNF507	1.83	1.74	2.49	3.28	2.74	2.41	4.9	3.83
APC	2.11	2	2.59	3.29	2.57	2.41	5.05	4.17
FAM132B	2.02	2.09	1.92	1.92	2.3	2.42	1.54	1.64
TYMP	2.47	3.09	3.07	2.44	2.48	2.42	1.68	1.92
ANKDD1A	1.8	1.9	2.03	2.12	1.88	2.42	2.57	2.15
MAPK8IP2	1.72	1.64	1.63	1.52	3.06	2.42	2.39	2.33
FAM76A	1.81	1.78	2.52	2.42	2.35	2.42	2.95	2.62
TIRAP	2.01	2.24	2.14	2.18	2.27	2.42	2.17	2.98
ITPR1	2.46	2.14	2.6	2.87	2.59	2.42	2.9	3.01
SYNC	1.52	2.21	1.8	2.13	2.79	2.42	2.58	3.18
ARID2	2.8	2.26	3.06	3.81	2.74	2.42	3.9	3.39
DUSP28	2.05	2.05	1.66	2.41	1.73	2.43	1.6	2.09
NXPH4	4.95	4.42	4.36	3.94	1.99	2.43	1.85	2.38
TXNDC16	1.98	1.7	2.27	2.75	2.27	2.43	3.63	2.65
RAPH1	2.08	1.84	2.14	2.5	2.6	2.43	3.1	2.71
CRIP1	2.71	3.04	2.17	3.1	2.87	2.43	1.7	2.96
POLE2	2.97	3.19	3.19	3.83	2.38	2.43	5.09	3.08
VCPKMT	1.95	1.85	2.76	2.63	2.8	2.43	4.69	3.21
ZNF34	2.57	2.48	2.35	2.81	2.32	2.43	2.92	3.43
GK5	2.12	2.01	2.29	2.73	2.29	2.43	3.93	3.47
HSD3B7	2.64	2.62	2.92	1.8	1.83	2.44	2.18	2.04
ITPKA	2.78	2.59	3.26	2.95	2.27	2.44	2.28	2.43
OTUD1	2.8	2.55	2.48	2.91	2.66	2.44	2.83	2.6
CHMP4C	2.26	2.07	2.49	2.42	2.64	2.44	3.28	2.63
ARHGEF28	2.63	2.7	3.15	3.13	3.17	2.44	3.72	2.91
RHBDL2	2.23	2.57	2.3	3	2.91	2.44	3.42	3.01
EIF5A2	1.95	1.72	2.33	2.56	2.41	2.44	3.43	3.23
BRIP1	2.2	2.01	2.76	3.44	2.56	2.44	4.31	3.5
LANCL2	2.08	2.42	2.21	2.16	1.9	2.45	2	2.03
SLC9B2	1.83	1.79	2.19	2.05	2.36	2.45	2.99	2.45
TOX3	2.41	1.88	2.54	2.82	2.42	2.45	3.1	2.94
C12orf76	2.71	3.17	2.8	3.58	3.22	2.45	3.15	3.14
RELL1	2.19	2.41	2.39	2.92	2.71	2.45	3.06	3.7
FICD	1.85	1.79	2.26	2.56	1.94	2.46	1.87	1.88
CENPP	2.2	2.06	2.45	2.04	2.88	2.46	2.62	2.12
FIBCD1	1.78	1.97	1.94	2.07	2.36	2.46	1.96	2.36
ACVR2B	2.06	2.01	2.42	2.85	2.44	2.46	2.92	2.82
S1PR4	1.22	1.62	1.37	1.81	2.3	2.46	2.84	2.85
SH3YL1	1.51	1.14	1.53	1.75	2.13	2.46	3.21	2.92

RAD54B	2.34	2.18	2.46	2.49	2.56	2.46	3.96	3.17
COLGALT2	3.51	3.73	3.72	3.73	2.4	2.46	2.56	3.18
ZNF320	2.27	2.01	2.55	3.08	2.33	2.46	3.76	3.18
NEIL3	2.44	2.15	2.82	2.76	3.22	2.46	3.59	3.24
CASP8AP2	1.97	1.87	2.58	3.07	2.27	2.46	4.82	3.68
GBX2	2.44	2.34	2.33	2.62	2.27	2.47	1.79	1.85
TRPV4	2.75	2.94	2.9	2.79	1.92	2.47	1.36	1.89
PCDHA3	2.19	1.63	2.37	1.87	2.34	2.47	1.95	2.28
RGL4	2.94	3.18	3.21	2.97	3.12	2.47	4.02	3.13
FGF1	1.86	1.72	2.4	1.87	2.42	2.47	2.97	3.23
LAMTOR3	2.27	2.21	2.98	2.83	2.92	2.47	3.84	3.45
FAXC	2.38	2.18	2.08	1.9	2.26	2.48	2.77	1.58
C14orf169	2.08	2.61	2.34	1.97	2.43	2.48	2.52	2.31
TTC12	2.31	2.34	2.32	2.27	2.18	2.48	2.28	2.38
TLE4	3.1	3.3	2.84	3.29	2.56	2.48	3.05	2.75
RAB40A	2.2	1.42	2.41	2.35	2.4	2.48	2.75	2.78
ZNF197	2.43	2.39	2.72	3.42	2.24	2.48	3.42	3.04
CEP290	1.88	2.03	2.07	2.41	2.41	2.48	4.12	3.46
DEPDC7	2.46	2.22	2.74	2.66	2.84	2.48	3.96	3.58
ECM1	1.88	1.7	1.69	1.64	2.09	2.49	1.58	1.91
HOXA4	3.27	2.96	2.62	2.73	2.83	2.49	1.99	1.91
KCNH3	2.05	2.36	2.15	1.84	2.43	2.49	2.07	2.27
MRAS	2.19	2.26	2.57	2.63	2.25	2.49	2.2	2.52
SMIM1	3.22	3.81	3.52	3.29	1.69	2.49	1.94	2.77
CRISPLD1	2.79	2.15	2.58	2.95	2.2	2.49	3	2.95
RPGRIP1L	1.96	2.04	2.11	2.46	2.3	2.49	3.46	3.25
CEP19	2.55	2.14	2.69	2.81	2.54	2.49	3.23	3.39
SLF1	2.28	2.45	2.74	3.22	2.34	2.49	4.95	3.47
MTERF2	2.14	2.34	2.28	3.25	2.78	2.49	3.8	3.5
CA5B	2.3	2.29	2.36	2.58	2.52	2.49	2.83	3.52
GULP1	2.19	3.28	2.98	3.41	2.63	2.49	3.95	3.57
SEMA4G	2.29	2.64	2.25	2.48	2.27	2.5	2.14	2.47
APOM	3.01	3.06	3.21	3.01	2.92	2.5	2.69	2.7
ZNF586	2.45	2.6	3.21	2.61	2.93	2.5	3.41	2.74
SAMD14	3.34	3.49	3.37	3.02	2.66	2.5	2.33	2.77
ESCO2	1.8	1.51	2.45	3.42	2.08	2.5	4.78	2.77
ZXDB	2.45	2.62	2.64	2.5	2.55	2.5	2.7	2.79
RFXAP	2.41	2.04	2.88	3.19	2.25	2.5	3.89	2.92
ALG9	2.28	2.49	3.11	3.05	2.73	2.5	2.79	2.96
USP49	2.35	2	2.63	3.23	2.58	2.5	3.92	3.29
TSPAN12	2.52	2.63	2.55	3.07	2.65	2.5	3.53	3.43
MANEA	2.55	2.91	3.07	3.61	2.55	2.5	4.5	3.48
DDX60	4.34	4	4.85	6.01	2.44	2.5	4.96	3.81
KIN	3.28	3.29	2.93	3.64	3.13	2.5	3.97	4
TNFSF10	3.86	4.07	4.92	6.1	3.35	2.5	4.57	4.87
SELM	3.28	3.42	2.67	2.17	1.95	2.51	1.29	1.29
CHRNB1	2.67	3.29	2.93	2.7	2.36	2.51	2.88	2.23
ZSWIM3	2.92	2.63	2.89	2.74	2.45	2.51	2.14	2.41
NFIA	2.16	1.94	2.33	2.94	2.4	2.51	2.88	2.83
TSTD3	2.62	1.88	1.96	2.64	2.42	2.51	3.05	2.99
UNC5D	3.42	3	3.73	4	2.3	2.51	3.12	3.02
ATP7A	2.22	2.09	2.71	3.19	2.46	2.51	4.11	3.47
HEATR5A	2.38	1.89	2.2	2.77	2.38	2.51	4.02	3.48
LOC107984799	2.45	2.57	1.93	2.45	2.33	2.52	1.72	2.27
CCDC120	3.03	2.82	2.64	2.84	2.7	2.52	2.39	2.32
RRAGD	1.74	1.44	2.2	1.91	2.3	2.52	2.54	2.44
C11orf71	3.47	2.79	3.16	3.04	2.08	2.52	2.2	2.8
TAF4B	1.97	1.73	2.24	2.6	2.77	2.52	2.99	2.93
TCHP	2.63	2.28	2.42	2.8	2.55	2.52	3.04	2.98
WDR7	2.63	2.44	2.73	2.91	2.85	2.52	3.22	3.07
NMU	2.89	2.07	2.68	2.43	3.67	2.52	2.9	3.2
DCC	1.73	2.49	2.18	2.5	2.31	2.52	3.19	3.23
FAM102B	1.8	1.72	2.33	2.94	2.68	2.52	4.14	3.49
MIS18BP1	2.35	2.2	2.82	2.59	2.94	2.52	4.93	3.81
STEAP2	1.83	1.94	1.96	2.91	2.31	2.52	3.74	3.84
JMY	2.13	2.16	3.06	3.57	2.77	2.52	4.72	3.97
CGB7	1.91	2.21	1.83	2.18	3.09	2.53	3.1	2.22
PIK3R3	2.47	2.67	2.52	2.59	2.81	2.53	2.85	3.15
GRIK2	1.99	2.39	2.48	3.06	2.26	2.53	3.63	3.26
DNA2	2.22	1.96	2.69	3	2.7	2.53	4.74	3.28
CREBRF	2.33	1.89	1.96	2.73	2.43	2.53	3.63	3.39
FAAH2	1.86	1.96	2.28	3.55	2.17	2.53	4.48	3.58
ZNF431	2.09	1.98	2.13	3.03	2.47	2.53	4.74	4.48
KLF15	1.4	1.57	1.78	2.16	3.14	2.54	2.11	1.77
DUSP1	3.81	3.46	2.83	2.87	2.44	2.54	2.51	2.04
44440	2.99	3.11	3.53	2.62	2.68	2.54	2.3	2.18
CAMK2B	4.1	4.44	4.01	4.11	2.01	2.54	2.27	2.32
FOLR1	2.54	2.76	1.92	2.92	1.96	2.54	1.14	2.68
MAGEH1	3.42	3.62	3.52	3.19	2.5	2.54	2.92	3.42
NFKBIZ	2.91	2.49	3.14	3.73	2.89	2.54	4.17	3.7
ZNF84	2.6	2.32	3.19	3.79	2.54	2.54	5.42	4.29
FAM135A	2.24	2.1	2.68	3.03	2.4	2.54	5.31	4.55
CFB	4.82	4.15	4.89	4.22	2.47	2.55	2.52	1.9
MYRF	2.87	3.03	2.83	2.7	2.53	2.55	2.33	2.62
ERN1	2.76	2.42	2.43	2.33	2.42	2.55	2.51	2.76

CENPJ	2.31	2.04	2.25	3.07	2.32	2.55	4.31	3.46
TGDS	2.71	2.4	3.45	3.73	3.15	2.55	4.4	4.38
RWDD2A	2.06	2.05	2.29	2.49	2.45	2.56	2.84	2.37
APOD	3.55	4.85	4.63	4.82	2.51	2.56	2.26	2.46
HOXA6	2.7	1.95	3.02	2.31	2.87	2.56	2.36	2.64
GRTP1	2.74	2.69	2.76	2.9	2.2	2.56	2.76	2.65
GYG2	2.8	2.39	2.41	2.71	2.8	2.56	2.51	2.72
KDELC2	2.55	2.13	2.42	3.78	2.35	2.56	3.31	2.88
PRDM15	2.03	2.12	2.27	2.47	2.99	2.56	2.96	2.95
SCAPER	2.02	1.83	2.29	2.74	2.68	2.56	3.61	3.38
RTN4R	2.9	3.59	3.01	2.01	2.28	2.57	2.1	2.12
TMC4	3.08	2.86	2.82	3.2	3.03	2.57	2.37	2.36
MAPK13	2.09	2.09	2.08	2.12	2.51	2.57	2.57	2.42
GPC2	2.27	2.43	2.39	2.92	2.83	2.57	2.92	2.67
SALL1	2.87	2.76	2.5	2.98	2.36	2.57	2.51	3.1
CDKN2AIP	2.43	2.58	2.67	2.87	2.75	2.57	3.54	3.16
TMCC1	2.6	2.53	2.87	3.12	2.53	2.57	3.34	3.25
ZNF654	2.36	2.32	2.38	3.03	2.39	2.57	4.06	3.57
FAM86B2	3.03	3.92	2.82	4.16	3.41	2.57	3.09	3.62
CEP350	2.26	2.01	2.87	3.7	2.64	2.57	5.43	4.21
BIK	1.71	1.86	1.29	1.9	2.21	2.58	2.22	1.79
TMEM145	2.6	3.06	2.85	2.63	2.14	2.58	2.81	1.9
OGDHL	1.39	1.56	1.6	1.53	2.41	2.58	2.41	2.52
JAZF1	1.94	2.11	1.96	2.08	2.37	2.58	3.3	3.01
USP38	2.41	2.45	2.58	3.22	2.65	2.58	3.35	3.48
NFXL1	2.39	2.09	2.73	3.41	2.93	2.58	4.56	3.68
ALMS1	2.14	1.95	2.64	3.19	2.64	2.58	4.22	3.76
ERBB2	2.48	2.59	2.36	2.12	2.18	2.59	2.32	2.05
B3GALT4	3.52	3.29	3.55	3.42	1.76	2.59	2.05	2.42
TENM4	2.9	2.97	2.84	3.05	2.38	2.59	2.57	2.66
DNAL1	2.19	2.18	2.26	2.69	2.48	2.59	2.98	2.75
C11orf57	2.28	2.24	1.92	2.44	2.5	2.59	3.01	2.85
AADAT	2	1.96	2.4	2.65	2.17	2.59	3.71	3.07
MND1	2.44	3.68	2.86	3.58	3.18	2.59	3.32	3.12
MBLAC2	2.76	2.72	2.68	3.22	2.81	2.59	3.74	3.12
LRRC37B	3	3.03	2.9	3.56	3.11	2.59	3.95	3.12
TRPC1	2.14	2.18	2.18	3.16	2.39	2.59	4.44	3.76
HOXA1	2.69	2.89	2.36	2.63	2.85	2.6	2.51	2.3
LOXL4	3.08	3.03	2.69	2.46	2.72	2.6	2.36	2.46
ZBTB14	2.55	2.07	3.08	2.83	2.64	2.6	3.35	2.73
XYLB	2.39	2.75	2.76	2.8	2.63	2.6	3.58	3.07
BCDIN3D	2.47	3	3.02	2.82	3.25	2.6	3.39	3.37
TRIP11	2.34	2.11	2.95	3.32	2.5	2.6	4.96	3.98
FDX1	2.26	2.26	2.48	2.22	2.73	2.61	2.71	2.65
ZNF512	2.67	2.52	3.06	2.96	2.61	2.61	3	3
RNF43	3.22	3.17	3.24	3.28	2.73	2.61	2.46	3.22
ICK	2.64	2.57	3.18	4.09	2.94	2.61	4.49	3.37
DENND2C	2.67	2.2	2.91	3.32	3.12	2.61	4.31	3.43
DIAPH3	2.44	2.04	3.08	3.03	3.06	2.61	4.42	3.7
ADM2	2.22	2.27	2.47	2.16	2.24	2.62	2.03	2.4
HARBI1	2.49	2.88	2.57	2.17	2.72	2.62	3.54	2.52
ARL10	2.71	2.22	2.83	2.81	2.23	2.62	2.95	2.56
SLFN5	2.84	2.92	3.25	3.86	2.58	2.62	3.28	2.99
SALL2	3.89	3.87	3.73	3.52	2.83	2.62	2.39	3.08
TRAK2	2.29	2.31	2.36	2.79	2.56	2.62	3.32	3.18
CPEB4	2.39	2.11	2.89	2.93	2.47	2.62	3.82	3.27
NOVA2	2.74	2.78	2.7	2.69	2.49	2.63	2.13	2.53
ING2	2.96	3.51	3.35	2.64	2.42	2.63	3.45	2.61
GSTCD	2.65	1.78	2.88	2.94	2.7	2.63	3.69	3.01
ZNF554	2.54	3.11	3.12	2.88	3.8	2.63	3.7	3.05
LOC100505549	2.16	2.71	2.54	2.41	2.88	2.63	2.95	3.16
DCLRE1A	2.36	2.38	2.68	3	2.35	2.63	4.41	3.17
DAB2	2.58	2.45	2.45	2.99	2.42	2.63	3.03	3.32
LOC100130451	2.1	2.04	2.13	2.92	2.99	2.63	3.53	3.61
SPIN4	2.06	2.12	2.25	2.58	3.04	2.63	4.02	3.62
ANKRD36B	1.86	1.91	1.99	2.83	2.83	2.63	4.27	3.96
ZMYM6	2.38	2.39	3.15	3.24	3.26	2.63	4.63	4.5
ZNF287	2.05	1.93	2.06	2.16	2.3	2.64	2.87	2.46
TCN2	3.05	3.16	2.51	2.82	3.01	2.64	2.46	2.49
MAP2K6	2.39	2.48	2.46	3.16	2.25	2.64	3.44	2.75
RAPGEF3	2.22	2.15	1.78	2.11	2.68	2.64	2.72	2.78
ENOX2	2.58	2.05	2.63	2.76	3.01	2.64	3.6	3.12
TTC26	4.12	2.43	2.86	3.86	2.71	2.64	3.67	3.55
C3orf33	2.95	2.22	3.17	3.6	3.5	2.64	3.83	3.55
EMSY	2.48	2.47	2.9	3.22	2.83	2.64	4.24	3.85
ZNF326	2.6	2.85	3.13	4.07	3.3	2.64	5.01	4.21
FBXO8	2.72	2.71	3.41	3.85	3.27	2.65	4.1	3.33
TIGAR	1.74	1.67	1.75	2.13	2.93	2.65	3.65	3.5
DLX5	3.65	4.14	4.03	3.62	2.43	2.65	4.04	3.61
TRMT11	2.09	2.05	2.33	2.85	2.72	2.65	4.49	3.81
ZNF354C	2.32	2.27	2.75	3.9	2.4	2.65	4.78	3.94
ZDHHC21	2.29	1.97	2.53	3.11	2.78	2.65	4.86	3.94
DCP2	2.13	2.18	2.57	3.32	2.44	2.65	4.76	4.07
MTX3	2.08	1.76	2.4	3.04	2.42	2.65	5.16	4.25

PPP1R12B	1.72	2.03	2.29	3.28	2.76	2.66	3.35	2.77
ZFP28	2.57	2.15	2.59	2.88	2.44	2.66	3.97	2.79
C1QTNF6	2.65	2.26	2.71	2.91	2.28	2.66	3.5	2.96
HHIP	3.04	3.02	3.19	3.6	3.11	2.66	3.44	3.23
ZNF331	2.39	2.45	2.53	3.19	2.61	2.66	3.72	3.33
PPFIBP2	4.16	4.03	4.61	4.38	3.73	2.66	4.04	3.55
COL11A1	3.25	3.31	3.7	4.03	2.69	2.66	3.64	3.76
ASB9	1.66	1.78	1.41	1.41	2.42	2.67	2.23	2.62
DMRT2	3.24	3.16	3.54	2.76	2.92	2.67	2.32	2.96
ZNF827	2.99	2.49	2.94	2.93	2.75	2.67	3.06	3.17
VAMP4	2.12	2	2.59	2.84	2.59	2.67	4.57	3.85
ZUFSP	2.31	2.45	2.88	3.31	3.37	2.67	4.59	4.14
C5orf42	2.52	2.26	3.1	3.89	2.73	2.67	5.15	4.56
IL15RA	2.66	2.55	2.47	2.32	2.22	2.68	2.88	1.86
C17orf96	3.32	3.28	2.85	2.54	2.34	2.68	2.19	2.55
ING1	2.53	2.91	3.21	2.84	2.7	2.68	2.88	2.92
MYNN	2.82	3.24	2.64	3.02	2.65	2.68	4.21	3.65
FZD3	2.81	2.75	2.71	3.71	2.98	2.68	4.5	4.26
ZNF91	2.59	2.61	2.67	3.81	2.74	2.68	4.69	5.09
KIF24	2.86	2.33	2.86	2.59	2.93	2.69	2.72	2.34
ADAMTS13	2.22	2.06	2.68	2.14	2.39	2.69	2.75	2.44
LZTS1	3.36	3.69	3.25	3.25	2.67	2.69	2.53	2.63
PEX11A	2.87	2.87	3.08	2.94	2.86	2.69	3.05	2.84
TCEANC2	2.3	2.08	2.68	2.49	2.88	2.69	3.32	3.02
PDZD2	2.59	2.26	2.86	3.17	2.73	2.69	3.66	3.11
RNASEL	2.78	2.87	2.96	3.45	2.84	2.69	3.32	3.22
PAPD5	2.12	2.01	3.01	3.27	2.66	2.69	3.92	3.24
LRP5L	3.8	3.74	3.44	4.31	3.34	2.69	4.24	3.73
ZBED8	2.98	2.63	3.16	3.54	2.94	2.69	4.35	3.84
ZNF292	2.59	2.55	2.94	3.94	2.68	2.69	4.83	4.29
ZNF22	5.23	4.93	5.37	6.62	2.78	2.69	4.94	4.45
CIB2	2.81	2.96	2.88	2.96	2.16	2.7	1.8	1.62
LIMS4	1.95	2.18	2.21	2.37	2.24	2.7	2.27	2.29
FOXL1	2.88	2.75	2.74	2.65	2.52	2.7	2.38	2.34
NOTCH3	3.43	3.95	3.52	3.45	2.65	2.7	2.26	2.86
POLR3B	2.35	2.16	2.44	2.83	2.79	2.7	2.99	2.95
AKAP7	2.11	2.34	2.42	3.55	2.38	2.7	3.51	3.17
USP46	2.53	2.08	3	3.37	2.79	2.7	3.9	3.56
PGAP1	2.54	2.56	2.85	3.96	2.64	2.7	5.12	4.56
MME	2.75	2.38	3.47	3.81	2.74	2.7	4.98	4.61
HLA-G	4.17	3.54	3	2.9	2.33	2.71	2.06	1.64
ARHGAP25	2.4	2.34	2.4	2.06	2.38	2.71	2.84	2.08
LOC107983998	1.81	1.26	1.5	2.25	1.8	2.71	2.16	2.19
NOD1	2.08	2.12	2.27	2.33	2.65	2.71	3.09	2.65
PPM1N	3.03	2.63	2.02	2.5	2.38	2.71	2.68	2.81
HERC1	2.54	2.41	2.71	2.8	2.46	2.71	3.11	3.1
PAQR6	2.51	2.47	2.67	2.95	2.82	2.71	3.32	3.38
MMS22L	2.33	1.95	3.08	3.39	2.88	2.71	4.75	3.57
NBEA	2.08	2.1	2.47	3.36	3.21	2.71	4.7	4.47
TGFBR3L	2.11	1.99	2.05	2.18	1.95	2.72	1.48	1.06
FAM118B	3.03	2.86	2.95	2.66	3.53	2.72	3.66	2.95
AGO3	2.53	2.26	2.58	3.22	2.74	2.72	3.07	3.17
NTN4	3.46	3.89	3.76	3.94	3.03	2.72	3.48	3.58
MYO5A	1.99	1.8	2.44	3.05	2.63	2.72	4.36	3.62
KIF14	1.76	1.72	1.98	2.73	2.51	2.72	5.79	4.66
NIM1K	1.88	1.53	1.9	1.51	2.56	2.73	2.19	2.56
SLITRK5	3.08	2.9	3.21	2.99	2.62	2.73	2.91	3.07
ANKRD42	2.1	2.35	2.61	2.62	2.83	2.73	3.84	3.13
CRY1	2.67	2.46	2.73	2.97	2.85	2.73	3.19	3.18
POLR3G	1.85	2.38	2.23	2.6	2.38	2.73	3.65	3.2
ARNTL2	2.55	2.25	2.83	2.87	3.32	2.73	3.85	3.32
HOXB5	3.54	3.71	3	3.05	3.08	2.73	3	3.76
PBX3	3.12	3.14	3.36	3.41	3.27	2.73	3.23	3.93
SNED1	3.35	3.47	3.43	3.83	2.3	2.74	2.23	2.63
DUSP18	2.66	3.03	2.6	2.99	2.59	2.74	2.77	3
SYNGAP1	3	3.12	3.04	3.24	2.82	2.74	2.84	3.05
CTH	3.51	3.27	3.16	3.18	3.09	2.74	4.03	3.8
MTBP	2.24	2.15	3.02	2.63	3.02	2.74	4.67	3.9
IQCG	2.16	2.28	2.33	2.45	2.41	2.75	3.43	2.74
C2CD2L	2.69	3.07	3.03	2.71	3.16	2.75	2.95	3.02
SOCS6	2.33	2.58	2.52	3.01	2.78	2.75	3.47	3.16
HIST1H2BD	3.7	3.73	3.97	4	2.42	2.75	3.67	3.19
ZKSCAN2	2.72	2.68	2.88	3.13	2.73	2.75	3.1	3.28
SNTB2	2.36	2.46	2.75	3.25	2.93	2.75	3.84	3.53
PIGB	2.7	2.47	2.59	2.79	2.39	2.75	3.49	3.59
TMEM17	2.95	2.32	2.58	3.22	3.04	2.75	4.09	3.98
CACNB4	2.64	2.43	2.99	3.31	2.68	2.75	5.14	4.49
GADD45B	2.75	3.17	3.33	2.47	3.28	2.76	2.41	2.16
LOC730183	2.53	2.71	1.84	2.5	1.51	2.76	2.47	2.2
TTLL11	2.02	1.89	2.13	2.22	2.72	2.76	2.27	2.7
ZNF16	3.46	3.17	3.36	3.52	2.79	2.76	3.1	2.85
PCYT1B	2.49	2.86	3	2.89	2.64	2.76	2.73	2.86
MDM1	1.85	2.45	1.97	2.88	2.54	2.76	4.53	3.25
CCDC138	1.98	1.93	2.06	2.45	2.59	2.76	4.26	3.49

KRBOX4	2.67	2.15	2.51	3.09	2.67	2.76	3.7	3.56
TRAPPC6B	3.41	2.2	2.62	3.5	3.04	2.76	3.94	4.16
G2E3	2.45	2.2	2.39	2.66	3	2.76	5.12	4.19
PPM1L	2.03	2.1	2.33	2.57	2.77	2.77	3.22	2.77
DDX10	2.07	1.92	2.35	2.54	2.72	2.77	3.28	2.77
LOC105379417	2.02	2.09	2.7	2.39	2.49	2.77	2.77	2.92
SYTL4	2.81	2.76	3.13	2.87	2.23	2.77	2.72	3.06
ANKRD34A	3.22	3.22	2.53	2.93	2.94	2.77	2.61	3.12
TUBGCP5	2.25	2.28	2.86	2.7	3.2	2.77	3.54	3.15
DNAJC12	2.92	1.91	3.09	3.08	3.25	2.77	4.57	3.19
RNF169	2.55	2.55	2.98	2.95	2.84	2.77	3.73	3.4
GNG2	2.29	2.68	3.03	3.18	2.72	2.77	3.89	3.5
ZNF700	2.57	2.94	3.05	3.59	2.63	2.77	4.49	3.67
CRIPT	2.57	2.37	2.52	3.23	2.77	2.77	3.92	3.68
MIER3	2.61	2.33	2.78	3.61	2.54	2.77	4.68	4.1
CBWD6	3.04	2.45	3.24	2.07	2.86	2.77	5.48	4.34
LOC100126447	3.19	2.24	2.91	4.82	3.33	2.77	4.5	4.87
DNAAF3	2.35	2.61	2.43	2.29	2.25	2.78	1.74	2.76
ZCWPW1	2.36	2.27	2.96	2.3	3.02	2.78	3.13	2.85
PRUNE2	2.3	2.68	2.75	3.13	2.75	2.78	3.56	3.27
GTPBP10	2.33	2.3	2.53	2.95	2.89	2.78	4.39	3.44
STON1	2.85	2.46	3.11	3.9	2.78	2.78	3.83	3.55
PLEKHG1	2.51	2.41	2.77	2.97	2.83	2.78	3.69	3.6
UTP14C	2.6	2.1	3.16	3.78	3.31	2.78	3.98	4.05
HOOK3	2.67	2.45	2.8	3.54	2.83	2.78	4.39	4.08
MYO1G	3.43	3.51	3.13	3.23	2.8	2.79	2.26	2.66
XPNPEP3	2.11	2.14	2.18	3.04	2.76	2.79	3.15	2.92
SERPINF2	3.33	3.61	3.48	3.76	2.74	2.79	2.79	3.1
SNX30	2.53	2.33	2.84	3.17	3.41	2.79	3.21	3.47
ZNF438	3.63	3.66	3.78	3.93	3.36	2.79	3.95	3.83
ZNF761	2.5	2.22	2.31	2.41	2.8	2.79	4.8	3.99
SCN1B	3.93	3.59	3.94	3.92	2.97	2.8	2.82	1.94
SYCE2	2.99	3.16	1.97	3.32	2.73	2.8	3.04	2.15
GSTM2	3.21	2.76	3.57	3.1	2.23	2.8	2.94	2.43
C17orf51	2.54	2.58	2.13	2.35	3.08	2.8	2.34	2.53
CDK5R1	2.28	2.64	2.44	2.5	2.6	2.8	2.72	2.99
ZNF365	2.3	2.39	2.19	2.93	3.28	2.8	3.9	3.37
FOXN2	2.55	2.69	2.53	3.05	2.51	2.8	4.61	3.53
PTCH1	3.85	3.7	4.24	4.04	2.9	2.8	3.78	3.71
ZNF419	3.05	2.57	3.73	3.51	2.48	2.8	3.72	3.84
FNIP1	2.04	1.99	2.35	3.42	2.66	2.8	4.84	3.88
GALNT13	1.8	2.12	2.51	2.52	3.3	2.8	4.08	3.9
DNHD1	2.14	1.92	2.36	2.64	2.78	2.8	4.12	3.96
RGAG4	3.43	4	3.04	3.44	2.46	2.81	2.34	2.86
DIAPH2	2.26	1.72	2.34	2.56	2.86	2.81	4.2	3.33
NHLRC2	2.57	3.07	2.47	4.01	3.46	2.81	5.01	4.45
EFCAB2	2.84	2.88	2.56	3.02	2.85	2.81	3.19	4.52
FLRT3	3.79	3.45	4.16	4.74	3.04	2.81	4.52	4.57
JMJD1C	2.43	2.15	2.9	3.75	2.79	2.81	5.43	4.66
SLC25A35	2.55	2.88	2.39	2.75	2.25	2.82	1.8	2.24
TUBB4A	3.46	4.07	3.67	3.19	2.4	2.82	1.8	2.47
WNT3	2.18	2.73	2.4	2.61	2.76	2.82	3.14	2.79
AMIGO3	3.64	3.38	3.59	2.94	2.68	2.82	2.79	3.03
CRIPAK	2.33	2.2	2.65	2.99	2.57	2.82	3.72	3.06
HACD1	3.46	3.11	3.18	2.67	3.04	2.82	2.97	3.09
RAD51B	2.91	2.81	2.83	2.92	3.13	2.82	3.33	3.14
MAGI1	2.95	2.46	3.1	3.51	3.03	2.82	3.77	3.31
FAR2	2.57	2.53	2.69	3.29	3.54	2.82	4.57	3.9
DTWD1	2.85	2.93	2.52	3.4	3.48	2.82	4.31	4.13
CLEC11A	2.63	2.64	2.05	2.66	2.01	2.83	1.78	1.64
BCL11A	2.96	3.23	3.14	3.16	2.49	2.83	2.73	2.44
TREM2	1.66	1.53	2.27	1.37	4.19	2.83	2.32	2.59
WASF3	2.43	2.47	2.38	3.19	2.73	2.83	3	2.98
MXD1	2.47	2.13	2.8	2.87	3.11	2.83	4.34	3.51
SYNJ1	2.35	2.29	2.64	3.15	2.73	2.83	4.14	3.62
ZBTB18	2.96	2.84	3.23	3.69	3.39	2.83	4.29	3.67
EXOG	2.37	1.99	3.2	3.21	2.92	2.83	3.45	3.82
ZNF506	2.94	2.63	3.35	4.13	3.02	2.83	4.77	4.01
C4orf36	2.89	2.55	2.91	3.93	2.94	2.83	5.15	4.49
HOXA5	2.58	2.97	2.35	2.31	2.54	2.84	3.26	2.25
DET1	2.44	2.65	2.3	2.64	2.55	2.84	2.81	2.88
PM20D2	2	1.94	2.86	2.44	2.48	2.84	3.12	2.96
OPHN1	3.05	2.68	3.11	3.35	2.65	2.84	3.6	3.28
KCNQ5	2.4	1.72	2.43	2.77	2.79	2.84	3.45	3.35
ZNF160	2.79	2.46	3.12	3.84	2.57	2.84	4	3.77
PCGF5	2.39	2.22	2.39	3.19	2.66	2.84	5.28	4.04
PCF11	3.08	2.83	3.25	3.98	3.35	2.84	4.58	4.23
FBLN7	3.24	3.46	3.42	2.79	2.59	2.85	2.35	2.55
MAF	3.4	3.07	3.15	3.17	3.03	2.85	2.7	2.76
ZNF425	3.21	3.17	3.29	3.13	2.59	2.85	2.34	2.89
OTUD6B	2.53	2.25	2.59	3.03	2.76	2.85	5.3	4.43
KIAA1109	2.43	2.18	2.82	3.7	2.65	2.85	5.9	4.85
CCDC149	2.99	2.82	2.92	2.81	3.21	2.86	2.93	2.95
CYB5RL	3.14	2.61	3.36	3.15	3.41	2.86	3.81	3.12
TDRD3	2.52	2.38	2.78	3.44	3.18	2.86	4.33	3.49

ROM1	2.97	3.64	2.97	3.03	3.63	2.86	2.71	3.57
AHI1	2.3	2.25	2.74	2.85	2.52	2.86	5.38	4.15
TEX30	2.61	3.01	3.22	3.55	2.95	2.86	4.04	4.22
ZNF124	2.7	2.34	2.86	3.84	3.37	2.86	5.3	4.57
AADAC	1.85	1.46	3.11	2.82	2.65	2.86	5.81	4.61
SOCS4	2.38	2.02	2.42	3.42	2.97	2.86	5.94	4.91
SLC2A5	2.45	2.4	1.8	2.25	2.32	2.87	1.89	2.38
MT1F	4.39	3.82	3.51	4.9	3.86	2.87	3.36	2.56
NOMO3	2.52	4.17	1.98	1.42	3.66	2.87	2.72	2.57
DNM3	2.86	2.55	2.5	2.75	3.11	2.87	3.2	3.26
SETD4	3.21	3.31	2.67	3.17	3.7	2.87	4.27	3.58
USP37	3.11	2.76	3.26	3.69	2.85	2.87	4.04	3.77
MSANTD4	3.12	3.26	3.02	4.15	3.25	2.87	4.64	4.59
KIAA0753	2.55	2.65	2.94	3.45	3	2.88	4.49	3.51
ITGB8	2.87	2.84	3.2	4.18	3	2.88	5.73	4.65
HLA-F	3.05	3.19	3.04	3.14	2.23	2.89	2.85	2.03
ZBTB47	2.37	2.63	2.35	2.36	2.52	2.89	2.44	2.58
TEKT4	2.71	2.8	2.39	2.3	2.97	2.89	2.84	2.77
SCNN1D	1.91	1.63	2.21	2.56	3.01	2.89	2.94	2.83
MAPK8	2.3	2.69	2.94	3.1	2.83	2.89	3.9	3.49
LCLAT1	2.79	2.28	3.19	3.34	3.24	2.89	4.17	3.82
TMEM170A	2.85	2.87	3.04	3.41	3.04	2.89	3.61	4.08
JRKL	3.27	2.95	3.45	4.08	3.44	2.89	6.07	4.8
F8	3.34	2.76	3.51	3.87	2.93	2.9	4.16	3.52
ZFHX3	2.89	2.72	3.09	3.78	2.93	2.9	3.53	3.76
SEC61A2	2.94	3.22	2.64	3.58	3.7	2.9	4.12	3.99
UACA	2.91	2.87	3.35	3.81	2.91	2.9	4.89	4.11
PRRG1	2.89	2.7	3.05	3.93	3.42	2.9	5.19	4.26
ID2	2.11	2.29	2.11	2.45	3.4	2.91	2.83	2.33
ZFAT	2.51	2.54	2.49	2.42	2.74	2.91	2.46	2.69
LSM11	2.36	2.09	2.8	2.66	2.89	2.91	3.07	2.78
SLC1A4	2.92	3.26	2.85	2.65	3.04	2.91	2.97	2.9
ARPC4-TTLL3	2.43	2.35	2.8	2.82	2.3	2.91	3.28	2.96
PYROXD1	2.38	2.1	1.88	1.95	1.99	2.91	3.17	3.04
UAP1L1	2.82	2.8	3.02	2.78	2.58	2.91	3.1	3.09
EFHC1	2.81	2.43	2.66	3.31	2.93	2.91	4.42	3.49
GKAP1	2.49	2.86	3.24	3.39	2.74	2.91	3.88	3.66
LOC107986800	2.81	2.54	3.01	3.27	2.78	2.91	4.4	3.7
TBC1D31	2.8	2.39	2.88	3.24	2.71	2.91	5.04	4.09
ZNF253	2.63	2.16	2.78	3.61	2.58	2.91	5.04	4.17
MTMR9	3.19	3.21	3.52	3.33	3.84	2.91	4.93	4.27
NPHP3	2.54	2.28	3.27	3.56	2.59	2.91	5.96	4.59
RCN3	4.64	5.4	3.85	4.5	3.13	2.92	1.96	2
PNCK	3.38	3.05	2.7	3.35	1.49	2.92	2.17	2.12
CCDC157	2.86	2.32	2.99	2.49	2.17	2.92	2.62	2.44
GALK2	2.36	2.66	2.42	2.24	3.27	2.92	2.75	2.92
SCG2	1.45	1.17	1.7	1.64	2.17	2.92	3.61	2.99
PTPDC1	2.58	2.43	2.78	2.67	2.85	2.92	4.24	3.63
RASAL2	2.33	2.28	2.84	3.13	2.98	2.92	4.21	3.82
HACE1	2.9	2.17	3.41	3.71	3.15	2.92	5.13	3.85
ERCC4	2.62	2.02	2.63	2.93	2.94	2.92	3.84	3.91
FBXO30	2.76	2.51	2.84	3.58	3.31	2.92	4.84	4.4
ERCC6L2	2.21	1.99	2.88	3.68	2.82	2.92	5.16	4.56
ROCK2	2.59	2.24	3.24	4.05	2.72	2.92	5.21	4.68
RUFY2	2.81	2.2	3.15	4.14	2.74	2.92	5.7	4.74
EDARADD	4.47	4.34	4.09	4.13	2.81	2.93	2.73	2.85
MBOAT1	3.55	3.22	3.13	3.88	2.99	2.93	4.02	2.91
TMEM218	2.98	3.08	2.75	3.27	3.39	2.93	3.34	3.08
NDUFAF4	3.02	3.14	3.65	3.31	3.81	2.93	3.9	3.72
ZNF18	3.63	3.42	2.86	3.8	3.35	2.93	3.79	3.94
EFCAB7	3.63	3.44	3.33	3.94	3.55	2.93	5.35	4.43
LZTS3	3.37	3.3	3.14	2.96	2.98	2.94	2.79	2.93
ZNF304	3.04	3.12	2.9	2.97	3.13	2.94	3.53	3.4
TOX	3.22	2.91	3.26	3.41	3.61	2.94	3.37	3.64
TTBK2	2.69	2.25	2.99	3.4	2.79	2.94	4.31	3.74
EPM2AIP1	2.65	2.38	3.07	3.45	2.89	2.94	5.24	4.31
CEBPA	3.13	3.56	3.15	2.88	2.85	2.95	2.29	2.36
ZNF256	3.03	2.79	2.87	3.05	3.15	2.95	3.41	2.68
SLC24A3	2.61	3.05	2.85	2.63	3.26	2.95	3.44	3.03
MDFIC	2.46	2.76	3.81	3.55	2.85	2.95	4.12	3.11
CCDC153	2.44	3.22	3.03	2.75	2.84	2.95	2.77	3.47
PPP4R4	1.99	2.18	2.58	3.45	1.49	2.95	3.17	3.47
CD274	2.71	3.1	2.98	3.84	3.28	2.95	4.69	3.48
SIMC1	2.9	2.35	3.12	3.44	2.56	2.95	3.15	3.54
CNTN1	1.93	1.99	2.31	2.97	2.74	2.95	4.67	3.88
HAUS3	2.91	2.74	2.68	3.26	3.56	2.95	5.65	4.59
C9orf116	2.77	2.63	2.84	3.19	3.17	2.96	2.9	2.4
LOC107985108	2.78	2.66	2.42	1.67	2.09	2.96	2.47	2.42
CDKL4	2.83	2.94	2.99	2.99	2.54	2.96	2.29	2.43
TESMIN	3.04	2.56	3.08	2.49	3.04	2.96	2.62	2.62
TSHZ3	3.55	3.47	3.22	3.76	3.24	2.96	3.01	2.68
ZBTB46	4.15	3.67	3.84	4.24	3.71	2.96	2.91	2.7
JAK3	3.51	3.9	3.53	3.23	2.77	2.96	3.15	3.1
TRIM9	3	2.85	2.9	2.98	2.77	2.96	3.2	3.11

NBPF19	3.63	3.65	3.76	4.32	4.27	2.96	4.41	3.61
VCPIP1	2.62	2.65	2.96	3.51	3	2.96	4.66	4.19
TBCK	2.54	2.04	3.03	3.59	3.33	2.96	4.24	4.28
EGFL8	1.76	1.72	1.94	1.8	2.28	2.97	3.2	2.2
IQCD	1.46	2.1	2.05	2.34	2.6	2.97	2.36	2.9
AAK1	2.79	2.85	2.98	3.37	3	2.97	3.55	3.33
ALG11	2.27	1.88	3.25	3.2	2.5	2.97	3.83	3.42
DCUN1D2	2.93	2.75	2.59	3.15	3.16	2.97	4.13	3.45
NEK4	2.99	2.63	3.12	4.19	3.38	2.97	4.12	3.52
SLC24A1	3.39	3.07	3.49	3.95	3.54	2.97	4.02	4.05
FAM76B	2.33	2.48	2.72	3.19	2.78	2.97	5.32	4.11
DMXL1	2.38	2.07	2.85	3.34	2.79	2.97	4.93	4.14
LOC107984138	3.17	3.31	3.53	3.57	2.87	2.97	5.02	4.33
RBAK	2.62	2.57	3.3	3.56	2.64	2.97	5.54	4.87
NEMP2	3.11	2.47	2.76	2.5	2.72	2.98	2.96	2.26
LOC105371397	2.38	2.68	2.23	2.64	2.99	2.98	3.51	3.14
HOXD4	2.5	2.92	2.42	3.18	3.42	2.98	2.6	3.23
TET3	3.36	2.89	3.26	3.4	3.2	2.98	3.54	3.42
ACSL1	3.13	3.48	3.41	3.4	3.43	2.98	3.79	3.54
EPB41L4B	2.79	2.87	3.07	3.38	3.32	2.98	3.99	3.55
BMP2	3	2.74	2.88	2.91	3.19	2.98	3.19	3.63
SGO2	2	1.81	2.15	2.46	2.62	2.98	4.78	3.66
B3GLCT	2.65	2.37	2.95	2.92	3.45	2.98	3.91	3.79
PELI1	2.94	3.29	3.06	3.7	3.58	2.98	4.11	3.96
SREK1IP1	2.38	2.52	2.88	3.33	2.78	2.98	4.44	3.99
FAM161A	2.48	2.63	3.03	3.86	2.42	2.98	5.55	4.01
ACADSB	2.13	2.19	2.81	2.86	2.72	2.98	5.05	4.37
KIF18A	2.85	2.44	2.67	2.93	3.52	2.98	5.78	4.4
IL32	5.66	4.77	4.34	6.03	5.83	2.98	3.94	4.44
ZNF208	2.1	2.03	2.69	2.89	2.52	2.98	5.75	4.48
SSTR5	2.95	3.07	2.7	2.98	2.39	2.99	2.21	2.57
METTL18	2.92	2.75	3.11	3.41	3.37	2.99	3.95	3.03
MMACHC	2.83	2.71	2.34	2.78	2.8	2.99	2.11	3.05
TRIM39	3.32	3.04	2.83	2.82	2.92	2.99	3.06	3.2
MEIS1	3.76	3.48	3.99	4.4	3.48	2.99	4.48	4.35
MDM4	2.94	2.85	3.26	4.22	3.41	2.99	4.43	4.36
BCAT1	2.61	2.5	3.23	3.71	3.23	2.99	5.4	4.41
IRAK1BP1	2.26	2.3	2.76	2.7	1.85	3	2.68	2.6
SEMA3G	2.42	2.19	2.43	2.23	3.3	3	2.66	2.92
C1S	3.47	3.48	3.82	3.7	3.51	3	3.98	3.23
ICA1	3.13	3.14	3.1	3.2	3.16	3	3.81	3.59
SLC7A2	2.24	2.26	2.86	2.9	3.5	3	4.3	4.1
BAGE5	2.76	3.38	3.12	3.07	3.42	3	4.32	4.18
ZNF148	2.44	2.6	2.83	3.39	2.89	3	4.72	4.37
ZBTB41	2.67	2.7	2.65	3.03	3.14	3	4.12	4.73
LOC101928120	2.45	2.68	3.07	1.75	1.93	3.01	1.47	1.29
CPVL	2.42	1.92	2.35	2.41	2.51	3.01	2.64	1.89
FAM131C	2.83	3.86	2.7	2.7	3.32	3.01	2.34	2.3
MRAP2	2.71	3.01	2.97	2.93	2.76	3.01	3.17	2.5
CAPN5	3.07	3.54	3.07	2.81	2.82	3.01	2.15	2.66
WDR76	3.48	3.17	3.27	3.22	3.06	3.01	3.49	2.91
VSIG10	3.11	3.26	3.56	3.53	3	3.01	3.73	3.06
RAD54L2	3.35	3.34	3.43	3.59	3.07	3.01	3.28	3.21
ZNF416	3.21	3.83	3.85	3.32	3.25	3.01	3.5	3.36
ATXN7	2.94	2.47	3.31	3.68	3.24	3.01	4.83	3.97
ZFYVE16	2.56	2.27	2.58	3.4	2.85	3.01	5.36	4.54
ZNF324B	3.16	3.41	3.11	3.43	3.02	3.02	2.49	2.85
ARID3B	3.8	3.79	4.01	4.17	3.39	3.02	3.14	3.46
CPNE7	2.8	2.27	2.42	2.16	2.96	3.02	3.15	3.49
TAPT1	3.15	2.53	3.12	3.15	3.1	3.02	4.15	3.87
LOC105370045	3.39	2.7	2.91	3.11	2.95	3.03	1.16	1.6
AGAP2	3.35	3.15	2.7	2.53	2.71	3.03	2.27	2.91
SUSD5	4.14	3.87	4.38	4.54	2.53	3.03	2.93	3.04
FRS3	3.84	3.79	3.77	4.65	3.36	3.03	2.87	3.28
STC2	5.01	4.4	4.71	4.35	3.44	3.03	3.37	3.31
LARP1B	2.41	2.62	2.9	2.73	3.26	3.03	4.2	3.7
FAM69A	3.02	2.29	2.74	2.8	2.99	3.03	3.94	3.89
LOC107984026	1.91	1.6	1.6	3.03	2.42	3.04	3.17	2.43
OTX1	2.95	3.6	2.82	3.02	2.76	3.04	2.59	2.74
PRELID3A	2.41	1.81	2.1	2.46	3.05	3.04	3.14	3.05
POU2F1	2.65	2.78	2.89	4.21	3.37	3.04	4.38	3.34
METTL11B	3.62	3.36	3.15	2.5	1.98	3.04	2.68	3.42
SMIM13	2.98	2.92	3.25	3.6	3.07	3.04	4.28	3.99
OR7E24	3.04	3.51	2.96	3.76	4.21	3.04	3.63	4.23
HOMER1	3.64	2.89	3.13	3.52	3.27	3.04	4.53	4.28
TTC21B	2.89	2.5	3.15	4.09	3.13	3.04	5.5	4.64
PCDHA4	3.14	2.68	3.23	2.96	3.12	3.05	3.23	2.76
SOWAHC	3.25	2.53	2.9	3.13	2.46	3.05	3.11	3.31
RIC8B	2.99	3.06	2.92	3.53	3.16	3.05	3.9	3.64
HSPBAP1	2.99	2.4	2.66	2.74	2.82	3.05	4.27	3.85
FBXO4	4.43	5.17	4.56	5.91	3.95	3.05	5.32	3.99
EAF2	2.8	2.61	3.3	3.34	2.7	3.05	3.69	4.03
AGO4	3.38	3.18	3.85	3.76	3.21	3.05	4.65	4.33

PRCD	2.3	2.42	2.74	2.32	3.39	3.06	3.69	3.45
BIRC2	2.54	2.63	3.34	3.4	2.89	3.06	4.58	3.73
HEG1	3.63	3.77	3.84	3.99	3.62	3.06	3.83	3.84
POLK	2.64	2.56	3.08	3.86	2.56	3.06	6.38	4.98
EPHX4	3.08	2.91	2.87	2.87	2.89	3.07	2.82	2.55
ZNF200	3.19	3.03	3.13	3.09	3.16	3.07	4.06	3.09
USP54	2.8	2.97	3.35	3.39	2.85	3.07	3.34	3.12
ZNF8	2.72	2.74	2.97	2.69	3.24	3.07	2.8	3.28
PCDHGA10	3.59	4.19	3.46	3.85	3.49	3.07	3.19	3.46
TBC1D3E	2.57	1.81	2.33	2.36	2.51	3.07	4.76	3.49
TAOK3	2.83	2.57	3.25	3.6	3.25	3.07	4.02	3.6
SLC16A9	3.62	3.28	3.89	4.36	3.26	3.07	3.81	3.7
SPTSSB	2.52	2.73	3	3.97	4.47	3.07	6.06	3.92
CEP192	2.68	2.31	2.98	3.49	3.28	3.07	4.61	4.08
RBM41	2.26	2.23	2.85	3.16	2.88	3.07	4.56	4.1
RAPGEF6	2.23	2.47	3.07	3.63	2.74	3.07	5.4	4.72
NSUN6	3.29	2.93	4.02	4.07	3.07	3.07	5.82	5.02
NAPB	2.99	2.74	3.25	3.84	3.49	3.07	5.73	5.05
ZNF121	2.99	2.71	2.5	3.46	3.16	3.07	6.33	5.44
ARHGEF19	3.39	3.73	3.76	3.16	3.14	3.08	2.44	2.78
PLCD1	4.14	4.44	4.05	3.7	3.12	3.08	2.95	2.83
TMEM91	2.9	2.44	3.03	1.89	2.64	3.08	3.07	3.04
TTC28	2.96	3.05	3.08	3.34	2.89	3.08	3.21	3.25
LOC107985805	3.06	3.3	4.83	3.61	3.47	3.08	3.69	3.34
KIAA1841	2.34	2	2.6	2.92	2.54	3.08	4.03	3.37
TRIM13	2.94	2.66	3.19	3.8	3.06	3.08	3.83	3.7
NBPF10	2.1	2.26	2.98	3.36	2.81	3.08	3.55	4
FOXS1	2.41	2.45	2.38	1.83	2.67	3.09	2.75	2.42
LRRC56	2.83	2.42	2.43	2.45	2.32	3.09	2.71	2.49
MOCOS	2.38	2.56	2.47	2.5	2.71	3.09	2.49	2.59
CD83	3.49	3.03	3.26	3.64	2.87	3.09	3.19	2.72
GCNT2	2.87	2.58	2.76	2.69	2.71	3.09	3.36	3.38
GTF2H2C_2	2.06	3.4	3.39	3.09	3.64	3.09	3.51	3.38
C8orf37	2.4	2.51	2.4	2.42	2.54	3.09	4.03	3.93
ZNF480	2.79	2.38	3.08	3.82	2.79	3.09	5.13	4.75
HYKK	3.22	2.3	2.39	3.01	2.42	3.1	3.21	2.34
RIN3	2.9	2.88	3.11	2.99	2.61	3.1	2.35	2.38
SLC2A12	2.85	2.9	2.95	3.54	3.12	3.1	3.68	3.85
TBRG1	2.73	2.47	2.59	3.03	2.78	3.1	3.9	3.95
UGGT2	2.71	2.74	2.77	3.25	3.06	3.1	5.13	4.56
CCDC151	2.99	3.31	3.08	2.87	2.72	3.11	2.97	2.78
CATSPER1	2.02	2.19	2.3	2.21	3.3	3.11	2.42	2.9
EPC1	3.37	2.7	3.41	3.37	2.99	3.11	4.29	3.56
GRPEL2	2.53	2.03	2.68	3.61	3.28	3.11	4.15	4.16
HYI	2.86	3.12	2.54	3.49	2.4	3.12	1.99	1.54
C1orf106	4.37	4.06	4.25	3.97	3.18	3.13	2.82	2.69
CDC25A	3.48	3.48	3.15	3.43	3.26	3.13	3.1	2.7
KIAA0513	2.78	3.12	2.81	2.65	3.23	3.13	2.5	2.97
EXD3	3.51	3.86	3.52	2.65	3.34	3.13	2.79	3.45
TSC1	2.96	2.86	3.13	3.44	3.22	3.13	4.17	3.58
ZNF551	3.04	2.73	3.18	3.61	3.29	3.13	4.12	3.76
PKD2	3	2.76	3.19	3.55	2.55	3.13	4.59	3.89
NLK	2.59	2.33	3.19	3.16	2.96	3.13	4.07	3.91
CDK19	3.24	3.43	3.58	3.87	3.39	3.13	4.78	4.48
ZC3H8	2.17	2.09	2.25	3.24	3.62	3.13	4.83	4.76
IFT81	3.34	3.19	3.67	3.78	3.33	3.13	5.53	4.9
ZNF69	2.75	2.33	2.98	4.18	2.62	3.13	5.19	5.02
WSCD1	3.26	3.45	3.49	3.1	2.91	3.14	2.35	2.38
ITFG2	2.29	2.8	2.62	2.42	2.97	3.14	3.13	2.8
C16orf87	3.15	3.57	3.08	3.79	3.7	3.14	4.47	3.46
TMCC2	4.35	4.21	4.26	3.84	3.58	3.14	2.65	3.69
ZNF354A	2.74	2.34	3.38	3.26	3.13	3.14	5.75	4.57
GTF2A1	2.91	2.83	3.08	4.13	3.38	3.14	5.72	4.84
MBTD1	3.03	2.88	3.47	4.15	3.9	3.14	6.81	5.28
TRPM7	2.18	2.17	2.92	3.85	3.2	3.14	6.54	5.85
SLC37A1	2.24	2.49	2.15	2.58	2.87	3.15	2.27	2.53
LATS2	2.69	2.73	3.04	2.87	2.83	3.15	3.02	2.87
ACOT2	3.87	3.96	4.11	4.34	3.74	3.15	3.22	3.03
TNFRSF11B	2.45	2.37	2.7	2.84	2.64	3.15	3.17	3.25
FAM126A	2.78	2.81	3.22	3.72	3.13	3.15	5.43	4.72
KIAA1586	2.56	2.07	2.74	2.34	2.22	3.15	4.75	4.79
KLF4	2.75	2.43	2.53	2.59	3.4	3.16	3.37	3.07
CD1D	4.31	4.4	3.6	4.38	3.22	3.16	3.67	3.12
TMEM265	2.63	2.98	3.26	3.4	3.01	3.16	3.26	3.52
ZNF286A	2.72	2.37	3.13	3.67	3.24	3.16	5.21	4.67
C1GALT1	3.24	3.18	3.62	4.83	3.03	3.16	5.58	4.88
CACNA1H	3.26	3.38	3.39	3.28	3.26	3.17	2.46	2.72
CNIH3	2.91	3.65	3.44	3.32	3.42	3.17	2.94	2.91
TSPAN7	2.89	3.11	2.62	3.01	3.13	3.17	2.97	3.24
FBXW7	2.6	2.31	2.84	3.61	3.21	3.17	4.96	4.34
CAMKMT	3.34	2.89	2.48	3.8	3.39	3.17	3.82	4.4
GEN1	2.47	2.4	2.94	3.29	3.1	3.17	5.91	4.64
PLA2G4B	3.23	3.61	4.12	2.53	4.13	3.18	3.18	2.3

TMEFF1	1.79	2.01	1.61	1.78	1.73	3.18	2.49	2.69
KRT4	1.64	1.46	1.8	1.4	3.55	3.18	2.29	2.75
RNF157	4.29	3.82	3.74	3.91	3.32	3.18	3.03	3.17
ZNF333	2.67	2.99	2.97	2.9	3.07	3.18	3.72	3.31
EPC2	3.01	2.9	3.11	3.75	2.89	3.18	4.42	3.77
GCNT1	2.77	3.04	3.28	3.61	3.22	3.18	4.02	3.91
INTS6	3.13	2.87	3.76	3.91	3.24	3.18	4.23	4.04
SOCS5	3.21	3.27	3.52	3.87	3.33	3.18	5.63	4.18
HELZ	3.12	3.15	3.67	4.54	3.4	3.18	4.83	4.74
PROB1	2.77	2.74	2.62	2.65	3.03	3.19	2.69	3.02
GYLTL1B	3.86	3.64	3.37	4.13	3.83	3.19	2.64	3.29
ZNF595	2.8	3.09	3.12	3.94	3	3.19	4.02	3.52
METTL21B	3.56	3.1	3.34	3.81	3.3	3.19	3.82	3.7
ZBTB2	3.15	3.4	3.16	3.68	3.21	3.19	4.1	3.91
FAM234B	3.74	3.65	3.96	4.29	3.74	3.19	4	4.27
SPATA7	2.38	3.6	3.66	4.07	3.78	3.19	4.94	4.68
DPP8	2.9	3.01	3.27	4.87	3.62	3.19	5.9	4.8
USPL1	2.72	2.39	3.38	3.57	3.08	3.19	5.39	4.81
NRIP1	2.71	2.56	3.65	4.14	3.08	3.19	5.82	5.06
LRRC27	3.13	3.09	2.75	2.86	2.92	3.2	2.97	3.16
WDSUB1	3.96	2.93	3.22	3.47	3.18	3.2	4.64	3.21
ANKRD33B	2.47	2.33	2.76	2.59	3.54	3.2	3.7	3.59
CCDC78	1.39	1.55	2.38	1.71	2.95	3.2	3.27	3.74
FBXL20	2.87	2.7	2.86	3.23	3.42	3.2	3.79	3.81
HUS1	2.32	2.18	3.12	3.12	3.55	3.2	4.1	3.83
PURA	3.47	2.88	3.71	3.75	3.99	3.2	4.74	4.06
MON2	2.97	2.76	3.43	3.62	3.37	3.2	5.38	4.14
FAM35A	3.03	3.17	2.84	3.38	3.44	3.2	5.8	4.7
ITGA1	2.89	2.55	3.69	3.61	4.07	3.2	6.31	4.86
PARP15	2.68	2.95	3.4	4.99	3.13	3.2	8.75	5.52
CHAC2	2.2	3.19	2.66	2.95	2.66	3.21	3.73	2.87
PER1	3.13	3.35	2.75	3.11	3.66	3.21	2.79	3.18
TNRC6B	2.77	2.64	2.95	3.35	3.16	3.21	3.57	3.36
TNFAIP3	3.73	3.52	3.49	3.64	3.51	3.21	4.13	3.58
NUFIP1	3.05	3.03	3.56	3.38	3.05	3.21	3.66	3.69
C3orf62	3.36	2.89	3.52	3.65	3.6	3.21	4.02	3.88
LGALSL	3.03	3.38	3.11	3.36	3.42	3.21	3.99	3.92
KIAA1524	2.98	2.73	3.61	3.59	3.87	3.21	5.46	4.36
FAM175A	2.21	2.29	2.52	3.34	3.09	3.21	5.51	4.42
WDR72	3.36	3.11	3.58	4.09	3.29	3.21	5.53	4.6
ANKRD18A	2.28	2.04	2.46	2.73	2.85	3.21	5.51	4.8
LOC101929950	3.56	2.58	4.31	4.59	3.26	3.21	5.23	5.02
KHDRBS3	2.92	2.44	2.75	2.29	3.73	3.22	2.81	2.4
LETM2	2.26	2.02	2.49	2.74	2.83	3.22	3.61	2.75
PCDHB9	3.96	3.92	3.93	4.26	3.41	3.22	3.4	3.37
C2CD3	3.24	2.92	3.17	3.48	2.84	3.22	4.1	3.48
NSUN3	2.66	2.05	2.66	3.65	2.34	3.22	4.26	3.72
HECA	2.86	2.37	3.11	4.07	3.23	3.22	5.04	4.27
RAB3IP	3.13	3.98	4.01	4.02	2.9	3.22	5.76	5.3
PIGBOS1	2.16	2.83	2.44	2.82	3.01	3.23	3.7	2.84
LOC101929796	2.1	2.77	3.23	2.99	3.41	3.23	2.4	2.87
BICD1	3.02	2.78	3.18	3.08	3.48	3.23	3.17	3.31
TMEM47	3.39	3.29	4.03	4.43	2.97	3.23	5.4	3.94
METTL8	2.3	2.17	2.59	3.04	3.98	3.23	4.68	3.97
STRN	2.51	2.16	3.12	3.32	3.35	3.23	5.4	4.31
NIPA1	3.43	3.12	3.88	4.34	3.8	3.23	5.29	4.51
COX7B2	2.37	2.95	2.4	2.04	3.91	3.24	3.61	2.02
FOXC2	2.97	2.95	3.2	2.46	2.4	3.24	2.17	2.51
MPP3	1.79	2.05	1.98	2.21	2.87	3.24	3.97	2.7
AP4S1	3.01	2.76	2.95	3.34	2.68	3.24	3.55	3.41
STAG3	2.41	3.05	3.28	3.2	3.46	3.24	3.33	3.54
NFYB	2.99	2.59	3.29	3.26	2.91	3.24	4.45	3.67
CYP20A1	3.27	3.34	3.33	4.21	3.31	3.24	4.2	3.77
PRIMPOL	3.22	2.91	3	3.7	2.74	3.24	3.71	3.97
GPAM	2.75	2.46	3.01	3.43	3.45	3.24	4.58	4
MIS12	3.3	3.24	3.57	4.09	3.89	3.24	5.43	4.94
ONECUT3	3.55	2.89	2.99	2.99	1.96	3.25	2.05	2.44
RINL	2.6	2.75	2.56	2.52	3.61	3.25	3.18	3.14
TNFRSF10C	3.48	2.74	3.07	3.1	3.15	3.25	3.75	3.22
ITGA10	5.92	5.91	5.86	6.26	2.96	3.25	3.18	3.56
TMEM216	4.62	4.16	3.76	4.28	3.09	3.25	4.06	3.68
PUS3	3.12	3.93	3.32	3.79	3.93	3.25	4.98	3.96
AP1AR	2.68	2.95	3.26	3.6	3.61	3.25	6.09	4
LOC107984351	5.06	4.5	3.72	3.61	3.19	3.25	4.43	6.47
GLYCTK	2.98	2.45	2.17	2.52	3.72	3.26	2.89	3.09
CAMKK1	2.79	3.04	2.87	2.9	3.52	3.26	2.41	3.17
FPGT	2.87	2.71	2.88	3.71	3.3	3.26	5.21	3.68
SLC25A30	3.27	2.66	3.52	3.88	2.75	3.27	3.58	3.29
GPATCH1	3.35	3.28	3.57	3.06	3.43	3.27	3.79	3.96
ZNF417	3.26	2.93	3.23	3.49	3.01	3.27	4.76	3.98
C6orf141	2.78	2.53	2.96	2.92	3.04	3.27	4.79	4.01
BIVM	2.66	2.4	3.03	3.4	3.33	3.27	5.25	4.04

CYP4V2	3.53	3.37	3.86	4.99	3.63	3.27	5.11	4.16
SAMD8	2.59	2.4	3.3	3.39	3.33	3.27	5.51	4.8
MYSM1	2.37	2.1	2.63	3.71	2.88	3.27	7.42	5.38
ZNF770	3.52	3.57	4.47	5.72	3.92	3.27	7.02	6.15
USP18	7.23	8.48	7.09	7.11	4.11	3.28	3.51	3.33
CCDC69	3.14	3.23	3.22	3.74	2.83	3.28	3.3	3.56
USP3	3.36	3.23	3.44	3.66	3.52	3.28	3.91	3.66
SGO1	2.94	2.39	3.21	3.37	3.39	3.28	5.42	3.9
UPRT	3.24	3.35	3.57	3.3	3.09	3.28	4.52	3.93
PPP1R15B	3.26	3.09	3.99	4.3	3.81	3.28	4.61	4.25
ZNF600	3.04	2.84	2.96	4.58	3.06	3.28	5.35	4.58
TTC8	3.49	3.46	3.86	3.99	4.41	3.28	5.6	5.23
BDP1	2.86	2.88	3.05	3.75	3.5	3.28	6.86	5.43
KCNC4	2.35	2.14	2.26	2.17	2.72	3.29	2.67	2.68
EFCAB11	3.49	2.52	3.45	3.22	3.15	3.29	3.96	2.9
SLC25A25	3.68	3.04	3.35	3.66	3.08	3.29	3.54	3.4
IFNLR1	3.22	3.71	3.27	3.72	4	3.29	3.87	4.07
RAB11FIP2	2.11	2.36	2.98	2.78	3.04	3.29	4.49	4.22
BDNF	3.63	3.34	3.97	3.86	3.93	3.29	4.49	4.27
SRBD1	3.04	2.59	3.62	3.46	3.41	3.29	5.72	4.35
SRFBP1	2.69	3.14	3.33	3.48	3.19	3.29	5.37	4.61
NAPEPLD	3.06	3.35	4.04	4.4	3.43	3.29	5.34	4.92
SATB1	3.29	3.7	3.8	4.25	3.27	3.29	4.96	5.28
VPS13A	2.67	2.62	2.92	3.59	3.25	3.29	6.64	5.78
DND1	3.68	2.95	2.78	3.44	3.05	3.3	2.26	2.01
CCDC189	2.76	2.72	2.93	2.83	3.48	3.3	3.1	3.04
GDAP2	2.74	2.81	2.68	3.15	3.32	3.3	3.75	3.06
PLEKHO2	3.89	3.72	3.41	3.36	3.32	3.3	2.85	3.33
ZNF329	3.37	3.79	3.74	3.46	3.71	3.3	4	3.33
IQCE	3.93	3.61	3.86	3.98	4.18	3.3	3.75	3.51
SLC35F3	4.28	4.53	4.29	4.36	3.45	3.3	3.67	3.6
PCSK5	3.76	3.39	4.03	3.97	3.31	3.3	3.39	3.78
ARHGAP26	3.07	3.05	3.52	3.23	2.94	3.3	3.47	4.03
MLH3	2.99	2.45	3.6	3.7	2.83	3.3	5.18	4.07
SENP1	3.21	2.88	3.64	4.36	3.5	3.3	4.74	4.26
WHAMM	3.61	3.36	3.94	4.14	3.72	3.3	5.26	4.52
LMBRD2	3.46	3.68	4.45	4.58	4.19	3.3	6.97	5.06
HS3ST3A1	3.29	3.06	3.06	3.17	3.21	3.31	2.7	2.81
FOXC1	3.86	3.79	3.63	3.59	2.95	3.31	3.12	3.18
ZNF517	3.67	3.5	3.99	3.51	2.72	3.31	2.67	3.22
EPB41L1	2.24	2.79	2.84	2.56	3.4	3.31	2.98	3.44
ZNF560	2.72	2.88	2.99	3.89	3.58	3.31	3.7	3.66
SEMA5A	3.44	3.32	3.94	4.2	3.12	3.31	4.04	3.87
CLSPN	3	2.49	3.56	4.02	3.28	3.31	4.68	3.99
ZNF841	2.82	2.12	3.03	3.92	3.26	3.31	4.62	4.15
KIF15	2.99	2.37	3.42	4.06	3.33	3.31	5.97	4.84
TMEM260	3.33	3.52	3.78	4.42	3.79	3.31	4.23	4.87
FAM117A	3	3.46	4.17	3.54	3.21	3.32	2.78	3.28
LY6G5B	2.09	2.15	1.24	2.87	2.92	3.32	3.89	3.29
GABPB2	3.22	2.62	3.04	3.83	2.96	3.32	3.49	3.38
MCM10	4.08	3.25	4.24	4.58	3.78	3.32	4.71	3.92
ZBTB26	2.39	2.9	3.11	3.19	3.28	3.32	4.84	3.95
NOVA1	3.3	3.24	3.22	4.31	3.27	3.33	3.92	3.34
PHKA1	3.02	2.7	3.06	3.21	3.53	3.33	3.92	3.67
NRDE2	3	3.4	3.16	3.26	3.38	3.33	3.85	3.85
HINT3	3.29	2.79	3.07	4.01	3.05	3.33	5.31	4.56
TUBD1	2.77	2.08	3.56	3.96	3.28	3.33	6.24	4.59
WDR19	3.14	3.01	3.49	3.98	3.69	3.33	5.42	4.73
FKBP14	3.14	2.64	3.54	3.67	3.31	3.33	5.84	4.95
NPIPB6	3.61	2.66	4.51	4.76	4.12	3.33	6.88	6.1
RFX2	2.91	3.14	3.28	2.67	3.16	3.34	2.62	2.92
LOC105372731	2.76	3.35	2.78	2.87	3.01	3.34	2.65	3.01
SHPK	3.28	3.6	3.08	3.14	2.95	3.34	3.1	3.25
LCAT	2.99	2.63	2.07	2.04	3.18	3.34	3.57	3.26
NPHP1	2.63	2.8	3.2	3.3	2.8	3.34	4.69	3.45
S1PR2	3.5	3.77	3.33	3.8	3.02	3.34	3.14	3.47
FGFR1OP	3.28	2.5	3.32	4.19	3.64	3.34	6.16	4.65
ZNF667	2.95	2.25	3.1	3.75	3.37	3.34	5.72	4.7
TRAF5	3.07	2.59	3.6	4.07	3.24	3.34	6.03	4.71
ZKSCAN8	3.95	3.65	4.36	5.5	3.44	3.34	5.93	5.33
PIK3C2B	2.99	2.85	2.73	3.25	2.96	3.35	3.3	3
PPP2R1B	2.79	3.15	2.85	3.17	3.23	3.35	3.95	3.4
TBC1D24	3.52	3.52	3.51	3.52	3.61	3.35	3.83	3.55
WDR31	3.55	3.39	3.67	3.68	3.56	3.35	3.48	3.58
ETS1	3.12	2.49	3.24	3.65	3.72	3.35	4.27	4.18
DNAJC24	2.54	2.54	3.16	2.94	3.27	3.35	4.81	4.66
SENP7	2.89	3.53	4.56	4.96	4	3.35	5.45	5.55
FAM110B	3.06	3.29	3.52	3.65	3.1	3.36	3.08	2.54
BMP4	3.7	4.15	3.84	3.86	2.95	3.36	2.57	2.85
NBPF12	3.09	2.92	3.33	3.69	3.15	3.36	4.43	3.73
ZNF236	3.25	2.72	3.49	3.6	3.6	3.36	4.72	3.9
ARRDC3	3.24	3.28	4.1	4.88	3.5	3.36	5.62	4.17
FNDC4	3.16	2.81	2.54	2.25	2.75	3.37	2.03	2.43
FCHO1	3.46	3.56	3.19	3.28	2.74	3.37	2.93	2.98

KIAA1549L	2.86	2.91	2.91	2.69	2.55	3.37	3.53	3.19
LBH	4.33	5.05	4.76	4.7	4.55	3.37	3.77	3.24
ZNF564	2.86	2.53	3.58	3.33	3.47	3.37	4.66	4.05
DYRK2	3.25	2.68	3.36	3.56	3.14	3.37	4.12	4.08
WDR5B	3.45	2.98	3.45	3.72	3.49	3.37	5.26	4.24
UTP15	2.67	2.64	3.12	4.05	3.68	3.37	5.27	4.43
ZBTB6	2.82	2.83	3.55	3.87	3.51	3.37	5.73	4.43
KLHL23	2.85	2.85	3.42	4.28	3.79	3.37	4.26	4.62
TRAPPC13	2.59	2.61	3.15	4.09	3.71	3.37	6.07	5.06
MTR	3.19	3.03	4.03	4.71	4	3.37	5.39	5.21
ZNF676	2.98	3.22	4.01	4.64	3.65	3.37	6.28	5.32
SHROOM3	4.14	3.52	4.01	3.59	3.4	3.38	3.76	3.46
USP2	3.65	3.22	3.27	3.43	3.67	3.38	3.31	3.55
EMP1	3.73	3.63	3.86	4.14	3.76	3.38	4.31	3.63
METAP1D	2.82	2.78	3.11	3.81	3.83	3.38	4.47	4.36
FAM117B	3.04	2.84	4.17	3.76	3.15	3.38	4.88	4.45
FBXL3	3.33	2.75	3.61	3.76	3.29	3.38	5.42	4.56
ZCCHC6	3.25	2.65	3.55	4.21	3.52	3.38	5.98	5.14
PARPBP	2.49	2.35	2.83	3.23	3.14	3.39	4.84	3.63
IL17RD	4.09	3.58	3.84	4.19	3.52	3.39	3.93	3.94
C5orf45	3.59	3.93	3.14	3.6	4.21	3.39	4.23	4.14
WRN	2.94	2.67	3.24	4.28	3.37	3.39	5.23	4.47
NXT2	3.46	3.77	4.43	4.1	3.62	3.39	6.16	4.92
PLEKHA4	5.29	5.08	4.44	4.88	3.35	3.4	2.38	2.64
COX19	2.74	3.01	3.02	2.79	3.71	3.4	3.15	3.34
POU4F1	2.75	2.33	2.8	3.05	2.86	3.4	3.7	3.34
MTFR2	2.59	2.07	2.56	2.27	2.82	3.4	4.19	3.56
SNRK	3.56	3.12	3.53	4.67	3.28	3.4	4.57	4.07
ZNF880	3.26	3.94	3.97	5.06	4.22	3.4	4.43	4.14
CCSAP	2.92	2.48	3.57	3.8	3.52	3.4	4.66	4.38
ZBTB1	3.57	3.49	3.77	3.94	3.42	3.4	5.28	4.62
SLC38A4	4.65	4.27	4.74	5.86	3.69	3.4	5.39	5.03
FAM120C	3.25	3.25	3.57	4.41	3.37	3.41	4.18	2.98
INPP5B	3.43	3.62	3.19	3.54	3.74	3.41	3.41	3.1
COQ2	2.75	2.49	3.13	2.41	3.37	3.41	4.02	3.45
AMACR	3.43	3.09	3.12	3.39	3.57	3.41	4.09	3.9
NAV2	3.46	3.27	3.33	3.7	3.8	3.41	4.04	4.12
PEX12	3.85	3.5	4	3.81	4.18	3.41	3.86	4.26
WDR89	2.94	2.7	3.14	3.44	3.57	3.41	4.6	4.27
FAM122C	4.98	3.59	3.92	4.21	3.88	3.41	5.59	4.63
SACS	2.21	1.93	2.82	3.27	3.32	3.41	6.97	5.5
CCDC125	2.85	3.05	2.81	3.68	3.44	3.42	3.98	2.85
IGF2BP1	3.79	3.55	3.72	3.52	3.18	3.42	3.53	3.39
BBS7	2.35	2.36	3.07	3.38	3.17	3.42	4.27	4.28
FASTKD2	3.14	3.03	3.6	3.69	3.53	3.42	5.36	4.59
ARMCX5	3.6	3.06	3.85	4.94	3.55	3.42	5.66	4.64
LINS1	2.96	2.48	3.6	3.81	3.43	3.42	6.14	4.86
FAM217B	3.34	2.84	4.06	4.41	3.89	3.42	6.16	4.88
NUDT17	3.27	3.61	2.92	3.4	3.38	3.43	3.23	2.79
EEF1A2	2.86	2.91	2.84	2.77	3.84	3.43	2.81	2.95
FBXO10	2.79	3.16	3.31	3.4	2.61	3.43	3.08	3.17
NHEJ1	3	3.39	3.65	2.89	3.69	3.43	3.54	4.01
RSBN1	3.45	3.25	3.69	4.13	3.47	3.43	4.21	4.02
ERMAP	3.78	3.59	3.51	4.46	3.5	3.43	3.97	4.1
TBC1D3L	3.26	2.59	3.37	5.14	3.94	3.43	3.52	4.3
RSC1A1	3.35	3.43	3.74	4.4	3.56	3.43	5.92	5.05
TMF1	3	2.9	3.45	4.01	3.47	3.43	6.37	5.32
LRRC29	2.8	2.73	3	3.03	3.1	3.44	1.78	3.41
SH2D5	2.76	2.59	2.92	2.94	3.68	3.44	3.23	3.46
CDKAL1	3.47	3.1	3.64	3.66	3.63	3.44	4.5	3.49
BAMBI	3.17	4.09	3.89	3.45	3.43	3.44	2.85	3.67
STK33	3.06	2.81	2.94	3.49	3.77	3.44	4.44	4.03
LRRC8B	2.87	2.26	2.88	3.72	3.34	3.44	4.86	4.17
RRAGB	3.5	3.2	4.11	3.42	3.8	3.44	4.35	4.67
HELLS	3.78	3.6	4.2	4.47	3.8	3.44	5.49	4.81
BATF2	5.36	5.54	5.67	5.09	2.86	3.45	2.85	2.71
MYL9	3.59	3.71	4.26	4.14	2.82	3.45	2.69	3.55
ARHGAP19	3.35	3.18	3.16	3.23	3.6	3.45	4.19	3.6
TULP4	2.84	2.78	3.13	3.7	3.39	3.45	3.91	3.96
DAGLA	3.37	3.38	3.37	3.4	3.16	3.46	2.81	2.81
WNT7B	3.04	3.7	2.98	3.05	3.34	3.46	3.01	3.37
IDNK	4.13	4.15	3.66	4.15	3.3	3.46	3.77	3.71
LRRC16A	3.3	3.37	3.62	3.83	3.7	3.46	4.36	4.19
IFIT5	4.54	4.33	4.69	5.78	3.58	3.46	5.48	4.32
NOV	3.15	2.95	3.06	3.05	3.85	3.46	3.86	4.39
ZNF211	2.99	2.84	3.31	3.87	3.65	3.46	5.19	4.4
INSIG2	3.99	3.7	4.31	4.79	2.89	3.46	5.04	4.51
ITSN2	2.73	2.69	3.54	4.3	3.25	3.46	5.25	4.54
EPB41L5	3.42	3.59	4.21	3.88	3.96	3.46	5.37	4.61
CBWD3	2.58	2.16	3.01	5.86	3.56	3.46	6.41	4.61
IFT88	3.29	2.92	4.11	4.14	3.35	3.46	5.83	4.64
PITPNM2	3.31	3.17	3.27	3.01	3.1	3.47	3.32	3.05
DOPEY2	3.58	3.69	3.66	4.12	3.48	3.47	3.61	3.53
OSGIN1	3.55	3.91	3.66	3.74	3.54	3.47	2.75	3.81
ZCCHC2	3.69	3.59	4.08	4.56	3.21	3.47	4.16	4.02

MYCBP2	2.9	2.7	3.7	4.01	3.25	3.47	4.88	4.41
CCDC181	2.94	3.26	3.26	3.55	2.77	3.47	4.32	4.43
C2orf76	3.55	4.81	3.47	3.56	4.02	3.47	4.79	4.65
CGREF1	3.88	3.35	3.46	3.34	2.84	3.48	3.01	3.32
RGL1	3.33	3.45	3.44	4.19	3.51	3.48	3.72	3.87
CECR2	3.53	3.49	3.63	3.82	3.44	3.48	4.27	3.92
TMEM184A	3.63	3.71	4.17	3.89	3.65	3.48	3.05	3.98
RBM45	3.25	3.46	3.65	4.66	3.51	3.48	4.52	4.03
SNX10	3.46	3.79	4.24	4.29	3.14	3.48	5.29	4.79
DCP1A	4.11	3.84	4.41	4.68	4.17	3.48	5.63	4.81
METTL14	3.14	2.84	3.97	4.48	3.66	3.48	5.27	4.86
POLI	3.9	3.35	3.72	4.06	3.1	3.48	5.87	5.23
PRRT3	5.82	5.48	5.44	5.28	3.13	3.49	2.38	2.95
WBSCR28	5.29	4.72	4.17	4.78	4.01	3.49	3.18	2.98
NEU3	3.76	2.87	3.55	3.81	3.75	3.49	3.52	4.21
ACSL5	3.81	3.16	3.5	3.17	4.22	3.49	4.44	4.21
PLA2G4A	3.34	3.27	3.84	4.5	4.25	3.49	5.9	4.93
GPR37	3.08	3.04	3.07	3.34	3.37	3.5	3.11	3.13
RAB15	4.23	3.8	3.98	3.9	3.64	3.5	3.55	4.22
SOS2	2.99	2.97	3.57	4.29	3.36	3.5	5.77	4.94
DRD4	2.69	3.12	2.37	2.49	3.06	3.51	2.21	2.71
ELMO1	2.81	3.14	2.73	2.7	3.56	3.51	3.11	3.14
FAAP24	3.81	3.32	3.75	3.42	3.35	3.51	3.41	3.15
LOC728392	3.64	3.87	3.64	4.22	3.57	3.51	3.26	3.2
TASP1	2.92	2.77	3.14	3.84	3.82	3.51	4.16	3.59
C18orf25	2.95	2.59	3.5	3.83	3.38	3.51	4.58	4.25
SLC25A16	2.4	1.92	2.87	2.82	3.35	3.51	4.23	4.31
NIPSNAP3A	4.16	4.22	4.79	3.94	3.97	3.51	5.4	4.67
ODF3B	3.81	4.54	3.76	3.74	2.36	3.52	1.78	2.33
SSC4D	5.53	6.63	5.34	5.09	3.66	3.52	2.6	3.18
LOC101930307	3.11	2.21	2.88	3.15	3	3.52	4.29	3.89
LOC642249	2.53	2.32	3.42	3.81	3.33	3.52	4.73	3.96
NAA16	2.94	2.42	2.67	4.11	2.84	3.52	5.78	4.2
C14orf142	4.08	3.5	3.79	4.47	3.64	3.52	5.49	4.55
HDAC9	2.96	3.51	3.29	4.35	4.13	3.52	6.01	4.86
NUBPL	3.58	3.39	3.62	4.43	3.84	3.52	5.34	4.92
LOC389831	3.87	2.45	3.86	4.72	4.19	3.52	5.63	5.03
NPIPB15	3.67	2.3	3.68	4.25	4.02	3.52	6.66	5.2
CRLF1	9.04	9.04	8.28	7.22	3.04	3.53	1.66	3.15
SULT1A1	4.84	4.85	4.85	3.78	3.75	3.53	3.69	3.4
ZBTB44	2.42	2.69	2.94	3.53	3.29	3.53	4.41	3.63
AMOT	3.82	3.88	4	4.26	3.58	3.53	4.03	4.12
CEP76	4.14	2.5	3.49	3.68	4.01	3.53	5.41	4.34
HTR7	2.38	2.55	2.53	2.94	3.75	3.53	4.11	4.42
PITX2	3.41	3.39	3.77	3.5	3.45	3.54	3.8	3.33
PECR	3.92	3.97	3.64	4.23	4.25	3.54	5.25	3.73
FER	2.66	2.32	2.94	4.02	2.49	3.54	5.4	3.92
SLC31A2	3.33	3.44	3.8	3.26	4.53	3.54	4.69	4.22
RAD51AP1	3.1	3.34	3.65	4.01	3.71	3.54	5.89	4.58
SLC5A3	3.51	3.16	4.05	4.48	3.7	3.54	5.94	5.18
TRMT13	3.5	2.77	3.28	3.75	3.29	3.54	6.94	5.89
U2AF1L4	3.05	2.81	2.69	3.11	2.78	3.55	2.57	2.99
DLX6	3.85	3.52	3.68	3.44	3.42	3.55	3.5	3.82
SIRT1	2.77	2.79	3.66	4.38	3.49	3.55	5.11	4.26
SFXN2	3.53	3.43	3.86	3.77	3.99	3.55	4.03	4.27
UTRN	2.76	2.82	3.85	4.76	3.74	3.55	6.22	5.48
NFAT5	3.29	2.68	3.78	4.55	3.56	3.55	6.57	5.71
UBE2D1	3.47	3.41	3.33	3.37	3.57	3.56	5.04	3.94
ADAL	3.73	2.79	3.36	3.74	3.98	3.56	5.03	4.27
CMTR2	3.32	3.04	3.32	4.09	3.49	3.56	5.04	4.68
B3GNT5	3.19	3.05	3.45	4.17	3.62	3.56	6.06	5.17
WDCP	3.5	3.3	3.46	3.6	3.6	3.57	5.02	4.17
TMED7-TICAM2	3.62	3.77	3.8	4.29	3.82	3.57	4.78	4.54
ZFP1	3.72	3.32	4.21	4.83	3.99	3.57	5.93	4.92
CCPG1	3.65	3.41	4.23	4.95	3.7	3.57	6.66	5.16
SAMD5	3.27	3.37	3.99	4.74	3.74	3.57	5.52	5.54
SASH1	2.89	2.45	3.44	3.77	3.58	3.58	4.19	3.83
MARS2	3.01	2.92	3.69	3.16	3.85	3.58	3.94	3.96
ATP6AP1L	3.56	3.55	3.85	4.18	4.02	3.58	5.02	4.67
ZNF226	3.02	3.39	3.3	3.67	4.07	3.58	5.11	4.69
F5	4.59	5.12	4.98	5.77	3.37	3.58	4.52	4.77
KIF3A	3.06	3.08	3.75	4.7	3.74	3.58	5.89	6.06
POU5F1	3.46	3.3	4.26	4.69	3.5	3.59	4.62	3.73
MID2	3.97	3.66	4.51	4.65	3.52	3.59	4.5	4.02
GPC6	3.28	2.94	3.85	3.7	3.65	3.59	4.77	4.54
SUV39H2	3.38	2.83	3.54	3.84	3.72	3.59	5.73	4.88
PRR14L	3.25	2.91	3.77	4.38	3.79	3.59	5.22	5.25
TMEM143	3.16	2.67	3.06	2.61	3.14	3.6	2.55	3.35
VANGL1	3.74	3.77	3.81	3.57	3.41	3.6	3.42	3.55
PARP16	4.27	4.32	4.08	4.13	3.66	3.6	4.11	3.6

TRAF6	3.02	3.31	3.31	3.84	3.5	3.6	4.19	4.01
CNKSR3	5.22	4.77	4.69	4.7	3.74	3.6	3.66	4.02
ESCO1	3.24	3.02	3.27	3.28	3.14	3.6	5.51	4.52
LRRC49	2.86	2.83	3.27	3.34	3.67	3.6	5.08	4.58
FRRS1	3.99	3.35	4.34	4.63	3.66	3.6	5.21	5.14
GCC2	2.28	2.53	2.87	3.65	3.1	3.6	6.55	5.84
TEAD3	3.93	3.91	3.86	4.2	3.54	3.61	2.82	3.36
TTC39B	2.85	2.94	3.38	3.34	3.12	3.61	4.52	3.77
L3MBTL3	3.85	3.44	4.2	4.46	3.59	3.61	4.71	4.06
WDR27	2.7	3.12	3.17	3.75	3.54	3.61	5.09	4.3
DCLRE1C	2.99	2.78	3.39	4.33	3.02	3.61	4.97	4.69
FGF13	2.59	2.42	2.15	2.49	3.61	3.62	3.52	3.37
RAB7B	2.95	3.68	3.55	3.45	2.9	3.62	2.83	3.48
ZNF275	3.8	3.67	3.87	4.18	3.74	3.62	3.86	3.68
IDO1	3.57	3.07	3.21	4.24	4.49	3.62	3.05	4.09
PCCA	3.28	2.91	3.3	3.42	3.18	3.62	4.54	4.17
MTPAP	2.81	2.89	3.11	3.54	3.66	3.62	4.87	4.3
MAGI3	3.22	2.86	3.6	4.11	3.28	3.62	4.75	4.41
AMMECR1	3.35	2.83	3.95	3.4	3.48	3.62	5.08	4.52
C12orf29	3.48	3.08	3.33	3.61	4.15	3.62	6.08	4.75
PCDHB12	4.42	4.18	4.38	4.77	3.69	3.63	4.06	4.48
ZNF37A	3.2	3.23	3.74	4.1	3.57	3.63	7.07	5.66
SBK1	3.9	4.15	3.92	3.74	3.36	3.64	2.67	3.01
CLEC18B	5.82	5.2	4.91	4.02	2.42	3.64	2.85	3.13
PHLPP1	3.83	3.63	3.45	3.72	3.3	3.64	3.04	3.2
COPZ2	4.4	5.38	4.33	5.08	4.6	3.64	2.68	3.7
TXNRD3	3.05	3.66	3.88	3.56	3.56	3.64	4.34	3.79
ZNF140	3.44	3.68	3.62	4.29	3.42	3.64	5.36	4.69
UTP20	3.31	2.8	3.46	4.28	3.73	3.64	5.44	4.82
GLMN	3.57	3.13	3.24	4.01	4.13	3.64	6.16	4.98
XRN1	3.3	2.84	3.47	4.85	2.65	3.64	6.35	5.2
ZNF559	3.74	2.92	3.8	4.16	3.93	3.64	6.19	5.22
PCBD2	2.81	2.4	2.99	3.48	3.11	3.65	4.08	3.32
NECTIN4	2.82	3.17	3.03	2.28	3.58	3.65	3.12	3.33
ARHGEF25	3.79	4.46	3.58	4.14	3.54	3.65	3.5	3.38
ADIG	2.87	3.71	2.94	3.66	4.55	3.65	2.94	3.49
IL18	2.68	3.27	3.2	3.17	4.81	3.65	5.26	3.66
LIN9	3.2	3.14	3.56	2.86	3.4	3.65	4.6	3.91
DUSP16	3.56	3.14	3.79	3.77	3.45	3.65	4.34	4.03
VPS13B	3.18	2.68	3.65	4.72	3.81	3.65	5.4	4.74
SYNJ2BP	2.83	2.87	3.3	3.96	3.86	3.65	5.31	5.02
BAGE2	3.41	2.61	3.85	4.29	3.31	3.65	5.64	5.02
HCN3	3.04	3.35	3.26	3.4	3.4	3.66	3.72	3.77
CRY2	4.05	3.98	3.87	3.64	3.67	3.66	3.31	3.83
SFT2D2	3.08	3.25	4.16	4.02	2.78	3.66	4.95	4.43
HMBOX1	2.91	2.9	3.28	3.92	3.33	3.66	4.73	4.56
FAM200A	3	2.79	3.7	4.48	3.03	3.66	6.16	4.66
ZNF621	2.99	3.15	3.86	3.87	3.53	3.66	5.14	5.23
PYROXD2	2.97	3.24	4.11	4.3	4.07	3.66	4.94	5.36
CLIP1	3.33	3.1	4.04	5.05	3.42	3.66	5.8	5.46
DNAJB4	3.36	3.61	3.45	4.07	3.82	3.66	5.72	5.63
UTP23	2.75	3.13	3.6	4.54	3.55	3.66	6.09	5.86
WDR25	4.47	4.69	3.69	3.15	3.57	3.67	3.13	3.56
CLCN4	3.65	3.73	3.88	3.88	3.53	3.67	4.4	4
RBM15	3.67	3.58	3.68	4.31	3.98	3.67	4.63	4.64
FRMD4B	3	2.74	3.44	3.95	3.89	3.67	5.2	5.08
BACH1	3.31	2.98	3.95	5.24	3.77	3.67	6.96	5.27
ZCCHC10	3.94	4.4	4.09	4.88	4.06	3.67	7.13	5.38
NRGN	4.25	4.01	3.41	3.44	3.35	3.68	3.02	2.74
PIBF1	3.47	3.26	3.92	3.59	3.22	3.68	5.82	4.47
BDH2	3.28	3.33	3.03	3.59	3.49	3.68	4.4	4.55
ZNF783	3.37	3.21	3.86	4.28	3.54	3.68	4.69	4.57
CMTM4	3.6	3.02	3.73	3.72	3.96	3.68	4.46	4.63
LATS1	3.71	3.73	4.54	4.95	3.31	3.68	5.43	5.37
CCDC82	3.85	3.4	3.5	4.01	4.13	3.68	6.77	5.48
DCAF17	3.69	3.09	3.46	4.34	3.76	3.68	6.12	5.82
VGF	3.2	2.45	2.49	2.8	3.35	3.69	3.23	3.08
C19orf68	3.47	3.68	3.44	3.7	3.62	3.69	3.15	3.23
PLCD4	4.15	4.21	4.3	4.06	3.95	3.69	3.86	3.79
CBLB	3.69	3.58	4.36	4.2	3.59	3.69	4.34	4.02
UVRAG	3.41	3.18	3.45	3.58	3.65	3.69	4.65	4.03
EXOC8	3.11	3.02	3.54	3.9	3.62	3.69	4.33	4.42
GSAP	3.46	2.99	3.86	4.02	3.78	3.69	5.35	5.19
ALG6	3.96	3.66	4.23	4.28	4.58	3.69	6.38	5.5
ATF1	3.28	3.16	3.93	4.53	4.24	3.69	6.61	5.79
GORAB	3.76	3.13	4.21	4.76	4.68	3.69	6.85	5.98
DHRSX	3.72	3.33	3.59	2.97	3.51	3.7	2.89	2.71
LOC81691	4.06	3.25	3.37	3.56	3.99	3.7	3.59	3.25
JRK	3.88	4	4.58	3.91	3.93	3.7	4.01	3.74
HAPLN3	5.14	5.15	4.52	4.86	4.88	3.7	3.16	3.88
CENPI	2.6	2.57	3.28	3.97	3.98	3.7	4.78	4.55
SMAD1	4.19	4.37	4.39	4.83	3.79	3.7	5.54	4.74
XPO4	3.04	2.75	3.55	4.3	3.58	3.7	5.9	4.89
CLK4	2.57	3	3.5	3.64	3.19	3.7	5.5	4.94
SCX	2.67	2.87	2.43	2.13	3.31	3.71	3.05	3.15
CENPL	3.26	3.38	3.22	3.3	3.98	3.71	4.62	3.73

SRD5A3	4.76	4.38	4.81	4.82	4.02	3.71	4.1	3.78
SSH2	3.52	2.93	3.06	3.59	3.13	3.71	3.68	4.29
BMPR2	3.1	3.11	3.63	4.51	3.45	3.71	5.42	4.93
C5orf51	2.89	2.67	3.52	4.79	3.74	3.71	7.05	5.81
TBC1D9	2.66	2.84	2.86	3.23	3.66	3.72	4	3.99
AGAP4	3.61	2.88	4.38	5.3	4.08	3.72	6.8	5.46
GABPA	3.71	3.11	3.86	4.89	4.09	3.72	6.98	5.99
SIK1	3.51	3.79	3.74	2.23	4.29	3.73	3.96	2.18
POU3F1	3.71	3.46	3.1	3.14	3.96	3.73	3.01	3.22
LOC391322	4.43	3.54	3.7	3.75	3.75	3.73	4.8	3.83
FBXO16	3.99	4.17	5.12	3.93	4.72	3.73	5.58	4.04
RASA4	3.71	3.98	4.09	3.22	3.63	3.73	3.03	4.33
RPS6KA5	3.15	3.72	3.14	3.99	3.94	3.73	5.32	4.62
GLTSCR1L	3.64	3.17	3.72	3.85	4.13	3.73	4.97	4.75
RELT	4.23	3.77	4.02	3.74	4	3.74	3.32	3.47
FAM122A	3.68	4.12	4.24	4.48	3.44	3.75	4.39	3.82
FBXL7	3.94	3.65	4.06	4.51	3.25	3.75	3.79	4
UHRF1BP1	3.98	3.94	4.03	4.28	3.87	3.75	4.35	4.27
ABCC4	3.36	2.64	3.22	3.25	3.27	3.75	4.19	4.44
LOC102724428	4	3.4	3.59	4.41	3.12	3.75	2.95	4.51
GCH1	3.26	3.96	3.86	4.09	3.52	3.75	4.21	4.59
ELF2	4.01	3.87	4.64	4.77	4.43	3.75	5.65	5.62
CFAP97	3.41	4.13	4.29	4.9	4.42	3.75	7.94	6.79
ZSCAN2	3.51	3.71	3.79	3.54	3.85	3.76	3.19	3.37
OR51B5	3.65	3.45	4.3	4.77	4.16	3.76	3.89	3.38
ZNF488	3.23	3.23	3.05	2.76	3.76	3.76	3.45	3.45
NAAA	3.86	4.23	3.85	3.97	3.96	3.76	4.3	4.16
FNBP1	3.9	3.84	4.14	4.33	3.92	3.76	4.26	4.31
PIGL	4.29	3.25	3.87	4.03	3.51	3.76	5.15	4.9
SLC25A4	3.85	3.89	3.71	3.35	3.79	3.77	3.15	3.18
NCOA1	3.35	2.95	3.72	4.53	3.37	3.77	4.32	4.28
THSD1	2.89	3.3	3.45	3.72	3.94	3.78	4.36	3.34
BRPF3	3.76	3.61	4.56	4.06	3.75	3.78	3.46	3.44
HIC2	3.67	3.89	3.88	3.69	3.72	3.78	3.34	3.64
MARK1	3.15	3.45	3.46	3.98	3.51	3.78	4.21	3.97
SPINT1	2.81	3.63	3.29	3.51	4.65	3.78	3.97	4.04
STON2	2.96	2.85	3.84	5.06	4.15	3.78	5.57	5.47
TLR4	2.77	2.61	3.43	4.21	4.37	3.78	6.89	5.98
LGALS7	4.92	2.44	3.52	3.65	2.96	3.79	2.84	2.41
SPERT	4.69	5.25	4.89	4.37	3.82	3.79	3.01	2.89
SHANK3	3.42	3.13	3.63	3.25	3.76	3.79	3.15	3.52
CSRNP2	3.76	3.47	3.23	3.62	3.79	3.79	3.31	3.6
ATP7B	3.35	3.43	3.56	3.68	3.74	3.79	4.1	4.22
CLEC18A	4.2	6.54	4.23	4.45	4.94	3.79	3.21	4.23
BRMS1L	2.99	2.83	3.84	3.85	4.24	3.79	6.15	4.59
MB21D1	3.81	3.64	3.81	3.75	4.24	3.79	4.82	4.92
MTF1	4.3	4.09	4.25	4.52	4.39	3.79	5.2	4.95
LOC107985082	4.49	4.23	4.7	5.37	4.71	3.79	6.08	5.43
BBIP1	3.46	4.15	3.54	4.3	4.03	3.79	5.59	5.63
LOC107987428	3.72	3.15	3.27	2.81	3.89	3.8	2.71	2.97
RDM1	4.17	4.18	3.05	3.21	4.55	3.8	3.77	3.76
RAB36	3.47	4.29	3.89	3.45	3.37	3.8	3.64	4.04
RTN4IP1	3.7	3.84	4.45	3.72	4.46	3.8	4.1	4.17
RAB21	3.26	3.12	3.86	3.83	4.09	3.8	5.69	5.08
RALGAPA1	2.98	2.66	3.54	4.29	4.01	3.8	6.24	5.39
FAS	3.02	3.1	3.67	4.3	3.51	3.8	6.62	5.54
RLF	4.17	3.99	4.77	5.81	3.83	3.8	7.03	6.18
APH1B	3.92	3.94	3.95	3.81	3.23	3.81	4.05	3.46
PEX3	3.31	3.23	3.67	3.67	2.93	3.81	4.54	3.57
MLKL	2.99	3.35	3.2	3.38	3.55	3.81	4.55	3.92
PDE4DIP	3.63	3.58	3.78	3.99	3.8	3.81	3.68	4.07
HNRNPA1L2	3.58	4.17	4.53	4.81	4.02	3.81	6.22	4.17
GPATCH11	2.64	2.48	3.44	3.78	4.08	3.81	6.33	4.74
CSGALNACT2	3.44	3.36	3.64	5.23	3.54	3.81	6.35	4.74
TNIK	3.64	3.46	4.04	4.63	3.91	3.81	5.1	4.85
AASS	3.03	3.02	3.41	4.43	4.05	3.81	6.17	5.86
C10orf88	2.96	2.92	3.11	3.86	3.62	3.82	4.35	3.92
THAP1	2.76	2.55	2.83	3.44	3.34	3.82	4.72	3.94
PTS	4.22	3.43	2.85	3.34	4.35	3.82	6.62	4.49
DOCK9	3.67	3.15	4.13	4.59	3.51	3.82	5.08	4.84
EDRF1	3.59	2.92	4.15	4.32	3.79	3.82	5.93	5.13
ATP6V1E2	3.47	3.41	3.3	3.47	3.26	3.83	4.02	3.55
GNAZ	3.89	3.62	3.7	3.38	3.74	3.83	3.31	3.56
CSNK1G1	3.44	3.5	3.92	3.61	4.09	3.83	4.46	4.29
TMEM192	3.2	3.14	3.5	3.8	4.13	3.83	4.83	4.48
RBM48	3.91	3.86	4.04	4.31	5.27	3.83	6.28	4.51
NANP	3.17	3.3	3.9	3.93	3.91	3.83	5.4	4.52
FMNL3	4.28	4.68	4.29	4.33	3.9	3.84	4.08	4.14
USP30	4.41	4.4	4.52	3.91	4.34	3.84	4.92	4.31

ANKRD13C	3.63	3.3	3.87	4.08	4.1	3.84	5.78	4.68
SLC25A51	2.7	3.86	3.71	3.38	4.17	3.84	4.68	4.77
PRELID2	5.01	5.41	6.26	5.24	4.3	3.84	4.88	4.9
ING3	3.91	3.81	3.91	3.77	4.08	3.84	6.46	5.42
KIAA0586	3.14	2.76	3.58	4.76	4.1	3.84	6.5	5.44
OTUD4	3.18	2.63	3.79	4.8	3.8	3.84	6.99	5.82
PTPRJ	3.61	3.46	3.23	3.77	3.28	3.85	3.54	3.69
C1orf56	4.29	4.93	4.97	4.8	4.03	3.85	3.81	3.74
SIX1	3.95	4.1	4.54	4.47	3.69	3.85	3.34	3.8
UST	3.49	3.59	3.55	3.98	3.32	3.85	3.77	3.82
SLC6A9	3.85	3.94	3.23	2.92	3.51	3.85	3.44	4.1
VPS54	3.67	3.39	3.97	4.59	3.48	3.85	6.44	5.35
LYRM7	2.74	2.52	3.3	4.27	3.41	3.85	7.36	5.45
SCML1	2.83	2.96	3.57	3.58	3.83	3.85	6.77	5.47
ZNF737	3.66	3.62	4.18	5.03	3.74	3.85	6.12	5.74
GSDMB	4.13	4.63	4.85	6.05	3.6	3.85	6.11	5.97
PTAFR	3.8	3.44	3.38	3.91	4.04	3.86	3.49	3.75
TMEM25	5.08	4.9	5.62	4.91	3.56	3.86	3.4	3.87
RFFL	4.2	4.29	4.65	4.98	4.01	3.86	4.77	4.75
NUDT6	4.09	2.78	3.45	3.16	3.15	3.87	4.17	2.55
ADAMTS7	3.43	3.39	3.16	3.4	3.54	3.87	2.9	3.15
RAP1GAP2	3.28	3.62	3.8	3.42	4.02	3.87	3.47	3.28
PKNOX1	4.67	3.94	4.62	4.61	4.26	3.87	4.34	3.96
BTRC	3.72	4.15	4.01	4.19	4.01	3.87	4.97	4.21
ATL3	3.66	3.91	4.06	4.43	4.14	3.87	4.63	4.46
CD3EAP	2.84	2.93	2.96	3.27	4.17	3.87	3.41	4.49
LRR1	4.66	3.63	4.13	4.28	4.64	3.87	4.65	4.82
PTPRZ1	3.38	3.33	3.79	4.58	3.26	3.87	4.46	4.93
ANKRD49	3.26	4.16	4.44	4.48	3.63	3.87	5.87	5.03
ZHX1	3.26	3.07	3.5	4.28	3.92	3.87	6.46	5.44
ZNF43	3.07	3.22	3.45	4.56	3.62	3.87	5.56	5.53
SMAD7	3.58	3.64	3.48	3.42	4.27	3.88	3.54	4.12
OSBPL7	3.01	3.14	3.36	4.18	3.56	3.88	4.29	4.18
PAMR1	2.95	3.12	3.27	3.34	4.71	3.88	3.72	4.32
UVSSA	3.59	3.05	3.36	4.58	3.12	3.88	5.27	4.55
SCRN3	3.32	3.38	3.89	4.79	4.24	3.88	6.09	5.21
SKA3	3.92	3.5	4.41	4.36	4.35	3.88	6.42	5.24
ZNF486	3.44	3.53	3.69	4.92	4.03	3.88	6.39	5.37
PAQR8	3.76	3.97	3.8	4.08	3.8	3.89	3.95	3.95
MAP7	3.8	3.46	3.74	3.57	4.58	3.89	4.19	4.02
SCAMP5	4.91	4.17	4.4	4.21	4.62	3.89	4.02	4.87
ERI2	3.37	2.76	3.17	4.33	3.92	3.89	6.23	5.4
NOS1	3.29	3.49	3.27	3.48	3.86	3.9	4.01	3.83
PDK1	5.38	4.35	5.1	5.09	3.36	3.9	5.99	4.08
LYSMD1	3.72	3.64	4.12	4	4.45	3.9	4.69	4.42
LOC100996763	3.76	3.39	2.85	3.59	3.31	3.9	4.93	4.57
PPP1R3B	4.63	3.89	4.17	4.91	4.28	3.9	5.1	4.9
RMI1	4.57	3.67	4.45	5.22	4.01	3.9	7.45	5.98
NATD1	3.9	3.77	3.65	3.55	3.49	3.91	3.27	3.76
SLC35D1	2.99	2.7	3.15	3.76	3.94	3.91	4.7	4.17
DPH3	3.32	3.15	3.57	3.79	4.17	3.91	5.87	4.91
NECTIN3	4.04	3.78	5.07	4.88	3.78	3.91	5.86	5.19
ATXN7L1	4.15	3.38	4.57	4.53	3.86	3.91	5.05	5.21
ZNF248	3.38	3.31	3.85	4.88	3.94	3.91	5.67	6.23
SLX4	3.53	3.52	3.52	3.56	3.75	3.92	3.37	3.39
CCDC134	3.35	4.02	3.95	4.22	3.9	3.92	3.85	3.91
SH3RF1	3.98	3.91	3.94	4.32	3.83	3.92	4.55	4.31
PPARGC1B	3.98	3.12	3.75	3.95	4.46	3.92	4.09	4.47
KLHL42	3.75	3.21	4.07	4.51	3.98	3.92	5.06	4.62
EFNA5	4.15	3.69	4.18	4.45	3.92	3.92	4.06	4.65
GABRE	2.89	2.91	3.27	4.16	3.59	3.92	5.75	5.29
NHS	3.92	3.06	4.2	5.3	3.47	3.92	6.47	5.63
BBS10	3.79	3.69	4.34	4.78	3.6	3.92	6.25	5.77
ZFP62	4.13	3.86	5.06	6.3	3.99	3.92	7.64	5.98
ZNF658	3.71	3.39	4.04	5.56	4.06	3.92	6.98	6.11
PANX2	4.1	3.9	4.13	3.7	3.4	3.93	2.6	3.18
PTCHD4	4.46	4.38	4.51	4.62	4.25	3.93	4.71	4.67
FAM160B1	3.3	3.19	3.65	4.57	4.3	3.93	5.66	5.26
ADAMTSL4	3.67	3.92	3.76	4.3	2.86	3.94	2.98	3.36
PASK	3.76	3.34	3.41	3.95	3.01	3.94	4.14	3.72
ZSCAN32	4.06	3.93	3.76	4.46	4.24	3.94	4.61	4.65
PDE7B	3.19	2.71	3.22	3.8	4.03	3.94	4.83	4.8
SERPINI1	4.31	4.1	4.04	4.91	3.1	3.94	5.61	5.06
LRP6	3.73	3.25	3.79	4.86	3.92	3.94	5.81	5.29
NBPF11	3.2	3.16	3.52	3.65	3.72	3.94	5.29	5.33
PTPRS	5.09	5.04	4.9	4.76	3.39	3.95	2.94	3.22
AZIN2	4.26	4.2	4.32	4.1	3.61	3.95	3.57	3.8
GAL3ST4	4.06	4.02	3.75	4.14	3.99	3.95	3.92	3.91
ARHGAP18	3.23	2.75	4.02	4.62	3.92	3.95	6.27	5.02
ETFDH	3.6	3.52	3.63	3.67	4.98	3.95	6.56	5.2
ZNF92	4.73	4.93	5	5.6	4.1	3.95	6.18	5.53
GABBR1	3.11	2.92	3.11	4.15	3.53	3.95	6.23	5.95

MT1E	3.66	3.59	2.93	2.85	1.82	3.96	1.35	2.72
MMP28	2.55	3.2	3.15	2.49	3.78	3.96	3.26	3.39
HINFP	4.24	3.93	4.43	4.24	4.52	3.96	4.34	4.07
SLC16A13	4.46	4.14	3.91	3.97	4.18	3.96	3.1	4.52
COX18	3.44	3.65	3.73	4.37	4.48	3.96	4.99	4.55
ZFYVE26	3.93	3.83	4.15	4.39	4.37	3.96	4.77	5.12
SETD7	3.06	3.03	3.59	4.42	4.06	3.96	5.75	5.21
ZNF341	2.84	2.47	2.57	2.24	3.96	3.97	3.35	3.47
MEIS2	4.04	3.98	4.1	5.16	3.32	3.97	4.49	4.05
SMIM14	3.33	3.41	3.34	4.06	3.77	3.97	4.6	4.48
CEP164	3.91	3.75	3.72	4.28	4.4	3.97	4.18	4.51
KATNBL1	4	3.55	4.67	4.64	3.78	3.97	5.92	4.88
RAP2A	3.57	3.64	3.66	4.51	4.01	3.97	5.48	5.16
DPY19L3	3.39	2.89	3.74	4.4	3.86	3.97	6.5	5.17
MOSPD1	3.72	3.49	4.29	3.83	4.6	3.97	6.74	5.23
STX17	3.61	3.13	4.04	4.91	3.9	3.97	5.98	5.36
FMO4	4.12	3.33	4.4	4.29	4.03	3.97	6.61	5.62
ZNF846	3.78	3.81	4.39	5.62	4.59	3.97	5.63	5.96
KIAA1324	3.62	4.96	3.42	3.92	3.35	3.98	2.66	4.13
CCDC112	3.46	3.63	3.95	4.26	4.34	3.98	6.01	5.16
BNIP2	3.43	3.56	3.92	4.5	4.24	3.98	6.85	6
LY96	3.96	3.63	3.3	2.48	4.6	3.99	5.12	4.03
IRAK4	3.33	3.33	3.68	4.51	3.77	3.99	5.94	4.78
R3HCC1L	3.71	3.06	3.68	4.08	3.67	3.99	5.96	5.49
ZNF300	3.64	3.21	4.07	5.28	4.43	3.99	7.96	6.4
RICTOR	3.5	3.47	4.09	4.82	4.32	3.99	7.88	6.69
VASN	6.24	6.64	5.96	5.79	3.91	4	2.68	2.88
USP13	3.51	2.85	3.23	3.9	3.27	4	3.76	3.62
MOK	4.14	3.87	3.37	4.04	4.22	4	4.72	4.02
ZNF473	3.83	3.82	4.32	4.48	3.97	4	4.31	4.11
ITPRIP	3.75	4.05	3.68	4.2	4.16	4	3.96	4.21
SH3D21	5.07	5.21	4.9	5.99	3.81	4	5.78	4.24
FRMD4A	4.26	3.8	3.9	4.46	4.22	4	4.54	4.53
LIN7C	2.53	2.98	3.06	3.96	4.08	4	6.3	5.44
RAB11FIP4	4.1	4.14	4.2	4.22	3.98	4.01	3.93	4.21
ZRSR2	3.69	3.38	4.47	4.38	4.29	4.01	4.79	4.8
LYSMD3	3.83	3.31	3.55	4.2	3.87	4.01	6.51	5.8
IL17RA	4.03	3.77	3.84	4.2	3.52	4.02	3.48	3.74
FIG4	3.47	2.97	3.61	3.64	4.35	4.02	5.15	4.73
MTF2	4.53	3.86	5.09	5.88	4.5	4.02	6.72	5.45
ASB16	3.76	3.2	3.55	3.77	3.52	4.03	3.8	3.48
UBA7	6.57	6.83	5.93	5.53	4.48	4.03	3.78	3.59
APAF1	3.47	2.99	3.42	4.73	3.74	4.03	5.64	4.76
KIF20B	3.42	2.91	3.61	3.33	4.43	4.03	6.68	5.77
DNAJB5	3.8	3.99	3.63	3.36	3.64	4.04	3.09	3.35
RAD18	3.41	2.6	3.95	3.77	3.57	4.04	5.12	4.53
LIMCH1	4.83	4.17	5.17	5.51	4.77	4.04	5.75	4.92
GPATCH2	3.51	3.75	4.16	4.59	3.83	4.04	5.69	5.61
SPIN2B	3.74	3.74	3.3	4.33	4.18	4.05	4.35	4.32
BEX2	3.21	2.38	2.45	3.17	3.32	4.05	3.76	4.49
TPM2	4.28	3.3	3.74	4.28	3.36	4.05	5.06	4.71
CHD7	3.76	3.5	4.21	4.5	3.7	4.05	4.96	4.95
RAB3B	4.37	4.23	4.94	5.57	4.57	4.05	5.19	4.98
YAF2	4.62	3.01	3.95	4.31	4.17	4.05	5.35	5.03
CBL	4.22	3.77	4.34	4.54	4.1	4.05	4.6	5.13
ZNF562	3.63	3.53	3.88	4.47	4.21	4.05	5.72	5.22
TBC1D23	3.97	4.02	4.31	5.49	4.47	4.05	7.95	6
CARNMT1	3.36	3.44	4.11	5.31	5.18	4.05	8.6	6.77
THAP8	5.08	4.55	4.03	3.86	4.1	4.06	3.71	3.79
SUPT3H	3.25	3.62	3.06	3.73	4.58	4.06	5.17	4.58
CNTRL	3.18	3.4	4.2	4.99	3.7	4.06	7.83	6.57
FAM110C	3.97	3.6	3.43	4.14	4.54	4.07	4.04	3.58
VRK2	4.87	3.88	4.86	5.26	4.25	4.07	6.65	5.65
ZNF302	4.45	3.27	5.22	5.46	4	4.07	8.76	5.84
ZNRF3	4.13	4.08	4.04	4.05	3.9	4.08	3.63	3.51
WDR62	4	3.38	3.33	3.8	4.57	4.08	3.72	3.64
SYNGR3	3.77	3.99	3.09	3.47	4.05	4.08	2.88	3.82
EYA4	3.53	3.44	4.24	4.55	4.06	4.08	6.56	5.08
PLPPR1	3.63	3.61	3.95	4.83	3.69	4.08	4.83	5.2
MED31	3.85	4.71	3.93	4.03	4.94	4.09	3.8	3.41
C2CD2	4.14	2.95	3.33	3.44	3.44	4.09	3.95	3.6
ZNF497	3.65	3.73	3.34	3.13	3.99	4.09	3.3	3.61
LYN	3.07	3.38	3.33	3.68	3.84	4.09	4.78	3.86
NKIRAS1	3.3	2.89	3.23	3.8	3.83	4.09	5.63	4.41
GIPR	3.06	2.79	3.84	3.54	3.81	4.1	5.36	4.49
LOC730268	3.62	3.6	2.94	2.83	4.02	4.1	3.87	4.51
SAGE1	4.12	3.25	3.46	4.11	4.72	4.1	6.91	5.23
DTNA	3.65	3.84	4.28	4.7	4.75	4.1	6.06	5.74
RNFT1	3.9	3.84	3.86	4.14	4.49	4.1	5.95	5.93
CTIF	3.68	4.17	4.27	3.74	4.11	4.11	3.51	3.73
EVL	4.4	4.67	4.76	4.5	4.3	4.11	3.51	4.09
FAM210A	3.77	3.4	3.47	3.91	4.41	4.11	5.33	4.41
PTGER4	4.31	3.99	4.24	4.37	3.73	4.11	4.21	4.51
SMAD4	3.94	3.71	4.28	4.38	4.47	4.11	5.06	4.57
DEPDC1	2.56	2.65	2.79	3.13	4.12	4.11	7.17	5.6
KDM6A	4.65	4.22	4.85	4.88	4.52	4.11	5.86	6.1

DNAAF2	3.83	3.93	3.78	4.19	4.56	4.12	5.34	5.03
MTRF1	2.22	2.82	2.97	3.83	4.52	4.12	8.02	5.27
ZNF397	4.92	3.99	4.65	5.56	4.51	4.12	6.84	5.43
ZC2HC1A	3.52	3.38	3.7	5	4.3	4.12	5.49	5.44
PRPF39	3.75	3.54	3.77	4.77	4.36	4.12	8.15	5.77
SNX29	3.48	3.79	3.36	3.72	3.96	4.13	3.31	3.94
FAM101B	3.73	4.17	4.02	3.91	4.31	4.13	3.77	4.01
PTP4A3	5.03	5.98	4.8	5.05	4.14	4.13	3.54	4.06
PAX9	4.02	4.55	3.99	4.46	4.06	4.13	3.55	4.06
PASD1	5.05	4.82	4.97	4.62	3.98	4.13	4.12	4.19
EME2	3.22	3.15	2.84	3.67	4.31	4.13	4.22	4.21
PTPN3	4.52	4.26	4.57	5.05	4.65	4.13	5	4.75
ERCC8	4.05	3.98	3.81	4.73	4.72	4.13	5.52	5.39
ARSK	3.92	4	4.35	5.15	4.43	4.13	5.99	5.45
RIOK2	5.97	5.46	6.36	5.71	4.74	4.13	6.77	6.16
SYT1	3.87	3.62	4.25	4.92	4.21	4.13	6.14	6.33
FAM72C	3.39	3.22	2.18	3.55	3.84	4.14	5.39	2.62
SPATA5L1	4.32	3.76	3.92	3.5	4.47	4.14	4.45	4.22
AP1S3	3.64	3.31	3.75	4.23	3.95	4.14	5.93	5.26
NUDT12	4.31	4.59	4.1	4.84	4.37	4.14	6.93	5.68
PHC3	4	3.21	4.6	6.71	4.39	4.14	7.71	6.48
IQCC	4.59	4.42	3.87	3.76	3.01	4.15	3.99	4.21
RYBP	3.97	4.12	4.86	5.51	4.21	4.15	6.1	5.38
MRE11A	3.43	3.24	4.4	4.69	4.22	4.15	7.51	5.89
KBTBD7	4.23	4.35	4.6	4.63	4.88	4.16	4.68	4.47
CCNO	5.08	3.99	4.65	4.45	3.88	4.16	3.16	4.92
SAMD9	7.17	7.64	8.2	8.45	4.11	4.16	7.2	5.79
UNKL	3.92	3.87	3.71	4.03	4.22	4.17	4.21	3.8
CHGB	4.29	3.81	4.15	4.67	4.02	4.17	3.71	3.86
ZNF862	4.73	4.52	4.61	5.09	4.25	4.17	4.81	4.57
SHOX2	4.76	4.41	4.38	5.51	4.18	4.17	4.72	4.68
RP2	3.1	3.13	4.4	4.6	4.4	4.17	6.77	6.14
ATP2B1	4.21	4.16	5.24	5.91	4.81	4.17	7.84	7.02
KLF13	3.49	3.75	3.39	3.69	4.44	4.18	4.12	3.92
B3GNT3	3.96	4.89	4.19	4.84	4.08	4.18	3.82	4.19
SLC35A3	3.76	3.31	4.2	4.43	3.48	4.18	6.91	5.26
LIN52	3.8	4.19	3.71	5.48	4.86	4.18	5.74	5.44
UBE2W	4.42	4.19	3.89	5.11	4.55	4.18	6.14	5.57
NEK11	4.03	4.27	4.39	4.67	3.71	4.18	6.97	6.5
TICRR	3.69	3.46	3.65	3.88	3.54	4.19	4.16	3.76
PRKCA	4.49	3.72	3.64	3.93	3.81	4.19	4.4	4.24
EDA2R	2.52	2.58	2.51	3.67	4.37	4.19	5.77	4.64
SPDYE16	2.48	3.34	3.59	4.06	4.24	4.19	4.67	4.84
TANC2	4.85	4.6	5.16	5.17	4.36	4.19	5	4.91
FCHO2	3.18	3.23	3.58	4.79	4.51	4.19	6.23	5.58
RFX3	4.35	4	4.82	5.64	5	4.19	6.99	6.39
EVC	3.77	3.96	3.84	4.46	3.77	4.2	3.97	4.11
LYSMD4	4.26	3.48	3.78	3.62	4.01	4.2	4.62	4.47
KLRC2	3.62	4.65	5.17	4.48	4.24	4.2	5.21	4.58
ABL2	4.41	3.93	4.39	4.63	4.22	4.2	5.16	4.75
NCBP3	3.89	4.52	4.79	4.77	4.58	4.2	5.46	5.09
LRRCC1	4.39	5.1	4.47	4.35	4.41	4.2	6.81	5.1
RFX7	3.46	3.39	4.29	5.38	3.92	4.2	6.07	5.53
SH2D4A	4.87	4.4	4.82	4.95	4.91	4.21	5.29	4.43
JADE1	3.09	3.14	3.83	3.76	4.28	4.21	5.4	4.81
SNX27	4.26	4.4	4.87	5.42	4.8	4.21	5.51	4.94
ZNF12	4.35	4.24	4.93	5.45	4.11	4.21	7.26	5.89
ZIC2	4.53	4.44	4.34	4.83	4.38	4.22	3.38	3.23
CASP3	4.62	3.89	4.61	4.68	4.33	4.22	7.06	4.79
FAXDC2	3.88	4	3.76	4.29	4.34	4.22	3.93	4.98
CMTM1	4.64	3.74	4.17	6.14	3.93	4.22	4.06	5.15
RHOJ	3.75	3.39	4.41	4.43	3.86	4.22	6.11	5.16
KAT2B	3.58	2.97	3.84	4.01	3.99	4.22	5.82	5.35
ADAM22	4.04	4.1	4.4	5.29	5.05	4.22	5.78	5.38
SLC38A6	4.26	3.99	4.18	3.98	4.68	4.22	5.77	5.59
TRIM23	4.1	3.39	4.4	4.89	4.3	4.22	7.77	6.15
ANKRD12	3.34	3.81	4.2	4.44	4.1	4.22	6.82	6.42
PIGZ	4.04	4.03	4.03	4.64	3.48	4.23	3.62	4.11
HIST2H4A	6.82	7.23	6.62	6.02	3.95	4.23	5.31	4.5
HIST2H4B	6.82	7.23	6.62	6.02	3.95	4.23	5.31	4.5
CGRRF1	4.39	3.44	4.38	4.77	4.59	4.23	5.47	5.07
PRICKLE4	4.36	4.9	4.36	5.74	4.35	4.23	5.12	5.28
ATP11C	3.99	3.78	4.91	4.82	4.33	4.23	7.87	6.46
ZNF789	5.67	4.88	5.26	6.88	4.85	4.23	6.03	6.79
APOL1	4.72	4.85	4.57	4.8	3.7	4.24	3.51	3.68
MST1	3.56	2.99	3.85	4.1	3.42	4.24	3.8	3.83
MXRA8	3.25	3.1	3.41	3.11	3.29	4.24	4.37	3.91
CDC37L1	3.55	3.77	3.87	4.45	4.41	4.24	5.78	5.53
FAM72D	3.45	2.74	5.53	4.85	3.88	4.24	5.34	5.8
C2CD5	4.21	3.67	4.57	5.6	4.62	4.24	7.55	6.68
TLE6	4.78	4.66	4.07	4.01	3.62	4.25	3.29	3.67
RSPH3	4.17	3.6	4.37	3.93	4.22	4.25	4.24	4.34
TSPAN33	4.3	5.07	4.22	4.18	5.03	4.25	4.33	4.46
ZNF587	4.09	3.85	4.44	5.06	4.14	4.25	6.13	5.98
DGKQ	4.09	4.47	4	4.01	3.46	4.26	3.11	3.54
TPCN2	3.77	3.55	3.73	3.75	4.15	4.26	3.93	4.39
CPA4	3.71	3.9	4.31	4.61	4.67	4.26	5.25	4.59
CGNL1	4.64	5.14	5.14	5.2	4.05	4.26	4.63	4.89
KIAA1804	3.12	3.33	3.85	4.49	4.08	4.26	5.08	4.92

TRAF3IP1	3.86	3.7	4.72	4.68	4.46	4.26	5.17	5.18
SLC17A5	4.21	4.16	4.45	4.7	4.47	4.26	5.31	5.34
CHEK1	4.67	3.95	3.97	4.57	4.25	4.26	5.6	5.45
MEGF6	5.24	5.07	5.08	5.08	3.88	4.27	3.93	4.46
ZNF318	4.02	3.78	4.7	4.94	4.34	4.27	5.14	4.7
TCP11L2	3.68	3.16	4.43	4.32	3.99	4.27	5.56	4.99
LRRC23	5.33	4.86	5.93	5.77	4.54	4.27	5.4	5.15
PGGT1B	3.45	3	3.61	4.32	4.28	4.27	5.92	5.48
CDH8	3.91	3.93	4.17	4.37	4.49	4.27	6.32	5.48
VPS50	4.31	4.75	5.22	5.5	5.38	4.27	8.65	7.16
SCYL3	3.59	3.25	3.86	4.64	4.44	4.28	5.92	4.53
FABP6	4.28	4.17	3.41	3.52	3.2	4.28	4.25	4.75
GTPBP8	4.02	3.48	4.05	3.55	4.94	4.28	5.95	5.18
ZNF800	4.06	3.78	4.79	5.28	4.37	4.28	5.88	5.89
ABCC10	4.13	4.52	3.73	3.7	4.5	4.29	4.25	4.08
ST7	4.01	3.87	4.03	3.75	3.8	4.29	4.35	4.23
HHAT	4.7	3.84	4.52	4.3	4.3	4.29	4.45	4.4
BDH1	3.65	3.54	4.11	4.25	4.23	4.29	4.36	4.76
ZNF426	3.58	3.54	4.39	4.48	4.47	4.29	5.58	4.88
C12orf4	3.81	3.59	4.75	4.85	4.98	4.29	6.85	5.71
PRDM8	3.3	3.11	3.85	4.01	3.93	4.3	4.01	4.23
SLC25A43	4.53	3.64	3.9	4.64	4.48	4.3	4.4	4.55
CCDC93	3.82	3.93	3.97	4.51	4.73	4.3	5.44	5.29
LRCH1	4.45	4.37	5.22	5.16	4.63	4.3	5.52	5.44
CDK3	3.88	3.48	4.73	4	3.76	4.31	4.13	4.25
STIM2	3.96	4.16	4.59	4.89	4.24	4.31	5.64	5.1
DNAJB14	3.86	3.16	4.45	4.13	3.33	4.31	5.99	5.88
TRNT1	3.63	3.62	4.4	4.85	4.45	4.31	7.18	6.09
BMT2	4.64	4.51	4.98	5.53	4.24	4.31	6.6	6.12
ARL15	4.5	4.8	5.14	5.59	4.89	4.31	7.01	6.13
MCTS1	3.91	3.92	3.93	4.29	4.12	4.32	5.33	4.79
CWC22	3.9	4.6	4.67	5.29	4.4	4.32	6.73	5.66
UBR1	4	3.55	4.7	4.82	4.8	4.32	6.75	6.04
ZNF83	3.71	4.08	4.44	4.68	4.21	4.32	8.3	6.84
ATP23	3.37	4.03	3.28	3.83	3.85	4.33	4.01	4.2
C12orf73	3.83	4.07	3.7	4.1	4.27	4.33	5.03	4.55
PLA2G12A	3.52	3.11	4.13	3.84	4.12	4.33	5.87	4.72
LNX2	4.94	4.03	5.16	5.49	4.95	4.33	5.52	5.55
SPTY2D1	4.3	3.71	4.7	5.16	4.54	4.33	6.23	5.98
SRGAP2B	4.1	3.38	4.59	5.47	4.44	4.33	7.92	6.68
ZZEF1	4.15	3.84	4.08	4.09	4.16	4.34	4.27	4.19
BUB1B-PAK6	3.88	4.69	3.23	4.32	3.72	4.34	4.34	4.87
CDC7	4.26	3.38	3.93	4.89	4.12	4.34	7.08	5.26
NOC3L	3.5	3.63	4.91	4.68	4.71	4.34	7.43	5.9
MTMR6	4.65	4.15	4.57	5.81	5.23	4.34	6.96	6.18
ARHGAP11A	3.7	3.12	3.85	4.04	4.39	4.34	6.98	6.73
FKTN	3.74	3.46	4.24	5.04	3.95	4.35	7.24	5.82
KIF5C	3.13	3.23	4.12	4.19	4.41	4.35	6.43	5.83
C1orf52	4.57	4.33	4.89	4.91	4.71	4.36	6.32	5.01
PARP8	4.38	4.73	4.83	5.03	4.51	4.36	5.71	5.11
CFL2	3.71	4.01	3.98	4.26	5.07	4.36	6.31	5.29
PEAK1	4.41	4.04	4.7	5.33	4.55	4.36	6.21	5.45
PPM1D	4.12	3.4	3.72	3.92	4.63	4.37	5.3	4.56
NOXA1	4.67	4.62	4.34	3.84	4.14	4.38	4.24	4.15
C6orf223	2.74	3.85	2.63	3.16	3.59	4.38	3.23	4.36
NCOA2	4.03	3.49	4.41	5.46	4.1	4.38	6.38	5.6
ELMOD2	4.16	3.83	4.25	5.02	4.12	4.38	6.29	5.85
SLC27A5	3.97	4.67	3.57	4.09	4.32	4.39	3.42	4
CIART	4.56	4.44	3.82	4.31	4.57	4.39	3.64	4.93
TRIM66	3.73	3.46	3.93	4.5	4.36	4.39	6.37	5.77
ATG5	4.64	4.64	4.41	5.23	4.72	4.39	7.61	5.98
FBXO42	5.03	5.3	4.82	4.8	4.44	4.4	4.62	4.27
CSAD	3.9	4.08	3.97	4.47	4.43	4.4	6.15	5.27
RWDD3	3.25	3.33	3.65	4.11	3.58	4.4	6.55	5.57
RAPGEF5	4.56	4.29	4.84	5.59	4.62	4.4	6.25	5.6
COQ3	4.34	4.46	4.73	5.08	3.89	4.41	5.37	4.77
RAP1GDS1	4.38	4.05	4.5	4.89	4.74	4.41	5.28	5.43
WDHD1	4.3	3.56	4.8	5.4	4.28	4.41	7.24	5.99
KIAA1033	3.35	3.33	4.05	4.78	3.97	4.41	7.4	6.18
RPIA	3.66	3.4	3.96	4.24	5.25	4.42	4.95	4.41
MAGEA10	5.39	5.05	5.17	5.37	5.26	4.42	4.94	4.43
SLC12A6	3.7	3.57	4.08	4.62	4.31	4.42	5.04	4.91
LOC105373132	4.26	3.6	4.67	5.47	3.98	4.42	6.09	5.13
LRTOMT	4.31	4.53	5.51	5.41	4.38	4.42	4.8	5.19
EOGT	3.76	3.25	4.24	5.58	4.47	4.42	8.29	5.81
SPRTN	4.09	3.39	4.05	5.19	4.87	4.42	6.31	6.05
ARHGAP12	3.7	3.72	4.51	5.64	4.34	4.42	7.39	6.85
S1PR3	4.68	4.69	4.97	5.02	4.09	4.43	4.18	4.07
TMEM241	4.07	4.23	4.27	3.75	4.17	4.43	4.47	4.31
COQ10A	4.13	3.11	4.64	4.09	4.09	4.43	4.11	4.46
PHF7	3.08	3.32	2.6	3.12	4.1	4.43	4.67	4.7
OSBPL11	3.51	3.66	3.87	4.63	4.59	4.43	5.35	5.1
KBTBD6	3.71	2.8	4.13	4.47	4.95	4.43	5.86	5.19

LYRM5	4.57	3.48	4.72	4.06	5.78	4.43	5.77	5.74
PPIC	3.83	3.63	3.67	4.16	3.86	4.43	5.51	5.83
PPIL4	5.24	5.36	4.69	5.72	5.65	4.43	7.39	6.7
SH2D3A	4.55	5.09	4.79	4.44	5.25	4.44	4.4	4.77
PEX13	3.57	3.13	4.08	4.14	4.1	4.44	6.29	5.1
DEPDC1B	4.26	3.16	3.95	4.27	4.73	4.44	8.28	6
LYRM2	3.81	3.23	4.32	5.29	4.92	4.44	7.51	6.17
TRIP4	4.59	4.48	4.67	5.34	4.44	4.44	5.58	6.19
GPR89B	2.91	2.61	3.49	2.54	3.88	4.44	4.41	6.47
ASPM	3.51	3.3	3.77	4.14	4.29	4.44	7.77	6.94
ZNF48	5.74	5.89	4.97	5.03	4.56	4.45	3.29	3.7
CCDC102A	4.9	4.66	4.51	4.42	3.91	4.45	3.38	3.75
FAM156A	4.4	5.05	3.22	3.55	4.1	4.45	3.1	3.9
DEPDC5	3.76	3.53	3.7	3.85	4.33	4.45	4.76	4.11
VTI1A	3.79	3.71	4.18	4.37	4.37	4.45	5.37	4.71
AMN1	2.93	3.05	2.95	3.05	4.11	4.45	5.43	4.9
FAM73A	3.22	3.48	3.82	4.82	4.28	4.45	7.59	6.19
INTS2	3.13	3.43	4.14	5.46	3.99	4.45	7.31	6.42
GALNT14	4.21	4.2	4.42	4.67	4.5	4.46	4.31	4.42
ANKRD1	2.58	2.24	3.12	3.82	4.85	4.46	5.52	4.7
ZNF589	4.66	4.9	5.05	5.1	4.52	4.46	5.07	5.06
SRR	5.16	3.78	4.6	5.22	3.79	4.46	5.25	5.08
MGA	3.68	3.72	4.95	5.56	5.09	4.46	6.52	5.9
WDR36	4.43	3.66	4.49	5.44	4.98	4.46	7.76	6.53
TRAPPC8	3.64	3.75	4.47	5	4.22	4.46	7.46	6.54
STK38L	4.71	3.91	5.79	6.85	5.21	4.46	8.5	7.42
SAMD10	4.83	3.92	4.38	4.3	3.85	4.47	3.52	3.85
KIF7	5.05	5.01	5.11	4.93	4.22	4.47	4.17	4.04
FAM72A	2.15	3.17	2.08	2.38	5.21	4.47	6.06	4.23
C1orf74	5.32	4.76	4.8	5.2	4.98	4.47	4.71	4.66
LRRC37A2	4.86	4.05	4.97	5.72	5.23	4.47	6.66	6.52
RLIM	3.53	3.34	4.56	5.79	4.27	4.47	7.31	6.52
PRDM11	6.43	5.66	5.4	4.79	4.57	4.48	3.37	4.19
UNC13B	3.87	4.01	3.97	3.89	4.54	4.48	4.68	4.78
TRIM34	5.19	5.17	5.62	5.62	4.14	4.48	6.67	4.89
ZCCHC8	5.04	3.95	4.88	5.16	4.82	4.48	5.89	5.19
CACNB1	5.08	4.57	4.66	5.72	5.19	4.48	6.06	5.37
TIGD1	4.52	3.87	4.47	4.9	4.89	4.48	6.92	5.99
STAG1	4	3.51	5.11	5.38	5.07	4.48	7.79	6.13
CDC42BPA	3.42	2.96	4.13	5.27	4.36	4.48	7.02	6.49
PEX11G	3.12	4.21	3.45	4.23	4.29	4.49	2.63	2.6
NFKBIE	5.36	5.29	4.48	4.17	5.63	4.49	3.89	3.83
LIN7B	4.81	4.42	3.57	4.42	4.82	4.49	4.52	4.2
PLD6	3.44	3.38	3.07	3.46	4.59	4.49	4.78	4.54
DFFB	4.09	3.36	3.56	3.98	3.69	4.49	5.04	5.01
CACNG7	5.53	6.42	5.7	5.16	4.15	4.49	3.4	5.06
EXD2	4.39	5.02	5.13	4.7	5.5	4.49	5.84	5.36
PAK3	3.39	3.95	4.77	4.92	4.02	4.49	4.94	5.71
MTMR10	4.08	3.7	4.64	4.85	4.91	4.49	6.21	6.04
KIAA2026	3.96	3.77	4.17	5.77	3.92	4.49	8.3	7.4
IRX5	4	3.79	3.96	3.61	4.35	4.5	3.79	2.99
APOL2	5.33	4.66	4.38	5.39	4.49	4.5	4.38	4.67
BLOC1S5	3.53	3.59	4.25	4.44	4.42	4.5	6.56	4.78
RUBCN	4.44	4.11	4.17	4.25	4.58	4.5	4.84	4.92
GPRC5A	6.73	6.67	6.24	6.5	5.31	4.5	4.87	5.2
MMP17	3.56	3.32	3.27	3.09	4.03	4.51	2.76	4.21
OXNAD1	3.9	3.13	3.53	3.9	3.89	4.51	5.28	4.22
EID2B	4.09	3.55	3.7	4	4.87	4.51	4.93	4.23
TMEM255B	4.48	4.31	4.71	4	4.43	4.51	3.76	4.36
ASB7	4.27	4.4	5.12	4.54	5.05	4.51	5.54	5.31
ZYG11B	3.59	2.94	4.08	4.68	4.11	4.51	6.53	5.69
LTN1	3.9	3.71	4.06	4.54	3.92	4.51	7.27	6.85
VCX3A	3.27	2.26	4.33	4.96	3.84	4.52	2.73	1.92
C4A	5.29	5.51	4.31	4.57	3.84	4.52	4.21	1.99
TRMT44	4.15	3.92	4.63	4.51	4.41	4.52	4.18	4.22
CCDC136	3.68	3.84	3.97	4.42	3.93	4.52	5.44	4.87
LURAP1L	3.24	3.3	3.98	5.12	4.33	4.52	5.44	5.33
FEM1B	4.54	4.12	5.02	5.73	4.64	4.52	6.17	5.67
ALS2	4.57	4.37	4.59	5.58	4.69	4.52	5.58	5.78
SLF2	4.69	3.14	4.42	4.92	4.24	4.52	7.45	5.89
GEM	3.87	4.43	3.42	4.76	4.14	4.53	5.71	4.41
C9orf91	4.04	4.45	4.25	4.41	4.79	4.53	4.82	4.71
CRTC3	4.3	5.03	4.9	4.77	4.79	4.53	4.73	4.92
ETV6	5.05	4.91	5.43	5.87	4.81	4.53	5.86	5.06
CCDC77	3.74	3.48	4.55	4.57	4.53	4.53	6.22	5.26
CENPQ	4.45	4.36	4.34	4.5	4.22	4.53	6.25	5.46
VWA8	4.08	3.83	4.46	5.17	4.89	4.53	6.89	6.38
ELK4	3.23	3.53	3.98	5.48	4.53	4.53	7.73	6.76
LOC102724770	4.28	5.35	4.77	5.47	3.95	4.54	4.93	2.79
INPP4A	4.3	4.71	4.48	4.64	4.34	4.54	5.37	5.34
GPATCH2L	3.97	3	4.14	4.86	4.07	4.54	6.36	6.36
ATR	3.58	3.08	4.32	5.45	5.66	4.54	8.78	6.73
DPYSL4	4.93	4.23	4.49	3.94	4.94	4.55	4.08	4.47

KIAA0922	3.91	3.94	4.02	4.2	4.2	4.55	5.06	4.68
AGAP6	3.39	3.45	4.02	3.63	3.19	4.55	5.9	4.74
MREG	4.02	4.17	3.38	4.56	4.68	4.55	4.19	5.01
DNASE1	4.86	4.03	5.33	5.86	4.6	4.55	5.9	5.13
MAP2	3.41	3.24	4.26	5.47	4.31	4.55	6.76	5.94
ZDHHC17	4.23	3.59	4.32	5.5	4.39	4.55	8.14	6.61
COG6	4.63	3.77	4.84	6.01	5.06	4.55	8.26	6.92
CCNT2	4.17	4.11	4.66	5.73	4.78	4.55	8.36	7
RARRES2	2.85	3.33	2.04	2.51	4.84	4.56	2.75	3.52
CABLES1	3.67	3.76	3.65	3.85	4.86	4.56	3.84	4.21
C16orf54	5.88	6.59	5.81	5.14	4.33	4.56	3.96	4.78
TRMT10B	2.82	2.76	4.3	4.34	3.83	4.56	4.87	5.07
NCK1	4.25	4.51	4.96	5.36	4.55	4.56	6.58	5.53
FBXL4	4.55	4.49	4.4	5.43	5.71	4.56	6.67	6.03
TRIM52	4.7	4.07	4.5	6.6	4.72	4.56	9.1	6.52
PIKFYVE	3.92	3.57	4.41	5.48	5.01	4.56	8.24	6.59
TMEM116	4.65	3.46	3.79	4.24	4.08	4.57	4.89	3.9
MTRNR2L3	3.91	4.57	4.09	4.34	3.88	4.57	4.65	4.09
PPP1R3D	4.08	4.46	4.78	4.1	4.59	4.57	4.29	4.31
SYNE3	4.3	4.2	4.24	4.1	4.56	4.57	4.16	4.54
NRP2	4.03	3.99	3.9	4.34	4.76	4.57	5.14	5.02
POC1B	3.22	3.51	3.6	3.7	4.29	4.57	6.07	5.21
PWWP2A	4.21	4.42	4.75	5.31	4.5	4.57	6.11	5.54
N6AMT1	4.01	3.4	4.37	4.59	6.45	4.57	5.99	5.96
MPP5	4.21	3.46	4.4	5	5.19	4.57	6.47	6.5
BAG4	3.99	3.44	4.76	5.72	4.7	4.57	6.34	6.52
FAM13B	4.2	3.77	4.64	5.74	4.72	4.57	8.43	7
PIK3CA	4.54	4.51	4.64	6.18	5.07	4.57	8.49	7.45
FKBP11	2.63	2.58	2.89	4.15	3.42	4.58	5.24	3.55
ARHGAP4	4.73	4.32	4.58	4.27	3.97	4.58	3.49	3.83
FANCE	4.62	5.54	4.22	4.69	4.88	4.58	4.59	4.54
C3orf58	5.1	4.83	5.55	6.37	4.49	4.58	5.89	4.87
PARP6	5.97	5.85	6.07	7.49	5.85	4.58	7.44	6.23
ZNF669	4.92	4.53	5.16	5.24	5.7	4.58	6.64	6.5
ASB3	5.13	4.92	5.28	6.1	5.4	4.58	4.18	7.25
TSPAN10	5.48	5.63	5.22	4.76	4.43	4.59	2.98	4.02
NRF1	5.19	5.49	5.1	4.74	4.64	4.59	5.11	4.26
SMIM10L1	3.83	3.66	4.59	4.49	3.98	4.59	4.73	4.27
ZFYVE9	4.48	4.55	5.01	4.62	4.36	4.59	5.52	5.11
CLMN	4.64	4.87	4.54	4.81	4.73	4.6	4.62	4.42
LHFPL2	3.97	3.52	4.06	4.44	4.36	4.6	4.59	4.84
XPA	3.54	3.8	4.02	5.16	4.47	4.6	6.69	5.19
ADAM23	4.09	4.22	4.44	5.06	4.63	4.6	5.19	5.39
METTL15	4.03	3.57	4.21	5.03	3.71	4.6	7.09	5.61
ZNF189	4.87	4.64	5.44	7.02	4.59	4.6	8.25	7.08
EFNB2	5.02	4.54	4.9	4.93	4.48	4.61	4.64	4.41
LRIG2	3.81	3.83	4.39	5	4.11	4.61	5.77	5.37
NBPF14	4.95	4.7	4.69	5.28	4.81	4.61	6	5.72
ZMYM1	3.96	4.12	4.57	5.55	5.27	4.61	8.77	7
TROVE2	4.87	4.49	4.85	5.78	4.71	4.61	8.79	7.06
ATP11B	4.15	3.78	4.64	5.5	4.63	4.61	8.03	7.23
DOK4	5.24	5.74	5.18	4.9	4.34	4.62	4.5	4.36
NAF1	5.49	4.77	5.4	5.74	5.42	4.62	5.48	5.41
TMOD3	4.11	3.91	5.25	5.75	4.58	4.62	6.53	6.29
MIOS	4.98	5.15	5.85	5.49	5.32	4.62	6.93	6.54
TMEM88B	4.54	3.8	3.91	3.67	3.76	4.63	2.76	3.33
ZNF784	4.83	4.69	5.11	3.97	4.32	4.63	3.5	3.54
GMEB1	3.75	4.22	4.46	4.44	4.63	4.63	4.23	4.02
WBP4	3.84	4.42	4.55	5.75	3.88	4.63	7.46	6.15
TTF1	4.53	3.74	4.29	5.17	5.12	4.63	5.98	6.35
FOXO6	6.51	7.01	5.9	6.01	4.23	4.64	3.03	3.6
GATA6	5.67	4.51	5.13	4.67	4.83	4.64	3.46	3.94
PLEKHN1	4.71	4.4	4.64	5.3	4.53	4.64	3.96	4.09
PRRG2	4.42	4.99	3.68	3.4	4.25	4.64	3.77	4.12
PLCXD1	4.92	4.78	4.65	3.91	4.47	4.64	3.98	4.68
BTN2A2	4.25	4.31	4.12	4.52	4.53	4.64	4.76	4.71
SGMS1	3.99	3.68	4.39	4.77	4.99	4.64	5.44	5.06
AP5B1	4.49	4.41	4.58	4.12	4.16	4.65	3.91	4.39
BUD13	3.99	5.02	4.43	5.01	4.09	4.65	4.64	4.49
LDLRAD3	4.38	4.56	4.27	4.33	5.51	4.65	5.86	5.14
CCDC113	4.12	4.29	5.09	5.47	5.06	4.65	4.9	5.34
XRCC2	3.59	3.41	4.21	5.5	4.6	4.65	7.76	5.56
CNTLN	4.09	3.79	4.31	5.08	4.99	4.65	7.85	6.29
SHISA9	4.02	3.27	4.52	5.77	4.01	4.65	8.31	7.08
SLC45A1	6.31	6.17	6.26	5.53	4.36	4.66	3.84	4.57
TTLL5	4.89	4.64	5.01	5.06	4.57	4.66	5.06	4.94
BTBD7	3.68	3.13	4.55	4.61	3.95	4.66	4.82	5.05
HAS2	5.77	4.95	5.56	6.75	4.49	4.66	6.19	5.31
C20orf194	4.59	4.87	4.94	5.63	5.37	4.66	5.97	5.45
TMEM161B	4.87	4.66	5.37	5.55	5.28	4.66	10.17	8.64
NTF4	4.42	4.42	3.64	4.34	3.7	4.67	4.15	3.85
CLTCL1	4.46	4.62	4.26	4.05	4.98	4.67	5.38	5.19
DLAT	4.04	4.44	4.92	5.03	5.52	4.67	6.62	5.7
MTERF1	3.99	4.6	4.92	5.26	3.99	4.67	7.76	5.75
MCEE	4.58	4.51	4.6	5.69	5.72	4.67	6.7	5.91

DCUN1D1	4.08	4.61	4.48	5.17	5.1	4.67	8.14	6.55
USP8	4.48	4.49	5.56	7.24	5.63	4.67	8.71	7.97
TBL1X	4.78	4.95	4.84	5.3	4.47	4.68	4.62	4.52
CCDC174	5.09	4.87	5.08	4.41	4.56	4.68	5.84	4.98
SLC35A5	4.62	4.74	5.14	5.86	5.18	4.68	7.25	5.39
FAM98B	4.35	3.84	4.48	4.91	5.24	4.68	7.48	5.95
SMIM3	6.33	5.94	5.75	6.16	4.22	4.69	4.84	4.31
ALKBH1	4.43	4.57	3.98	3.92	4.47	4.69	4.2	4.52
KIAA0355	4.41	4.28	4.45	5.12	5	4.69	4.85	4.89
PDSS1	3.55	3.41	3.57	4.8	4.75	4.69	5.48	5.09
NAPG	3.32	3.79	4.18	4.63	4.19	4.69	6.71	5.95
DBP	3.89	5.16	3.86	3.54	4.15	4.7	3.24	3.84
KHDC1	5.14	5.17	3.79	3.79	3.89	4.7	3.89	4.56
ZNF398	5.5	5.45	5.58	5.32	4.66	4.7	4.82	4.68
PXK	4.45	4.43	4.46	5.03	4.16	4.7	4.5	4.83
ARNTL	4.81	4.11	4.4	5.15	4.8	4.7	5.63	4.84
FMO3	4.75	4.86	5.79	5.37	5.39	4.7	6.13	5.42
FAM175B	4.29	3.59	4.78	5.66	4.69	4.7	7.22	6.2
NAA25	3.78	3.31	4.85	5.2	4.58	4.7	7.84	6.86
SNAI3	3.78	4.51	3.7	3.52	3.68	4.71	3.18	3.89
POC5	4.32	3.56	4.19	5.41	4.71	4.71	7	5.31
RASSF8	5.21	4.94	5.28	7.08	4.81	4.71	7.97	6.62
SPDL1	4.53	3.4	4.68	5.04	4.46	4.71	8.57	7.47
ZNF710	5.18	5.52	4.77	5.23	5.24	4.72	4.55	4.56
LOC107986794	2.62	3.42	3.17	3.65	3.76	4.72	5.27	4.83
KCNK2	4.11	3.55	4.29	4.67	4.53	4.72	5.15	5.07
LMO4	5.2	5.23	5.08	5.27	4.65	4.72	4.86	5.25
THSD4	4.88	4.92	4.94	5.17	4.85	4.72	5.23	5.25
ZNF766	3.9	3.8	4.71	5.11	4.35	4.72	6.21	5.94
RABIF	4.51	5.32	5.08	5.5	4.7	4.73	5	4.73
UBE4A	3.79	3.53	3.67	4.62	4.35	4.73	6.27	5.08
VANGL2	4.45	5.35	5.11	4.96	4.87	4.73	4.2	5.11
FABP5	4.04	4.58	4.98	5.44	4.45	4.73	5.91	5.94
LGALS8	5.31	4.28	6.07	6.21	5.19	4.73	7.35	6.4
LOC101926982	3.37	3.81	3.33	4.86	2.67	4.74	5.95	4.23
CLASP2	4.45	3.83	4.93	4.63	5.14	4.74	6.16	5.43
ATG14	4.74	4.35	4.83	5.11	5.11	4.74	7.09	5.72
AKR1B15	4.37	4.41	5.18	5.37	4.31	4.74	4.85	5.99
L3HYPDH	5.45	4.88	4.53	4.78	5.26	4.74	6.57	6.05
DSC2	5.04	5.03	5.95	6.13	4.72	4.74	6.8	6.53
GPR3	4.41	4.26	4.78	4.72	4.93	4.75	4.33	4.57
HIVEP3	4.21	4.14	4.1	4.53	4.48	4.75	4.38	4.92
SOX11	4.47	4.52	4.89	5.38	5	4.75	4.89	5
TBC1D4	4.49	3.79	5.03	5.53	4.62	4.75	6.02	5.7
TMEM62	5.35	5.45	4.97	5.27	5.71	4.75	6.67	5.76
SAR1B	3.93	3.53	4.52	5.49	4.84	4.75	6.79	6.43
HIPK3	3.85	4.16	4.82	5.64	4.86	4.75	6.96	6.51
ICE2	4.36	3.82	5.21	5.33	4.78	4.75	8.87	6.83
KRAS	3.98	4.16	4.93	5.91	5.38	4.75	8.66	7.24
SMCHD1	4.01	3.39	5.17	6.12	4.51	4.75	9.14	7.57
ARMC9	5.24	5.45	5.62	5.76	4.75	4.76	4.77	4.79
THNSL1	4.02	3.11	4.35	4.73	4.71	4.76	6.76	5.49
EAF1	4.03	3.71	4.19	4.29	5.15	4.76	5.54	5.7
HOMEZ	5.88	6.41	5.58	5.6	5.54	4.76	4.68	5.73
CHM	3.99	3.91	4.68	5.39	4.49	4.76	7.82	6.97
EPHB2	4.49	4.67	4.42	3.92	4.52	4.77	3.95	4.53
RREB1	4.92	5.11	4.77	4.83	4.01	4.77	4.59	4.59
STEAP1	3.83	3.87	4.02	4.41	5.1	4.77	5.77	4.75
PALM	8.84	10.12	8.27	7.27	4.69	4.77	3.34	5.14
WDR44	4.23	3.97	4.12	4.91	4.6	4.77	6.18	5.92
SLC7A11	3.34	3.27	4.18	5.36	4.25	4.77	9.78	7.03
WDFY2	4.55	4.6	4.45	5.71	4.72	4.78	4.98	4.88
SYNJ2	4.45	4.77	5.49	5.3	5.09	4.78	5.38	5.29
ANKRD6	4.12	4	4.89	5.1	4.51	4.78	6.89	6.17
LOC107986877	4.99	5	4.99	6.43	5.63	4.79	4.24	4.43
OPN3	4.67	4.7	4.96	5.37	5.2	4.79	5.57	5.2
CLCA2	3.7	2.91	4.22	4.17	4.64	4.79	6.42	5.35
STAM	4.22	4.01	4.86	5.58	5	4.79	6.42	5.67
POGLUT1	4.47	4.11	4.69	5.15	5.3	4.79	7.6	5.97
PAN3	4.06	3.53	4.1	5.66	4.29	4.79	6.06	6.26
SPDYE2	3.11	4.22	3.98	4.23	3.86	4.79	4.73	6.84
ZNHIT6	5.08	4.27	4.84	5.71	5.53	4.79	8.21	7.17
LRRC8E	4.38	4.13	4.45	4.25	4.38	4.8	3.31	4.06
QRSL1	3.54	4	4.12	4.28	4.63	4.8	5.16	4.88
NR1D2	2.68	2.65	3.36	4.19	4.49	4.8	7.26	6.5
SHOC2	4.38	4.34	5.01	6.07	4.87	4.8	8.41	7.51
ARHGAP39	4.76	4.75	4.26	4.5	4.86	4.81	4.04	4.22
UBE2D4	5.05	4.86	4.87	4.87	5.25	4.81	5.06	4.71
BRAF	4.29	4.64	4.88	5.93	5.01	4.81	6.11	5.97
CEACAM1	5.34	5.48	6.35	5.82	6	4.81	4.53	6.12
PCGF6	4.26	4.36	5.09	5.27	6.25	4.81	6.62	6.16
NHLRC3	4.34	4.34	5.19	6.82	4.56	4.81	7.19	6.47
SEMA3D	5.12	4.54	5.29	7.13	4.45	4.81	8.73	7.03
FAM177A1	4.18	4.36	4.47	4.96	4.92	4.82	5.71	5.42

HIST2H2AC	3.59	3.91	4.66	3.91	2.94	4.83	2.6	3.64
IL27RA	6.22	6.27	5.64	4.96	4.66	4.83	3.86	3.95
ZBED3	4.63	4.88	4.23	4.39	4.75	4.83	4.2	4.01
SPDYE3	3.83	3.87	4.04	4.71	4.52	4.83	4.93	4.43
ZNF85	4.71	5.18	4.93	5.72	5.74	4.83	6.58	5.19
GLB1L	5.61	6.01	6.1	5.92	5.57	4.83	6.2	5.38
EIF2AK3	4.39	4.19	5.09	5.63	5.34	4.83	7.03	5.6
CYLD	4.02	3.74	4.6	5.27	5.06	4.83	5.88	5.91
ENTPD5	5.05	4.62	4.72	6.41	5.24	4.83	7.2	6.63
TCAIM	4.97	4.25	5.33	6.3	6.47	4.83	7.6	6.8
NBPF8	3.77	3.4	5.51	6.79	5.07	4.83	7.77	6.82
REV3L	4.01	4.11	5.31	6.56	4.58	4.83	9.05	7.81
CENPO	4.83	4.54	4.86	4.84	5.81	4.84	5.63	4.86
PHF10	5.65	5.34	5.64	6.08	5.31	4.84	6.37	5.31
TRIM45	4.33	4.24	4.21	5.32	4.51	4.84	4.94	5.61
EPG5	4.12	4.04	4.65	5.18	4.71	4.84	6.1	5.66
CHD9	4.26	3.91	5.15	6.53	4.63	4.84	9.93	8.4
DQX1	3.59	3.87	4.17	3.81	4.89	4.85	4.83	5.94
ZNF254	5.4	4.48	5.74	5.74	4.87	4.85	7.29	6.57
DIS3	4.3	3.96	5.03	5.44	4.63	4.85	7.76	6.62
BOD1L1	3.75	3.92	4.53	6.11	4.27	4.85	8.15	7.57
STIL	5.09	3.75	5.46	6.2	4.97	4.86	7.11	5.85
C2orf49	4.34	4.49	4.94	5.23	4.01	4.86	6.08	6.04
ZNF174	4.76	5.15	4.72	4.97	4.54	4.87	4.47	4.43
RPL22L1	3.93	4.33	3.62	5.14	4.71	4.87	6.27	5.34
CEP120	4.55	4.02	5.18	6.07	4.6	4.87	8.27	6.69
KCTD7	4.46	4.7	4.7	5.03	4.94	4.88	5.47	5
VPS53	5.54	5.68	4.54	5.44	4.92	4.88	5.65	5.65
ULBP3	4.85	4.81	4.98	5.4	4.57	4.88	5.18	5.81
SH3D19	4.68	4.08	4.86	5.26	5.02	4.88	6.59	5.94
TBCCD1	5.46	4.84	4.94	5.49	4.66	4.88	5.88	6.35
SPINK5	3.79	3.67	4.19	5.82	4.37	4.88	7.78	7.43
UPF2	4.23	3.96	5.23	6.42	5.69	4.88	9.08	7.66
MAP7D3	4.15	3.24	5.03	5.3	4.9	4.89	6.59	6.03
GPX8	4.79	5.12	6.26	6.62	5.05	4.89	7.5	6.6
ARRDC4	4.42	4.29	5.02	4.93	5.23	4.89	6.86	6.82
KMT2C	4.66	4.31	5.08	6.59	5.12	4.89	7.44	7.18
MAP4K3	5.11	4.37	4.95	6.42	5.43	4.89	8.98	7.19
RNPC3	4.44	4.19	4.66	5.02	6.22	4.89	9.17	8.53
RAD51	5.75	4.88	5.65	5.17	5.9	4.9	4.95	4.44
ZCCHC24	6.01	6.12	5.72	5.89	4.71	4.9	4.31	4.78
NMI	4.56	5.31	5.57	6.84	4.54	4.9	5.92	4.96
SRGAP3	4.08	4.87	4.13	5.08	4.59	4.9	5.11	5.1
NUDT7	5.02	4.48	5.41	5.2	5.1	4.9	6.4	5.89
ERI1	4.42	4.59	5.79	4.94	5.16	4.9	6.91	6.21
RGS20	5.15	5.5	6.13	4.47	5.7	4.9	6.02	6.22
CHORDC1	5.76	5.84	5.78	6.43	5.33	4.9	8.93	7.98
FZD8	6.27	6.17	5.48	6.01	4.36	4.91	4.19	4.31
LHX6	5.56	4.9	4.9	4.27	4.39	4.91	3.77	4.36
TBC1D22B	4.31	4.5	4.99	4.54	4.45	4.91	4.61	4.56
ANKMY1	4.2	4	3.94	4.27	5.03	4.91	5.11	4.68
CREM	4.8	4.93	4.35	4.12	5.95	4.91	6.73	4.74
CNNM2	5.15	4.75	4.33	4.41	5.28	4.91	4.75	4.85
RNGTT	4.31	4.06	5.06	4.85	4.87	4.91	6.48	5.63
ZCCHC4	3.74	4	4.7	4.53	5.06	4.91	6.5	5.73
CCDC117	4.8	4.5	4.98	4.99	6.13	4.91	6.72	6.29
SNAPC1	5.17	5.25	5.82	6.07	5.76	4.91	7.28	6.62
SNX24	4.57	4.23	4.61	5.49	5.45	4.91	5.78	6.93
MDP1	7.07	7.66	7.18	7.16	6.42	4.92	5.09	4.03
SLC16A2	3.66	4.15	3.66	3.74	4.99	4.92	4.43	4.96
VPS36	4.52	4.18	4.88	6	5.28	4.92	7.69	6.49
CEP89	6.49	5.67	6.35	6.21	6.06	4.92	6.75	6.54
AP5M1	4.52	4.05	4.59	5.92	5.18	4.92	8.59	7.19
CYP2R1	4.8	4.78	5.51	5.62	5.17	4.93	7.68	5.92
SPAG16	4.67	4.84	4.83	5.74	4.26	4.93	7.2	6.08
KLHL20	5.22	4.2	4.83	6.45	5.46	4.93	7.84	6.34
NBPF26	4.16	3.45	4.12	5.33	5.83	4.93	7.09	6.61
PDS5B	4.51	3.86	5.35	5.83	5.07	4.93	7.9	6.61
ACAP2	3.84	4.14	5.14	5.57	4.69	4.93	7.98	6.88
DNMT3A	4.54	5.35	4.88	5.02	4.54	4.94	4.59	4.76
ME1	4	3.55	4.01	4.7	5.06	4.94	6.27	5.63
LYPD6	5.17	4.47	5.42	5.36	5.35	4.94	6.78	6.03
SDHAF3	4.64	5.94	5.45	5.11	5.8	4.94	7.55	6.09
GLCE	4.46	4.11	5.21	5.6	5.77	4.94	6.68	6.23
TMEM184C	4.26	4.59	4.99	5.6	4.93	4.94	7.16	6.71
SCGB1A1	2.08	3.24	1.74	2.37	4.71	4.95	3.15	4.01
PNMA5	6.77	7.31	7.14	6.77	5.17	4.95	4.59	4.31
METTL16	4.61	4.74	5.16	4.88	4.76	4.95	4.88	5.15
HECTD4	4.87	4.4	4.67	5.16	4.64	4.95	5.52	5.26
SLC18B1	4.54	3.61	4.64	4.66	4.8	4.95	6.05	5.71
BRWD3	4.53	4.22	4.52	5.93	4.69	4.95	8.4	7.14
THAP5	3.87	4.53	4.7	5.79	4.49	4.95	10.12	7.98
ZFC3H1	4.16	3.95	4.76	6.24	4.81	4.95	9.07	8
KAZALD1	5.9	5.51	5.24	4.7	4.63	4.96	4.28	3.98
DERL2	3.91	4.34	4.36	4.72	5.03	4.96	5.05	4.58

GPR176	3.72	3.86	4.26	3.88	4.69	4.96	4.09	4.75
PPP1R13B	5.3	5.46	5.59	4.64	4.9	4.96	4.79	5.27
SLC1A1	5.65	4.96	6.17	6.55	4.33	4.96	5.94	5.53
DYRK1A	5.52	4.77	5.76	6.31	5.27	4.96	6.52	5.95
FARP2	5.71	5.66	5.47	5.34	5.98	4.96	5.94	5.96
TGFBR1	3.06	3.26	3.81	4.57	5.03	4.96	8.14	6.23
SMARCAD1	4.12	3.52	4.46	5.41	4.97	4.96	8.41	7.68
SOS1	4.34	4.07	5.51	6.5	5.6	4.96	8.97	8.16
SWSAP1	5	4.23	4.49	4.23	5.5	4.97	4.34	4.34
GATA2	4.79	4.96	4.52	4.78	4.49	4.97	3.77	4.61
EMP2	4.44	4.51	4.7	5.02	4.77	4.97	4.86	4.84
HEMK1	4.4	4.7	4.3	4.91	5.04	4.97	5.57	5.23
TMEM234	5.76	4.69	4.88	4.41	6.44	4.97	4.32	5.3
SKA1	4.22	3.75	4.38	5.58	5.06	4.97	6.06	5.33
GNPDA2	3.93	4.08	4.05	5.27	4.43	4.97	7.04	5.8
PACRGL	5.64	4.69	4.96	6.03	6.38	4.97	6.96	6.05
LACTB	3.42	4.85	4.12	4.84	4.89	4.97	5.87	6.34
ELP4	4.3	4.09	4.33	4.08	4.72	4.98	5.77	4.7
NUDT9	4.03	4.71	4.71	5.19	4.99	4.98	6.55	4.7
DENND3	3.56	3.54	3.92	4.11	5.26	4.98	5.13	4.8
CA9	9.05	9.94	8.96	7.55	4.78	4.98	5.57	5.01
CDH11	4.38	4.58	4.81	5.25	4.77	4.98	6.04	5.49
PPTC7	4.29	3.75	4.71	4.62	4.56	4.98	5.77	5.71
C17orf75	5.63	5.02	5.67	6.39	6.07	4.98	6.45	6.44
MAML2	4.54	4.44	5.43	6.71	4.89	4.98	7.58	6.65
PLCB1	4.31	4.31	5.32	5.57	4.98	4.98	8.1	6.95
PIGK	4.27	4.65	4.43	5.13	5.42	4.98	8	7.01
IFT80	3.89	4.15	4.39	5.96	5.21	4.98	9.49	7.59
ACOT11	5.97	5.74	5.98	4.97	4.11	4.99	4.62	4.69
PCDHGB5	5.31	6	5.74	5.64	4.22	4.99	4.38	4.95
MAP1LC3B2	3.9	7.73	5.23	3.76	5.08	4.99	5.81	6.45
CTAGE5	4.35	4.01	4.32	5.13	4.86	4.99	7.46	6.77
C9orf85	3.66	3.41	4.27	4.96	4.76	4.99	7.91	7.23
PPM1J	6.99	6.89	5.8	5.13	4.85	5	4.69	4.6
TRMT12	4.12	4.3	4.25	4.3	4.23	5	4.67	5
EFNA3	7.16	7.28	6.62	6.28	5.55	5	4.41	5.24
E2F5	4.8	4.72	4.84	5.15	4.77	5	5.63	5.9
BTN3A1	4.88	5.39	5.65	6.18	5.61	5	6.35	6.24
FASTKD1	3.49	3.85	4.18	4.84	4.68	5	8.31	6.4
DPH3P1	4.81	5.18	5.19	6.19	4.83	5	5.54	6.48
DZIP3	4.3	3.84	4.85	5.56	4.99	5	8.85	7.55
ZNF764	5.07	5.59	4.56	4.7	4.92	5.01	4.31	4.98
STK36	4.02	4.23	4.46	4.7	4.59	5.01	5.37	5.5
NPIPA7	5.14	4.67	5.66	6.4	5.98	5.01	7.5	5.63
MED21	4.61	4.69	4.53	5.09	4.8	5.01	7.44	6.4
TEFM	4.48	5.15	4.96	4.39	4.86	5.01	6.55	6.43
DENND4C	4.4	4.19	5.98	6.61	5.06	5.01	8.82	6.86
GJB5	3.98	5.32	3.92	3.89	3.69	5.02	3.15	3.51
FARP1	5.37	5.29	5.41	5.06	5.41	5.02	4.54	4.63
ARSB	4.58	4.78	4.77	5.4	4.98	5.02	5.17	4.99
NUP35	3.77	3.62	4.68	5.16	4.63	5.02	6.03	5.09
CXorf57	4.67	4.6	4.94	5.95	4.51	5.02	7.56	6.32
DNAJC3	5.25	4.32	6.14	6.89	5.8	5.02	8.3	7.32
REEP3	4.24	4.18	5.39	6.79	5.04	5.02	9.26	8.6
ADCK1	3.67	3.77	3.82	3.77	3.75	5.03	4.03	3.73
ADGRB2	6.09	5.92	5.59	5.84	4.47	5.03	3.85	4.42
FAM86KP	5.83	2.81	4.57	4.19	3.84	5.03	5.16	4.44
KBTBD11	4.24	3.25	4.06	3.81	4.72	5.03	5.13	4.59
SMYD4	4.7	4.71	5.02	5.62	4.67	5.03	4.92	5
DSE	3.3	3.4	4	4.41	5.08	5.03	6.66	5.79
ATG16L2	3.76	3.4	3.7	3.66	5.5	5.03	6.81	5.91
RNF217	5.5	4	5.36	6.49	4.61	5.03	6.66	6.18
FBXO5	5.09	4.52	4.79	5.42	5.47	5.03	7.11	6.35
PCNX4	5.21	4.58	5.6	6.54	5.37	5.03	8.63	6.93
IMMP1L	6.1	4.82	4.54	5.12	6.56	5.03	6.37	7.41
NAPRT	3.37	3.57	3.05	3.21	3.77	5.04	5.48	4.69
ZNF576	4.73	4.78	4.9	4.45	5.35	5.04	4.03	4.95
UBE3D	4.43	5.19	4.72	5.65	5.58	5.04	6.26	5.03
PCDHGA4	5.01	4.66	5.13	4.86	4.4	5.04	4.88	5.41
RWDD4	4.61	4.43	5	5.15	4.6	5.04	7.05	5.66
POLR2M	4.37	4.49	4.5	4.78	5.03	5.04	6.66	5.7
ERMARD	4.86	3.79	4.54	5.52	5.67	5.04	6.51	5.99
PAPOLG	4.44	4.3	5.16	5.61	5.49	5.04	7.61	6.39
MBIP	4.85	4.62	5.07	5.48	5.09	5.04	7.09	6.91
GEMIN8	5.8	5.79	5.93	5.92	6.64	5.04	6.7	7.08
SUSD2	3.95	3.6	4.24	3.29	5.24	5.05	4.15	4.58
RAB28	5.68	5.99	5.25	5.82	5.36	5.05	6.44	6.25
CKAP2L	3.7	3.39	4.22	5.13	4.86	5.05	9.21	6.95
CCDC91	4.52	4.48	4.87	5.34	5.41	5.05	8.38	7.66
SMAD6	5.37	5.45	5.65	5.25	4.66	5.06	4.47	4.1
SHROOM1	5.15	5.09	4.89	4.45	5.01	5.06	3.91	4.7
DEF6	5.96	5.79	6.57	5.21	4.99	5.06	4.03	4.76
PCNT	4.45	4.18	4.97	5.03	5.14	5.06	6.1	5.72
THUMPD2	3.42	3.45	4.19	4.9	5.57	5.06	7.15	5.85

PNPLA8	4.6	5.12	4.82	5.51	5.46	5.06	8.65	7.14
TLK1	4.38	4.24	5.21	6.67	5.34	5.06	8	7.19
ZNF644	6.26	6.06	6	6.65	5.45	5.06	9.61	7.37
ARL14EP	6.05	5.8	5.75	6.76	5.56	5.06	9.26	7.81
NIPBL	4.89	4.41	5.65	6.99	4.8	5.06	9.77	8.27
MPHOSPH9	4.48	4.06	4.98	6.01	4.65	5.06	9.16	8.3
BMI1	5.55	5.86	6.18	8.04	7.21	5.06	7.75	9.5
SAA1	4.29	3.96	5.56	2.89	7.4	5.07	6.45	4.56
EEF1AKMT1	4.62	4.01	4.4	5.32	4.36	5.07	5.93	4.86
PIAS2	4.82	4.51	5.29	5.61	5.02	5.07	6.72	5.98
RNF138	5.45	5.41	5.45	5.67	5.39	5.07	6.53	6.2
MLLT10	5.18	4.32	5.3	5.7	5.7	5.07	7.75	7.62
TTLL4	4.87	5.26	5.12	5.16	4.86	5.08	5.23	5.55
RNF111	5.65	5.03	5.49	5.95	5.26	5.08	6.39	5.65
SCFD2	5.86	6.78	6.34	5.81	7.37	5.08	6	5.84
NT5DC3	4.39	4.05	4.36	4.86	5.27	5.08	6.62	6.25
RALGPS2	4.41	4.69	4.82	5.68	5.25	5.08	7.25	7.05
NFKB1	5.11	4.72	5.22	5.37	5.28	5.1	5.65	4.89
BRAP	5.01	4.73	5.07	5.3	5.22	5.1	6.23	5.72
PTPRG	4.88	4.48	5.16	6.07	4.56	5.1	6.23	6.12
GSKIP	4.46	4.29	4.89	5.36	5.88	5.1	7.79	6.61
STRN3	5.22	4.38	5.78	6.74	5.53	5.1	8.84	7.38
APOOL	4.17	3.99	3.77	4.43	4.09	5.11	6.15	5.8
OSGEPL1	4.04	4.07	3.99	4.84	5.21	5.11	6.54	5.89
MMD	5.28	5.44	5.87	6.64	5.15	5.11	6.68	6.35
EGR1	8.98	8.27	8.37	8.61	5.4	5.12	5.86	5.15
RBMXL1	4.07	3.95	4.63	5.29	4.71	5.12	6.73	5.48
BCL2L11	5.61	5.3	6.24	5.81	5.54	5.12	5.36	5.71
DCK	4.5	4.58	4.75	5.5	5.68	5.12	7.26	5.72
PARM1	6.14	5.86	5.68	6.92	4.6	5.12	5.7	5.79
MSL2	6.11	5.93	6.48	6.45	6.38	5.12	7.12	6.32
SGPP1	5.17	4.92	5.74	5.99	5.36	5.12	6.32	6.47
ZBTB33	4.5	3.87	5.47	6.14	5.5	5.12	8.56	7.24
TRIB1	4.84	5.58	5.37	5.44	5.05	5.13	5.76	5.01
CTPS2	5.14	4.59	4.78	5.54	5.11	5.13	5.49	5.08
TTC23	5.14	4.85	4.87	4.99	4.72	5.13	5.94	5.24
C19orf44	4.86	5.34	5.1	5.78	5.34	5.13	5.46	5.33
BTN3A2	4.63	4.73	4.9	5.22	5.54	5.13	5.43	5.55
SIK3	4.93	5.02	4.82	5.31	5.29	5.13	4.91	5.89
IFIT2	5.6	5.77	6.3	7.43	4.98	5.13	6.42	6.4
C1orf27	4.01	3.72	3.82	4.65	5.41	5.13	8.72	6.44
MLPH	5.23	5.09	4.99	5.19	5	5.14	4.86	4.93
THG1L	5.94	5.54	5.79	6	4.83	5.14	5.32	5.02
FAM105A	2.87	3.36	3.46	3.7	4.85	5.14	6.11	5.75
ATP11A	5.02	5.02	5.25	5.27	5.23	5.14	5.72	5.89
ANGEL2	5.46	4.88	5.33	5.73	5.49	5.14	7.81	7.64
UFSP1	4.71	5.11	5.16	4.42	4.54	5.15	2.72	3.97
TBKBP1	5.59	6.07	5.83	5.72	5.05	5.15	4.2	4.51
CPQ	5.43	6.04	5.28	5.08	5.1	5.15	5.55	4.66
MTHFS	5.01	5.6	5.6	5.21	5.15	5.15	4.86	5.01
FBXO45	4.25	3.79	4.37	4.87	4.96	5.15	6.15	5.09
EXO1	5.55	4.84	5.97	6.02	5.11	5.15	6.92	5.29
TAF1	4.94	4.95	6.26	6.44	5.34	5.15	8.14	6.88
DPYD	5.34	4.34	5.46	5.8	5.64	5.15	8.23	7.01
PCOLCE	5.8	4.68	4.8	5.57	4.45	5.16	4.76	5.1
PDE12	3.87	3.97	4.09	5.17	4.84	5.16	5.89	5.34
CPS1	5.66	6.02	5.97	6.06	4.9	5.16	6.1	6.43
RABL3	4.21	3.99	4.58	5.38	5.01	5.16	8.4	6.46
UBR3	3.89	3.97	4.19	5.72	4.97	5.16	6.81	6.6
PAPSS2	5.56	5.1	5.54	5.66	5.13	5.17	5.42	4.92
ADAM19	6.47	6.83	5.99	6.7	4.98	5.17	5.05	5.42
SIPA1L1	5.51	5.53	5.79	5.77	5.4	5.17	6.03	5.7
SH3BP5	4.74	4.91	4.87	6.14	6.36	5.17	6.45	6.1
FIGNL1	4.67	4.13	5.68	6.28	5.48	5.17	8.65	7.27
SFXN5	4.67	5.04	5.33	4.71	4.96	5.18	4.85	5.55
RNF38	4.75	4.78	5.32	6.06	5.64	5.18	7.24	6.3
TADA2B	4.57	5.1	4.81	4.76	5.35	5.19	4.73	5.02
DSCC1	4.55	4.37	5.08	5.04	5.4	5.19	7.36	5.98
DLX1	4.67	4.57	4.19	4.24	4.77	5.2	5.74	5.45
C12orf43	5.22	5.23	5.62	4.98	5.61	5.2	5.24	5.48
STX2	4.59	4.36	4.95	5.48	5.9	5.2	8.21	6.09
TTC13	5.32	4.62	5.63	5.66	5.67	5.2	8.27	6.72
NADK2	4.31	4.47	4.99	5.59	5.36	5.2	8.45	6.91
CHML	4.52	4.14	5.41	6.01	5.46	5.2	9.6	7.57
INPP1	4.92	5.02	4.75	4.36	5.97	5.21	5.3	4.79
COQ7	4.74	5.3	5.33	5.69	5.55	5.21	5.23	5.13
C3orf38	4.98	4.52	5.01	5.55	5.61	5.22	6.97	6.02
TACC1	4.65	4.37	5.2	5.74	5.28	5.22	6.89	6.42
STK3	4.97	4.66	5.08	5.87	6.01	5.22	7.89	7.05
ST20	4.76	3.1	3.98	4.6	4.12	5.23	5.35	4.5
NGEF	5.89	6.48	6.32	5.63	5.26	5.23	4.57	4.97
SNX1	5.63	5.26	5.68	6.25	5.71	5.23	6.67	5.88
FOXF2	3.84	4	4.21	4.73	4.41	5.24	3.76	3.74
TSSK6	5.27	4.63	5.48	4.28	4.16	5.24	3.78	3.84
B4GALT4	4.64	4.75	5.37	5.31	5.18	5.24	6.79	5.37
RABGAP1L	3.89	3.75	4.76	5.15	4.73	5.24	6.71	6.71

RBPJ	5.79	5.62	5.97	6.73	5.81	5.24	7.17	7.02
NEDD1	5.29	5.93	5.87	6.79	5.81	5.24	9.58	7.91
MRGPRX3	3.78	4.37	4.87	5.12	4.4	5.25	3.89	4.28
DNER	2.81	3.03	2.61	2.76	5.12	5.25	6.01	5.58
LRRC1	5.24	4.92	5.54	5.12	5.22	5.25	6.48	6.09
TRIM38	6.09	5.41	5.6	6.19	5.03	5.25	7.19	6.15
PCDHB10	7.55	6.41	7.19	8.81	6.22	5.25	6.66	6.37
ZNF71	5.25	5.82	5.67	4.8	5.3	5.27	4.15	4.87
PPM1M	5.68	5.38	4.57	5.28	5.22	5.27	5.11	4.94
HSD17B11	5.77	5.15	5.07	5.26	5.47	5.27	6.14	5.74
TEP1	4.64	4.86	4.8	5.44	5.31	5.27	5.7	5.8
RAD52	4.64	4	4.17	4.95	5.12	5.27	7.1	5.99
HOXB6	4.6	4.31	3.89	4.65	4.17	5.28	5.26	4.24
PRTFDC1	4.84	4.38	4.58	4.77	5.23	5.28	6.42	5.32
CENPA	4.17	3.31	2.97	3.42	4.69	5.28	5.27	5.44
C2orf42	6.05	5.38	5	5.85	5.2	5.28	6.14	5.82
GANC	4.39	4.24	4.6	5.64	5.06	5.28	6.21	6.16
ACADS	5.44	6.27	5.44	4.56	5.71	5.29	3.52	3.75
TSPYL2	5.5	6.1	5.57	5.93	5.13	5.29	4.96	5.62
RDH10	4.06	3.92	4.29	5	5.02	5.29	6.74	5.85
CXADR	3.89	3.57	4.69	5.37	3.64	5.29	6.35	6.18
ZNF134	5.11	4.86	6.01	5.89	5.74	5.29	6.88	6.25
PPAT	4.39	4.11	5.09	5.87	5.84	5.29	9.41	7.41
ZNF883	5.11	4.99	5.59	6.38	5.07	5.29	9.59	8.07
HIST2H2AA3	5.27	5.24	4.68	5.29	4.22	5.3	4.41	4.07
HIST2H2AA4	5.27	5.24	4.68	5.29	4.22	5.3	4.41	4.07
IL11RA	5.74	5.29	5.83	5.41	4.66	5.3	4.58	4.54
RBKS	5.82	6.25	5.94	5.16	5.92	5.3	4.83	5.51
TUBGCP3	5.56	5.19	5.34	5.82	5.45	5.3	5.59	5.58
SUDS3	5	5.39	5.35	5.64	5.46	5.3	6.44	6.43
ZC3H10	4.51	4.89	4.19	4.55	5.19	5.31	3.8	4.75
SEC22A	4.63	4.34	4.68	5	5.54	5.32	6.68	5.14
E2F6	5.2	4.64	5.44	5.62	6.17	5.32	6.36	5.35
TMEM107	5.5	4.91	5.75	5.28	5.47	5.32	5.61	5.55
SCYL2	4.97	4.44	5.45	5.98	5.58	5.32	8.92	7.02
ETAA1	5.78	5.8	6.43	6.27	5.64	5.32	8.52	7.07
ACTR6	4.2	5.05	4.99	4.93	5.56	5.32	8.33	7.11
INPP5F	4.37	3.98	5	5.21	5.19	5.32	7.51	7.15
ZNF528	4.38	4.82	5.52	6.94	4.86	5.32	8.13	7.22
FAM72B	5.17	3.73	4.48	5.53	5.53	5.32	7.39	7.25
ANKRD50	4.54	3.78	4.58	6.15	5.31	5.32	8.44	7.42
RPAP2	4.91	4.05	5.65	6.27	5.19	5.32	8.1	7.7
PTAR1	4.1	4.54	5.72	5.89	4.64	5.32	9.01	8.74
AP3S2	4.47	4.05	3.74	3.85	5.29	5.33	4.53	4.92
UBOX5	6.18	6.16	5.83	5.57	5.41	5.33	4.77	5.38
INVS	4.67	4.8	4.82	5.66	4.89	5.33	6.32	6.02
RNF214	5.06	5.03	6.28	5.36	5.43	5.33	6.59	6.06
TMEM64	4.42	4.58	5.09	5.37	5.44	5.33	6.6	6.07
PRKAB2	3.85	4.23	4.9	5.41	6.02	5.33	8.1	6.77
TAF1A	4.81	4.26	6.01	6.77	5.51	5.33	8.51	7
EHBP1	5.5	4.61	5.61	6.3	5.67	5.33	8.94	7.33
KCNS3	5.77	6.88	6.3	6.54	4.45	5.34	5.16	5.44
CXorf40A	5.71	4.87	5.24	5.72	6.06	5.34	5.67	5.45
KLHL13	4.83	4.33	5.38	5.97	5.68	5.34	6.4	5.65
MDN1	4.51	4.12	4.81	5.17	5.65	5.34	7.28	6.07
CDC14B	4.91	4.77	5.68	6.32	5.84	5.34	7.52	6.8
ZNF821	5.31	5.47	6.56	5.09	5.45	5.35	4.88	5.22
FAM49A	3.87	3.91	4.03	3.9	5.56	5.35	6.21	5.41
IFT172	5.41	5.48	5.53	5.46	5.26	5.35	5.67	5.42
TOE1	5	4.36	4.49	5.12	6.08	5.35	5.41	5.58
PLAUR	6.22	6.56	7.34	5.79	5.32	5.35	6.85	6.1
UMAD1	5.08	5.13	5.29	6.36	6.31	5.35	8.19	6.74
KLHL25	5.14	5.41	4.9	4.8	5.49	5.36	4.18	4.8
ABLIM3	5.69	6.12	5.83	5.84	4.59	5.36	4.63	4.96
OPA3	4.93	4.72	4.68	4.99	4.95	5.36	4.73	5.22
SNIP1	6.16	5.62	5.65	5.79	5.74	5.36	6.07	5.94
CHST7	4.44	4.36	3.61	3.67	4.7	5.37	4.02	3.65
MAP6D1	4.88	5.61	5.34	4.92	4.46	5.37	3.89	4.2
ZNF696	4.94	4.65	4.76	5.19	4.33	5.37	4.69	4.91
CENPK	3.75	5.09	4.29	4.76	4.5	5.37	8.03	5.2
ABHD3	5.41	5.58	5.6	5.44	4.78	5.37	7.97	5.58
C5orf30	4.66	4.36	4.79	5.68	5.2	5.37	6.65	5.69
ZADH2	5.03	4.64	5.37	5.6	5.41	5.37	6.62	6.22
PTGR2	5.78	4.89	5.97	7.31	6.01	5.37	9.55	7.43
AKAP9	4.29	4.37	5.02	5.57	4.97	5.37	9.5	8.18
SNX13	4.75	4.14	5.44	6.89	5.43	5.37	10.43	8.6
RBPMS	5.73	5	5.09	5.06	5.62	5.38	4.03	5.05
BAG2	4.6	4.61	4.82	5.19	5.01	5.38	6.42	5.91
ZNF330	4.79	5.84	5.97	6.73	6.2	5.38	6.56	6.51
REPS2	5.65	5.62	7.12	5.86	5.69	5.38	6.04	6.57
FAM8A1	4.69	4.53	4.94	5.53	4.59	5.38	6.89	6.66
C9orf40	5.06	4.67	4.96	4.9	4.77	5.39	4.94	4.6
DDI2	4.2	3.7	4.22	5.33	4.28	5.39	5.46	5.78
OMA1	4.66	5.15	5.5	5.72	6.35	5.39	7.32	5.85
HMGXB4	5.65	4.95	5.85	6.16	6.1	5.39	6.58	6.58
C6orf203	5.58	5.27	5.75	5.51	6.23	5.39	8.21	6.58

FAM84B	4.96	5.23	6.32	7.38	5.87	5.39	6.77	6.7
LOC102724985	4.98	5.79	5.34	5.58	6.97	5.39	6.58	6.93
CCSER2	3.93	4.31	4.91	6.39	6.42	5.39	7.75	8.67
FAM101A	6.61	7.68	5.93	6.23	3.51	5.4	4.29	3.9
GLTSCR1	6.22	5.73	5.66	5.39	5	5.4	4.13	4.7
CES3	6.26	5.69	5.82	6.38	5.25	5.4	6.01	5.16
LBHD1	3.63	4.35	4.61	4.59	5.83	5.4	6.51	5.2
LDHD	4.32	6.11	5.01	4.53	5.05	5.4	4.23	5.43
CLPX	4.24	3.95	4.68	5.28	5.07	5.4	6.63	6.04
PATJ	5.42	4.85	5.53	5.8	5.58	5.4	6.85	6.75
FAM204A	4.78	4.79	5.48	5.82	6.08	5.4	8.2	6.95
PBDC1	5.63	4.82	4.99	4.8	4.65	5.41	6.32	5.54
JMJD7-PLA2G4B	3.8	3.09	2.57	4.87	3.14	5.41	5.6	6.44
PAXBP1	4.47	4.32	5.12	6.41	5.09	5.41	8.03	7.16
TRMO	3.72	3.76	3.61	3.99	5.23	5.42	6.17	4.64
METTL12	4.78	5.02	5.25	5.14	5.8	5.42	5.15	5.39
KDM6B	6.51	6.54	6.86	6.69	5.15	5.42	4.82	5.78
UMPS	4.77	4.46	5.29	4.98	5.51	5.42	6.03	5.81
PTCD2	4.57	4.09	4.21	5.24	5.75	5.42	6.2	5.83
BMPR1A	5.01	4.31	5.64	5.82	5.52	5.42	7.19	6.37
GEMIN2	4.95	4.63	5.12	5.63	4.92	5.42	7.06	6.45
FAM114A2	5.4	5.64	6.29	6.61	6.8	5.42	8.5	7.21
ARFIP1	5.12	4.48	5.49	6.91	6.12	5.42	8.5	7.23
NABP1	3.46	3.72	4.57	4.78	4.79	5.42	9.59	7.24
SFR1	4.95	5.39	6.4	6.52	6.57	5.42	10.52	7.75
CHAMP1	5.35	5.2	6.11	5.73	5	5.43	5.79	5.63
PAQR3	4.61	4.12	4.62	5.28	4.98	5.43	7.38	5.66
SPA17	5.08	5.71	4.37	5.58	4.88	5.44	4.83	5.3
ZNF142	5.79	5.73	5.92	5.56	5.42	5.44	5.58	5.43
PDE8A	5.49	4.17	5.41	5.2	5.6	5.44	6.77	6.06
KIAA0232	5.09	4.26	5.5	6.04	4.98	5.44	7.29	6.2
MIS18A	4.99	5.35	6.18	5.98	6.56	5.44	6.58	6.49
PIAS1	6.03	5.62	6.37	7.27	6.19	5.44	8.06	7.4
C9orf172	5.35	5	4.95	4.67	4.48	5.45	4.43	3.95
ZNF79	4.41	3.75	4.66	5.05	5.24	5.45	5.47	5.3
TRIM16	5.41	5.34	5.46	4.41	5.1	5.45	5.8	5.43
CENPU	5.27	4.75	5.31	6.42	5.96	5.45	8.74	6.66
TRIM2	5.15	4.82	4.94	5.82	4.12	5.46	5.46	5.65
NR2C1	4.73	5.7	5.33	6.42	4.82	5.46	7.81	5.87
TTLL1	5.5	6.47	5.69	6.25	5.25	5.46	5.31	6.07
ASB8	5.78	5.81	6.33	6.8	6.66	5.46	6.87	6.28
ZBTB25	4.87	4.28	5	5.83	5.2	5.46	6.97	6.64
STK32A	4.83	4.38	4.95	6.07	5.79	5.46	7.64	7.06
MCTP1	4.38	4.49	4.98	5.66	5.58	5.46	9.11	7.72
RASA4B	6.66	6.97	6.22	7.37	5.14	5.47	4.81	5.09
RBSN	5.43	5.73	5.78	5.65	5.36	5.47	5.41	5.5
COQ10B	4.99	3.98	5.46	6.16	5.1	5.47	8.53	6.88
AQP10	5.03	4.46	5.19	4.68	4.46	5.48	3.8	4.43
CRAMP1	5.01	4.88	5.28	5.24	5.25	5.48	5.79	5.84
EPB41	4.75	5.1	4.96	5.59	5.38	5.48	6.56	5.9
FKBPL	4.95	4.59	4.19	3.54	5.26	5.49	5.06	4.67
JUN	8.18	7.49	7.21	7.22	5.79	5.49	4.83	4.73
FBXO6	6.15	6.38	7.17	6.4	5.42	5.49	4.77	4.9
GPD2	5.61	4.35	5.79	6.82	5.79	5.49	8.18	6.72
RPAP3	4.75	4.51	5.83	6.91	5.64	5.49	10.18	7.94
ACBD5	4.46	4.12	5.66	6.89	6.11	5.49	9	8.15
MTRNR2L9	6.3	3.49	4.8	3.87	5.31	5.5	2.62	3.78
HOXB4	4.67	4.84	4.46	4.82	4.66	5.5	3.41	5.36
WHRN	5.53	5.23	5.32	5.67	4.66	5.5	4.95	5.53
URB2	4.95	4.68	5.27	5.01	5.27	5.51	5.34	5.41
DNAJC15	6.12	6.15	6.5	6.33	5.47	5.51	6.57	5.41
PIK3CD	5.38	5.72	5.42	5.51	5.58	5.51	5.53	5.59
UBA5	4.72	4.42	6.03	6.16	4.86	5.51	8.24	7.16
NBPF9	3.63	4.59	5.48	5.15	5.68	5.51	8.6	7.3
DBT	4.11	3.52	4.41	6.18	5.44	5.51	9.36	7.53
ATXN7L2	4.8	4.66	4.42	4.7	6.34	5.52	5.43	5.4
LTBP1	4.81	4.88	5.3	5.4	6.03	5.52	6.34	5.97
ANAPC4	4.92	4.18	5.32	6.51	4.96	5.52	9.8	7.49
CXorf38	4.79	4.63	5.5	5.63	5.24	5.53	5.94	5.85
FAM24B	3.64	4.48	5.16	4.02	5.23	5.53	6.31	5.88
PPARA	5.23	5.41	5.71	5.63	5.71	5.53	5.62	5.98
LPIN2	5.51	5.19	5.47	5.96	5.98	5.53	6.45	6.13
GPHN	5.11	5.35	5.44	4.99	5.65	5.53	7.16	6.44
ITSN1	6.2	5.29	6.61	7.03	6.26	5.53	7.29	6.89
COG3	5.17	4.43	5.53	6.21	5.94	5.53	8.22	7.33
ETV1	6.18	5.43	6.69	6.41	6.02	5.53	8.01	7.6
THUMPD1	4.53	4.83	6.79	7.42	6.39	5.53	10.3	9.4
CCP110	5.1	4.54	6.1	7.05	5.63	5.53	12.77	9.71
TMEM53	5.01	5.65	4.88	4.51	5.19	5.54	3.99	4.84
TRAIP	6.19	6.69	5.07	4.88	6.26	5.54	5.93	5.73
ZNF786	5.83	5.56	5.54	6.19	6.2	5.54	5.75	6.11
RIPK1	6.03	5.92	6.41	5.36	5.04	5.54	5.53	6.26
SS18L1	5.2	4.42	5.08	6.35	5.26	5.54	8.83	7.53
LPP	4.27	4.03	5.64	6.69	5.32	5.54	8.55	7.92

PPIP5K2	4.56	4.64	4.8	5.92	5.47	5.54	9.63	8.14
IRS1	4.85	4.67	5.29	5.09	5.72	5.55	5.48	5.87
KLHL18	4.93	4.91	4.56	4.87	6.02	5.55	6.38	5.96
HABP4	4.51	4.9	5.02	5.09	5.57	5.55	6.17	6.21
GTF2E1	5.83	5.27	5.37	6.37	5.95	5.55	7.39	6.66
P3H2	6.1	5.52	5.54	5.87	6.24	5.55	7.37	7.01
TMTC3	4.36	4.37	5.3	5.96	4.98	5.55	9.14	7.64
EPS8	5.28	4.27	6.2	7.31	5.86	5.55	7.95	7.68
TOPORS	5.51	5.85	6.28	7.05	7.05	5.55	9.47	7.71
TADA1	6.59	5.78	6.72	6.98	5.63	5.56	7.21	6.66
ALG13	4.1	4.62	5.02	5.29	6	5.56	7.88	6.75
ASXL2	4.69	3.88	5.42	6.23	5.54	5.56	7.28	6.79
ATF2	4.66	4.88	5.8	7.42	5.36	5.56	9.64	7.76
DNAJB9	5.44	6.19	6.15	6.7	6.37	5.56	9.47	7.98
G0S2	5.26	5.06	4.45	4.7	5.3	5.57	3.9	4.99
ZNF324	5.95	5.82	5.25	5.5	6.24	5.57	5.06	5.84
BCKDHB	6.16	6.94	7.02	7.96	5.72	5.57	8.21	7.85
MAGOHB	6.91	6.85	6.19	6.75	7.09	5.57	8.78	7.95
LCMT2	6.31	5.68	6.24	5.16	5.38	5.58	5.31	4.88
MYO1E	5.85	6.45	6	5.61	5.93	5.58	5.93	5.58
CABYR	4.53	5.33	4.96	5.13	5.73	5.58	6.82	5.88
RTTN	5.21	4.7	5.25	5.49	6.07	5.58	7.08	6.11
ZBTB40	4.4	4.34	4.66	5.37	5.5	5.58	6.84	6.37
ZSCAN29	4.86	4.95	5.52	6.13	5.11	5.58	6.67	6.8
EPT1	4.45	3.75	5	5.75	6.16	5.58	9.06	7.42
CRLF3	5.92	6.02	6.06	6.95	6.85	5.58	9.46	7.58
PDGFRA	5.19	5.18	5.06	5.68	5.18	5.58	6.56	7.76
MORC3	4.84	4.99	5.72	6.41	5.12	5.58	8.44	7.94
SLC36A1	3.93	4.63	4.82	4.39	4.4	5.59	5.65	4.96
C7orf25	5.83	4.76	5.73	6.67	6.31	5.59	8.53	6.67
KRT13	4.75	5.11	4.79	4.79	4.43	5.6	4.87	5.22
PDSS2	4.86	5.06	5.08	5.14	5.16	5.6	5.66	5.22
ZSCAN25	6.52	6.02	6.11	6.79	5.85	5.6	5.5	5.38
ELMOD3	5.41	5.45	6.63	5.77	6.04	5.6	5.55	5.94
USP12	4.99	4.63	5.14	6.55	5.26	5.6	8.11	7.04
WDR20	5.27	6.3	5.94	6.16	6.7	5.6	6.48	7.08
TRAPPC11	6.17	4.94	5.44	5.83	5.82	5.6	8.02	7.44
C15orf41	5.86	5.61	5.03	5.72	4.81	5.61	6.19	4.71
HYAL3	4.85	5.12	5.17	4.6	5.67	5.61	5.13	5.21
TDP1	5.37	5.58	5.42	5.31	5.9	5.61	6.44	5.81
MSRB3	4.44	4.49	5.58	5.81	5.16	5.61	7.1	6.24
ACTR8	5.32	5.2	6.35	5.84	6.18	5.61	6.36	6.72
ZNF343	5.29	5.27	5.54	6.5	6.32	5.61	6.94	7.5
ZSWIM5	6.23	5.95	5.76	6.14	5.08	5.62	5.11	5.3
NAA30	4.6	4.44	4.81	5.53	5.06	5.62	6.45	5.59
AVEN	5.87	5.07	5.31	5.1	5.15	5.62	5.64	5.88
ZNF623	5.88	5.28	6.12	6.67	5.53	5.62	7.03	6.4
UBR2	5.02	4.83	5.68	6.04	6.06	5.62	7.58	6.55
DTD2	5.18	5	5.61	5.92	6.39	5.62	9.06	7.35
NME6	8.15	6.76	7.14	6.92	7.06	5.62	7.73	7.76
CEP70	5.12	5.53	5.79	6.17	5.55	5.62	10.4	8.1
PRAG1	6.22	6.41	6.14	6.27	5.36	5.63	4.22	4.46
B9D2	5.12	5.95	4.96	4.6	4.23	5.63	4.52	4.56
GINS3	5.69	5.51	5.21	4.17	5.58	5.63	5.35	4.78
TECPR2	6.22	6.07	6.36	5.97	5.67	5.63	5.56	5.41
ALG14	4	3.63	4.62	5.17	5.57	5.63	6.55	5.81
CYP2U1	5.01	4.91	5.12	5.87	5.96	5.63	7.33	6.87
CNEP1R1	5.99	5.35	6.81	7.07	6.62	5.63	9.1	7.67
PTPN13	6.18	5.62	7.32	8.97	5.87	5.63	10.37	8.71
MIER1	5.21	5.01	6.15	7.49	6.41	5.63	10.91	9.25
ITPKB	5.18	6.47	5.38	5.31	5.42	5.64	4.25	4.69
PRSS3	6.52	5.78	5.47	5.39	6.09	5.64	3.97	5.34
RAD54L	6.7	6.99	6.03	6.74	6.21	5.64	6.03	6.18
FCHSD2	5.41	5.3	5.43	6.2	6.53	5.64	7.54	6.78
GTF2IRD2B	5.53	4.69	5.13	5.26	6.13	5.65	6.41	5.28
PCK2	8.83	8.25	7.91	7.37	5.2	5.65	4.57	5.55
TRERF1	5.91	5.45	6.42	6.52	5.55	5.65	6.39	6
SPATA6	5.21	5.55	5.85	6.11	5.73	5.65	7.37	6.13
SP140L	6.87	6.47	7	7.68	6.26	5.65	8.54	8.39
UPF3B	5.32	4.86	5.65	7.69	6.42	5.65	11.12	10
ORC1	6.67	6.1	6.75	6.43	6.41	5.66	6.43	5.71
ZNF75A	4.87	4.03	5.9	6.53	5.01	5.66	9.07	5.81
NXPE3	4.62	4.4	4.87	5.2	5.9	5.66	6.81	6.81
FASTKD3	5.82	4.98	5.31	6.34	5.65	5.66	9.53	7.85
DICER1	4.77	4.54	6.2	7.51	5.83	5.66	9.53	8.12
UGT8	6.38	5.53	6.71	8.07	6.4	5.66	8.97	8.46
TTC14	4.69	3.94	4.89	5.88	5.34	5.66	10.81	9.16
C17orf53	6.17	5.95	6.27	5.11	6.53	5.67	5.75	5.95
ZNF451	4.69	5.26	5.94	7.2	6.56	5.67	10.19	8.41
GPR75-ASB3	5.67	5.11	4.41	5.06	5.28	5.68	10.65	4.26
TMEM86B	5.52	5.19	5.72	5.37	5.59	5.68	4.8	5.14
GABRA3	5.85	6.25	5.41	5.98	5.7	5.68	6.68	6.05
EFL1	5.47	5.3	6.36	6.35	6.16	5.68	7.72	6.76
CLCN3	4.98	5.19	5.8	6.99	6.38	5.68	8.69	7.89
IGFLR1	7.25	6.89	6.48	6.29	5.39	5.69	4.81	4.41
STK40	6.17	6.77	6.58	6.67	6.13	5.69	5.09	5.43

PPIP5K1	5.7	5.55	6.4	5.92	4.96	5.69	5.97	5.54
NFIL3	6.73	7.21	7.01	6.99	5.37	5.69	6.71	6.06
SLC44A5	5.01	5.12	5.76	6.86	5.05	5.69	9.67	7.16
AASDHPPT	5.39	5.56	6.24	6.6	6.29	5.69	8.43	7.43
CCDC103	6.49	6.14	5.57	5.36	4.84	5.7	4.11	4.74
WWOX	6.55	5.73	6.33	5.28	5.5	5.7	4.57	4.84
C1R	7.39	8.24	7.23	6.97	4.95	5.7	5.48	5.21
C20orf196	6.15	7.48	7.17	6.94	5.77	5.7	5.92	5.82
CIT	5.13	4.99	5.43	4.89	5.71	5.7	6.18	6.09
FOXJ2	5.76	5.98	5.65	5.56	5.58	5.7	6.11	6.23
CEBPG	4.5	4.42	5.2	5.92	5.49	5.7	6.89	6.61
AHCYL2	5.32	5.55	5.56	5.9	6.25	5.7	7.59	7.07
ARL4A	5.09	5.59	5.32	5.54	5.82	5.7	7.95	7.08
MOB4	5.59	5.06	6.08	7.03	5.75	5.7	9.94	7.53
ANKS3	4.81	4.88	5.05	5.55	5.44	5.71	5.81	4.87
GATA4	6.05	6.06	5.64	5.58	5.77	5.71	4.09	5.2
TTC39C	4.98	5.35	5.16	5.31	5.53	5.71	4.75	5.23
NOL12	4.98	5.21	5.63	5.38	6.01	5.71	5.88	5.45
SIPA1L2	5.32	5.66	5.58	5.36	4.89	5.71	5.51	5.93
SLC9A7	4.18	4.54	4.36	5.34	5.46	5.71	5.89	6.43
GLS2	6	5.38	5.32	5.09	5.96	5.71	5.75	6.52
E2F7	5.32	4.8	5.97	5.85	6.05	5.71	7.52	6.68
TBC1D15	5.43	5.11	5.67	5.96	5.74	5.71	8.99	6.82
KIF16B	5	4.77	5.66	6.62	6.14	5.71	7.6	7.11
PDE7A	7.22	6.15	7.03	7.93	5.62	5.71	9.19	7.82
C14orf93	5.03	5.61	5.11	4.84	6.13	5.72	4.84	5.92
SLC22A5	5.28	5.57	5.41	5.94	6.22	5.72	6.2	5.95
PLEKHA8	5.21	4.83	6.1	6.16	5.6	5.72	8.22	6.78
GDAP1	5.07	5.09	5.46	6.12	6.63	5.72	8.77	7.25
MINA	4.85	3.92	5.06	6.75	5.88	5.72	8.25	7.33
FAM111A	5.35	4.93	6.77	8.18	5.65	5.72	10.5	8.48
SEMA6C	5.65	5.55	5.7	5.23	5.37	5.73	4.5	5.32
NAIF1	6.26	6.4	5.43	5.78	5.93	5.73	5.62	5.46
KIAA1462	4.92	4.61	5.34	5.18	5.98	5.74	6.15	5.76
RPP40	5.08	4.47	5.05	4.31	6.56	5.74	7.09	5.84
BCL2L2	5.42	5.09	5.42	5.41	5.31	5.74	6.84	5.9
IL15	5.87	5.92	6.47	7.28	5.7	5.74	5.91	6.57
OFD1	4.7	4.15	5.08	6.95	5.99	5.74	10.15	7.24
TCF4	6.84	6.4	7.81	8.86	5.84	5.75	9.07	8.35
IREB2	4.66	4.58	5.57	6.8	5.63	5.75	9.71	8.4
LEO1	5.57	5.34	6.71	7.45	6.63	5.75	9.14	8.49
PPCDC	5.39	5.07	5.76	5.48	5.44	5.76	6.64	4.83
CDH24	6.49	6.28	6.19	6.73	5.29	5.76	5.51	5.49
GATA3	7.07	6.85	6.34	6.97	5.11	5.76	5.01	5.63
MLLT4	6.06	5.37	6.44	6.46	5.68	5.76	7.51	6.49
PEX7	6.95	6.56	6.79	7.56	6.78	5.76	7.97	7.21
KCTD18	5.96	5.64	6.23	6.41	5.96	5.76	7.07	7.44
MPHOSPH8	5.75	5.11	6.06	7.89	6.27	5.76	8.23	7.81
NIN	5.6	4.44	6.03	6.89	6.08	5.76	9.34	7.95
CAPRIN2	4.6	3.93	5.86	6.71	5.86	5.76	9.91	8.55
ZNF629	8.7	9.06	8.09	7.81	5.16	5.77	4.94	5.54
USP35	5.27	4.72	5.1	4.6	5.57	5.77	5.87	5.7
C1orf21	6.18	6.06	6.45	6.3	5.67	5.77	6.21	6.51
TAF3	5.11	4.94	5.09	5.69	6.24	5.78	5.73	5.63
FAM185A	4.96	3.77	5	4.8	5.47	5.78	6.89	5.71
BBS4	5.4	5.34	5.35	5.83	7.19	5.78	7.36	6.51
LRIF1	5.99	6.3	6.93	7.28	6.53	5.78	8.65	7.88
ADGRG6	5.27	4.98	6.14	7.11	5.77	5.78	9.25	8.21
ARHGEF39	5.19	5.07	6.32	3.95	5.63	5.79	6.94	4.54
SAP30L	5.75	5.04	5.55	5.95	5.97	5.79	5.93	5.65
CASP7	6.1	6.04	6.34	6.27	5.91	5.79	6.38	5.87
TIAM1	6.53	5.98	7.09	6.82	5.84	5.79	6.61	6.2
CWC25	6.66	5.92	6.36	6.57	6.99	5.79	7.62	7.52
RNF146	5.58	4.85	6.32	7.17	5.55	5.79	8.78	7.95
FGFR1OP2	7.19	6.66	6.75	6.22	7.83	5.79	7.98	8.88
QPRT	8.93	9.54	8.25	8.41	4.68	5.8	5.17	5.32
OIP5	4.59	3.57	4.29	4.01	4.85	5.8	6.22	5.59
ZFP41	6.56	6.72	6.96	6.44	6.37	5.8	5.83	5.81
LAYN	4.07	4.44	4.97	4.82	6.18	5.8	5.68	6.3
IMPA1	5.07	5.45	6.05	6.35	6.63	5.8	9.06	7.59
STYX	4.44	4.1	5.08	6.98	5.35	5.8	9.17	7.92
ZNF500	6.13	6.33	6.53	5.79	5.2	5.81	5.53	5.37
ZNF627	6.47	6.32	5.94	5.95	6.81	5.81	7.02	6.05
IFT57	4.95	5.31	5.31	5.48	5.91	5.81	7.29	6.25
WIPF1	6.04	6.17	6.19	6.96	6.02	5.81	6.71	6.55
E2F3	4.95	5.43	5.8	6.32	6.11	5.81	8.12	7.11
EED	6.59	6.61	6.19	6.14	6.95	5.81	8.04	7.17
PRDM2	6.79	6.9	7.16	7.98	6.19	5.81	8.07	7.88
OSGIN2	4.83	4.73	5.75	6.68	6.71	5.81	8.94	8.21
MAP4K5	5.66	5.54	6.45	8.09	6.15	5.81	10.84	9.31
GAPVD1	5.34	4.91	5.87	5.52	5.87	5.82	7.44	6.35
MANSC1	7.36	7.29	7.42	8.17	6.47	5.82	8.3	7.39
BRCA1	5.02	5.05	5.69	7.42	6.11	5.82	10.08	7.46
HPF1	5.99	5.91	6.51	7.31	7.11	5.82	8.88	7.66

PALB2	5.07	4.36	5.5	6.48	6.06	5.82	9.95	8.09
SEC24A	5.65	6.12	6.65	7.84	5.88	5.82	8.06	8.18
CCDC142	5.46	5.29	5.48	5.85	5.98	5.83	6.36	6.06
DIP2B	4.98	4.91	5.42	4.96	5.4	5.83	6.46	6.08
PXMP4	5.74	5.51	5.86	5.96	5.64	5.84	6.02	5.61
ELMSAN1	6.33	5.91	6.38	6.85	6.01	5.84	5.89	6.31
MTRF1L	5.58	5.56	6.36	6.03	6.2	5.84	8.42	7.46
PRPF18	5.22	5.54	6.59	6.27	7.99	5.84	8.09	7.59
APBB1	5.43	5.77	5.64	5.02	5.59	5.85	4.86	5.36
ADARB1	5.18	5.03	4.81	5.11	5.48	5.85	6.47	5.99
KIAA1549	5.37	5.08	5.43	5.6	5.33	5.85	5.51	6.05
TSEN2	5.43	4.52	5.05	4.94	6.39	5.85	7.8	6.29
STK38	5.93	5.94	6.45	6.81	6.32	5.85	7.33	6.59
TIPARP	5.76	5.19	6.1	7.64	5.87	5.85	8.12	6.94
SMAD2	5.89	5.81	6.59	6.74	6.09	5.85	7.23	7.29
MC1R	5.34	4.44	4.72	5.38	5.13	5.86	5.99	6.14
XRRA1	5.62	4.96	5.47	5.23	6.18	5.86	6.01	6.54
RNF170	5.4	5.23	6.23	7.34	5.73	5.86	8.68	6.93
TRUB1	5.24	5	5.6	6.35	5.94	5.86	8.92	7.37
SEC24B	5.87	4.66	6.35	6.89	5.76	5.86	8.19	7.93
FAM206A	5.34	5.04	6.37	6.9	6.73	5.86	9.82	8.03
UEVLD	4.98	5.51	6.61	8.07	6.33	5.86	10.48	8.18
YAE1D1	6	5.55	6.72	6.41	5.73	5.86	10	8.51
CSTA	9.29	8.88	8.82	8.1	6.83	5.87	8.59	6.49
TMEM254	6.54	6.06	6.2	7.38	6.71	5.87	7.61	6.82
ASF1A	5.27	6.61	7.66	6.14	6.43	5.87	7.56	7.29
FMR1	4.82	4.83	6.16	6.62	5.18	5.87	8.15	7.39
LRRC58	5.05	4.79	5.88	7.89	5.48	5.87	11.55	9.29
IBTK	5.07	4.71	5.59	7.45	6	5.87	11.03	9.44
STK32C	5.51	6.48	5.2	4.6	7.21	5.88	6.1	4.72
NINL	5.21	4.86	5.47	5.81	5.71	5.88	5.78	6.53
TRIQK	5.46	5.22	5.53	5.88	6.1	5.88	9.23	7.49
SACM1L	5.17	4.8	5.14	6.12	6.03	5.88	9.26	7.8
ZBTB38	5.21	4.6	6.63	7.54	6.06	5.88	9.41	7.85
PODNL1	9.14	8.83	9.11	8.14	5.08	5.89	4.38	4.12
SNX4	5.81	5.63	6.28	7.29	6.19	5.89	9.11	7.7
SENP6	4.89	4.7	5.58	6.51	5.96	5.89	10.19	8.11
RAB9A	6.87	6.16	6.17	6.43	7.19	5.89	8.68	8.4
NPIPB9	5.02	5.25	6.32	6.63	5.85	5.89	11.2	9.09
PROSER2	6.57	6.12	6.58	5.17	5.77	5.9	4.97	5.07
HPS5	5.4	4.71	5.72	5.93	5.67	5.9	7.44	6.52
ATG16L1	5.47	5.71	5.29	5.68	5.77	5.9	6.39	6.69
SIDT2	5.87	5.96	6.36	5.82	5.39	5.91	5.09	5.99
FKBP1B	6.2	6.94	7.02	6.04	5.49	5.91	4.96	6.64
DDAH1	4.36	4.48	5.05	5.7	6.69	5.91	8.89	6.71
HSF4	3.46	3.15	4.17	4.25	4.75	5.91	8.4	8.02
IL33	2.97	3.47	3.14	3.21	5.65	5.91	9.35	8.83
SAPCD2	4.76	4.3	4.42	3.92	4.79	5.92	4.33	4.78
TNFSF13	7.69	7.01	7.01	7.05	5.85	5.92	5.04	5.77
IPPK	5.27	5.37	5.54	5.1	6.56	5.92	5.79	6
MYLIP	6.08	5.49	6.07	7.1	5.09	5.92	6.59	6.93
CRYZL1	5.23	5.13	5.61	6.63	6.08	5.92	8.36	7.27
RIT1	6.27	5.74	6.59	6.93	6.75	5.92	7.72	7.64
ARHGAP22	4.42	4.13	4.57	4.61	5.65	5.93	5.8	5.34
AKAP13	5.54	5.08	5.9	6.38	5.92	5.93	6.48	6.26
IPP	6.36	6.14	6.08	6.64	6.05	5.93	7.31	6.71
DSTYK	5.62	5.65	6.4	6.34	6.08	5.93	6.61	6.95
RBL1	5.45	4.52	6.89	7.57	6	5.93	9.14	7.43
NDC80	5.84	5.38	6.16	5.77	6.46	5.93	9.16	7.56
SENP5	5.67	4.83	6.01	6.72	6.18	5.93	8.63	7.67
C9orf72	4.13	3.92	4.86	4.61	5.95	5.93	8.02	7.76
CNTNAP3B	5.71	5.47	6.3	6.6	5.47	5.94	7.76	6.79
DUS4L	6.35	5.59	5.83	6.08	6.11	5.94	8.87	7.72
RAB22A	4.88	4.71	5.68	6.49	6.48	5.94	8.69	8.13
MBNL2	5.24	5.14	5.86	7.02	6.16	5.94	10	9.17
C10orf35	6.85	5.86	5.07	5.92	6.2	5.95	5.46	5.65
MED7	5.22	5.08	5.88	5.69	5.58	5.95	7.6	5.99
IFT122	7.57	7.45	6.91	6.5	6.77	5.95	6.39	6.67
AAED1	6.16	5.38	5.43	6	5.54	5.95	6.96	6.89
SLC9A3	5.88	5.35	5.86	5.38	5.68	5.96	4.47	4.88
MCU	4.95	4.6	4.99	5.28	6.24	5.96	6	5.62
NEK8	4.54	4.84	4.14	4.51	5.39	5.96	6.02	6.07
MID1	6.3	6.12	6.51	6.5	5.67	5.96	7.35	6.86
RBM27	5.39	5.65	6.58	7.38	6.06	5.96	9.29	9.1
KDELR3	8.25	7.71	7.64	7.17	6.69	5.97	5.86	6.1
OTUD7B	6.2	6.48	6.45	6.62	6.03	5.97	6.88	6.53
EPHA4	6.41	6.22	6.47	6.9	5.76	5.97	6.68	7.01
FRYL	5.84	4.74	6.7	7.82	6.53	5.97	10.01	8.31
PRKD3	4.93	5.46	6.13	6.75	6.5	5.97	10.42	8.81
CEP72	4.82	4.6	5.06	4.82	4.63	5.98	5.24	4.94
P2RX5	3.92	4.74	3.9	4.04	4.77	5.98	4.76	5.13
UFSP2	5.52	5.28	6.76	6.11	6.62	5.98	7.97	6.76
ZC3HAV1L	5.87	5.57	6.28	7.18	5.62	5.98	6.71	7.06
MASTL	6.08	4.71	6.22	7.51	6.86	5.98	8.12	7.33
CHKA	4.55	4.25	4.91	4.71	6.18	5.99	6.77	5.54
SIM2	7.11	7.03	7.1	7.03	4.96	5.99	5.18	5.89

TOMM40L	7.05	7.06	6.73	6.46	6.74	5.99	7.46	6.8
PLEKHA3	5.49	5.05	5.61	6.13	6.59	5.99	8.11	6.97
TJP1	6.19	5.8	6.8	7.46	6.56	5.99	8.17	7.97
EXOC5	5.26	5.16	6.14	6.62	5.74	5.99	10.05	8.8
LOC107986805	6.02	4.53	5.41	6.33	5.56	6	7.42	6.83
ZSCAN21	6.35	6.48	6.65	6.35	7.17	6	7.02	7.14
C1orf53	5.02	5.17	3.42	3.57	4.37	6.01	2.74	3.29
DOK7	6.11	5.43	5.71	5.63	4.62	6.01	2.85	4.63
NFYA	5.83	6.11	6.31	6.64	6.03	6.01	6.94	6.65
CDC40	4.95	4.36	5.05	6.2	5.45	6.01	9.93	7.17
TIMM23B	4.43	6.03	5.23	5.99	5.24	6.01	7.73	7.4
PBRM1	5.3	4.72	6.86	6.9	7.16	6.01	9.89	8.37
PRKACB	5.63	5.46	5.84	6.93	6.8	6.01	10.05	8.69
MRM3	5.63	5.22	5.39	6.15	5.9	6.02	5.45	5.45
TANGO6	4.85	5.65	5.65	5.17	5.9	6.02	5.5	5.65
KCTD11	7.42	7.55	6.68	6.75	5.61	6.02	5.23	5.66
RASSF5	6.18	6.68	6.2	6.02	5.55	6.02	5.67	5.9
TMEM18	5.76	5.63	5.86	6.29	6.98	6.02	6.48	6.23
MRPL1	6.67	5.59	5.91	6.95	6.46	6.02	7.78	8.04
WDR37	5.94	5.72	6.24	6.43	6.05	6.03	7.59	6.49
WDR91	6.38	6.3	6.38	6.06	6.13	6.03	6.35	6.6
LRBA	5.62	5.09	6.08	6.94	6.67	6.03	8.85	7.84
NAA15	5.03	4.69	5.84	6.99	7.07	6.03	10.46	8.34
SLC25A20	5.57	6.12	5.88	5.22	5.54	6.04	6.6	5.72
POLA1	5.99	5.31	6.8	7.17	6.03	6.04	7.24	6.1
ACOT13	5.86	6.03	6.14	6.32	6.52	6.04	6.73	6.6
C21orf2	6.16	6.67	6.21	5.93	6.12	6.05	4.88	4.95
CYSRT1	4.86	3.55	4.67	3.65	6.46	6.05	5.06	5.82
TBP	5.81	6.02	5.11	6.32	5.97	6.05	6.41	5.94
SOBP	7.33	7.5	7.72	7.88	5.64	6.05	5.69	6.16
RBMS2	6.68	7.07	6.45	5.85	5.99	6.05	5.25	6.26
TNFRSF19	7.57	8.26	7.27	7.75	5.55	6.05	6.77	6.61
MFAP3	5.32	4.58	6.02	6.58	6.38	6.05	8.53	7.56
ARMC8	6.15	5.44	6.05	7.43	5.55	6.05	8.02	7.73
FAM110A	5.57	4.66	4.88	5.14	5.93	6.06	4.4	4.9
IFIT1	13.84	13.97	14.74	16.06	6.53	6.06	8.38	6.11
LYPLAL1	5.61	5.25	5.77	6.26	6.44	6.06	8.35	7.03
CRYBB2	6.71	6.4	5.62	6.4	7.75	6.06	5.47	7.33
CDC5L	6.29	5.97	7.15	7.82	6.67	6.06	9.17	8.27
ANAPC10	5.01	6.25	6.79	5.19	6.46	6.06	8.17	8.37
CD2AP	4.8	5.18	5.72	7.64	6.85	6.06	11.03	10.55
BCORL1	7.21	7.71	7.26	6.69	5.61	6.07	5.27	6.01
CASP1	5.61	4.67	6.5	6.52	4.77	6.07	8.17	7.63
ZBTB11	5.09	4.37	5.44	6.26	5.82	6.07	8.9	7.77
DDX52	5.79	5.24	6.23	6.95	7.04	6.07	8.87	8.33
MBLAC1	5.94	6.21	5.99	5.27	5.18	6.08	4.03	4.38
CDPF1	6.04	5.67	6.7	6.13	7.41	6.08	5.78	5.36
KLRC3	7.02	6.76	7.95	9.75	7.42	6.08	9.15	8.48
CASP6	6.18	6.63	6.43	6.87	6.13	6.09	7.65	6.31
FBXL17	5.79	4.45	6.26	6.49	5.16	6.09	6.74	6.64
PEX2	6.91	6.14	7.18	6.93	6.64	6.09	7.33	6.93
AQR	6.04	5.32	6.76	6.54	6.16	6.09	8.66	7.6
SPDYE2B	4.72	4.56	5.14	5.74	6.13	6.09	9.64	7.7
ZNF131	5.82	6.15	7.09	7.73	6.13	6.09	10.2	8.99
DPH6	6.78	4.21	7.2	6.45	7.1	6.1	7.71	6.28
IQSEC1	5.88	5.73	5.99	6.19	6.18	6.1	6.17	6.6
DIRAS1	6.92	6.21	5.85	5.95	6.11	6.1	5.41	6.95
SLC2A11	6.32	6.29	6.8	5.98	6.44	6.1	7.32	6.99
ZNRF2	6.1	5.06	6.11	5.43	5.9	6.11	6.49	4.98
ALKBH3	6.08	5.78	5.53	5.59	5.02	6.11	6.36	5.48
KPTN	5.9	6.17	5.4	5.62	7.28	6.11	4.85	5.7
PARS2	5.97	6.49	5.35	5.46	6.82	6.11	6.18	5.79
SPECC1	5	5.4	5.34	5.22	6.02	6.11	6.63	5.82
TYW3	5.41	5.41	5.22	6.07	6.25	6.11	9.36	7.44
CHD1	5.46	5.26	6.13	7.59	6.01	6.11	10.67	9.46
ATP9B	5.18	5.43	5.49	6.01	5.64	6.12	5.7	5.58
C22orf46	6.43	5.35	6.7	7.32	5.9	6.12	6.62	6.86
KNTC1	6.29	5.26	6.32	8.01	6.26	6.12	11.29	8.18
SIKE1	5.02	4.74	5.4	6.91	5.92	6.12	10.98	9.26
TYMSOS	6.04	6.07	5.48	5.13	5.97	6.13	5.5	5.97
CISD1	5.61	6.71	6.16	6.43	7.06	6.13	8.12	6.29
NDUFAF7	5.08	5.27	5.56	6.12	5.99	6.13	8.7	6.97
CLN5	6.22	5.31	6.91	6.7	6.72	6.13	7.52	7.35
OSBPL8	5.3	5.27	5.84	6.95	6.61	6.13	11.21	10.16
TMEM121	7.43	7.89	7.5	5.86	5.19	6.14	3.58	4.58
PSMC3IP	6.35	6.96	6.58	6.3	7.11	6.14	7.79	5.65
ADAMTSL5	6.12	5.53	5.09	5.94	6.15	6.14	4.95	6.36
HLCS	5.88	5.86	6.07	6.9	5.64	6.14	6.81	6.99
ARHGAP5	5.05	4.92	6.2	6.99	5.79	6.14	10.86	8.97
SHROOM2	6.83	6.27	6.46	6.6	5.84	6.15	5.47	5.83
FBXL8	5.2	4.03	4.93	5.37	4.78	6.15	5.5	5.89
ANKEF1	6.05	5.71	6.44	6.61	6.44	6.15	7.83	6.77
RBM12B	5.85	5.41	6.66	7.55	6.28	6.15	9.45	8.75
ZNF653	5.48	6.44	5.42	5.61	5.16	6.16	4.62	4.87
ORAOV1	4.58	4.73	4.47	5.05	5.89	6.16	7.07	6.25

PLXDC2	6.12	5.86	6.98	7.2	5.94	6.16	6.93	7.01
LOC101929839	5.76	5.88	4.49	7.23	6.76	6.16	6.02	7.31
TCF12	6.13	5.56	7.29	8.35	6.41	6.16	9.83	8.1
MED23	5.86	5.32	6.95	6.5	6.49	6.16	9.3	8.58
RUSC2	5.91	6.41	5.64	5.63	6.17	6.17	5.44	5.92
DDTL	5.35	5.56	4.44	6.09	6.96	6.18	6.9	6.06
ACYP1	6.38	6.21	4.63	6.85	6.18	6.18	8.54	6.26
C6orf99	4.9	4.76	3.88	6.67	6.28	6.18	6.31	6.55
AKAP11	5.98	5.38	7.2	8.27	6.51	6.18	13.26	10.28
PID1	4.13	3.69	4.52	4.58	6.76	6.19	6.57	5.52
FKRP	6.69	7.29	6.07	6.45	5.77	6.19	5.55	5.7
TSC22D2	5.91	5.91	6.46	7.24	5.78	6.19	6.03	6.43
TMCO4	6.39	7.36	7	5.17	5.97	6.2	4.2	5.16
KLHDC9	7	7.79	6.65	6.6	6.4	6.2	5.8	5.38
C2orf81	6.54	7.13	6.71	6.01	6.21	6.2	5.57	5.79
LDOC1L	6.32	6.32	5.94	6.02	6.38	6.2	5.91	5.98
SLC35F2	5.06	4.88	5.47	5.33	6.21	6.2	6.6	6.2
C8orf88	5.82	5.22	4.8	6.23	6.31	6.2	8.2	6.57
C3orf14	6.91	5.36	7.73	7.39	6.98	6.2	8.28	7.57
UBXN8	4.64	5.08	4.62	5.27	6.16	6.2	7.99	7.61
SDAD1	6.05	6.11	6.75	7.43	7.75	6.21	10.07	9.13
CDAN1	5.91	6.67	5.26	5.44	6.11	6.22	5.42	5.61
AGAP1	5.96	6.23	6.13	6.49	5.98	6.22	6.3	6.4
SOCS2	5.46	5.94	5.84	6.22	6.7	6.22	7.58	6.67
GFPT2	6.87	6.89	6.51	6.4	6.82	6.22	6.17	6.86
AVL9	5.37	5.28	6.5	6.99	6.03	6.22	8.27	7.58
WDR47	6.22	5.55	7.29	8.25	7.33	6.22	9.31	8.75
C5orf22	5.79	5.42	6.67	7.71	7.51	6.22	9.73	9.01
PPP3CC	6.05	6.18	5.75	6.09	6.27	6.23	7.21	5.72
TMEM40	8.19	6.61	7.17	6.03	7.49	6.23	5.87	6.43
TAF8	6	5.86	6.31	6.35	6.2	6.23	6.7	7.24
ACER3	6.78	5.84	6.25	7.6	7.26	6.23	8.3	8.4
APBA2	6.16	5.81	5.57	5.92	5.56	6.24	5.38	4.93
PLIN4	5.12	5.15	5.12	4.85	5.79	6.24	5.24	5.88
WDR53	5.22	4.17	5.69	5.34	5.44	6.24	6.63	5.91
C1orf112	5.81	5.72	6.98	6.41	7.11	6.24	8.24	6.92
LONRF1	5.14	4.79	5.45	6.65	6.04	6.24	7.45	6.96
DCP1B	5.83	6.83	6.52	7.41	6.02	6.24	6.57	6.99
FBXO34	5.73	5.66	6.23	6.51	6.55	6.24	7.05	7.07
SLC25A12	6	5.63	5.9	6.44	6.51	6.24	7.97	7.38
ANO9	4.98	5.07	4.71	5.59	5.55	6.24	7.67	8.15
RBM26	6.74	6.12	7.06	8.45	7.81	6.24	10.54	9.58
WWC3	6.3	6.25	6.67	6.4	5.94	6.25	6.55	5.93
DUSP5	6.4	5.66	5.58	5.58	5.65	6.25	5.77	6.57
SPTLC2	6.02	5.75	6.54	6.16	6.53	6.25	6.89	6.85
CHRNA5	4.92	5.71	6.62	6.14	6.12	6.25	6.64	6.91
TRIM16L	4.58	6.06	5.81	6.95	6.69	6.25	6.27	6.98
DMD	5.08	5.05	5.48	7.15	7.48	6.25	9.48	7.83
RNF141	5.29	5.38	7.19	7.48	6.25	6.25	10.31	9.04
TPRG1	6.74	6.22	6.73	7.34	6.95	6.26	10.05	7.41
TMEM191B	5.59	4.38	5.09	4.28	4.5	6.27	4.11	4.86
LOC107985911	5.99	7.6	6.1	6.03	6.19	6.27	6.45	6.2
RMND5A	5.18	4.82	5.91	6.53	6.25	6.27	7.67	7.23
LTB4R	4.39	4.17	4.45	5.05	5.9	6.27	8.53	7.46
HDHD2	5.73	5.38	6.56	6.79	7.03	6.27	9.42	7.73
FRAT2	4.85	5	4.7	4.94	5.48	6.28	4.82	5.19
NUPR1	7.8	8.18	7.81	7.35	6.31	6.28	5.49	5.19
C21orf58	7.36	6.27	6.45	5.88	6.27	6.28	7.57	6.46
HAUS2	5.33	4.71	6.1	6.54	7.25	6.28	7.99	6.51
ATP10D	5.39	5.4	6.05	6.49	6.62	6.28	7.68	7.51
DCUN1D4	5.5	5.87	5.94	7.72	6.1	6.28	11.51	9.92
LTB	5.68	5.21	6.84	5.66	6.24	6.29	4.4	3.92
TUFT1	7.01	7.06	7.71	7.23	6.11	6.29	5.4	5.93
PCOLCE2	5.59	5.89	6.15	5.82	6.93	6.29	7.08	5.93
ELF1	6.07	5.5	6.84	7.03	5.92	6.29	8.17	6.68
L2HGDH	4.67	4.75	5.11	6.31	6.08	6.29	7.94	7.68
GCLM	5.66	4.99	6.37	6.59	6.54	6.29	8.88	7.71
CFAP126	6.29	5.86	6.49	7.15	7.37	6.29	6.12	7.81
RIF1	5.05	4.74	6.26	7.07	5.97	6.29	10.74	9.09
PIK3C2A	4.21	4.51	5.05	7.52	5.41	6.29	12.7	10.6
MON1A	6.29	5.54	5.77	4.34	5.12	6.3	4.6	5.1
PAOX	6.71	7.85	6.53	5.97	5.8	6.3	4.2	5.27
GNG4	6.29	6.33	6.62	6.22	6.88	6.3	6.21	6.43
C11orf74	5.4	6.14	4.76	6.79	4.93	6.3	7.88	6.74
ERCC5	5.03	4.81	4.88	5.39	6.2	6.3	6.69	6.93
BRCC3	5.32	4.66	5.33	7.27	5.29	6.3	9.16	7.62
ZNF143	4.45	3.4	3.88	4.97	7.04	6.3	10.08	8.25
MAB21L3	4.87	4.7	5.92	8.14	6.13	6.3	12.17	11.12
SLC45A4	7.32	6.43	6.38	6.8	6.6	6.31	5.81	5.67
STK19	5.72	6.08	5.77	5.19	6.38	6.31	5.88	6.07
LEMD3	5.71	4.94	6.32	6.75	6.58	6.31	7.28	6.53
AKR1C1	5.96	5.77	6.29	6.18	6.74	6.31	6.75	7.29
CACHD1	7.8	7.48	8.34	8.17	6.22	6.31	7.78	7.69
SETD2	5.63	5.39	6.59	7.92	6.86	6.31	9.62	8.95
RASA3	6.3	5.56	5.73	5.47	5.59	6.32	5.58	5.81
TMED8	4.92	4.74	5.84	5.17	7.39	6.32	7.67	6.29

FAM157B	6.31	4.99	6.47	7.09	6.51	6.32	10.24	8.82
IQCJ-SCHIP1	6.45	6.21	6.97	6.1	5.6	6.33	5.54	5.11
CENPBD1	6.7	6.63	6.67	6.12	7	6.33	6.11	6.68
WARS2	5.86	6	5.88	6.54	6.45	6.33	8.03	6.98
CASK	6.76	5.89	7.22	8.01	6.78	6.33	8.09	7.81
HNMT	5.14	4.2	4.81	6.49	5.31	6.34	7.31	5.09
ZNF362	8.37	9.58	8.31	8.7	5.68	6.34	4.71	5.1
OXSM	5.44	5.58	6.22	5.72	6.06	6.34	7.71	6.2
KAZN	5.92	6.09	6.39	6.14	6.96	6.34	6.57	6.75
POLD3	6.33	6.08	7.1	7.26	6.68	6.34	7.35	7.23
MAP2K4	5.6	4.99	6.32	6.94	6.38	6.34	9.2	8.51
LOC107985476	5.32	5.09	5.71	6.13	4.83	6.35	5.97	4.64
LENG9	6.73	6.69	6.55	5.53	5.36	6.35	4.79	5.28
ZDHHC14	6.63	6.07	5.84	5.81	5.61	6.35	5.16	5.33
RTEL1	6.52	5.88	5.41	5.45	5.85	6.35	5.79	5.68
CNTROB	6.53	6.19	6.44	6.15	6.38	6.35	5.69	6.56
GLIS3	7.3	6.79	7.94	8.16	5.81	6.35	7.3	6.76
MLLT11	6.86	6.71	6.87	7.43	6.55	6.35	8.49	7.95
FUNDC2	6.46	6.47	6.67	6.38	7.64	6.36	6.1	6.81
HOXA7	6.86	6.7	6.8	6.82	5.95	6.36	6.27	6.99
PPM1A	5.59	5.3	6	6.61	6.1	6.36	8.31	7.89
AFTPH	5.83	5.46	6.57	7.63	6.62	6.36	10.43	8.96
AP3M2	6.7	6.63	6.79	7.3	7.07	6.37	8.45	7.2
RIOK1	5.84	5.52	5.91	6.74	7.1	6.37	7.91	8.19
FBXO28	5.65	5.53	6.25	7.29	7.11	6.37	9.83	8.5
PKIA	7.3	7.37	8.03	8.37	6.4	6.37	8.74	8.71
PCMTD1	5.48	5.48	6.02	7.61	6.17	6.37	10.27	9.2
KBTBD4	6.37	5.84	6.75	5.99	7.55	6.38	6.54	6.53
LSM5	5.59	6.43	6.45	6.97	7.16	6.38	9.1	7.86
MRPL19	5.69	5.5	6.46	6.54	6.93	6.38	8.87	8.46
ADGRA2	8.44	7.93	7.8	7.87	5.88	6.39	4.6	5.1
USP42	5.89	5.56	6.47	6.89	6.08	6.39	6.43	6.85
VPS13D	5.67	5.46	5.86	6.55	6.48	6.39	7.72	7.48
RPS6KC1	5.55	5.89	5.62	6.61	5.43	6.39	8.89	7.7
SNX2	5.16	5.13	5.87	7.27	6.62	6.39	10.37	8.49
FARS2	5.73	6.09	5.78	5.64	5.8	6.4	5.15	5.33
PPAN-P2RY11	4	5.93	4.81	5.88	6.46	6.4	3.31	5.96
KDELC1	5.79	6.02	6.71	6.79	6.5	6.4	7.4	6.34
RPF2	5.25	5.25	5.52	6.03	6.76	6.4	7.92	6.9
DUSP22	5.01	5.89	5.32	6.65	6.46	6.4	6.24	7.32
INPP4B	5.83	5.69	5.88	8.22	6.34	6.4	9.89	9.02
MSX1	5.63	6.02	5.26	5.71	5.55	6.41	5.04	4.99
HFE	7.14	6.61	6.51	6.64	5.84	6.41	6.17	6.31
ZNF691	6.13	5.61	6.66	5.93	6.73	6.41	6.96	6.46
SETD1B	6.32	6.55	5.93	6.68	6.13	6.41	6.26	6.79
ACAD8	5.47	5.85	5.41	5.7	6.46	6.41	6.24	6.93
YAP1	6.53	5.77	7.38	7.76	6.5	6.41	8.4	7.73
CDCA2	5.56	5.3	5.74	6.15	7.65	6.41	8.68	8.16
GALNT7	6.65	6.16	7.83	8.68	6.31	6.41	9.94	8.56
FAM69B	9.46	8.9	8.48	8.3	6.49	6.42	5.41	6.33
NMT2	7.58	6.13	8.06	7.62	6.72	6.42	7.67	7.55
TMA16	6.16	6.53	7.47	8.6	7.78	6.42	9.43	8.44
CLK1	6.4	7.6	8.82	8.64	6.1	6.42	11.54	9.31
UFL1	5.12	5.19	6.05	7.73	5.92	6.42	12.21	10.2
NEURL4	5.87	5.83	5.43	5.7	6.01	6.43	5.37	5.46
PCNX2	5.86	5.62	6.02	6.91	6.91	6.43	7.6	7.5
FAM208B	5.04	4.81	6.68	7.75	6.57	6.43	11.07	8.9
MPDZ	6.19	5.57	7.41	8.42	7.17	6.43	10.79	9.32
PPP4R2	6.06	5.95	7.15	8.08	7.04	6.43	11.96	10.14
MZT1	5.97	5.33	5.63	6.41	6.88	6.44	9.33	7.36
USP25	5.78	5.46	7.65	7.68	6.44	6.44	10.5	8.32
EDEM3	5.55	5.06	6.73	7.7	6.46	6.44	12.85	11.07
ZNF717	6.78	6.08	7.2	7.95	6.83	6.45	8.24	8.22
FBXO22	4.69	4.85	5.86	4.75	6.38	6.46	5.97	6.64
HSF2	5.32	5.82	6.58	6.7	6.41	6.46	8.23	7.77
ATP5S	7.18	6.64	6.55	8.59	7.92	6.46	8.69	7.87
C15orf57	6.9	6.31	6.19	7.53	6.96	6.46	5.29	8.13
UBXN2A	7.01	6.34	7.84	7.58	7.77	6.46	9.08	9.26
LOC102724813	5.8	5.01	5.51	7.18	5.78	6.46	10.5	10.24
ARL5B	6.05	6.13	6.71	7.49	6.07	6.47	10.41	9.23
ZNF646	7.28	6.89	7.1	6.95	6.49	6.48	5.42	5.95
TBK1	5.33	5.21	5.81	6.01	6.28	6.48	10.56	7.83
PLCE1	5.9	5.76	6.7	7.67	6.63	6.48	8.58	7.89
TWISTNB	5.93	4.94	5.85	7.68	6.39	6.48	10.12	9.04
CCNG2	5.93	6.15	6.58	6.7	5.94	6.49	9.42	7.59
SLC9A6	5.75	6.41	6.81	7.33	6.82	6.49	8.81	7.96
CASD1	4.97	4.72	5.45	6.63	5.96	6.49	10.88	9.06
DNAJC13	5.48	4.76	6.7	7.79	6.31	6.49	10.31	9.39
ATE1	6.61	4.96	7.08	8.66	7.5	6.49	9.62	10.12
CHST10	6.7	6.64	6.41	6	6.61	6.5	5.95	5.95
RNF103	5.63	5.02	6.14	6.22	5.89	6.5	7.74	6.47
ZHX3	6.08	5.9	5.85	6.27	6.34	6.5	6.87	6.62
PROSER1	6.46	6.59	6.44	7	6.99	6.5	7.8	7.57
AAGAB	6.6	6.03	6.51	7.33	8.06	6.5	9.16	8.27
GMCL1	5.75	5.33	7.04	7.56	7.29	6.5	10.61	9.2

E2F1	7.78	7.61	6.63	7.21	6.43	6.51	5.58	5.31
DNAJC16	5.75	5.61	6.44	6.54	6.44	6.51	7.75	7.11
MTERF4	6.77	6.44	6.79	7.39	6.57	6.51	7.85	7.33
MVB12B	6.78	6.19	6.29	6.87	7.12	6.52	6.09	6.62
LOC107987205	6.52	4.98	7.4	5.96	4.17	6.52	6.95	6.66
PAXIP1	6.58	5.88	6.81	7.24	7.52	6.52	9.3	7.58
CBLL1	6.28	6.55	7.35	7.47	6.97	6.52	8.06	8.25
APPBP2	6.63	5.94	7.11	7.97	6.73	6.52	11.06	8.81
MOSPD2	5.06	5.41	6.64	7.56	5.99	6.52	11.87	10.24
FBF1	6.67	6.15	6.29	5.97	6.58	6.53	5.86	5.95
MRPL22	6.06	5.89	6.38	6.36	6.76	6.53	7.73	6.43
C11orf95	7.17	6.79	6.46	6.69	6.79	6.53	6.09	6.63
NFRKB	6.14	5.2	5.81	5.84	6.42	6.53	6.93	6.72
RPP14	5.89	5.99	5.97	5.78	7	6.53	7.59	6.97
CNST	5.06	4.56	5.53	6.02	6.59	6.53	8.93	7.48
DBR1	6.44	6.38	6.34	6.83	7.27	6.53	7.87	7.67
BRWD1	6.57	6.15	7.18	8.44	6.73	6.53	9.72	8.32
CREB1	5.92	6.55	6.66	8.28	5.75	6.53	8.22	8.8
GPCPD1	6.66	6.66	7.84	8.85	5.97	6.53	10.21	9.19
ARHGEF18	7.02	7.28	6.36	6.75	5.79	6.54	5.62	6.02
TDRD7	7.58	7.7	7.69	9.07	6.43	6.54	7.92	6.1
FAM104B	5.65	6.41	6.02	5.28	6.29	6.54	8.47	6.65
MAPRE2	7.37	6.55	7.51	7.75	7.21	6.54	7.85	7.26
VPS8	5.88	5.55	6.18	7.29	6.55	6.54	9.39	7.81
LOC100996414	7.3	6.11	6.89	7.23	7.29	6.54	10.19	8.11
DBF4	5.23	4.82	5.7	6.38	6.49	6.54	10.27	9.16
HIBCH	6.38	6.09	6.87	7.43	7.5	6.54	9.62	9.61
RNF6	6.13	5.58	6.8	8.63	7.06	6.54	10.95	10.05
MAN2A2	6.27	6.85	6.89	6.9	5.66	6.55	6.09	6.51
GOLGA1	6.39	5.66	6.56	6.5	6.68	6.55	7.09	6.63
GNG11	6.48	6.9	7.37	6.68	6.64	6.55	7.43	6.76
GID4	5.2	5.83	5.38	6.32	6.32	6.55	6.93	6.99
KMT5B	6.13	6.22	7.04	8.59	7.35	6.55	9	8.68
DOCK5	5.84	5.47	6.57	7.46	6.57	6.55	9.8	8.93
ADAT3	6.94	7.19	6.11	6.09	6.06	6.56	4.48	5.1
DCUN1D5	5.57	5.3	5.66	5.93	6.72	6.56	5.78	5.86
RNF24	7.18	6.89	7.34	7.33	5.61	6.56	6.61	6.1
IKBKE	8.31	8.16	7.13	7.17	6.63	6.56	6.17	6.69
KHNYN	6.78	6.29	6.51	6.08	6.09	6.56	6.6	6.9
MTMR2	6.24	5.39	6.54	6.78	7.23	6.56	8.96	8.34
STAM2	5.88	5.33	6.41	7.28	6.34	6.56	10.16	8.54
DDX59	6.92	6.43	6.82	7.23	7.14	6.56	8.46	9.24
MMP15	8.52	9.3	8.37	7.79	6.36	6.58	5.3	6.34
ZRANB1	6.43	5.71	6.37	6.48	6.23	6.58	7.27	7.54
RNF144B	7.08	6.58	7.2	7.64	6.82	6.58	8.87	7.8
POLR3F	5.98	4.88	6.48	6.64	7.03	6.58	11	9.15
FRA10AC1	7.1	6.75	6.06	7.19	8.64	6.58	10.17	10.14
TMEM99	4.94	5.87	5.71	5.87	6.91	6.59	6.29	5.58
RPUSD4	5.64	5.3	4.88	5.2	7.29	6.59	6.27	6.54
TUBGCP4	5.86	6.19	6.6	6.22	6.53	6.59	6.45	7.02
EIF4ENIF1	7.32	6.85	6.2	6.59	6.26	6.59	6.64	7.17
FOXN3	7.47	6.58	7.41	7.89	7.36	6.59	8.04	7.53
USP6NL	6.4	5.95	6.62	7.63	7.18	6.59	8.51	7.81
PIK3C3	6.86	6.21	6.85	7.93	6.88	6.59	8.82	8.23
CUTC	5.56	5.93	7.73	6.3	6.66	6.59	8.08	8.82
CORO7	5.9	5.74	5.81	6.39	5.02	6.6	4.77	5.15
ANGEL1	5.94	6.56	6.08	6.58	6.33	6.6	6.26	6.77
PDP2	5.48	5.93	5.46	6.05	6.55	6.61	6.89	6.82
CCDC188	5.29	5.64	6.41	6.26	6.27	6.61	8.08	7.29
SIRPB1	5.27	7.06	6.04	5.81	5.91	6.62	4.42	4.44
MEF2B	5.54	5.52	6.22	6.12	5.66	6.63	4.12	5.12
PELI3	7.76	7.69	7.23	5.79	6.68	6.63	5.38	5.7
FBXO41	6.28	6	6.46	6.67	6.06	6.63	6.26	6.02
ALDH6A1	6.55	6.56	6.34	7.08	6.6	6.63	8.1	7.43
PRR3	6.68	6.73	6.86	6.14	7.47	6.63	7	7.49
PIGM	6.7	6.61	7.36	8.19	7.56	6.63	8.64	7.66
SLAIN2	6.53	5.88	7.21	7.87	7.29	6.63	9.18	8.67
TRMT1L	5.79	5.96	6.63	7.95	6.93	6.63	10.17	8.75
SP3	6.82	7.29	8.02	9.09	6.85	6.63	10.57	9.85
FMNL1	7.96	8.31	8.24	7.5	5.85	6.64	5.09	5.52
ZNF266	5.79	5.73	5.78	6.61	6.81	6.64	8.16	7.92
BCHE	6.06	6.26	7.1	6.91	6.16	6.64	9	8.36
CACUL1	5.93	5.51	6.68	7.34	6.92	6.64	8.85	8.55
RGMB	7.13	6.76	6.17	7.03	6.62	6.65	7.13	6.86
ZNF592	6.87	6.89	6.88	7	6.59	6.65	6.42	6.97
ZZZ3	5.98	5.65	6.22	8.23	6.54	6.65	10.55	8.97
RPS6KA3	5.7	5.1	7.24	8.34	7.26	6.65	11.57	9.47
SLC25A15	6.21	6.02	5.73	5.85	7.45	6.66	7.35	7.14
DBF4B	6.23	6.12	6.9	6.29	7.27	6.66	7.48	7.49
SLC39A8	6.64	4.79	5.19	6.39	7.14	6.66	8.79	7.52
MPPE1	7.38	7.54	6.27	7.13	5.74	6.66	6.6	7.65
RAPGEFL1	7.07	8.29	6.89	7.32	6.32	6.66	6.42	7.87
DYNC2LI1	6.76	6.18	6.47	6.99	7.54	6.66	9.51	8.16
NEK7	4.88	5.02	5.53	6.21	6.73	6.66	11.17	8.63
GCFC2	5.97	5.93	6.54	7.21	6.36	6.66	8.25	8.66

F3	6.62	6.59	7.34	8.15	7.42	6.66	10.57	9.14
NR3C1	6.39	5.5	7.31	8.55	7.37	6.66	10.36	9.36
ZNF117	4.73	4.41	5.85	7.04	5.78	6.66	14.88	12.1
PARD6A	6.65	5.37	5.94	4.22	6.27	6.67	5.67	4.96
KCTD21	5.84	5.96	6.62	6.25	6.6	6.67	5.99	6.27
CC2D2A	6.17	6.82	5.76	6.93	5.91	6.67	7.3	6.77
IFNAR2	8.1	7.63	7.96	9.55	7.53	6.67	7.45	8.53
CUL2	5.63	5.48	6.66	7.57	6.81	6.67	10.03	8.58
GRAMD3	8.46	8.88	8.85	10.37	7.51	6.67	9.57	9.83
TIPIN	5.61	4.93	5.89	6.41	6.37	6.68	7.12	6.85
SPIRE1	5.06	5.56	5.36	6.65	7.51	6.68	7.33	7.76
ARID4B	6.42	6.29	7.39	9.7	6.53	6.68	12.53	11.52
ELOVL6	4.47	4.57	6.85	7.64	6.11	6.69	9.97	8.35
SLC7A6	5.35	5.16	6.31	6.28	6.76	6.69	9.25	8.38
TTK	5.82	5.87	5.64	5.59	7.07	6.69	10.31	8.64
RAB3GAP2	5.69	5.28	7.19	8.24	7.48	6.69	11.7	9.3
FLVCR1	5.83	5.47	6.75	6.4	5.84	6.7	8.45	7.09
PET117	6.59	5.61	7.32	5.66	6.76	6.7	7.52	7.39
USP47	5.89	5.6	7.39	8.85	7	6.7	11.14	9.56
SOCS3	6.25	6.5	6.2	5.93	6.05	6.71	5.77	5.84
OSBPL5	9.57	8.7	8.75	7.89	6.05	6.71	5.24	6.38
MED13L	6.04	5.56	6.7	7.86	7.03	6.71	9.1	8.63
PPARG	7.18	6.95	7.21	6.84	7.71	6.71	7.54	8.67
UBXN7	5.66	5.27	7	7.54	6.41	6.71	9.04	8.78
PLEKHA2	7.24	7.04	7.57	8.1	6.5	6.72	7.42	7.19
KIF13A	7.22	5.73	6.53	7.77	7.48	6.72	9.82	7.5
PHF3	5.69	5.24	6.94	9.09	6.74	6.72	12.65	10.79
MZF1	6.33	6.15	5.18	6.2	6.42	6.73	6.65	5.77
SFI1	8	7.53	6.95	6.52	7.17	6.73	7.04	6.81
UBE2J1	5.92	5.92	6.18	6.88	6.45	6.74	7.69	6.98
INTS12	5.91	5.98	6.56	6.04	7.35	6.74	9.59	7.6
APPL1	5.88	5.26	7.06	7.94	6.76	6.74	10.1	9.79
BHLHE41	8.36	8.25	8.64	8.14	7.32	6.75	7.78	7.34
NKTR	5.83	5.17	6.51	8.49	6.02	6.75	13.99	11
ZNF503	8.06	8.38	7.82	7.85	5.77	6.76	5	5.27
PROSER3	7.08	7.16	6.26	6.2	5.91	6.76	5.8	6.57
PRKAG2	4.9	5.3	5.46	6.03	7.08	6.76	8.65	6.58
EXOC1	6.66	5.43	7.46	7.52	7.11	6.76	10.72	9.51
LIAS	6.74	6.23	7.2	8.13	8.9	6.76	11.93	10.98
CDC25C	5.29	5.08	5.83	5.61	6.77	6.77	6.37	6.12
PCDHB8	7.17	6.91	6.83	7.89	6.23	6.77	6.25	6.54
ARSJ	6.58	6.31	7.25	7.89	7.06	6.77	8.5	7.43
PCNX1	6.83	6.59	7.37	8	6.98	6.77	10.84	8.76
PEX1	6.18	5.84	6.61	7.57	7.43	6.77	11.24	9.05
ATG12	6.24	5.91	6.92	8.05	7.41	6.77	10.74	9.13
PHLDB2	6.5	6.04	7.36	8.54	6.65	6.77	10.86	9.31
MAGEA2	12.56	4.29	9.92	9.23	9.25	6.78	6.84	1.29
FAM109A	8.56	7.21	6.81	7.41	7.23	6.78	5.87	5.27
ESPL1	6.54	6.21	6.85	6.3	6.82	6.78	6.15	5.65
SLC29A3	6.43	6.37	6.31	5.72	6.45	6.78	5.95	6.34
SPRED3	6.71	6.9	6.35	6.64	6.11	6.78	6	6.84
HDAC8	7.8	6.92	7.17	7.17	7.13	6.78	8.09	8.54
BOK	6.76	6.07	5.34	6.05	7.04	6.79	5.33	6.26
PDLIM5	6.46	5.75	7.47	8.06	6.77	6.79	8.69	8.1
ZNF626	6.4	5.85	7.06	7.19	7.22	6.79	9.2	8.78
THOC1	5.79	5.72	6.03	7.42	6.73	6.79	10.94	10.91
MKS1	7.37	7.33	6.39	7.52	7.1	6.8	6.87	7.3
MAK16	5.68	4.51	5.89	7.12	6.64	6.8	9.71	7.9
ARHGEF3	5.8	6.2	6.94	6.9	6.81	6.8	8.01	8.03
CDK17	5.95	5.74	6.98	7.66	7.34	6.8	10.27	8.3
TATDN3	5.85	5.51	5.57	7.35	6.35	6.81	7.84	6.57
RCAN1	5.27	5.77	6.57	5.74	6.16	6.81	5.93	6.58
UNC5B	6.73	7.11	6.58	6.5	6.6	6.81	6.07	6.81
RCAN3	5.46	4.92	5.46	6.82	7.31	6.81	6.83	7.03
HIST1H2AC	8.19	9.81	11.46	9.49	5.26	6.81	5.33	7.43
NDEL1	6.97	6.83	7.3	6.85	7.41	6.81	8.24	8.03
ACSS3	7.08	6.72	7.84	9.11	7.95	6.81	10.17	8.47
BORCS5	5.93	7.8	6.35	6.85	6.7	6.81	7.38	8.64
CEP63	6.21	5.31	6.98	8.33	6.14	6.81	9.07	9.11
USP24	6.26	5.3	6.7	7.47	7.46	6.81	10.28	9.42
ATRX	5.93	5.91	7.23	8.6	7.19	6.81	12.88	11.59
PPP1R3F	6.05	5.32	5.96	5.17	4.62	6.82	4.24	4.3
TEF	5.49	5.98	5.64	5.4	5.98	6.82	5.27	5.65
MMP24	6.51	6.13	6.18	5.83	6.04	6.82	5.76	5.84
ARNT2	6.85	6.72	6.56	6.97	6.97	6.82	6.67	6.62
CCNT1	6.27	6	6.61	7.51	6.89	6.82	8.78	8.02
RNF168	5.9	5.41	7.05	8.07	7.44	6.82	9.15	8.7
ZNF195	5.46	6.94	6.25	6.55	6.72	6.82	10.03	8.78
KIAA1468	6.51	6.72	7.22	8.18	7.3	6.82	10.38	9.22
MAGEB2	6.12	6.76	6.8	6.29	6.95	6.83	7.11	6.66
ZNF558	7.17	6.03	6.83	8.24	6.74	6.83	10.51	8.39
CSAG2	4.75	6.01	2.68	5.27	6.4	6.83	15.2	14.59
RHBDF1	6.1	5.91	5.93	6.49	6.92	6.84	6.35	6.98
ZAK	5.08	4.71	6.74	7.02	6.73	6.84	10.06	7.53
CT45A10	5.76	7.55	8.38	7.7	8.06	6.84	10.81	9.81
ZNF319	7.8	6.73	7.01	6.7	6.98	6.85	5.58	6.29
ZNF707	8.34	6.99	7.81	7.31	6.26	6.85	6.61	6.34

PPP2R5B	7.54	7.98	6.58	7.57	6.53	6.85	6.14	7.18
C2orf47	6.03	6.23	7.26	7.2	6.67	6.85	6.99	7.31
SLC25A42	5.98	6.78	6.41	6.4	6.47	6.86	6.14	5.55
PIGW	5.35	4.98	5.87	6.21	6.41	6.86	7.7	7.59
TSSK3	5.61	4.91	6.25	7.56	7.03	6.86	8.4	8.67
FAM172A	5.2	4.69	5.48	6.49	7.33	6.86	9.59	8.67
EPS15	6.4	6.2	7.33	7.64	8.44	6.86	11.32	9.36
ZDHHC20	5.9	5.72	6.73	8.67	6.75	6.86	10.33	9.67
SREK1	6.13	5.81	6.83	8.65	6.77	6.86	11.72	9.76
ANKRD10	6.28	5.96	7.63	8.62	6.97	6.86	10.16	9.86
LOC107983970	8.48	8.18	7.52	6.79	6.92	6.87	4.87	4.48
OSGEP	6.64	6.11	6.71	7.41	7.74	6.87	7.14	6.45
SLC22A23	6.32	5.96	7.39	6.71	6.85	6.87	6.97	7.37
KDM5A	6.89	6.77	7.32	7.88	6.83	6.87	8.1	7.7
TRAPPC2	6.1	7.2	7.07	8.1	7.24	6.87	9.43	9.91
CAMK2D	5.54	5.81	6.25	6.42	5.99	6.88	7.45	6.86
ZFP36	7.84	9.2	9.28	8.74	6.84	6.88	7.12	7.66
ACAT1	5.42	4.36	6.3	5.51	6.76	6.89	7.47	7.1
PRDM4	6.31	6.15	6.31	6.68	6.56	6.89	7.54	7.16
CEP85	7.15	6.29	6.98	6.91	7.68	6.89	7.95	7.29
RNF8	6.35	6.36	6.04	6.76	6.55	6.89	7.55	7.55
ISY1	7.08	6.47	7.81	7.76	7.22	6.89	7.37	7.65
NBPF3	6.77	6.78	6.68	6.98	7.59	6.89	7.88	8.1
DONSON	6.36	5.64	6.03	7.06	6.51	6.9	8.01	6.34
RAD51D	6.39	5.88	6.28	6.73	6.51	6.9	7.08	6.84
FYCO1	6.84	6.64	6.84	6.97	6.88	6.9	6.93	7.17
C20orf96	6.87	5.82	6.1	6.99	5.88	6.9	7.25	7.22
RHPN1	6.36	6.35	6.33	5.82	6.7	6.9	7.07	7.48
HAUS6	6.54	6.21	7.07	8.37	7.41	6.9	13.1	10.28
C19orf25	6.2	6.21	6.43	5.53	6.4	6.91	4.3	5.55
STAP2	8.73	7.33	6.88	6.69	5.83	6.91	6.4	5.95
GABPB1	6.76	5.52	6.26	6.55	6.18	6.91	7.57	7.18
TENM3	6.46	6.39	6.77	7.39	6.55	6.91	8.18	7.21
PRPF40B	7.3	7.15	6.89	6.6	7.74	6.91	7.66	8.34
IL6R	4.87	6.01	5.5	5.4	6.26	6.92	6.72	6.49
ARHGEF7	7.45	7.81	7.31	8.48	8.08	6.92	8.26	7.62
ARG2	6.3	5.66	6.68	6.3	7.63	6.92	8.01	8.2
YTHDC2	6.42	5.77	7.63	8.25	7.71	6.92	12.43	10.5
SAP30	7.9	7.2	6.8	8.26	7.72	6.93	6.69	6.87
GNPNAT1	6.48	6.25	6.93	7.46	8.54	6.93	9.8	9.06
RNF122	9.41	9.99	8.13	8.46	7.43	6.94	5.86	6.33
BRPF1	6.44	6.63	6.45	6.5	6.65	6.95	5.6	5.65
DCLRE1B	7.27	6.03	6.08	6.61	6.75	6.95	7	5.73
C15orf61	5.38	5.43	5.01	5.81	6.51	6.95	4.96	5.98
ZFYVE1	6.46	6.51	5.99	6.39	5.71	6.95	6.41	6
MANBA	6.56	6.54	7.13	7.02	6.67	6.95	9	7.59
SPAST	5.86	5.98	7.46	8.79	6.33	6.95	10.51	9.72
ARHGEF5	7.39	7.72	7.69	7.37	6.82	6.96	6.43	7.1
XRCC4	6.83	8.06	7.09	7.55	7.29	6.96	8.6	7.75
AGTPBP1	5.66	5.42	6.76	7.52	6.41	6.96	10.8	8.59
TCN1	4.21	5.18	5.4	5.23	6.06	6.97	7.64	5.97
PPP1R21	6.42	6.91	6.85	7.02	7.09	6.97	8.81	7.73
S100A3	5.75	5.9	5.62	5.89	7.18	6.98	6.09	6.99
SOCS7	6.48	6.74	6.74	6.91	7.13	6.98	7.06	7.36
CLEC16A	6.15	7	6.43	6.05	7.76	6.99	6.25	6.84
SLC38A9	6.33	5.72	6.76	6.97	7.42	6.99	10.19	8.48
FBXW8	7.64	6.33	6.53	6.56	6.56	7	5.94	6.28
NPIPB4	5.42	4.18	6.54	8.24	5.96	7	9.55	8.82
NCAPG	6.09	5.49	6.92	8.22	7.22	7	12.14	9.36
COMMD10	7.24	7.58	8.81	8.85	8.26	7	9.06	9.37
VPS37D	8.34	9.3	8.11	8.07	5.91	7.01	4.57	5.56
CDC42EP2	6.83	7.27	7.27	6.81	6.65	7.01	5.71	5.78
TCEAL1	6.3	4.89	6.32	6.27	8.5	7.01	8.4	7.09
LMBR1L	6.92	7.92	7.71	7.78	7.73	7.01	7.85	7.41
NEDD4L	7.13	7.2	7.41	7.6	7.47	7.01	7.95	7.52
RUFY3	6.76	6.55	7.37	8.33	6.65	7.01	9.47	8.26
KAT6A	7.11	6.57	7.73	8.17	7.23	7.01	9.27	8.3
ALS2CL	4.83	4.74	4.49	5.45	7.14	7.01	8.6	8.5
UBA6	6.84	5.9	6.55	8.22	6.38	7.01	11.4	9.81
TRMT10C	8.11	7.54	7.2	8.22	8.44	7.01	12.92	10.03
TP53RK	6.47	7.14	6.15	6.17	6.87	7.02	7.58	7.03
TMTC4	7.08	7.99	8.16	8.06	6.7	7.02	8.57	8.17
MAST4	6.56	6.57	7.12	7.75	7.17	7.02	7.94	8.21
CHUK	5.41	4.96	6.33	6.93	7.44	7.02	9.32	8.76
HEATR5B	5.79	5.48	6.29	6.92	7.05	7.02	9.22	8.77
ANKRD28	6.28	5.56	8.07	8.78	7.38	7.02	10.83	9.61
CASP4	6.33	6.68	7.43	7.28	6.67	7.03	8.15	5.91
HAUS5	6.19	6.23	6.29	6.93	6.13	7.03	6.93	6.15
FBXO46	6.39	6.84	6.66	7.04	6.23	7.03	6.28	6.3
FGGY	7.72	7.65	8.2	7.58	7.08	7.03	8	6.6
LTB4R2	4.84	4.22	4.71	5.9	6.18	7.03	8.41	6.68
ADCY9	6.87	6.69	6.8	6.6	6.46	7.03	6.4	6.71
MAGEA9B	9.81	7.54	7.24	5.88	4.54	7.03	5	6.82
VIPAS39	7.35	7.13	6.71	5.98	7.51	7.03	8.68	8.73
UHRF1	8.99	8.04	9.06	9.06	6.42	7.04	6.67	6.28
GPR161	7.46	7.24	7.42	7.67	6.91	7.04	7.5	7.38

AKR1B10	5.34	5.88	5.22	4.6	7.06	7.04	7.64	9.04
UBTD2	7.34	7.09	8.63	8.13	7.95	7.04	9.45	9.09
HERC4	6.19	6.41	6.97	7.78	8.04	7.04	11.17	10.31
BCL6	7.74	7.37	8.79	8.32	6.77	7.05	8.03	7.52
TXLNG	5.64	5.1	7	7.47	7.17	7.05	10.32	8.86
P2RY11	6.41	4.96	5.71	4.86	5.69	7.06	7.24	5.03
MAOA	6.09	6.52	6.42	6.72	6.97	7.06	7.73	7.2
CASP8	7.64	6.91	6.88	8.18	8.36	7.06	9.94	7.86
MED6	6.59	6.27	7.43	7.48	7.66	7.06	8.49	7.99
RSBN1L	6.94	6.86	8.15	8.18	7.94	7.06	9.65	9.24
AGL	6.59	6.11	7.47	8.59	7.24	7.06	12.04	10.21
KIAA1147	6.32	5.96	6.75	7.94	7.98	7.06	11.91	10.37
STOML1	7.15	7.17	6.92	7.09	7.61	7.07	5.52	5.7
UQCC3	11.64	11.5	11.09	10.77	6.27	7.07	4.79	5.95
EGFL7	8.59	9.09	7.47	7.13	6.6	7.07	5.34	6.05
ALDH3B1	6.23	6.9	6.02	5.94	7.45	7.07	7.02	6.65
GNL3L	6.22	6.72	6.6	6.39	7.18	7.07	6.87	7.44
TMEM251	7.14	5.73	5.29	7.49	6.07	7.07	7.61	7.56
C1orf216	6.45	7.36	6.86	7.26	7.43	7.07	6.92	8.04
ETNK1	6.1	6.05	7.23	7.04	6.79	7.07	9.57	8.73
UBE2E2	5.94	6.66	7.26	6.37	7.03	7.08	7.27	6.84
PIGH	6.32	6.26	6.62	5.93	7.34	7.08	7.57	7.21
SIRT5	7.55	6.94	8.38	7.5	6.95	7.08	8.93	8.31
COMMD2	6.15	5.69	6.43	7.73	6.91	7.08	10.66	8.7
SPRYD7	7.34	6.82	7.64	8.06	7.84	7.08	9.03	9.06
SLC4A7	6.63	6.42	6.83	8.44	7.8	7.08	13.08	10.8
PRPF4B	5.93	6.19	7.02	8.31	6.8	7.09	11.78	10.28
SRGAP2C	6.52	7.31	6.83	8.5	7.23	7.09	10.73	10.62
CEP78	5.42	5.01	5.89	6.91	7.17	7.1	8.65	7.53
TFEB	6.55	6.36	5.67	5.78	6.25	7.11	5.61	5.33
RSG1	5.98	5.89	6.07	4.99	6.68	7.11	5.39	6.11
CNNM4	7.13	6.52	6.01	6.6	6.54	7.11	5.9	6.19
CCL2	7.17	6.65	7.78	9.72	6.22	7.11	8.13	6.29
ABHD6	7.66	7.79	7.23	7.7	8.12	7.11	6.78	6.6
EFHD1	9.1	10.59	9.12	8.46	7.77	7.11	7.3	8.2
FAM134B	7.34	6.12	7.44	6.96	7.15	7.11	9.12	8.63
ARMT1	6.59	7.14	7.94	8.15	8.52	7.11	11.39	9.96
RBFA	6.42	6.36	6.57	6.46	7.63	7.12	6.16	7.08
MSH5	6.1	5.19	5.82	6.37	6.77	7.12	9.09	7.37
C19orf12	6.52	6.58	6.57	6.51	7.09	7.12	7.32	7.49
ZNF609	7.85	8.78	8.74	8.51	7.81	7.12	7.94	7.9
RAVER2	6.99	5.92	6.56	7.14	7.71	7.12	8.29	7.97
DLGAP5	5.52	5.19	5.89	5.96	6.95	7.12	10.6	9.13
TYRO3	7.13	7.75	7.36	6.65	6.82	7.13	6.67	6.64
BTD	6.37	7.01	6.59	6.82	7.6	7.13	7.71	7.2
EYA3	6.84	7.07	8.01	7.77	7.51	7.13	9.84	9.01
RAB18	5.76	5.37	6.93	7.28	7.34	7.13	11.35	9.14
FBXO3	6.18	5.63	7.73	8.16	8.21	7.13	11.22	9.77
WNT5B	8.29	8.71	8.02	8.13	6.52	7.14	5.06	6.31
GATB	6.02	6.09	5.52	6.02	7.2	7.14	7.13	7.01
NICN1	5.92	6.57	6.26	6.04	6.35	7.14	7.36	7.17
DCAF5	7.74	7.78	7.91	8.73	6.86	7.14	7.61	7.84
DHX35	6.89	7.46	7.47	6.96	7.71	7.14	8.21	8.44
KLHL24	6.93	7.38	7.93	9.86	7.17	7.14	11.94	11.99
CABLES2	7.81	8.33	8.39	7.12	6.93	7.15	6.05	6.63
NDUFAF1	7.98	7.46	7.52	7.68	8.8	7.15	8.84	8.17
OXR1	6.82	6.56	6.87	8.6	7.58	7.15	11.2	9.49
KIF11	6	5.06	6.95	8.78	7.4	7.15	13.26	10.91
BUB1B	5.53	5.1	5.96	6.2	7.12	7.16	9.74	7.86
XIAP	7.15	6.75	8.14	9.35	7.23	7.16	10.62	9.59
PTBP3	6.14	5.74	6.87	7.8	7.64	7.16	12.34	9.96
EVPL	7.14	7	6.59	6.43	6.89	7.17	5.96	6.58
TRMT2B	5.87	6.53	6.29	6.47	6.91	7.17	6.97	7.77
ERC1	6.77	6.81	7.33	7.03	6.89	7.17	8.52	7.95
APBA3	7.27	7.19	6.52	6.33	7.57	7.18	5.38	5.56
FANCC	7.89	7.77	8.99	8.12	7.53	7.18	8.07	7.66
PKP2	7.08	6.35	7.76	7.25	7.59	7.18	8.61	7.76
SLC25A40	6.41	6.16	7.3	7.41	7.17	7.18	11.2	9.22
MNT	7.19	6.77	6.59	6.86	7.34	7.19	6.23	6.42
NET1	6.2	5.81	7.1	7.51	7.39	7.19	9.25	8.09
SLC35C1	7.75	7.8	6.45	8	9.11	7.19	6.93	8.97
S1PR5	8.37	8.04	8.45	7.45	6.97	7.2	5.37	5.83
FUK	7.13	7.15	6.71	5.93	6.57	7.2	5.58	5.88
OXCT1	5.88	5.5	6.18	6.65	7.25	7.2	9.02	7.23
NOL9	6.77	6.51	6.63	6.28	7.57	7.2	8.27	7.95
TMEM186	6.69	7.38	7.24	7.78	8.49	7.2	7.25	8.1
TMEM168	5.92	5.38	6.96	7.71	7.31	7.2	10.95	10.04
ARL17B	6.96	8.45	5.67	5.93	5.89	7.21	3.67	5.52
POPDC3	5.79	5.71	6.25	7.27	6.29	7.21	7.04	7.05
FLRT2	8.7	7.68	9.15	9.61	7.68	7.21	9.34	9.17
TMEM55A	8.33	6.54	8.49	8.16	7.21	7.22	8.14	8.78
TPP2	5.85	5.76	6.94	8.19	7.91	7.22	10.8	9.48
MKL2	6.85	6.43	7.95	8.45	7.55	7.22	10.2	10.01
EEPD1	6.9	6.44	6.83	6.89	6.92	7.23	4.91	5.05
LOC107985803	7.2	6.08	6.19	7.03	7.19	7.23	6.11	5.78
GLB1L2	6.94	7.85	6.6	6.4	7.44	7.23	5.99	5.84
PTGS1	8.33	9.7	7.88	8.38	6.9	7.23	7.02	8.62

DOHH	7.97	6.69	6.31	6.13	8.59	7.24	5.63	6.35
NECAP1	6.17	6.25	6.63	6.71	6.96	7.24	8.08	7.96
STRBP	6.19	5.91	6.9	8.01	7.73	7.24	10.89	8.78
ZBTB5	6.8	7.02	6.78	7.24	7.05	7.25	8.33	7.56
SBNO1	5.27	5.2	6.99	8.28	7.03	7.25	10.79	9.81
TTC7A	7.02	7.3	7.08	6.72	6.59	7.26	6.18	5.86
ZNF205	7.79	7.23	7.86	6.97	6.85	7.27	5.19	6.48
RNF115	7.42	7.88	7.25	8.64	6.45	7.27	6.19	6.81
WDR90	7.02	6.43	6.69	7.28	6.71	7.27	7.16	7.09
UBIAD1	7.61	7.76	6.94	7.44	8.03	7.27	7.45	7.57
PMS1	6.78	6.42	7.66	9.18	7.68	7.27	12.79	10
LOC100507507	7.18	7.31	7.02	7.17	5.9	7.28	5.45	6.31
UBE2Q2	5.63	5.39	6.78	6.48	6.48	7.28	6.55	6.41
PAFAH2	7.11	7.26	6.92	6.85	7.67	7.28	7.63	7.16
MCPH1	6.55	6.46	6.94	6.98	6.72	7.28	8.98	7.38
MTMR3	7.85	7.8	7.97	7.92	7.67	7.28	8.09	7.62
NREP	10.07	9	10.05	10.6	6.3	7.28	7.94	7.89
COL9A2	6.5	7.33	7.31	6.7	7.33	7.28	6.71	7.99
RAD1	7.07	6.99	6.97	7.11	7.28	7.28	8.87	8.37
KRIT1	5.86	5.79	7.31	8.05	6.68	7.28	14.8	11.07
KLHL5	6.54	5.95	6.92	8.4	7.3	7.29	8.78	8.29
TDG	6.04	5.94	7.1	7.14	7.78	7.29	11.09	9.41
ARL5A	6.93	6.54	6.27	7.05	7.49	7.29	12.83	9.68
MBTPS2	7.05	6.54	8.29	9.23	8.53	7.29	12.44	10.12
TBC1D25	6.59	6.72	6.7	6.73	6.9	7.3	6.28	6.67
PADI3	9.3	10.09	9.28	7.93	7.11	7.3	5.86	6.7
IQGAP3	6.06	6.24	6.21	5.77	7.41	7.3	7.86	7.05
NSMAF	7.33	6.67	7.02	6.69	6.92	7.3	8.48	7.98
QTRT2	6.37	5.36	6.54	6.52	7.12	7.3	8.91	8.23
PKN2	6.71	6.52	7.71	9.29	7.97	7.3	12.89	11.59
LRRC57	7.04	6.02	7.61	7.55	7.09	7.31	8.22	7.9
GEMIN7	7.95	8.46	8.67	7.04	7.3	7.31	6.47	7.92
ABHD17C	6.38	6.27	6.51	6.34	8.19	7.31	8.25	7.97
BORCS6	5.96	6.04	5.86	5.24	6.53	7.32	4.61	5.65
MED26	7.82	7.23	6.79	6.82	6.81	7.32	5.45	6.45
NBAS	7.26	6.96	7.18	6.59	7.24	7.32	8.03	7.13
ZNF830	6.94	6.47	6.47	6.95	6.28	7.32	7.79	7.2
UCN2	6.42	5.66	6.74	7.28	6.54	7.32	8.56	7.28
MTO1	5.56	5.43	7.01	6.69	8.26	7.32	8.31	7.46
SLC30A7	6.18	5.65	5.99	8.21	6.79	7.32	10.26	7.96
ABCB7	6.91	6.68	6.99	6.87	7.12	7.32	9.21	8.22
DNAJC22	7.95	7.64	7.12	7.77	7.45	7.32	8.29	8.7
LMNTD2	7.66	7.89	6.83	6.78	6.54	7.33	6.56	6.78
FAM173B	6.37	6.31	7.1	7.61	6.93	7.34	9.72	8.53
ATAT1	8.83	8.37	8.9	8.78	9.02	7.34	9.26	8.89
SHQ1	6.15	6.2	6.91	6.59	5.99	7.35	7.11	6.25
MLYCD	7.03	6.99	6.9	7.08	7.67	7.35	5.83	6.28
TRPM4	7.07	7.25	6.24	6.54	6.99	7.35	7.01	6.33
TRIM62	7.62	8.45	7.84	8.08	6.7	7.35	6.19	6.7
FAM193A	7.51	7.84	8.16	7.78	7.12	7.35	6.93	7.41
MAP3K7	6.22	6.14	7.34	8.35	7.3	7.35	11.51	9.02
ZMYM2	7.52	7.08	8.67	10.01	7.49	7.35	12.13	11.79
PIP4K2A	7.99	7.25	7.34	7.88	7.65	7.36	8.69	8.58
DOCK4	6.64	5.86	7.32	8.76	7.21	7.36	10.11	8.77
DTX3L	11.25	11.39	13.46	14.4	8.33	7.36	11.22	8.95
TAF1B	7.6	7.21	7.5	8.75	8.19	7.36	11.78	9.48
SMC5	5.21	5.13	6.73	7.8	6.37	7.36	13.72	10.32
PSD3	5.81	5.04	6.42	8.13	7.42	7.36	11.53	10.38
APLP1	4.87	5.65	4.97	4.99	7.23	7.37	6.12	6.1
MFSD2A	5.89	5.72	4.8	6.04	5.8	7.37	4.83	6.19
DAGLB	7.61	8.12	7.57	6.89	7.98	7.37	6.47	7.1
MCTP2	6.15	6.55	6.81	8.31	7.91	7.37	9.95	9.11
AP1S2	8.23	6.81	8.79	9.04	9.23	7.37	12.79	10.14
LRRC28	7.24	7.39	8.11	6.87	6.88	7.38	7.19	7.88
UTP3	6.21	6.05	7.55	7.5	8.34	7.38	9.48	8.23
GUF1	5.76	5.56	6.34	7.26	7.51	7.38	12.61	9.79
ISG20	8.43	6.81	7.78	7.13	6.92	7.39	6.09	5.86
CSTF2T	8	7.93	8.15	8.3	8.09	7.39	8.15	8.01
CMC4	9.5	10.55	9.21	10.88	9.34	7.39	7.69	8.45
MOCS2	7.2	5.54	6.74	7.2	8.7	7.39	11.3	8.57
LSM6	7.36	7.92	7.72	6.93	7.81	7.39	8	8.62
PHF21A	8.51	8.02	8.43	8.79	7.66	7.39	8.69	8.85
ZWILCH	6.45	5.95	7.38	7.27	7.75	7.39	12.64	10.21
ODF2L	6.21	5.48	6.49	8.49	6.73	7.39	13.27	10.83
ZNF785	6.56	7.22	7.34	7.5	6.52	7.4	7.51	7.07
IGHMBP2	7.4	8.42	7.61	7.65	7.29	7.4	7.04	7.21
ANKFY1	7.18	7.01	7.68	7.76	7.12	7.4	7.88	7.21
EIF1AD	6.99	6.59	7.17	7.67	7.62	7.4	7.75	7.39
SUPT7L	6.25	6.41	6.76	8.27	7.57	7.4	9.95	8.99
UNC13D	7.97	7.37	6.29	6.5	7.25	7.41	6.31	6.56
DMPK	6.64	6.43	6.6	7.24	7.15	7.41	6.14	6.78
ZNF584	7.27	7.88	7.44	6.83	7.56	7.41	7.42	7.58
RCOR1	6.91	7.19	7.62	7.96	7.25	7.41	7.95	7.74
DRAM1	5.42	4.8	5.83	6.28	8.71	7.41	9.76	8.81
RABEP2	8.01	8.06	7.59	7.3	8.37	7.42	6	7.52
APBB2	7.79	6.41	7.06	8.26	7.1	7.42	8.71	7.54
STX7	6.58	6.49	7.38	9.28	7.89	7.42	10.51	8.82
HOXA10	6.81	6.61	6.47	6.98	7.77	7.43	7.7	8.11

RALGAPA2	7.87	7.77	8	8.17	7.56	7.43	8.58	8.8
DDX58	13.56	11.56	14.67	17.84	7.24	7.43	11.77	9.63
ZBED6	6.52	5.43	7.26	10.14	7.09	7.43	12.31	12.64
ACAD11	6.25	6.63	6.85	7.25	7.35	7.44	9.01	8.69
CCDC59	6.32	8.13	7.25	7.93	8.01	7.44	10.5	8.79
RGS17	6.99	5.53	6.99	8.17	7.22	7.44	11.18	9.2
GMFB	6.84	7.58	6.72	7.57	7.78	7.44	11.23	9.91
SHISA3	7.3	7.01	7.16	7.57	7.33	7.45	6.68	5.43
SLC45A3	6.9	7.48	6.16	7.14	6.99	7.45	6.31	6.29
HEATR3	6.23	5.6	5.72	5.03	6.99	7.45	6.93	6.85
BLOC1S3	6.47	5.8	6.03	5.66	6.58	7.45	6.05	6.89
UCN	6.13	5.95	6.88	6.01	7.75	7.45	7.06	7.81
MOB3C	8.23	8.46	8.07	7.99	7.92	7.45	7.25	7.85
RBM33	5.92	5.94	6.92	8.01	7.53	7.45	9.84	9.41
TNIP3	5.73	5.78	7.02	9.12	6.71	7.45	14.27	11.28
ZNF628	5.87	5.57	5.26	5.04	7.12	7.46	4.83	5.24
TMEM102	8.46	8.71	8.36	7.15	7.24	7.46	5.95	6.25
BACE2	7.67	7.19	7.38	6.92	6.78	7.46	7.5	7.14
INTS7	8.24	6.96	8.03	7.95	8	7.46	9.49	8.18
CROCC	7.04	6.92	7.03	7.76	7.39	7.46	7.57	8.19
INIP	6.68	7.12	8.11	7.61	7.85	7.46	9.94	8.72
TFCP2	8.43	7.32	8.05	8.92	7.94	7.46	10.54	8.86
UBXN2B	6.17	5.59	8.09	9.59	8.84	7.46	11.49	9.66
KRCC1	9.1	8.93	9.12	9.79	8.04	7.46	10.59	10.17
MCM8	7.25	5.59	8.2	9.48	7.68	7.47	12.35	9.61
CREBZF	6.69	6.85	7.36	8.55	7.23	7.47	12.04	10.34
PAK1IP1	6.86	7.32	7.03	9.59	10.02	7.47	12.09	10.67
CDK8	6.16	5.45	6.88	6.73	7.7	7.48	8.4	7.74
PLEKHG2	8.11	8.13	7.71	8.57	7.09	7.48	6.84	7.92
FAM133B	6.67	7.32	8.31	9.05	8.11	7.48	11.79	10.62
C16orf59	7.59	5.99	6.42	5.79	7.88	7.49	6.32	5.2
KLHL29	8.11	7.27	7.27	8.08	7.82	7.49	7.21	8.06
SETX	7.07	6.31	9.03	11.23	8.15	7.49	13.16	12.21
CAPG	7.6	7.13	7.09	7.45	6.89	7.5	5.41	6.28
RABEP1	6	4.85	7.57	7.02	8.15	7.5	8.96	7.99
KATNA1	7.44	7.26	8.23	7.87	8.45	7.5	9.77	8.36
TMEM45A	10.67	9.91	11.25	9.87	6.95	7.5	10.24	8.75
MYO6	6.5	6.01	7.92	8.64	7.1	7.51	11.01	10
PPP2R3B	7.76	6.46	5.97	7	7.98	7.52	6.54	6.4
ZNF74	7.63	7.14	7.91	6.82	8.2	7.52	6.55	7.53
TMEM19	6.37	6.12	6.66	6.58	7.6	7.52	8.61	7.72
RPS6KA2	7.86	8.11	8.08	7.52	8.3	7.52	7.16	7.87
IPO11	7.17	5.56	7.66	7.47	8.51	7.52	10.07	8.98
STX3	6.04	6.24	7.27	6.24	7.65	7.52	7.6	9.33
PTPN2	6.52	7.12	7.7	9.47	7.9	7.52	11.85	9.45
LPAR6	9.43	9.17	9.27	9.71	7.59	7.52	11.33	9.92
MKLN1	8.89	6.67	8.74	9.33	8.69	7.52	12.99	11.41
F12	7.36	8.33	6.87	7.42	8.18	7.53	5.6	5.99
TRIM68	8.28	8.41	7.85	8.17	7.56	7.53	6.37	6.89
PLRG1	7.52	7.54	8.1	8.65	8.51	7.53	10.89	9.47
FAM199X	6.65	6.5	8.15	9.54	7.42	7.53	12.97	11
ABHD17B	6.5	6.99	7.79	8.69	7.92	7.54	6.13	7.89
SBF2	5.97	5.92	6.99	8.02	7.15	7.54	10.94	8.74
RPP38	7.58	8.7	7.79	8.82	8.39	7.54	9.19	8.81
RSF1	6.7	6.53	8.31	10.5	7.44	7.54	12.95	11.31
BRSK1	7.38	6.99	7.59	7.3	7.83	7.55	6.68	7.16
DDX31	6.66	7.59	7.26	7.41	8.21	7.56	8.78	7.32
CCNJL	8.27	8.68	8.34	8.48	7.58	7.56	7.24	7.37
SPATA2	7.63	7.81	7.22	7.44	7.66	7.56	6.6	8
SPRED1	7.84	6.71	8.47	9.53	7.56	7.56	10.49	9.63
MYPOP	6.65	7.23	7.38	5.67	6.99	7.57	6.15	7.02
AGA	6.09	6.86	7.64	7.08	7.42	7.57	11.05	8.37
MTERF3	6.38	6.85	7.55	8.44	7.98	7.57	12.55	9.3
SLC35B3	7.01	6.58	6.7	7.54	6.67	7.57	10.62	9.35
SFT2D3	8.33	7.53	7.53	7.57	7.56	7.58	7.02	6.27
CLCN2	7.73	8.38	8.02	7.88	7.57	7.58	7.22	7.25
TRMT61B	6.35	6.56	5.95	7.84	7.45	7.58	8.86	7.96
CNOT4	7.48	7.57	8.25	9.4	7.95	7.58	9.3	8.13
DDX28	6.54	6.51	6.39	5.87	7.71	7.59	6.02	6.5
AGPS	6.2	5.73	6.95	8.13	7.92	7.59	12.03	10.61
PARVB	6.91	7.56	7	7.72	7.43	7.6	6.45	5.82
SDSL	8.07	8.09	8.4	8.01	7.75	7.6	5.51	6.69
ZNF689	7.49	7.87	6.86	6.61	7.55	7.6	6.43	7.35
VPRBP	7.25	7.41	7.32	7.64	8.25	7.6	9.12	8.06
CHST9	5.98	7.05	7.19	7.92	7.1	7.6	10.07	9.35
MAFF	7.77	7.78	7.85	7.84	7.1	7.61	6.21	6.14
CBX7	7.48	7.5	7.84	7.34	7.87	7.61	7.66	7.31
TAMM41	7.67	6.62	6.74	6.57	6.87	7.61	8.05	7.72
HSD17B14	6.06	6.19	5.88	6.44	6.78	7.62	6.75	6.6
ANKRD13A	6.75	7.11	6.55	6.52	8.5	7.62	8.99	8.02
ABCB10	6.01	5.86	6.71	7.65	7.05	7.62	9.26	8.21
CHMP2B	5.86	6.89	6.05	8.54	7.82	7.62	9.27	8.53
WDR73	8.41	8.33	7.89	7.71	8.53	7.62	8.77	9.12
AMH	5.99	5.48	6	6.25	6.44	7.63	8.23	7.08
GJC1	6.7	6.9	7.52	7.34	7.63	7.63	8.12	8.09

CYB5R4	5.99	5.56	6.39	6.9	7.77	7.63	10.04	10.12
TIMM22	7.17	7.18	7.62	6.66	7.86	7.64	7.26	7.54
ZMYND11	7.85	6.59	7.81	8.39	7.71	7.64	10.27	8.2
PSTPIP2	10.02	9.54	9.9	10.43	8.92	7.64	11.14	10.13
HOXB2	6.64	8.64	8.93	6.29	6.54	7.65	5.84	6.69
WASF1	7.46	7.08	9.15	9.5	8.56	7.65	10.63	10.05
CRBN	6.08	7.68	6.2	8.23	7.29	7.66	10.42	8.53
LPIN3	7.3	6.76	7.2	7.13	7.25	7.66	8.82	8.64
SLC8B1	6.9	7.02	7.64	6.35	8.47	7.67	6.98	7.19
EME1	7.46	7.58	6.84	6.75	8.57	7.67	8.68	7.98
JARID2	8.04	7.94	8	9.07	7.14	7.67	7.76	8.38
UGCG	6.71	6.21	8.05	8.71	8.16	7.67	10.98	9.75
ATG10	7.38	8.16	10.18	9.82	8.12	7.68	9.83	9.71
PI4K2B	7.14	7.09	8.97	9.4	8.73	7.68	11.71	9.9
PPP1R12A	6.04	6.45	7.45	9.01	7.99	7.68	11.51	10.64
PARVA	7.98	7.8	8.09	7.91	8.33	7.69	8.38	7.89
ATP6V1A	6.5	5.51	7.46	7.65	7.73	7.69	11.43	8.78
EIF4E	6.85	7.8	7.77	8.28	8.62	7.69	10.09	9.41
BBX	7.15	6.25	8.65	10.46	7.66	7.69	12.56	11.34
NUFIP2	6.88	6.73	7.91	9.78	8.04	7.69	13.25	11.59
RELB	7.79	8.2	7.32	6.94	7.62	7.7	5.83	6.44
MCUR1	7.58	7.11	7.69	7.96	8.19	7.7	8.39	7.73
BUB1	6.17	4.91	6.55	6.4	7.49	7.7	9.08	8.72
HBS1L	7.36	6.67	7.79	8.47	8.37	7.7	10.98	10.11
BCL3	9.9	10.12	9.09	9.24	7.92	7.71	5.73	6.4
TMEM5	6.88	6.94	7.45	7.69	8.65	7.71	9.52	8.56
TPMT	6.86	6.43	7.56	8.37	9.16	7.71	10.6	9.78
LEFTY1	8.02	8.54	8.27	7.41	6.76	7.72	5.14	8
NUDCD1	6.61	7.05	6.93	8.08	7.92	7.72	11.35	9.57
USP15	5.85	6	6.64	7.19	7.3	7.72	11.12	9.6
CCDC28A	6.89	6.24	7.24	8.42	6.93	7.72	10.17	9.87
MEF2A	6.95	6.14	7.94	9.32	8.06	7.72	11.86	10.18
QSER1	6.41	6.26	7.66	9.22	7.98	7.72	12.5	11.66
DHODH	7.36	7.37	7.07	6.63	7.93	7.73	7.39	7.37
SLC48A1	7.34	8.29	7.69	7.51	8.41	7.73	7.98	7.44
NEMP1	6.58	5.84	6.82	7.63	7.84	7.73	10.73	9.31
RNF2	8.28	7.75	8.37	8.96	7.87	7.73	11.09	9.76
ARID5B	7.98	7.31	8.3	9.35	8.14	7.73	9.72	9.81
ZNF791	6.84	5.86	7.94	9.24	7.47	7.73	12.01	10.35
FNDC3A	6.13	6.06	7.65	9.39	7.67	7.73	13.28	11.46
ASRGL1	8.86	8.41	8.73	8.49	9.32	7.74	6.87	6.48
FAM222B	8.15	9.28	8.53	8.34	7.79	7.74	8.15	8.42
CDK14	6.76	6.27	7.33	7.76	7.09	7.74	10.39	8.46
EBAG9	7.77	6.21	6.77	8.52	8.66	7.74	11.07	10.19
PEX6	9.13	8.94	8.46	8.25	7.89	7.75	6.26	7.04
MAP3K4	8.34	7.77	8.62	9.05	7.06	7.75	8.99	8.23
TTPAL	6.85	6.59	7.18	7.53	7.41	7.75	8.83	8.56
CCNYL1	7.53	6.87	8.02	8.51	8.56	7.75	10.68	10.42
A1BG	7.5	8.54	7.57	6.08	7.66	7.76	5.78	6.57
TMEM38B	7.16	6.51	7.13	6.13	7.13	7.76	9.45	8.18
GOLT1B	6.67	6.71	7.28	8.63	8.27	7.76	12.44	10
RANBP6	7.15	6.61	8.67	9.32	7.98	7.76	11.94	10.67
STARD4	6.52	6.77	8.44	10.07	8.06	7.76	15.86	13.1
VPS33B	6.8	7.54	6.82	6.35	6.79	7.77	7.23	7.18
DHX34	7.36	7.34	7.11	7.43	7.7	7.77	7.05	7.52
NOP9	6.19	6.65	6.38	6.71	7.28	7.77	8.88	8.21
PGM2	6.98	5.39	6.63	7.39	8.08	7.77	10.27	8.6
SMURF2	8.12	6.7	8.52	8.93	7.53	7.77	10.7	9.36
MCC	7.13	6.18	7.67	8.6	7.95	7.77	10.25	9.61
ANO6	7.6	7.01	8.35	8.91	8.01	7.77	10.51	10.6
ZNF213	8.04	7.84	7.87	7.11	7.66	7.78	6.78	7.26
FANCD2	7.05	6.72	7.72	7.95	7.58	7.78	10.29	7.5
KDM2B	8.18	8.17	7.89	7.98	7.76	7.78	7.51	8.09
MTFMT	6.49	6.7	7.2	7.28	6.94	7.78	8.19	8.65
NUP54	6.65	7.21	8.03	8.51	8.07	7.78	11.67	9.25
FAM149B1	7.27	7.65	7.31	8.22	8.45	7.78	10.26	9.52
VMA21	6.39	5.83	7.12	8.83	8.12	7.78	12.35	11.37
DENND6A	7.69	7.11	8.2	8.36	9.26	7.79	10.77	9.36
LGALS9	12.17	11.01	10.21	9.35	8.42	7.8	7.48	7.14
PARG	5.61	6.29	7.46	7.94	6.82	7.8	9.21	8.66
GPALPP1	7.1	7.49	7.25	8.27	7.79	7.8	10.94	9.61
TIMM8A	7.38	6.95	7.46	6.85	8.75	7.8	8.38	10.04
ZNF747	8.21	8.15	7.95	8.02	7.69	7.81	6.48	7.49
ZDHHC1	8.57	8.83	9	8.07	7.78	7.82	5.94	6.23
ATRIP	6.35	6.26	7.07	6.03	7.7	7.82	7.18	6.57
SLCO4A1	8.88	7.77	8.26	7.95	8.09	7.82	7.15	7.37
HOXA2	7.97	8.42	8.97	7.73	7.36	7.82	9	8.38
CCDC9	8.64	7.74	7.91	7.79	7.35	7.83	6.19	6.52
PHLDB3	6.39	6.41	5.83	5.86	7.11	7.83	5.31	6.64
SDE2	7.75	7.38	8.03	9.44	7.54	7.83	9.71	9.21
KPNA4	6.66	6.3	7.76	8.58	8.42	7.83	11.11	10.08
ABHD10	7.61	6.96	8.41	9.07	9.73	7.83	10.4	10.25
BACE1	7.34	7.68	8.22	7.41	8	7.84	7.93	7.74
C14orf79	7.35	6.79	8.05	7.51	7.79	7.85	6.77	7.01
GAREM2	7.32	6.99	7.13	6.62	7.94	7.85	6.57	7.35
WAPL	7.28	6.42	7.45	8.77	8.29	7.85	11.4	10.04
FAM64A	6.3	6.72	7.2	5.74	7.19	7.86	6.52	6.2

KLHDC8B	8.54	8.49	7.47	6.83	8.05	7.86	5.84	6.8
NDUFAF6	6.25	6.4	6.64	7.21	7.52	7.86	8.73	7.23
DNAJC18	7.25	7.22	6.97	6.87	8.3	7.86	8.32	7.98
SAAL1	7.58	7.81	8.04	8.93	7.94	7.86	9.3	8.21
TCTN2	10.93	10.11	9.53	10.66	8.72	7.86	8.45	8.52
TP53BP2	6.97	6.45	8.59	8.48	8.64	7.86	10.07	9.01
NEK2	6.23	6.19	6.33	7.24	8.31	7.86	10.89	9.42
LRRC37A	5.75	6.37	7.51	7.5	6.87	7.86	10.8	9.71
CPNE8	7.61	7.82	8.57	9.74	7.95	7.86	12.59	10.95
FZD2	9.75	9.95	9.54	8.4	7.22	7.87	6.21	6.95
ENGASE	7.64	6.91	7.27	8.81	7.76	7.87	7.81	7.45
ZNF436	8.2	8.35	8.9	8.21	7.58	7.87	8.62	8.22
ZNF839	7.88	7.86	7.23	7.76	8	7.87	8.21	8.77
IDE	6.38	6.53	7.84	8.53	8.69	7.87	12.72	11.03
RSPRY1	7.18	6.99	8.38	9.73	8.34	7.87	12	11.08
TRMT5	7.85	6.94	8.5	8.82	8.64	7.87	11.64	11.34
SLC25A36	7.05	6.49	7.99	9.7	7.57	7.87	14.54	13.15
C16orf95	7.44	8	6.43	5.74	6.23	7.88	5.19	4.59
GPR157	7.4	7.35	6.36	6.21	8.09	7.88	6.18	6.13
SLC25A14	5.73	6.04	5.25	6.24	7.18	7.88	8.5	7.49
FAM73B	8.19	7.88	7.27	7.83	8.34	7.88	7.41	7.76
HK2	11.8	10.89	11.43	11.94	8.33	7.88	9.14	8.43
NKRF	7.17	6.49	7.36	8.51	8.61	7.88	9.75	9.28
WDR60	7.11	6.7	8.58	9.79	7.62	7.88	10.4	10.07
IFIT3	11.96	11.03	13.14	15.31	8.03	7.88	12.06	10.52
SUCO	8.47	8.31	9.52	12.34	8.06	7.88	13.47	11.55
DIS3L	7.09	7.32	6.76	7.83	7.88	7.89	9.03	8.96
TXNL4B	6.84	6.23	6.35	6.66	7.65	7.9	8.08	7.43
FAM84A	6.49	6.48	6.38	6.56	7.24	7.9	6.73	8.21
COA5	6.91	6.48	7.37	7.1	8.87	7.9	10.23	8.59
NPIPA5	4.95	6.26	7.38	6.32	7.26	7.9	11.89	8.9
LOC101929959	6.08	5.6	7.14	6.87	7.86	7.9	8.29	9.12
TRIT1	8.28	6.59	7.96	9.09	9.48	7.9	11.06	10.04
GTF2IRD2	5.24	6.74	5.03	7.4	8.54	7.91	5.54	5.79
TTC38	8.74	8.16	8.32	7.5	7.88	7.91	7.45	7.45
S100PBP	8.46	7.27	7.69	7.51	7.64	7.91	9.94	8.33
SPRYD4	7.34	7.79	7.35	7.87	8.18	7.91	8.07	8.6
EZH1	7.53	7.74	7.96	9.02	8.46	7.91	10.11	9.66
KIAA1324L	7.76	7.44	8.97	9.46	8.26	7.91	11.22	10.34
FBXO27	8.91	8.72	8.32	8.7	7.7	7.92	7.59	6.71
FANCF	7.63	7.05	7.93	7.55	7.64	7.92	7.78	7.94
COL4A3BP	7.1	6.4	7.75	9.79	7.82	7.92	10.73	10.03
PRAP1	5.4	6.57	5.93	4.9	7.75	7.93	6.48	5.88
BPHL	8.06	7.88	8.32	7.65	8.85	7.93	7.19	8.36
PPP2R3C	6.48	5.66	6.9	7.17	7.86	7.93	10.19	9.4
IDUA	6.76	5.91	6.24	6.24	5.82	7.94	6.07	7.12
GSPT2	7.73	8.64	8.53	8.33	7.49	7.94	8.78	8.69
ZCCHC11	6.45	6.65	8.21	9.65	7.88	7.94	13.95	11.81
HSD11B1L	7.96	8.2	7.03	8.12	7.45	7.95	6.93	6.78
ASB1	6.73	6.49	7	6.9	7.97	7.95	8.23	7.4
NDUFAF5	6.98	7.04	7.97	8.87	10.34	7.95	9.41	8.7
ISOC1	7.89	6.77	7.85	8.67	9.24	7.95	9.52	8.94
TANC1	8.09	7.95	8.46	8.67	8.15	7.95	9.45	9.17
SPATA24	9.3	8.22	7.25	7.77	7.42	7.95	6.89	9.28
TSTD2	8.12	7.14	7.98	8.98	8.58	7.95	10.71	9.43
CTDSPL2	8.41	8.03	9.55	10.17	9.18	7.95	12.4	11.7
B3GNT2	8.47	7.78	8.24	9.33	8.85	7.96	10.29	9.35
RFK	6.79	5.29	6.85	7.73	8.69	7.96	12.23	9.58
BTBD19	5.46	5.5	5.96	7.6	6.74	7.97	10.05	8.78
ATAD2	8.56	7.02	9.64	11.29	8.82	7.97	14.06	10.78
ISY1-RAB43	8.27	7.72	7.94	7.22	9.57	7.98	8.24	8.15
RECK	7.73	8.02	7.83	8.05	7.9	7.98	9.24	9.76
MED28	8.09	8.14	8.67	7.99	9.17	7.98	9.45	9.78
TAF2	6.4	5.72	7.24	8.59	8.36	7.98	12.13	10.33
SERGEF	8.05	9.31	8.25	8.3	7.94	7.99	6.99	7.85
FUT8	6.03	5.6	7.57	7.57	8.53	7.99	10.08	9.68
PGRMC2	8.1	7.73	8.39	9.56	9.13	7.99	10.27	9.76
SGCB	8.61	7.52	9.19	10.66	9.51	7.99	13.36	10.72
CBX8	10.84	10.35	9.12	8.64	7.89	8	5.97	6.99
PARP14	10.48	9.38	12.08	15.29	7.59	8	13.78	11.03
TTI2	8.44	9.11	8.14	7.6	8.65	8.01	7.57	7.23
YARS2	7.77	6.69	7.64	7.37	9.09	8.01	9.46	8.45
KMT2A	8.01	7.8	8.5	8.95	8.07	8.01	10.21	9.9
NLRX1	8.42	8.53	8.54	7.31	7.32	8.02	6.9	7.53
MED20	8.45	8.71	8.68	7.86	7.22	8.02	8.28	8.19
APOBEC3F	9.09	10.71	9.36	10.08	8.7	8.02	9.47	8.61
FAN1	7.75	7.69	8.91	9.43	8.71	8.02	11.06	10.34
TCF7L1	8.36	10.08	8.8	8.73	9.12	8.03	6.79	8.41
SLC6A6	7.53	7.4	7.8	7.41	8.15	8.03	8.58	9.3
ZSWIM7	6.08	6.02	6.23	6.44	7.43	8.04	8.21	7.12
RAP2B	6.93	6.37	7.5	7.07	7.89	8.04	8.28	7.83
CHRAC1	7.87	7.68	8.06	8.29	9.03	8.04	10.22	9.17
ZNF106	7.68	6.77	7.73	8.43	8.61	8.04	10.15	9.21

ZDHHC2	7.69	7.71	8.24	8.31	7.92	8.04	9.76	10.35
AIFM2	7.9	7.79	7.58	7.5	7.58	8.05	7.54	7.14
EHF	6.27	6.39	7.06	8.14	8.68	8.05	11.98	10.36
ORC2	7.64	7.15	8.86	9.6	9.34	8.05	11.22	10.51
CHURC1	6.82	5.76	8.35	10.51	8.54	8.05	14.91	11.84
LRP4	8.08	8.25	7.92	8.96	7.94	8.06	7.85	8.34
ABHD2	8.45	7.92	8.74	8.81	8.53	8.06	9.83	8.96
SIAH1	8.46	8.52	8.53	9.91	8.45	8.06	10.78	10.03
DCAF16	7.53	6.5	8.37	9.32	9.5	8.06	12.09	11.84
NTNG1	7.51	7.64	7.8	8.32	8.02	8.07	9.31	9.74
TRIM22	11.25	10.21	11.36	13.27	8.53	8.07	11.33	9.85
NDUFA5	8.11	8.61	7.65	8.55	9.06	8.07	12.02	10.92
NSUN4	7.15	7.33	7.33	7.09	6.99	8.08	7.37	7.24
FAM63A	8.2	9.71	9.41	7.98	7.97	8.08	7.22	7.46
PHF20L1	6.85	7.12	8.47	10.18	7.99	8.08	14.74	11.42
NDST2	7.47	7.69	7.88	8.61	7.66	8.09	7.11	7.6
TMEM70	7.07	5.9	8.32	7.11	8.42	8.09	9.35	8.24
CAMTA2	8.03	7.99	7.8	7.7	7.87	8.09	7.19	8.33
RIPK2	6.14	6.33	6.9	7.06	8.32	8.09	10.63	9.63
SLC25A46	7.66	7.4	8.62	8.91	8.14	8.09	12.25	10.88
ZNF24	9.36	7.57	10.65	12.47	9.99	8.09	14.77	11.24
TMEM106B	7.73	7.62	8.09	9.66	8.6	8.1	13.92	12.02
NBL1	5.88	4.53	9.29	6.09	6.59	8.11	2.1	4.24
CTSF	7.31	8.99	8.95	7.52	7.67	8.11	5.85	6.93
MALT1	6.83	6.6	8.54	7.69	6.32	8.11	10.15	7.65
ZBTB12	8.09	8.45	8.99	7.66	7.35	8.12	6.93	7.42
EGLN3	12.69	11.88	12.67	11.72	7.72	8.12	9.58	7.43
LOC107987372	7.75	6.84	7.95	6.98	5.64	8.12	6.87	7.5
RMND1	7.3	7.1	6.5	6.71	8.18	8.12	12.01	9.6
PPWD1	7.2	6.82	7.72	8.85	6.84	8.12	13.1	10.25
LTV1	7.56	6.69	8.83	9.02	9.11	8.12	12.44	12.95
DDX51	7.54	7.35	8.53	7.68	10.31	8.13	7.14	8.06
C16orf70	7.05	7.8	8	8.77	7.63	8.13	8.28	8.92
BTAF1	6.9	5.88	7.31	8.75	6.71	8.13	13.99	11.33
ATP6V1C1	6.92	6.36	7.39	8.97	8.43	8.13	13.86	11.48
DOT1L	7.9	7.91	8.16	7.99	7.88	8.14	7.22	7.99
ZDHHC13	7.84	7.68	7.82	8.55	8.8	8.14	11.46	9.64
SUOX	8.84	9.5	8.28	7.82	8.32	8.15	7.38	8.41
PHTF2	6.77	5.6	8.1	9.33	8.63	8.16	13.68	10.97
IRS2	7.95	7.49	7.62	8.05	7.41	8.17	6.59	6.89
C19orf47	7.77	7.7	7.72	6.91	8.34	8.17	7.23	7.34
ADSSL1	10.77	11.08	9.85	10.06	7.11	8.17	6.04	7.53
TAPBPL	9.92	9.22	9.42	9.11	7.99	8.17	7.02	7.76
NR1H3	7.13	7.8	7.34	6.91	9.15	8.17	8.09	8.91
SPPL2A	6.62	6.88	7.18	8.12	8.38	8.17	11.36	10.26
ITGA4	8.93	8.41	10.13	12.81	7.84	8.17	12.52	11.28
MNX1	8.35	8.31	8.05	8.2	7.07	8.18	5.36	6.06
FOXQ1	6.26	5.92	6.06	5.92	6.59	8.18	6.86	6.3
ASNSD1	5.63	5.66	6.87	7.49	7.82	8.18	11.54	9.42
TMEM245	7.61	7.31	8.42	8.92	8.35	8.18	11.16	9.7
SH3BP2	8.91	8.86	9.65	10.3	8.03	8.18	9.45	9.86
CLUAP1	7.8	7.76	8.95	8.7	8.38	8.18	10.18	10.21
GNPTAB	7.43	7.2	8.02	9.52	8.98	8.18	12.16	10.92
LRRC40	7.33	8.12	8	9.04	8.73	8.18	13.42	11.81
CHURC1-FNTB	7.16	8.19	7.86	6.72	5.29	8.19	5.49	6.17
ANKRD16	8.08	6.45	7.92	7.36	7.08	8.19	6.04	6.31
FGD1	9.3	9.52	8.55	8.69	7.74	8.19	6.89	7.59
N4BP1	8.57	8.4	8.25	8.67	7.39	8.19	8.85	8.45
AREL1	7.33	7.25	7.5	7.53	8.41	8.19	9.23	8.9
MAN1A1	7.8	7.84	8.68	10.26	8.01	8.19	9.36	8.94
TMEM50B	8.76	8.13	9.1	9.56	9.17	8.19	11.27	9.92
MLXIP	6.94	7.68	7.3	7.66	7.69	8.2	7.83	8.27
CREBL2	7.58	7.14	7.56	8.35	7.72	8.2	9.9	9.08
TMEM135	6.31	7.18	7.61	8.59	8.39	8.2	11.5	10.25
PGAP3	10.06	9.65	9.02	9.27	8.01	8.21	7.03	6.97
CLDN15	6.84	6.29	6.18	8.01	7.79	8.21	9.03	7.83
AEBP2	7.41	7.19	7.78	9.21	8.29	8.21	12.56	10.29
YJEFN3	5.87	5.69	5.4	7.45	7.66	8.21	14.8	10.76
B4GALNT1	7.34	8.64	7.96	7.98	7.46	8.22	5.95	6.75
PDK3	8.41	7.34	8.35	8.08	7.85	8.22	7.69	7.89
TMX4	8.07	8.04	9.08	9.26	8.77	8.22	9.97	9.36
DMTF1	8.23	7.39	9.26	11.04	8.78	8.22	16.95	13.93
USP31	7.41	7.02	8.11	8.22	7.63	8.23	8.22	7.22
ZNF574	9.15	9.06	8.72	8.68	7.75	8.23	6.6	7.29
CAAP1	8.22	7.97	8.58	9.33	8.3	8.23	10.48	9.58
ZNF608	8.59	8.16	9.51	11.28	8.95	8.23	11.47	10.75
IMPACT	7.14	6.47	8.09	9.35	9.68	8.23	13.35	11.4
YEATS4	7.28	6.53	6.89	7.38	8.31	8.24	10.26	8.79
SEC24D	9.34	8.68	10.22	9.5	8.25	8.24	9.84	9.33
SHCBP1	7.8	7.12	8.44	8.32	9.42	8.24	9.93	9.86
GIT2	9.19	8.03	8.56	9.05	9.55	8.24	10.13	9.91
CIR1	7.48	7.82	8.5	10.12	9.71	8.24	13.19	11.78
BIRC6	7.42	6.61	9.21	10.56	8.55	8.24	13.42	12.07
SERTAD1	9.15	8.42	7.93	7.69	8.81	8.25	7.78	7

ACOX3	7.76	7.55	7.51	6.93	8.21	8.25	7.21	8.14
GPC4	10.58	10.15	9.66	9.48	7.98	8.25	8.28	9.51
ZNF462	8.15	7.31	8.84	10.12	7.37	8.25	9.73	9.7
PLD1	8.13	6.69	8.48	8.54	7.89	8.25	10.61	9.7
PPM1B	7.33	7.82	9.51	11.14	8.71	8.25	11.68	10.09
B3GALNT1	7.64	7.3	8.72	10.16	9.18	8.25	12.5	10.48
BOLA1	9.75	9.07	8.87	8.49	8.93	8.26	7.39	6.95
LARS2	8.2	8.36	8.25	7.71	7.51	8.26	8.02	8.42
ADIRF	10.94	10.02	10.55	10.88	9.64	8.27	7.03	7.21
MPHOSPH6	7.96	8.38	8.51	8.09	7.78	8.27	11.05	8.12
NLRP1	7.38	6.88	7.25	7.66	9.03	8.27	9.43	9.19
EXOC6B	7.44	7.96	8.42	8.51	9.32	8.27	11.05	10.01
ZNF322	9.76	8.51	10.49	11.85	7.54	8.27	12.42	11.64
ZNF740	7.23	8.72	8.21	8.41	8.17	8.28	8.33	8.3
SMU1	8	7.88	8.23	8.53	7.9	8.28	9.68	9.05
TMEM87B	7.13	6.62	7.78	9.21	8.34	8.28	11.14	9.94
CCDC88A	7.51	7.29	7.7	9.69	7.71	8.28	14.2	12.64
FAM120B	8.97	8.06	10.34	9.85	7.98	8.29	9.38	9.49
TBPL1	8.67	9.37	9.06	9.66	9.74	8.29	12.31	9.72
ZNF281	8.63	7.48	9.53	11.15	8.98	8.29	11.96	11.24
TBX1	9.59	8.45	9.61	8.99	7.68	8.3	6.98	7.07
ZBTB9	8.51	8.57	8.74	7.83	8.21	8.3	7.45	7.81
AGFG1	8.03	8.19	8.22	9.33	8.84	8.3	11.04	9.79
MUT	7.62	6.65	8.65	9.66	9.85	8.3	13.09	10.17
PHIP	6.68	7.01	7.96	9.72	7.66	8.3	12.75	11.42
ZC3H12A	7.41	7.46	7.29	7.75	9.04	8.31	7.75	7.04
SNAPC4	6.81	6.79	6.19	6.68	7.2	8.31	7.42	7.36
ACTR5	8.2	8.55	8.49	8.73	8.89	8.31	9.06	8.64
MTMR11	8.01	8.18	8.78	9.05	7.71	8.31	8.99	8.73
TOP3B	7.95	7.1	8.13	7.55	7.57	8.32	8.13	7.02
TMEM79	7.75	7.29	8.17	8.28	8.31	8.32	8.17	8.23
PDZD8	6.68	6.52	7.34	8.19	7.73	8.32	9.91	8.62
COQ6	8.06	8.42	7.82	8.2	9.29	8.32	8.8	8.66
TMEM185A	8.82	9.35	9.29	9.95	9.25	8.32	9	9.64
BAG5	7.36	6.83	7.84	8.58	8.39	8.32	10.06	9.68
RFX1	8.67	8.58	7.91	7.42	7.49	8.33	5.5	6.31
MPC1	8.41	6.64	7.55	7.16	7.95	8.33	9.4	7.41
ABCB9	7.91	7.64	8.16	7.59	8.28	8.33	6.54	7.57
FXN	7.53	7.53	8.21	6.53	8.42	8.33	7.12	7.6
C1orf50	6.88	7.75	9.44	7.48	8.86	8.33	9.2	8.7
SORL1	7.46	6.99	8.15	8.78	8.66	8.33	9.58	9.83
TSPYL4	7.83	8.16	8.59	9.01	8.44	8.34	9.79	9.09
RND3	6.68	6.64	7.11	8.98	9.08	8.34	13.49	10.36
NDFIP2	7.62	7.73	8.22	9.12	9.12	8.34	11.93	10.38
MED11	7.46	7.51	6.09	7.66	7.37	8.35	7.18	7.74
NCALD	8.61	8.08	8.51	7.86	7.92	8.35	8.01	8.1
TMEM41A	8.18	8.92	9.47	7.73	9	8.35	8.84	8.75
MYCBP	7.47	7.05	8.01	8.73	8.53	8.35	11.37	9.15
PIK3R4	7.92	7.41	8.57	8.68	8.75	8.35	11.52	10.77
BLOC1S6	6.64	5.35	7.81	8.96	8	8.35	14.7	12.04
TAB3	6.22	6.31	7.93	9.57	8.21	8.35	13.55	12.31
KTI12	8.63	8.82	7.38	7.39	8.47	8.36	7.38	7.06
TECPR1	7.76	7.65	7.41	7.36	7.81	8.36	7.26	7.36
PDE4A	7.13	7.24	6.96	7.24	7.8	8.36	7.05	7.86
LIN37	7.95	8.93	8.04	7.4	9.89	8.36	7.17	8.27
HYAL1	7.3	7.33	8.02	7.46	9.21	8.36	7.75	8.68
N4BP2L2	7.83	7.43	8.97	10.65	8.96	8.36	16.37	12.33
TNFRSF14	6.66	7.47	6.61	7.34	7.9	8.37	8.33	7.48
TSNARE1	8.05	8.28	7.55	6.8	8.25	8.37	8.25	8.6
CRNDE	7.49	7.43	8.28	8.83	7.81	8.37	8.08	9.42
DDX20	8.2	6.7	8.07	8.41	8.55	8.37	10.94	9.61
SPG20	8.17	7.44	8.94	9.64	8.41	8.37	12.9	11.39
RABL2A	7.42	7.33	6.6	7.79	7.11	8.38	7.45	7.62
AKAP10	7.23	6.4	8.66	9.42	8.27	8.38	11.58	9.29
TMTC2	9.13	9.31	10.04	10.74	8.74	8.38	10.69	9.47
SKP2	9.17	8.12	9.07	9.58	9.21	8.38	12.02	10.06
KIAA1143	7.08	7.45	7.88	8.79	9.72	8.38	11.87	11.02
RSRC1	6.49	7.13	8.15	8.95	9.07	8.38	12.69	12.99
PLA2G6	7.8	7.34	7.56	7.25	7.48	8.39	8.12	7.63
PCDHGB1	7.47	8.21	7.82	7.98	6.79	8.39	7.36	8.53
RTN2	6.44	6.24	5.93	5.89	7.72	8.4	5.99	6.85
SPCS3	7.28	7.22	9.08	10.62	8.89	8.4	12.77	11.09
TAOK1	7.24	6.43	8.83	10.81	8.59	8.4	14.72	13.04
TMEM164	8.97	9.58	9.22	9.01	9.52	8.41	9.49	9.25
NEMF	7.31	7.17	8.38	9.35	7.86	8.41	13.25	11.94
RPL17-C18orf32	7.05	7.7	9.85	8.41	10.73	8.41	13.19	14.44
CLDN4	6.96	8.05	6.76	8.04	7.57	8.42	7.14	7.01
TTC5	9.09	8.79	8.92	8.63	8.03	8.42	7.55	7.45
DHX57	8.89	8.32	9.23	9.4	9.59	8.42	12.3	9.97
ARFGEF1	7.13	6.44	8.35	9.84	8.74	8.42	14.97	12.81
NIPA2	7.32	7.05	6.92	8.29	8.48	8.43	9.99	9.85
ZNF7	7.62	7.73	8.68	9.28	8.55	8.43	10.64	9.87
FAM229A	6.86	6.23	4.89	6.29	6.84	8.44	6.95	7.21
PRIM2	8.4	7.75	8.4	7.8	8.07	8.44	9.52	8.57

AGO2	7.5	8.16	7.82	8.51	8.25	8.44	9.59	9.43
NGLY1	7.97	6.97	7.9	8.11	8.41	8.45	9.63	10.77
SLK	7.54	6.88	7.94	10.15	8.47	8.45	13.5	12.5
HIST1H4C	4.21	3.95	3.81	4.45	7.61	8.46	7.54	7.43
SLC38A7	7.05	7.38	6.89	6.96	8.3	8.46	7.52	7.93
MTMR1	8.24	7.89	7.76	8.63	8.03	8.46	8.63	8.09
PEAR1	7.2	7.55	7.44	7.46	8.29	8.46	8.74	8.67
BPGM	7.06	7.32	7.24	7.25	9.27	8.46	11.64	9.55
CDR2L	7.79	8.69	7.74	7.65	7	8.47	6.29	7.29
NUDT19	8.46	6.56	6.82	7.3	8.3	8.47	7.77	8.01
DHX33	6.28	6.63	6.98	7.17	8.87	8.47	9.82	8.3
CDK18	10.29	9.79	10.23	10.06	7.77	8.49	8.2	8.38
FAM212B	7.37	7.54	6.89	6.83	9.11	8.49	8.86	8.85
ZMAT3	4.91	4.76	5.6	7.02	8.61	8.49	12.86	11.56
DPY19L4	8.58	8.31	9.3	11.08	9	8.49	14.25	12.91
LYAR	8.66	8.62	9.53	10.07	11.67	8.49	14.78	13.21
FAM86C1	7.91	8.61	8.5	7.5	8.45	8.5	7.56	8.24
ZBED4	8.59	8.16	8.86	8.75	8.85	8.5	9.34	8.82
FOXO3	9.34	8.31	9.61	10.17	7.91	8.5	9.32	9.68
C2orf74	6.69	7.77	7.1	8.79	7.02	8.5	11.93	9.74
ANKRD17	8.44	7.45	9.56	9.79	9.02	8.5	11.17	10.5
FMNL2	7.66	7.26	8.34	9.48	8.44	8.5	12.43	11.15
ARMCX3	8.34	8.31	9.97	11.48	9.74	8.51	12.83	11.74
MAP3K6	9.35	10.01	9.91	8.72	7.43	8.52	6.43	7.32
MKL1	9.87	9.25	9.06	8.57	9.03	8.52	7.49	8.3
AZI2	8.59	9.29	11.01	10.2	8.98	8.52	12.71	11.77
NFATC3	8.67	8.22	9.08	9.38	8.38	8.53	10.3	9.24
ZNF639	7.91	7.43	8.99	9.15	9.35	8.53	13.48	12.05
LOC102724751	7.17	8.1	7.58	8.36	8.09	8.54	6.04	6.45
MOAP1	7.52	7.28	7.86	8.57	8.47	8.54	9.65	8.76
SH3RF2	8.42	8.47	8.53	9.17	9.35	8.54	10.02	8.94
TRAPPC10	8.22	7.44	8.79	9.24	9.16	8.54	10.22	9.94
ZSWIM4	8.4	9.14	7.98	7.49	8.2	8.55	7.08	7.8
UBR7	9.81	9.68	9.8	10.37	9.96	8.55	11.44	9.12
BPTF	7.64	6.97	9.22	11.34	8.25	8.55	13.64	12.5
WDR24	9.03	9.59	8.52	8.54	7.57	8.56	6.69	7.21
GINS4	8.23	7.47	7.88	7.68	9.53	8.56	9.34	8.25
TMEM38A	8.54	8.66	8.45	7.93	8.17	8.56	7.95	8.67
ZNF251	7.87	7.13	7.76	8.66	8.12	8.56	10.99	9.07
CLIC3	12.32	14.5	11.53	10.51	6.17	8.58	4.21	6.18
ASB6	8.52	8.81	8.28	7.61	8.96	8.58	6.87	7.57
PHF8	8.92	8.85	9.31	9.16	8.62	8.58	8.19	8.35
GTF2H2	6.57	6.46	5.85	8.13	6.44	8.58	12.41	8.48
PFKFB4	14.64	12.22	13.72	12.15	9.16	8.58	11.35	9.11
RNF128	9.27	8.74	8.89	10.9	8.2	8.58	10.47	9.57
THAP12	8.69	7.87	9.07	9.9	8.51	8.58	10.84	10.38
B3GALNT2	6.9	6.37	7.54	9.36	8.17	8.58	11.31	10.44
ROCK1	7.37	7.27	7.58	9.66	7.95	8.58	13.53	11.79
C11orf80	8.06	8.68	8.12	7.81	7.9	8.59	9.51	7.39
TIMELESS	10.01	9.34	9.66	8.64	9.32	8.59	9.39	8.52
CCDC88C	9.17	9.05	8.77	9.01	9.13	8.59	8.1	8.82
ERBIN	7.9	7.9	8.81	10.55	7.75	8.59	14.2	12.88
RAD9A	9.92	8.69	8.75	9.5	9.02	8.6	7.57	8.85
MESDC2	10.11	9.57	9.88	10.2	9.53	8.6	9.61	10.06
ALAD	9.74	9.2	9.27	9.5	9.29	8.6	9.66	10.11
LPAR2	8.82	8.1	7.9	9.08	9.6	8.6	9.14	10.4
RNMT	7.66	6.68	8.67	10.84	9.2	8.6	12.22	12.15
SHTN1	7.81	7.47	9.46	11.06	8.8	8.6	13.93	12.77
ZNF526	9.74	9.92	9.45	9.53	8.49	8.61	7.46	7.66
RRP15	7.1	7.31	8.14	8.64	8.74	8.61	10.62	11.05
PDP1	7.28	7.27	7.98	9.01	9.38	8.61	12.27	11.35
TMEM154	4.32	4.74	5.73	5.97	9.65	8.61	13.81	11.45
BNIP1	7.87	8.22	7.83	7.34	7.94	8.62	8.74	7.72
GMPR	12.25	12.53	11.52	10.91	9.06	8.62	7.38	8.68
ZBTB22	7.95	9.68	8.39	8.65	8.58	8.62	7.13	8.79
ARL3	8.73	8.59	9.23	9.54	9.29	8.62	9.64	9.39
SUZ12	8.47	8.39	8.9	10.38	8.82	8.62	15.28	12.72
TGFBRAP1	7.19	6.96	7.23	7.33	9.27	8.63	9.03	8.98
TMEM128	6.96	7.21	9.07	8.7	9.42	8.63	11.78	10.16
CDK13	8.84	7.97	10.23	9.77	8.92	8.63	10.08	10.2
INO80	7.79	7.5	7.71	8.52	8.83	8.64	9.68	8.67
ZC3H13	8.45	7.94	9.52	11.94	9.31	8.64	13.75	12.69
C16orf91	7.83	8.29	7.94	8.06	9.19	8.65	7.3	7.02
TICAM1	8.61	10.16	9.33	8.71	8.63	8.65	6.26	8.02
URGCP	8.7	9.65	9.15	9.14	8.94	8.65	7.54	8.45
ZNF668	7.74	7.82	8.13	8.02	8.57	8.66	7.65	8.2
TEAD4	8.03	8.21	8.01	6.89	7.36	8.66	6.48	8.22
MAP3K5	8.05	7.33	8.89	9.38	9.17	8.66	11.17	9.97
LHPP	8.32	9.43	8	8.56	8.96	8.67	7.58	7.86
NRN1	9.82	9.53	8.51	9.89	8.73	8.67	9.26	8.8
EVI5L	8.93	9.87	9.44	7.7	8.85	8.68	6.58	7.2
TANK	7.34	7.72	7.77	10.04	7.76	8.68	12.9	10.28
FAM156B	7.69	5.73	8.43	10.99	8.81	8.68	13.58	11.81
CTU1	7.71	6.79	6.6	6.13	7.35	8.69	5.15	5.96

KIF3C	7.48	8.58	7.96	7.57	7.73	8.69	6.72	8.04
ATXN1L	8.5	8.67	9.13	9.16	9.16	8.69	9.12	8.93
SIAE	8.19	9.39	8.84	9.83	9.88	8.69	11.11	10.04
WDR92	7.79	6.92	8.44	9.35	8.37	8.7	10.63	8.84
RAB4A	8.27	8.23	8.3	8.06	10.62	8.7	9.67	9.51
HSPA14	8.46	7.29	8.78	10.02	9.13	8.7	11.97	10.13
TCP11L1	8.18	7.38	7.3	8.66	8.39	8.71	9.42	9.23
EFR3A	7.41	6.76	9.14	9.61	9.29	8.72	12.85	11.03
TNRC6A	8.13	7.29	9.18	11.17	8.72	8.72	12.94	11.69
LNPEP	8.39	8.06	9.09	11.17	8.63	8.72	15.16	12.88
CETN3	7.08	8.83	9.59	9.31	7.99	8.73	12.89	10.03
MNAT1	9.24	9.9	9.8	10.16	8.43	8.73	12.46	10.2
RAP2C	7.38	7.31	9.34	9.38	9.55	8.73	12.45	10.85
GOLGA4	6.13	6.06	7.49	8.5	8.09	8.73	14.71	12.71
SMG6	7.64	8.04	8.11	7.72	7.38	8.74	7.26	7.89
ADCY7	8.43	7.54	8.62	8.87	7.99	8.74	8.8	8.15
TBC1D2B	7.87	8.43	8.13	8.61	8.88	8.74	8.64	8.29
EPOR	8.33	7.92	8.96	7.55	8.74	8.74	7.39	9.26
RPS6KB1	6.49	7.23	8.93	9.91	8.57	8.74	15.21	12.42
WRAP53	8.7	8.89	7.79	7.68	8.42	8.75	7.34	7.41
YIPF6	7.31	7.75	8.66	9.56	8.34	8.75	11.6	11.06
CCDC71L	9.31	8.88	8.6	8.06	8.18	8.76	7.28	7.3
ARHGEF4	7.21	5.61	6.52	8.4	8.19	8.76	7.12	8
VDR	8.82	8.85	9.44	8.2	8.04	8.76	7.66	8.1
USP20	9.37	9.72	8.66	8.48	8.69	8.76	7.8	8.31
TMEM237	7.98	7.58	9.06	9.16	8.86	8.76	10.66	9.55
ANOS1	10.56	9.64	11.5	12.63	7.82	8.76	10.41	10.24
FANCL	11.4	9.74	9.32	11.9	10.35	8.76	14.67	12.12
TBL1XR1	8.18	8.27	9.15	11.19	8.93	8.76	14.83	12.83
TOP3A	9.16	9.35	9.17	9.28	8.84	8.77	8.48	7.97
DIRC2	6.73	7.95	8.15	7.2	8.11	8.77	9.56	8.39
BCOR	10.99	10.62	10.82	11.01	7.78	8.77	8.87	9.1
TSPAN5	8.68	8.79	8.38	8.99	8.11	8.77	8.82	9.13
CNTNAP3	9.83	9.33	9.98	11.83	8.64	8.77	10.65	10.03
YES1	6.73	6.93	8.22	10.21	8.49	8.77	14.58	12.48
REV1	8.09	7.34	8.76	9.88	9.49	8.78	14.43	12.59
FBXL14	8.34	8.68	9.3	7.66	7.75	8.79	5.41	6.7
NMB	9.19	10.96	8.63	7.9	10.4	8.79	7.24	7.63
OASL	13.45	15.4	14.59	14.02	9.32	8.79	7.31	8.39
DDX6	8.58	7.73	9.46	9.83	9.24	8.79	10.75	9.43
PIK3R1	8.14	6.9	10.15	11.53	9.38	8.79	14.02	12.96
DYRK4	8.69	8.64	8.59	8.19	8.15	8.8	8.1	7.61
BCL9	10.01	10.69	10.84	10.59	9.25	8.8	9.24	9.64
ATL2	8.65	8.46	10.11	10.43	9.1	8.8	14.36	12.1
PNPLA4	8.42	9.01	8.29	8.04	8.32	8.81	7.63	8.02
DENND6B	9.36	10.96	9.18	9.75	7.83	8.81	8.02	8.56
SYNRG	9.12	8.98	9.06	8.75	8.63	8.81	9.61	9.62
AGO1	8.67	8.35	8.73	9.21	8.97	8.81	9	9.7
PPP6C	7.94	7.68	7.77	8.68	9.51	8.81	10.62	9.95
MDM2	6.11	5.74	7.27	8.81	8.94	8.81	14.23	12.97
THOC2	8.1	7.7	9.97	11.6	9.3	8.81	16.27	14.8
PCSK7	8.68	8.75	8.59	8.28	8.95	8.82	8.88	8.05
CYB561D1	10.17	9.27	9.46	9.87	9.82	8.82	8.96	9.34
COMMD8	7.1	8.24	7.38	7.12	8.49	8.82	10.98	9.38
CCZ1B	9.34	9.67	12.11	8.02	13.65	8.82	12.52	12.62
WDR4	9.78	8.71	8.52	8.07	8.61	8.84	8.15	8.58
AK6	9.52	9.43	8.05	7.69	9.95	8.84	12.41	11.49
TAF5L	9.42	8.97	10.04	10.22	10.16	8.85	9.71	9.22
GOLPH3L	10.02	9.12	10.65	11.07	8.9	8.85	10.41	10.17
ACER2	6.79	7.31	8.84	9.78	9.9	8.85	11.84	11.62
IFT74	8.01	8.12	8.67	9.29	8.26	8.85	12.75	11.97
RNF19A	8.98	8.17	10.85	12.24	8.86	8.85	15.33	12.39
MORN2	8.28	7.42	7.28	8.68	7.47	8.86	9.23	9
KCTD20	8.82	8.1	9.01	10.1	8.56	8.87	10.07	9.95
RBBP5	8.54	8.05	8.76	8.85	8.9	8.87	10.46	10.2
PURB	8.81	7.66	9.3	9.88	9.34	8.87	11.62	10.86
USP32	7.34	7.71	8.44	9.35	9.35	8.87	12.29	11.14
TGS1	8.29	7.81	9.45	8.66	9.74	8.87	12.95	11.41
SCO1	7.72	7.23	7.8	8.41	9.26	8.88	9	9.74
FAM216A	7.16	7.13	8.39	7.63	7.64	8.89	10.66	8.31
CHCHD7	9.63	8.77	9.01	9	9.88	8.89	10.52	10.53
IFT27	8.46	10.64	9.78	8.38	7.47	8.9	8.12	7.58
MIEF2	10.09	10.04	9.83	9.18	8.95	8.9	7.16	7.87
GTSE1	6.92	6.59	7.29	6.6	8.57	8.9	8.04	8.39
HIF1AN	8.35	9.06	9.07	9.46	8.65	8.9	8.55	8.99
TPD52L1	10.59	10.47	10.66	10.18	10.45	8.9	9.61	10.68
EEF1E1	10.58	9.02	8.07	8.3	10.59	8.9	12.21	11.45
NCOA3	7.54	7.1	9.08	9.23	8.84	8.91	12.68	11.39
GTF3C3	9.45	9.27	9.81	10.44	10.51	8.91	12.5	12.14
TWIST1	10.36	9.19	8.91	8.61	7.91	8.92	6.18	7.22
NUAK2	7.67	8.67	7.89	7.14	8.08	8.92	7.98	8.28
PRKX	7.12	6.59	8.16	8.34	9.5	8.92	10.67	10.01
CTBS	8.6	8.56	9.59	10.22	9.54	8.92	12.75	11.26
INPP5E	7.63	7.89	7.91	7.5	7.55	8.93	7.25	6.79
RDH13	8.22	8.07	8.69	7.95	9.41	8.93	8.24	8.11
ST7L	8.24	8.89	9.45	9.73	9.16	8.93	12.07	8.89
DTL	11.21	10.42	11.95	12.79	10.04	8.93	13.4	10.1
RFC3	10.02	8.3	10.85	9.56	8.47	8.94	12.14	11.39

RC3H2	7.61	6.5	8.79	9.68	8.59	8.94	13.06	11.79
CPSF2	7.67	6.53	8.35	9.24	9.63	8.94	13.18	12.04
SCAF11	8.32	7.3	10.12	12.11	8.88	8.94	16.9	14.31
POLE4	10.06	10.86	10.22	9.05	10.82	8.95	9.26	7.8
ASPHD1	8.84	9.03	8.28	7.5	9.29	8.95	9.07	9.44
CLCN6	7.09	7.38	7.32	8.55	8.97	8.95	10.49	10.47
KPNA3	8.41	7.76	9.83	10.52	9.73	8.95	12.84	11.83
CFLAR	8.87	8.23	9.4	10.68	9.42	8.95	13.73	12.05
TIMM8B	6.89	7.37	7.39	5.83	8.65	8.96	6.75	7.45
TMEM110	8.3	8.57	8.23	8.42	8.46	8.96	9.18	8.64
STRADB	9.6	9.84	9.44	9.49	8.84	8.96	10.7	9.78
KLF3	8.88	8.49	10.08	11.14	8.91	8.96	11.75	12.07
PLEKHH2	7.39	6.61	8.86	10.83	8.94	8.96	17.66	13.58
TRIM3	7.14	8.13	7.38	7.41	8.81	8.97	8.34	7.92
MIPEP	8.1	8.62	8.04	8.61	8.43	8.97	9.2	8.45
NUDT15	6.83	7.27	7.99	7.98	8.8	8.97	10.42	9.87
SESN1	8.21	7.01	8.11	7.88	10.54	8.97	12.7	10.9
FNBP1L	9.68	8.65	10.37	13.29	9.77	8.97	15.05	13.6
VEZT	9.01	8.45	10	12.11	9.44	8.98	13.53	11.58
CBR3	9.53	10.22	10.33	9.1	8.45	8.99	6.93	6.94
STAC	8.23	8.17	7.58	7.84	8.28	8.99	7.28	8.06
SLC35E2	7.87	7.67	8.12	9.78	8.55	8.99	9.79	8.82
RNF41	8.7	8.94	8.3	9.08	8.65	8.99	8.68	9.1
ADAMTS1	8.26	8.43	8.69	8.04	9.36	8.99	9.99	9.91
LOC105371063	7.73	12.13	11.17	8.72	7.99	8.99	13.22	11.26
ALDH16A1	11.41	9.88	9.67	9.3	8.6	9	6.61	8.69
GRHL3	4.56	4.25	4.68	4.52	8.95	9	8.39	9.16
DPH7	7.68	8.32	8.98	8.07	10.59	9	9.84	10.39
SLC25A32	7.79	7.43	8.11	9.66	8.69	9	13.57	11.3
CTTNBP2NL	8.81	8.87	9.84	10.56	10.15	9	13.2	11.38
PNISR	8	6.8	8.38	10.61	8.62	9	17.16	13.82
CNTNAP1	8.69	9.09	7.95	8.64	8.4	9.01	8.42	8.55
PBK	8.61	7.4	8.5	9.45	9.5	9.01	13.31	10.77
METTL6	8.35	7.6	7.2	8.19	7.51	9.02	9.78	8.04
ACOX1	7.7	7.99	8.34	8.31	8.67	9.03	9.94	9.49
L3MBTL2	9.85	10.23	9.94	8.91	10.09	9.04	8.58	8.06
MRPS18C	7.45	8.46	8.22	8.73	8.55	9.04	10.09	9.56
BTN2A1	8.81	8.91	8.46	10.76	9.25	9.04	9.72	10.39
PGM3	9.94	8.35	11.4	13.16	9.78	9.04	13.92	11.83
NUDT11	8.54	8.7	8.31	9.18	8.78	9.05	10.13	9.11
TMEM65	9.6	8.37	8.32	9.76	9.35	9.05	8.66	9.54
NUDT8	11.11	10.32	10.37	8.65	8.65	9.06	7.11	8.15
TRIM4	8.56	8.76	8.75	8.87	8.69	9.06	9.62	9.07
SMCR8	8.76	7.67	9.05	8.76	9.34	9.06	9.15	9.42
EXOSC8	8.67	8.44	9.32	10.21	10.75	9.06	14.39	10.12
ADAT1	8.04	8.12	8.26	9.64	9.12	9.06	11.08	10.18
ZNF263	8.72	8.89	8.21	8.32	9.22	9.07	8.42	8.72
C7	6.71	7.99	7.58	8.22	7.82	9.07	9.67	9.25
NUS1	7.25	6.82	8.27	8.16	8.63	9.07	13.11	9.92
GON4L	9.29	8.75	10.02	10.88	9.47	9.07	11.26	10.76
NPIPB5	7.29	8.23	9.78	14.19	9.31	9.07	16.93	15.46
CEMIP	4.47	4.73	4.68	4.86	8.44	9.08	8.67	9.34
NUMB	8.75	8.14	9.18	9.96	9.5	9.08	10.49	10.88
USP34	7.53	7.16	9.09	11.06	9.04	9.08	16.81	14.54
MSH3	7.99	6.58	8.29	9.51	9.37	9.09	12.62	10.84
ARHGAP21	8.68	7.85	9.83	11.47	9.23	9.09	13.71	12.48
FUZ	9.28	10.16	8.05	9.22	8.09	9.1	6.33	5.97
TRIM35	8.45	9.35	7.83	8.03	9.34	9.1	7.49	8.71
RIDA	8.78	8.29	9.68	9.63	10.04	9.1	15.53	11.2
SUV39H1	9.52	9.64	8.93	8.49	9.38	9.11	8.03	7.72
ZNF276	6.98	7.43	7.25	7.79	8.88	9.11	9.1	8.96
WDR48	8.21	8.19	8.98	8.56	9.67	9.11	11.91	10.42
PLEKHA5	9.32	8.24	10.04	12.16	9.58	9.11	13.06	12.62
HSPB8	8.71	8.81	8.27	6.79	11.19	9.12	9.41	8.71
HIRIP3	11.17	10.13	9.5	11.52	10.22	9.12	9.59	9.23
CCDC109B	9.49	8.77	8.99	9.23	10.03	9.13	9.21	7.62
SLC4A1AP	8.2	7.22	9.08	8.71	9.39	9.13	9.65	8.87
RAB2B	9.35	8.86	8.71	9.15	8.75	9.14	10.5	8.84
DTX3	9.75	10.05	8.96	9.18	9.53	9.15	9.02	9.56
MALL	9.32	9.11	9.17	8.64	9.29	9.15	9.3	9.6
UBE3B	9.03	9.12	8.43	9.54	9.53	9.15	9.69	11.22
KIF13B	9.36	9.11	8.85	8.62	9.22	9.16	8.21	8.78
TK2	10.31	11.11	10.09	10	8.48	9.16	9.56	9.2
GNB5	10.12	8.92	9.04	9.37	9.87	9.16	9.37	9.37
GIGYF2	9.25	9.13	10.24	10.36	10.04	9.16	10.3	10.61
MED4	9.35	9.46	9.86	10.61	10.68	9.16	14.44	11.6
NAV1	9.38	9.18	9.3	9.53	9.24	9.17	9.83	9.66
TMEM231	9.92	10.39	10.02	9.28	9.04	9.18	8.52	9.32
HIPK2	9.38	9.29	9.97	11.04	8.56	9.18	9.97	10.45
SMC2	8.68	8.34	9.68	11.37	9.73	9.18	16.86	14.18
RAD17	9.19	7.21	9.83	10.6	9.57	9.18	16.34	14.52
GZF1	9.25	9.05	10.1	9.59	8.47	9.2	10.27	8.45
CCNA2	7.54	6.25	7.36	7.41	8.8	9.2	10.88	9.45

C15orf40	8.67	6.91	9.14	9.61	9.18	9.2	10.99	9.59
DHTKD1	8.56	9.08	9.23	8.82	9.01	9.2	9.93	10
COIL	8.96	8.84	9.45	10.52	10.33	9.21	12.66	11.9
FNBP4	9.21	8.32	9.7	11.38	10.7	9.21	16.02	14.08
MICAL3	8.44	9.06	8.59	8.53	8.92	9.22	8.73	8.84
C1orf198	10.52	10.38	9.43	9.06	9.54	9.22	8.78	8.96
DBNDD2	8.68	7.73	7.86	7.65	9.85	9.23	9.51	8.99
MR1	9.23	9.69	9.7	9.77	9.86	9.23	10.86	9.44
SNX19	7.86	7.23	8.33	8.64	8.66	9.23	10.74	10.75
KIF18B	7.02	6.92	7.39	7.9	8.61	9.24	9.44	9.28
NUPL2	8.29	7.43	8.78	9.86	9	9.24	12.84	11.26
GJD3	9.39	9.62	8.94	8.86	8.9	9.25	6.8	8.1
KAT14	9.78	9.61	10.13	10.21	10.7	9.25	10.06	9.45
KDM1B	10.19	8.89	10.19	10.39	10.17	9.25	11.56	10.71
WDR41	8.6	9.3	10.35	11.74	9.87	9.25	12.67	11.2
ZMYM4	9.59	9.13	11.06	13.02	9.91	9.25	14.2	12.52
KIAA0754	7.63	7.04	7.71	7.72	8.41	9.26	9.74	9.05
MED13	8.32	7.67	10.07	12.1	9.84	9.26	15.21	13.46
ANTXR2	9.18	8.39	10.82	10.27	9.13	9.27	11.14	10.56
ANKMY2	8.59	8.48	9.08	9.91	8.51	9.27	13.09	10.73
SLC30A6	9.08	8.14	8.86	9.48	9.84	9.27	13.48	11.31
VPS45	9.61	8.51	9.88	11.28	10.12	9.27	13.48	11.42
UGDH	9.5	8.52	10.12	11.02	9.8	9.27	12.46	12.11
TRIM7	8.17	7.24	7.03	6.53	8.44	9.28	6.87	6.99
C11orf1	10.75	9.62	10.62	9.41	10.42	9.28	9.29	7.9
KIAA0556	8.85	8.06	8.96	8.98	8.98	9.28	8.59	8.53
RNF121	8.62	9.17	9.74	8.1	9.13	9.28	9.39	9.1
CLASP1	9.13	8.81	9.49	10.85	9.51	9.28	11.21	10.34
CAB39	7.73	7.2	8.32	9.77	9.08	9.28	12.78	10.87
NR2C2	7.88	7.33	8.41	9.64	8.84	9.28	12.43	10.89
C15orf52	8.46	8.23	8.5	9.05	11.99	9.28	12.31	10.99
NT5C3A	7.81	8.32	9.08	10.09	9.2	9.28	13.67	11.24
MORN1	9.39	9.8	9.3	10.02	9.05	9.29	7.49	8.13
ZNF212	8.27	9.15	8.19	8.12	9.18	9.29	7.76	8.69
NCAPD3	7.91	8.68	8.45	9.17	9.45	9.29	10.01	9.11
APBB3	8.99	7.86	8.65	8.13	9.26	9.29	11.63	9.7
LCP1	8.29	8.79	8.92	9.49	9.6	9.29	10.19	10.13
MRPL42	9.11	8.93	9.18	10.75	10.04	9.29	14.62	11.62
R3HDM1	8.98	8.29	9.24	9.9	10.16	9.29	12.45	11.89
POMGNT2	9.52	10.49	9.79	9.24	8.26	9.3	7.21	7.85
FOXRED2	8.69	8.55	8.94	8.67	8.3	9.3	8.38	8.92
LIMK2	10.08	9.98	10.67	10.22	9.14	9.3	10.07	9.17
CAMSAP1	8.68	8.02	8.55	8.73	9.01	9.3	8.62	9.33
ENC1	9.27	8.33	9.16	9.42	8.72	9.3	11.01	10.06
NXF2	9.01	10.21	9.67	10.15	10.06	9.3	10.67	10.25
CDCA3	6.81	7.24	7.56	5.95	9.25	9.31	8.54	8.01
CCDC107	11.41	12.1	11.17	9.99	9.89	9.31	7.67	8.07
RBM19	8.23	7.21	8.26	8.18	9.84	9.31	10.49	9.57
PLS1	7.76	7.96	9.33	10.41	9.13	9.31	15.6	12.42
BCAN	13.94	12.45	12.76	12.27	8.38	9.32	8.51	8.5
FHOD3	9.69	10	9.78	10.16	9	9.32	9.39	9.25
DNAL4	10.12	10.88	10.23	10.02	9.57	9.32	7.98	9.26
VRK1	9.13	9.05	10.36	9.49	10.22	9.32	11.58	9.28
HSDL1	8.6	7.59	9.22	10.46	9.43	9.32	12.1	10.21
AMMECR1L	7	9.15	8.45	8.73	7.38	9.33	10.42	9.63
ARID1B	9.35	8.14	9.13	10.2	8.39	9.33	9.04	9.68
ST6GAL1	8.83	8.94	9.03	8.94	10.02	9.33	9.9	10.2
PDCL	9.08	9.33	9.92	10.68	10.71	9.33	11.25	10.27
ARMC5	8.38	7.75	7.62	6.92	8.06	9.34	5.83	6.96
ABCC3	9.19	9.1	9.88	9.68	8.16	9.34	8.57	8.15
FBXO38	8.61	7.54	8.95	9.29	8.96	9.34	11.73	9.78
DDIT3	10.31	11.52	12.16	12.11	10.87	9.34	11.12	9.91
SPG11	8.1	7.12	9.4	9.98	8.74	9.34	12.17	10.93
EGLN1	12.17	10.56	12.5	13.42	9.42	9.34	12.42	11.07
DOCK7	10.01	8.09	10.71	11.97	9.39	9.35	14.32	12.22
GLIS2	10.47	10.89	10.47	9.57	9.04	9.36	7.4	8.25
MTHFSD	9.43	10.33	9.02	9.09	9.73	9.36	9.33	9.57
TRIM32	11.23	10.55	11.2	11.52	9.14	9.36	11.05	11.15
AKAP17A	10.11	9.23	9.71	10.12	9.92	9.37	9.3	9.49
COX10	8.33	7.84	7.9	8.13	9.76	9.37	9.89	9.8
ETV3	9.82	7.98	8.88	9.89	10.01	9.37	10.25	10.55
ZCCHC7	7.01	7.02	9.07	10.1	9.48	9.37	12.42	10.63
NAXD	9.14	8.88	8.28	9.02	9.22	9.38	9.45	8.78
KCNK1	9.32	9.55	10.1	9.79	9.97	9.38	9.49	10.21
SUCLA2	7.7	8.24	9.5	9.16	9.31	9.38	14.15	10.53
PUDP	8.19	8.49	10.23	9.25	9.93	9.39	9.5	9.26
MSRA	11.89	10.44	11.1	10.57	9.64	9.39	7.94	9.27
MFSD13A	8.22	8.46	8.69	7.75	10.05	9.39	9.17	9.62
GTF3C4	8.96	8.45	9.17	9.94	10.23	9.39	11.23	9.72
DNAJC19	9.34	8.52	8.59	8.43	10.46	9.39	12.72	11.4
CELSR3	7.94	8.16	7.98	7.96	8.19	9.4	7.71	8.21
TTL	8.11	8.3	7.54	8.72	9.31	9.4	10.66	10.17
DHX32	9.02	9.26	9.6	9.54	9.67	9.4	10.47	10.22
RNASEH2C	9.81	10.12	9.47	9.24	9.35	9.41	8.52	8.57
ARHGAP27	8.67	9.11	7.72	8.69	9.03	9.41	7.82	9.1

PRUNE	10.57	10.96	11.7	10.83	10.13	9.41	10.9	11.11
SMC6	8	8.62	8.81	9.77	9.5	9.41	14.8	13.02
L1CAM	10.6	10.68	10.58	10.43	9.55	9.42	8.17	8.51
TOX2	11.9	11.23	10.34	10.72	8.25	9.42	8.34	8.55
OSBPL1A	9.86	8.72	9.37	10.11	9.59	9.42	11.82	10.12
RPUSD2	9.56	9.06	8.73	8.7	8.85	9.43	7.43	6.86
LOC105379554	8.65	8.76	9.27	8.54	9.69	9.43	8.84	7.59
DUS3L	9.54	8.57	8.68	8.37	9.33	9.43	8.18	8.34
SZT2	9.25	9.51	9.52	9.5	8.98	9.43	9.7	9.8
POLR1B	8.65	7.93	8.63	9.02	9.9	9.43	11.47	10.32
STOM	9.3	8.08	9.72	9.91	11.28	9.43	12.18	10.75
ITGB3	9.15	9.85	9.5	10.14	11.09	9.43	12.12	10.86
FYTTD1	9.16	8.56	9.62	11.15	9.51	9.43	12.44	11.78
MAST3	9	9.29	8.77	9.1	9.15	9.44	8	8.76
DTNBP1	11.16	10.72	10.21	11.02	10.07	9.44	8.44	9
LIF	8.38	8.63	8.39	8.67	9.88	9.44	8.75	9.04
SLC7A6OS	8.48	7.94	7.65	8.76	9.03	9.44	9.32	9.35
DIP2A	8.2	8.62	9.04	9.55	9.45	9.44	10.44	10.37
ICAM3	8.43	9.13	8.23	7.38	8.83	9.45	7.02	7.65
ZCCHC14	7.38	7.53	8.96	9.34	9.02	9.45	9.03	9.16
MCM6	10.97	10.6	11.65	11.55	8.91	9.45	10.56	10.23
BAHD1	9.94	10.95	9.87	8.86	9.4	9.46	7.63	8.85
HACL1	11.59	9.83	9.55	9.79	10.89	9.46	11.62	10.07
CBFA2T2	9.41	9.04	10.19	10.41	9.08	9.46	10.29	10.26
RPAIN	9.01	8.06	9.14	10.09	10.32	9.46	12.08	10.54
SNAPC5	6.66	6.92	6.03	9.42	10.39	9.46	8.6	10.82
STXBP3	8.68	8.75	9.25	10.94	9.9	9.46	17.21	13.62
FIZ1	10.4	10.71	10.36	9.59	9.06	9.47	6.93	7.74
PDCD7	9.6	9.36	9.62	9.85	9.18	9.47	8.54	8.95
NOTCH2	9.63	9.44	10.02	10.28	9.37	9.47	10.52	10.52
C4orf3	12.87	12.22	12.75	14.44	9.41	9.47	11.93	11.49
MAPK6	8.24	8.2	10.31	10.94	9.96	9.47	14.28	12.26
FBXO31	9.2	8.89	8.53	8.53	9.45	9.48	8.07	8.55
FAM160A2	9.04	8.87	9.65	10.06	9.28	9.48	10.34	10.67
NRBF2	10.24	8.76	9.34	10.72	10.54	9.48	12.13	11.08
C1orf109	8.78	7.81	7.55	8.26	10.41	9.49	10.71	9.66
CEP250	9.76	9.68	10.62	10.36	10.11	9.49	10.99	10.05
CHFR	8.97	8	9.05	8.6	9.17	9.5	8.04	8.15
REXO2	7.03	7.42	7.25	7.41	9.79	9.5	9.39	10.11
SVIL	9.47	9.04	9.95	10.35	9.9	9.5	10.91	10.22
PAN2	9.62	9.17	9.59	9.83	9.76	9.5	11.81	10.42
PNPT1	10.81	8.98	11.16	13.81	10.07	9.5	15.07	12.86
SUFU	10.33	10.04	9.42	10.43	8.96	9.51	7.77	8.65
MTMR12	10.12	7.86	10	10.01	9.97	9.52	11.37	9.91
GPR89A	7.67	7.4	8.23	8.64	9.07	9.53	12.01	7.64
LTBP2	8.53	8.06	8.4	8.8	8.93	9.53	8.55	9.06
CLYBL	8.42	8.2	7.94	7.94	9.19	9.53	10.96	9.21
ACVR1	8.86	10.11	10.87	10.05	9.37	9.53	10.89	9.53
TBC1D7	9.59	10.93	10.07	10.48	10.83	9.53	10.68	10.69
SCAF8	10.04	9.55	11.05	12.04	9.98	9.53	12.18	11.76
TRNAU1AP	8.68	8.74	8.66	7.56	9.19	9.54	8.7	6.88
LRRC8D	8.5	7.29	9.66	9.82	10.19	9.55	13.12	11.22
RILP	8.96	9.02	9.35	7.29	8.61	9.56	7.6	8.01
NACC2	8.98	8.33	9.23	8.92	9.39	9.56	8.71	8.85
FBXW9	11.02	9.54	9.43	10.22	10.47	9.56	8.2	9.09
ZKSCAN5	8.69	9.13	9.67	9.74	10.19	9.56	10.5	11.27
PAK6	7.39	6.86	8.29	7.08	9.34	9.57	7.1	6.51
MOCS3	10.19	8.74	9.23	8.34	9.16	9.57	8.74	8.51
MBP	8.52	7.66	8.75	8.87	10.41	9.57	9.89	9.84
TRIM21	14.8	15.82	14.44	14.73	11.42	9.57	9.45	11.3
PLEKHG3	9.26	9.45	9.42	8.53	8.53	9.58	8.14	8.35
SMG8	9.4	9.74	9.9	9.04	9.01	9.58	10.34	9.2
PARP9	17.38	15.87	17.04	19.8	9.62	9.58	13.26	9.92
USP40	8.96	9.32	10.09	9.53	9.94	9.58	11.38	10.42
APITD1	8.59	9.09	9.35	10.36	10.22	9.58	10.72	11.21
NUF2	8.36	7.7	8.33	9.19	9.55	9.58	15.08	11.27
SLTM	8.36	8.31	9.8	12.88	10.55	9.58	15.75	15.78
CHST3	8.64	9.15	8.64	8.38	8.63	9.59	8.54	8.83
SLC29A2	8.21	9.17	8.66	9.11	7.98	9.59	7.99	9.48
STK35	8.87	9.81	8.98	8.65	9.34	9.59	8.96	9.5
NKAP	9.1	9.57	7.82	9.87	9.38	9.59	11.51	10.47
CDCA7	9.94	9.99	10.91	10.59	11.53	9.59	12.54	10.72
IRX2	9.45	8.77	9.9	9.25	8.61	9.6	7.86	7.66
ZNF771	10.36	9.8	10.23	9.88	8.14	9.6	6.45	9.36
SLC4A3	10.37	11.94	10	10.67	9.01	9.6	8.77	9.74
PTCD3	7.81	7.2	8.22	9.77	9.72	9.6	13.31	12.08
TMEM212	7.52	6.53	9.18	11.78	8.21	9.6	17.61	16.05
TFAP4	10.13	9.5	10.17	9.6	10.87	9.61	8.55	9.25
ADCY6	9.96	9.99	10.28	10.29	9.27	9.61	9	9.51
PTPRE	8.53	6.69	8.8	9.01	9.57	9.61	11.55	10.5
GAR1	8.15	9.53	10.21	10.7	11.87	9.61	11.69	12.95
PAPD4	9.17	7.8	9.72	11.89	9.65	9.61	13.65	13.55
SLC46A1	10.02	10.19	9.58	9.07	10	9.62	9.57	9.53
CREBBP	10.28	9.71	10.07	10.56	9.55	9.62	9.34	9.81
MAMDC2	11.49	10.94	11.51	11.97	9.42	9.62	12.37	10.77

SLC35E4	7.33	8.79	7.32	7.47	8.68	9.63	6.14	7.56
ECHDC1	8.31	8.28	9.46	10.35	10.84	9.63	15.41	13.1
CAPN7	8.83	8.78	9.11	9.91	9.68	9.64	13.27	11.72
LARP7	9.1	9.92	9.49	10.91	10.1	9.65	13.09	12.25
LRRC24	10.35	9.44	8.91	6.87	7.06	9.66	6.73	5.83
SPATA33	9.55	9.05	8.9	9.61	9.87	9.66	7.25	7.9
TOB2	9.01	9.56	9.37	9.38	9.17	9.66	8.09	9.02
TMEM80	8.61	10.06	10.09	11.01	10.56	9.66	10.81	11.1
COPS2	8.39	10.09	9.6	9.86	10.67	9.66	13.99	11.7
C18orf21	10.22	8.15	9.24	9.53	10.97	9.66	13.94	11.89
LOC100996717	8.89	8.18	9.26	9.47	9	9.67	7.44	9.31
ASIC1	10.92	11.8	10.59	10.96	9.54	9.67	8.71	10.22
MSL3	9.05	7.62	9.62	11.02	10.1	9.67	11.01	10.71
DYNLT3	9.46	9.56	8.95	9.55	10.5	9.67	14.76	11.84
TROAP	8.38	8.11	6.56	6.11	9.33	9.68	9.87	8.3
KANK1	11.65	10.32	10.8	11.21	9.75	9.68	11.02	10.34
GSTA4	10.99	12	13.07	12.16	11.66	9.68	14.2	11.49
BBC3	7.55	7.27	7.75	7.58	9.02	9.69	6.58	6.94
SMARCAL1	10.77	9.02	10.18	10.41	9.99	9.69	9.6	9.23
SELENBP1	13.49	12.96	12.64	12.18	9.38	9.69	9.95	10.21
C1orf131	8.83	8.02	9.99	9.07	9.26	9.69	12.49	11.49
GPSM2	7.64	6.66	8.06	8.49	9.77	9.69	14.22	12.04
MAD2L1	11.6	10.9	11.55	11.52	13.26	9.69	17.8	13.17
SH3PXD2B	11.01	11.26	10.38	10.48	9	9.7	8.51	8.68
SEMA4C	12.23	11.03	11.85	11.16	9.19	9.7	8.71	9.55
C8orf76	8.8	9.55	9.92	10.57	11.5	9.7	10.62	9.75
MORN4	8.43	8.68	8.45	8.37	10.17	9.7	10.18	10.25
SLC25A33	8.55	9.96	8.22	8.67	10.12	9.7	10.39	10.66
ATMIN	9.19	8.94	9.9	10.22	9.36	9.7	11.5	10.68
ZFP90	9.35	8.92	9.49	11.38	9.61	9.7	12.83	11.25
RSU1	9.23	9.34	9.57	9.73	11.3	9.7	11.14	11.57
RINT1	8.83	8.04	9.89	10.42	10.51	9.7	13.53	12.1
CACFD1	10.32	10.5	10.36	9.76	9.24	9.71	7.47	8.61
TMED5	8.24	8.39	9.04	10	9.51	9.71	13.98	12.3
SLC2A9	10.44	10.77	10.31	10.71	9.45	9.72	10.14	9.63
SETMAR	9.28	8.75	10.68	11.78	8.86	9.72	11.99	10.19
CHEK2	9.56	8.54	8.85	8.82	9.26	9.72	11.27	10.38
ANKRD36C	7.26	6.5	8.37	9.9	8.49	9.72	16.95	13.81
RNF135	9.08	9.2	8.5	9.08	9.37	9.73	8.51	8.2
BRF2	10.25	10.25	9.69	8.41	9.08	9.73	8.99	8.85
DGKD	9.26	8.17	8.78	9.31	9.95	9.73	10.16	9.43
MPHOSPH10	8.41	8.87	9.91	10.75	9.55	9.73	15.93	13.17
TMEM187	9.69	9.99	9.27	10	8.42	9.74	7.58	8.14
MAPK8IP3	9.17	8.96	9.18	9.74	9.38	9.74	10.08	9.71
NPR2	9.37	9.5	9.78	9.64	10.09	9.74	10.5	11.03
PHF6	7.56	7.47	8.97	11.23	9.24	9.74	14.88	13.53
HOXA3	10.06	9.72	9.62	10.39	9.66	9.75	8.15	9.43
FAM174A	8.83	8.99	9.5	9.15	8.84	9.75	8.78	9.67
GCC1	9.01	9.04	9.19	10.22	9.83	9.75	10.35	9.97
SLC35G2	7.37	7.51	8.04	7.97	8.94	9.75	10.09	10.86
MEGF9	7.24	6.73	8.19	9.22	9.43	9.75	12.92	11.21
TSPAN15	9.7	10.63	9.04	8.93	9.27	9.76	7.9	8.48
B3GNT7	11.29	11.96	10.76	10.4	8.36	9.76	6.97	9.4
MORC4	10.45	9.46	10.38	10.78	10.73	9.76	11.63	10.17
ITPRIPL2	9.76	9.57	10.08	9.76	11.39	9.76	10.11	10.81
SLMAP	7.96	8.2	9.53	11.48	10.56	9.76	13.57	13.49
LARP4	7.85	7.17	9.04	10.65	9.93	9.76	16.21	14.5
TMEM120B	8.1	8.45	8.4	8.93	9.98	9.77	9.11	9.33
TACC2	9.49	9.38	9.31	10.29	8.96	9.77	9.29	9.55
CD47	9.65	8.39	9.98	11.27	9.93	9.77	13.99	11.94
PLAT	20.64	20.64	18.92	17.32	10.18	9.78	9.69	9.65
ALG1L	11.92	11.48	10.78	11.55	8.86	9.78	10.43	11.4
C18orf32	11.2	9.35	7.15	10.62	9.8	9.79	11.14	8.06
TMEM98	12.14	13.36	11.09	11.76	9.65	9.79	9.52	9.36
MAP2K5	8.68	9.36	8.78	8.78	9.15	9.79	8.34	9.43
KMT2D	9.16	9.19	9.05	9.37	9.22	9.79	9.26	10.01
ABCD1	9.87	10.55	9.7	10.03	9.49	9.8	7.39	8.75
METTL22	8.54	8.4	8.68	8.86	8.65	9.8	9.14	9.1
RDH14	8.13	7.88	7.94	8.53	8.25	9.8	10.51	10.57
TULP3	11.3	10.44	11.04	11.63	10.58	9.8	11.33	10.72
KIF23	7.89	7.55	8.49	9.37	11.07	9.8	15.47	13.24
HEATR6	8.49	8.79	8.64	8.16	9.31	9.81	9.94	9.39
PARP3	8.87	9.46	9.55	9.96	9.36	9.81	9.23	10.02
SNX33	10.3	10.24	9.83	9.92	9.53	9.81	8.45	10.16
TOR1AIP2	8.94	8.64	9.52	11.56	10.01	9.81	13.02	13.04
TMEM177	10.18	10.68	10.24	8.78	10.46	9.82	9.12	9.46
LAMA3	10.15	9.08	10.26	11.24	10.09	9.82	12.33	10.06
PLAA	8.1	7.64	8.18	9.64	10.6	9.82	12.26	11.7
ERO1A	10.88	10.53	12.9	14.4	10.99	9.82	15.19	14.27
MED17	8.83	8.33	9.75	9.82	10.41	9.83	10.73	10.01
EXOSC9	10.76	9.61	10.68	10.58	11.22	9.83	12.69	11
DNM1L	8.57	7.47	10.08	11.42	10.03	9.83	16.22	14.09
ECE2	9.46	9.08	9.91	8.69	9.1	9.84	8.12	8.47

RCBTB1	8.12	7.68	9.33	10.58	10.57	9.84	13.36	11.29
HOXD10	9.25	7.88	10.18	12.09	10.18	9.84	14.03	14.84
GOLGA8A	10.11	8.69	10.36	14.02	10.19	9.84	22.97	16.81
P2RX4	12.06	11.75	10.45	11.11	10.8	9.85	7.4	9.63
C12orf49	9.49	8.83	9.31	9.24	9.79	9.85	9.33	9.82
EVA1C	11.17	9.47	10.12	9.25	10.58	9.85	11.02	10.04
HEXDC	10.04	11.02	10.65	9.42	9.26	9.86	9.41	8.46
SNX18	9.91	8.2	9.44	9.37	9.1	9.86	8.37	8.54
SUSD6	10.08	9.55	10.29	10.26	10.13	9.86	10.45	10.2
MBD4	9.49	10.67	10.33	11.52	10.06	9.86	15.03	14.87
PPP1R15A	10.68	10.12	9.41	9.82	12.3	9.87	9.28	10.12
CHD2	11.04	9.79	12.37	13.38	10.57	9.87	14.29	13.54
SPHK2	8.66	8.81	8.02	7.47	10.03	9.88	7.94	8.9
MRPS14	10.21	10.49	10.38	11.13	10.53	9.88	11.17	10.38
C1GALT1C1	8.98	8.49	9.3	10.08	11.15	9.88	14.46	11.05
QKI	11.32	10.33	12.09	14.12	9.66	9.88	14.35	12.04
SEC23IP	9.21	9.47	10.53	10.99	11.08	9.88	14.34	12.46
CASC4	7.91	7.38	9.15	10.78	8.86	9.88	14.37	13.39
POLE	9.92	9.55	9.74	9.38	9.43	9.89	9.62	9.18
RMDN3	9.57	9.69	10.12	10.02	10.49	9.89	10.29	9.97
SNRNP35	10.53	11.66	11.18	13.17	10.21	9.89	9.36	10.14
GPRIN1	8.42	8.27	8.21	7.26	8.99	9.9	7.06	8.58
BANP	12.42	11.6	11.43	10.5	9.84	9.9	9.3	8.59
CCL5	10.25	10.3	9.04	12.25	6.61	9.9	7.94	8.98
MAST2	10.19	10.06	9.97	9.53	10.16	9.9	9.35	9.75
PLEKHF2	9.76	8.39	9.87	11.03	10.38	9.9	15.63	11.62
TGFBR3	10.41	9.94	12.38	13.57	10.55	9.9	12.27	12.31
SCFD1	7.71	8.31	9.2	9.77	9.45	9.9	15.69	12.47
IFITM2	15.81	19.6	15.94	16.48	9.37	9.91	9.15	8.46
SPACA9	8.56	9.38	9.04	8.7	9.47	9.91	8.64	9.08
FLI1	8.5	8.16	9.51	10.46	10.07	9.91	11.39	9.38
CWF19L1	9.2	8.65	8.99	9.42	9.99	9.91	10.18	9.39
MRPL46	10.99	9.2	10.52	9.75	10.99	9.91	11.65	11.2
ZBTB17	11.14	12.55	10.72	10.5	10	9.92	8.73	9.27
DSN1	9.86	8.26	10.72	11.85	12.41	9.92	14.77	11.59
NME7	11.32	11.13	11.61	11.49	11.84	9.92	15.51	11.66
DZIP1	8.28	8.23	9.38	10.15	10.21	9.92	13.41	12.21
TMEM33	8.45	8.14	8.74	10.41	10.16	9.92	13.28	12.3
CYB561	9.48	9.84	9.18	9.89	10.06	9.93	9.38	8.41
KANSL3	9.57	9.75	9.78	9.7	9.92	9.93	9.86	9.81
LMTK2	9.07	9	9.53	10.19	9.83	9.93	10.57	10.2
PRKCI	7.69	6.92	9.14	11.61	9.69	9.93	16.1	13.27
RASA1	8.09	7.96	10.38	12.44	9.42	9.93	17.15	15
SPATA2L	10.61	9.9	10.99	8.92	9.29	9.94	7.7	7.77
SETD6	8.89	8.45	8.44	9.22	9.8	9.94	11.04	9.52
SDCCAG8	8.28	7.58	9.69	10.87	9.35	9.94	13.55	11.6
SLC35F5	8.92	8.94	10.21	10.77	9.87	9.94	13.48	11.76
FAM188A	8.4	9.2	10.12	10.68	9.48	9.94	13.11	13.1
TWSG1	8.69	8.57	10.08	11.95	11.17	9.94	16.75	13.87
HSCB	7.91	8.74	8.29	9.56	10.2	9.95	9	9.17
TMEM199	9.2	8.95	8.93	9.04	9.96	9.95	10.2	9.42
CNRIP1	9.6	9.97	10.45	10.8	9.94	9.95	9.54	10.88
HPS4	9.28	9.49	9.12	10.35	9.93	9.95	11.71	11.19
ZNF544	10.6	9.75	11	11.97	10.42	9.95	12.82	11.76
HECTD1	9.04	7.51	10.07	12.28	10.59	9.95	16.05	14.33
CAMK1	10.67	10.73	11.47	9.67	9.47	9.96	9.07	8.66
SLC5A6	8.22	9.04	8.77	8.59	10.33	9.96	9.29	9.29
ERICH1	10.32	9.76	10.65	11.23	11.77	9.96	13.09	12.35
LIMS1	8.83	8.03	10.13	11.71	10.35	9.96	14.6	13
CLDN12	8.95	8.41	10.48	12.08	11.35	9.96	17.62	14.47
CEP170	9.75	8.44	11.12	13.39	10.25	9.96	17.28	15.54
BIN1	10.86	11.55	10.81	9.42	9.42	9.97	7.1	8.32
NPIPA8	10.1	7.94	9.69	11.74	7.99	9.97	14.27	11.05
TRMU	9.4	7.19	8.36	8.33	10.07	9.98	9.78	9.38
TSHZ1	10.81	10.24	10.12	11.35	9.84	9.98	10.02	10.45
NVL	9.53	8.77	10.01	11.11	11.66	9.98	12.42	11.56
NT5DC1	8.64	8.65	9.31	9.83	9.64	9.98	14.31	11.63
KIAA0895L	7.03	6.65	7.38	8.9	9.34	9.98	12.37	12.3
AIMP1	8.98	8.33	10.1	10.48	9.96	9.98	14.86	13.78
ADD3	11.12	10.16	12.37	13.95	10.52	9.98	16.34	14.1
PIAS4	10.59	10.85	10.94	9.75	9.19	9.99	7.58	9.05
POMT2	9.21	9.93	9.09	9.15	9.46	9.99	8.52	9.46
MROH6	9.41	9.57	9.4	8.47	9.48	9.99	9.31	10.45
MCCC1	9.61	10.15	10.92	12.18	10.79	9.99	12.45	11.83
WFS1	9.98	10.98	9.27	9.8	8.98	10	7.72	8.84
ZNF410	9.84	9.97	9.96	10.14	11.06	10	10.81	9.4
GORASP1	10.08	10.68	10.35	10.91	9.26	10	8.12	9.7
CRADD	9.48	8.33	9.57	9.66	10.37	10	10.85	9.97
KANSL2	9.67	11.15	9.84	9.93	11.62	10	11.5	10.47
SNRNP27	9.78	9.48	10.78	11.18	9.26	10	12.25	11.07
NARS2	8.95	8.3	8.89	9.25	9.07	10	13.04	11.17
GEMIN4	9.74	9.65	9.52	8.71	10.21	10.01	8.27	8.69
PCDHB15	11.15	11.67	10.73	11.43	9.62	10.01	10.66	9.72
FAM86B1	8.05	8.19	9.19	8.42	9.04	10.02	9.5	10.72
C15orf39	10.8	11.33	10.32	9.89	9.9	10.03	8.11	9.6
PIN4	8.29	8.32	9.5	11.12	9.31	10.03	10.43	11.32
NDC1	8.76	7.82	10.01	9.86	11.51	10.03	14.27	11.65

NPIPA1	10.07	9.58	10.96	10.69	9.73	10.03	11.55	12.35
NFATC1	10.19	10.03	9.67	10.51	8.89	10.04	7.02	8.12
LIME1	8.6	8.3	8.21	8.25	9.68	10.04	9.76	9.38
FHL2	14.04	13.81	13.72	13.12	11.64	10.04	11.58	9.76
SNX9	8.4	8.34	9.6	10.98	9.63	10.04	12.23	11.82
KRT19	9.32	8.97	8.44	8.22	9.35	10.05	8.2	8.24
MICB	8.4	9.02	8.29	8.42	9.59	10.05	9.64	9.26
TSC22D3	10.03	11.12	9.85	10.23	9.42	10.05	8.53	12
AHCTF1	8.7	7.59	10.12	12.15	10.23	10.05	17.68	15.36
ANO10	9.43	10.94	9.78	9.21	10.63	10.06	10.19	9.62
EXTL2	9.86	8.92	11.01	12.15	9.23	10.06	14.76	13.16
RP9	12.16	12.38	12.97	13.5	12.38	10.06	11.74	13.3
MKRN2	10.07	9.6	10.06	10.27	10.48	10.07	12.1	10.51
KITLG	6.12	5.85	6.53	7.44	10.95	10.07	16.82	13.93
PAQR7	8.13	8.06	8.93	8.49	8.83	10.08	8.13	9.57
UBE2G1	9.11	8.97	8.92	9.31	9.14	10.08	11.08	9.63
CLCC1	10.31	10.24	10.52	12.32	10.97	10.08	14.82	13.19
POC1A	9.06	8.38	7.69	6.99	9.9	10.09	8.8	8.22
PXDC1	11.14	11.28	11.6	10.29	10.27	10.09	9.22	9.65
MRS2	8.76	7.92	8.52	9.77	9.92	10.09	11.41	9.76
ABI2	9.62	9.4	11.36	10.76	10.02	10.09	13.19	11.67
HPS3	8.98	8.18	10.05	11.73	9.62	10.09	15.13	12.93
CEP95	8.58	8.16	10.67	11.41	10.99	10.09	16.63	13.68
PCM1	8.19	7.49	10	12.56	10.42	10.09	19.5	15.75
ABI1	9.7	8.61	9.43	11.93	10.5	10.1	14.91	12.38
COG7	10.12	8.92	8.76	9.63	9.15	10.11	9.96	9.23
PEX26	8.44	9.47	9.05	9.37	9.75	10.11	8.83	9.66
SDHAF4	8.84	10.71	10.71	10.44	8.99	10.11	10.99	10.22
PARP2	9.14	9.23	10.16	10.61	11.51	10.11	11.54	10.73
COA7	8.41	8.27	9.04	10.45	10.29	10.11	12.81	11.81
CHD6	10.44	9.63	11.29	13.27	9.84	10.11	13.57	12.43
FUT11	12.33	12.24	12.98	11.88	9.69	10.12	9.91	8.72
KIFAP3	10.84	9.45	10.97	11.03	10.05	10.12	12.15	10.09
FASTKD5	8.68	8.65	8.88	8.72	10.19	10.12	11.66	10.37
DNAJC17	9.52	9.63	10.76	8.65	8.85	10.13	8.51	7.3
E4F1	10.37	9.16	10.14	9.01	9.57	10.13	8.24	7.87
HDHD3	10.61	10.15	10.06	9.36	10.22	10.13	8.71	9.48
SNUPN	10.49	10.4	8.73	10.19	12.95	10.13	9.87	10.49
MBD2	11.12	8.82	11.21	10.79	10.37	10.13	10.35	10.75
PIGF	11.56	11.01	10.54	12.31	12.38	10.13	17.07	14.29
HR	11.91	11.47	11.45	10.73	10.7	10.14	8.69	9.6
SARM1	9.47	9.44	9.17	8.9	8.94	10.15	8.01	8.76
TADA2A	9.13	8.42	9.47	9.31	10.23	10.15	10.08	9.56
KIZ	9.02	8.57	10.38	10.32	11.14	10.15	14.11	11.99
MRPS25	9.28	9.13	8.86	10.1	10.28	10.15	14.31	12.47
ARL1	10.17	8.81	9.65	11.54	10.6	10.15	13.93	12.66
KIAA1161	9.63	10.75	9.52	9.8	8.94	10.16	9.01	10.03
ANKS1A	10.24	10.04	9.76	9.5	10.22	10.16	9.43	10.56
C17orf80	9.42	8.26	9.78	9.99	9.97	10.16	12.85	12.09
TRIM24	9.52	9.02	10.57	12.09	10.95	10.16	14.29	12.47
STARD10	9.36	9.15	8.53	7.38	9.95	10.17	7.81	8.49
DIS3L2	9.94	8.96	9.14	9.01	9.71	10.17	8.74	8.7
CNPY4	9.96	9.39	9.72	11.62	8.8	10.17	9.88	10.44
SLC9A8	9.94	9.69	10.69	10.29	10.51	10.17	10.88	11.01
MSH2	9.14	8.4	10.59	11.38	10.53	10.17	16.14	13.18
SMARCA5	9.3	8.49	11.24	13.12	10.34	10.17	15.18	13.94
GOLGB1	7.44	7.42	9.54	11.42	9.47	10.18	19.17	17.14
POP1	8.57	8.47	9.59	9.28	10.15	10.19	10.03	10.45
GAS8	8.98	9.09	9.4	9.15	9.28	10.2	9.71	9.58
KMT5C	10.92	10.69	10.5	9.36	10.01	10.2	8.89	9.78
ZNF346	10.57	9.86	10.77	11.34	10.78	10.2	10.15	9.88
FAM57A	10.32	10.63	10.85	10.17	9.91	10.2	8.63	10.18
KIDINS220	9.09	8.54	10.65	12.33	10.66	10.2	15.1	13.84
COPS4	9.04	9.55	9.39	10.04	10.97	10.21	11.75	9.53
FANCA	10.07	9.83	10.52	10	10.51	10.21	10.9	9.58
ZSWIM1	11.23	9.62	11	9.37	10.25	10.21	10.25	11.11
KDM4C	11.83	11.36	11.76	13.24	11.21	10.21	13.16	12.26
CWC27	10.34	11.18	9.93	12.39	9.62	10.21	12.58	12.35
ELN	18.72	19.19	17.33	16.5	9.73	10.22	7.88	8.49
AARS2	8.85	9.09	9.2	8.58	9.31	10.22	8.46	8.58
SOX13	12.17	12.07	11.65	10.79	9.28	10.22	8.9	10
TRAF3	11.16	10.22	10.79	11.55	10.31	10.22	10.25	10.98
MRPS31	8.97	9.13	10.33	10.45	11.41	10.22	13.56	12.06
MITD1	10.09	8.64	10.48	11.5	11.49	10.23	14.37	11.51
PREPL	8.8	8.29	9.99	11.75	10.08	10.23	16.02	13.58
PQLC3	9.22	7.27	9.15	7.62	9.25	10.24	10.69	9.06
PHAX	8.91	7.94	9.27	10.64	10.58	10.24	14.01	13
PTPN18	9.55	9.31	9.74	9.61	9.51	10.25	8.26	8.58
PCDHB14	12.21	12.85	12.08	13.6	9.91	10.25	10.65	10.76
RILPL2	10.61	11.75	10.48	10.48	10.4	10.25	10.85	10.87
GSK3B	8.43	8.44	9.49	10.91	10.37	10.25	12.53	11.7
ZFAND2A	10.95	9.17	8.09	8.36	11.98	10.25	10.67	11.72
ABCA1	6.57	7.02	7.26	9.37	9.4	10.25	14.92	13.94
ADI1	10.78	11.79	10.02	10.27	11.57	10.26	8.81	8.64
TXNDC15	12.23	11.49	11.28	11.64	10.86	10.26	10.69	11.23
THUMPD3	10.13	9.59	11.19	11.93	11.13	10.26	15.6	13.13
DHX29	8.08	7.46	8.82	10.87	9.79	10.26	14.87	13.73

ASH1L	9.72	8.8	11.03	13.67	11.07	10.26	15.84	14.48
FAM208A	9.22	8.65	10.96	13.13	10.28	10.26	18.31	15.67
SLC25A19	9.72	7.87	7.98	8.33	10.75	10.27	8.86	8.72
PHF2	11.83	11.55	11.7	11.02	10.67	10.27	10.11	10.89
DSP	10.14	9.46	11.23	11.78	10.75	10.27	12.62	11.85
OGFOD3	10.73	10.67	10.86	10.13	9.85	10.28	7.82	9
CDH4	12.06	12.67	11.28	11.61	10.35	10.28	8.67	9.85
IFNAR1	8.65	7.97	9.29	10.47	10.51	10.28	13.32	11.14
NCEH1	7.58	7.58	8.95	8.73	10.06	10.28	12.72	11.51
PRMT3	6.61	7.04	8.22	8.69	9.78	10.28	14.64	11.87
CCAR1	8.93	8.44	10.37	12.22	10.52	10.28	17.99	15.12
ARHGAP17	10.47	10.94	10.95	11.58	11.25	10.3	9.49	10.34
ARL6IP6	10.73	10.65	10.82	12.41	9.23	10.3	12.91	10.98
OXSR1	8.94	8.83	10.16	10.68	10.85	10.3	13.85	11.83
C6orf120	9.74	9.18	10.05	12.23	8.93	10.3	13.74	12.5
TMEM41B	8.95	9.19	9.83	12.01	9.96	10.3	15.05	14.2
BROX	8.34	7.99	8.86	11.32	9.98	10.3	18.83	15.21
DENND1A	10.25	10.28	10.47	10.49	9.91	10.31	8.88	8.97
ST3GAL1	9.63	10.23	10.11	10.87	10.87	10.31	10.72	10.75
THADA	9.59	8.57	9.71	9.57	10.75	10.31	10.59	11.21
CSNK1G3	7.79	7.88	8.99	10.55	9.36	10.31	14.96	13.31
EML4	12.39	10.58	13.36	14.07	11.79	10.31	15.64	13.41
NPRL2	10.45	10.92	8.78	9.46	10.42	10.33	10.33	10.29
MEAF6	9.72	8.97	9.5	10.43	11.04	10.33	11.52	11.33
PPIE	9.26	11.3	8.32	8.36	11	10.34	9.7	8.81
ZNF133	9.47	9.01	9.35	10.4	9.46	10.34	11.91	11.13
DCAF10	10.25	9.93	10.45	11.17	10.17	10.34	11.55	11.88
TIA1	10.47	9.13	10.83	13.31	10.23	10.34	19.59	15.71
KIF4A	10.46	9.02	9.35	10.02	11.2	10.35	11.78	10.02
INPP5K	10.01	10.22	9.94	10.06	10.6	10.35	9.25	10.05
NEIL2	9.52	9.92	10.62	10.25	11.1	10.35	10.86	10.61
ACYP2	9.15	10.42	8.33	9.13	10.68	10.35	11.48	11.59
NSD1	9.21	8.9	9.85	10.37	10.56	10.35	12.54	11.68
DIEXF	8.89	8.09	10.3	10.12	10.5	10.35	13.7	12.09
SMOX	11.13	11.56	11.82	10.5	10.29	10.36	8.01	9.07
FBXO11	10.8	9.75	11.77	12.15	10.33	10.36	14.51	13.28
CACTIN	9.46	10.31	9.38	8.97	9.35	10.37	7.83	8.84
IFFO2	12.08	12.82	12.39	11.74	10.66	10.37	10.42	10.28
SLC25A17	9.72	9.17	9.7	9.12	11.44	10.37	11.26	10.29
HKR1	10.56	10.92	11.12	10.68	10.9	10.37	11.1	11.07
VPS41	9.49	8.97	11.07	10.7	11.71	10.37	15.38	13.63
PRKAA1	9.1	8.81	10.78	12.78	10.68	10.37	18.81	15.05
ZNF408	10.73	10.13	9.58	8.91	8.88	10.38	6.74	7.42
IFFO1	9.33	8.92	9.04	9.72	10.07	10.38	11.65	10.93
TRIM33	8.36	7.95	11.04	11.87	9.5	10.38	13.71	12.65
IQCB1	10.22	10.75	11.63	13.9	12.27	10.38	17.26	13.82
ZNF777	10.75	11.7	11.06	10.96	8.77	10.39	7.77	8.62
THTPA	10.01	9.6	10.3	9.18	10.75	10.4	9.53	10.67
AP4B1	9.59	10.45	11.82	10.07	10.1	10.4	10.77	11.25
SLC31A1	10.82	11.26	12.24	11.34	11.23	10.4	11.83	11.48
ATP13A3	9.03	8.02	9.83	12.32	10.69	10.4	18.29	15.04
MRM1	9.12	8.14	8.23	7.75	11.79	10.41	8.22	8.27
C6orf47	9.1	9.83	10.57	9.53	9.43	10.41	9.36	8.81
FOSL2	13.31	11.66	13.27	11.5	10.36	10.41	9.6	9.83
RUNX1	11.92	10.53	11.9	12.46	10.31	10.41	10.81	11.2
DUS2	10.07	10.01	9.1	9.55	10.88	10.42	9.77	9.89
FAM92A1	7.9	8.24	9.68	8.92	9.97	10.42	12.57	11.12
C1orf159	10.88	10.38	10.33	9.69	10.28	10.43	9.99	9.08
CLP1	8.85	9.61	9.5	9.33	11.27	10.43	10.14	9.84
MRPL39	9.59	8.57	9.63	9.24	11.96	10.43	14	12.07
TXNRD2	12.46	12.15	11.63	10.89	10.21	10.44	8.77	9.22
PSEN2	10.16	10.91	10.88	9.78	10.04	10.44	8.92	10.08
IPO8	8.95	8.24	9.81	10.15	10.35	10.44	13.93	12.04
MAPK7	11.86	12.87	11.55	10.83	10.49	10.45	8	9.64
CDYL	11.39	10.24	10.97	11.02	10.31	10.45	11.75	10.69
GATAD2B	10.27	11.45	11.11	11.1	10.31	10.45	10.8	11.98
ORC3	9.39	8.78	10.61	9.61	10.6	10.45	14.17	12.14
CNNM3	10.25	9.89	10.71	9.53	9.75	10.46	7.73	8.06
IRAK2	12.52	12.15	12.45	12.37	11.01	10.46	10.34	10.63
ZNF446	11.61	10.78	10.57	8.92	10.07	10.47	8.78	9.12
FBXL5	9.81	8.65	9.87	9.33	10.6	10.47	12.08	10.75
AAMDC	12.69	14.25	11.33	11.96	12.24	10.47	12.75	12.97
AEN	9.09	8.6	8.44	8.5	11.84	10.48	10.95	9.52
COG2	9.38	9.23	10.23	8.98	9.93	10.48	11.41	10.84
TBC1D20	11.21	10.83	10.53	11.42	9.54	10.48	11.07	11.25
MED9	10.05	10.19	9.55	9.18	10.79	10.49	9.39	9.9
PPP2R2D	9.86	9.91	10	10.69	11.62	10.49	11.43	10.97
SS18L2	9.32	10.47	9.7	10.16	8.85	10.49	10.97	11.09
SMAP1	10.78	9.44	10.62	11.84	10.59	10.49	12.57	11.98
TMEM189-UBE2V1	16.32	9.98	12.09	13.74	10.4	10.5	8.86	5.76
C9orf114	10.55	10.61	10.19	10.3	10.38	10.5	9.26	9.8
STX18	9.55	9.81	11.97	10.16	11.8	10.5	11.27	11.48
EIF4EBP2	10.85	10.96	11.63	11.5	10.36	10.5	11.61	11.59
SEL1L	9.75	8.94	10.36	11.61	11.29	10.5	15.04	13.97
NOL8	8.3	8.25	9.45	11.99	11.27	10.5	19.16	16.62
CLPB	8.97	9.12	7.95	8.59	10.19	10.51	8.51	8.98

DMRTA1	10.14	9.56	11.04	12.35	10.6	10.51	14.6	12
TSFM	9.89	9.49	8.83	8.18	11.06	10.52	9.58	9.44
MAP3K12	9.75	11.15	10.36	10.71	9.8	10.52	10.33	11.5
HS2ST1	10.05	8.98	11.81	13.8	12.09	10.52	14.4	13.91
NSRP1	8.41	8.32	10.13	12.34	11.33	10.52	16.76	15.89
BCAR3	10.3	9.92	10.18	9.98	10.61	10.53	9.32	10.11
PHKA2	9.93	9.83	10.26	10.22	9.74	10.53	11.67	10.48
NCAPH	11.26	9.38	9.23	9.9	11.03	10.53	10.8	10.54
DXO	10	8.84	9.88	10.19	11.26	10.53	10.71	10.93
SLC25A28	11.61	11.51	12.33	11.98	11.31	10.53	10.41	11.45
FAM168A	11.12	11	11.62	11.54	11.08	10.53	11.52	11.48
LRRC17	9.94	9.66	10.29	11.36	9.77	10.53	13.56	13.46
RAPGEF1	11.09	10.34	10.43	10.47	10.39	10.54	9.31	10.12
MBOAT2	7.71	9.88	9.32	10.89	10.23	10.54	13.36	12.24
RHOT1	10.08	9.53	10.66	12.31	12.2	10.54	16.03	14.03
ANKRD39	10.57	10.2	9.54	8.79	9.7	10.55	8.54	7.95
OGFOD2	9.85	8.9	9.76	8.77	11.09	10.55	8.62	9.17
C22orf39	11.96	9.69	10.43	10.3	10.28	10.55	10.32	9.86
TMEM51	10.28	11.47	11.29	10.38	10.84	10.55	7.87	10.21
VMAC	10.42	11.21	10.44	10.91	9.6	10.55	9.32	10.43
ENDOD1	12.38	11.96	12.36	12.47	10.18	10.55	11.66	11.11
PVRIG	7.62	7.96	8.38	9.48	10.69	10.55	13.17	11.94
OSBPL3	9.29	9.02	10.21	11.21	12.06	10.55	16.2	13.95
GPBP1	10.75	10.55	12.43	14.46	12.64	10.56	18.61	16.29
CEBPD	12.27	12.33	12.4	10.72	8.94	10.57	7.98	8.45
NPHP4	9.98	9.82	9.84	9.61	9.63	10.57	9.09	9.07
PDGFC	10.81	10.54	11.56	14.12	12.12	10.57	13.87	12.87
BLZF1	10.39	9.76	11.66	12.26	11.02	10.57	17	13.64
AGGF1	9.67	9.41	10.93	13.54	11.41	10.57	16.07	14.77
ELL	9.81	10.67	10.3	9.15	9.48	10.58	8.2	9.12
PIGX	10.34	9	10.41	10.82	11.02	10.58	14.48	11.95
TNKS2	10.16	9.35	10.97	12.68	10.93	10.58	15.88	14.43
DHRS7B	10.14	10.33	9.39	9.48	9.96	10.59	8.42	9.88
DNAJC1	10.7	10.96	11.54	11.35	10.89	10.59	10.41	10.03
ZNF185	11.97	11.27	11.6	10.68	11.72	10.59	10.8	10.25
BTF3L4	9.81	9.92	10.49	10.91	11.72	10.59	13.81	11.7
ERP44	10.03	10.25	12.18	11.9	10.47	10.59	13.92	11.7
ATF7	11.48	9.02	9.85	10.96	10.4	10.59	11.19	12.29
MEX3C	10	9.5	11.74	12.13	10.55	10.59	13.37	12.59
MSMP	8.32	8.53	6.14	9.54	7.12	10.6	4.79	7.01
KDM4B	11.94	12.26	11.61	11.18	10.01	10.6	8.76	9.51
TFPI2	13.05	12.88	12.87	12.58	9.67	10.6	11.81	9.85
RHBDL1	5.01	4.48	4.69	5.42	10.27	10.6	11.19	10.22
OTULIN	9.58	9.2	10.24	11.32	10.52	10.6	12.93	11.66
TERF1	10.08	11.22	11.84	12.43	11.45	10.6	15.71	14.18
CENPW	8.72	8.66	7.74	8.55	10.06	10.61	9.92	8.35
DPH1	10.07	10.83	10.05	9.2	11.39	10.61	8.99	11.16
CSRP2	15.18	12.65	13.3	13.32	11.37	10.62	11.91	11.43
ALG2	10	9.73	9.75	10.23	11.03	10.63	11.81	11.15
KYNU	10.14	8.5	10.84	10.03	11.08	10.63	15.61	12.21
GOLGA5	11.01	9.34	11.25	12.71	11.78	10.63	15.6	14.2
SMPD2	11.52	12.15	11.03	9.39	12.22	10.64	9.3	9.77
DYNC1I1	10.3	11.24	11.52	12.06	10.99	10.64	12.23	12
ZNF638	9.14	8.95	11.36	12.92	10.16	10.64	17.37	14.97
PLEKHO1	11.41	12.92	11.55	11.13	10.13	10.65	9.38	10.75
CEP41	10.71	10.3	10.93	11.62	10.67	10.65	13.33	11.79
BST2	28.98	30.39	28.97	29.55	8.99	10.66	9.19	7.29
TGIF2	10.61	10.94	11.24	10.96	10.5	10.66	11.15	11.23
FBXW2	9.14	8.89	10.48	11.08	11.28	10.66	13.52	12.57
B3GNTL1	8.54	8.94	7.62	8.6	10.01	10.67	9.04	8.58
TMEM150A	11.7	11.41	10.74	9.86	11.21	10.67	10.27	10.92
TMEM263	9.43	8.69	11.05	13.96	10.7	10.67	16.34	14.98
RECQL	9.36	8.68	9.72	11.68	10.66	10.67	18.83	15.04
SERTAD2	10.6	10.62	11.21	11.77	11.35	10.68	13.69	12.73
NUCB2	11.86	9.89	11.3	11.91	11.18	10.68	16.12	14.29
METTL1	9.69	11.41	10.28	9.47	11.88	10.69	9.41	9.14
SLC26A11	10.33	11.18	10.53	9.94	10.02	10.69	9.07	10.06
SOX7	12.38	12.45	12.94	12.05	10.79	10.69	11.06	10.99
C10orf2	7.86	9.18	8.73	8.45	11.42	10.69	10.35	11.09
GATC	10.71	10.78	10.71	10.44	11.59	10.69	12	12.03
AKTIP	10.8	10.91	11.85	12.06	12.33	10.69	14.5	14.01
SSX2IP	10.48	9.74	11.79	13.58	10.97	10.69	17.53	15.18
SH2B2	12.02	11.77	11.37	10.63	10.15	10.7	6.69	8.49
GMEB2	9.73	10.75	10.38	9.77	11.17	10.7	7.95	9.96
PCGF3	9.8	9.62	10.56	11.72	10.71	10.7	12.05	11.91
PHF14	9.53	8.75	10.65	12.62	11.68	10.7	16.01	14.65
GPATCH3	9.18	10.25	9.79	8.44	9.95	10.71	8.75	8.92
NAGPA	8.47	8.21	8.94	7.94	10.36	10.71	8.19	9.05
USP4	10.95	10.94	10.94	11.61	11.79	10.71	11.86	10.95
ANKRD52	10.42	10.52	10.88	11.29	10.84	10.71	10.94	11.31
TP53INP1	7.38	7.2	8.33	9.34	11.03	10.71	15.34	14.72
SULF1	8.96	9.48	10.06	10.54	11.27	10.72	12.97	11.85
KLHL7	10.71	9.65	9.78	10.8	11.11	10.72	12.37	12.09
CAMK2N2	7.23	7.16	6.62	5.35	9.83	10.74	6.61	7.37
SUPV3L1	9.54	8.99	10.13	9.64	12.1	10.74	12.98	10.55
CYB561D2	10.27	11.3	10.4	9.2	11.64	10.74	9.73	10.62

DDX55	8.71	8.46	10.17	9.79	11.48	10.74	12.91	11.1
HERPUD2	11.17	10.86	11.46	11.84	11.15	10.74	12.78	11.57
TREX1	13.37	13.4	12.99	11.67	10.86	10.75	9.71	9.78
FAM127C	13.23	12.67	12.56	12.5	11.19	10.75	9.1	10.13
RNF14	10.13	9.01	10.2	10.18	11.08	10.75	12.51	12.52
DPY19L1	9.81	8.82	10.63	11.56	11.09	10.75	15.5	12.85
RB1CC1	9.28	9.03	9.68	10.36	10.55	10.75	16.16	15.16
TPRN	8.58	9.7	8.04	8.04	9.09	10.76	7.2	6.46
ADO	10.14	9.49	10.46	10.94	11.39	10.77	12.48	11.36
LOC105371303	9.09	8.82	8.99	10.66	10.14	10.77	10.39	11.93
GDF11	8.11	9.32	8.96	7.85	9.71	10.78	7.49	8.51
AMBRA1	11.44	11.53	11.75	10.96	11.03	10.78	9.02	9.77
ATG7	10.31	10.36	9.91	9.82	10.4	10.78	10.82	10.93
DESI2	10.1	8.98	10.99	11	11.03	10.78	14.46	13.01
ATF7IP	10.05	10.48	12.95	13.62	10.81	10.78	15.18	14.98
SRSF8	10.15	9.36	9.74	9.96	10.91	10.79	12.05	10.43
CBS	14.75	11.71	9.32	10.02	12.86	10.79	10.88	11.34
CAMSAP2	9.45	8.82	11.08	13.59	11.21	10.79	17.92	15.81
PPIG	11.41	10.96	11.96	13.7	12.1	10.79	18.94	18.38
ABCB8	9.24	9.53	9.21	8.28	9.96	10.8	8.69	8.81
RASSF4	11.41	11.2	11.3	12.04	11.74	10.8	13.32	12.67
ANKIB1	8.83	8.52	11.42	13.29	11.19	10.8	17.88	15.29
BAZ1A	10.26	9.06	11.12	13.56	10.88	10.8	19.2	16.54
SNAPC3	9.54	9.78	11.68	11.86	10.62	10.81	15.28	14.46
ABHD14A	9.32	11.66	9.39	7.67	10.57	10.82	7.89	8.19
C6orf136	9.21	8.94	9.96	8.84	9.84	10.82	10.53	10.42
CXorf56	11.84	10.14	11.1	11.17	12.69	10.82	11.64	11.7
C5orf24	8.83	8.81	11.38	13.45	10.35	10.82	18.68	15.37
TMCO6	8.66	10.02	9.66	10.56	12.16	10.83	10.97	9.17
BRI3BP	12.25	10.94	12.61	12.25	9.38	10.83	8.99	9.85
HIPK1	10.66	10.97	11.71	12.35	10.88	10.83	13.71	13.45
ZC3H4	10.96	11.22	10.58	9.89	10.59	10.84	9.44	9.26
UAP1	9.75	9.61	11.4	11.81	11.38	10.84	14.24	12.05
CBR4	9.97	9.84	9.31	10.84	11.77	10.84	14.34	12.31
NSL1	10.69	9.97	10.68	11.24	10.97	10.84	12.03	12.68
MTRNR2L8	8.73	9.84	9.09	8.48	9.45	10.85	9.02	11.99
MED1	9.77	9.27	10.77	12	11.39	10.85	14.19	12.79
C11orf54	9.17	7.85	10.3	10.77	11.83	10.85	16.61	14.11
CCNF	9.05	9.27	8.95	7.26	11.53	10.86	9.55	9.98
ANAPC1	9.7	9.05	9.91	10.03	10.89	10.86	13.11	12.06
ZDHHC6	9.62	8.96	10.14	10.76	10.43	10.87	14.94	13.76
SKAP2	9.66	8.68	10.75	13.2	10.86	10.87	16.54	14.76
ULK3	10.02	9.53	9.58	9.22	10.37	10.88	10.75	9.46
CCDC22	11.45	12.01	10.6	10.31	11.45	10.88	10.8	10.09
TAF11	8.02	7.93	8.71	9.32	8.97	10.88	11.63	11.15
APIP	7.88	7.18	7.8	10.57	11.58	10.88	14.65	11.91
SLC11A2	11.17	11.29	12.5	12.22	12.1	10.88	13.92	13.34
TMEM158	14.26	12.48	12.87	11.95	9.94	10.89	8.01	6.9
SIX5	11.84	11.16	11.16	10.48	9.97	10.89	7.9	8.49
NIF3L1	10.14	9.29	10.28	11.46	9.92	10.89	12.62	11.24
MTURN	9.06	8.17	8.86	8.9	12.62	10.89	13.26	13.13
VPS26A	9.11	9.9	10.31	11.7	11.28	10.89	16.23	14.32
SCD5	11.03	11.24	12.1	13.31	11.41	10.89	15.82	15.27
SLC27A3	16.79	15.96	15.22	13.19	10.28	10.9	8.43	8.62
TINF2	10.66	11.02	10.78	11.27	10.44	10.9	10.82	10.51
RANBP10	11	10.86	10.79	10.27	10.71	10.91	10.66	11.69
TTC7B	10.65	11.7	11.23	10.97	12.86	10.91	11.54	12.4
RNF208	12.08	11.91	11.23	10.36	11.09	10.92	9.45	9.64
POLG	10.65	10.62	10.21	9.67	10.89	10.92	9.96	10.5
ACAA2	12.04	12.23	11.65	11.93	10.93	10.92	11.52	10.58
NUP88	10.65	10.03	11.62	10.87	12.25	10.92	13.28	11.86
MRPS36	10.6	9.83	10.13	10.65	14.64	10.92	14.39	12.94
POLR3K	10.97	8.93	9.71	9.25	9.02	10.94	9.33	9.51
CCDC28B	11.67	12.66	12.06	11.73	10.52	10.94	9.41	9.53
TTC27	10.06	8.83	10.12	10.19	11.45	10.94	13.03	12.18
SPC25	9.65	7.33	10.19	9.42	10.36	10.94	15.92	13.96
OSTM1	10.43	10.9	11.08	12.53	11.29	10.94	15.64	14.26
ZNF697	10.89	9.42	11.19	11.54	11.29	10.95	12.43	11.63
MERTK	9.42	8.73	9.7	10.9	11.93	10.95	13.73	12.18
RBM14	11.83	11.92	12.05	11.17	10.45	10.96	8.91	9.56
PISD	9.84	10.17	10.36	10.27	11.84	10.96	10.74	10.56
SNX11	11.19	12.74	10.39	10.92	11.48	10.96	10.33	10.86
CUL4A	9.64	9.32	9.96	10.88	10.81	10.96	13.52	11.74
ST3GAL5	9.46	10.36	10.96	10.46	11.51	10.96	11.74	11.84
SLIT3	9.41	9.39	8.64	9.3	10.31	10.97	10	10.14
CHPT1	10.49	10.15	11.06	11.82	10.32	10.97	11.43	11.03
EXOSC2	10.78	10.78	10.98	11.37	10.97	10.97	11.38	11.26
RSRC2	11.33	11.95	12.55	13.59	11.8	10.97	15.75	15.61
FAM219B	11.3	9.85	9.12	8.97	9.97	10.98	9.77	9.38
RAB31	9.48	9.55	10.14	10.14	10.69	10.98	12.56	11.38
ZNF76	9.88	9.31	9.02	8.9	10.57	10.99	8.96	9.76
HIST1H1C	13.4	13.07	11.76	12.79	10.9	10.99	6.94	10.97
TMEM175	9.25	10.89	10.97	11.06	10.88	10.99	10.87	11.33
GTF2B	10.57	11.2	10.76	12	11.46	10.99	15.15	13.15
ZNF688	11.73	11.83	10.06	10.54	10.81	11	9.73	9.94
TOR2A	12.36	13.59	10.31	10.57	13.02	11	8.84	10.21
GPATCH4	9.01	9.83	11.36	10.24	10.13	11	13.02	11.49

ST3GAL3	11.07	12.03	10.53	9.76	11.61	11.01	10.05	10.2
IRF2	11.12	11.3	11.74	11.96	11.1	11.01	14.27	10.95
TOR1AIP1	10.1	8.91	11.04	11.64	11.4	11.01	15.25	13.59
ERGIC2	10.55	11.69	10.7	10.82	11.66	11.01	16.02	13.79
ECT2	9.18	9.1	9.47	11.08	11.84	11.01	20.4	15.56
SYNE2	10.02	9.14	12.17	14.6	11.29	11.01	19.16	17.09
VPS11	9.72	11.17	9.59	10.13	10.61	11.02	9.83	9.38
BBS1	11.32	11.37	10.65	11.49	11.99	11.02	11.34	11.64
PDF	10.65	10.18	9.36	7.86	9.91	11.03	10.01	9.48
MYH10	11.05	10.23	11.51	11.96	11.22	11.03	12.06	12.05
USP16	9.07	9.76	9.57	10.22	10.33	11.03	15.63	14.3
RDX	9.82	9.08	11.39	13.97	10.96	11.03	17.3	16.33
PPP3CA	10.55	9.47	10.99	13.01	11.59	11.04	13.47	12.24
DCAF4	8.82	8.89	9.14	9.43	10.8	11.05	11.55	10.38
ARFGEF2	8.81	7.89	9.58	10.92	11.71	11.05	16.75	14.27
ICE1	9.89	8.42	10.91	12.59	10.4	11.05	16.71	14.68
CBX2	12.77	12.6	11.98	11.86	12.07	11.06	9.11	9.39
HSPA1A	12.52	14.7	10.04	11.86	9.68	11.06	7.41	10
ACVR1B	11.71	11.74	11.18	12.11	11.25	11.06	11.06	10.18
NUMBL	11.49	11.35	10.35	10.43	10.64	11.06	9.02	10.82
C10orf76	10.16	10.1	10.25	9.81	10.71	11.06	11.76	11.42
RFC5	11.88	12.45	11.53	12.36	13.18	11.06	12.78	12.41
PPP2R5E	10.31	9.95	11.82	11.27	10.92	11.06	13.41	12.73
CBWD2	9.34	8.74	11.27	10.73	12.24	11.06	17.76	15.73
ADCK5	11	10.4	10.96	9.65	9.28	11.07	8.68	9.19
PGP	8.59	9.34	8.47	7.8	9.83	11.08	8.85	8.8
PIP5K1C	10.95	11.39	10.32	10.72	9.68	11.08	8.45	9.54
ENO2	19.49	18.1	17.68	17	10.78	11.08	12.91	11.61
TCF19	10.58	10.49	10.81	10.28	10.76	11.09	10.82	9.37
UPP1	12.56	11.96	12.15	11.08	10.94	11.09	10.97	10.75
PATL1	10.89	11.69	12.83	12.12	11.09	11.09	13.35	13.38
MFN1	8.82	8.6	10.83	12.24	11.55	11.09	16.61	14.32
UBXN11	11.17	11.79	9.43	10.16	12.16	11.1	10.23	10.52
POLR3C	12.86	13.49	11.38	13.11	9.92	11.1	10.77	10.55
KNSTRN	7.59	9.03	8.58	8.4	11.25	11.1	12.96	11.23
RNASEH1	9.65	10.17	10.57	10.1	11.98	11.1	11.67	11.93
PLXNA3	11.07	11.55	10.65	11.91	10.45	11.11	10.53	10.88
CACNB3	12.4	12.49	12.28	10.91	10.27	11.11	11.16	10.98
TXNRD1	10.05	9.76	10.84	11.29	11.96	11.11	13.86	12.88
TRIM37	10.05	9.8	11.89	12.11	11.52	11.11	14.44	13.27
THBS3	11.05	11.85	9.93	11.25	10.84	11.12	11.29	10.65
RNASEH2B	9.62	10.27	11.8	11.37	12.22	11.12	13.04	12.47
ARHGEF12	9.86	9.36	11.42	12.81	10.85	11.12	14.51	13.2
GPATCH8	11.4	11.39	12.7	13.34	12.15	11.12	13.79	13.28
CASP9	11.2	10.44	10.68	9.9	10.32	11.13	10.12	8.47
STPG1	8.21	8.55	8.72	7.71	10.32	11.13	10.39	10.66
GPN1	12.81	12	11.84	12.22	11.85	11.13	12.39	11.65
SOGA1	9.87	10.18	10.11	10.11	11.3	11.13	10.75	11.84
POLG2	8.31	8.45	9.82	10.5	11.5	11.13	15.43	13.25
AGFG2	12.01	12.71	12.42	11.26	12.23	11.14	10.43	10.8
INTS4	10.19	8.89	9.3	9.61	10.28	11.14	11.75	10.93
ECD	10.65	10.77	12.44	11.03	12.08	11.14	14.59	12.37
TOPBP1	9.68	7.94	11.01	12.02	11.82	11.14	17.49	14.25
HAUS8	10.59	11.57	11.26	10.03	11.52	11.15	10.58	10.06
ILKAP	11.42	12.83	11.71	10.49	12.82	11.15	12.58	10.75
LOC105375355	10.06	10.94	11.78	12.44	11.55	11.15	12.86	11.94
PPIL2	11.55	11.16	10.42	10.68	12.52	11.15	11.68	12.26
MELK	11.37	9.77	12.77	11.55	12.2	11.15	14.5	12.46
INAFM1	9.88	10.3	10.96	9.62	8.01	11.16	6.06	7.22
NAT6	10.24	10.85	8.63	9.49	9.59	11.16	8.38	9.34
LOC100996720	14.96	13.97	15.83	14.41	12.17	11.16	10.15	9.77
CHAF1B	12.33	14.15	11.92	11.46	11.53	11.16	11.12	10.08
CDKN2C	13.52	12.47	12.58	11.7	10.67	11.16	9.13	10.39
C18orf8	9.09	9.72	9.64	10.02	10.14	11.17	11.9	11.9
GGPS1	10.35	8.92	11.33	12.42	10.89	11.17	14.96	13.93
FAR1	9.62	8.63	10.44	12.96	10.94	11.17	20.45	17.02
TMC7	8.75	9.61	9.3	10.13	11.25	11.18	12.54	11.29
AFAP1L2	10.27	10.16	11.01	10.72	11.22	11.18	11.21	12.5
KCTD9	10.18	8.89	10.92	12.59	12.63	11.18	16.39	14.96
DOLPP1	10.37	10.98	10.64	10.5	10.45	11.19	8.88	9.9
NDOR1	10.48	10.1	9.4	10.19	10.46	11.19	9.78	10.49
PHF20	10.94	9.75	12.06	12.93	11.76	11.19	13.99	13.5
SPC24	10.83	10.66	10.87	11.86	10.92	11.2	11.08	10.24
PIK3IP1	9.98	10.59	9.28	10.71	10.66	11.2	9.68	10.47
FAM220A	12.34	10.91	12.31	12.13	11.3	11.2	12.38	11.76
KYAT1	9.7	10.44	10.25	9.09	11.82	11.2	11.51	12.15
VPS37A	10.63	10.2	10.85	12.18	11.2	11.2	12.95	12.31
POGZ	11.72	11.51	12.28	13.36	11.6	11.2	13.73	13.19
COMMD9	10.74	10.18	9.78	9.09	12.75	11.21	12.16	10.74
ZDHHC18	12.27	12.35	13.1	11.89	11.88	11.21	10.38	10.82
FADS2	10.47	12.84	10.7	10.46	11.27	11.21	9.8	11.53
GTF2H1	10.54	9.28	10.3	10.78	12.31	11.21	15.92	13.82
RANBP2	8.66	8.52	11.83	13.8	10.99	11.21	19.74	17.05
OPLAH	11.02	10.85	10.95	9.75	9.46	11.22	8.32	9.17
WDR81	9.75	10.42	10.44	9.23	9.49	11.22	8.94	9.62

ACTR3B	9.77	8.95	9.58	9.76	12.79	11.22	10.89	10.49
AKAP8	10.06	9.53	9.54	10.85	11.07	11.22	11.84	11.45
HOXD9	8.42	8.1	9.1	10.78	9.57	11.22	11.24	12.06
INTS9	11.37	11.24	10.88	10.67	12.09	11.22	11.21	12.15
NUP153	11.15	10.72	12.19	12.32	11.16	11.22	14.12	12.77
SKA2	10.25	9.03	10.52	12.1	11.07	11.22	17.8	13.96
HSPA4L	8.32	7.97	10.21	10.86	10.86	11.22	18.34	14.33
LOC101929479	11.08	11.98	10.21	13.54	11.81	11.23	11.23	11.84
TTC31	12.24	12.72	11.58	12.07	12.56	11.23	13.42	13.34
ZC3H15	9.71	10.58	10.17	10.98	11.37	11.23	13.23	13.69
ESF1	10.16	11.43	10.87	11.84	12.1	11.23	15.37	15.26
VPS33A	9.8	10.5	10.04	9.05	10.79	11.24	11.7	10.38
SH3KBP1	11.6	11.76	12.29	12.95	11.54	11.24	11.81	11.02
HTT	10.65	11.07	10.6	10.42	10.97	11.24	10.63	11.13
TYW1	11.1	10.33	10.36	11.03	11.88	11.24	12.7	12.3
TOM1	10.68	10.81	11.13	9.3	9.8	11.25	8.92	9.63
PINX1	9.91	10.3	8.84	8.17	11.14	11.26	10.29	10.84
PPT2	11.04	11.02	11.24	10.97	11.04	11.26	10.98	11.6
RRN3	9.95	9.29	11.56	11.4	12.25	11.26	15.13	12.92
CDC42SE2	10.15	10.31	11.54	11.99	11.34	11.27	14.85	13.58
CUL3	10.74	9.62	10.45	12.28	12.45	11.27	15.64	14.99
TMEM159	10.07	10.75	10.62	10.91	11.41	11.28	10.08	9.99
IQSEC2	11.41	12.39	10.29	11.75	11.93	11.28	10.05	11.01
RUNDC1	12.59	12.05	11.99	12.27	11.97	11.29	11.03	11.7
STX8	11.07	11.13	11.94	10.9	11.86	11.29	12.08	12.66
CCNDBP1	10.84	9.56	10.39	10.62	12.35	11.3	14.18	12.08
STX12	10.78	10.91	10.84	10.77	12.41	11.3	14.04	12.99
FAM45A	8.84	8.97	10.2	11.21	11.63	11.3	16.28	14.35
TAF9B	10.52	9.13	10.82	11.6	11.64	11.31	16.22	12.91
KRR1	9.01	9.1	9.8	9.56	11.3	11.31	17.5	14.51
KCTD3	11.4	10.81	13.12	14.05	12.26	11.31	18.03	14.94
GPT2	12.77	12.34	11.97	11.49	11.77	11.32	9.69	10.53
POLR3A	10.68	11.01	11.2	10.62	11.37	11.32	11.8	11.01
FAM214B	10.86	11.11	10.16	11.66	10.64	11.32	10.26	11.66
GNB4	9.08	8.71	10.88	13.89	11.39	11.32	15.93	13.71
PFAS	9.51	10.1	9.37	9.65	10.7	11.33	9.89	10.16
SLC15A4	11.27	10.74	12.21	11.67	11.08	11.33	11.98	10.38
MMGT1	10	9.97	10.29	11.46	10.74	11.33	14.9	12.02
GTF2H2C	10.91	8.75	10.4	13.33	12.45	11.33	18.28	16.13
RAB40B	10.37	9.57	9.22	9.93	10.65	11.34	11.19	11.02
C14orf80	12.14	11.26	11.23	9.47	10.93	11.35	8.5	8.51
CDC16	10.08	10.69	12.28	12.4	13.74	11.35	15.6	14.53
HLTF	10.01	10.17	11.96	13.68	12.6	11.35	19.76	17.04
LRCH3	10.32	10.68	11.06	11.02	9.94	11.37	10.63	10.83
RNF25	12.25	11.31	11.92	11.7	11.16	11.37	10.06	11.14
MECOM	11.25	10.2	13.84	15.44	11.64	11.37	16.94	15.64
RASSF1	7.96	9.4	8.23	7.69	10.13	11.38	8.91	8.76
MTHFR	11.81	12.05	11.36	11.31	10.76	11.38	8.84	10.89
TNS2	13.65	12.84	13.18	13.16	10.41	11.38	9.83	10.98
STK11IP	11.83	12.36	10.66	10.39	12.12	11.39	10	9.52
TATDN2	11.11	10.72	10.74	11.16	10.9	11.39	10.75	10.84
TMEM126B	10.01	10.77	11	11.72	12.59	11.39	18.02	15.15
SLC30A9	11	11.42	12.1	13.71	13	11.39	18.6	15.63
YTHDC1	11.78	10.48	12.92	14.51	12.73	11.39	16.69	15.94
NUP107	9.93	8.97	10.96	13.32	12	11.39	18.81	16.03
SPSB1	12.24	13.08	12.19	11.71	11.56	11.4	11.21	10.31
SYNPO	13.89	14.44	13.29	13.16	12.04	11.4	10.84	10.6
TNFAIP8L1	9.43	10.55	8.85	9.44	10.75	11.4	10.14	11.05
CYB5D2	10.64	11.69	11.17	12.39	11.19	11.4	9.61	11.48
CCNB2	10.15	10.22	10.73	10.06	11.74	11.4	12.39	12.06
CNOT2	10.24	10.27	11.09	11.35	11.66	11.4	13.77	12.66
HSPA13	12.36	11.04	11.87	13.8	13.37	11.4	19.99	15.91
ATF5	15.08	14.38	15.22	14.94	11.46	11.41	9	10.4
ENY2	10.79	11.82	11.29	12.99	12.2	11.41	14.39	12.61
PLA2G15	11.43	12.16	10.97	11.24	11.01	11.42	10.52	10.69
RNF149	9.98	9.72	10.07	10.82	11.37	11.42	12.56	11.97
CNOT10	10.65	10.83	11.25	11.71	12.81	11.42	15.25	12.41
GEMIN6	9.99	8.85	8.49	9.78	10.39	11.43	11.13	12.63
IFT140	12.32	11.36	11.61	11.12	10.17	11.44	9.32	10.79
RNF34	11.2	11.18	11.88	12.13	12.03	11.44	11.75	11.77
FAM114A1	12.6	13.11	13.46	14.32	11.96	11.44	14.17	12.89
GPANK1	10.85	12.55	11.06	10.01	11.99	11.45	9.64	9.83
SGCE	12.99	12.85	15.08	14.57	11.72	11.45	16.93	14.55
ABCD3	10.88	11.2	13.28	14.2	14.09	11.45	18.9	16.69
GOLGA3	11.4	12	12.32	12.04	12.43	11.46	12.29	12.81
RIC1	10.03	9.31	11.87	12.81	11.76	11.46	15.46	13.51
CYBRD1	11.75	11.16	13.01	13.98	12.05	11.46	15.85	14.49
RBM4B	10.59	10.39	12.55	11.33	12.49	11.47	12.87	11.51
PLD2	10.99	11.21	10.9	11.47	11.8	11.47	10.91	11.83
CCDC92	11.88	12.95	13.2	13.02	9.25	11.48	9.18	10.79
CDC6	14.3	12.49	13.88	14.39	12.97	11.48	13.37	12.18
SCHIP1	10.08	9.91	11.29	13.53	11.13	11.48	13.98	14
CEP55	8.55	7.51	9.18	9.95	11.19	11.48	16.63	14.08
FAM107B	10.91	10.23	11.89	12.6	13.35	11.48	15.99	14.26

RRP8	12.24	11.76	11.84	11.17	12.01	11.49	10.42	9.04
SNTA1	11.51	13.04	12.4	10.17	10.45	11.49	7.78	9.07
ZNF865	11.88	10.77	11.54	11.08	11.19	11.49	9.43	10.26
PCID2	9.13	10.01	10.12	10.5	11.29	11.49	12.34	10.66
KLHDC10	12.1	11.63	11.3	12.59	12.86	11.49	13.95	12.99
STAU2	11.87	11.07	12.86	13.59	12.04	11.49	16.68	14.29
NAB2	12.99	13.55	13.22	11.99	11.13	11.5	10.04	9.95
CCDC159	10.77	10.31	9.42	10.15	12.09	11.5	10.4	11.18
APPL2	11.28	10.68	11.02	10.81	13.03	11.51	14.01	13.31
COPS7B	12.94	13.4	12.63	13.4	11.96	11.52	12.5	11.66
ANKHD1	12.25	11.59	12.13	12.36	13.95	11.52	14.8	12.18
GFPT1	11.12	11.27	12.18	13.01	12.04	11.52	15.4	13.61
MFSD6	10.89	10.04	11.75	12.98	12.99	11.52	14.97	14
RCOR3	10.81	10.13	12.56	14.01	12.45	11.52	15.71	15.38
POLA2	13.61	13.97	13.01	11.99	12.73	11.53	12.35	12.04
ING5	9.51	10.02	10.07	10.37	12.02	11.53	13.26	12.25
PDIA5	11.93	14.2	11.92	12.69	12.14	11.53	14.02	13.26
ADAM10	11.01	10.27	13.19	15.47	12.27	11.53	20.09	17.32
ANKRD54	11.65	10.31	10.5	11.45	10.52	11.54	9.53	10.21
OAS1	33.92	36.47	35.7	35.42	11.07	11.54	11.22	10.35
SCAF4	11.89	12.67	12.33	12.45	10.96	11.55	12.72	12.31
C12orf45	9.69	8.71	10.03	10.07	12.28	11.55	10.82	12.43
ZNF775	12.67	12.57	12.72	11.88	10.09	11.56	8.17	8.74
NOM1	9.3	9.42	10.18	10.86	11.26	11.56	12.72	11.51
MTRNR2L2	12.28	11.82	11.75	13.31	12.82	11.56	13.66	13.24
ARNT	11.67	11.99	12.17	12.41	12.03	11.57	13.28	12.79
PPID	10.55	9.71	10.54	13.07	13.13	11.57	14.43	13.33
USP1	11.26	11.43	11.21	12.92	12.3	11.57	16.16	14.64
RAB43	14.01	13.81	13.03	12.85	8.58	11.58	7.43	8.69
OAF	8.76	9.59	8.49	8.09	11.23	11.58	8.4	9.55
TNKS	11.51	10.8	12.31	12.83	11.62	11.58	14.42	13.52
SECISBP2	10.64	9.24	11.4	11.92	12.01	11.58	14.77	13.8
UBLCP1	9.44	8.5	10.85	13.08	11.51	11.58	19.82	17.35
POT1	10.75	12.02	10.7	12.78	10.18	11.6	16.38	12.99
RAB12	9.27	8.83	9.27	9.72	11.07	11.61	12.17	10.95
KPNA1	10.42	9.37	10.44	10.2	10.97	11.61	14.41	12.27
LRRC37A3	11.27	10.4	12.14	13.18	11.58	11.61	15.86	14.26
ANKS6	10.69	10.74	10.56	10.73	12.38	11.62	12.07	10.85
CUL9	12.19	11.49	12.18	11.1	12.01	11.62	11.08	11.45
PGAM2	7.96	8.19	9.21	11.78	10.97	11.62	14.94	11.87
LRRFIP2	9.19	8.04	11.07	11.05	13.85	11.62	15.07	13.65
WDR12	10.14	9.13	9.55	10.85	13.23	11.62	14.88	13.82
F2RL1	12.81	11.09	12.78	13.04	12.58	11.63	15.4	12.61
SPRY4	11.78	12.71	12.39	12.8	11.2	11.64	11.34	12.31
RBBP6	11.83	11.07	12.67	13.34	10.31	11.64	13.05	12.85
PTGES	25.78	23.67	22.78	21.48	10.77	11.66	9.08	10.53
TMEM206	11.42	11.05	10.17	12.1	12.3	11.66	12.09	11.91
ANKRD27	10.39	10.43	10.35	12.15	12.18	11.66	12.37	12.72
DDHD2	10.84	10.28	11.39	12.02	12.48	11.66	14.42	14.4
CENPF	8.14	7.45	10.24	11.12	11.14	11.66	19.03	17.27
CORO1A	15.38	15.52	14.71	12.99	12.86	11.67	8.74	11.26
MRPL54	11.21	12.69	10.82	9.64	11.74	11.68	7.73	9.74
NLN	10.41	9.33	11.72	12.59	12.35	11.68	16.39	14.57
SSR1	11.1	11.2	12.52	13.86	13.01	11.68	17.69	15.51
DOLK	11.38	11.99	11.35	11.13	12.81	11.69	8.63	10.94
MAN2C1	13.12	12.95	12.32	11.09	11.07	11.69	11.18	11.28
FAM193B	11.15	10.75	11.46	11.42	12.06	11.69	12.63	12.49
RBMS1	11.31	10.36	12.29	14.9	11.99	11.69	17.55	16.02
CPSF6	12.28	11.23	12.36	13.42	12.56	11.7	14.94	14.03
RBM17	11.39	11.63	11.97	13.11	11.72	11.71	14.39	12.94
ABHD15	10.74	10.26	10.15	10.34	11.2	11.72	8.89	10.07
HIRA	11.4	11.29	11.14	11.58	11.75	11.72	10.86	11.36
SMG1	10.49	9.75	12.52	14.4	11.77	11.72	19.71	17.15
KMT2E	11.89	11.33	13.86	17.96	12.09	11.72	18.36	18.33
LMAN2L	12.27	12.97	12.15	11.98	11.75	11.73	11.76	11.79
PLPP6	10.31	9.98	10.87	10.82	12.13	11.73	11.99	12.15
RFC1	10.42	9.08	12.09	13.75	12.4	11.73	17.55	15.44
TONSL	12.19	11.32	12.32	11.61	11.67	11.74	10.3	9.97
OLFML2A	14.94	15.48	14.69	15.44	12.27	11.74	10.3	11.6
GPD1L	9.66	9.57	10.56	10.71	12.28	11.74	13.99	13.63
SNX8	10.87	10.33	11	9.92	10.36	11.75	8.71	9.31
PNP	9.81	9.98	10.08	9.56	12.28	11.75	11.95	11.09
MTSS1	14.11	14.92	15.28	16.07	10.53	11.76	12.18	13.88
GFM1	9.93	9.46	11.94	13.01	13.33	11.76	16.16	14.84
GFOD2	11.59	11.24	11.54	11.11	12.02	11.77	10.74	11.04
BAG1	10.35	10.75	10.32	8.94	12.69	11.77	11.04	11.62
GBE1	11.95	11.44	12.43	13.02	11.98	11.78	14.11	11.37
CNOT6	9.58	8.76	11.07	12.54	11.99	11.78	16.01	14.62
SGSM2	10.98	11.48	11.67	11.27	12.09	11.8	11.54	11.33
EDC3	13.27	14.02	13.38	12.82	12.53	11.8	12.33	11.96
TAF1C	10.49	10.46	9.94	10.04	11.66	11.8	11.99	13.29
FOPNL	10.61	9.25	10.83	12.1	13.24	11.8	18.55	14.18
MICU2	10.91	10.02	11.13	12.4	12.47	11.8	18.21	15.87
TP53INP2	11.53	12.2	11.81	12.43	11.75	11.81	10.64	12.1
GMPPB	11.18	10.99	10.94	10.17	10.36	11.82	8.22	8.86
HSBP1L1	13.04	12.73	11.28	13.95	13.27	11.82	14.33	12.89
SOAT1	11.16	10.85	11.91	12.67	12.03	11.83	15.85	13

TBC1D8	11.44	11.61	12.5	12.57	11.79	11.83	13.63	13.49
KDM3A	18.56	16.27	18.32	19.99	11.4	11.83	16.42	14.63
ARVCF	10.35	10.39	10.34	9.93	11.08	11.84	10.04	10.35
SRRD	9.55	9.09	9.2	8.95	11.33	11.84	10.97	10.73
TMEM92	12.84	14.01	15.05	11.77	11.18	11.84	8.8	10.85
ERCC2	12.75	10.76	11.19	11.1	11.82	11.84	10.41	11.46
EID2	9.98	9.39	11.36	10.28	11.62	11.85	10.73	10.57
TNFRSF21	13.03	13.42	13.05	13.14	10.66	11.85	10.14	11.01
SLC35E3	10.71	9.46	10.8	10.51	11.87	11.85	10.81	11.31
GOPC	9.16	9.27	11.34	13.55	12.11	11.85	19.54	16.53
44264	12.63	12.42	10.85	11.4	12.52	11.87	10.32	11.04
RANBP9	11.1	10.61	11.46	13.33	10.75	11.87	13.62	13
SP1	11.3	10.36	11.44	12.3	11.96	11.87	14.43	13.28
NAB1	9.86	9.59	11.07	13.41	11.63	11.87	18.14	15.67
TFF3	13.79	12.67	11.87	10.05	11.34	11.89	10.28	9.29
CBSL	8.28	11.71	15.79	13.49	6.1	11.89	8.07	9.83
SMG9	9.6	9.67	10.34	9.48	12.16	11.89	10.94	10.75
PTPN9	11.37	12.2	12.12	11.18	11.36	11.89	11.36	11.52
KIAA0391	11.29	10.96	11.42	11.4	12.82	11.89	14.19	13.08
AK4	13.52	12.83	13.74	15.04	12.14	11.89	14.98	13.83
PPARD	11.06	10.95	12.48	11.37	10.86	11.9	11.01	11.06
FAM103A1	11.25	11.94	11.48	14.99	12.9	11.9	14.55	15.24
SEC62	11.45	11.72	12.66	14.96	12.69	11.9	20.21	17.3
ANP32E	10.98	10.27	13.07	15.15	12.8	11.9	22.72	18.96
INO80C	11.16	12.4	10.88	12.93	13.88	11.91	11.86	11.57
SMAD3	10.46	10.58	11.01	11.12	11.87	11.91	11.89	11.6
RNF113A	14.84	13.47	14.37	12.83	12.96	11.91	10.76	11.61
TPD52	11.65	10.97	12.72	13.12	11.83	11.92	14.93	12.64
CBARP	10.76	10.56	9.46	8.91	10.31	11.93	7.84	8.89
DCAF11	11.85	12.02	11.75	11.69	13.26	11.93	11.05	12.59
SENP2	9.93	9.12	11.58	11.77	12.24	11.93	15.24	14.47
ARV1	10.02	11.01	11.21	11.84	12.51	11.93	16.2	14.66
DYRK3	10.04	9.5	10.4	10.06	12.09	11.94	11.82	10.68
EP300	12.86	12.2	12.91	14.16	12.31	11.94	13.54	13.69
LRRC20	11.57	12.4	11.89	10.78	11.89	11.95	9.02	10.62
TAF4	11.35	10.36	11.07	11.51	10.78	11.95	11.65	11.49
CDK5RAP2	10.99	10.14	12.31	12.96	12.09	11.95	14.43	13.07
RWDD2B	9.57	9.95	10.89	11.08	12.08	11.95	15.29	13.15
RAB3A	10.39	11.52	10.03	8.98	9.58	11.96	7.8	10.59
NEDD9	14.96	15.18	14.78	16.18	12.16	11.96	12.8	12.6
CCNE1	13.78	12.46	12.22	12.75	13.11	11.96	12.72	13.09
MXD3	8.5	9.32	9.99	8.29	12.17	11.97	9.73	9.18
MRPS11	12.12	10.98	11.46	10.04	12.85	11.97	10.34	12.15
ARHGEF11	12.77	11.83	13.05	12.81	11.23	11.97	12.24	12.19
ZNF384	12.81	12.34	12.73	13.13	11.9	11.97	13.26	13.52
ALG5	12.31	12.16	13.6	11.89	13.87	11.97	13.98	14.63
LOC107983993	11.05	10.76	10.21	11.52	11.54	11.97	17.98	15.62
GCLC	9.89	9.29	10.53	12.46	12.84	11.97	17.23	16.55
WEE1	10.76	10.4	11.24	11.05	11.94	11.98	12.59	11.8
PARP12	18.56	17.36	18.2	18.46	12.45	11.98	12.99	11.94
CDK6	9.98	9.26	11.47	15.86	11.99	11.98	21.48	18.8
CYTH3	9.27	10.11	10.14	10.33	11.94	11.99	11.98	11.31
CCDC71	12.87	12.83	12.44	12.03	12.99	11.99	9.03	11.73
KLHL9	11.28	10.78	12.88	13.29	12.58	11.99	15.69	14.54
NAA35	11.55	10.34	12.31	13.08	12.95	11.99	16.33	15.33
CASKIN2	13.04	12.82	12.43	11.81	11.21	12	9.03	9.22
RCOR2	20.46	18.43	18.45	17.82	11.71	12	9.94	10.95
RANGRF	12.26	12.84	11.53	12.17	12.32	12	13.5	11.31
HMG20A	12.22	11.98	11.91	12.47	12.62	12	13.37	13.73
PSMB9	16.72	16.98	16.46	15.79	14.41	12.01	11.48	11.54
C7orf43	10.71	10.5	10.66	11.17	11.92	12.01	10.87	11.87
AUH	11.2	7.94	12.42	12.16	11.96	12.01	17.36	13.6
SNX21	11.32	9.84	10.72	9.85	10.19	12.02	11.5	9.96
PDCD2L	10.35	10.79	11.04	9.16	12.34	12.02	9.99	10.04
FITM2	10.95	10.96	9.61	9.23	11.89	12.02	8.13	10.86
LOC101060022	9.93	9.01	10.42	12.6	10.37	12.02	14.29	13.79
ZNF32	12.72	10.36	11.46	11.73	9.94	12.03	11.91	10.62
ARSD	13.12	13.59	12.65	12.96	11.75	12.03	11.49	11.41
RNF216	13.65	13.27	13.71	13.17	11.59	12.03	11.38	12.23
GPC3	20.12	23.99	19.9	18.8	12.29	12.03	11.89	13.32
ME2	11.09	10.43	11.11	11.39	13.34	12.03	15.9	13.37
KIAA0907	12.77	10.62	12.24	13.86	11.72	12.03	17.94	14.54
ZBTB8OS	9.44	9.6	10.34	11.11	11.63	12.04	14.66	13.15
PRSS33	11.16	13.59	11.41	10.31	8.66	12.05	8.52	10.02
PPFIBP1	12.8	11.32	13.83	15.03	12.84	12.05	17.14	15.8
DSCR3	11.75	11.97	12.04	12.47	12.03	12.06	13.94	12.96
RTF1	11.68	11.33	12.71	13.22	13.12	12.06	13.5	13.78
FAM58A	11.32	11.04	10.57	9.66	10.02	12.07	9.54	9.1
TRMT61A	9.92	9.83	10.05	8.91	11.97	12.07	9.58	9.24
ARMC7	11.54	12.9	11.96	11.04	11.56	12.07	10.11	9.99
FLCN	11.7	11.91	11.66	11.1	11.75	12.07	12.11	11.02
MAB21L1	10.21	8.68	10.08	10.87	11.29	12.07	12.02	11.12
TSR1	11.51	10.85	11.11	12.2	13.67	12.07	13.88	12.55
C6orf226	12.47	12.04	12.58	8.45	10.32	12.08	8.82	9.44

ALKBH4	11.72	11.52	10.93	10.09	10.76	12.08	9.69	9.9
PHC1	11.17	10.86	10.77	11.59	12.12	12.08	12.57	11.96
UNK	12.72	12.14	12.27	12.06	11.81	12.09	10.65	11.25
MAML1	11.78	11.38	12.77	12.16	11.48	12.09	11.85	11.44
URB1	10.65	10.54	10.71	10.54	11.96	12.09	11.24	11.8
CUL4B	11.1	10.54	12.88	14.01	12.47	12.09	19.38	15.83
KIAA1429	12.39	11.2	13.97	13.84	14.37	12.09	18.07	15.97
FOXRED1	9.84	10.45	10.28	9.76	12.34	12.1	11.59	10.38
FAHD2B	13.26	14.79	12.62	12.83	12.15	12.1	9.92	11.77
MAD2L1BP	13.97	14.19	15.03	13.85	12.24	12.1	13.39	13.42
CRK	11.27	10.31	11.56	13.49	11.9	12.11	16.42	13.44
SLC27A4	10.06	10.66	9.9	9.23	10.93	12.12	8.88	10.07
KYAT3	10.83	11.2	11.74	12.05	12.5	12.12	15.34	13.04
PHKG2	10.96	11.9	11.63	10.05	11.33	12.13	10.86	11.42
BRD1	12.82	12.01	13.4	12.72	11.84	12.13	12.91	12.63
PHTF1	10.68	10.97	11.43	13.05	11.65	12.13	13.38	13.92
NELL2	9.44	10.42	10.63	11.2	12.34	12.13	14.98	14.09
CKAP2	11.52	10.03	11.8	13.59	12.29	12.13	18.9	16.04
REEP6	13.67	14.22	12.27	12.03	12.19	12.14	8.34	8.94
ATXN2	10.77	10.16	11.16	12.13	10.79	12.14	12.38	12.09
MED12	12.27	13.27	11.69	11.58	12.03	12.14	12.19	12.69
OCRL	13.38	12.36	13.86	13.41	12.16	12.14	14.28	13.13
KIF1BP	11.21	10.96	11.67	12.72	12.16	12.14	13.99	13.23
DLL1	12.29	11.54	11.27	11.74	12.34	12.15	11.03	10.99
DHDDS	12.48	12.92	11.69	11.45	12.93	12.15	10.51	11.32
NBEAL2	10.2	10.47	11.29	10.4	11.12	12.15	11.01	11.64
TRAFD1	13.32	13.12	12.93	12.15	14.22	12.15	11.37	11.66
MAN2A1	11.63	10.45	13	15.55	11.64	12.15	16.57	14.88
ADAM17	12.3	12.31	14.28	13.86	12.78	12.15	15.3	15.14
FAM136A	9.59	9.03	10.65	11.25	10.34	12.16	14.57	11.48
DERA	12.9	12.75	12.7	13.22	11.93	12.16	16.23	11.81
BCL10	11.23	9.84	12.19	12.92	12.83	12.16	16.08	13.85
DECR2	11.84	10.28	10.84	10.19	11.99	12.17	10.36	9.25
GSTO2	12.59	12.1	12.99	12.23	13.18	12.17	11.62	12.54
KIF1B	11.77	11.91	12.4	12.99	12.26	12.17	15.5	14.44
CCNL1	12.27	12.3	12.72	15.73	12.72	12.17	23.02	19.47
ZNF524	11.64	10.81	12.18	9.99	9.59	12.18	6.96	6.95
NDRG4	12.13	11.76	12.42	11.76	12.2	12.18	11.71	11.26
TRIM29	11.76	13.34	12.11	11.64	12.74	12.18	10.72	11.73
COL5A1	17.4	17.78	17.27	18.49	10.85	12.18	11.68	12.86
PHACTR4	12.69	13.15	13.23	14.1	13.15	12.18	14.37	14.13
SMC3	11.38	9.76	13.2	15.58	13.03	12.18	22.6	19.5
RCCD1	10.49	10.34	11.1	9.06	11.64	12.2	9.81	9.69
VPS18	12.06	12.45	11.8	10.27	12.3	12.2	9.92	10.97
TIMM10B	10.63	11.69	11.31	12.02	12.07	12.2	13.99	14.35
SPTSSA	9.57	10.28	10.26	11.8	13.07	12.2	16.62	14.91
CAPN10	11.26	10.55	10.61	9.94	11.16	12.21	9.86	11.11
KMT2B	10.82	11.69	10.78	11.02	11.95	12.21	10.89	11.74
USP33	11.93	10.91	13.71	14.73	12.41	12.21	20.31	17.37
SOX21	10.89	10.43	10.3	10.52	10.45	12.22	10.62	10.87
APITD1-CORT	11.21	12.82	11.39	12.24	12.82	12.22	12.53	11.27
DOCK1	11.01	10.21	11.94	13.41	12.15	12.22	15.68	14.16
FAM212A	14.29	12.6	12.12	10.42	11.27	12.23	9.48	9.41
LOXL1	18.34	18.82	17.42	16.27	10.94	12.23	8.97	9.42
LRFN3	13.2	13.04	13.48	11.9	11.12	12.23	8.9	10.78
DNAJC9	13.18	11.33	13.2	13.74	13.24	12.23	14.02	12.37
DUSP12	13.25	13.35	12.47	13.75	13.14	12.23	15.7	12.9
VAMP8	15.99	15.68	14.37	13.11	15.23	12.23	13.54	13.95
AMOTL2	12.86	11.48	12.71	12.43	13.47	12.24	12.37	11.29
NEPRO	9.4	10.8	10.06	12.92	13.02	12.24	17.95	14.71
PUS7	10.53	10.92	12.05	14.31	13.28	12.24	18.31	15.18
TAF13	8.54	9.08	10.5	11.91	10.95	12.24	16.93	18.42
C1orf174	10.42	11.17	11.56	12.68	12.16	12.25	13.77	13.85
IL6ST	10.54	10.57	12.61	15.15	11.83	12.25	20.54	18.25
MED14	11.57	10.77	12.63	13.17	12.87	12.26	15.9	14.37
44262	11.48	11.98	12.87	15.13	11.6	12.26	21.01	17.69
HAUS7	12.61	13.09	11.48	10.81	12.61	12.27	10.42	11.72
SS18	10.83	8.78	12.25	14.28	11.15	12.27	19.09	15.35
RHOBTB2	13.78	14.72	13.99	13.42	12.33	12.28	10.41	11.63
PDXK	12.92	12.82	12.83	12.29	12.2	12.28	10.59	11.77
RBM14-RBM4	14.22	11.11	11.57	10.9	9.44	12.28	10.58	12.1
OARD1	10.14	11.06	11.15	13.92	13.47	12.28	14.75	14.66
WASL	12.05	10.95	13.05	14.32	13.52	12.28	17.76	16.84
ASAP1	10.43	9.88	11.39	13.62	11.77	12.29	16.37	15.15
OPA1	8.99	8.63	11.08	12.87	13.07	12.29	21.66	18.12
RTKN	12.53	12.73	12.36	11.53	11.5	12.3	8.74	10.19
LENG1	11.58	12.94	11.7	11.03	11.63	12.3	9.74	11.12
XRCC1	13.24	12.42	12.21	11.11	10.89	12.31	10.27	11.06
WBP1L	11.99	12.7	11.84	11.61	12.33	12.31	11.03	12.83
NOL10	10.6	8.89	11.25	12.01	13.18	12.31	15.72	13.76
VTA1	10.49	11.34	11.81	12.8	12.37	12.31	17.64	15.3
WDR33	12.34	11.84	12.64	12.84	13.27	12.32	13.94	12.89
GTF2A2	13.14	14.6	12.21	15.87	14.02	12.32	17.35	16.73
GINS1	12.98	11.89	12.53	11.93	12.75	12.33	14.42	11.74
WWP1	10.96	9.83	11.9	13.03	12.74	12.33	17.62	15.44
AGK	13.54	12.93	13.92	15.17	12.24	12.33	16.05	15.73

ABHD16B	15.44	12.75	12.31	12.24	11.56	12.34	8.96	9.47
MRPS5	10.34	11.34	11.07	10.83	13.49	12.34	14.09	13.29
SUPT20H	12.37	11.78	13.57	15.18	12.15	12.35	17.43	16.59
BCL7A	11.78	11.98	11.1	11.35	13.06	12.36	11.67	11.75
ARPP19	10.04	9.94	12.03	14.3	13.11	12.36	19.42	17.72
HMBS	12.34	13.62	12.63	10.64	12.29	12.37	9.22	10.89
TFIP11	12.11	10.39	12.38	11.41	11.96	12.37	11.08	11
PGPEP1	10.92	11.8	10.58	11.47	12.29	12.37	11.54	11.15
PVR	11.87	11.66	11.41	11.06	11.98	12.37	11.43	11.19
NBN	12.17	11.12	11.54	12.75	12	12.37	19.22	16.33
IL17RC	13.96	15.06	13.72	13.17	12.17	12.38	10.47	11.21
SP2	12.03	11.48	12.27	12.33	12.9	12.38	11.29	12.49
MYO1D	13.63	13.21	12.77	12.87	13.26	12.38	12.9	12.91
TRA2A	13.39	13.32	14.97	16.8	12.76	12.38	20.79	18.35
LRPAP1	11.71	11.86	11.47	11.06	11.72	12.39	9.75	10.96
CCDC51	13.53	12.05	13.04	10.77	13.78	12.39	11.63	12.35
NFIB	10.45	10.39	12.28	14.52	12.75	12.39	16.84	15.38
RPAP1	12.06	12.71	11.29	11.2	12.45	12.4	10.78	11.81
ORC6	10.14	9.72	11.06	12.54	11.94	12.4	15.11	12.35
CEP68	11.88	11.4	13.97	12.66	12.93	12.4	12.65	13.07
SDC2	13.92	13.24	14.63	13.87	12.45	12.4	13.59	13.57
KBTBD2	12.17	10.34	12.49	11.6	11.95	12.4	17.01	13.68
POLH	11.26	10.67	11.05	11.59	13.11	12.4	14.27	13.87
PIGN	11.54	11.13	12.79	12.91	12.69	12.4	17.26	15.21
FEM1A	11.74	11.85	11.56	10.83	11.54	12.41	9.84	10.98
CHCHD4	9.36	8.4	8.79	9.52	12.14	12.41	11.66	11.33
LOC100652901	11.08	10.01	12.75	15.66	11.29	12.41	20.4	19.29
TPST2	10.83	11.71	12.04	11.92	12.11	12.43	10.7	11.48
SGPL1	11.69	12.12	11.8	13.06	12.28	12.44	12.73	13.72
LIG3	10.55	11.8	11.11	11.7	12.25	12.45	12.91	12.58
FIP1L1	13.45	12.86	14.01	14.76	13.75	12.45	16.42	15.45
TMEM39A	11.3	12.34	12.99	15.15	12.14	12.46	14.4	13.36
CXCL6	12.92	12.67	13.64	13.54	14.76	12.46	16.85	15.65
IFI44L	25.83	24.48	30.49	36.65	11.98	12.46	21.06	16.3
RBM25	11.23	11.26	12.68	15.32	12.69	12.46	19.14	18.47
RGS14	13.18	13.09	13.4	11.96	11.88	12.47	9.12	10.5
ARID5A	16.31	15.85	16.81	14.33	11.96	12.47	9.55	11.84
CHMP4A	12.09	12.86	12.47	12.96	12.63	12.47	12.84	13.62
SLCO3A1	10.44	10.48	11.05	9.94	10.48	12.49	10.67	11.32
PPRC1	12.66	11.66	11.86	11.96	12.72	12.49	12.23	11.77
INPP5A	11.63	11.91	11.7	12.23	11.66	12.5	12.14	11.51
RPE	11.18	11.01	13.04	13.12	12.36	12.5	17.9	15.83
GTPBP1	13.57	14.06	13.21	13.5	12.87	12.51	10.46	12.46
PLAGL2	12.6	13.29	14.34	13.33	11.88	12.52	12.69	13.26
ACSF3	12.62	12.29	12.17	11.6	11.65	12.53	10.83	11.08
C17orf58	14.38	10.64	12.45	12.98	11.66	12.53	14.43	12.2
ANKRD40	12.67	12.87	13.69	14.75	13.07	12.53	15.1	15.58
ZFPM1	11.94	11.78	11.51	10.79	10.48	12.54	7.62	7.71
RBM28	10.09	9.68	11.4	10.85	12.92	12.54	15.39	14.32
NBPF15	10.67	10.43	11.16	14.77	13.22	12.54	17.53	17.48
ENTHD2	10.16	10.01	10.11	10.95	12.48	12.55	12.82	11.85
CTNNAL1	11.36	11.16	12.98	12.91	12.89	12.55	15.6	13.42
MTMR4	14.22	13.93	13.77	14.24	13.14	12.56	14.08	13.63
PIK3CB	11.15	10.81	12.26	12.54	12.65	12.56	15.93	15.15
PPIL3	12.54	11.38	12.82	13.41	13.9	12.56	17.81	15.97
RB1	10.33	10.1	12.05	13.92	12.48	12.56	23	17.29
DNM1	13.77	13.34	13.6	13.58	11.49	12.57	10.06	12.01
MAP3K10	12.07	11.64	12.11	10.88	12.33	12.58	9.51	10.35
UBE2K	11.63	10.84	13.34	13.73	13.17	12.59	18.98	15.07
UHRF2	11.53	10.9	13.43	14.94	13.58	12.59	18.22	16.03
LPGAT1	10.26	10.47	12.14	16.06	13.15	12.59	18.94	16.41
WHSC1L1	11.73	10.59	12.05	13.5	12.55	12.61	16.83	14.6
ADGRA3	11.67	11.06	12.6	14.04	12.46	12.61	15.37	15
MUTYH	15.02	12.18	11.76	11.12	11.21	12.62	11.04	10.82
RNF185	11.95	12.84	11.66	11.94	12.22	12.62	11.91	11.75
EXOC2	12.5	11.06	13.35	13.99	13.29	12.62	16.43	15.35
KLHL22	12.45	12.55	13.08	12.04	11.13	12.63	9.53	10.05
SLC25A44	12.54	12.69	11.74	12.58	13.17	12.63	12.38	14.23
NT5M	13.26	11.26	12.52	11.48	12.55	12.64	10.44	10.73
MED30	12.92	12.69	14.91	12.92	12.34	12.64	13.83	11.12
ZFAND2B	11.68	12.16	12.13	12.96	11.28	12.64	11.36	11.41
TMEM68	8.94	10.59	11.99	12.26	14.82	12.64	19.26	17.19
LSP1	32.97	27.7	30.46	25.32	10.81	12.65	9.94	8.95
VKORC1L1	12.83	11.33	12.81	13.25	12.93	12.65	14.87	13.4
SLC7A1	12.45	11.84	11.68	12.12	11.93	12.66	11.58	12.11
FKBP15	14.17	13.81	14.03	14.1	12.58	12.66	14.1	13.35
PPP1R26	12.02	11.74	12.21	11.87	12.54	12.67	11	11.09
TTC9C	11.71	12.53	11.24	12.51	11.39	12.68	13.63	12.68
RAB5A	11.77	11.23	14.48	16.13	13.42	12.68	18.58	16.84
NF1	11.26	10.84	14.09	15.01	13	12.68	18.49	17.11
IFT20	10.26	10.61	11.1	10.59	12.29	12.69	14.72	12.67
TMLHE	13.36	12.54	13.35	13.36	13.15	12.69	13.54	14.95
MAGEA11	12.81	12.4	11.5	12.36	12.97	12.7	14.02	13.3
NUP155	12.73	11.53	13.31	13.33	13.45	12.7	16.49	14.43
GLS	10.11	10.1	11.48	13.11	12.94	12.7	20.32	16.8
RAB3D	13.35	14.94	13.84	13.32	12.61	12.71	11.34	13.67

LOC100289561	11.76	12.28	9.19	10.87	11.25	12.72	12.83	12.76
MYADM	15.93	17.26	16.63	16.24	12.83	12.72	10.77	13.73
NMNAT1	11.5	12.39	11.68	13.48	12.03	12.72	14.68	14.63
HEXIM2	13.56	12.54	12.9	11.5	12.17	12.73	10.48	10.41
KLHL36	12.12	12.44	12.71	12.38	12.88	12.73	13.39	13.07
VAV3	10.76	8.89	11.98	12.35	14.09	12.73	19.65	16.98
HOXD11	13.31	12.71	13.29	15.58	12.82	12.74	12.83	11.9
TTC4	12.84	13.32	12.16	11.9	13.35	12.74	13.54	12.9
SCNN1A	14.08	15.6	13.39	14.64	11.68	12.76	10.77	12.51
KDM4A	12.38	13.56	11.87	13.14	12.82	12.76	12.01	12.56
FAM83D	10.08	9.03	9.47	8.16	12.96	12.76	12.29	13.21
SUGCT	8.22	9.98	9.57	8.12	13.03	12.77	11.35	10.52
FAM118A	10.75	9.18	11.46	13.7	11.67	12.77	16.24	14.99
TFDP2	12.73	12.22	14.19	12.9	14.28	12.78	15.78	13.89
CXXC1	13.31	12.65	12.81	12.12	12.38	12.81	9.92	9.88
GP1BB	12.1	13.31	12.06	12.8	11.25	12.81	9.17	10.31
POMT1	12.63	12.57	12.34	12.45	13.03	12.81	12.04	11.76
NUDT4	12.78	11.67	14.15	14.99	12.72	12.81	16.95	13.83
MTA3	14.25	13.11	13.13	13.79	14.2	12.81	15.35	14.65
CCDC85C	12.97	13.04	12.82	11.97	12.33	12.82	10.21	11.15
SVBP	14.84	14.39	13.92	14.03	14.14	12.82	15.09	14.1
PBX1	15.99	14.42	16.87	16.69	14.44	12.82	16.27	15.3
SLC25A24	12.14	11.88	13.59	14.84	14.08	12.82	20.26	16.27
LLPH	10.56	11.4	12.23	12.85	13.16	12.82	17.83	17.12
ASCC1	11.62	12.28	12.9	12.99	13.83	12.83	14.92	14.22
CREB3L2	13.79	12.74	13.98	14.77	13.41	12.83	16.02	15.28
YTHDF3	11.92	10.25	13.99	15.84	13.79	12.83	18.71	16.35
SIAH2	14.2	13.96	14.29	13.58	12.27	12.84	11.08	10.73
TUBGCP6	11.41	10.66	11.08	11.78	12.33	12.84	13.42	13.49
SMIM20	10.06	10.7	10.7	10.34	11.97	12.84	16.75	13.71
ARIH1	12.84	11.99	13.63	15.43	12.84	12.84	15.28	14.93
GATAD1	12.47	12.27	13.08	14.28	13.18	12.84	17.76	16.47
C1D	12.78	13.22	13.94	12.22	15.44	12.84	16.68	16.57
TJAP1	13.15	12.61	12.28	11.35	13.47	12.86	11.61	11.9
RARG	14.43	14.64	13.17	12.43	12.76	12.86	10.64	12.02
PPA2	11.37	11.51	12.58	13.56	12.39	12.86	16.29	13.37
DYM	12.13	12.22	11.85	12.61	13.57	12.86	15.15	15.35
CADM4	13.46	15.34	14.69	12.99	12.76	12.87	10.52	12.91
MSL1	12.47	11.98	13.17	11.89	12.06	12.87	12.64	13.02
SLC35F6	12.69	13.2	12.29	12.39	12.37	12.87	11.71	13.1
ARFGAP3	12.8	12.38	12.37	13.73	13.03	12.87	14.68	13.24
USP9X	13.2	11.2	14.97	16.59	13.5	12.88	19.3	18.17
HES6	9.99	11.62	11.45	9.57	10.92	12.89	9.47	9.24
TIMM9	12.83	13.5	13.87	14.29	15.9	12.89	16.99	14.3
CRYL1	14.21	15.29	14.01	13.29	14.22	12.9	11.75	11.74
TMEM44	11.3	11.47	11.01	12.11	11.92	12.9	11.82	12.17
NUP214	13.32	13.02	13.36	13.33	13.08	12.9	12.76	13.53
UNC50	12.58	12.73	13.42	14.31	12.44	12.9	16.93	15.8
CPED1	10.88	10.71	12.41	12.63	13.55	12.9	17.64	16.04
THAP3	12.05	13.53	11.86	11.99	14.16	12.91	10.67	11.53
LIG1	13.59	12.26	12.11	11.47	13.46	12.91	12.5	13.35
CDR2	12.85	13.5	13.38	14.48	14.09	12.92	15.75	13.18
TP53BP1	12.94	13.12	14.61	14.95	13.69	12.92	16.35	14.25
RAD50	10.01	9.86	12.91	13.76	12.46	12.92	21.14	17.56
CBY1	13.83	15.36	13.62	11.91	14.03	12.93	11.46	13.59
44450	11.59	10.97	13.01	14.88	12.99	12.93	19.24	16.87
BTBD3	12.72	12.22	14.17	15.9	13.45	12.94	18.42	16.37
LRRC45	14.39	13.7	13.65	12.96	12.41	12.95	10.15	10
NBPF1	13.05	13.18	13.13	13.56	12.23	12.95	13.89	12.78
GTF2H3	10.78	9.35	11.27	13.95	13.23	12.95	19.08	16.39
RMI2	13.76	13.27	12.48	12.48	13.83	12.96	13.54	12.47
AKT3	11	12.35	14.56	15.04	13.71	12.96	18.63	16.89
ARHGEF40	13.98	15.2	13.8	12.32	13.15	12.97	11.72	12.35
TBCE	10.68	10.99	11.15	10.99	12.96	12.97	14.58	13.35
ROBO1	13.16	12.49	14.61	16.8	12.51	12.97	16.42	15.57
SLC6A15	10.16	9.53	11.73	12.23	13.63	12.97	16.93	16.32
ATPAF2	13.02	12.3	12.11	9.75	11.41	12.98	10.2	10.89
PRIM1	11.83	11.64	11.47	12.99	13.57	12.98	15.6	12.26
F8A1	14.48	13.78	12.11	13.59	12.55	12.98	10.9	12.55
RRAS2	11.5	10.52	11.75	11.86	12.46	12.98	13.7	12.61
SLC30A1	9.33	8.84	8.77	10.11	12.71	12.98	13.97	13.51
RSRP1	11.63	10.94	13.54	16.29	12.48	12.98	22.55	18.56
PDPK1	11.25	10.88	11.53	12.37	12.13	12.99	13.55	12.81
GRPEL1	11.78	12.78	12.85	12.07	13.87	12.99	13.44	13.33
GMDS	11.58	12.19	11.92	10.39	12.49	13	11.37	10.49
TOM1L2	12.21	11.87	12.25	12.15	13.51	13.02	10.98	12.21
SUGT1	12.66	12.94	13.22	14.44	14.32	13.02	18.94	16.81
SCAMP1	12	12.29	13.22	14.93	13.83	13.02	21.65	18.63
C1QL1	19.31	19.53	17.22	16.9	12.39	13.04	7.81	9.22
SNAP29	11.37	11.31	12.95	12.4	13.54	13.04	13.29	13.3
CHD3	12.52	13.53	12.61	13.34	13.29	13.04	12.45	13.31
MARK3	13.4	13	13.71	13.65	14.69	13.04	16.91	15.75
GFER	13.72	14.39	12.61	13.17	14.75	13.05	9.68	12.31
SPIDR	13.18	12.92	12.65	13.34	12.3	13.07	15.66	14.46
SMIM15	11.99	12.29	13.31	14.75	14.58	13.08	19.57	17.52
C14orf159	13.27	12.51	12.55	11.97	13.09	13.09	12.6	12.42

WDR75	11.89	10.97	13.27	15.17	13.38	13.09	18.29	15.81
ZC3H7A	11.59	11.49	13.47	13.5	13.29	13.09	19.43	16.61
LOC101060341	11.08	9.58	11.69	13.61	12.25	13.1	19.41	16.97
GGT1	10.58	11.44	11.08	11.47	11.12	13.11	10.04	10.38
OSCP1	14.23	14.14	13.52	12.85	13.86	13.12	11.15	13.91
ELK3	12.33	11.65	14.54	16.63	13.57	13.12	17.53	16.67
TMEM87A	10.47	10.33	12.85	13.07	14.98	13.13	17.01	14.54
PSEN1	13.5	13.51	14.63	13.75	13.21	13.13	15.36	14.68
PCTP	13.53	15.31	14.44	14.86	13.48	13.14	13.48	14.78
CCNC	11.7	11.98	12.14	12.71	14.07	13.14	18.89	15.67
CD109	13.48	12.36	15.32	17.95	13.21	13.14	19.54	17.92
RGS19	12.22	12.28	12.24	11.45	13.61	13.16	10.51	11.34
FOXJ3	13.7	13.11	14.51	14.7	14.29	13.17	17.29	15.44
KLF10	14.82	13.74	14.11	14.13	14.24	13.18	16.61	14.24
NFX1	11.86	11.71	12.62	14.14	14.01	13.18	15.84	14.89
ABCD4	11.6	11.75	11.34	11.44	10.77	13.19	11.63	11.43
CTDP1	13.65	13.72	12.79	12.55	12.6	13.2	10.85	11.31
NSMCE3	12.72	12.73	10.85	12.01	10.35	13.2	10.55	11.6
PIM2	12.29	12.85	12.24	11.97	13.09	13.2	11.26	12.19
ZFYVE19	11.95	12.88	13.1	12.92	13	13.2	12.45	13.25
EEF2K	12.34	13.1	12.83	12.48	12.82	13.2	12.39	13.71
TPGS1	12.04	12.19	11.62	9.67	12.66	13.22	8.43	8.7
TMEM185B	12.13	12.51	11.92	11.89	13.12	13.22	12.2	12.34
PPP4R3A	10.75	10.55	12.77	13.69	13.92	13.22	18.25	16.77
RBM6	12.5	12.46	13.94	14.62	14	13.22	18.75	17
POLM	11.37	12.06	10.81	11.43	13.15	13.23	13.04	12.71
EXOSC3	12.15	11.96	12.8	13.38	13.83	13.23	15.32	12.82
CLN8	8.97	8.45	8.47	8.99	13.22	13.24	13.94	12.95
SPAG4	20.75	18.47	17.4	17.63	11.86	13.25	11.16	12.36
CCNY	12.3	12.71	11.74	12.89	13.74	13.25	13.88	13.14
ZNF317	13.96	14.55	15.26	13.81	14.38	13.25	13.91	13.19
TRAF2	13.75	12.8	13.12	11.81	13.12	13.27	11.41	11.9
THEM4	13.43	11.99	15.11	15.66	14.48	13.28	18.73	17.66
CDK11A	12.07	13.17	12.99	12.46	13.11	13.29	14.35	12.94
LIMD1	14.23	14.23	14.42	15.51	13.58	13.29	12.91	13.79
MESDC1	11.35	10.73	11.95	10.67	12.26	13.3	8.86	10.22
HCCS	13.08	13.61	13.69	13.16	14.36	13.3	13.4	13.58
EDIL3	15.53	14.73	16.74	20.55	14.58	13.3	21.08	18.33
SERINC5	11.08	11.12	12.28	12.7	13.75	13.31	16.37	16.2
ZNF277	11.54	11.92	13.06	14.87	15.06	13.31	19.7	16.75
KLHL26	14.67	14.25	13.66	12.79	12.08	13.32	9.66	10.81
ABTB2	12.21	13.14	12.38	11.95	11.96	13.33	10.79	11.11
ALKBH2	13.98	14.48	12.63	12.53	13.52	13.33	12.34	12.16
EMC2	13.35	14.88	15.12	15.85	15.21	13.33	23.07	22.24
ELP5	12.55	12.26	10.39	11.97	11.79	13.34	10.23	11.33
CCDC43	11.06	10.3	11.67	13.23	13.61	13.34	17.65	15.4
UFM1	11.63	11.51	12.82	15.1	14.15	13.34	23.27	19.69
POLR2J2	19.02	17.43	14.55	17.59	16.69	13.35	14.68	16.63
SVIP	12.45	10.16	12.08	11.89	12.8	13.36	11.81	11.87
RGS2	14.11	15.02	16.14	16.12	11.58	13.36	17.99	14.73
ELL2	14.19	14.08	14.97	17.27	13.41	13.36	18.97	16.82
RBMX2	13.82	13.23	13.13	12.69	15.05	13.37	12.47	14.4
SMARCA2	15.91	15.83	16.19	18.62	15.01	13.37	17.62	17.43
SEMA3A	15.09	14.03	17.36	21	13.69	13.37	22.19	18.87
SIPA1L3	12.99	13.67	13.18	13.07	12.74	13.38	11.41	12.51
DHX37	13.16	12.75	12.26	11.15	13.21	13.39	11.8	11.26
ZNF516	12.97	12.11	12.76	13.14	11.98	13.39	12.37	13.47
TEAD2	15.05	16.27	14.45	13.75	14.45	13.39	11.69	13.75
FTO	12.85	13.85	12.86	13.43	13.54	13.39	14.64	14.42
MEX3A	15.69	15.17	15.89	15.91	14.31	13.39	14.05	14.51
RABGEF1	12.34	11.57	13.29	13.69	14.02	13.4	15.93	15.55
PSPC1	13.82	13.13	13.49	13.2	14.13	13.41	14.64	13.74
SSH1	14.34	14.06	14.47	14.57	13.62	13.42	15.15	14.59
ISCA1	11.31	12.76	12.93	13.91	13.49	13.42	18.09	14.94
RPA3	14.35	14.57	14.74	14.99	16.14	13.42	16.22	15.17
PDHX	10.75	12.02	12.28	12.84	13.59	13.42	17.41	16.06
ARMC1	11.89	11.57	12.33	14.26	14.67	13.42	19.94	16.75
SOX15	13.66	14.85	12.55	13.33	12.07	13.43	8.37	11.28
SLC37A4	11.76	11.66	11.41	10.94	12.87	13.43	12.09	11.77
LUC7L	14.15	12.47	14.07	13.25	12.51	13.43	15.12	14.1
TCF20	12.68	12.62	12.67	13.08	14.13	13.43	13.89	14.27
SMUG1	17.03	14.97	15.52	13.51	13.99	13.43	12.68	15
ADCK4	15.62	15.46	15.01	14.26	13.03	13.44	11.21	13.97
IGFBP5	16.26	19.03	16.3	17.76	11.63	13.44	11.43	14.83
CD58	12.24	13.32	13.23	14.38	12.69	13.44	22.65	15.93
KRBA1	12.9	12.58	12.71	12.11	13.96	13.45	11.92	13.6
VCAN	11.24	9.79	11.75	13.42	14.12	13.45	20.73	20.92
KLHL17	11.13	9.48	11.11	10.67	12.58	13.46	10.98	11.62
REPS1	14.58	13.28	15.05	14.6	12.62	13.46	15.15	14.35
PDCD4	9.97	10.8	13.53	14.42	14.45	13.47	19.18	19.21
ZNF316	12.44	12.44	12.5	11.5	12.58	13.48	10.61	10.77
ASAP3	13.25	14.75	13.2	13.5	13.26	13.49	12.06	13.77
ZC3HAV1	15.21	14.57	16.97	17.94	14.17	13.49	15.63	15.42
DHX36	11.17	10.74	13.61	15.55	13.74	13.49	23.62	19.08
MSRB1	14.78	15.97	14.23	12.53	14.13	13.51	10.62	11.31
TJP2	13.31	12.94	12.33	12.89	14.09	13.51	13.19	12.54

FBXW4	15.52	14.85	15.24	15.19	13.09	13.52	13.14	13.62
CDC23	12.45	13.63	13.73	13.82	14.6	13.54	17.68	15.42
ASCC3	11.05	9.11	11.81	13.11	13.44	13.55	20.12	17.4
C16orf72	13.17	12.78	15.14	15.99	14.7	13.56	18.64	16.35
IFNGR1	13.77	12.1	14.62	14.77	13.79	13.56	19.8	17.38
TLE3	15.4	15.39	14.67	14.76	12.25	13.57	10.28	12.57
MFSD11	12.27	12.71	12.87	13.72	13.86	13.57	15.36	14.53
DMWD	14.45	14.13	14.16	13.27	12.84	13.58	10.86	11.23
TTC19	12.31	11.86	13	13.17	13.42	13.58	15.13	13.98
UPF3A	12.53	11.3	14.12	14.59	14.78	13.58	19.1	17.29
LMBRD1	13.24	13.23	13.96	15.03	13.72	13.59	19.52	15.94
HACD2	12.17	10.59	13.14	13.17	14.21	13.61	19.52	17.24
BCLAF1	11.59	11.15	14.42	17.18	14.65	13.61	24.86	20.75
CDC45	16.26	13.63	14.01	13.87	14.8	13.63	12.44	11.33
CCDC74B	14.44	14.06	13.31	13.36	13.86	13.63	12.37	13.21
EEF2KMT	12.98	12.97	11.7	11.83	14.15	13.63	12.17	13.4
MDC1	14.83	14.51	14.51	14.94	13.28	13.64	13.49	14.3
TMBIM4	13.47	12.88	12.99	14.35	13.48	13.64	16.71	16.18
FUNDC1	12.85	14.05	12.95	14.44	15.36	13.66	17.03	16.76
NF2	11.43	12.02	11.28	11.52	13.61	13.68	12.12	12.16
HMMR	11.93	11.52	12.43	13.7	14.81	13.68	20.55	18.43
TRAPPC9	13.76	14.69	13.51	12.72	13.15	13.69	11.83	12.82
MORN5	12.89	13.41	13.74	12.46	15.17	13.69	13.85	16.25
UBE3A	12.11	11.11	13.31	14.4	13.85	13.69	19.54	16.26
PAWR	12.19	11.91	14.15	16.42	12.96	13.69	18.43	16.61
IFT22	14.71	14.79	14.54	13.91	15.36	13.7	13.83	14.34
RABGAP1	13.21	13.17	14.49	16.41	14.63	13.7	18.12	18.42
MTIF3	11.87	11.6	13.07	13.41	14.48	13.71	17.04	14.52
44449	12.88	11.78	12.16	14.66	13.59	13.71	18.53	17.12
SDHAF1	11.77	11.96	10.14	9.78	11.19	13.72	9.14	11.83
IKBIP	11.76	12.33	14.34	15.44	13.9	13.72	19.57	16.25
JMJD7	9.88	12.52	12.75	10.49	14.26	13.73	13.48	12.3
KANK2	14.58	13.7	13.73	13.7	12.93	13.73	11.67	13.22
ZNF513	12.92	14.71	14	12.62	14.8	13.73	12.67	14.12
FAHD1	12.33	12.99	12.36	13.02	16.55	13.73	15.24	15.62
TSNAX	10.76	9.34	13.07	17.23	14.04	13.73	25.98	21.5
ARL4D	13.11	13.34	13.15	12.47	13.81	13.74	11.08	11.52
PANK4	13	13.25	12.05	11.75	13.21	13.74	10.65	11.82
ATHL1	11.26	11.43	11.69	11.88	12.22	13.74	14.53	14.29
OBFC1	11.93	10.95	12.11	12.38	14.49	13.74	15.07	14.9
TMEM181	10.91	10.61	12.89	15.62	14.07	13.74	19.84	19.51
UBE2L6	25.6	28.32	24.4	22.04	14.71	13.75	12.88	14.16
LIX1L	13.28	14.31	14.77	15.11	13.62	13.76	14.09	15.6
PHYKPL	12.97	12.61	12.82	12.3	13.92	13.77	14.24	13.45
MAP2K3	12.64	14.34	13.04	12.24	12.6	13.78	11.09	12.35
PDXDC1	11.57	11.4	12.66	12.15	10.9	13.81	14.52	13.14
MINPP1	11.41	11.15	12.62	13.1	13.47	13.81	15.36	14.45
FAT2	12.52	13	12.63	13.7	12.9	13.81	15.42	14.88
SP110	21.61	21.47	21.4	21.85	16.11	13.81	17.44	14.9
SNAI2	12.13	10.85	14.26	13.59	13.15	13.81	17.9	16.1
TARBP1	12.53	11.87	13.05	14.5	14.21	13.81	20.78	17.07
CTR9	12.85	11.7	13.47	15.01	14.82	13.81	20.25	17.52
PPP4R3B	13.95	11.65	14.88	16.83	14.66	13.81	20.61	18.09
TPR	11.22	11.22	14.34	17.01	14.14	13.81	27.99	22.97
UCK1	13.46	13.59	12.34	12.34	13.92	13.82	12.86	13.35
MYD88	16.07	17.01	16.56	16.65	13.92	13.82	12.86	14.32
SEC63	13.25	12.28	15.21	16.48	15.5	13.82	21.46	18.96
VAMP7	11.92	11.31	13.91	14.92	15.82	13.82	20.57	19.98
EVA1A	15.55	14.39	14.29	14.98	14.5	13.83	13.15	12.72
HILPDA	16.16	15.49	15.72	15.58	13.98	13.83	15.64	14.5
SLC23A2	13.35	11.88	13.63	14.01	14.54	13.83	15.77	14.63
CAMKK2	12.62	12.98	13.17	12.5	13.8	13.85	12.92	13.19
SYAP1	12.55	12.14	13.45	14.8	14.08	13.85	17.7	16.37
FBXL6	11.89	11.68	11	10.79	12.71	13.86	13.05	12.34
UGGT1	11.64	11.42	12.32	13.12	14.04	13.86	16.21	15.44
LOC100653061	13.32	11.47	12.45	15.43	8.77	13.87	15.36	15.57
TRIM5	16.48	16.82	18.33	18.9	14.34	13.87	18.55	16.28
ZNF428	15.97	16.63	16.42	14.55	15.5	13.88	12	12.71
MAPKBP1	12.92	12.93	12.57	11.94	13.59	13.88	12.43	13.75
SELT	12.75	12.15	13.15	13.6	13.79	13.9	18.11	15.26
VPS37C	15.07	14.64	14.14	14.22	14.72	13.91	12.57	13.65
SAV1	14.1	12.96	14.77	16.98	15.57	13.91	19.89	16
PLOD2	17.85	17.26	20.58	22.99	14.03	13.92	23.17	20.24
WDYHV1	12.93	13.69	11.6	12.99	13.88	13.93	13.96	12.57
MMP10	21.84	24.51	24.92	23.38	12.92	13.93	15.08	17.42
RRM2B	9.78	9.12	12.05	14.13	14.98	13.93	26.52	20.98
DISP2	9.99	9.67	9.11	8.71	13.17	13.94	11.18	11.59
RAB29	11.14	11.96	11.35	11.96	14.09	13.94	16.6	14.32
NCOR1	13.43	12.42	14.62	15.66	14.54	13.94	17.71	16.47
ETNK2	15.98	16.26	15.31	15.18	13.78	13.95	11.52	12.87
UCHL3	10.6	11.04	12.2	10.38	12.58	13.95	15.63	14.58
PAFAH1B2	13.62	11.94	13.45	14.02	14.14	13.95	17.15	14.82
TRMT6	12	11.84	13.44	13.9	15.38	13.95	19.92	17.82
ITCH	12.37	11.28	14.04	15.68	14.79	13.95	20.9	17.83
UBN1	14.49	13.61	14.18	14.46	14.45	13.96	13.66	14.35
EPB41L2	15.7	15.45	15.71	17.04	15.85	13.96	17.99	16.43
PILRB	10.95	10.84	10.44	12.38	13.74	13.97	19.63	15.71

CHTF18	12.78	12.03	13.11	11.38	12.05	13.98	11.07	11.09
BLOC1S4	12.72	14.48	13.59	11.73	13.01	13.98	11.08	11.36
RAB11FIP3	12.89	12.78	13.26	14.07	13.4	13.98	12.32	12.5
LRRC14	11.84	12.09	12.19	12.03	13.14	13.98	11.94	12.72
PANX1	13.18	12.53	13.08	14.16	14.33	13.98	16.06	14.82
VRK3	12.85	13.47	13.39	12.85	12	13.99	11.2	12.12
IRF1	16.58	15.58	15.35	14.27	12.86	13.99	12.58	12.65
CASP2	16.32	15.6	15.82	15.96	14.63	13.99	15.86	15.66
C6orf1	12.66	12.68	10.57	11.71	14.3	14	12.6	11.98
NUP58	12.93	11.21	13.1	15.64	13.59	14.01	18.89	15.7
FAM91A1	12.28	11.15	13.37	16.46	15.39	14.01	23.48	20.03
POP4	11.36	12.31	11.69	11.7	13.69	14.02	13.12	13.03
SPAG9	11.7	9.8	12.87	14.62	14.18	14.02	17.55	15.97
MCAT	10.95	11.59	9.76	9.77	13.34	14.03	10.85	9.02
ZFYVE27	14.28	14.02	13.15	13.52	14.89	14.03	14.08	14.63
CEL	16.04	15.77	15.26	14.19	12.97	14.04	12.9	12.45
ABHD4	14.46	15.36	14.69	13.99	14.48	14.04	13.68	12.64
UGT1A6	8.68	9.05	10.1	9.67	13.27	14.04	16.51	12.88
RALA	12.44	13.05	13.49	13.9	15.1	14.04	19.22	15.72
ANLN	12.87	10.68	13.54	15.12	14.57	14.04	23.35	19.53
PPFIA1	13.02	12.09	14.02	14.7	15.28	14.05	16.27	14.9
HERC2	12.87	12.73	12.79	14.47	14.16	14.05	16.2	15.56
RPP30	13.91	11.91	13.68	15.11	14.82	14.05	17.27	16.2
HDDC3	13.11	17.6	15.06	14.59	13.89	14.06	12.24	13.55
TTF2	13.41	11.29	12.85	14	16.02	14.08	19.18	16.79
ORMDL1	11.55	10.24	12.13	13.36	13.45	14.08	22.84	16.79
MAFK	11.9	11.97	12.54	11.65	14.2	14.09	11.79	12.24
ZPR1	12.1	12.72	12.23	11.85	15.51	14.1	14.61	13.27
SMNDC1	13.75	13.13	13.96	15.83	17.93	14.1	18.23	16.7
SMAD5	11.3	10.01	13.53	16.05	15.42	14.1	25.52	21.21
EP400	12.93	13.17	12.83	13.81	13.26	14.11	13.33	13.78
SIPA1	15.98	15.73	15.06	13.81	12.15	14.12	10.04	11.54
POLR2D	12.07	13.2	11.56	12.79	15.34	14.12	13.81	14.92
C7orf26	14.84	16.57	15.42	14.34	14.31	14.13	12.06	12.44
SPECC1L	15.03	14.26	15.12	15.25	15.79	14.13	15.36	15.33
C6orf89	13.88	13.59	13.24	13.47	14.98	14.13	15.14	15.92
SPEN	14.42	13.7	15.59	16.78	14.24	14.13	17.24	16.47
SEH1L	12.22	11.73	13.48	13.73	13.67	14.14	16.95	14.92
MAPRE3	15.15	16.58	13.6	12.44	13.84	14.15	13.95	12.95
SETD5	14.74	13.85	16.3	17.58	13.82	14.15	17.26	16.78
PKP4	12.57	13.02	13.25	14.21	14.27	14.16	16.6	16.49
HTATIP2	14.38	14.33	14.3	15.23	14.51	14.17	15.29	14.26
TEX10	12.98	11.2	13.69	13.29	15.77	14.17	16.91	15.69
FAM131A	14.94	14.65	13.24	12.71	13.74	14.18	10.53	12.13
FAM21C	11.85	10.42	12.57	12.8	14.4	14.18	15.27	13.07
MAPKAPK5	11.85	12.42	13.35	12.43	14.61	14.18	14.64	13.16
TMEM42	14.61	17.48	15.23	14.64	16.04	14.18	12.88	15.21
RCL1	11.87	12.32	13.22	12.73	15.41	14.19	15.41	14.73
FAM20B	11.35	11.23	13.22	14.07	14.43	14.19	16.84	15.81
CCDC94	16.36	14.97	14.18	13.05	13.55	14.2	10.77	11.8
ZBTB45	14.91	15.49	14.43	13.73	12.9	14.21	9.96	10.48
VPS39	14.2	14.72	13.89	15	14.52	14.21	15.57	15.44
DHRS7	13.36	15.14	14.79	14.86	14.43	14.22	15.78	15.68
MRPS9	12.95	12.09	14.03	12.66	14.84	14.22	16.87	16.82
SNX14	13.35	12.39	14.15	15.58	13.94	14.22	20.95	18.64
GTF2F2	13.18	11.37	13.22	13.44	14.52	14.22	19.05	19.07
DHRS4	14.88	14.05	14.59	14.1	11.59	14.23	13.11	9.39
ARHGAP35	13.73	12.9	13.66	13.95	13.92	14.23	14.58	14.07
GCAT	14.45	15.14	14.44	13.36	14.68	14.24	11.24	11.65
KANSL1	13.72	12.68	14.24	15.2	13.5	14.24	14.65	15.19
AP5Z1	12.77	13.07	12.31	12.29	14.17	14.25	11.66	11.88
NLGN2	14.27	15.53	14.71	14.48	14	14.25	13	14.23
FBXO21	13.16	13.47	13.92	13.27	14.22	14.25	16.2	14.46
SWI5	16.56	14.2	14.28	14.24	16.42	14.25	13.7	15.03
DUSP11	16.28	15.19	14.92	16.63	17.34	14.25	20.61	18.42
ADNP2	12.77	12.57	13.58	13.46	14.67	14.26	15.98	14.25
GLA	12.66	12.33	13.23	12.81	13.38	14.26	12.88	14.41
ABCE1	12.63	12.6	12.85	13.63	15.61	14.26	21.5	16.89
AP3M1	12.99	12.14	14.83	14.52	15.58	14.26	19.08	17.62
TDP2	12.57	11.65	12.84	14.1	14.59	14.26	18.91	18.29
MED18	14.26	14.42	14.58	14.18	15.15	14.27	13.64	14.98
GGA3	13.79	14.59	13.79	14.23	14.74	14.27	14.5	15.57
PABPC1L	10.95	9.93	11.97	13.75	12.66	14.27	21.08	18.57
MIEF1	13.59	13.03	13.29	13.53	15.27	14.28	14.19	14.56
MAGED4B	17.03	17.59	16.66	15.77	12.96	14.29	11.22	13.81
ATF6	14.85	14.9	16.04	17.24	15.09	14.29	17.16	15.04
PCED1A	16.04	16.45	14.7	16.53	15.06	14.3	14.26	16.12
ZNHIT2	13.05	14.02	13.98	12.24	12.95	14.31	9.89	10.86
MIER2	12.73	13.21	12.61	12.22	12.52	14.31	10.52	11.76
PYCRL	14.62	14.37	12.73	12.2	13.08	14.31	10.92	12.43
TERF2	13.45	14.3	13.65	13.84	14.79	14.31	15.06	14.08
AVPI1	13.58	13.82	14.22	11.59	13.89	14.32	12.21	11.64
STK16	13.63	14.24	13.2	13.45	14.61	14.33	12	14.09
PDE6D	12.39	14.53	12.63	13.75	14.41	14.33	14.61	16.22
INTS8	12.99	12.02	14.21	15.79	14.73	14.33	19.72	17.01

LRP12	12.76	12.33	14.09	16.5	15.42	14.33	19.52	17.82
RABL2B	14.3	13.89	14.22	15.26	14.18	14.34	16.13	15.12
C1orf123	14.63	13.62	14.34	13.38	17.21	14.35	15.85	14.79
ARHGEF16	15.71	15.65	14.46	14.16	13.8	14.36	11.58	12.75
SFSWAP	14.82	13.75	14.28	14.08	15.19	14.36	14.33	14.97
DNTTIP2	14.21	14.33	15.77	15.41	16.17	14.36	20.91	18.59
FXR2	14.19	14.98	14.1	12.49	13.74	14.37	11.95	14.3
ZNRD1	15.21	15.7	14.03	13.55	13.67	14.37	13.01	14.36
KLHDC2	12.15	13.62	15.01	13.88	14.49	14.37	14.75	14.88
SAMHD1	20.87	20.77	20.99	21.83	15.41	14.37	18.23	15.41
DNAJC10	13.07	13.36	15.05	17.59	15.83	14.37	23.5	21.73
FBXO25	11.77	13.08	12.04	12.28	15.29	14.38	16.76	16.24
CHSY1	17.15	16.45	17.4	18.24	14.39	14.39	16.08	16.27
KIAA0196	12.79	12.13	13.97	15.04	14.58	14.39	19.63	17.13
SRP19	12.98	14.36	13.45	14.87	16.17	14.39	20.5	17.36
SETD1A	13.8	13.71	13.33	14.02	14.12	14.4	12.5	14.24
MRPL30	12.01	11.08	12.67	12.91	13.6	14.4	14.84	14.47
ADGRL1	15.27	15.79	14.59	14.65	13.95	14.4	12.55	14.49
LOC107987001	14.09	11.94	12.98	16.37	15.17	14.4	16.73	16.87
ACAD10	13.45	14.17	13.27	13.04	14.5	14.41	13.69	13.51
SULT1A4	3.48	9.72	13.9	14.05	6.66	14.42	13.21	13.03
DENND4B	14.84	15.11	15.16	15.13	13.89	14.42	14.18	14.04
IDH3A	12.2	11.45	12.67	14.14	15.15	14.42	18.11	14.99
KCNMA1	10.02	10.88	11.61	10.27	13.77	14.43	12.44	12.31
ABCC5	11.87	11.14	12.1	13.17	14.84	14.43	18.69	18.16
NACAD	14.78	15.78	13.73	13.44	13.59	14.44	10.95	12.87
MLF1	13.33	12.21	12.64	14.22	12.91	14.44	14.91	14.33
PUM2	11.86	11.04	14.39	16.79	14.5	14.44	22.09	19.14
ZNF385A	14.37	15.27	13.56	13.22	14.8	14.45	10.95	13.85
CSF1	16.56	16.92	15.67	16.87	14.69	14.45	13.42	14.19
TMEM57	14.12	12.91	13.88	15.07	14.09	14.45	16.74	15.07
CAMK2G	13.76	13.59	13.46	13.79	15.86	14.45	15.8	15.56
CGGBP1	13.58	12.7	14.71	17.11	15.15	14.45	22.81	19.87
TSPAN1	14.17	14.57	13.43	11.43	14.15	14.47	12.16	13.34
NSF	13.41	12.4	13.58	14.68	16.05	14.47	19.09	16.23
NRARP	15.88	15.64	15.36	14.24	13.72	14.48	13.02	13.77
RAB3GAP1	13.4	12.38	13.87	15.59	15.14	14.48	17.61	17.18
ADGRL2	16.98	14.2	17.97	19.87	14.34	14.48	20.68	17.5
HJURP	12.06	9.82	11.44	11.94	15.01	14.49	15.93	13.97
FAM219A	14.72	14.72	14.63	14.55	15.33	14.49	12.11	13.99
GSE1	16.18	14.53	16.14	15.64	14.05	14.49	15.14	15.42
TBCC	17.26	16.38	16.25	14.84	13.71	14.5	12.76	12.69
FAM21A	12.47	12.94	12.52	13.83	15.39	14.5	16.47	15.83
ZBED1	16.07	16.14	15.63	14.93	13.63	14.51	12.29	12.83
THOC6	14.2	14.7	14.12	13.05	15.82	14.51	10.45	14.16
TSEN15	15.58	13.98	16.1	15.69	13.62	14.51	17.8	16.73
EZH2	15.07	14.47	15.05	16.47	17.71	14.51	18.88	17.64
LOC107987034	14.05	11.04	13.81	13.1	16.13	14.52	8.97	15.49
ATG2A	13.92	13.93	14.29	13.13	13.91	14.53	11.55	12.56
PELO	13.3	14.03	14.42	13.56	14.2	14.53	14.16	14.48
SLC35B4	12.08	10.9	12.27	13.66	14.19	14.53	17.32	16.08
COG5	13.45	12.61	14.24	14.84	15.92	14.53	20.15	17.82
VWA9	14.95	15.48	14.67	15.35	15.47	14.55	15.88	15.64
GALNT1	14.92	13.4	17.2	19.61	15.47	14.55	24.55	20.91
PLEKHM1	12.8	12.88	12.81	13.58	13.38	14.56	13.97	14.33
NAGA	14.57	15.53	14.16	15.13	14.01	14.57	12.29	12.54
GEMIN5	14.15	13.65	13.56	13.65	15.41	14.57	15.9	14.47
CALCOCO1	13.99	15.74	14.52	13.33	15.21	14.57	14.74	15.54
SRGAP2	15.45	14.8	17.2	17.21	14.68	14.57	16.22	15.79
WDR70	13.91	12.99	13.55	14.7	13.85	14.59	14.28	13.87
SLC25A26	11.08	12.26	13.7	11.63	13.95	14.6	12.67	13.14
FBXO17	14.11	13.46	12.65	13.09	13.03	14.62	12.47	12.87
COX15	12.2	12.81	12.69	13.02	13.01	14.62	16.07	13.44
NUDT3	11.77	11.17	12.52	11.47	12.41	14.62	14.15	14.37
MAP4K4	14.46	14.05	14.93	16.04	15.32	14.62	16.84	16.19
ZNF394	14.18	13.43	14.17	15.23	14.58	14.63	13.05	13.2
SAP130	14.24	14.07	14.24	13.98	14.59	14.63	13.81	14.08
SLC33A1	13.06	12.52	14.68	14.09	13.31	14.63	16.41	15.33
NFE2L2	13.89	12.65	15.67	17.58	15.18	14.63	21.64	18.57
SLC39A11	14.73	15.3	14.86	16.1	13.94	14.64	15.02	14.67
ASMTL	14.26	14.29	14.54	13	15.23	14.65	12.39	12.64
NUBP1	13.02	14.1	14.42	14.06	14.14	14.65	14.18	14.3
LSG1	12.71	13.71	14.16	13.01	16.47	14.65	16.3	15.65
TFB2M	12.77	10.68	12.45	12.74	14.47	14.65	18	17.52
ACP5	16.03	17.31	15.85	15.42	12.12	14.66	10.92	11.43
CLASRP	13.43	14.27	12.72	12.48	14.77	14.66	12.15	12.41
RAB11FIP5	14.4	14.38	13.39	12.32	13.25	14.66	11.63	12.44
METTL17	11.87	13.7	11.89	12.41	14.72	14.66	15.77	14.12
DPCD	15.33	15.23	16.05	14.66	14.59	14.67	11.87	13.29
TRIM26	15.21	15.25	15.55	15.07	14.04	14.68	12.2	13
CCHCR1	16.08	14.92	15.57	13.65	15.59	14.68	13.3	14.49
TXNDC9	12.88	13.72	14.55	14.97	14.15	14.68	20.61	16.26
NAA40	14.02	14.14	13.17	15.33	13.58	14.69	15.58	14.83

ZNF335	12.86	12.12	12.22	11.84	13.71	14.7	14.87	13.98
LOC102724993	13.11	15.37	13.59	16.26	16.5	14.7	19.05	19.85
RPTOR	15.95	15.03	15	14.6	14.16	14.71	12.19	12.92
SWAP70	12.8	11.15	13.75	15.1	16.3	14.71	21.69	18.56
COG8	16.58	16.94	15.92	16.4	14.82	14.72	12.59	13.9
PLLP	17.24	18.92	15.01	15.67	15.82	14.72	12.62	15.39
SMAP2	16.21	15.83	17.61	15.24	15.33	14.72	15.57	15.69
FAM98A	12.44	12.13	14.13	15.23	16.71	14.72	19.14	17.25
SPTBN2	13.06	13.2	12.7	12.61	13.61	14.73	11.46	12.56
IMMP2L	12.39	12.33	12.8	12.39	14.9	14.73	14.15	14.36
ZNF561	12.06	11.95	13.21	13.41	16.88	14.73	22.16	18.36
MKKS	12.85	13.07	13.74	14.7	14.95	14.74	18.14	14.79
LYRM4	17.22	16.46	15.41	15.87	16.82	14.74	17.38	19.02
ARMCX6	16.18	18.12	17.35	15.85	16.04	14.75	15.31	16.14
CDKN3	14.31	14.61	14.25	13.43	16.42	14.76	20.91	17.89
LGR5	16.38	15.76	17.64	19.56	14.42	14.76	20.36	19.66
SMARCA1	15.4	14.76	17.52	18.5	15.09	14.76	21.72	19.82
TNFRSF25	12.39	10.58	10.28	13.34	12.11	14.77	16.87	15.13
CELF1	15.1	14.67	17.59	17.31	17.23	14.77	18.86	18.82
TUSC1	13.94	13.98	13.29	12.51	13.21	14.78	10.32	11.14
ENKD1	14.77	14.48	15.9	11.95	13.64	14.78	12.76	12.35
NTRK2	14.54	13.75	16.33	16.78	14.48	14.78	18.01	17.21
PNN	12.09	10.81	13.82	17.05	15.1	14.8	26.51	20.08
PSMD9	13.75	14.63	13.03	13.74	16.28	14.81	13.9	14.29
SPAG5	13.79	12.36	13.72	12.54	14.77	14.81	16.01	15.32
SLC39A9	15.02	13.6	13.88	14.27	16.14	14.81	16.43	15.94
APOBEC3B	13.86	13.48	13.99	11.6	14.02	14.82	14.12	11.84
TOR1B	13.76	14.22	14.55	14.69	14.88	14.82	15.63	15.2
RBFOX2	14.15	14.36	14.09	15.68	14.5	14.82	16.27	15.41
ACBD3	14.14	11.9	15.41	17.5	15.18	14.82	21.83	18.99
CSTF3	17.27	16.68	17.75	19.34	18.71	14.82	21.41	20.04
PIGP	14.69	11.89	14.75	16.19	15.18	14.83	18.58	14.51
FECH	15.58	12.85	15.63	15.33	17.09	14.83	15.95	15.37
PHYH	15.28	13.61	14.69	15.06	15.84	14.83	15.78	15.44
ASUN	10.76	10.84	14.12	14.25	15.75	14.83	19.6	18.58
ZNF664	15.61	13.98	16.25	19.37	15.75	14.83	22.49	19.88
OTUB2	12.06	12.09	12.74	11.52	15.73	14.84	15.04	14.6
FOCAD	14.43	14.08	14.72	14.62	14.37	14.84	15.75	15.83
IWS1	12.9	12.43	14.82	15.42	15.41	14.84	18.86	16.52
TXNDC11	15.57	15.58	14.81	15.76	14.27	14.85	13.63	14.47
LOC730098	13.09	11.47	13.61	12.01	11.72	14.86	12.15	10.98
TMEM39B	15.91	15.38	15.74	13.56	14.93	14.86	13.72	12.7
PICK1	13.55	14.71	14.23	13.2	16.26	14.86	13.23	15.05
VEGFC	17.07	15.76	16.93	17.67	14.44	14.87	15.62	14.67
BCL2L13	14.37	14.51	14.07	14.34	14.96	14.87	14.54	14.8
GNE	13.36	12.71	13.58	14.1	14.91	14.88	15.31	14.89
GATS	12.52	14.64	14.09	14.65	13.44	14.88	15.31	15.41
METAP1	15.34	14.51	15.11	16.04	15.34	14.88	17.95	17.23
MICAL1	15.01	15.13	14.64	13.73	14.03	14.89	13.5	13.47
MECP2	14.31	14.74	14.81	14.5	14.25	14.89	13.2	13.52
SRPK1	11.82	11.13	13.22	15.39	16.46	14.89	21.38	18.64
DPAGT1	14.15	14.14	13.7	12.41	15.79	14.9	15.21	15.91
COX16	14.68	15.26	15.01	14.98	14.52	14.9	17.78	16.3
MRPL48	13.44	13.95	15.13	13.89	15.78	14.91	16.2	13.78
SHISA4	17.29	17.47	16.88	17.39	16.78	14.91	11.91	14.52
MTRR	13.54	12.18	15.54	16.01	16.29	14.91	21.84	18.91
PANK3	11.86	11.87	13.56	15.14	13.84	14.91	21.87	20.02
TCTA	15.45	14.67	14.6	15.01	16.33	14.92	14.5	14.82
TIMM17A	12.96	12.86	13.6	15.36	15.91	14.92	19.61	20.36
CENPT	13.01	12.3	12.49	12.87	16.95	14.93	16.42	13.57
KCTD10	14.4	14.72	15.09	14.68	16.4	14.93	15.37	15.82
MSI2	12.72	11.9	14.35	16.81	16.46	14.93	21.35	19.54
CCDC14	10.05	9.69	12.37	15.88	13.39	14.93	27.5	22.77
PINK1	12.82	14.32	13.36	12.66	14.54	14.94	12.57	13.55
ATAD3B	12.64	10.2	11.02	12.82	14.2	14.95	14.6	12.59
HADH	12.87	13.19	13.31	12	15.48	14.95	13.93	13.85
SIN3A	15.72	15.65	16.01	16.92	14.95	14.95	16.77	17.78
IKBKAP	13.81	12.7	13.56	14.97	15.02	14.96	18.34	16.35
RFX5	13.56	14.5	14.21	14.78	15.63	14.96	17.35	16.64
44260	14.32	13.89	15.99	16.54	15.61	14.96	18.34	17.74
C8orf58	19.18	18.35	18.88	19.54	14.99	14.97	14.53	13.79
TMEM138	11.96	11.23	11.67	13.21	14.88	14.97	14.56	15.09
U2SURP	13.03	13.29	15.35	17.9	14.4	14.97	24.77	21.34
IRF2BP1	15.72	14.97	13.65	13.48	14.43	14.99	9.77	11.44
NAA60	14.89	14.31	13.57	14.07	15.34	14.99	11.99	13.81
CEP104	14.62	14.09	14.22	15.16	15.06	14.99	16.04	14.84
PPP4R1	15.07	15.15	15.98	16.21	16.01	14.99	18.42	15.93
TRNP1	16	16.11	13.02	14.09	13.53	15	11.2	12.78
TRAM2	14.92	14.77	15.83	16.46	15.75	15	16.97	15.94
GRWD1	12.89	13.65	13.49	12.19	15.12	15.01	11.11	12.79
KAT5	13.93	14.51	13.53	11.94	13.69	15.01	12.92	13.27
RAB26	12.39	15.1	12.58	12.35	11.42	15.02	12.47	14.58
POLR3GL	13.83	14.22	15.18	15.3	15.6	15.03	16.44	18.53
EIF2AK2	19.44	19.35	23.63	26.01	16.22	15.03	21.97	19.45
GPR137B	14.27	14.72	14.74	16.47	15.33	15.05	14.62	15.73
GNB1L	13.63	14.36	12.71	11.34	13.33	15.06	11.25	11.72

FZR1	15.54	15.74	14.47	13.26	14.48	15.07	11.09	12.28
PRSS12	14.09	12.8	13.78	14.76	15.93	15.07	16.42	15.96
STRIP1	13.64	13.93	13.7	13.21	14.36	15.08	15.02	14.4
MCMBP	14.62	14.69	14.98	15.01	15.55	15.08	17.02	14.65
FZD1	17.62	16.49	16.87	16.85	14.08	15.09	13.18	13.92
EPN2	15.44	15.15	15.07	15.15	15.58	15.09	15.12	15.1
CALCOCO2	14.77	15.23	16	16.12	16.59	15.09	19.28	18.3
POLR1E	14.2	14.89	14.15	14.13	16.71	15.1	16.61	15.56
C12orf65	14.78	14.69	13.11	16.6	15.12	15.1	14.31	16.99
ADRA1D	13.76	15.74	13.56	14.06	15.81	15.11	12.26	13.97
GTF2H4	15.55	14.92	13.99	13.59	15.22	15.11	12.87	15.34
ZCCHC9	16.2	16.14	18.05	18.09	17.96	15.11	22.28	19.29
CDH5	14.36	15.28	15.86	16.62	16.11	15.12	16.16	14.92
TBC1D13	15.82	16.66	16.41	15.39	14.49	15.13	12.6	15.17
RBM12	15.18	14.16	15.22	15.29	15.81	15.13	17	17.2
NSMCE4A	17.39	15.97	16.53	17.05	14.97	15.13	18.44	17.38
MCOLN1	12.74	13.41	13.15	12.75	12.97	15.14	11.98	12.41
PTCD1	14.95	14.7	14.43	13.9	14.54	15.14	12.63	13.3
SSBP2	18.79	18.81	19.78	18.2	14.1	15.14	18.03	16.09
RAB32	15.94	15.76	15.2	14.86	14.34	15.15	13.3	13.5
TELO2	14.87	14.78	14.34	13.21	14.15	15.16	12.03	12.7
TBC1D22A	14.91	15.62	14.54	15	16.53	15.17	13.67	13.34
MAPK14	16.59	14.97	13.75	14.67	17.24	15.17	15.79	15.52
TCEB3	14.59	14.02	14.67	15.52	16.2	15.17	16.58	15.81
ANTXR1	19.1	16.69	17.81	19.32	16.26	15.17	18.75	18.09
GTF3C6	14.3	13.69	13.33	14.63	16.79	15.17	17.75	18.17
RHOQ	13.74	13.15	14.6	15.3	16.9	15.17	24.33	21.45
POLL	14.56	13.81	14.07	14.03	15.1	15.2	13.47	13.14
DNAJC14	16.27	14.75	15.52	15.69	16.5	15.2	15.93	17.15
GNA13	12.16	11.51	14.4	16.44	14.85	15.2	21.69	17.84
AK3	13.17	12.06	13.79	15.41	16.44	15.2	20.93	18.62
TAB1	16.68	16.41	16.16	14.49	13.62	15.21	11.91	12.82
MYL5	13.3	15.66	14.92	13.04	13.28	15.21	13.53	14.96
LRP11	14.65	12.55	15.62	15.81	16	15.21	17.92	17.17
WDR43	12.57	10.9	14.58	15.55	16.11	15.21	21.54	19.51
OGG1	15.7	14.37	14.34	15.38	15.21	15.22	12.82	13.94
RRP12	13.87	14.71	13.73	13.14	17.38	15.22	13.65	14.73
LEPROTL1	14.62	13.15	14.04	16.03	15.26	15.23	17.67	15.93
SARS2	14.05	14.71	14.02	13.53	14.56	15.24	12.85	13.44
NARFL	14.25	14.04	13.9	12.24	13.56	15.25	11.47	11.52
MROH1	13.46	13.42	12.82	12.51	13.28	15.25	12.64	13.71
PDK2	15.18	16.39	15.62	13.78	14.77	15.26	12.36	13.44
SGK1	17.58	18.43	18.2	17.82	15.22	15.26	17.33	15.7
VPS26B	15.68	15.43	16.78	14.99	16.09	15.27	13.92	15.31
UBE2B	14.16	14.67	14.75	15.83	16.72	15.27	24.24	19.93
NQO2	16.6	18.07	17.45	15.66	17.39	15.28	14.85	13.99
LZIC	14.84	14.69	14.04	16.37	17.44	15.28	19.98	18.14
CTU2	14.94	14.47	14.17	12.8	17.03	15.29	12.18	13.12
ULK1	15.28	14.75	14.11	13.48	14.28	15.29	13.13	13.99
RFT1	13.93	15.11	14.5	14.42	14.27	15.29	14.1	14.38
ABT1	15.32	16.05	15.29	15.65	15.46	15.29	15.44	14.57
TOP2B	14.25	13.5	16.87	19.44	15.08	15.29	24.06	21.28
NFKBIB	15.28	15.25	13.52	10.96	16.68	15.3	12.46	13.61
SRPRB	13.55	14.14	13.03	12.97	14.28	15.3	15.72	14.57
RUFY1	13.72	13.18	14.31	14.15	14.25	15.3	13.77	15.5
C7orf55	15.9	14.49	14.33	11.83	14.09	15.31	9.86	12.26
STK4	13.92	12.04	14.67	15.2	15.8	15.31	19.03	18.24
SEMA3C	10.96	11.67	13.07	15.73	16.2	15.32	25.36	21.78
BORCS8	14.72	15.13	14.25	12.04	15.01	15.33	12.23	13.07
MED22	15.15	14.66	14.32	13.76	14.08	15.34	12.59	13.96
C22orf29	14.25	14.49	14.45	14.18	14.83	15.34	13.74	15.22
SETD3	15.98	16.65	17.22	17.11	17.36	15.34	18.9	17.77
SLC25A37	14.77	16.1	15.49	18.05	16.98	15.35	19.76	18.1
TTYH2	14.5	14.86	14.79	14.26	15.09	15.36	13.71	14.24
TNC	26.89	25.62	23.97	25.55	14.3	15.36	14.12	14.56
TDRKH	15.35	14.86	16.73	15.99	16.92	15.36	16.43	15.89
SETDB1	17.36	16.18	16.21	16.85	16.83	15.36	17.26	16.71
GPN3	12.54	13.52	14.56	15.99	16.37	15.36	19.98	18.12
VAMP2	14.92	16.28	15.97	16.27	16.43	15.37	15.47	16.68
TMEM167B	14.37	12.88	14.37	15.38	16.46	15.37	19.86	19.59
HOXB7	17.59	20	18.33	17.61	16.4	15.38	14.62	14.56
POGK	15.73	15.73	16.31	16.21	15.75	15.38	17.22	15.81
KIF2A	13.17	12.44	16.62	18.54	15.65	15.38	25.91	23.1
PIGC	16.38	17.29	16.58	18.49	17.58	15.39	18.81	16.78
TMEM126A	13.42	12.92	13.85	13.59	14.58	15.39	19.23	16.94
NAA50	13.61	12.51	15.47	16.7	16.95	15.39	21.68	19.63
ANKHD1-EIF4EBP3	12.93	12.24	14.96	15.86	12.78	15.4	17.36	16.85
PRADC1	12.86	15.17	11.66	12.05	15.75	15.41	11.43	12.39
MKI67	14.62	12.86	15.84	15.35	16.53	15.41	19.87	18.22
C10orf10	17.27	17.21	16	14.94	17.45	15.42	16.32	13.81
HHLA3	19.32	19.87	18.7	18.83	17.43	15.42	14.63	17.74
BRF1	16.12	16.21	16.04	14.47	16.26	15.43	12.33	12.88
ENDOV	15.69	16.01	13.81	13.43	13.9	15.43	13.46	13.91
CDKN1B	16.08	16.06	17.43	17.92	14.94	15.43	17.43	17.72

IER5L	15.75	15.36	13.73	13	13.23	15.44	9.5	11
BHLHE40	17.84	18.01	17.94	18.71	14.62	15.44	16.27	15.26
CREB3L4	14.98	15.76	15.07	14.04	15.13	15.46	13.22	14.24
LOC100996709	13.22	12.25	16.4	16	16.44	15.46	19.44	16.86
HSD17B12	12	12.41	13.53	14.25	15.66	15.46	22.33	17.87
NCKAP5L	14.86	15.97	15.63	14.59	14.99	15.47	12.45	14.43
ACADM	13.47	12.85	15	16.45	15.49	15.47	22.63	19.73
TOM1L1	12.75	13.37	14.12	15.6	15.93	15.47	20.37	20.1
SRSF10	15.94	14.22	17.26	21.24	16.06	15.47	27.63	21.6
RRP1B	13.71	13.77	14.66	15.45	16.94	15.48	16.46	16.72
C19orf54	16.95	17.38	16.83	15.85	15.13	15.48	13.81	17.02
ULBP2	13.89	13.32	13.96	13.15	14.69	15.49	13.62	15.76
SLC26A6	15.72	15.38	15.59	15.77	15.01	15.49	14.82	16.44
DHRS13	18.39	18.61	16.59	16.17	14.55	15.5	11.71	12.88
RNASET2	16.1	17.92	16.28	15.02	15.98	15.5	13.53	13.48
DYRK1B	14.82	14.75	13.85	13.92	14.06	15.5	10.94	13.85
MAP3K3	14.87	15.13	14.36	14.51	13.94	15.5	12.69	14.11
GLI3	16.7	16.88	16.8	17.09	15.27	15.5	15.85	15.84
NUP160	14.86	13.8	14.84	15.61	17.18	15.5	21.17	18.04
SESN3	13.26	13.05	16.28	19.8	14.89	15.5	24.68	23.78
SMYD3	14.35	15.12	14.62	14.8	15.3	15.51	14.17	14.47
TOMM70	12.9	12.13	14.24	14.96	16.69	15.51	21.59	16.58
SLC12A2	15.96	15.33	18.26	20.65	13.96	15.51	23.08	21.81
TRIM41	13.77	13.36	14.51	14.47	15.31	15.52	14.18	14.5
TMEM11	15.84	15.39	15.4	13.52	14.57	15.53	14.05	13.35
HNRNPLL	15.29	14.51	15.25	16.75	15.56	15.53	16.26	15.76
RARA	17.5	18.8	18.37	16.63	13.19	15.54	11.48	13
GSTT1	15.43	16.06	12.54	13.44	15.35	15.54	12.07	13.86
ABL1	17.4	17.7	16.64	16.87	14.98	15.54	14.07	14.4
CPT1C	17.74	20.67	17.99	17.94	14.63	15.55	14.3	14.86
CRNKL1	16.06	13.92	16.37	18.01	16.48	15.55	19.7	19.02
CD68	14.22	13.81	13.7	12.93	16.32	15.57	14.33	14.27
ZNF274	15.77	14.34	13.99	15.69	15.63	15.57	16.94	16.83
TAB2	15.1	14.5	18.43	19.38	16.42	15.58	22.94	20.04
CENPM	17.74	16.71	16.69	13.84	14.78	15.59	12.42	14.29
KCTD13	17.19	15.94	14.57	16.59	15.54	15.59	14.85	15.3
KCNK6	13.62	13.21	12.82	13.18	13.94	15.59	13.51	15.69
CLK2	16.93	15.63	17.03	16.7	17.94	15.59	20.45	18.51
ITPKC	17.24	16.99	17.62	15.54	15.74	15.6	13.51	14.57
ZNF672	17.44	17.33	16.77	16.86	15.93	15.61	12.17	14.5
COQ5	16.12	15.94	16.22	14.91	15.55	15.61	15.27	15.91
RABEPK	15.82	14.94	14.57	15.07	17.31	15.61	14.75	16.35
SRGN	13.5	16.08	16.04	17.28	16.81	15.61	20.27	19.19
JMJD6	18.33	17.12	16.73	17.8	17.05	15.62	15.68	15.95
DROSHA	15.47	14.6	15.5	17.37	15.73	15.62	19	17.76
LOC100506248	19.28	14.22	17.31	19.27	18.24	15.63	16.22	15.13
CPE	16.18	14.87	16.03	17.69	15.49	15.63	17.31	16.33
TM2D1	15.21	16.02	15.94	17.86	14.91	15.63	21.58	19.49
SCOC	13.65	15.27	15.02	15.45	16.17	15.63	22.08	19.76
RNF31	17.89	18.96	17.85	15.65	14.59	15.65	12.4	13.41
TCEAL3	17.54	15.8	16.85	16.37	15.57	15.65	14.66	17.46
KCNJ2	8.22	7.26	9.61	10.83	18.28	15.65	22.88	20.41
LSM1	16.25	14.16	17.62	17.86	16.15	15.66	17.89	18.98
TTC17	15.17	14.02	15.61	16	16.96	15.66	21.01	19.23
GATSL3	15.96	18.11	16.07	15.93	14.36	15.67	13.42	14.9
MADD	16.57	17.29	15.91	15.73	15.32	15.67	15.28	15.51
SQRDL	15.19	15.73	15.38	15.66	16.27	15.67	16.67	15.64
RBM18	14.83	13.84	15.86	18.21	18.26	15.67	23.2	21.2
RRAGC	13.66	13.46	14.79	14.82	15.56	15.68	16.88	16.92
CBX5	15.36	14.75	16.49	18.04	17.32	15.68	19.06	19.12
PRKAR1B	13.83	14.32	13.79	12.89	15.55	15.69	11.19	12.16
SULF2	11.76	12.4	12.27	11.83	17.16	15.69	16.37	17.27
ARL17A	14.67	11.21	14.41	15.9	14.05	15.69	22.32	19.13
NOA1	13.22	13.67	14.44	13.05	14.74	15.7	13.36	13.32
SFXN3	16.7	16.84	16.13	17.04	15.77	15.7	16.06	16.11
IRX3	15.52	16	13.29	13.87	13.6	15.72	10.59	13.28
BRD3	16.95	17.01	16.89	17.4	15.19	15.72	14.14	14.8
SH2B3	12.52	12.5	13.15	13.36	15.37	15.72	15.58	15.33
NIP7	15.17	13.21	15.67	15.24	16.78	15.72	16.04	15.44
ZNF692	10.56	10.29	10.95	12.3	15.12	15.72	17.06	15.8
RPRD1B	14.29	15.1	15.83	15.81	16.48	15.72	15.99	16.3
CCDC25	12.47	12.12	14.23	15.43	15.17	15.72	19.48	17.9
GSTM4	17.93	17.28	14.84	15.61	16.03	15.73	16.09	15.93
PPIL1	15.56	14.24	14.23	14.69	17.75	15.73	18.1	15.96
MUM1	15.63	17.54	17.06	16.68	15.77	15.73	15.12	16.2
DENND5A	16.99	17.08	17.78	17.2	17.83	15.73	17.28	16.42
RFWD2	15.76	17.28	16.19	17.11	15.74	15.73	17.76	16.92
BCS1L	15.51	15.64	15.45	13.37	14.2	15.74	12.96	13.97
ANKRA2	14.15	12.03	13.88	15.34	14.83	15.74	19.94	18.43
PRPF38A	16.67	14.65	17.23	18.09	15.58	15.75	20.04	17.57
RNF139	14.84	15.26	15.78	15.69	16.96	15.75	19.33	18.01
VPS9D1	13.72	14.54	14.77	13.82	16.09	15.76	13.58	14.58
KCNAB2	12.77	14.67	13.83	13.06	15.22	15.78	12.53	13.19
PAFAH1B1	14.31	13.49	15.34	16.54	16.04	15.78	20.97	17.48

TCERG1	14.1	13.68	15.02	17.05	16.03	15.78	23.28	21.9
MFSD5	17.84	15.62	16.45	16.61	16.61	15.79	14.73	14.47
TMX1	15.11	14.75	16.04	17.79	16.89	15.79	24.5	20.14
ZRANB2	15.31	14.39	16.61	19.25	16.25	15.8	26.51	21.81
CARS2	13.94	13.84	13.55	14.93	15.79	15.81	14.04	15.11
ZNF706	15.84	14.22	14.24	15.2	16.41	15.82	16.67	16.46
IGF1R	16.72	16.6	16.05	18.14	14.88	15.83	16.49	17.13
DDX50	13.65	12.25	14.16	15.46	15.27	15.83	19.69	18.16
MEST	25.02	23.05	25.58	25.84	15.75	15.84	18.06	17.45
MRPL44	13.36	12.91	13.34	14.64	14.17	15.85	16.6	16.11
MEF2D	17.39	17.23	16.55	16.83	15.65	15.86	14	15.21
SMC1A	15.85	14.85	16.65	16.57	16.36	15.86	17.39	16.56
SGF29	18.16	20.46	15.43	16.9	16.84	15.88	12.89	14.52
KIAA0430	15.34	14.93	16.26	16.51	15.75	15.88	19.79	17.57
FAH	16.15	16.98	16.39	14.08	16.52	15.89	13.78	14.02
GNL1	15.61	16.52	16.44	15.22	15.6	15.89	14.31	14.19
CTDSPL	16.94	16.02	17.01	16.32	15.84	15.89	17.98	16
LPXN	14.76	15.92	15.97	15.13	16.8	15.89	18.25	16.93
NUP43	14.34	14.24	14.48	15.61	15.63	15.89	20.6	17.22
COPS8	14.83	14.18	15.6	16.98	17.42	15.89	19.98	18.36
PLEKHA1	14.64	14.69	16.99	20.55	16.94	15.89	25.73	23.64
AP1G1	15.47	14.49	16.27	17.94	16.76	15.9	20.7	18.96
LSM14A	14.55	13.15	16.21	18.41	16.09	15.91	21.28	19.73
ZNF146	14.21	13.66	15.62	17.46	15.06	15.91	25.43	22.07
TAP2	16.26	16.45	16.35	16.49	15.95	15.92	13.76	13.91
NKD2	17.56	18.4	17.88	16.79	16.05	15.92	14.45	15.82
GRAMD4	15.14	14.99	15.53	14.4	16.03	15.93	14.62	15.06
ASAP2	14.2	13.04	16.33	17.86	15.33	15.93	20.52	18.74
MFSD12	14.46	14.86	14.07	14.7	13.83	15.94	12.38	12.05
AXL	22.69	23.63	22.66	21.23	15.64	15.94	15.24	15.89
PDPR	14.56	15.31	15.57	15.22	15.26	15.94	17.03	16.82
DCBLD1	13.62	14.81	15.36	14.71	16.01	15.95	17.51	16
TLK2	15.66	14.71	17.46	18.59	16.26	15.95	19.15	18.42
MAPK11	13.7	13.79	11.6	12.72	15.93	15.96	14.37	15.03
CCDC90B	14.15	13.39	13.99	14.73	15.16	15.96	20.91	17.21
EIF3J	14.7	13.89	17.31	18.54	16.07	15.97	21.02	18.73
GOLGA8B	10.82	9.89	13	15.29	14.22	15.97	29.66	24.35
ZNF496	21.55	20.16	21.52	21.51	14.58	15.98	14.18	15.01
C16orf62	15.2	16.06	15.44	15.31	16.26	15.98	16.03	16.52
CTCF	17.63	17.92	19.29	18.54	16.54	15.98	17.18	17.21
RHOBTB3	14.56	12.88	16.27	18.31	16.48	15.98	23.08	19.88
UBAP2	14.12	13.81	14.71	14.89	15.92	15.99	15.83	16.33
FAM122B	14.23	13.31	16.08	16.48	17.18	15.99	22.72	20.36
PSMD5	15.24	15.11	15.97	15.79	17.02	16	19.66	16.78
VPS4B	13.69	12.39	17.11	18.34	16.69	16	23.91	21.76
AFF4	14.48	12.63	17.33	20.5	16.84	16	24.89	22.22
GSTT2B	15.83	16.64	17.01	16.06	17.93	16.01	13.42	14.24
RWDD1	15.07	14.84	15.83	15.54	17.33	16.01	18.94	18.52
DLG1	11.84	10.46	15.16	15.75	18.05	16.01	25.92	21.45
KIF5B	13.35	12.44	15.71	19.16	16.02	16.01	27.93	24.07
NCOA5	17.32	17.09	17.39	17.02	16.34	16.02	16.11	16.59
FYN	18.88	16.7	19.55	19.76	16.54	16.02	17.44	16.61
UTP14A	14.43	16	16.47	15.12	17.62	16.02	17.58	17.98
LOC107984841	13.02	10.77	15.03	18.17	14.47	16.02	22.54	19.22
CEP57	14.6	14.06	16.45	17.4	16.72	16.03	24.75	21.46
VARS2	14.62	15.2	14.51	13.84	14.78	16.04	13.92	12.99
DGCR8	17.14	17.76	17.68	17.9	16.15	16.04	16.42	16.01
MICU1	14.76	15.18	15.78	15.68	17.22	16.04	15.83	16.47
HDAC11	13.88	15.86	13.95	13.72	15.89	16.05	11.99	14.47
TMEM2	17.18	16.86	18.95	20.9	16.56	16.05	20.26	18.38
ATP2B4	15.57	16.21	16.67	17.94	15.41	16.06	19.02	18.94
B3GNT9	16.69	17.35	15.53	14.45	15.44	16.07	13.04	14.87
FOXO1	16.11	15.01	17.11	16.86	15.18	16.07	16.55	16.07
LETM1	14.65	14.43	13.73	13.26	15.88	16.08	13.95	15.4
CDCA8	13.05	14.01	12.2	11.44	16.69	16.08	16.08	16.03
MFSD1	14.02	13.32	15	14.54	17.37	16.08	20.11	18.18
BTBD10	15.04	13.52	16.55	16.27	17.14	16.08	21.85	19.05
IPO4	15.32	15.07	13.89	14.08	14.56	16.09	12.91	12.44
PIGV	14.49	15.12	14.94	14.66	16.68	16.09	14.94	15.15
TMEM104	14.9	15.74	14.7	14.32	15.07	16.1	13.65	16.53
RALGAPB	13.92	12.99	14.91	16.04	16.43	16.1	21.09	18.58
SAT2	15.39	16.41	15.47	15.36	18.84	16.1	15.63	19.55
AGPAT5	12.92	11.83	14.56	14.75	17.35	16.1	23.47	19.81
FBXL15	16.44	15.29	13.67	12.62	15.43	16.11	10.55	12.82
ABCB6	15.51	15.7	15.49	14.82	15.31	16.12	14	14.19
MTG1	16.24	16.44	16.17	16.61	18.17	16.12	15.2	15.51
HEY1	16.97	17.34	16.61	17.56	14.76	16.12	15.35	16.51
CA2	9.32	9.39	9.45	9.2	17.89	16.12	20.46	17.06
DNAJB12	12.9	13.47	14.18	13.44	15.9	16.13	14.68	14.63
FN3K	19.89	20.6	18.64	17.64	16.64	16.14	12.6	14.08
TMX3	12.68	13.06	14.81	18.16	16.38	16.14	29.87	25.65
MRPL50	16.12	16.66	17.09	19.16	17.76	16.15	23.26	20.39
MOB2	14.62	14.07	14.14	13.69	16.07	16.17	13.2	13.62
WNK2	18.63	18.92	17.44	17.1	14.6	16.17	12.4	14.22
METTL2A	14.66	14.62	15.33	15.09	16.21	16.17	16.52	16.48
WDFY1	12.83	12.86	15.18	16.28	15.77	16.17	21.23	20.79
REXO1	15.1	15.19	14.93	14.22	15.79	16.18	12.48	13.13
FOXK1	16.22	15.91	15	15.16	16.66	16.18	13.95	16.25

UNC119B	15.6	15.01	15.5	15.95	17.87	16.18	16.55	16.76
TSPYL1	15.31	15.2	15.2	15.28	15.71	16.19	15.79	16.01
GMIP	14.11	15.14	14.3	14.33	14.21	16.2	14.47	14.61
KLHDC4	14.15	15.04	12.78	13.51	14.74	16.2	14.62	14.77
LRSAM1	16.2	16.11	15.19	14.83	15.29	16.2	13.11	15.43
RHNO1	14.39	14.43	14.98	14.49	16.4	16.2	17.75	15.78
SEC22C	15.21	13.15	14.64	15.77	15.78	16.2	16.68	16.77
UIMC1	13.3	14.96	17.09	16.32	16	16.2	19.35	16.77
HARS2	15.75	15.04	16.35	17.02	17.24	16.2	17.6	17.24
NR1D1	12	14.96	12.05	13.07	14.59	16.21	12.3	14.5
PUS1	14.95	12.77	12.95	13.13	15.74	16.22	15.46	14.46
LRCH4	16.37	16.27	14.75	14.72	15.19	16.23	13.22	14.42
DUSP3	15.97	16.5	16.28	15.98	16.77	16.23	16.97	16.11
TEX2	13.83	14.91	15.23	15.69	16.23	16.23	17.72	17.24
DNAJC2	15.66	14.8	17.25	17.54	18.42	16.23	26.24	22.89
CAND1	15.9	13.35	16.82	19.63	16.8	16.24	23.83	21.86
TM2D3	13.98	13.79	14.52	17.31	16.22	16.26	16.56	18.75
UBXN4	16.34	14.83	18.53	22.76	18.4	16.26	29.79	24.19
JADE2	20	19.39	19.19	18.67	16.51	16.27	14.33	15.19
GRIPAP1	16.93	17.32	16.85	16.66	18.14	16.27	17.84	17.17
MRPL35	18.25	16.31	17.72	15.97	22.66	16.28	22.95	17.14
SERF1B	13.18	12.65	13.33	15.26	18.81	16.28	19.92	18.43
ZFP91	15.6	14.62	16.58	16.77	17.77	16.29	21.3	18.94
PLK3	12.73	12.9	12.02	12.78	15.2	16.3	14.16	13.76
RPH3AL	15.15	17.69	16.17	15.3	15.26	16.3	11.98	15
GNG10	16.32	18.26	16.81	20.58	19.77	16.3	28.05	23.41
FAM229B	18	16.24	15.23	15.45	14.21	16.31	14.49	14.86
TRAK1	15.48	15.28	16.05	17.29	17.1	16.31	17.6	17.59
MTFR1	13.36	13.02	15.51	16.2	17.04	16.31	19.42	18.08
DCTN6	13.36	13.07	12.98	16.2	15.31	16.33	20.35	17.81
EMC9	15.33	13.61	15.25	14.34	13.68	16.34	12.34	13.05
MTIF2	13.87	13.99	14.97	17.41	17.94	16.34	25.21	22.1
DNAJC30	16.63	16.21	14.52	14.58	15.28	16.35	11.9	12.67
COX20	16.55	14.08	15.56	17.26	16.51	16.35	20.31	18.02
TFAM	14.3	14.83	16.09	17.02	17.16	16.35	21.24	19.61
ACSF2	19.1	20.36	17.06	18.45	16.82	16.36	14.83	17.79
ARL8B	14.2	14.07	16.29	17.25	17.89	16.36	23.06	20.43
GRSF1	14.99	14.97	15.07	15.39	16.96	16.37	20.79	18.8
TMEM209	15.25	14.7	16.89	20.65	16.95	16.37	24.28	19.65
CDK12	16.31	14.68	16.59	17.25	16.95	16.39	19.49	18.14
D2HGDH	13.57	12.29	13.09	13.63	14.57	16.4	15.86	15.49
BBS2	14.85	14.79	14.67	15.45	16.29	16.4	18.53	18.43
LOC100289279	19.06	18.12	18.75	19.45	14.28	16.41	14.69	14.65
KCMF1	15.94	16.43	15.74	17.48	16.81	16.41	16.69	15.32
SLC25A38	15.16	14.91	15.56	14.48	16.42	16.42	15.13	16.47
KAT7	16.44	17.35	16.67	16.23	17.53	16.43	16.97	16.65
POLR1C	16.21	14.96	15.81	16.42	16.85	16.43	17.11	17.27
CHMP1B	15.44	13.41	15.58	16.34	16.88	16.43	17.89	17.4
AHSA2	11.45	10.99	13.28	17.88	15.11	16.43	29.04	24.53
CCDC57	14.62	14.66	14.73	15.15	15.71	16.44	15.41	15.27
TVP23B	14.5	15.05	15.29	16.21	17.99	16.44	24.31	21.06
PQLC2	15.58	14.72	14.21	14.59	15.67	16.45	14.02	14.98
TIAL1	13.95	14.37	14.98	14.77	15.84	16.45	20.53	18.74
TMEM198	17.22	15.63	14.52	13.7	15.24	16.46	11.4	13.29
SURF6	15.2	15.05	15.72	16.12	16.83	16.46	15.28	16.36
EFNA1	23.7	23.91	24.31	20.03	18.76	16.46	16.99	17.24
WRB	13.76	12.81	14.02	17.15	15.74	16.47	18.74	17.89
SLC2A6	12.92	14.65	11.89	11.6	13.8	16.48	12.21	13.85
WARS	19.6	18.65	18.54	17.02	15.16	16.48	15.73	14.15
PPP2R5A	15.31	15.09	17.67	18.89	16.04	16.48	19.24	17.89
COL5A2	16.63	16.01	17.2	18.57	16.85	16.48	18.54	19.13
IL13RA1	15.93	15.32	16.58	17.03	17.66	16.48	21.15	19.96
IPO9	16.17	15.71	17.01	17.16	16.87	16.49	19.01	17.59
KREMEN1	17.37	17.47	17.56	17.62	15.74	16.5	14.53	16.17
TBC1D2	14.49	14.83	15.16	13.68	16.83	16.51	14.12	14.2
SLC41A1	15.08	16.75	15.88	15.53	17	16.51	16.88	18.21
METRNL	15.77	15.43	14.77	14.18	13.76	16.52	12.27	12.03
SPPL3	17.33	18.08	16.52	16.85	17.86	16.52	16.54	17.04
SP100	23.33	22.15	25.59	28.52	19.73	16.52	25.49	20.63
DUSP9	34.19	32.6	30.71	26.03	16.12	16.53	11.54	14.39
FBRSL1	15.42	14.81	14.35	14.51	14.46	16.53	12.27	14.41
TES	20.09	17.89	20.65	20.84	17.78	16.53	21.97	21.27
FAM53C	15.76	16.17	16.47	15.4	17.14	16.54	14.94	16.08
RAB14	15.43	14.66	17.01	17.25	17.22	16.54	20.04	18.24
LRRC75B	17.92	17.95	19.14	16.27	14.73	16.55	14.97	14.88
PPP6R3	15.85	14.11	17.65	20.16	18.69	16.55	26.81	23.39
PWP1	15.76	15.15	17.23	17.09	17.67	16.56	20.42	19.07
PTPN11	13.07	12.02	16.04	18.01	17.58	16.56	24.92	21.66
DNLZ	18.22	16	12.41	15.24	15.65	16.57	10.84	12.96
CPOX	15.44	13.29	14.99	15.29	15.22	16.57	17.15	16.36
PWWP2B	15.95	16.71	14.75	13.7	15.06	16.58	10.75	12.64
MAP3K14	17.55	16.98	17.68	16.4	17.16	16.58	14.54	14.95
ALDH1A3	13.94	13.36	14.18	15.86	17.35	16.58	18.73	16.83
RPRD2	17.7	18.14	17.82	18.27	17.58	16.58	19.04	19.52
UBQLN2	19.01	17.29	17.53	18.99	17.78	16.59	18.73	19.13
IER3IP1	17.29	17.47	17.05	20	17.36	16.59	23.06	20.59
INCENP	15.62	13.71	15.09	14.57	16.91	16.6	14.1	15.15
LAT	19.03	18.33	17.84	17.91	17.94	16.6	14.19	16.4

LEPROT	14.69	14.73	16.71	17.69	17.05	16.6	20.31	20.19
SART3	17.7	16.97	17.29	17.24	17.39	16.61	16.98	17.09
UBP1	14.56	14.61	15.04	15.87	16.53	16.62	19.52	18.52
GGNBP2	17.51	15.73	18.24	20.25	19.11	16.62	23.59	21.7
NUDT5	15.58	15.35	17.22	16.17	17.4	16.63	20.31	18.75
FHOD1	15.48	15.73	15.09	13.43	16.73	16.64	14.07	14.71
COMMD1	16.14	17.39	17.76	16.71	17.28	16.64	17.37	16.16
NFKBIL1	16.46	16	13.38	14.88	16.97	16.65	12.23	13.54
AP5S1	14.35	14.39	14.05	13.02	15.89	16.65	13.87	15.36
TMEM131	15.71	14.03	16.63	19.42	17	16.65	21.25	19.72
CMC1	18.48	14.26	16.2	16.19	15.35	16.67	17.13	16.51
ZCCHC3	16.96	17.3	17.51	17.5	16.76	16.69	14.66	14.85
EIF2S1	14.33	14.4	15.51	16.4	18.23	16.69	22.24	19.47
LOC613037	14.89	14	16.85	20.96	16.33	16.69	26.38	21.32
PNO1	16.09	14.08	16.62	15.89	19.15	16.7	20.03	19.15
TRAPPC2L	16.03	14.63	14.89	13.32	16.53	16.71	13.57	14.9
AP4M1	16.77	16.88	16.27	14.97	15.25	16.72	13.64	15.98
PAAF1	16.15	15.79	16.83	16.26	18.29	16.72	15.56	17.11
ADM	20.48	16.06	19.37	17.51	19.54	16.73	19.25	15.04
GINS2	19.06	19.09	18.51	17.08	18.64	16.73	16.26	15.08
LRP8	12.28	12.81	13.26	14.19	16.82	16.73	17.19	18.02
KLHL12	15.62	15.56	15.67	16.69	18.35	16.73	20.27	18.22
DRAM2	17.28	18.1	19.89	18.98	19.43	16.73	22.6	20.89
PSMD12	13.77	14.09	16.46	16.68	17.68	16.75	22	20.12
CDC73	16.25	16.02	17.46	19.81	16.72	16.75	22.17	20.84
CEPT1	15.55	15.65	16.46	17.81	18.08	16.75	23.94	21
CERS6	14.82	13.3	16.88	20.27	17.21	16.75	23.93	21.62
LYNX1	15.24	17.23	15.27	15.05	15.87	16.76	12.79	14.13
ZNF746	15.26	15.41	14.82	15.02	15.73	16.76	12.52	14.36
FCF1	13.93	13.52	15.39	16.64	16.73	16.76	20.48	18.91
NUP50	15.07	14.17	16.25	19.03	17.09	16.76	24.23	19.76
KIFC3	14.49	14.96	14.57	14.67	16.21	16.77	12.53	14.13
CHD1L	15.29	16.15	16.33	16.37	15.91	16.77	18.49	17.49
SGSM3	17.01	17.78	16.13	16.17	15.62	16.78	15.01	14.91
HAUS1	13.14	15.54	16.67	14.86	16.37	16.8	21.5	16.14
SRPK2	14.45	13.55	16.04	17.53	16.56	16.8	21.15	20.49
ORC4	14.34	13.38	15.97	21.03	14.7	16.8	33.5	26.28
DCTN5	16.71	16.4	16.78	17.59	17.75	16.81	20.52	19.54
RNF123	15.73	15.36	14.95	13.92	16.41	16.82	14.37	15.34
CPT2	15.29	17.25	15.74	16.63	18.14	16.82	15.91	16.27
WDR55	16.13	17.59	16.34	16.38	17.76	16.82	16.91	16.29
FCHSD1	16.97	17.98	16.73	17.76	16.99	16.82	16.08	17.11
RECQL5	15.98	16.4	15.69	15.34	17.33	16.83	14.31	15.34
EIF4G3	16.02	14.66	18.22	20.45	19.38	16.83	25.84	23.37
ZC3H3	17.73	18.31	16	15.68	15.77	16.84	14.24	15.01
CDS2	15.98	16.48	15.93	16.63	16.99	16.84	16.81	16.32
DNAJC21	14.15	13.64	16	19	16.78	16.85	23.31	21.93
TIMM21	15.72	15.23	16.79	18.37	19.97	16.85	27.56	22.7
CNOT9	17.3	17.29	18.34	18.37	17.98	16.86	19.85	18.06
SPIRE2	14.66	13.25	13.41	12.64	14.35	16.87	12.28	12.96
SKIV2L2	14.23	13.54	16.35	18.45	16.91	16.87	25.41	22.23
CCDC6	13.08	13.09	14.08	15.6	16.8	16.88	20.84	18.51
FBXO9	14.33	13.33	15.22	15.21	17.86	16.88	21.83	20.69
NPIPB3	14.5	13.69	13.58	14.11	16.89	16.89	17.63	19.79
RELL2	11.53	12.9	11.92	11.01	15.8	16.9	14.31	15.34
ELP3	16.81	16.09	16.52	15.55	17.43	16.9	16.37	16.72
ANKZF1	19.29	17.4	16.51	17.94	15.73	16.91	16.54	16.46
USP48	13.68	12.05	15.16	16.33	17.35	16.91	23.84	20.21
EARS2	14.38	13.98	14.09	13.74	15.98	16.92	15.16	15.65
MRPS10	14.56	13.22	14.75	16.59	16.54	16.92	19.87	17.56
BIN3	17.02	17.64	17.81	17.09	16.03	16.93	15.32	17.81
INTS5	16.12	17	16.41	13.47	15.35	16.94	12.99	14.74
CSTF2	15.58	16.7	17.17	15.8	17.62	16.94	17.39	16.89
DCPS	19.75	19.87	19.21	16.18	17.32	16.94	15.22	17.27
RFWD3	16.98	17.75	17.29	18.09	18.9	16.94	18.91	17.42
H1F0	31.7	32.24	32.33	29.67	19.19	16.94	17.23	17.85
ORC5	13.25	12.42	14.46	15.58	17.49	16.94	22.92	19.1
OSBPL2	17.41	17.41	17.7	18.1	18.14	16.94	19.85	19.12
BET1	13.32	15.28	16.8	16.8	17.27	16.94	23.16	20.5
HGH1	14.96	13.94	13.6	12.58	14.56	16.95	10.81	12.89
DLG5	18.54	19.35	19.43	19.39	16.5	16.95	16.2	17.02
STAG2	15.72	15.34	17.93	18.88	16.28	16.95	25.75	23.89
RBM15B	17.65	18.19	16.51	16.11	16.75	16.97	14.31	15.53
UNG	17.98	17.66	18.38	17.07	17.96	16.97	17.17	16.34
NCOA6	16.6	15.84	16.65	18.54	16.47	16.97	18.52	18.3
BLOC1S2	15.06	17.3	17.17	17.7	20.2	16.97	26.66	21.14
ZXDC	16.34	16.11	18.66	16.16	16.45	16.98	15.5	15.9
KLC4	15.98	17.2	15.51	14.87	18.13	16.98	16.24	15.96
MED27	16.65	18.74	19.08	15.72	18.87	16.98	17.13	17.41
HMGB3	13.31	14.31	15.19	16.18	16.39	16.98	18.84	18.56
PEX5	14.88	15.89	14.88	14.46	17.54	16.99	14.76	14.97
CINP	15.98	15.32	15.92	15.07	17.81	16.99	16.07	15.33
SEPHS1	14.7	13.87	15.74	14.67	18.1	17	17.99	17.1
CEBPZ	15.18	14.75	16.52	18.31	17.94	17	27.46	23.46
IFT43	18.76	17.52	17.08	17.54	15.49	17.02	16.45	17.13
KPNA6	15.46	15.61	16.24	17.42	17.36	17.02	19.25	18.49
SLC2A8	19.21	16.98	17.54	16.49	14.76	17.03	14.03	14.16
KDM2A	17.37	18.33	17.36	17.9	16.21	17.03	16.51	16.1

UQCRB	14.11	16.11	15.15	16.54	17.42	17.03	20.32	18.98
TP53I3	17.74	16.77	16.09	15.82	14.38	17.04	14.31	15.01
UBA3	15.74	14.79	16.91	20.07	18.02	17.05	26.34	23.83
CCDC58	18.19	18.78	19.27	21.94	18.97	17.06	22.24	20.02
AMD1	13.96	14.88	14.02	16.9	17.46	17.07	22.88	19.46
IFI27L1	19.5	20.86	20.98	17.76	17.24	17.09	15.03	17.69
ENOPH1	13.92	13.64	15.33	14.81	17.48	17.09	19.67	18.06
C2orf68	15.56	17.4	18.32	18.09	16.7	17.11	16.61	16.15
MIA3	14.96	14.3	17.68	20.45	17.58	17.11	24.73	23.63
WSB1	17.64	15.93	18.11	22.5	17.52	17.11	29.4	26.94
PRKAR2A	17.21	15.32	16.83	15.65	16.27	17.12	14.97	15.37
PLPP1	18.67	19.09	18.03	17.26	16.32	17.12	16.57	15.9
MS4A4E	16.85	15.24	15.75	17.35	15.77	17.12	13.01	17.31
UBE2V2	15.02	15.62	17.14	16.58	17.8	17.12	26.1	21.4
MAP4K2	15.85	16.51	15.44	15.3	17.09	17.13	14.4	16.09
PIP4K2C	15.61	16.1	16.15	14.22	19.46	17.13	15.75	17.21
RRP7A	17.36	15.81	16.16	15.51	16.94	17.14	14.13	14.84
MBD6	17.96	18.69	18.13	19.06	19.42	17.14	16.96	18
COX11	15.39	14.39	16.85	17.69	17.84	17.15	19.94	17.94
PCGF1	13.74	14.55	13.86	13.18	16.04	17.16	16.15	15.18
PCYT1A	16.17	15.25	16.67	16.09	17.97	17.17	18.35	17.82
BMS1	16.64	14.19	17.86	18.7	18.77	17.17	22.42	21.31
ENAH	17.84	16.96	19	22.52	18.12	17.17	24.54	23.16
TFE3	17.78	18.08	17.73	17.86	17.05	17.18	14.61	17.23
H6PD	21.61	21.53	21.21	21.5	17.88	17.18	15.93	17.55
TAF6L	14.57	15.21	14.46	13.6	15.02	17.19	11.88	13.59
NUDT18	17.6	18.43	17.95	17.08	15.8	17.19	13.43	13.98
MON1B	15.92	16.65	16.29	16.06	17.25	17.19	15.85	16.88
STAT5B	17.99	18.13	17.97	17.41	18.11	17.19	17.31	17.84
RPS27L	16.07	16.67	16.02	17.04	20.26	17.19	22.77	19.94
MARK4	15.77	16.12	14.72	15.59	17.56	17.2	14.24	14.08
SLC19A1	16.9	15.68	15.34	14.67	16.6	17.2	13.76	15.14
HBEGF	13.36	14.43	13.77	14.68	18.46	17.2	17.37	18.17
GNA11	19.38	19.6	18.27	17.92	16.42	17.21	13.19	14.71
MRM2	16.45	17.67	16.32	15.58	18.15	17.21	18.85	17.24
MAPK8IP1	14.8	15.73	14.6	13.69	15.4	17.22	12.88	14.81
CTBP2	17.52	17.85	18.31	19.22	17.66	17.22	19.75	18.91
AMDHD2	14.94	15.17	13.72	13.76	16.18	17.23	13.35	14.03
CMSS1	16.27	15.92	15.09	16.06	17.95	17.24	19.39	19.12
ZC3H14	18.13	14.21	18.2	19.26	18.44	17.24	23.68	20.92
DGAT2	15.02	15.16	14.17	14.07	17.97	17.25	14.26	14.44
FGFRL1	18.24	18.74	18.14	16.93	16.88	17.25	13	15.43
PHF12	19.32	18.82	19.06	19.43	17.37	17.26	18.82	18.41
ARHGAP29	14.56	13.95	17.85	20.57	18.24	17.26	30.71	24.92
C3	18.62	18.32	17.43	16.76	17.55	17.27	15.76	15.65
TMEM60	17.62	17.01	16.9	17.33	19.78	17.27	22.24	20.8
IP6K1	17.22	18.07	17.84	16.68	17.11	17.28	15.09	16.2
JAM3	19.07	18.22	18.64	20.37	18.92	17.28	19.56	18.17
ALDH9A1	19.15	18.05	18.55	18.58	19.94	17.28	18.63	18.76
TSPAN31	16.72	16.21	16.15	17.43	18.04	17.29	19.2	20.22
MXI1	16.21	15.54	17.41	18.38	18.69	17.3	21.56	19.51
LANCL1	15.63	13.81	16.56	18.89	17.76	17.31	24.85	22.44
MKNK1	18.04	16.89	16.67	17.27	17.63	17.32	20.31	19.65
MARK2	17.77	16.36	16.39	15.74	16.67	17.33	14.01	15.55
CERS4	18.08	18.21	16.78	16	18.65	17.34	15.48	16.98
HEATR1	16.07	14.7	17.1	17.72	18.8	17.34	22.99	20.27
PI4K2A	17.03	17.59	15.89	16.22	17.29	17.35	16.24	16.78
MCM5	22.71	20.74	19.8	19.35	17.89	17.36	13.42	13.52
PUSL1	15.76	15.36	13.82	14.64	16.09	17.37	12.14	12.14
ALKBH6	18.97	18.84	17.01	16.64	15.52	17.37	18.44	15.45
RBP1	25.94	28.65	24.26	23.33	17.27	17.37	14.38	16.8
OVCA2	13.06	15.61	14.53	14.53	15.82	17.38	11.46	12.85
EIF2B3	16.7	17.93	16.96	16.87	18.15	17.39	18.29	17.85
RBBP9	16.79	15.2	17.35	17.95	17.91	17.39	20.51	19.31
C1RL	16.59	18.13	17.79	19.89	16.03	17.4	19.78	18.66
HIBADH	14.64	14.49	15.83	17.49	17.79	17.4	21.47	19.18
YIPF5	16.28	15.8	17.71	18.66	17.08	17.41	22.41	19.24
MFAP1	18.9	20.23	21.84	21.78	17.91	17.41	20.22	20.3
SLC35A2	17.39	18.57	17.64	17.81	17.48	17.42	15.8	17.97
ELAVL1	17.97	18.22	17.55	16.39	16.06	17.43	15.23	15.31
FANCI	15.22	13.66	15.98	16.65	18.29	17.43	22.46	18
RALB	18.48	19.62	19.8	18.8	19.39	17.43	21.21	19.8
TANGO2	14.86	16.15	14.49	15.35	15.65	17.44	15.26	15.64
SPATS2	16.2	15.13	16.97	17.3	17.89	17.44	21.89	19.7
IFI44	27.78	28.84	29.12	34.93	17.11	17.44	31.06	20.64
WDR11	15.89	15.8	19.64	20.47	18.22	17.45	21.98	18.67
USP21	16	17.42	16.4	16.91	16.25	17.46	15.85	15.89
PMS2	16.34	15.51	15.21	16.12	17.93	17.46	18.75	17.7
EXOSC7	15.38	16.67	17.45	18.69	19.01	17.46	18.05	18.83
REEP5	16.85	15.58	18.11	18.96	17.56	17.46	25.37	21.77
ZBED5	14.77	14.25	15.83	19.16	18.02	17.47	29.42	24.97
DDX46	13.8	10.8	15.87	18.99	17.81	17.48	25.03	23.57
ZMYM3	19.73	20.58	19.18	20.3	16.59	17.49	16.23	16.71
XPO5	15.61	15.94	15.94	15.82	18.59	17.49	19.83	17.73
GALT	16.06	16.14	16.26	14.88	17	17.5	15.85	17.14
APOO	17.74	13.64	16.25	15.56	16.1	17.5	18.45	17.22
ANO8	20.57	20.5	20.63	19.96	14.68	17.51	14.15	16.96
ARIH2	16.54	16.74	16.85	16.78	18.95	17.51	19.66	19.1

RNF19B	16.38	17.53	18.23	17.04	17.72	17.52	15.03	14.6
SLC44A1	17.08	16.17	20.4	21.48	16.6	17.52	23.43	19.75
WWP2	18.26	17.59	17.03	16.71	17.41	17.54	15.63	15.3
HMHA1	18.1	18.52	17.09	16.31	16.89	17.55	13.55	14.05
PC	14.14	16.7	15.39	14.01	16.44	17.55	12.9	14.95
PGS1	20.71	19.64	18.25	19.43	17.72	17.55	18.23	17.46
ABLIM1	14.18	13.68	15.02	16.21	18.59	17.55	21.37	19.68
SMC4	14.64	14.42	16.99	19.18	17.77	17.55	31.14	25.73
NGDN	16.74	16.8	17.33	19.23	16.96	17.56	20.35	21.23
KCTD2	19.04	18.24	17.31	17.52	18.26	17.58	15.62	17.76
POLB	18.9	16.15	16.71	16.62	16.88	17.58	20.09	18.12
NAMPT	15.74	15.49	17.52	20.3	18.82	17.58	27.72	23.58
GCHFR	19.31	19.36	18.82	16.96	17.49	17.59	14.49	16.33
TRIO	17.48	16.55	17.35	18.23	16.94	17.59	19.09	17.9
FAM104A	17.13	16.48	17.17	17.12	16.69	17.59	16.55	18.27
RFC4	17.11	15.65	18.93	20.45	18.67	17.59	21.85	19.05
EEFSEC	20.31	21.49	19.81	17.76	15.95	17.6	13.96	13.79
GNAI1	16.64	16.01	17.98	20.04	19.57	17.6	27.48	24.92
PJA1	20.01	19.72	18.57	18.5	17.59	17.62	18.94	18.76
PCDHB5	20.21	19.68	20.12	20.54	16.9	17.64	16.69	16.51
TRIM44	17.33	17.41	19.24	19.78	19.62	17.64	22.45	21.23
TMEM35B	18.61	17.49	18.74	19.92	17.84	17.65	17.26	16.67
CHIC2	13.13	11.66	13.19	14.36	11.99	17.66	13.58	13.96
STX1A	19.35	18.67	19.39	20.28	19.75	17.67	17.34	17.08
METTL3	18.47	17.21	17.23	17.46	16.87	17.67	20.04	17.63
SRP54	17.11	15.47	18.5	20.53	17.44	17.69	26.15	22.07
PTPMT1	18.27	16.92	16.77	17.06	16.32	17.7	15.79	16.32
SESN2	16.7	17.2	16.43	14.73	17.11	17.71	15.48	15.86
MLH1	17.64	16.35	17.52	17.34	18.01	17.71	19.81	18.16
GLRX	15.91	17.77	17.02	15.26	17.3	17.71	18.63	18.94
PRPSAP2	17.06	16.74	17.46	16.59	18.25	17.72	20.45	19.46
LYRM1	16.98	14.71	16.83	16.03	18.98	17.72	23.01	20.39
ARAP3	17.38	18.26	18.43	17.21	16.62	17.73	16.25	16.48
ZFAND1	18.2	17.62	16.31	16.16	18.93	17.73	24.32	22.7
PARD6G	18.24	16.72	17.34	16.9	17.38	17.74	14.84	15.27
PNMA1	17.42	15.94	17.82	18.04	18.27	17.74	17.3	18.57
JKAMP	16.49	17.95	17.86	20.54	20.29	17.74	28.04	23.49
EHD4	19.62	20.32	19.14	18.46	18.85	17.75	16.23	16.41
USO1	14.7	13.93	16.52	18.7	18.29	17.76	27.87	24.28
RPF1	15.69	14.82	16.56	18.71	18.49	17.77	22.54	19.24
PJA2	16.12	14.67	19.67	21.57	17.8	17.77	27.63	24.23
PAPD7	16.66	16.5	17.29	19.35	18.4	17.78	20.69	18.75
ARRDC2	14.84	13.45	13.95	14.58	17.64	17.79	16.19	16.27
CLN6	18.04	17.41	18.86	16.51	18.21	17.8	16.57	16.45
ARPC5L	16.15	14.85	15.12	15.42	17.01	17.81	17.27	17.66
DDX11	15.89	15.32	15.18	15.87	18.38	17.82	19.63	17.85
CUL7	22.18	21.06	20.66	20.2	16.93	17.83	15.34	15.61
MRC2	32	33.43	30.7	28.33	15.56	17.83	14.27	15.69
KAT8	18.99	20.46	16.31	16.27	18.98	17.84	15.48	16.38
CCDC97	18.84	20.43	17.83	16.25	18.39	17.84	15.25	17.02
SUCLG2	16.61	13.93	16.79	17.93	18.7	17.84	21.6	20.73
MAN2B2	17.03	17.05	17.03	16.54	15.41	17.85	15.79	16.13
LARP6	19.28	19.92	18.4	19.1	18.19	17.85	17.92	17.95
DVL2	18.02	19.9	17.39	17.09	17.1	17.86	13.79	15.26
AACS	16.73	18.29	17.58	16.14	16.35	17.86	14.5	15.88
DYNC1LI1	15.38	15.01	15.6	16.15	17.65	17.86	20.47	19.99
ZER1	17.21	17.62	17.1	16.58	16	17.87	13.81	15.48
CYB5R1	18.07	20.11	17.82	17.52	18.75	17.88	17.67	19.25
FAM96A	16.95	16	15.71	16.92	19.03	17.88	23.04	20.53
MRPL33	19.61	18.66	15.48	17.73	20.77	17.88	22.79	21.34
UBE2F	13.26	14.12	14.84	14.73	17.99	17.89	20.73	19.76
CRYZ	17.84	17.26	19.31	21.36	19.69	17.89	29.12	23.9
EHMT1	18.89	19.34	18.09	18.53	18.14	17.9	16.07	17.68
TMCO1	18.53	17.82	18.71	19.19	19.99	17.9	22.06	21.89
ATP6V1D	16.41	16.83	17.18	17.89	17.93	17.91	20.97	19.94
EFNA4	21.79	20.91	18.32	19.32	18.21	17.92	14.32	15.24
GTPBP2	20.39	18.97	17.77	18.8	18.65	17.92	17.61	18.49
CMTR1	21.91	22.13	20.55	19.47	18.05	17.94	16.82	16.64
PFKFB3	19.19	18.93	19.04	18.6	17.27	17.95	17.31	17.37
RNF26	18.45	18.58	16.31	15.86	18.87	17.96	14.37	16.86
FCGRT	21.09	25.44	21.45	18.95	17.47	17.97	14.62	15.03
KMT5A	14.09	14.28	16.08	16.41	16.27	17.97	17.94	18.17
CNOT8	14.81	14.47	15.41	16.63	16.43	17.97	20.4	19.1
NUDT16	14.04	15.62	16.4	15.43	17.11	17.99	14.45	14.91
GLI4	16.34	14.29	14.73	15.12	18.07	17.99	14.74	15.77
GOLIM4	16.9	16.18	18.84	23.2	19.65	17.99	27.88	24.13
TTC3	14.74	13.97	18.04	22.48	17.15	17.99	29.04	26.11
SIRT3	18.72	19.25	17.68	17.49	17.09	18	16.3	16.51
AFMID	16.34	15.07	16.54	16.13	17.21	18	17.98	18.34
HDDC2	17.29	17.86	18.65	17.88	18.87	18	19.91	18.89
WDR83	18.2	17.19	16.01	14.2	17.03	18.01	13.57	14.5
MMP11	23.55	23.04	23.02	21.77	18.36	18.01	12.89	15.12
MECR	15.96	17.81	16.29	15.81	18.43	18.01	15.34	17.28
PPP1R37	17.76	17.46	16.62	14.73	16.38	18.03	12.82	14.43
MRRF	15	14.76	16.52	14.64	17.93	18.03	19.93	20.08
YIPF1	17.53	18.74	18.02	18.19	18.26	18.04	19.74	17.28
NXF1	17.26	17.68	17.42	17.29	19.36	18.05	20.13	18.36
ST6GALNAC6	17.23	20.4	18.64	18.2	18.28	18.05	17	18.58

ZSWIM8	16.66	17.27	17.19	16.22	16.73	18.06	15.55	16.85
DMRTA2	19.87	18.56	17.62	17.2	16.61	18.07	14.55	14.39
MAD1L1	19.19	19.04	18.36	17.62	17.67	18.07	14.9	15.83
XPR1	15.18	14.66	16.72	18.53	18.43	18.07	24.23	22.54
OGFOD1	16.77	16.73	17.44	17.83	19.26	18.08	21.16	18.09
NFATC2IP	17.82	17.17	16.91	17.81	18.91	18.08	21.51	19.8
NCBP1	14.97	12.39	16.91	18.69	19.28	18.1	24.34	20.84
CCDC167	16.81	16.58	17.23	14.82	18.16	18.11	14.57	15.4
CERK	15.41	15.18	15.07	14.61	17.45	18.11	16.6	17.96
QSOX2	13.57	13.93	14.09	14.71	17.18	18.12	18.15	17.92
PDCD6IP	16.8	15.4	18.65	18.86	19.46	18.12	26.76	22.79
MIB1	16.65	14.87	18.61	21.35	16.71	18.12	25.25	23.78
TRRAP	18.29	18.42	18.57	18.81	17.23	18.13	18.54	17.96
ITGB3BP	18.57	18.09	19.98	18.78	21.12	18.13	25.45	22.51
JAGN1	21.99	18.85	19.74	16.71	17.44	18.14	17.71	17.46
ATP6V0A1	15.37	15.5	15.38	15.98	17.87	18.14	18.79	18.82
TWF1	17.17	16.62	17.59	18.42	18.78	18.14	28.02	23.65
EHD1	18.62	18.93	17.57	16.73	17.46	18.16	13.63	14.92
TBC1D1	17.37	16.72	17.41	16.82	18.66	18.16	18.36	17.97
SDHD	17.02	14.58	15.49	18.04	17.8	18.16	23.5	20.64
LOXL2	26.02	25.55	23.63	24	17.46	18.18	15.57	16.75
ZNF337	15.13	14.13	17.26	19.53	17.49	18.18	26.04	21.94
QPCTL	17.31	16.82	15.51	15.84	15.53	18.19	12.85	16.23
GFM2	15.95	15.6	15.74	17.81	19.39	18.19	23.67	20.93
ANAPC16	18.23	17.35	18.02	18.74	18.72	18.2	19.26	18.57
JMJD4	15.8	16.17	15.4	15.04	15.89	18.21	12.7	13.56
PPHLN1	15.75	15.64	16.6	18.55	18.95	18.21	25.23	21.64
SLU7	15.1	14.35	18.27	20.31	19.03	18.21	26.99	21.83
SYS1	13.95	13.76	13.12	12.01	17.08	18.22	15.6	15.59
ARHGEF2	21.97	22.94	22.6	21.55	17.56	18.22	16.59	18.4
EHD3	18	17.66	17.53	17.12	17.51	18.23	15.64	17.09
ZC3HC1	16.48	17.25	16.61	14.44	18	18.23	17.86	17.88
NUP133	16.24	14.82	16.53	17.33	17.74	18.23	21.75	19.53
CBFB	15.51	14.48	17.93	20.11	18.59	18.23	22.51	21.57
PALMD	18.23	17.03	18.37	20.32	18.52	18.23	23.82	23.39
FADD	18	18.78	19.13	15.43	17.83	18.24	15.43	15.48
NSMCE2	15.53	15.41	14.81	16.77	19.21	18.24	20.39	17.06
HOOK2	16.17	18.78	17.72	18	18.84	18.24	17.64	19.4
AHDC1	20.54	19.89	19.93	19.25	17.03	18.25	14.41	16.26
RILPL1	16.46	16.74	16.17	17.11	19.69	18.25	16.67	17.92
PARN	16.48	16.51	18.55	19.26	18.51	18.25	21.93	20.74
EIF1AX	15.58	14.86	17.13	18.2	19.65	18.26	26.34	22.75
NTMT1	19.62	19.31	17.36	16.96	18.75	18.27	13.69	15.88
HIST3H2A	16.94	14.79	19.3	15.84	15.99	18.28	14.11	11.17
NUP37	15.02	15.6	16.4	16.33	16.98	18.28	21.63	16.31
HIST1H2BK	21.53	23.65	24.02	21.03	18.61	18.28	18.13	16.95
IPO13	15.73	16.14	15.58	14.94	17.84	18.28	16.62	16.99
MRPS35	17.93	17.29	18.33	19.01	19.82	18.28	26.53	22.52
RMND5B	16.53	16.69	16.09	14.3	15.98	18.29	15.17	14.23
BCAS3	18.91	17.82	19.54	19.12	16.97	18.29	16.63	17
HTATSF1	16.82	14.3	17.3	19.58	18.82	18.29	22.65	21.42
CCDC115	21.22	18.7	19.57	17.56	18.95	18.31	16.83	15.74
PHRF1	19.49	19.64	19.09	17.37	17.95	18.31	14.9	16.31
PALLD	19.27	18.18	19	19.49	19.43	18.32	21.15	19.29
UBAP1	18.27	17.09	17.91	19.34	17.6	18.33	17.13	18.42
FSTL1	20.62	19.3	21.1	22.33	19.6	18.33	22.75	21.39
MORC2	18.95	19.98	19.51	19.94	17.03	18.34	17.82	19.42
PHF19	16.77	16.01	16.38	16.21	18.32	18.35	17.05	14.91
SCLY	15.64	15.85	16.35	15.13	18.88	18.35	16.5	15.36
RARS2	16.48	15.84	14.36	17.05	17.42	18.35	20.68	18.24
MED29	20.02	18.48	19.19	18.96	18.63	18.36	17.47	16.69
ST3GAL2	19.52	20.54	19.55	18.74	17.55	18.37	14.27	16.33
HCFC1	19.33	19.84	17.97	18.54	17.36	18.37	14.75	17.38
R3HDM2	17.19	17.08	17.61	19.58	18.07	18.37	19.89	20.37
STXBP1	17.31	18.47	17.46	17.37	17.31	18.38	16.69	17.74
SH3GLB1	16.75	16.7	19.46	17.93	16.6	18.38	20.22	20.6
TEAD1	14.88	13.13	17.72	21.65	19.21	18.38	29.53	25.32
MSH6	21.49	19.36	22.22	23.87	19.85	18.39	25.42	23.49
CABIN1	18.94	18.81	19.17	18.77	17.52	18.4	15.68	17.56
DHX16	18.09	19.29	18.3	18.93	18.41	18.4	18.29	17.59
AMOTL1	18.6	17.47	18.56	17.55	18.67	18.44	18.8	17.13
FOSL1	18.21	16.38	16.61	14.42	18.3	18.44	16.06	17.75
CDK2	20.73	19.45	19.91	21.42	19.01	18.44	19.98	20.63
YPEL5	17	16.84	18.43	19.44	20.46	18.44	22.94	22.27
TESK1	19.12	19.49	17.66	17.3	17.15	18.45	15.82	17.07
PABPN1	19.99	18.08	18.44	19.57	18.99	18.45	15.02	17.27
TMEM167A	17.41	17.41	18.44	21.44	19.25	18.45	27.5	25.32
DKK1	9.11	8.28	10.93	10.97	21.01	18.46	24.39	18.5
ARID1A	18.53	18	18.68	19.72	17.41	18.46	18.16	18.95
BRD8	15.93	15.61	16	16.86	19.37	18.46	22.31	19.79
NT5C2	17.13	15.66	17.3	20.59	18.75	18.46	23.77	20.9
ELMO2	18.89	18.67	19.13	18.65	18.62	18.48	20.31	19.01
CLDND1	16.41	17.15	19.58	22.54	21.06	18.48	29.52	25.37
ALG1	16.93	16.28	16.33	15.31	17.62	18.49	17.69	15.77
TAF1D	13.45	13.95	14.81	17.54	16.54	18.51	29.89	23.45
HSD17B8	19.56	20.63	18.09	18.79	19.05	18.52	14.01	14.87

SMIM11B	15.79	14.96	15.3	15.16	15.94	18.52	15.64	15.57
MBD1	17.44	14.76	16.42	17.38	17.07	18.52	17.03	16.79
GLOD4	16.59	15.81	15.67	15.25	18.07	18.53	17.1	16.73
MINOS1	17.82	18.4	18	18.04	19.2	18.54	21.18	19.88
SLC10A3	19.63	20.25	17.06	17.09	18.78	18.55	14.96	16.82
SYT11	16.9	16.62	16.13	16.59	19.84	18.55	18.27	19.08
RAB11A	17.69	18.24	18.59	19.05	18.38	18.55	21.13	20.45
RTCA	15.79	16.13	17.59	19.82	19.32	18.57	24.43	20.81
XXYLT1	18.79	18.23	17.99	19.51	19.42	18.58	17.67	18.64
44447	17.94	18.04	17.73	18.85	18.12	18.58	18.31	18.99
YLPM1	19.83	19.88	20.03	21.57	18.81	18.6	21.23	21.55
RASSF10	17.97	16.03	16.81	17.03	16.98	18.61	14.28	15.86
ATAD1	14.8	16.27	18.19	18.83	20.06	18.61	27.35	22
SEMA4F	18.51	20.29	18.95	17.87	17.47	18.63	16.31	17.03
TMEM8B	20.19	20.41	19.18	19.6	18.83	18.63	16.48	19.03
PITPNA	18.34	19.16	17.89	18.3	19.12	18.64	18.86	17.62
ZNF414	19.88	19.01	17.14	18.12	17.8	18.65	12.99	14.68
REXO4	15.98	17.47	17.31	16.86	19.32	18.65	17.1	17.98
BTG3	16.41	14.41	17.47	17.68	22.7	18.66	26.6	20.97
MFHAS1	19.33	17.89	19.42	17.54	16.66	18.67	15.32	17.28
MTFR1L	14.81	16.4	15.43	14.84	18.66	18.68	18.31	18.41
PFDN4	16.8	17.85	17.68	17.92	20.75	18.69	25.01	22.23
SMN1	15.45	19.37	20.47	19.48	22.08	18.69	27.36	22.48
MCM3AP	18.14	18.18	18.03	18.57	19.51	18.7	18.09	18.68
ASTN2	14.35	13.73	17.73	21.62	16.13	18.7	31.8	28.73
KCTD5	17.96	17.08	19.07	17.49	19.22	18.74	16.12	16.35
ZNF385D	19.43	17.45	18.49	21.6	19.31	18.74	21.69	20.79
CTXN1	17.56	17.81	16.39	15.22	18.69	18.76	10.79	15.44
PORCN	15.36	16.73	15.41	14.71	18.27	18.77	16.76	17.18
FBXL18	17.81	17.73	16.83	16.1	18.53	18.77	16.55	18.62
PPP3CB	16.55	15.39	15.82	17.07	19.72	18.78	22.25	20.35
RALBP1	16.68	16.04	16.52	17.16	20.14	18.78	20.21	20.39
GTSF1	21.76	23.91	23.72	22.2	21.82	18.79	21.63	21.27
HMGXB3	18.07	19.31	17.37	17.69	19.29	18.8	17.46	18.14
FBXW11	18.11	17.18	18.93	19	20.41	18.8	21.77	21
PCDH7	18.8	16.55	19.2	19.65	17.38	18.81	17.78	18.98
FGFR1	21.12	22.76	20.02	20.97	16.3	18.82	16.11	17.04
MGRN1	17.13	18.16	18.1	17.11	18.25	18.82	14.55	17.29
APEX2	20.61	21.88	18.58	19.94	19.78	18.83	18.15	16.52
TGFA	21.84	20.13	21.07	21.87	19.96	18.83	21.18	18.63
UBE2N	17.39	15.72	17.49	19.85	18.46	18.83	22.39	19.99
DBNDD1	18.58	18.14	17.51	17.63	17.7	18.84	15.72	17.57
ALDH3A2	16.33	15.85	17.08	16.7	18.7	18.84	21.34	20.1
C14orf1	19.73	20.03	21.22	20.79	19.38	18.84	19.99	20.12
CHKB	18.13	20.24	18.45	17.81	18.54	18.85	20	18.44
CXCL8	14	15.28	16.6	16.46	22.69	18.85	30.08	22.48
ARRB2	15.71	17.29	16.55	16.02	17.66	18.86	15.41	17.98
IL10RB	20.3	21.7	20.4	19.34	19.29	18.87	19.62	19.77
SNAP23	17.23	15.96	18.94	20.45	19.86	18.87	23.42	22.11
COQ4	15.99	17.28	15.38	16.98	18.67	18.91	14.72	17.34
SLC37A2	19.53	20.12	19.67	20.08	17.16	18.91	18.85	17.9
PRPS1	18.39	18.3	18.25	18.69	19.13	18.91	20.42	17.98
PHF13	21.13	22.37	20.63	20.17	20.16	18.92	17.75	19.7
NNT	16.41	14.87	17.02	17.74	19.19	18.92	22.01	19.88
ITPK1	17.19	18.62	16.44	16.05	17.78	18.93	14.78	15.38
OPTN	17.27	17.35	18.84	19.79	20.29	18.93	23.05	21.95
HGSNAT	19.39	19.06	20.11	20.69	19.04	18.94	21.2	20.1
EPDR1	19.75	19.39	18.83	20.97	19.31	18.95	18.21	18.99
RSAD1	17.26	17.34	17.3	17.78	19.63	18.95	19.84	19.76
PRPF38B	15.29	14.68	17.28	21.28	18.33	18.95	24.99	22.99
PRKRIP1	17.55	19.34	17.52	17.4	19.64	18.96	18.82	18.22
VHL	20.02	19.1	19.8	19.91	19.4	18.96	20.22	19.1
MAGT1	20.7	19.06	21.63	22.98	19.59	18.96	23.95	23.2
TEN1	17.5	18.5	17.54	16.35	17.23	18.97	15.68	14.65
GGCX	16.93	18.39	17.44	16.31	17.97	18.98	15.37	16.39
CHCHD6	19.77	19.59	18.52	16.58	21.32	18.98	18.06	16.86
NDUFV3	16.43	16.9	16.86	17.23	18.41	18.98	17.59	18.13
UBR5	17.66	16.22	19.02	21.21	19.11	18.98	24.26	23.06
DGCR14	16.74	15.61	16.15	14.37	18.68	18.99	15.64	15.65
BCL9L	20.38	21.64	20.52	21.48	17.15	19	15.18	17.61
NEK9	18.41	19.23	20.5	19.94	19.42	19	22.35	21.35
RAB5B	19.7	20.87	19.16	20.35	19.24	19.01	19.73	21.54
FRG1	21.35	17.91	21.91	22.61	21.45	19.01	25.38	23.08
HIP1	18.93	19.49	19.52	20.39	19.57	19.02	18.74	19.8
SNRPA1	15.41	15.83	16.02	19.27	17.47	19.02	23.43	20.66
SPRYD3	19.68	20.69	18.22	18.09	20.23	19.04	15.78	17.09
ACAP3	18.55	17.44	17.18	15.82	18.33	19.06	17	17.64
PRR11	17.23	15.34	17.85	17.58	19.45	19.06	20.82	20.79
MBNL1	15.39	14.04	17.43	22.69	20.61	19.06	35.09	28.97
HPS6	18.29	17.86	17.43	16.08	17.25	19.07	12.83	14.49
GMNN	18.49	18.58	19.6	18.86	21.46	19.08	25.14	21.93
RHOB	17.19	16.91	17.42	14.45	16.56	19.09	14.65	15.74
TLDC1	20.41	20.08	19.43	18.21	19.65	19.09	16.04	17.49
DTNB	18.29	20.09	20.12	21.13	18.22	19.09	22.51	21.15
MPDU1	17.97	19.02	18.88	17.29	19.03	19.1	15.22	18.95
ANO1	18.38	18.68	18.93	21.14	18.35	19.12	19.32	18.73
ABHD16A	19.63	17.85	18.28	17.67	18.25	19.12	15.99	18.89
FRMD8	19.95	20.6	17.49	17.56	16.21	19.13	15.57	16.61
IFT52	19.7	18.92	19.77	19.42	18.59	19.13	21.95	19.9

CEBPZOS	17.67	16.02	18.17	19.07	21.15	19.13	24.65	25.94
NUDT2	17.04	17.33	17.08	16.48	18.98	19.14	18.69	20.25
IGF2BP3	19.15	15.78	19.72	22.64	19.89	19.16	29.01	23.78
TTI1	19.56	19.28	18.94	19.62	20.28	19.17	21.92	19.77
NDUFAF2	19.36	19.24	16.28	16.78	24.94	19.17	24.01	20.66
PRKRA	14.5	15.14	16.78	15.5	20.38	19.17	24.62	21.34
CENPN	14.65	14.45	13.89	15.52	17.15	19.18	18.9	17.17
SLC20A2	18.18	16.63	16.38	18.17	18.82	19.18	18.51	19.05
CCDC74A	18.95	18.19	19.63	17.81	17.46	19.19	18.56	18.4
ENTPD4	17.01	16.64	19.14	21.05	19.33	19.19	25.93	23.51
DAPK3	19.87	19.28	19.23	18.13	18.35	19.2	13.09	16
PATZ1	20.49	20.94	19.85	19.04	18.13	19.2	15.79	17.13
RHBDD3	20.29	19.27	18.8	18.05	18.88	19.2	17.21	18.86
ZFAND6	17.86	18.05	21.54	22.86	18.74	19.2	25.69	25.16
PAPSS1	19.23	19.54	20.65	19.78	20.21	19.22	21.97	20.7
RING1	23.84	22.91	21.78	20.79	19.02	19.23	16.96	17.11
HECTD3	17.92	17.27	17.95	17.61	18.26	19.23	16.78	18.36
GLE1	16.81	16.88	17.67	17.84	19.71	19.23	18.08	18.39
TSSC1	18.82	18.71	19.93	17.06	21.05	19.23	16.93	18.48
SHISA2	19.85	20.56	20.19	22.16	17.27	19.23	19.98	20.48
RBBP8	17.67	18.06	20.43	21.22	19.81	19.23	30.44	25.77
SRRM1	19.73	20.51	20.93	24.51	20.83	19.26	25.89	24.79
TMEM223	21.2	20.39	20.39	18.07	18.69	19.27	15.48	18.89
SRCAP	18.7	19.27	18.61	19.45	18.96	19.29	18.1	19.59
SH3BP4	20.7	22.53	22.73	21.84	19.51	19.29	19.44	19.63
EIF2AK4	17.93	16.92	18.8	18.91	19.23	19.29	21.5	20
UBE2G2	16.8	16.46	17.2	18.22	19.02	19.29	25.53	23.14
PARP10	23.19	20.13	20.51	19.96	18.85	19.3	16.47	15.2
UBE2O	20.68	21.2	20.93	19.84	18.26	19.3	16.28	17.2
HBE1	21.58	21.78	19.44	19.36	16.94	19.3	16.58	19.19
PRPF4	19.37	20.22	18.47	19.54	20.83	19.3	21.25	19.41
RNF215	19.04	18.6	16.74	17.73	18.11	19.31	17.41	17.21
ANAPC2	19.16	18.98	18.63	16.99	19.82	19.32	16.31	17
NMRK1	20.57	20.14	21.05	21.87	21.1	19.32	27.36	24.29
ZNF579	21.29	22.44	19.86	18.54	17.79	19.33	12.82	14.46
MUS81	17.76	17.58	17.44	15.67	18	19.33	15.16	16.34
LPAR1	19.71	17.77	21.49	22.69	20.38	19.33	26.68	22.57
CDK7	16.44	17.84	17.68	19.79	20.58	19.34	26.4	21.36
TATDN1	16.51	18.49	17.96	18.18	20.84	19.34	27.67	25.26
PCMTD2	17.7	16.93	17.96	22.65	20.6	19.34	28.72	25.27
SDK2	17.81	18.6	17.85	18.51	16.96	19.36	16.35	18.01
TUT1	21.29	20.39	20.22	19	19.62	19.36	15.88	18.74
DCAF13	16.12	16.95	17.69	17.39	20.21	19.36	25.23	22.96
RNF13	17.46	17.21	18.74	20.46	19.61	19.36	29.81	25.66
PPP1R8	16.49	17.22	19.04	16.13	17.19	19.37	19.39	18.31
MTX2	17.79	17.14	17.15	18.91	19.07	19.38	22.11	20.45
PITHD1	15.28	15.78	16.6	18.13	19.54	19.39	20.13	19.85
RITA1	15.84	16.63	16.71	14.89	19.03	19.4	15.63	16.41
RRS1	16.92	15.77	16.79	14.13	19.71	19.4	17.61	17.28
SH3BP5L	18.19	18.69	18.75	16.87	17.94	19.4	16.29	17.35
HDAC7	18.94	19.95	19.95	19.58	19.36	19.4	18.78	18.39
DCAF12	17.67	18.58	19.72	19.08	20.21	19.4	19.3	20.5
PKMYT1	20.24	17.6	19.93	16.49	20.56	19.41	14.45	14.04
TYSND1	17.53	16.94	17.5	15.42	17.59	19.41	14.75	16.11
ADPRHL2	22.46	22.69	20.25	19.28	20.18	19.41	16.65	18.33
HDGFRP3	19.28	17.46	19.27	21.41	20.45	19.41	25.72	22.89
DDX19A	17.13	17.35	16.75	16.73	19.89	19.42	18.88	18.17
SYF2	17.62	19.45	18.38	19.88	22.24	19.42	27.57	24.22
SMDT1	19.63	20.49	19.08	16.15	18.81	19.43	14.04	17.81
SLC25A23	21.86	24.2	22.1	22.03	18.6	19.43	15.73	18.87
WDR61	18.79	16.96	19.1	18.48	20.23	19.43	21.78	20.07
ODF2	20.16	18.46	20.25	21.64	20.19	19.43	21.03	21.06
RXRB	19.4	20.42	19	18.47	19.39	19.44	18.39	18.74
PPP1R2	16.94	18.71	18.27	21.2	18.8	19.44	17.15	19.3
LUZP1	19.12	18.91	19.79	19.94	19.99	19.44	20.61	21.65
MAN1A2	17.37	19.35	20.02	24.12	21.76	19.44	27.3	28.14
FAM171A2	25.61	26	24.02	21.19	17.53	19.45	12.68	15.64
ASH2L	21.33	20.5	21.03	20.3	20.68	19.45	20.9	19.53
LRRFIP1	16.73	15.32	19.7	23.41	20.43	19.45	30.45	28.12
UHMK1	16.57	13.93	19.71	22.29	19.57	19.46	30.37	26.89
TTC37	16.42	14.64	19.24	22.05	19.38	19.47	33.18	28.45
PDS5A	15.93	14.97	18.52	21.44	18.91	19.48	28.17	23.41
CLSTN3	23.81	24.66	23.6	22.14	18.7	19.49	17.48	19.51
MTOR	17.7	17.31	16.82	17.69	19.16	19.49	19.69	20.46
PITPNB	15.67	15.83	17.35	18.6	20.28	19.49	24.69	20.48
MRFAP1L1	19.34	19.07	21.88	23.07	19.73	19.5	23.45	22.17
TNK2	19.41	19.9	19.02	19.35	18.51	19.51	16.6	18.01
ZCCHC17	19.69	20.13	18.18	18.88	17.96	19.52	19.17	19.95
ZKSCAN1	18.78	16.57	21.7	25.06	18.68	19.52	26.6	24.92
C19orf52	17.95	17.27	18.55	15.64	20.36	19.54	14.43	16.53
CYTH1	19.01	19.61	19.47	20.45	19.37	19.54	19.38	19.17
SLC12A4	15.76	16.63	15.86	16.69	19.08	19.54	18.04	19.3
LARP4B	18.89	18.72	18.82	20.86	19.34	19.54	20.9	21.43
ERCC3	18.64	18	17.64	16.99	19.59	19.55	19.65	17.85
MSANTD3	17.01	17.59	18.17	19.2	20.82	19.55	21.02	19.74
ANAPC7	16.27	16.71	17.24	18.69	20.07	19.55	20.92	21.05

CRTC2	20.82	20.93	20.19	19.59	20.54	19.55	18.98	21.25
DCTN4	18.18	17.5	18.23	19.62	22.1	19.55	24.56	22.37
NEK6	19.78	20.41	19.82	19.45	19.61	19.56	17.86	18.3
PRSS23	17.01	18.54	16.94	19.26	21.76	19.56	22.34	18.4
PTPN23	20.9	21.08	19.72	18.73	18.39	19.56	15.62	18.72
MED19	18.77	20.35	17.06	16.84	19.28	19.56	16.71	18.9
KDM3B	20.2	20.24	20.88	20.53	19.11	19.56	20.14	20.99
MACF1	17	16.86	18.54	19.86	19.91	19.56	24.24	22.77
PARD3	20.02	20.48	21.1	20.31	20.43	19.57	21.17	22.11
SAR1A	19.01	17.03	20.04	21.21	19.81	19.59	23.71	22.19
RACGAP1	17.15	16.63	17.05	14.13	18.94	19.6	19.63	18.16
GGA2	21.57	21.5	21.94	22.54	20.31	19.6	21.02	20.93
SH3PXD2A	18.49	17.96	18.6	19.59	18.83	19.6	20.27	21.14
C11orf49	21.42	22.33	21.94	19.75	20.1	19.61	17.45	19.46
CTPS1	17.54	17.48	18.33	18.46	20.3	19.62	19.96	18.64
UXS1	18.9	18.39	18.44	18.9	22.76	19.62	21.35	21.51
POLR2B	15.87	15.09	17.95	19.29	21.34	19.62	26.64	23.09
ACBD4	18.49	18.73	18.18	18.15	19.41	19.65	18.35	18.68
PPL	20.04	20.76	19.99	19.85	19.06	19.65	17.43	18.94
CROT	16.75	15.03	18.67	20.67	20.86	19.65	29.81	24.29
NUP210	21.59	21.18	21.14	21.03	20.35	19.66	19.63	20.53
GCDH	16.36	17.51	16.1	14.73	20.71	19.67	16.43	16.21
PDRG1	19.69	21.42	20.46	17.98	21.45	19.67	17.68	20.97
TBC1D5	17.56	17.42	19.6	21.25	20.66	19.67	25.44	22.42
ZFR	21.23	18.68	23	25.49	22.29	19.68	29.42	25.94
DHRS1	22.04	21.05	19.1	19.65	21.33	19.72	16.89	18.26
VAPB	18.66	17.91	19.56	19.25	20.37	19.72	21.49	20.88
LGR4	17.77	17.19	19.41	21.27	19.31	19.73	27.18	25.09
RABGGTA	17.64	18.57	17.36	15.74	21.3	19.74	16.43	17.18
SMIM19	20.97	20.95	22.33	19.32	19.44	19.74	19.12	17.83
TSN	17.74	16.59	18.94	18.52	20.24	19.74	24.15	22.8
CCNG1	17.43	17.55	18.09	18.6	19.45	19.74	27.05	24.18
ITGAV	18.18	17.32	20.43	24.51	20.18	19.74	33.68	29.98
COG1	19.41	20.92	18.88	19.21	18.25	19.75	17.77	17.62
SORD	17.1	16.84	15.94	16.96	20.15	19.75	18.14	19.26
IRF9	32.11	33.28	32.33	32.17	20.06	19.75	18.63	19.79
WIPF2	21.37	21.39	21.06	21.35	18.83	19.75	19.28	19.85
TMEM238	24.58	20.33	19.33	15.52	16.24	19.76	9.62	10.56
TAZ	19.59	18.58	18.83	20.86	19.47	19.76	18.49	20.3
LRIG1	23.01	21.58	21.63	22.31	18.51	19.76	19.29	20.3
MAX	17.32	20.05	19.67	18.55	20.04	19.76	20.48	21.36
BID	18.64	18.54	18.44	18.71	17.96	19.77	13.66	15.52
MED8	20.51	21.63	21.29	20.97	22.13	19.77	22.71	22.47
SRP72	17.73	16.56	18.92	21.23	21.8	19.77	26.67	22.49
PLXND1	20.02	21.05	19.26	19.49	19.27	19.78	15.89	18.14
ZBTB7A	20.52	20.73	20.66	19.93	19.94	19.78	16.92	18.18
PHF23	19.1	20.83	18.34	17.2	18.01	19.78	16.34	18.97
LOC102724159	16.75	16.31	17.01	15.01	18.38	19.79	16.37	16.98
SERTAD3	23.54	25.14	23.24	21.69	19.2	19.79	19.56	20.39
BCL2L12	21.98	19.52	20.22	18.93	21.37	19.8	17.51	17.16
FBN1	18.53	19.77	19.4	20.6	19.27	19.8	21.96	21.49
GLT8D1	18.67	18.64	21.72	21.43	21.16	19.8	26.43	25.2
CHAF1A	23.37	21.12	21.41	21.31	19.69	19.81	18.51	17.22
CCNH	16.17	17.08	15.95	18.75	19.02	19.81	25.28	21.02
EIF2B4	19.53	19.88	19.32	19.37	19.12	19.82	18.05	18.17
SRF	19.72	19.21	19.03	19.18	19.28	19.83	16.09	18.01
PREP	20.6	20.6	19.17	19.28	19.42	19.83	18.9	18.85
ACOT9	19.14	16.78	18.65	16.87	20.47	19.83	21.54	21.09
ERMP1	19.01	19.37	20.99	22.41	19.89	19.83	23.68	22.9
LLGL2	18.97	19.7	19.67	18.17	19.03	19.84	16.37	17.1
TMEM109	23.75	22.72	22.4	19.47	21.78	19.84	17.04	18.98
TOLLIP	17.54	18.17	17.77	17.34	17.85	19.85	15.48	15.49
KCTD1	22.39	20.57	20.46	20.16	18.14	19.85	17.74	19.37
MRPL15	17.23	16.49	19.44	17.57	18.88	19.85	20.15	19.91
CHRM3	22	21.22	21.69	23.25	19.99	19.85	23.22	21.65
BCAP29	18.45	18.74	19.68	21.58	19.66	19.85	30.52	24.34
NOL6	18.22	17.98	17.65	16.6	19.86	19.86	17.19	18.86
PRPF3	18.57	16.63	17.93	20.05	18.84	19.86	22.85	21.89
THRA	21.1	22.01	20.22	19.53	17.78	19.87	16.91	18.42
VGLL4	21.28	20.87	20.25	22.01	20.81	19.87	19.79	19.7
NUSAP1	17.43	16.16	18.89	19.63	20.75	19.87	29.52	24.1
CEP131	20.24	19.64	19.44	18.04	18.92	19.89	15.02	17.07
USP36	17.91	16.59	17.57	18.28	19.62	19.9	20.36	19.59
NUAK1	30.72	28.94	29.65	29.28	20.68	19.9	21.13	20.01
TCEA2	21.7	25.35	22.86	20.01	21.45	19.91	16.98	17.43
LIPG	14.81	16.84	15.15	17.36	17.67	19.91	18.89	19.93
TOX4	20.44	20.49	19.84	20.27	20.96	19.91	19.17	19.96
CCDC53	21.83	19.87	21.85	21.22	22.7	19.91	22.88	23.29
SPAG7	17.86	21.25	18.75	17.85	21.98	19.93	16.53	20.6
STAT2	26.08	26.73	25.32	26.41	19.63	19.93	22.03	21.68
OSBPL10	20.1	18.93	20.1	19.68	18.72	19.94	19.26	18.29
TCTEX1D2	24.16	21.04	21.7	23.14	19.16	19.94	21	18.65
METTL2B	17.69	17.19	16.28	18.02	19.25	19.95	21.38	19.09
MT1X	45.7	42.24	47.91	37.82	21.7	19.97	20.15	20.27
RAB24	18.21	17.88	17.98	17.47	20.92	19.97	18.37	21.31
ABTB1	19.36	20.42	18.9	17.73	19.7	19.98	15.8	18.2

CCDC50	18.09	16.61	17.99	19.88	19.64	20.02	21.1	20.19
RGP1	18.93	18.59	17.83	17.37	17.79	20.03	17.32	19.16
TRIP10	21.43	20.5	20.41	18.92	20.7	20.03	18.08	19.56
SPIN1	19	17.88	20.64	22.32	21.42	20.03	27.06	23.42
FAM102A	20.68	20.92	20.2	19.88	19.08	20.04	15.52	17.19
TIGD5	19.48	20.56	17.3	16.82	17.6	20.05	12.42	14.17
LMCD1	22.77	20.89	21.35	21.41	19.93	20.05	17.51	16.81
TCTN1	18.78	18.43	21.41	21.88	18.93	20.05	26.06	24.97
AAR2	21.82	20.33	20.92	20.43	22.2	20.07	18.46	20.17
CMAS	19.09	17.43	19.87	19.75	20.88	20.07	26.02	22.4
ENDOG	20.59	20.11	18.99	17.39	17.67	20.08	13.27	15.3
NCKIPSD	22.25	22.01	23.27	23.44	19.64	20.08	18.84	19.84
GOSR1	18.15	18.59	19.42	20.01	18.76	20.08	21.33	20.4
PTRH1	17.91	20.15	18.43	16.77	19.09	20.09	13.6	17.05
HAT1	18.91	20.46	19.6	20.05	19.11	20.1	25.67	21.39
RNF166	20.79	17.8	20.16	16.82	18.63	20.11	15.83	16.43
HPS1	21.2	20.16	20.1	20.47	20.35	20.12	17.08	17.66
POLR3H	19.21	18.89	19.57	18.84	21.59	20.12	18.54	18.73
ZDHHC7	17.47	19.66	18.57	19.26	21.19	20.14	20.89	19.8
PIP5K1A	21	20.99	20.91	22.48	21.86	20.14	22.6	22.28
SELK	18.85	21.83	20.44	21.07	22.38	20.14	23.45	22.62
TMEM261	20.1	18.55	19.14	17.62	19.51	20.15	17.75	18.7
ATG3	16.51	16.75	18.36	17.84	18.77	20.15	22.23	20.29
SHMT1	22.04	21.92	21.04	20.82	20.34	20.17	17.85	18.31
STK10	19.06	19.62	18.67	18.74	19.88	20.17	18.81	19.93
QDPR	19.14	20.78	20.29	20.08	17.6	20.19	16.7	17.18
GTF3C2	20.34	19.51	19.21	18.7	19.47	20.19	19.37	19.66
AIFM1	18.73	19.64	19.49	18.62	21.14	20.19	20.55	22.08
PTGS2	20.59	17.31	21.45	24.55	25.97	20.2	36.83	26.94
PDCD10	20.13	21.17	21.58	21.26	23.43	20.21	29.01	27.59
HSD17B1	20.87	19.26	21.12	21.76	19.97	20.25	16.57	20.55
LOC107987020	20.55	18.54	18.33	21.41	18.73	20.26	21.54	20.47
CDCA5	21.17	19.1	18.89	18.63	20.59	20.27	16.99	16.9
FAHD2A	17.19	17.83	18.75	15.42	17.95	20.27	20.48	19.53
VAC14	18.96	17.73	17.64	17.89	18.41	20.28	15.97	16.98
INTS10	17.06	16.94	18.15	18.6	20.96	20.28	23.14	21.19
COMMD5	18.66	18.43	17.65	17.51	19.98	20.29	16.54	17.23
VEZF1	19.29	18.48	20.23	22.85	20.48	20.29	25.43	24.92
SLC1A3	19.19	18.17	19.69	21.2	21.56	20.31	25.89	23.82
KIAA1671	19.18	18.81	19.94	21.02	20.23	20.32	22.81	22.94
SNRPD1	21.77	20.86	21.61	22.7	21.73	20.33	26.46	24
IQCK	24.47	24.72	24.87	25.08	20.72	20.33	23.28	24.08
CRTC1	20.35	21.33	19.5	17.45	17.55	20.34	15.07	16.2
METTL13	18.38	19.62	19.18	18.52	20.58	20.34	19.93	19.01
MRPS17	19.02	19.89	21.09	21.29	21.26	20.35	22.66	23.5
TMED7	20.14	19.11	19.74	22.01	19.19	20.36	30.2	25.34
TCF7L2	22.35	22.75	23.94	22.81	19.92	20.37	20.13	21.85
SLC30A5	19.88	18.87	21.34	22.57	21.11	20.37	27.17	23.1
EFCAB14	17.73	17.24	18.48	20.58	20.46	20.37	23.19	24.22
TUSC2	18.69	18.28	17.24	16.7	18.47	20.38	16	16.78
ING4	19.78	20.94	20.14	19.37	20.37	20.38	20.43	19.19
PSRC1	18.68	20.6	18.51	17.2	20.8	20.38	17.26	19.63
DIMT1	18.35	19.12	20.44	21.14	22.93	20.38	27.4	23.97
GBF1	21.59	22.32	21.8	20.97	19.13	20.4	18.1	19.93
OCEL1	21.38	24.83	21.54	19.72	20.48	20.41	16.38	17.63
TM2D2	19.99	20.98	20.6	21.49	21.06	20.42	23.46	20.66
TPRKB	15.99	18.95	20.53	20.81	19.92	20.42	28.85	24.15
FAAP100	21	21.11	19.63	19.93	19.67	20.43	16.7	18.12
RAI14	16.62	14.91	19.22	22.2	21.91	20.43	33.09	27.79
ANKLE2	18.64	19.25	20.66	19.41	20.73	20.44	21.92	19.75
MPRIP	18.52	18.51	18.93	19.14	19.82	20.44	21.34	20.67
ATXN7L3B	19.78	20.59	19.94	20.35	20.46	20.44	22.14	21.07
FAF2	19.16	19.02	20.69	20.55	22.39	20.44	24.26	22.76
MRPL16	19.27	16.35	17.8	17	19.02	20.45	18.95	19.4
PSMG4	21.28	20.58	18.86	19.56	21.98	20.46	17.12	17.73
DIABLO	22.86	21.02	21.88	20.78	21.33	20.46	21.81	20.63
ADK	18.68	17.94	20	22.1	21.6	20.47	27.66	24.8
OSER1	19.05	19.93	19.29	20.24	21.06	20.49	22.22	23.12
GABARAPL1	16.9	17.85	17.9	18.14	21.11	20.5	23.47	22.61
POLR3E	17.77	17.99	16.25	16.87	19.66	20.51	20.1	19.86
PPP1R13L	20.84	20.95	21.49	19.24	20.26	20.54	16.76	17.49
HS3ST1	18.87	18.66	17.94	17.44	18.02	20.54	17.42	19.23
GPAT4	18.52	18.86	19	19.41	20.22	20.54	22.49	21.11
PRR12	21.28	21.22	20.27	21.01	18.37	20.57	14.67	16.94
IMP4	17.99	17.65	17.94	16.7	22.22	20.57	18.62	18.68
CHD8	21.21	21.29	21.97	21.87	21.42	20.57	22.42	22.86
CYCS	17.41	18.1	17.82	18.97	21.83	20.57	27.16	24.95
CYR61	15.04	14.51	15.23	15.08	22.05	20.59	20.03	18.86
GLRX2	15.38	15.72	16.23	17.66	19.7	20.6	23.03	21.14
DHRS11	20.41	19.83	19.33	21.06	20.67	20.61	18.91	18.5
OSBP	19.17	19.78	20.58	19.99	20.36	20.61	21.96	21.53
PIP4K2B	20.49	20.27	20.92	19.92	21.94	20.62	20.98	22.37
CARHSP1	22.63	23.22	22.72	23.02	20.68	20.63	18.58	18.73
CCDC127	21.41	21.75	20.65	21.76	21.9	20.64	20.14	21.47
ZNF3	20.41	21.94	20.66	22.26	19.12	20.65	22.66	22.06
GPBP1L1	18.28	19.03	20.04	21.82	22.97	20.65	26.32	24.18

TNNT1	25.1	28.93	26.12	24.93	20.69	20.67	16.29	22.67
PRPS2	19.22	18.03	20.99	22.37	21.21	20.68	24.32	20.77
EIF5B	20.29	18.07	22.8	26.89	24.27	20.68	34.73	32.88
B9D1	21.79	21.51	23.05	20.82	18.97	20.7	16.77	16.05
TARBP2	20.44	22.34	21.65	18	20.32	20.72	15.76	17.53
ZMYND8	18.77	17.99	20	21.65	19.68	20.72	21.99	22
GPR107	20.2	21.17	21.5	22.05	21.62	20.73	23.21	22.02
PDCL3	18.54	16.8	20.23	18.83	21.11	20.73	25.55	24.52
DNASE1L1	18.97	17.97	16.81	17.57	21.31	20.74	17.83	18.66
ZWINT	20.12	18.66	21.53	21.44	20.12	20.74	21.36	18.89
SNCA	18.64	17.3	19.42	21.44	23.77	20.74	26.15	24.88
SCNM1	19.54	21.58	20.93	19.64	19.93	20.75	16.21	18.91
POLR2A	25.29	26.12	25.03	25.12	20.38	20.77	18.81	20.26
ZNFX1	24.85	24.51	24.29	24.84	20.83	20.77	21.68	20.28
WAC	19.14	18.16	21.31	24.51	20.85	20.77	29.46	27.32
FUCA2	17.66	18.16	17.43	19.02	20.43	20.78	19.52	17.97
NMRAL1	19.5	21.18	17.88	19	19.14	20.78	15.65	19.32
PREB	20.78	19.95	20.2	19.09	21.26	20.78	18.16	19.6
NOL11	18.08	18.09	20.07	21.6	21.38	20.79	28.66	25.12
NQO1	21.67	22.65	21.32	20.66	20.87	20.8	19.03	19.57
C9orf3	20.48	23.38	22.06	22.83	22.72	20.8	23.29	22.31
TSPAN6	20.55	20.51	19.36	20.88	21.59	20.8	23.88	24.59
RAP1A	22.05	19.32	22.79	24.79	22.23	20.8	31.31	25.55
NUDCD3	20.83	20.91	19.78	19.58	20.65	20.81	18.47	18.57
UQCC2	19.94	22.22	18.43	18.72	21.66	20.81	18.3	19.13
ALG8	20.37	19.54	21.97	21.31	21.25	20.81	24.24	21.81
RBM22	23.27	22.05	23.98	22.46	23	20.82	23.19	19.99
SRD5A1	17.93	18.38	18.06	17.81	21.78	20.82	23.37	20.63
RNF213	22.87	21.7	22.78	24.78	20.12	20.83	23.51	22.77
TLE1	23	20.9	22.26	21.61	20.49	20.84	18.97	18.85
PCDHB2	24.12	22.65	24.28	25.77	19.83	20.84	18.93	19.09
RAB2A	21.88	22.02	23.88	24.22	24.12	20.84	27.82	24.96
ZBTB48	22.23	21.9	21.8	20.54	19.32	20.85	17.33	19.33
ZMPSTE24	15.85	15.55	18.26	19.69	20.45	20.86	28.88	25.57
GPKOW	21.82	21.81	20.24	20.13	19.8	20.87	18.67	18.8
ELP6	18.98	19.06	18.71	17.48	21.79	20.87	18.04	19.46
MPND	22.92	23.35	22.09	19.45	19.91	20.88	17.82	17.74
GYG1	19.43	18.67	20.46	18.18	20.93	20.89	22.34	21.72
FSD1	19.66	19.64	19.02	17.88	20.49	20.9	16.2	17.39
TMEM55B	17.46	19.45	17.91	17.31	22.07	20.9	17.03	19.44
RPRD1A	18.09	17.1	20.62	21.56	21.14	20.9	26.55	25.65
NUP188	19.23	19.27	19.3	18.78	19.5	20.91	19.24	18.06
ZDHHC9	21.46	21.8	20.98	19.77	21.78	20.91	20.78	21.38
FOXP4	25.23	25.35	23.39	22.51	20.2	20.92	15.97	19.18
ELF4	21.71	23.58	21.72	23.34	21.07	20.92	19.12	20.85
NUP205	19.09	18.62	20.11	21.34	22.27	20.92	24.79	22.13
LZTR1	20.97	21.48	19.34	19.21	21.36	20.94	18.67	19.98
GTF2IRD1	20.56	22	22.41	21.9	19.16	20.94	18.99	20.67
PDCD11	18.55	18.84	18.64	18.36	20.86	20.94	20.43	20.88
PHKB	19.48	17.71	20.57	20.95	22.63	20.94	29.04	25.21
GNAI3	19.62	18.66	21.97	23.68	22.89	20.94	29.23	26.12
ZFYVE21	21.35	20.85	22.04	21.21	20.34	20.96	19.91	20.58
USF1	21.75	21.76	21.65	19.76	22.67	20.96	19.67	20.78
RCN1	18.34	17.37	19.48	20.23	21.86	20.96	27.17	25.67
EMC8	18.19	18.62	16.72	15.7	18.89	20.97	16.51	15.75
FAM43A	18.67	17.86	18	16.67	18.74	20.97	14.21	17.75
NIFK	17.52	16.19	18.57	20.15	21.53	20.97	27.34	23.89
USP7	20.05	19.02	20.99	21.46	21.27	20.97	24.38	24.89
FAM171A1	19.74	19.83	20.06	20.37	20.05	20.98	18.55	20.37
SLC25A29	19.58	19.08	18.41	19.25	19.48	20.99	20.95	20.57
FERMT1	19.73	18.25	19.17	19.47	22.35	21	23.37	22.35
CDK5RAP1	17.89	17.13	17.85	18.09	19.24	21.02	20.11	19.96
ATP9A	22.19	23.67	22.79	23.96	21.42	21.02	21.45	22.61
PLEKHF1	20.88	19.71	19.2	17.6	18.98	21.03	14.46	16.5
PLPP5	22.01	23.05	22.61	20.78	18.84	21.03	23.11	21.69
POP5	20.4	22.31	19.2	19.75	23.72	21.03	24.61	22.19
KLF6	17.39	15.98	17.46	18.55	21.71	21.03	23.65	22.94
DR1	20.08	19.09	21.12	21.76	19.41	21.03	23.07	23.23
TMEM127	21.14	22.17	20.49	20.39	21.1	21.04	19.5	20.61
TCF7	19.77	21.57	21.71	20.66	21.23	21.05	19.76	21.51
SFT2D1	17.39	20.64	20.05	21.04	20.52	21.06	23.81	21.82
TSR2	21.94	23.61	22.44	20.98	21.4	21.07	19.97	23.03
SMYD5	19.71	21.69	20.08	18.71	21.38	21.08	19.3	20.88
DDX18	19.83	16.84	23	25.51	22.29	21.08	30.98	27.29
KIAA0368	17.97	17.17	18.8	20.29	20.28	21.09	26.08	22.78
BBS5	20.89	19.77	21.76	23.26	21.94	21.09	21.39	23.04
AKIRIN2	19.84	19.33	20.19	20.78	20.96	21.1	18.7	18.85
SDHAF2	19.86	20.37	19.44	19.86	20.42	21.11	20.31	22.03
BRIX1	21.36	18.65	21.05	22.51	23.67	21.12	28.34	24.9
CIAO1	18.72	17.54	18.87	19.01	20.65	21.13	20.18	20.77
TUBB2A	23.11	21.66	22.78	19	19.89	21.14	16.84	18.88
CXorf40B	18.81	18.55	19.22	17.32	21.9	21.16	20.13	20.48
CHMP7	21.58	22.1	22.65	20.44	22.29	21.16	20.62	21.01
MRPL45	18.39	18.7	20.1	18.88	22.31	21.16	24.56	21.77
KDSR	17.38	15.96	18.8	20.34	21.46	21.17	25.63	22.38

TMEM97	19.75	20.18	20.2	20.2	21.75	21.17	21.7	22.41
SPEG	20.12	19.74	20.27	19.84	18.09	21.18	17.42	19.38
SMG7	20.88	20.57	21.26	21.72	21.57	21.18	21.87	22.21
AKAP2	15.54	14.99	16.96	18.18	22	21.19	25.6	24.16
LOC107985355	20.03	16.08	20	26.39	20.13	21.19	31.95	26.44
SPPL2B	18.3	17.91	19.14	18.2	18.39	21.2	18.68	18.33
ATG101	22.68	22.9	20.78	19.75	19.89	21.2	16.13	19.84
GLYR1	18.03	19.72	20.97	20.15	20.25	21.2	20.66	21.74
PACS2	21.14	22.03	20.45	19.7	21.96	21.22	20	19.75
MAPK9	16.88	16.04	19.05	20.98	19.45	21.22	24.91	23.64
WHSC1	21.87	20.64	21.61	21.4	21.22	21.23	21.88	21.07
SIN3B	20.47	19.5	19.33	20.31	22.01	21.24	19.83	20.41
ALDH1B1	19.08	19.24	19.73	17.62	22.38	21.24	19.6	21.18
PML	28.26	28.22	24.79	24.88	21.12	21.25	15.98	19.32
PRRC1	21.81	21.91	25.02	24.67	22.8	21.25	28.16	25.42
HAGHL	18.1	16.68	15.42	15.01	18.03	21.26	14.91	16.49
KRT6A	19.48	17.65	19.6	18.42	21.77	21.26	19.01	17.99
FHL3	22.59	27.33	22.86	22.05	21.64	21.26	17.25	19.25
MAP2K7	21.54	20.96	20.84	19.72	22.69	21.27	16.95	18.5
MTCL1	17.34	15.85	18.05	18.59	19.75	21.27	21.31	20.72
TRAPPC4	19.55	18.3	20.61	19.85	20.7	21.27	20.36	21.14
COMMD7	25.81	26.23	24.16	23.74	22.05	21.27	19.04	22.85
BYSL	18.04	19.72	17.87	17.26	19.9	21.28	17.47	16.38
SMYD2	20.65	21.08	18.72	18.44	20.21	21.29	22.6	20.7
SAC3D1	15.96	17.35	15.36	13.85	20.07	21.31	14.49	15.87
DEDD	21.9	21.3	21.35	20	20.35	21.31	19.4	20.35
ORMDL2	23.04	25.67	23.07	24.01	24.25	21.31	23.55	23.99
NLE1	19.58	21.59	19.53	17.61	21.43	21.32	16.78	17.66
CDKN2D	20.11	19.62	17.78	17.57	20.51	21.32	17.87	17.92
YRDC	19.73	20.35	19.43	19.48	23.98	21.32	21.61	21.87
UCHL5	20.78	20.8	24.43	24.59	24.41	21.32	34.09	30.03
TNFAIP1	22.54	22.98	20.73	20.74	22.1	21.33	19.45	21.45
TXNDC17	23.4	22.99	23.04	21.75	23.3	21.33	23.55	21.57
PPP1R16A	20.99	21	19.91	19.17	18.19	21.36	14.84	16.7
PPP1CB	20.06	20.47	22.16	25.48	21.06	21.36	32.81	30.53
TFPT	20.14	20.89	22.08	20.13	22.05	21.39	16.73	19.44
CDK1	20.65	20.06	19.65	21.42	20.93	21.39	29.52	25.51
ST6GALNAC4	19.58	22.24	18.12	18.2	21.47	21.4	15.88	17.11
PRMT7	21.68	20.58	20.14	20.21	21.34	21.4	18.45	18.66
RPA1	22.99	21.92	21.79	21.78	22.71	21.4	22.7	21.1
RBL2	19.47	18.03	21.14	23.83	22.76	21.4	33.09	28.65
URM1	22.45	19.92	19.1	19.63	20.98	21.41	18.97	18.63
AXIN1	21.59	21.02	20.36	20.54	19.89	21.42	17.24	18.68
TRIAP1	16.54	16.39	17.47	16.49	22.61	21.42	23.46	23.19
NGF	29.28	29.23	25.96	26.34	20.23	21.43	18.88	21.78
FJX1	18.9	19.19	18.83	16.44	19.99	21.46	14.96	16.43
FUBP3	19	17.76	20.85	21.92	21.8	21.46	24.87	22.73
PUM3	15.76	15.51	20.23	20.87	22.1	21.47	31.35	27.37
FAM60A	21.21	21.3	20.69	21.88	22.94	21.48	26.78	23.55
MFF	20.06	21.68	22.79	20.64	23.61	21.48	27.71	23.94
WNK1	19.74	19.62	21.93	23.22	21.58	21.49	25.29	24.66
DPM2	20.33	22.09	20.17	18.67	20.24	21.5	16.09	20.12
EAPP	21.21	22.34	22.03	23.95	23.97	21.54	25.97	24.72
ZMIZ1	22.38	22.51	22.97	23.74	19.76	21.56	19.55	21.21
TM9SF1	23.88	22.84	22.11	21.47	20.54	21.57	19.23	20.35
SEC14L2	23.71	25.4	22.77	21.74	21.96	21.57	19.04	20.88
WDR59	21.86	21.96	22.45	21.54	23.9	21.57	24.18	24.2
TSPAN14	22.51	22.34	23.6	21.05	21.58	21.58	20.91	21.39
C16orf45	20.44	20.4	21	19.78	21.98	21.58	20.68	21.75
PBX2	21.42	23.56	22.18	21.63	21.52	21.58	21.26	23.68
HES4	31.19	27.31	27.7	28.89	18.36	21.6	15.01	16.83
KATNB1	21.21	21.14	18.82	18.66	21.24	21.6	17.17	18.1
FANCG	22.06	23.09	21.92	18.63	21.68	21.61	18.09	18.76
C11orf24	21.22	21.54	21.95	21.28	20.79	21.61	18.9	20.28
CHMP6	23.95	26.18	22.44	20.36	21.74	21.61	18.9	20.92
PPP5C	21.22	22.12	20.53	18.78	21.11	21.62	18.25	19.45
LBR	20.82	18.97	23.01	25.61	22.33	21.62	31.22	29.89
ASCC2	21.62	21.81	22.57	21.94	23.28	21.63	19.04	19.86
NXN	22.23	21.39	21.91	18.96	21.37	21.63	17.86	21.01
CDC26	20.27	23.05	21.07	23.2	20.81	21.63	25.29	27.45
TRIP12	20.93	20.33	23.41	24.17	22.74	21.64	28.96	26.11
TMEM201	20.64	21.13	19.97	19.19	20.17	21.65	15.84	18.96
TBC1D16	22	22.51	21.32	19.1	19.11	21.65	16.62	19.04
CC2D1B	21.31	21.87	20.44	20.38	20.92	21.65	20.16	19.53
RPS6KA4	21.27	20.83	20.4	18.68	20.35	21.66	13.9	16.49
BFAR	21.08	21.1	22.62	22.52	21.81	21.66	23.77	21.89
PIR	18.91	20.62	18.9	19.65	21.8	21.68	20.66	23.14
NCS1	20.19	21.85	20.61	19.95	20.62	21.7	18.38	19.69
AFAP1	22.87	22.12	22.72	22.75	21.08	21.71	22.43	22.32
C14orf119	23.57	22.86	23.33	20.92	22.04	21.71	21.96	25.14
CFDP1	19.65	21.65	22.2	22.31	22.2	21.71	25.53	26.83
CPSF3	19.88	20.63	20.05	22.68	22.94	21.73	26.89	22.26
GPR108	21.23	21.49	18.97	18.45	21.63	21.74	18.63	21.05
JOSD1	21.35	21.58	21.54	21.93	22.17	21.76	21.41	21.89
MAP2K1	23.13	22.42	23.04	20.95	22.75	21.77	20.6	21.69
YEATS2	27.22	26.94	28.59	30.01	22.44	21.77	25.93	24.72

SUB1	22.06	24.46	23.06	22.09	21.87	21.77	29.2	25.48
TMCO3	21.35	20.76	22.73	23.63	23.15	21.78	22.05	22.24
AARSD1	21.77	21.04	21.69	20.65	22.84	21.79	20.28	20.82
UCK2	20.84	21.15	21.57	20.68	21.94	21.8	20.5	21.04
DHFR	22.25	17.98	23.02	22.32	23.94	21.8	25.37	21.62
PMAIP1	21.08	23.69	23.74	24.23	23.93	21.8	32.9	26.94
PIGO	23.48	22.41	21.16	20.49	20.81	21.82	18.93	21.71
ERLIN1	21.92	22.28	23.31	23.72	20.99	21.83	24.44	22.49
MGAT5	24.64	25.8	24.67	24.59	20.74	21.83	22.17	23.9
TFB1M	20.31	20.42	20.61	20.93	21.71	21.84	26.48	23.67
ERAP1	24.59	23.71	26.32	28.23	23.92	21.84	31.25	27.28
CERS5	23.41	24.68	23.46	22.5	22.54	21.85	24.68	23.2
DDX47	24.03	23.45	22.33	24.31	24.71	21.86	23.89	23.31
LLGL1	22.25	22.55	21.43	19.65	20.66	21.87	16.33	18.23
MTMR14	21.28	21.76	20.12	19.37	22	21.87	18.72	19.93
VPS16	21.67	22.08	21.41	19.22	21.85	21.88	19.07	19.24
EIF2B1	20.03	18.73	20.45	21.21	23.3	21.88	23.92	22.79
RAF1	22.35	22.86	21.61	21.5	23.58	21.88	24.56	23.15
SPOP	20.7	22.93	21.6	21.52	25.96	21.88	25.55	23.27
CEP170B	19.6	18.15	19	19.59	20.62	21.89	19.06	18.86
C19orf66	28.6	28.62	28	27.78	21.81	21.89	21.52	20.72
NCAPG2	20.94	18.05	22.73	24.34	24.18	21.89	28.75	24.71
DGAT1	20.12	22.45	21.47	19.46	21.31	21.9	18.49	19.96
SNRNP25	20.95	23.32	21.74	19.27	22.38	21.91	18.29	18.48
PIGG	22.75	20.8	20.11	22.08	22.31	21.92	21.92	22.76
XRCC3	23.98	21.91	21.78	20.39	21.67	21.95	17.57	19.71
DESI1	20.88	20.1	19.33	19.33	22.96	21.95	21.93	22.69
SPCS2	21.99	22.73	22.27	23.64	24.6	21.95	27.52	24.27
CAPN15	22.52	21.69	20.32	18.41	21.35	21.97	17.71	17.99
VAPA	20.96	21.39	22.81	22.49	23.8	21.97	27.86	25.72
POLR2F	18.16	18.59	17.52	19.44	20.59	21.99	17.76	18.22
TNPO3	22.26	21.34	22.35	23.59	23.21	22.01	24.55	23.52
UNC119	24.13	23.3	24.91	23.56	23.91	22.02	17.64	19.2
SMURF1	21.89	21.43	21.53	22.07	20.89	22.02	21.1	21.48
SEMA3B	22.61	22.47	20.95	20.8	20.82	22.03	18.69	18.4
FAM160B2	22.71	21.78	20.33	20.88	20.41	22.03	19.97	21.04
HBP1	18.49	19.41	22.61	22.34	21.24	22.03	28.13	24.83
PIGQ	20.58	19.85	18.9	17.58	19.47	22.04	16.6	17.55
NOC4L	22.72	23.03	20.44	19.55	23.85	22.04	16.14	17.98
ITFG1	21.04	19.35	23.31	25.45	21.48	22.04	30.03	29.08
MEGF8	25.09	24.98	24.33	23.95	20.04	22.05	17.87	19.18
GOSR2	23.76	21.35	23.43	23.61	23.54	22.06	23.4	23.63
DYNC1LI2	20.4	18.73	21.75	23.54	21.55	22.07	30.02	24.17
DNAJB2	24.33	23.19	24.33	21.59	20.99	22.08	18.72	21.3
KDM5B	26.01	25.69	26.56	26.84	22.26	22.08	25.35	26.18
NPAS2	21.73	22.34	23.12	22.84	21.56	22.09	22.87	23.42
VBP1	24.6	23.83	23.02	24.49	24.95	22.11	30.59	27.31
SIRT7	24.89	23.68	22.3	22.54	23.16	22.13	18.88	20.41
OAZ2	22.54	22.76	22.62	21.43	22.5	22.13	19.19	22.57
MRPS22	17.29	20.17	19.63	21	22.53	22.13	25.4	23.23
SENP3	22.6	23.39	21.15	20.7	23.04	22.16	19.78	20.24
ABHD8	24.77	24.64	22.93	21.63	20.58	22.17	15.87	18.49
VEGFA	29.11	27.74	27.25	28.94	23.05	22.17	28.39	26.39
PLEKHB1	22.22	25.23	22.2	20.77	22.46	22.19	19.99	22.37
GPR137	21.64	22.65	21.2	19.5	20.39	22.2	16.2	19.44
ARHGEF10	20	18.5	20.61	20.47	21.31	22.2	23.74	23.15
NOL3	21.28	19.78	19.17	19.45	18.62	22.21	21.34	20.33
MGAT2	22.75	22.06	22.52	22.81	20.99	22.21	21.8	21.91
POFUT2	22.56	22.57	20.76	22.16	21.03	22.22	19.96	20.93
MLEC	22.66	24.38	22.92	23.46	22.18	22.22	22.79	23.19
PYM1	23.49	23.8	22.15	20.48	23.55	22.23	19.24	19.4
AIM1	20.12	18.92	21.71	23.79	22.91	22.23	27.26	24.02
SLC43A2	18.59	19.56	17.89	16.58	21.05	22.24	16.65	18.55
TAF12	20.33	20.3	22.97	24.12	25.34	22.24	25.37	26.46
TLCD1	22.05	23.1	20.2	19.11	23.57	22.29	19.87	20.53
WIPI2	21.58	22.65	21.12	20.44	23.24	22.29	20.58	20.76
PARP4	23.49	21.49	24.73	26.09	23.28	22.3	28.6	25.12
PSMC6	21.43	22.02	20.55	23.53	23.74	22.3	28.48	26.27
SMPD1	21.11	23.92	21.18	19.86	18.73	22.31	17.66	19.25
MMAB	18.86	20.78	16.37	16.69	24.57	22.33	18.76	20.76
RNF20	21.61	20.81	23.17	23.64	23.28	22.34	25.37	24.58
DUSP7	20.23	20.06	20.41	20.76	22.17	22.35	19.93	22.39
PDXP	22.5	20.99	20.78	17.7	20.04	22.36	17.04	16.51
NRBP2	20.38	19.58	20.47	20.82	19.03	22.36	24.5	23.68
SLBP	22.38	22.02	23.72	25.46	23.79	22.36	24.62	24.25
PEG10	26.93	25.89	28.87	34.82	21.19	22.37	27.41	25.8
AGPAT3	23.12	23.57	22.43	22.96	20.94	22.38	19.46	21.76
SCCPDH	21.81	21.98	22.9	23.76	22.88	22.38	26.99	23.99
ZBTB4	21.97	22.83	21.64	21.69	21.69	22.39	19.21	21.05
TIMM13	23.68	21.42	21.04	20.71	22.15	22.41	16.42	18.38
PRKCDBP	25.89	27.21	25.29	22.36	21.61	22.43	15.55	17.29
THOC5	19.02	19.77	18.6	18.2	23.09	22.43	20.55	21.24
PSME4	19.8	18.27	21.77	23.51	23.29	22.44	30.42	27.21
NGFR	23.32	24.51	20.68	21.25	23.56	22.46	18.91	23.45
ERLEC1	20.64	20.81	23.01	24.83	24.69	22.46	32.62	28.51
ZNF687	25.86	26.19	24.62	23.12	23.21	22.47	18.56	20.84
HSDL2	18.41	15.85	20.78	21.52	23.3	22.47	30.46	25.56

UBE2E1	21.43	22.86	23.28	23.7	25.43	22.47	29.83	25.84
PREX1	20.34	19.72	19.41	17.71	21.94	22.49	19.14	19.62
PI4KA	20.85	20	20.39	20.02	21.52	22.49	23.25	22.72
MYO19	21.3	18.82	20.6	22.45	21.62	22.49	25.14	23.28
SSR3	20.74	20.2	21.1	21.24	24.27	22.49	26.27	24.32
PPP3R1	21.27	20.69	23.46	23.92	24.53	22.49	25.71	24.34
CDC27	21.33	19.32	21.82	26.05	23.46	22.49	32.41	27.27
ELP2	20.89	20.7	21.14	21.01	22.68	22.5	24.14	22.62
MTG2	22.19	22.63	21.9	22.3	21.37	22.52	21.55	20.9
ECI2	23.18	23.32	23.31	21.77	23.88	22.55	24.61	26
HELZ2	44.88	44.04	42.86	42.63	20.18	22.56	18.5	19.33
PHF5A	21.14	20.13	22.02	21.44	21.97	22.56	20.18	20.29
RAI1	25.65	23.61	25.07	24.23	21.97	22.56	19.81	20.42
SIRPA	21	23.94	21.47	22.19	23.28	22.56	20.36	22.84
TRIM14	29.4	27.58	27.37	29.47	20.15	22.58	20.88	19.06
C8orf33	18.23	20.2	18.66	17.67	22.6	22.58	21.89	20.92
GLTP	21.78	22.23	21.66	22.37	23.48	22.58	23.19	24.07
RPA2	22.49	23.52	21.56	22.95	26.17	22.59	26.67	23.15
ARGLU1	21.52	20.93	23.12	28.49	23.08	22.59	34.04	32
UBAC2	19.94	20.68	20.31	19.94	22.03	22.6	20.73	20.75
DPH2	20.44	18.37	18.81	17.86	22.03	22.61	19.9	20.23
MPV17L2	19.47	20.83	19.61	19.37	21.59	22.61	19.55	20.43
CRKL	25.92	25.69	25.72	25.34	22.98	22.63	22.19	21.72
WDTC1	21.73	23.29	22.95	21.1	21.75	22.64	20.54	22.23
YIPF4	19.15	19.2	20.1	20.74	21.16	22.65	28.83	25.69
HNRNPDL	20.57	20.63	22.16	25.4	24.02	22.65	34.34	29.69
NELFA	22.12	23.68	22.06	21.48	21.15	22.67	16.71	18.93
TRUB2	21.31	20.85	20.14	18.37	23.53	22.67	21.29	22.27
ADSS	20.1	18.73	21.14	24.42	24.46	22.67	33.01	27.59
FBXO7	21.43	23.15	22.3	21.78	22.99	22.7	21.9	22.76
ETHE1	25.57	25.13	22.25	22.17	25.73	22.71	18.46	21.58
OSMR	22.33	22.28	24.83	25.74	22.2	22.73	28.43	25.94
HMGN4	21.07	19.83	20.42	21.92	24.44	22.73	30.73	26.36
EPS15L1	24.11	23.22	21.45	21.26	21.6	22.74	20.26	20.98
ARFIP2	23.09	22.58	22.12	20.96	23.32	22.74	21.09	21.74
GGA1	21.41	21.85	21.52	19.62	21.38	22.75	21.49	20.47
STARD3NL	19.03	22.18	19.4	23.1	22.1	22.75	26.2	21
TSSC4	22.76	20.68	19	19.53	20.54	22.76	15.79	17.84
CYTH2	21.16	20.58	20.42	19.74	24	22.77	21.74	20.83
OAS3	51.16	50.88	50.52	50.76	22.94	22.77	24.06	20.92
TCEAL9	25.79	26.46	25.94	27.66	24.23	22.77	33.89	31
TRIB3	21.63	23.88	21.54	19.94	21.17	22.78	17.08	19.03
HDAC10	19.12	19.76	18.7	18.32	20.94	22.79	20.38	20.46
BRE	22.42	24.05	23.9	24.89	23.49	22.79	23.18	22.68
ISCA2	24.72	25.93	23.15	22.07	23.58	22.79	21.04	24.22
HSD17B3	22.28	24.28	22.68	21.92	19.18	22.8	19.76	20.52
MRPS28	20.27	19.73	20.44	18.59	22.55	22.81	26.4	21.24
MIB2	21.5	21.54	21.06	19.22	22.38	22.82	18.32	19.07
MRPL17	24.34	24.29	22.33	23.51	24.14	22.82	21.37	21.59
TMEM9B	20.77	21.42	22.77	22.14	24.24	22.82	28.79	23.98
GGH	22.61	19.94	24.87	25.84	24.74	22.82	35.36	25.61
CFAP36	23.86	22.01	24.06	27.63	24.72	22.82	30.79	26.3
AGPAT2	25.59	27.1	24.47	22.26	21.16	22.84	17.14	17.56
TOR1A	22.98	21.58	21.25	20.7	22.07	22.84	23.56	20.04
RASSF7	25.25	26.39	22.03	21.63	19.78	22.86	18.34	19.06
GBA2	23.11	23.3	22.39	22.72	23.81	22.86	23.89	22.17
CNOT11	23	23.5	22.47	22.29	23.31	22.86	22.78	23.86
ADCY3	20.41	21.06	19.67	20.33	21.89	22.89	19.57	20.28
PPP2R5C	21.68	21.29	22.65	23.08	24.86	22.91	33.97	29.72
USP14	20.66	19.39	22.58	21.81	23.44	22.92	29.52	26.65
DCAF6	21.73	20.5	22	25.74	24.11	22.96	30.55	26.9
DALRD3	24.08	24.45	22.79	21.23	20.76	22.97	19.07	19.55
SHB	21.98	23.51	22.34	20.81	21.22	22.97	18.94	19.73
PLSCR3	22.76	24.07	23.09	21	23.4	22.98	17.17	20.11
IMPAD1	19.31	17.77	21.81	23.52	22.6	23	29.63	26.24
CENPV	21.55	20.37	21.99	20.41	20.11	23.01	17.49	18.72
PSKH1	23.8	25.66	24.3	22.18	22.56	23.01	17.61	20.22
PMM2	23.09	20.73	21.33	23.13	23.3	23.02	22.15	23.54
44257	24.96	22.89	23.14	20.43	23.06	23.03	17.79	21.53
SH2B1	22.27	22.54	21.87	22.52	23.36	23.03	21.85	23.4
PAF1	25	25.74	25.07	21.38	24.48	23.04	20.22	21.98
ZNF395	29.99	29.06	29.54	29.18	23.2	23.04	21.92	22.07
VPS37B	21.88	21.92	22.14	21.19	22.56	23.05	19.06	20.02
RECQL4	22.36	19.58	21.1	18.98	22.18	23.06	18.55	17.76
UGP2	21.76	20.83	22.46	25.74	23.47	23.06	32.7	28.78
HEBP1	21.02	21.89	23.15	21.5	22.92	23.08	19.91	20.52
SKI	24.79	25.79	23.99	24.37	21.32	23.08	19.01	21.51
FAF1	22.97	21.74	22.63	23.4	24.31	23.08	23.56	23.74
DPH5	23.9	22.12	23.09	22.51	26.49	23.08	29.33	26.39
TNS3	21.52	20.67	20.91	21.19	23.47	23.11	22.46	21.94
AP3B1	21.63	18.5	22.56	24.19	25.23	23.11	30.81	26.81
SMIM12	21.42	22.89	21.41	19.21	23.62	23.13	18.26	20.68
SDC4	26.27	26.96	26.5	27.58	26.86	23.13	25.6	24.49
DOK1	25.5	25.68	23.54	22.91	20.45	23.15	16.53	18.77
BAIAP2	23.54	23.89	22.19	21.89	22.91	23.16	19.43	20.15
SMTN	20.23	19.76	18.62	18.89	22.96	23.17	17.86	21.84
SEL1L3	20.3	20.11	20.57	22.01	23.3	23.17	26.68	24.59
LETMD1	20.41	18.38	22.55	23.05	25.24	23.17	27.48	27.39

CCS	24.78	26.12	23.23	22.64	23.19	23.18	19.64	20.77
PHF1	22.81	23.57	22.65	22.4	23.78	23.18	22.35	21.44
MAGOH	23.39	24.92	23.62	27.27	26.13	23.18	27.75	30.99
BTBD6	21.96	21.43	22.76	20.94	20.62	23.21	17.89	18.05
MAFG	21.44	21.15	21.39	21.53	24.71	23.21	21.61	21.88
UBFD1	28.98	27.46	24.34	26.33	24.3	23.21	23.97	22.24
MTHFD1L	24.17	23.01	21.65	23.67	26.11	23.21	22.85	23.37
ATG13	23.76	24.27	22.76	22.19	23.57	23.25	21.91	22.6
ACO1	19.46	20.22	19.41	19.37	24.03	23.27	23.72	24.08
ORAI1	21.8	20.5	19.91	19.33	19.47	23.3	16.16	17.76
BCAT2	26.32	26.11	23.16	21.42	24.04	23.3	19.39	20.32
TP73	25.79	25.58	25.2	23	22.16	23.3	19.74	21.14
TIMM10	22.64	22.07	21.33	20.73	25.72	23.31	21.97	20.76
GMPR2	22.36	23.99	21.55	23.6	22.7	23.31	22.74	24.36
SLC37A3	23.75	22.88	24.17	28.03	23.01	23.32	29.04	26.88
TMEM59L	39.21	42.44	38.22	35.03	20.14	23.34	15.84	18.85
ERAP2	23.18	21.13	25.93	31.76	24.82	23.36	39.02	32.7
RALGDS	24.23	23.56	25.39	24.53	22.99	23.39	20.93	22.53
VPS29	20.3	20.11	22.92	22.91	25.58	23.39	28.76	26.1
NMD3	21.74	22.31	23.45	25.87	24.48	23.4	35.11	29.31
STK39	23.17	19.91	25.5	25.59	23.58	23.43	27.52	25.32
GLDC	19.88	20.56	20.27	20.33	24.12	23.45	25.38	23.31
CAD	22.9	22	21.68	20.12	22.25	23.47	20.12	21.19
BTG1	23.27	25.4	23.72	24.02	22.07	23.49	22.02	22.38
GTPBP4	21.22	20.65	22.34	22.8	24.39	23.51	28.25	28.09
EXT1	28.15	28.08	27.5	27.11	25.22	23.52	22.64	22.9
MEMO1	20.25	19.61	22.14	19.73	25.12	23.53	27.57	23.95
GINM1	22.9	20.14	22.63	27.18	24.69	23.53	32.63	28.03
CDK10	21.97	22.39	20.43	19.45	25.86	23.55	22.12	23.81
FOXG1	19.97	18.6	21.62	20.01	20.24	23.56	21.06	21.01
LRRC42	24.7	26.27	24.77	24.33	24.83	23.56	25.05	23.17
NCKAP1	22	21.14	23.43	27.04	23.17	23.56	36.52	30.87
C9orf142	22.83	23.39	22.39	19.13	24.32	23.58	20.18	21.03
PGAP2	24.12	22.11	22.72	21.97	23.85	23.59	22.22	21.87
TRIM65	22.48	22.53	23.3	22.04	22.55	23.6	20.2	21.67
CBX4	28.29	27.54	26.56	26.72	23.12	23.61	17.5	18.75
RPUSD1	22.06	21.15	21.87	19.38	25.14	23.61	20.37	20.68
MMS19	23.94	23.35	24.21	23.04	22.9	23.62	23.59	23.55
SGSH	22.72	23.72	22.19	21.48	21.13	23.63	17.65	19.59
C9orf78	19.95	21.08	21.73	23.04	25.43	23.63	25.89	26.79
44444	25.01	26.52	25.67	23.43	22.55	23.64	19.81	22.19
NFU1	20.98	20.97	22.81	22.03	23.18	23.64	27.34	25.81
KIF20A	17.2	17.17	16.59	15.08	22.62	23.65	23.47	23.44
PPCS	24.14	23.12	25.1	21.58	23.94	23.66	19.53	22.44
DDX23	24.67	26.47	25.21	24.76	24.7	23.66	23.61	23.35
TBL3	24.22	23.91	22.27	20.45	22.02	23.67	17.82	19.6
VSTM2L	19.84	21.3	18.44	17.19	25.21	23.7	18.25	20.15
EDC4	26.06	24.15	24.25	22.72	23.58	23.7	20.08	20.53
PCGF2	24.17	24.49	25.33	24	23.63	23.7	20.35	21.61
PEX14	25.21	25.26	26.05	21.52	23.31	23.7	18.89	21.81
LPIN1	14.49	16.4	15.91	20.12	21.62	23.7	28.11	27.47
NUDT21	21.83	20.03	24.99	25.75	26.2	23.7	34.12	28.49
SH3BGRL	22.8	25.14	23.72	28.1	26.85	23.7	34.93	28.54
PIGY	22.37	22.39	23.59	24.31	24.29	23.71	27.02	23.58
NAE1	22.39	20.84	23.63	25.92	24.73	23.71	38.66	30.59
LONP2	22.42	21.93	22.74	22.01	21.42	23.72	24.65	23.54
ZFAND5	22.11	21.36	24.07	25.44	23.57	23.72	28.52	26.35
MSRB2	19.31	19.11	19.26	19.73	20.04	23.74	16.3	16.13
MIIP	22.26	19.64	19.87	20.46	21.31	23.74	18.85	20.84
MAGEF1	23.43	22.47	21.95	23.08	23.08	23.74	22.76	21.9
KIF1C	20.75	20.86	20.31	18.81	23.69	23.74	19.14	22.21
PAGR1	23.63	22.7	22.66	21.47	24.09	23.75	21.36	20.94
FAM173A	23.41	22.53	21.33	18.81	22.98	23.76	14.67	18.49
TIMM44	22.25	21.3	21.14	21.42	23.49	23.78	19.39	20.95
WDR5	23.26	22.99	21.7	22.74	24.97	23.78	22.94	22.83
CMTM8	28.57	27.56	26.96	26.65	23.6	23.79	21.11	21.4
UTP6	21.93	22.62	23.29	23.73	27.82	23.79	30.64	26.78
TOR3A	23.67	23.12	21.7	21.1	23.16	23.81	21.03	22.12
FHL1	26.32	28.52	26.98	29.4	23.8	23.81	26.15	25.75
PPIF	24.91	24.02	23.52	22.98	24.62	23.82	22.53	23.76
COX14	23.93	25.35	23.34	19.93	24.97	23.83	20.18	22.91
TCEAL8	24.73	24.58	26.72	29.2	24.87	23.83	31.69	28.43
GIGYF1	21.78	20.4	21.67	23.16	23.46	23.85	24.75	25.2
MOB1A	21.57	21.3	25.21	30.17	25.37	23.85	40.14	36.11
SDR39U1	20.47	21.33	17.69	20.39	24.63	23.87	21.19	23.6
TCTN3	23.66	22.58	22	24.48	25.24	23.87	26.28	24.26
PSMG1	20.89	22.09	20.99	19.95	26.36	23.87	28.73	25.32
ABRACL	24.76	23.38	23.45	26.01	25.55	23.89	25.51	25.54
TMEM214	23.91	24.97	23.45	24.57	23.8	23.9	21.43	21.49
ASNS	27.2	27.48	27.23	26.82	26.04	23.9	26.6	24.47
TCAF1	25.46	22.52	27.58	30.58	26.27	23.91	32.8	28.76
IVD	25.48	25.81	25.07	23.43	24.24	23.92	23.26	24.63
THYN1	23.83	23.11	22.5	21.12	27.15	23.92	26	27.82
GTPBP3	22.86	21.19	21.89	21.78	24.16	23.93	21.39	21.64
PI4KB	24.53	26.26	25.26	24.35	23.18	23.93	22.38	22.8
INPPL1	22.87	23.28	23.17	22.77	23.77	23.93	23.53	25.07
NAT10	24.51	23.46	22.48	22.51	23.59	23.94	23.41	23.51
SARNP	26.03	23.19	24.58	24.02	27.36	23.94	27.18	23.86
NIPAL3	19.43	19.56	20.06	20.33	23.29	23.94	24.69	24.13

ZNF532	23.96	22.77	24.91	28.98	24.72	23.95	30.59	28.54
HYPK	22.99	23.24	22.86	24.19	27.57	23.96	21.58	22.63
THAP7	23.29	23.14	21.91	23.18	21.86	23.99	16.78	18.3
SCO2	22.12	20.65	23.13	18.98	21.4	24	14.78	16.11
TBC1D14	21.93	21.4	21.01	22.14	24.87	24	23.46	25.22
GNPAT	21.76	20.17	22.75	23.66	25.17	24	27.56	26.53
PRPF40A	22.88	22.31	23.47	27.12	24.26	24.01	37.7	33.93
AGBL5	22.97	22.42	23.74	22.13	23.67	24.02	20.08	22.09
NRM	29.07	28.9	30.48	28.18	24.47	24.02	21.63	22.15
PLAU	21.95	24.4	23.17	22.38	23.34	24.03	22.51	23.65
KIAA0101	23.14	22.8	25.37	23.12	26.61	24.03	30.32	25.27
SAMM50	20.81	22.69	21.28	20.16	23.63	24.04	23.1	24.77
C12orf75	19.95	20.15	21.48	21.06	25.93	24.05	27.59	26.76
METAP2	20.54	18.71	23.84	27.68	25.72	24.06	38.26	33.38
RRP1	21.47	23.84	22.06	21.41	26.46	24.07	20.5	21.91
SPATC1L	25.43	25.95	23.35	23.33	24.39	24.07	19.34	23.04
MPI	25.41	26.2	25.69	22.63	22.82	24.09	20.7	21.49
PRC1	20	17.87	20.93	21.17	25.69	24.09	25.83	23.66
GATAD2A	23.72	24.83	22.62	22.88	23.73	24.1	21.07	21.73
SPTLC1	23.43	23.7	24.7	27.52	28.8	24.1	33.92	29.61
TRIM56	24.24	22.4	24.95	27.26	22.86	24.11	25.93	25.59
DHX8	23.2	24.71	24.3	23.47	25.41	24.12	24.56	23.52
CLK3	23.24	23.13	22.46	22.94	23.43	24.15	22.88	23.94
MUL1	22.03	22.15	20.22	20.53	22.48	24.16	22.16	22.17
ASXL1	25.94	23.87	27.35	25.03	26.16	24.16	25.13	26.47
ERV3-1	23.51	24.4	26.11	28.85	24.87	24.16	34.94	30.3
C21orf59	23.5	22.87	23.98	24.32	22.79	24.17	23.68	22.97
PRKCD	25.99	26	24.62	21.69	24.49	24.19	21.32	22.71
SLC38A1	18.95	17.83	21.71	24.16	26.47	24.19	39.97	33.02
SCAMP4	23.96	25.46	23.17	22.64	21.82	24.21	18.79	21.11
PLEKHG5	22.31	22.69	21.76	23.39	22.9	24.22	23.41	24.49
ACAD9	25.49	23.7	23.84	21.84	22.39	24.24	21.35	23.6
FAM213B	24.63	23.81	23.66	21.66	22.62	24.25	17.87	20.43
HAUS4	23.64	22.65	24.2	20.01	24.45	24.28	21.1	22.94
IKBKB	24.68	23.38	25.43	24.03	23.37	24.28	25.59	23.3
JAK1	27.07	24.99	26.94	28.66	25.22	24.28	28.8	27.39
PRMT6	22.18	22.88	23.52	23.56	23.46	24.29	21.69	22.42
NCDN	21.93	21.84	20.63	21.04	21.93	24.31	19.51	21.29
NEU1	23.73	25.73	23.76	21.37	24.77	24.31	20.85	23.18
SYMPK	24.76	24.09	23.79	22.72	23.71	24.32	19.94	22.13
USP19	24.68	24.61	23.54	23.23	22.77	24.32	21.24	22.14
CYP2S1	24.38	26.94	23.62	22.45	23.3	24.32	20.18	24.23
QRICH1	24.33	24.96	25.86	25.21	24.54	24.33	25.22	24.57
RBM5	24.58	22.22	24.46	27.66	24.19	24.34	28.97	27.82
G3BP2	25.56	22.07	26.17	27.34	24.79	24.35	31.9	29.53
MRPL47	20.11	20.66	24.63	24.21	23.9	24.36	30.32	26.96
PAK2	22.28	21.35	24.07	25.74	26.31	24.36	32.02	29.09
BMP1	25.87	24.17	23.9	23.94	23.09	24.37	21.74	22.46
WDR3	21.3	17.98	22.08	23.04	26.94	24.38	31.68	27.85
BET1L	23.67	23.81	24.19	24.08	23.9	24.39	21.93	22.89
BTBD1	20.83	19.96	20.93	23.17	24.58	24.4	31.46	27.44
CPD	24.54	21.89	27.48	29.55	24.74	24.41	34.31	31.02
SUGP1	24.56	26.86	25.29	23.79	26.26	24.42	25.12	24.41
PXMP2	23.25	26.24	22.46	22.47	21.22	24.43	19.3	19.94
TSEN54	23.07	24.83	24.04	23.03	24.48	24.43	21	23.26
SPSB2	19.1	22.05	19	18.91	22.3	24.44	20.01	19.28
FBXL16	22.86	21.53	22.28	21.15	22.43	24.44	17.68	20.05
ITGA5	27.71	28.67	26.09	24.18	24.37	24.47	21.76	24.21
SLC9A1	24.72	25.95	24.55	25.38	23.79	24.47	21.94	24.26
PLXNB3	27.25	25.74	26.13	27.29	21.45	24.48	23.83	24.46
DHRS3	28.48	29.55	27.53	24.22	24.24	24.49	18.83	20.67
PMVK	28.44	27.17	25.67	23.49	27.52	24.49	21.98	21.16
PEX19	25.21	24.7	24.49	24.38	26.39	24.49	26	25.09
COL27A1	15.74	14.83	17.1	19.47	22.88	24.49	31.52	31.12
HDAC6	22.19	21.98	21.84	21.24	22.8	24.5	21.93	21.06
CCND3	27.47	27.95	26.8	24.45	24.56	24.51	18.84	20.49
FBXO18	23.64	23.59	22.8	22.98	22.88	24.51	22.28	23.41
MAPKAPK3	25.1	23.08	22.12	22.14	24.98	24.52	20.51	21.77
HDAC5	27.45	26.38	26.66	24.07	24.47	24.53	19.39	21.63
MRPL13	24.59	25.87	23.51	24.34	26.11	24.53	34.23	27.87
SNX6	22.33	22.73	22.53	24.41	24.95	24.53	32.76	29.03
PPOX	22.5	21.2	22.03	20.71	23.44	24.54	20.65	21.22
ADPGK	25.72	23.97	24.35	26.1	26.81	24.54	26.73	26.28
SPRED2	25.57	25.36	27.01	28.15	26.12	24.55	25.91	26.04
PLK2	21.21	18.83	22.25	22.75	26.41	24.56	29.11	24.68
EIF1B	23.99	23.38	23.33	23.64	26.07	24.56	25.97	25.02
DARS2	19.52	20.03	20.26	21.09	25.62	24.56	28.51	26.64
SNRPD3	23.72	23.46	23.75	25.24	24.08	24.57	27.34	27.35
UBTD1	24.28	23.79	23.3	20.96	21.41	24.58	17.17	20.19
VPS52	24.11	25.5	24.92	23.21	23.22	24.58	24.62	24.34
C11orf73	22.3	25.1	22.93	21.81	26.43	24.58	27.47	26.19
RBBP4	24.58	24.03	26.52	27.34	25.54	24.58	30.54	27.38
MTFP1	28.85	27.4	27.24	24.3	25.91	24.59	20.19	21.26
PSMG2	23.22	22.79	22.2	24.36	26.98	24.59	30.71	28.14
ABCA7	22.73	23.07	21.77	22.44	21.33	24.61	19.74	22.78
TPST1	24.72	26.16	24.8	26.19	25.24	24.62	25.36	25.32
ARCN1	24.77	23.55	27.62	26	25.58	24.62	29.2	26.95
HMGN3	24.86	27.36	21.43	25.22	22.58	24.63	28.15	27.13

TNPO1	20.7	18.92	22.91	25.87	24.87	24.63	37.33	32
ARMC6	22.55	20.28	21.39	18.97	24.28	24.64	18.4	21.14
FBXL19	22.67	22.75	22.16	21.06	23.45	24.64	18.15	22.83
KCTD17	25.42	26.41	25.29	23.65	23.07	24.65	16.44	18.48
YBEY	26.11	26.6	24.57	25.04	28.83	24.65	27.23	24.1
TCEAL4	25.81	24.67	25.92	30.89	25.96	24.66	30.27	30.59
GPN2	23.03	22.5	24.23	22.65	25.36	24.67	19.11	19.76
VAMP5	25.95	27.44	27.37	25.64	22.32	24.67	22.4	21.52
SDF2	24.69	26.46	26.67	22.94	23.92	24.67	23.59	22.33
DDAH2	27.79	30.01	27.53	25.7	23.27	24.68	18.34	22.2
C22orf23	20.69	20.6	21.89	20.04	26.35	24.68	22.96	24.54
DEDD2	26.24	26.38	26.29	23.3	22.98	24.72	18.87	20.56
PSMF1	24.43	24.06	23.6	23.44	24.33	24.73	20.38	22.63
GCSH	23.75	21.73	21.66	22.11	26.2	24.73	30.74	26.2
HPCAL1	25.23	26.31	23.74	23.14	23.19	24.74	19.44	21.9
NFKB2	24.75	25.33	24.5	22.4	25.1	24.74	21.99	22.96
NFS1	21.19	21.45	19.98	21.1	24.72	24.74	23.89	23.69
PRICKLE3	25.76	25.82	25.44	23.21	23.93	24.77	19.71	21.11
HS1BP3	24.08	25.78	24.44	22.3	25.41	24.79	19.16	20.27
PPM1F	26.16	25.85	23.99	24.08	24.89	24.8	20.86	22.64
KIFC2	18.94	20.1	18.17	19.78	22.67	24.8	25.91	23.9
MGEA5	22.34	21.44	25.16	27.59	26.32	24.8	38.58	31.59
DCTD	24.53	24.65	23.81	21.97	24.85	24.82	25.72	23.3
SUMF1	22.38	22.2	23.55	23	24.76	24.82	24.11	25.14
CCDC34	22.64	20.1	22.91	22.92	23.58	24.82	26.36	25.23
PTDSS2	19.88	20.95	20.41	18.04	23.58	24.83	19.5	21.41
FAM120AOS	26.31	25.96	26.06	24.55	24.68	24.83	24.41	24.1
PLGRKT	18.76	19.31	20.94	19.76	25.11	24.84	29.84	26.19
C17orf49	25.91	23.21	24.91	23.71	23.58	24.85	20.91	20.5
SNCG	35.45	33.59	33.6	30.68	23.64	24.85	20.06	21.27
ARL16	24.6	25.48	25.3	25.8	24.96	24.86	26.25	25.09
GATSL2	22.17	21.44	20.63	22.64	22.08	24.88	19.25	22.65
RAD51C	23.23	19.57	23.33	23.5	26.95	24.89	32.59	28.89
AP1G2	25.99	25.27	25.27	25.78	25.67	24.92	28.39	26.3
PEX16	22.71	23.76	23.4	20.57	25.91	24.93	18.41	20.16
FAM98C	25.2	24.81	24.09	22.24	26.32	24.98	17.93	18.24
ST3GAL4	25.35	27.29	24.17	24.39	24.51	24.98	20.95	23.36
NRSN2	28.87	31.23	29.71	26.94	26.68	24.98	22.1	24.79
ALG12	22.85	22.68	23.31	22.96	23.06	24.99	19.34	20.56
TRMT2A	22.17	22.07	21.6	20.1	23.73	24.99	20.81	20.89
THAP11	24.98	23.97	24.07	22.47	24.41	24.99	20.67	22.01
ARRDC1	24.26	24.1	21.62	19.28	25.48	25	19.59	21.21
CDH13	26.71	25.69	28.26	27.54	24.53	25	28.04	27.16
UBE4B	21.34	22.64	23.11	22.35	25.72	25.01	27.01	25.17
GPR87	22.63	22.23	23.04	23.08	27.51	25.02	35.97	31.05
CMTM3	28.02	27.14	27.34	26.55	24.55	25.07	22.57	24.64
CLINT1	23.1	22.31	26.22	27.06	26.98	25.07	34.18	30.29
TMEM115	23.88	25.09	23.61	21.65	23.55	25.08	20.05	22.54
DHX38	23.13	22.2	22.34	20.72	26.89	25.09	22.96	25.58
DEXI	22.71	22.2	22.86	21.6	23.32	25.1	20.23	21.52
EMC6	23.34	20.93	21.01	19.52	24.7	25.13	18.3	19.13
PSMD11	25.93	26.16	25.48	26.92	27.04	25.14	26.79	26.54
BAZ2A	26.33	26.76	27.8	28.36	23.43	25.15	26.06	27.68
TBC1D10A	27.39	27.52	25.48	24	22.3	25.16	18.86	20.47
AK1	26.61	27.23	26.84	24.85	24.85	25.16	20.81	24.75
CDC42BPB	24.08	24.29	25.08	24.33	25.11	25.17	24.75	25.98
NUDT16L1	29	27.1	24.55	23.9	21.87	25.18	17.75	21.54
CSNK2A2	23.49	22.29	23.58	21.84	25.05	25.19	27.12	24.29
SRA1	26.57	25.03	25.03	23.92	25.56	25.2	21.26	21.92
TSPAN9	32.62	33.55	31.43	29.97	25.36	25.2	22.29	23.3
RAB40C	25.28	25.22	24.92	22.62	23.57	25.21	18.14	19.92
ZGPAT	24.77	22.69	22.53	21.83	23.9	25.21	20.27	21.57
CAV2	22.56	20.84	24.49	29.18	25.84	25.21	36.29	31.32
TNIP2	21.61	22.82	21.94	21.93	24.59	25.25	20.72	21.76
ACTR10	22.65	21.6	25.35	26.39	24.82	25.26	30.28	28.44
GMPPA	28.04	27.07	27.44	26.3	25.8	25.27	22.56	20.87
ACP2	24.72	26.68	24.84	24.63	22.9	25.28	19.65	21.55
ALCAM	23.35	22.24	26.75	30.75	25.97	25.28	40.97	35.03
GYS1	30.01	30.46	29.36	27.18	24.86	25.3	21.82	22.46
TMEM123	25.73	26.71	27.96	33.68	26.56	25.31	41.83	37.29
TBL2	26.75	26.11	24.89	24.6	25.77	25.32	23	24.09
CRELD1	24.72	28.5	24.71	23.92	24.2	25.34	21.57	23.84
AURKB	24.44	23.12	23.19	21.32	28.5	25.36	23.54	23.2
RGS12	23.64	22.96	22.24	22.15	26.1	25.36	22.64	23.28
NRAS	24.81	21.19	29.41	31.13	27.87	25.36	39.71	32.22
SNX15	21.11	22.78	22.31	21.35	25.07	25.37	20.21	21.11
ZNF511	21.76	22.3	21.09	18.96	22.07	25.38	17.22	18.59
PLSCR1	45.13	43.7	46.09	48.28	27.84	25.38	33.57	26.38
RGS10	24.7	25.46	22.83	23.97	25.3	25.41	26.05	26.12
YY1AP1	27.06	28.8	27.06	27.25	26.64	25.41	28.49	28.15
POLR1A	23.33	23.5	22.73	22.3	25.14	25.42	23.96	23.68
MYBBP1A	20.68	21.05	19.66	19.75	25.69	25.45	21.51	23.1
R3HCC1	26.91	26.76	26.05	26.64	23.81	25.46	20.55	23.01
ICT1	24.32	28.6	24.31	24.9	24.36	25.47	23.45	23.99
COL16A1	21.23	22.29	21.63	22.16	24.58	25.48	24.95	26.21

MTDH	22.14	20.99	23.07	27.26	25.48	25.48	32.56	30.15
WRNIP1	26.47	24.43	24.2	24.43	24.01	25.49	21.74	22.6
RIOK3	23.08	20.24	24.31	25.49	28.03	25.5	34.63	30.84
PTPRK	23.47	23.02	26.08	27.35	25.24	25.51	30.98	28.21
SEZ6L2	19.57	22.69	19.9	19.24	21.41	25.52	18.03	20.72
RNF130	27.32	25.99	27.07	27.47	25.98	25.52	23.85	25.66
HS3ST3B1	23.06	20.13	24.4	25.53	27.16	25.53	29.89	26.78
PEX11B	26.4	25.47	26.37	26.06	26.53	25.54	25.24	25.15
TMEM14A	22.79	26.16	26.9	26.83	26.49	25.54	29.75	26.55
P3H3	34.05	35.1	31.75	29.47	23.76	25.56	20.42	22.02
SLC35D2	25.85	25.98	25.55	25.58	27.67	25.57	27	26.34
FEZ2	24.86	26.38	25.54	27.52	26.1	25.59	32.2	27.41
ZNF217	25.28	23.09	26.9	30.68	26.9	25.59	35.69	32.11
ZNF768	30.93	32.12	30.44	28.94	22.3	25.61	19.76	22.3
RTCB	23.79	23.39	22.52	23.32	24.97	25.62	22.03	24.37
BPNT1	24.08	23.56	23.13	23.55	25.54	25.63	28.4	26.7
GPRC5C	26.91	28.42	25.2	23.14	23.41	25.65	19.7	21.3
BLMH	24.44	24.05	26.18	24.04	25.44	25.65	26.28	25.59
ZSCAN18	25.72	25.94	24.58	24.51	25.42	25.66	24.05	23.93
ISCU	21.71	22.33	20.98	21.88	26.82	25.66	28.95	28.31
ZNRF1	29.61	27.06	25.67	25.45	23.87	25.67	16.89	21.68
VAV2	25.3	24.69	25.92	24.65	25.62	25.69	24.08	24.83
RIN2	19.66	18.66	21.31	22.85	27	25.69	30.77	29.44
RCE1	26.5	28.5	26.2	25.02	26.8	25.7	20.55	24.74
SMIM4	22.11	23.92	20.13	22.15	24.83	25.72	16.7	20.19
NDFIP1	24.11	24.57	24.36	25.58	25.63	25.73	27.33	25.95
GOLGA2	25.89	26.4	27	27.55	26.92	25.74	26.12	26.7
TSC2	23.68	24.34	23.2	21.31	23.51	25.75	20.68	22.27
EGLN2	24.84	25.67	23.39	22.85	25.99	25.75	18.75	22.86
TM9SF3	23.79	23	26.65	33.42	26.91	25.75	42.54	38.79
NTHL1	23.8	24.27	19.52	20.38	23.94	25.76	20.17	17.02
PIGU	25.7	25.99	25.02	26.37	26.16	25.76	26.63	28.15
P4HA1	40.48	38.65	41.14	45.55	25.18	25.76	33.45	30.66
ZNF512B	26.1	27.66	26.59	28.01	25.18	25.81	25.01	25.1
MRPL32	22.06	21.25	21.59	24.03	27.78	25.81	31.21	29.92
BAP1	25.04	26.77	25.57	24.81	26.95	25.82	22.23	24.07
KDM5C	28.98	28.98	27.95	28.43	24.35	25.82	22.55	24.77
CHMP3	22.22	22.2	24.25	24.02	27.1	25.82	29.06	26.96
XPOT	20.77	20.46	23.94	25.75	28.14	25.82	36.09	31.72
PCBD1	27.37	26.03	29.6	25.55	23.48	25.84	22.11	25.79
SEC14L1	22.79	24.75	23.38	24.69	24.42	25.85	25.91	26.12
SNW1	24.5	24.42	26.45	30.58	28.86	25.85	34.71	28.89
ADCK2	28.9	28.25	26.4	26.31	23.5	25.86	20.23	20.61
PLEKHM2	26.63	26.27	26.5	24.98	26.2	25.86	22.08	23.69
MCM3	28.64	28.93	29.31	28.63	26.19	25.88	26.24	23.22
GAK	23.97	24	23.36	22.42	23.56	25.89	20.98	21.57
CITED2	29.22	27.33	28.5	26.14	24.02	25.9	21.2	21.84
CBX6	28.64	29.87	28.17	26.34	24.42	25.9	20.53	23.78
IPO5	22.07	20.81	22.58	22.3	27.13	25.9	30.3	25.9
C11orf68	25.02	24.84	23.77	24.47	24.25	25.93	19.18	20.45
TXLNA	26.96	28.97	26.94	25.76	26.61	25.93	24.65	25.45
PSMA5	25.72	23.17	25.17	25.58	26.27	25.93	29.7	27.33
DCBLD2	24.66	22.38	27.58	31.26	26.56	25.93	35.89	34.77
RBM23	26.35	25.39	25.24	24.91	24.65	25.99	24.31	23.22
PTN	31.57	34.09	32.84	37.97	23.85	26	31.84	29.46
PLCH2	22.63	22.16	21.87	22.38	25.53	26.02	24.65	24.3
GSPT1	24.14	23.01	24.98	24.97	26.04	26.02	30.32	28.35
TRIM27	25.79	25.77	24.81	23.62	25.6	26.03	24.84	24.4
FN3KRP	26.16	25.71	26.14	23.58	25.81	26.05	22.86	24.13
LEMD2	26.14	25.28	26.19	23.8	25.68	26.05	22.5	24.56
LAMB2	28.77	28.91	28.39	27.79	24.56	26.05	23.11	24.64
LSM10	29.02	28.79	25	23.75	25.04	26.06	22.73	24.75
GID8	25.58	25.77	25.14	24.84	25.88	26.06	26.06	25.91
EML2	23.17	23.86	23.01	20.96	26.69	26.07	22.47	23.76
STAMBP	26.27	25.66	25.76	26.21	28.6	26.07	32.8	28.09
RAB6A	24.43	22.54	26.64	29.31	28.49	26.07	33.73	34.34
KIFC1	24.78	22.73	24.48	22.62	27.1	26.08	24.45	23.13
LMBR1	24.32	24.21	26.28	27.92	28.1	26.11	34.92	31.01
TIPRL	25.09	22.51	22.49	25.17	25.63	26.14	27.83	27.53
STARD3	23.19	24.24	22.54	22.86	26.34	26.15	20.98	24.25
MVK	24.53	24.18	24.32	22.01	24.27	26.16	19.21	21.57
GOLPH3	25.99	24.24	25.12	24.82	24.57	26.16	25.97	25.53
AIDA	26.32	23.93	28.37	31.22	25.61	26.16	35.86	32.22
PLPPR2	24.58	26.6	23.57	22.8	24.57	26.19	20.51	22.61
COL12A1	26.88	26.1	28.64	32.35	24.42	26.19	32.73	33.27
HTRA2	22.42	23.45	21.62	19.25	27.13	26.2	21.59	23.23
ANKRD13B	18.53	19.64	18.93	18.15	26.16	26.2	22.39	23.57
APOPT1	22.5	22.37	23.44	22.8	24.65	26.21	26.62	25.41
CCDC12	21.46	23.73	22.46	24.9	24.12	26.22	21.43	21.68
PSMG3	26.66	28.18	25.87	25.58	26.43	26.22	21.6	25.69
NFE2L3	29.97	27.53	31.1	30.87	28.12	26.26	32.46	28.06
ZDHHC3	23.05	24.71	24.29	24.39	24.92	26.27	25.13	24.22
BOD1	23.35	22.97	23.15	24.89	26.96	26.27	25.24	24.95
ANAPC15	22.14	21.49	23.34	22.9	26.1	26.27	25.31	25.07
FKBP4	27.66	27.51	24.43	24.71	27.24	26.29	24.44	25.22
RNF11	25.44	23.82	26.73	30.07	27.85	26.29	34.22	31.71
DCAF8	25.75	26.08	26.49	26.36	28.22	26.3	27.76	27.84

PPP6R2	27.57	27.95	26.93	26.04	25.95	26.31	22.93	22.71
SKIV2L	25.42	26.21	24.22	23.29	25.33	26.32	22.94	23.05
NOP14	25.32	23.38	23.23	23.8	28.25	26.32	26.1	26.39
GRB10	25.42	25.59	26.24	24.92	26.61	26.32	27.05	27.69
RRM2	31.44	28.24	31.05	29.45	27.53	26.33	28.06	23.88
CENPH	24.99	23.18	24.14	23.59	28.5	26.33	29.93	26.13
SEC11C	29.97	28.01	24.13	26.5	28.02	26.33	35.77	30.93
PTRH2	24.28	21.84	25.71	27.23	31.59	26.34	32.89	32.29
NFYC	27.89	27.08	28.02	27.88	26.69	26.35	25.25	24.17
SCMH1	24.31	24.02	24.04	24.96	25.96	26.35	24.78	26.57
KRT10	25.31	23.35	22.69	23.28	25.16	26.36	20.62	22.15
CDK9	26.1	29.27	27.63	24.63	25.46	26.36	21.5	23.88
CRCP	23.69	25.71	25.74	25.59	28	26.36	25.69	25.15
TMEM243	21.59	22.13	25.81	24.61	28.21	26.38	34.42	29.19
GOLM1	27.46	26.22	29.6	29.66	28.2	26.38	33.51	30.27
TMEM69	23.61	23.84	26.17	27.68	29.42	26.38	35.65	31.18
GTPBP6	23.92	23.76	22.37	21.52	23.73	26.39	18.79	23.4
ZFAND3	28.17	28.88	27.92	27.38	27.06	26.39	24.54	24.99
CASC3	24.57	25.26	25.56	24.24	27.54	26.4	29.14	28.53
RMDN1	21.72	20.88	23.38	24.57	24.07	26.4	34.68	31.17
ZNF444	27.86	27.14	26.31	24.95	24.31	26.41	20.42	22.73
SFXN4	20.39	21.03	20.86	19.64	25.52	26.41	26.46	25.21
ZNF787	30.09	29.58	26.79	26.35	23.89	26.43	18.95	18.64
BCAS2	22.41	24.25	24.82	30.38	27.11	26.43	39.79	33.1
PNKP	26.82	25.82	26.02	22.5	24.51	26.44	21.46	21.45
MYO18A	26.75	26.56	24.89	25.04	26.34	26.45	22.36	25.53
FOXM1	23.38	22.7	22.29	20.69	25.17	26.46	24.62	23.83
MRPS18A	27.73	32.69	29.84	23.77	25.83	26.46	22.2	23.96
SAP30BP	26.81	27.62	26.86	26.01	25.74	26.46	26.18	24.65
PPP1R10	23.19	25.01	22.8	22.62	25.35	26.46	25.55	26.73
LCMT1	26.37	23.67	23.83	25.09	30.2	26.47	25.13	25.22
TUBG2	25.82	26.37	25.78	25.15	27.45	26.49	23.1	24.84
EIF5	24.84	23.62	27.26	29.11	28.5	26.5	36.36	32.84
CCDC130	26.91	23.56	24.15	25.33	23.79	26.52	25.06	26.96
PTP4A2	27.42	26.47	28.3	29.42	28.58	26.53	30.67	28.7
TGFB1I1	25.52	25.33	24.06	22.36	23.54	26.54	21.03	21.76
RAB35	24.99	26.02	24.47	23.25	24.99	26.54	21.19	23.4
ADIPOR2	23.89	24.54	23.43	25	26.76	26.54	27.44	28.16
GGCT	19.98	20.41	23.87	22.79	26.71	26.54	33.33	28.24
NOP2	24.57	25.23	24.73	22.91	27.58	26.57	23.89	24.83
HIP1R	24.08	23.99	23.51	21.99	25.42	26.58	21.84	23.11
PEPD	23	23.03	20.56	21.05	24.2	26.59	19.25	23.12
AKIP1	27.57	27.85	25.16	26.97	29.08	26.6	24.62	28.37
TARS2	26.65	28.25	26.85	24.77	27.17	26.62	25.08	25.68
MYLK	20.37	20.18	21.57	22.53	28.82	26.63	30.52	27.33
CDCA4	27.17	26.27	25.98	25.02	24.78	26.64	21.03	21.76
MCRS1	25.19	25.74	24.6	23.5	27.38	26.64	23.08	22.89
NID1	34.05	34.85	35.36	36.65	26.06	26.64	26.55	28.07
ZNHIT3	25.51	25.97	26.21	25.78	28.38	26.64	29.52	28.19
PELP1	27.01	27.13	26.23	24.38	25.17	26.65	21.32	23.38
CBWD1	21.13	18.47	21.92	28.84	26.65	26.67	33.9	31.49
COA6	21.24	19.92	20.24	22.15	30.05	26.68	28.84	27.58
PRR14	26.63	27.69	27.04	24.8	27.5	26.69	22.71	23.85
ZFP64	23.48	24.24	21.51	22.19	25.81	26.69	25.37	25.46
TIMP3	22.29	24.7	22.04	22.21	25.69	26.7	25.38	27.84
RIPK4	28.45	26.92	28.31	25.6	25.41	26.71	21.59	23.87
RAB10	25.31	25.23	26.84	28.21	26.71	26.73	30.99	29.65
WTAP	26.84	26.16	27.11	31.37	28.43	26.73	34.31	31.8
PDLIM2	28.99	28.1	27.1	23.96	26	26.74	19.18	20.32
MFSD14A	23.94	24.3	25.02	28.07	27.57	26.75	36	30.13
SLC35E1	26.63	26.18	25.14	26.47	26.23	26.76	24.99	24.59
MCM4	30.09	26.96	29.92	29.62	28.64	26.76	29.04	26.07
TSC22D1	30.98	29.53	31.9	31.84	27.79	26.76	32.15	31.69
MRPS33	26.84	26.94	25.69	23.62	31.62	26.77	29.34	29.76
RAB4B	23.91	25.64	25.02	21.72	24.89	26.78	20.18	21.76
GOLGA7	25.17	22.39	24.97	25.95	29.59	26.8	32.68	28.55
CAMLG	21.16	19.42	22.45	21.38	28.28	26.81	29.22	26.54
GNL2	25.62	22.74	28.29	32.51	30.38	26.81	38.04	35.46
DPF2	26.32	27.86	27.16	26.13	25.19	26.83	22.67	24.1
HNRNPH3	25.33	24.45	28.11	33.1	28.73	26.84	38.47	34.54
HPRT1	26.18	28.38	30.21	29.88	29.73	26.87	34.94	30.64
PICALM	26.24	24.25	28.1	31.83	28.35	26.87	40.08	35.69
GLG1	27.4	30.55	29.02	29.58	27.54	26.88	28.24	29.96
IGSF9	33.56	37.2	31.33	32.38	23.43	26.91	22.18	25.11
ZMAT5	27	26.33	24.07	22.05	26.18	26.93	19.21	20.72
FAM134C	27.58	31.3	27.66	27.13	26.39	26.95	25.5	26.65
LMNB1	31.35	28.65	32.08	31.25	29.67	26.96	33.61	27.57
MICAL2	24.83	23.81	26.04	23.64	27.84	26.97	29.08	27.55
NDUFS1	21.49	21.75	22.22	22.01	27.58	26.97	29.05	27.65
NDUFA9	26.99	25.99	25.93	27.33	29.63	26.97	28.43	28.16
SIRT6	27.95	26.4	24.68	23.32	26.22	26.98	18.01	21.74
SYDE1	28.19	26.62	25.24	23.56	25.46	26.98	19.74	22.7
NDUFC1	29.11	29.59	26.57	27.19	25.97	26.99	26.67	27.54
INAFM2	22.81	23.32	22.83	22.53	26.66	27	24.37	24.07
RABGGTB	20.87	22.53	21.8	24.67	26.15	27.02	38.01	35.41
PSMB8	32.46	31.39	28.66	32.31	28.77	27.03	26.62	22.23
ASB13	24.73	26.06	25.22	23.33	25.78	27.04	21.79	23.96
TAOK2	25.28	26.13	24.78	23.83	26.73	27.04	22.96	26.25

OGT	22.27	21.26	24.18	30.76	25.94	27.04	42.18	34.89
KIF2C	22.67	21.09	22.4	20.29	27.09	27.06	24.73	25.18
DERL1	23.34	23.66	24.02	23.01	25.29	27.06	27.09	26.21
MAPK1	25.48	26.47	25.69	28.61	29.7	27.07	31.56	29.42
LPCAT4	27.39	27.81	26.28	27.07	25.41	27.11	24.58	26.83
DYNC1I2	27.12	24.8	29.58	30.19	30.26	27.15	37.65	34.61
SNAP47	29.06	27.82	26.99	25.52	27.98	27.17	23.74	26.06
METTL21A	25.47	24.55	25.34	27.84	25.95	27.19	28.59	27.89
CDC42EP3	27.67	26.27	29.35	31.08	26.61	27.2	33.29	31.02
GPSM1	24.16	26.07	22.66	22.53	23.57	27.21	21.15	23.03
CSTF1	26.7	26.19	27.7	27.65	28.37	27.21	30.22	27.07
RPL36A-HNRNPH2	22.75	23.2	26.49	31.33	26.12	27.21	37.9	37.76
SPR	24.04	26.92	22.85	21.18	26.07	27.22	21.73	23.19
DENR	26.23	23.37	27.79	29.77	29.09	27.22	36.49	31.78
FEN1	28.71	27.94	28.96	27.03	28.36	27.24	28	25.59
ERAL1	30.08	30.73	30.61	28.46	28.7	27.25	24.74	27.6
STX4	24.23	22.74	24.37	23.81	27.05	27.28	27.55	23.78
UBL4A	25.8	26.07	24.87	23.89	27.73	27.28	23.19	24.86
KIF3B	25.14	24.24	25.92	27.19	27.54	27.28	30.2	29
SCAMP2	28.69	30.2	29	28.88	26.81	27.29	26.28	26.39
SNRPB2	27.16	26.15	30.47	29.35	29.34	27.29	37.31	34.26
CCNK	28.96	29.6	28.8	28.85	28.67	27.3	27.51	29.23
CCDC24	21.73	20.89	21.42	19.28	24.77	27.33	24.62	23.1
GPX2	28.69	31.92	27.11	27.74	25.91	27.33	21.55	27.24
SYVN1	27.35	26.85	25.68	25.5	27.84	27.36	24.46	26.04
SSBP3	31.78	35.07	31.38	31.03	28.91	27.36	27.51	29.7
PCBP4	21.08	21.43	20.51	18.76	26.3	27.37	21.75	22.45
PAQR4	27.79	29.84	28.24	26.27	26.26	27.37	20.26	23.55
SNX12	27.95	28.74	27.82	25.8	29.35	27.37	26.84	26.33
PAGE5	25.51	23.15	20.46	19.32	26.55	27.38	21.36	22.64
PROSC	27.41	26.58	28.49	28.8	28.53	27.41	30.64	29.54
ST5	26.26	27.48	26.82	26.88	24.8	27.43	24	25.81
PGRMC1	27.09	24.07	27.02	29.53	27.78	27.44	32.08	29.34
RPS6KB2	25.78	25.97	26.58	24.37	29.2	27.45	22.21	21.99
PCIF1	29.39	29.41	28.36	27.21	27.73	27.45	22.42	24.88
FAM192A	26.8	27.72	26.82	27.28	28.98	27.46	30.54	28.39
ANKRD9	25.95	26.01	25.72	23.08	25.87	27.47	17.96	21.24
GSTZ1	25.28	26.83	24.65	23.18	29.47	27.47	22.45	25.21
OSTF1	24.66	26	27.6	29.86	30.11	27.47	30.25	30.81
DNAAF5	25.44	24.94	24.04	22.75	27.05	27.5	22.16	24.14
PRKAB1	20.89	23.53	21.89	20.3	25.68	27.53	23.95	24.47
NTAN1	22.78	25.09	23.75	21.5	27.25	27.54	27.14	26.27
UTP11	28.14	24.35	27.3	30.16	28.96	27.59	34.3	28.62
CHCHD1	27.02	27.05	25.03	26.97	26.53	27.61	25.34	28.54
DUSP14	21.89	20.29	20.64	20.64	29.39	27.62	26.79	26.11
GAS6	34.09	32.6	31.06	28.78	25.97	27.63	20.91	23.62
MAEA	25.52	25.66	24.51	23.39	27.49	27.63	24.19	25.19
STK24	28.91	30.68	28.72	28.25	29.14	27.63	28.64	27.95
PTPN14	26.57	25.15	27.38	29.03	27.93	27.63	32.01	30.02
GALNT11	26.3	26.55	26.07	28.47	30.33	27.63	32.03	31.14
SNAPC2	27.77	29.13	27.27	24.63	26.5	27.64	17.41	21.71
WDR26	25.24	24.21	27.33	30.04	27.26	27.64	38.56	33.91
ACTR1B	27.13	29.54	28.04	26.21	24.62	27.65	21.9	24.58
BNIP3L	30.74	29.65	35.2	37.95	27.54	27.65	40.61	34.3
ATPAF1	23.94	24.18	23.57	24.74	23.88	27.66	25.06	25.09
SERINC1	23.97	21.67	28.81	34.94	27.87	27.66	49.31	38.97
XPNPEP1	28.08	28.08	27.65	27.06	28.71	27.67	27.1	26.22
PTPN12	28.3	26.95	30.8	34.91	29.18	27.67	44.75	39.57
PNRC2	26.75	26.39	29.49	34.14	28.28	27.67	44.57	40.21
ORAI2	28.24	28.76	27.31	26.58	26.38	27.68	23.55	26.37
HSD17B7	24.3	23.49	23.53	24.03	27.54	27.69	33.39	30.59
CAPZA2	28.16	29.55	27.53	28.47	28.78	27.7	41.83	35.86
NDRG1	46.84	41.09	43.67	39.33	25.67	27.71	28.38	25.43
KIRREL	30.35	29.62	28.38	30.66	26.54	27.72	26.66	27.24
PYGL	27.87	28.42	28.01	28.04	28.9	27.72	31.17	29.38
POMZP3	23.63	24.68	24.39	21.92	23.55	27.73	18.35	22.42
PPP1R12C	25.99	25.93	24.16	23.55	27.44	27.74	22.73	25.03
HS6ST2	25.16	24.42	25.73	30.51	27.56	27.74	37.66	35.66
URI1	23.77	22.4	25.33	26.05	27.65	27.75	34.19	32.5
APTX	23.59	25.16	26.16	25.47	29.06	27.76	27.25	27.62
MGST1	24.89	25.28	23.76	25.94	28.02	27.78	32.76	30.31
CETN2	28.32	30.96	32.38	29.85	28.26	27.8	32.02	28
C9orf69	26.36	25.23	25.66	23.45	26.4	27.81	20.86	22.2
UQCC1	25.34	26.29	25.3	24.6	27.95	27.82	26.5	27.93
TAX1BP1	23.42	23.96	27.6	28.65	27.15	27.82	40.73	37.07
NFKBIA	32.03	31.97	30.79	29.61	28.09	27.83	27.18	25.56
CD82	27.93	28.79	26.52	26.49	26.67	27.85	22.37	23.72
ZNF219	25.58	23.67	23.52	22.57	27.01	27.87	19.51	21.92
NPEPL1	29.21	28.05	26.94	25.79	25.6	27.88	24.11	25.35
MEN1	25.96	28.11	24.32	23.61	25.84	27.88	22.41	25.38
CRIM1	24.76	21.97	26.51	30	29.47	27.88	35.71	31.04
NDUFC2	26.78	26.94	25.67	25.75	26.42	27.89	25.56	23.97
RTN3	30.18	31.02	30.66	29.66	29.82	27.89	32.58	32.01
ATXN10	25.67	26.2	26.78	27.77	29.05	27.91	33.56	29.98
ATP5SL	28.48	28.66	27.06	26.83	29.64	27.93	22.78	24.89

EMC3	26.16	26.71	23.83	26.21	29.27	27.94	30.31	25.87
EXOSC1	26.37	26.13	26.59	27.91	28.26	27.94	30.73	29.23
WWTR1	28.95	26.65	31.59	33.83	29.27	27.94	35.18	31.88
SPSB3	24.87	25.05	25.92	24.52	24.69	27.95	24.8	22.31
BSDC1	29.13	27.84	27.63	29.34	29.05	27.96	29.94	30.27
DDX19B	27.25	27.23	28.57	27.5	28.31	27.97	26.72	27.22
FAM65A	26.9	28.64	26.14	25.2	26.85	27.98	22.64	25.48
RNF4	28.3	28.56	27.87	27.73	28.17	27.98	26.19	28.17
RIC3	23.01	21.21	27.39	36.98	26.1	27.98	59.27	48.77
SYNGR1	27.62	29.55	29.68	26.56	26.78	28.04	21.88	23.8
MALSU1	26.57	26.67	25.31	29.66	28.07	28.07	28.24	27.96
CPSF7	28.45	28.65	28.88	29.39	30.01	28.08	31.02	30.6
BTG2	21.95	22.1	22.64	21.85	31.3	28.09	28.87	29.74
MGST2	26.17	29.38	28.08	25.51	27.29	28.12	29.53	26.59
C7orf73	28.41	29.51	31.5	30.12	31.19	28.12	33.12	31.08
GPS2	31.7	30.98	30.18	28.99	30.98	28.13	26.49	27.82
FBXO32	26.2	24.64	26.94	27.33	30.78	28.13	32.92	29.69
PPP1R7	27.03	30.99	26.48	26.77	25.37	28.15	25.69	26.85
EIF2B2	28.16	29.24	26.58	26.06	28.24	28.16	25.53	27.25
RBM10	31.81	32.15	32.82	31.35	30.21	28.17	26.16	27.51
PSMB10	30.03	26.88	24.58	23.21	25.94	28.18	21.54	21.35
RBM34	23.09	27.41	26.62	27.68	28.53	28.19	35.44	30.63
OLA1	25.36	24.22	27.41	27.87	30.85	28.19	37.61	31.98
C6orf106	26.88	28.43	27.19	26.05	27.24	28.2	25.23	25.92
GNG12	26.61	24.53	29.66	31.78	32.23	28.23	42.46	36.51
BAK1	26.69	28.26	24.92	25.39	27.14	28.24	21.73	23.56
IDI1	23.22	23.45	23.15	27.8	27.78	28.24	39.05	34.94
MSTO1	24.74	25.94	25.36	25	28.65	28.25	26.23	26
SAMD1	32.41	29.41	30.05	26.97	25.62	28.27	19.94	21.62
HEXIM1	30.06	30.2	32.01	30.78	28.74	28.3	28.32	27.31
IKBKG	28.79	26.67	25.77	25.92	26.62	28.31	21.89	23.89
MICA	25.15	28.71	24.94	23.84	27.79	28.31	27.44	27.67
ZMIZ2	27.45	26.96	27.06	26.86	27.86	28.35	25.81	25.86
XPC	23.99	23.71	24.89	24.21	30.38	28.35	29.56	28.39
CYB5B	30.5	29.7	29.53	28.59	29.69	28.35	30.39	29.18
SEC16A	27.8	27.52	27.21	27	25.56	28.36	25.34	27.09
PSMD10	28.28	28.79	27.77	30.2	30.38	28.36	33.45	28.89
ASPSCR1	29.1	28.5	26.4	24.77	28.74	28.37	21.98	22.64
DMAP1	24.98	25.15	25.28	24.56	27.83	28.37	24.97	25.1
PNRC1	30.44	29.99	30.91	30.06	28.39	28.37	29.04	27.63
NPRL3	26.82	25.49	24.08	22.55	26.48	28.38	21.32	24.56
FAM53B	28.77	29.05	29.2	26.62	27.39	28.39	22.7	24.73
AAAS	33.1	32.96	30.78	29.49	29.96	28.39	24.67	26.05
SSFA2	18.71	18.6	21.97	24.31	28.22	28.4	44.23	37.18
TRIM11	26.08	27.44	25.91	25.18	26.19	28.41	21.94	23.65
ATG4B	24.87	26.78	23.54	23.93	25.73	28.42	22.36	25.41
NPC1	24.94	26.84	25.86	26.61	27.68	28.44	29.13	29.01
PUM1	30.17	28.15	32.16	32.9	30.24	28.44	33.73	33.26
GALK1	30.44	28.66	27.06	23.57	26.66	28.45	21.41	20.87
STX6	28.53	26.63	29.97	30.87	28.51	28.45	31.89	29.95
CCDC137	25.5	26	26.28	25.68	29.24	28.48	26.4	27.84
SPRY1	31.05	31.79	32.94	33.54	27.82	28.5	28.37	31.18
KTN1	23.42	22.33	25.84	30.92	28.36	28.51	46.8	41.36
MRPS30	23.1	22.84	23.36	23.9	25.47	28.52	28.68	28.31
HERPUD1	29.78	31.17	30.27	30.21	29.21	28.54	29.1	30.02
ZMYND19	26.81	25.65	22.61	23.65	27.75	28.55	22.87	23.55
TACO1	28.05	29.18	27.12	26.82	29.23	28.57	26.21	26.21
OSBPL9	25.79	23.64	30.05	33.18	27.36	28.57	43.12	36.06
PMF1	31.07	30.44	30.69	28.49	28.56	28.58	27.94	23.87
BIRC5	25.94	24.17	24.15	22.36	28.92	28.58	24.42	25.56
STRADA	28.4	28.31	27.45	28.15	27.1	28.6	30.36	29.03
RNPEPL1	29.72	30.98	28.94	27.7	25.44	28.64	21.19	23.21
GNA12	26.63	29.34	27.55	25.8	27.37	28.64	25.95	26.31
PQLC1	28.59	29.5	27.43	25.35	26.82	28.65	20.28	22.07
UBALD1	28.96	28.17	24.73	25.69	27.98	28.65	21.69	23.85
ADCK3	23.04	22.94	23.08	20.09	30.06	28.66	25.67	25.11
EMP3	35.93	32.73	32.89	33.93	24.92	28.67	21.81	26.41
DLGAP4	29.16	28.64	29.61	28.26	28.97	28.67	26.52	27.24
BCL2L2-PABPN1	31.91	35.78	33.4	35.08	32.87	28.67	36.45	36.35
MAPK12	24.67	25.23	24.35	23.05	26.06	28.69	25.46	25.93
C1orf35	32.31	29.18	28.57	28.63	27.44	28.71	21.83	25.13
CTSL	27.26	25.83	26.69	29.44	27.75	28.71	30.12	29.72
NUB1	28.5	27.73	30.34	29.37	31.73	28.72	33.66	31.43
COPS5	24.26	24.13	26.78	28.26	28.84	28.73	33.23	28.81
SCARB2	28.75	27.23	30.73	33.84	29.5	28.74	38.53	35.09
FAM3A	26.76	25.65	24.76	24.09	27.21	28.75	21.32	25.63
TPRA1	30.61	30.34	30.17	28.28	29.35	28.77	23.47	24.49
NDUFB3	27.22	29.21	28.19	27.56	30.16	28.77	37.21	32.58
STC1	35.97	32.79	36.99	39.71	26.4	28.77	35.86	34.69
ZNF598	24.4	23.5	24.56	22.69	26.49	28.78	22.6	24.26
PGAM5	26.9	27.73	25.58	24.1	28.15	28.8	22.02	25.08
TGOLN2	28.49	27.87	31.02	31.63	29.22	28.81	34.23	31.07
TMEM63A	27.65	29.03	28.92	29.31	27.62	28.81	29.61	31.81
RYK	27.31	24.99	28.15	31.29	30.95	28.82	37.8	32.94
OAT	23.54	24.05	25.23	27.86	30.48	28.83	37.92	35.18
PAPOLA	25.62	23.15	29.98	33.02	31.04	28.83	46.3	40.65
TMPO	26.12	24.2	28.63	29.31	29.79	28.84	35.1	30.87

C16orf74	28.25	29.45	26.05	24.51	27.9	28.86	21.22	24.08
NUP98	28	28.23	27.72	27.98	28.79	28.86	31.02	29.86
SURF2	27.08	27.82	26.05	23.66	27.86	28.88	22.1	24.76
PDCD2	28.28	25.25	26.65	25.58	29.6	28.89	28.1	28.4
TACC3	27.49	25.33	25.55	24.5	29.77	28.9	26.4	27.67
RAB8A	29.41	30.78	30.71	30.9	32.17	28.92	32.08	31.34
TSPAN13	30.62	28.63	31.81	35.12	28.76	28.92	35.01	33.71
CPTP	28.57	29.39	26.17	24.95	26.18	28.93	21.81	24.05
HAGH	25.55	26.12	22.11	23.56	26.62	28.93	22.35	24.89
PRCP	31.81	31.17	30.18	30.68	30.4	28.95	31.25	29.99
PSAT1	28.06	27.27	29.31	28.92	28.53	28.96	34.35	29.77
ABHD11	29.1	30.81	30.8	27.46	29.7	28.97	24.81	28.64
DARS	29.35	27.92	31.47	33.67	31.28	29.01	40.93	35.21
RBM8A	29.33	29.14	31.87	32.31	31.86	29.03	33.02	29.96
CUL1	28.37	27.09	30.75	32.45	29.02	29.03	34.52	32.83
RDH11	27.66	29.45	28.22	29.71	32.28	29.04	35.33	32.86
SCP2	28.2	27.98	29.82	31.81	31.9	29.06	43.73	38.81
RPUSD3	28.06	28.89	27.04	24.63	27.71	29.09	24.65	27.58
UBAC1	25.8	25.41	25.67	23.72	26.95	29.1	22.21	24.8
DNAJA3	25.34	26.45	24.72	23.66	29.66	29.1	26.33	26.06
ARFGAP2	30.88	31.57	28.8	29.35	28.84	29.11	26.19	26.17
ABCG1	19.22	23.45	19.77	22.02	28.6	29.12	27.26	29.94
GHDC	28.67	29.1	28.76	27.02	27.71	29.13	24.51	24.63
ARF6	29.92	28.91	31.96	32.33	27.86	29.13	30.93	31
ADNP	29.01	28.61	31.42	31.65	29.13	29.13	34.86	31.81
FLYWCH2	32.8	33.34	31.58	28.53	26.58	29.15	22.7	24.82
MLLT6	30.51	29.78	29.03	29.18	26.86	29.18	26.49	28.17
AFG3L2	23.75	27.27	24.24	25.77	31.8	29.18	31.52	29.87
A4GALT	32.65	34.32	30.91	29.28	28.64	29.2	23.58	25.62
PPME1	35.99	35.62	34.36	34.37	30.16	29.2	29.41	27.75
CD55	27.22	25.02	27.32	31.23	30.01	29.2	37.61	31.01
API5	27.4	23.95	30.66	32.06	29.84	29.22	39.85	33.57
CCDC106	31.9	33.73	30.1	26.66	27.43	29.23	20.61	25.14
STX5	31.49	31.58	29.27	29.97	28.75	29.23	25.09	28.68
ORMDL3	30.1	30.06	30.77	29.24	29.82	29.27	24.59	28.78
AKAP8L	29	28.68	29.79	27.68	30.65	29.28	26.98	27.27
BOLA3	29.14	29.25	31.28	30.96	27.85	29.3	28.67	28.74
NPEPPS	27.73	28.96	30.44	29.77	29.54	29.31	33.52	31.91
ERLIN2	30.56	27.87	30.47	32.06	33.11	29.31	34.5	32.49
GUCD1	25.53	26.51	25.91	24.58	27.01	29.4	25.02	25.94
SUGP2	25.78	23.91	26.09	29.32	30.37	29.45	38.14	33.23
SMARCD3	30.23	27.82	28.7	27.9	29.61	29.46	23.35	26.97
UBR4	26.32	26.14	27.25	27.5	29.26	29.47	30.33	30.17
TPM1	30.87	28.69	31.47	33.08	31.88	29.48	34.53	31.17
ECSIT	27.1	27.16	22.87	22.83	31.8	29.49	24.14	25.56
BRD9	24.94	27.12	25.48	25.79	28.37	29.49	26.17	27.1
ABCF1	29.71	29.98	30.56	28.93	30.23	29.49	27.41	28.79
SLC25A13	25.12	24.96	25.74	26.95	29.7	29.49	30.92	30.04
AURKA	23.92	22.74	24.71	24.84	31.63	29.49	33.3	33
PMM1	30.09	29.76	29.41	25.89	28.9	29.5	25.55	24.64
PTGFRN	32.82	33.46	33.49	33.66	28.66	29.51	29.48	29.77
CECR5	32.51	29.43	31.75	29.47	28.83	29.53	25.39	23.93
EIF2B5	29.27	29.22	27.85	27.43	31.19	29.53	30.5	27.45
ABCF3	28.23	27.98	27.02	27.14	29.33	29.54	26.08	26.81
FARSB	26.57	24.64	25.48	26.05	31.3	29.54	36.76	28.81
BCCIP	23.6	25.6	27.03	29.53	27.45	29.57	38.5	34.02
FTSJ1	25.43	25.73	25.33	23.86	29.36	29.59	24.69	26.48
KLF5	24.95	24.21	26.54	27.86	28.09	29.59	30.92	30.65
PDHB	28.72	29.03	28.47	29.02	29.23	29.61	32.51	32
NDUFA10	25.64	25.87	25.19	26.32	29.63	29.65	28.25	28.81
PAM16	28.12	27.98	24.33	24.01	30.34	29.67	26.07	27.51
SEC22B	29.39	30.48	31.01	31.61	30.84	29.68	36.85	35.09
COPRS	32.21	32.82	31.92	28.87	30.39	29.7	26.43	26.55
CRLS1	30.62	29.1	30.05	25.92	30.46	29.7	27.39	26.64
PMP22	29.77	31.36	30.78	29.99	28.81	29.7	28.58	31.09
BCAS4	31.35	33.27	31.6	30.2	30.01	29.71	23.46	28.42
USP39	29.58	30.84	31.88	29.61	31.78	29.74	30.09	28.61
SDCCAG3	24.13	23.36	23.14	21.66	29.8	29.75	28.62	27.54
SURF1	26.82	27.53	25.11	23.18	30.01	29.76	21.53	26.41
LAS1L	32.91	29.18	31.25	29.62	33.43	29.76	28.94	29.53
CNOT7	26.7	27.52	29.09	31.83	32.8	29.76	39.9	37.36
MCCC2	25.06	23.81	25.18	27.23	28.73	29.8	34.44	32.1
MED16	31.23	31.21	29.37	26.94	27.68	29.81	20.74	22.61
ELK1	27.47	30.35	27.35	27.09	26.87	29.81	22.83	25.6
CWC15	28.98	28.35	30.06	34.42	31.66	29.81	37.98	37.03
DTX2	28.26	26.39	26.71	24.91	27.7	29.82	25.12	25.85
YME1L1	27.73	24.56	30.82	33.75	30.37	29.82	42.76	36.32
ALAS1	30.01	32.13	29.8	29.67	28.67	29.83	27.47	28.03
C7orf49	28.21	30.36	28.57	28.48	30.45	29.85	28.43	26.37
LAMC2	31.45	30.55	29.71	30.77	30.14	29.86	32.11	28.16
VIMP	28	27.31	26.12	30.76	30.82	29.86	28.93	29.79
WDR82	31.66	31.94	30.94	31.95	31.16	29.86	29.83	31.02
TAP1	34.73	35.18	33.09	30.94	30.62	29.87	27.63	26.68
TMEM30A	27.43	26.34	31.92	38.26	32.76	29.87	51.03	42.07
AJUBA	30.33	28.93	30.28	30.28	31.12	29.89	28.13	27.97
KIAA0141	28.17	24.68	26.64	26.69	28.95	29.9	25.73	26.79
NSDHL	30.22	31.21	30.08	28.32	28.37	29.91	26.72	27.72
PPP2R5D	28.55	28.16	26.86	26.11	29.58	29.92	26.95	28.31

GRK5	32.73	32.3	33.21	31.52	27.92	29.94	27.78	31.06
MFSD14B	25.65	25.45	29.36	29.59	28.95	29.95	33.99	32.63
CISD3	29.36	28.35	27.25	27.43	30.27	29.96	22.54	25.09
RSL24D1	26.21	26.9	27.2	29.48	30.55	29.96	39.3	33.73
TRIM74	28.44	28.76	29.23	32.11	29.35	29.97	32.26	31.98
TNFRSF12A	27.98	26.54	28.49	26.05	32.69	29.99	24.3	21.08
TARDBP	30.46	28.98	31.39	32.75	32.68	29.99	40.39	37.4
ZBTB7B	31.1	31.41	29.42	30.24	31.13	30.02	25.08	26.83
CDC42EP4	36.54	36.12	33.1	32.34	32.56	30.02	26.84	30.33
SCARF2	27.08	26.62	26.15	25.5	26.25	30.03	19.86	24.03
INTS3	28.34	27.7	27.96	27.63	28.14	30.04	30.07	30.38
DDX54	27.9	28.49	26.87	25.36	29.83	30.1	23.27	24.89
PTRHD1	32.16	27.75	31.14	29.61	29.89	30.1	25.57	25.93
AIMP2	29.04	27.72	25.29	26.57	30.16	30.1	25.46	28.24
PYGO2	32.68	34.06	31.65	30.82	30.77	30.11	28.49	30.01
XBP1	38	37.68	36.21	36.01	32.59	30.12	31.13	28.6
CDC42EP1	28.73	29.7	28.69	25.93	30.45	30.13	21.95	24.19
FKBP3	30.09	32.26	32.1	33.93	33.16	30.13	40.4	36.29
MTX1	31.77	31.92	27.8	27.38	31	30.14	25.62	26.13
UBE2A	28.87	29.01	29.17	29.5	32.27	30.14	32.91	30.99
NOP58	27.85	26.03	29.46	32.73	30.53	30.15	41.44	37.31
CREB3	31.58	34.78	31.86	31.27	27.53	30.16	24.7	27.46
ACD	28.25	30.14	26.87	26.6	29.89	30.17	22.92	25.15
ASL	31.86	31.53	30.86	27.4	29.92	30.18	22.83	25.12
ZC3H7B	33.39	32.84	33.02	32.59	29.76	30.18	24.3	27.27
THEM6	31.05	31.8	29.55	27.76	27.87	30.19	22.12	22.56
WSB2	22.95	24.97	23.58	24.19	30.05	30.21	30.27	28.41
TRAPPC12	27.13	29.28	26.71	25.73	29.18	30.23	26.27	25.8
IRF2BPL	32.59	32.64	33.21	31.45	27.7	30.24	22.62	23.14
IAH1	30.92	30.51	28.56	29.7	30.25	30.25	26.86	27.41
POLR2K	27.87	31.71	31.25	31.77	31.87	30.25	38.63	34.34
CAT	28.22	28.04	30.89	30.43	30.4	30.26	34.36	31.28
ACTR3	28.89	27.43	30.73	33.23	31.82	30.26	39.19	35.39
ISG20L2	29.96	31.44	29.29	29.52	30.17	30.28	28.24	31.06
PGM1	33.75	34.31	34.39	33.06	27.17	30.34	26.92	26.99
MAU2	29.53	26.35	27.88	26.54	29.75	30.35	26.35	26.73
FNTA	26.5	26.68	27.46	30.11	30.07	30.36	35.75	32.55
PRKD2	33.39	34.39	30.47	29.54	26.7	30.37	23.78	26.88
MLLT1	31.4	29.4	30.26	30.32	30.12	30.37	24.61	27.01
SEPHS2	28.78	27.9	27.59	26.78	31.66	30.38	28.45	30.39
SV2A	27.29	30.59	28.45	30.16	29.9	30.38	28.68	30.62
PIEZO1	27.48	26.95	27.21	26.54	27.9	30.4	23.38	25.64
SDF2L1	31.43	32.72	31.18	25.06	25.93	30.41	19.28	19.75
ACOT8	29.76	29.65	27.56	25.56	29.46	30.44	23.02	25.49
COMMD3	30.83	29.71	28.48	29.42	30.05	30.45	31.9	29.58
GTF2F1	31.3	32.77	31.52	29.13	30.58	30.47	24.85	28.29
SLC39A14	26.79	26.22	27.05	26.84	31.58	30.49	32.93	30.89
COPG2	26.91	29.12	27.86	27.43	30.13	30.5	32.03	31.65
VCL	29.06	30.66	30.1	31.45	31.04	30.51	32.56	31.67
TMEM208	30.59	30.22	28.49	27.67	30.17	30.52	26.7	24.6
CSPG4	32.56	36.04	30.77	30	26.57	30.52	23.01	28.08
DNAJC11	29.96	29.52	30.26	28.14	30.58	30.54	29.39	29.66
LDLRAP1	29.35	32.08	29.46	28.9	27.95	30.55	26.49	27.14
POLD1	34.06	32.9	31.11	29.39	28.02	30.56	22.54	22.12
CHTOP	28.38	28.07	30.09	28.17	29.52	30.56	28.11	27
NIT2	30.01	25.33	29.58	31.97	28.29	30.56	32.95	32.75
LSM3	31.02	30.91	29.15	30.61	32.01	30.58	32.98	33.46
NECAP2	31.33	32.17	29.41	29.18	32.21	30.59	27.76	30.99
TRIOBP	34.7	34.83	31.76	30.07	30.42	30.6	26.93	26.9
HS6ST1	34.99	35.51	34.6	30.77	30	30.6	23.95	27.61
TFRC	24.26	22.97	26.89	30.06	33.61	30.6	42.36	34.54
CNIH1	29.88	30.29	30.85	30.42	31.57	30.6	37.46	35.36
OSTC	28.45	31.66	30.04	31.9	32.11	30.6	38.18	35.43
RERE	27.4	28.98	28.76	30.45	29.22	30.61	26.84	28.9
NECTIN2	33.49	36.1	30.68	31.47	30.05	30.62	23.79	27.37
SEC23A	29.89	28.03	30.35	33.31	30.81	30.64	40.31	36.9
BCR	29.87	31.03	30.35	27.41	28.56	30.65	24.88	27.41
TGIF1	27.77	30.49	29.11	30.55	28.52	30.67	29.12	30.87
TM9SF2	28.65	28.08	31.07	32.35	29.57	30.69	37.17	32.97
BRD7	29.92	28.8	32.8	33.4	33.02	30.69	38.54	34.63
CDK11B	32.3	30.65	30.89	29.11	30.48	30.7	28.8	29.12
UBE2T	30.65	26.88	29.45	29.98	31.19	30.7	36.26	32.9
YY1	28.38	28.32	32	29.72	28.66	30.73	29.79	29.36
DLD	24.88	23.12	27.09	30.69	30.36	30.73	43.79	37.92
XPO7	28.93	29.78	29.63	29.99	29.92	30.75	32.82	30.58
DDX21	22.75	20.21	26.61	29.63	34.77	30.75	47.46	42.47
SAFB2	31.28	29.46	28.65	29.72	32.55	30.77	29.19	30.31
DIDO1	31.26	29.38	31.96	32.02	31.12	30.78	33.59	31.21
ATP6V1B2	28.76	28.24	27.31	27.97	30.33	30.79	31.52	28.42
PAIP1	30.59	28.42	32.9	32.85	31.34	30.8	37.55	33.76
NDUFA6	28.57	29.14	28.48	27.84	30.47	30.82	26.92	28.1
MACROD1	28.32	26.25	27.67	27.99	27.46	30.84	23.14	24.47
NUP93	29.33	30.78	29.53	28.28	29.21	30.85	28.33	28.18

NR2C2AP	26.85	27.47	27.63	28	29.72	30.86	27.95	26.3
RNF7	28.59	30.3	31.53	29.04	31.99	30.87	31.5	32.7
SEPW1	39.84	40.84	37.92	34.32	33.29	30.89	28.35	31.74
TRIP13	26.53	23.9	25.99	25.47	32.96	30.9	34.76	27.79
EMG1	30.99	30.8	30.53	29.64	33.87	30.93	30.95	31.14
C8orf82	30.1	31.37	30.25	26.59	27.26	30.94	20.1	24.39
MAP3K11	31.8	32.79	29.56	28.79	29.5	30.99	22.03	25.67
SLC35E2B	28.76	29.55	30.15	29.66	30.78	31.06	30.81	30.31
MTHFD2	29.85	27.66	31.53	30.36	34.38	31.06	35.99	35.26
ZNF655	27.95	26.95	28.48	33.42	31.54	31.06	44.97	41.09
ALKBH5	33.4	35.26	33.08	32.61	28.99	31.07	24.41	26.97
MAVS	29.25	29.82	28.93	28.69	30.57	31.07	29.37	30.51
DKC1	31.12	29.65	31.26	31.92	35.39	31.07	37.29	34.85
WDR45	27.17	30.24	28.26	26.66	28.94	31.08	26.46	28.32
CBWD5	32.67	25.45	32.22	32.4	32.37	31.09	40.07	38.64
PCNX3	27.09	28.47	27.6	26.33	27.77	31.12	25.72	28.4
MRI1	30.89	26.34	28.53	29.28	28.33	31.13	27.04	28.88
TEX261	27.85	29.82	29.53	30.04	33.24	31.18	32.77	33.82
SOX12	32.44	33.66	30.66	29.47	29.26	31.19	23.08	26.04
EHMT2	32.85	31.46	31.17	29.7	29.16	31.2	26.03	28.11
HMOX2	29.81	29.7	28.24	28.27	32.7	31.22	26.82	28.79
STEAP3	26.47	26.92	25.13	25.97	30.1	31.23	28	29.23
IMPA2	36.43	35.35	34.03	34.05	30.29	31.23	25.45	30.23
ABCC1	30.63	30.63	30.25	31.24	30.66	31.25	32.21	30.64
NCBP2	26.77	25.63	29.58	31.89	34.81	31.25	43.48	40.5
USE1	33.52	34.92	31.54	32.12	34	31.28	25.93	26.9
SEC31A	33.31	32.51	33.26	34.09	33.92	31.28	36.29	34.96
NECAB3	27.89	26.66	27.15	26.72	29.67	31.29	26.27	27.75
CDT1	34.31	32.98	31.01	29.71	32.26	31.31	23.46	23.74
LGALS3	28.43	30.52	26.89	27.79	30.64	31.32	29.81	30.92
HIGD1A	28.21	28.76	28.62	30.58	33.28	31.32	39	35.15
DEF8	33.4	32.4	30.11	28.84	30.72	31.33	24.76	27.12
LSM12	32.16	32.88	32.06	35.54	36.23	31.35	36.14	36.33
SIRT2	29.4	31.42	29.19	27.99	31.07	31.39	25.07	29.44
ITGB1BP1	25.87	25.11	29.15	29.42	31.12	31.39	34.4	29.62
UROS	29.6	30.23	30.03	28.06	31.29	31.39	32.15	29.76
PHLDB1	35	34.75	32.75	33.53	31.13	31.39	27.42	30.22
MINK1	32.44	31.97	31.15	30.4	30.01	31.4	29.25	30.14
DECR1	27.29	26.55	28.53	30.1	33.59	31.4	36.38	34.19
EHBP1L1	31.71	30.1	29.21	28.68	28.75	31.42	25.41	27.51
SIL1	28.75	30.09	29.07	27.52	29.23	31.5	28.66	29.22
BOLA2B	25.37	23.68	22.6	21.5	32.46	31.51	24.66	26.85
BOLA2	25.37	23.68	22.6	21.5	32.46	31.51	24.66	26.85
PPAN	30.82	26.96	29.19	25.12	31.17	31.52	27.24	26.57
CLUH	27.87	26.84	26.78	24.9	30.97	31.54	24.77	26.98
ANXA4	29.8	30.3	30.26	30.49	33.13	31.55	37.1	32.72
SBNO2	32.03	31.63	30.2	27.9	29.4	31.57	24.14	25.35
HSD17B4	29.08	29.77	29.72	32.06	31.05	31.57	38.74	35.19
ZC3H11A	26.93	25.11	32.5	39.18	33.13	31.57	50.48	44.57
NSFL1C	33.44	34.84	33.23	32.95	32.8	31.58	29.47	29.26
METTL9	29.85	27.57	30.48	29.59	31.9	31.58	36.09	33.93
AIG1	30.24	30.73	29.72	31.3	30.36	31.58	35.02	34.1
ACTA2	28.65	29.07	27.73	28.71	31.86	31.59	25.63	26.74
VEGFB	33.58	33.2	31.49	30.76	28.35	31.61	23.2	25.79
P4HTM	32.42	31.87	31.02	28.36	29.26	31.62	25.31	25.82
EDEM1	26.63	25.17	30.27	32.65	33.16	31.62	38.02	34.88
ANKRD11	30.95	29.24	31.63	33.89	31.78	31.63	32.83	32.75
HES2	23.49	23.43	22.84	22.49	29.45	31.65	30	30.14
DAXX	32.72	32.14	31.73	30.45	31.65	31.66	29.03	29.68
PFKM	28.42	29.64	28.83	27.98	33.09	31.66	31.05	32.45
POLR3D	29.04	30.66	29.96	28.83	33.45	31.69	30.75	28.19
TMEM165	29.38	27.93	29.46	28.16	31.77	31.72	30.72	30.21
MRGBP	31.12	29.69	31.03	29.18	32.43	31.74	28.39	29.73
SORT1	28.9	28.36	30.86	31.45	32.3	31.74	35.18	34.78
LENG8	28.23	25.41	29.88	32.13	30.97	31.75	40.99	37.73
C19orf70	30.37	32.26	27.91	25.92	33.97	31.78	24.27	27.73
TTC1	31.06	32.91	34.3	33.83	38.21	31.79	38.29	33.84
TBC1D17	29.52	30.64	29.43	28.98	29.51	31.82	24.38	28.79
MFSD3	30.17	31.19	30.09	25.93	28.15	31.83	21.26	24.19
SOD2	26.87	22.91	27.45	31.02	30.39	31.85	33.1	30.83
RNF167	34.36	33.76	33.22	33.67	32.17	31.85	27.08	33.43
ADGRE5	25.69	26.35	24.77	24.62	32.24	31.88	29.03	29.99
SPG7	29.22	29.19	30.33	30	30.2	31.89	32.52	31.23
PSMD1	28.02	26.4	29.71	31.26	32.32	31.89	38.21	35.26
ASAH1	27.61	27.68	30.89	35.28	32.67	31.89	46.4	40.52
POLDIP3	29.66	31.61	31.41	30.55	31.7	31.9	27.84	26.15
CRAT	33.03	33.05	30.77	29.74	31.77	31.9	26.01	27.76
S100A13	35.24	36.05	33.81	32.9	33.49	31.92	29.69	31.92
C19orf24	35.02	31.38	32.53	30.15	30.28	31.96	22.68	26.96
EGFR	32.17	30.05	33.51	36.65	32.1	31.96	35.15	34.34
TRIM25	40.98	41.06	39.86	39.35	31.93	31.97	29.9	29.96
AKR1C3	33.89	31.49	32.64	29.95	32.71	31.97	32.75	34.69
TNKS1BP1	37.71	37.12	36.55	36.03	30.71	31.99	25.96	30.1
BAZ1B	34.9	31.09	36.06	35.92	33.74	32	35.93	35.49
UBTF	36.69	37.61	36.13	34.49	32.69	32.03	28.57	29.3
PACSIN2	35.68	36.99	34.62	34.01	33.37	32.03	30	30.35
CC2D1A	32.39	32.61	31.57	28.97	32.3	32.04	26.08	27.58
SMG5	31.56	32.86	31.21	31.08	31.84	32.04	29.96	31.37

LRWD1	32.2	31.58	29.03	28.49	30.47	32.05	22.93	26.33
KREMEN2	35.14	35.33	32.71	31.23	28.12	32.08	23.55	26.62
AKR7A2	30.71	32.33	30.29	27.32	30.45	32.1	25.22	27.23
NAGK	33.33	33.48	30.53	29.12	30.57	32.11	28.2	26.16
GLRX5	30.67	30.41	31.84	28.75	33.39	32.12	29.89	29.07
RHBDF2	29.57	29.09	29.55	27.31	32.11	32.12	27.3	29.18
B3GALT6	29.52	29.48	27.91	24.82	29.24	32.14	23.75	26.86
TXNDC12	33.99	33.38	36.34	36.03	35.35	32.14	38.69	37.51
CLCN7	26.47	26.53	25.69	24.25	30.41	32.2	25.56	27.49
SPTAN1	34.06	33.82	33.46	31.84	30.96	32.21	30.53	29.88
PRRC2C	30.56	30.83	34.4	40.25	33.48	32.22	46.27	44.7
COL17A1	29.28	29.61	30.78	31.45	33.2	32.23	33.85	32.37
SLC26A2	33.74	30.24	38.61	44.22	31.05	32.23	46.08	45.13
UBE2R2	33.21	35.14	32.65	32.25	31.97	32.25	30.11	30.5
TAF9	31.06	30.42	31.25	32.28	38.28	32.25	43.45	41.05
CPNE3	29.51	28.43	32.76	33.09	34.91	32.27	42.56	37.92
GTF2E2	33.58	31.47	30.63	30.85	35.03	32.28	36.1	31.64
MRTO4	29.74	30.97	30.39	27.57	32.65	32.31	29.54	30.74
CIAPIN1	30.03	30.83	31.09	27.87	31.02	32.31	32.58	32.01
ARMC10	33.43	29.94	33.85	33.96	33.24	32.32	37.11	34.45
GJA1	34.69	34.07	41.07	43.89	35.4	32.32	50.22	41.87
HMCES	35.27	33.91	34.85	32.63	32.65	32.33	30.61	29.51
DDX41	34.82	33.67	34.68	31.9	31.78	32.33	27.56	30.64
GOT1	29.7	30.12	29.09	27.18	32.76	32.33	30.16	33.23
SERPINB6	31.84	32.62	32.77	30.6	32.52	32.34	31.89	32.04
CTNNBL1	33.42	32.27	31.68	29.67	30.9	32.36	30.42	30.27
TSPAN17	32.35	33.68	32.8	31.06	31.84	32.38	26.99	29.4
TCEB1	29.17	31.74	32.01	36.15	34.37	32.4	40.71	37.12
MAPKAP1	28.14	28.52	29.22	31.55	32.73	32.41	33.27	30.3
VTI1B	30.43	26.89	29.8	30.97	32.84	32.41	31.41	33.01
SFXN1	31.92	31.09	33.41	32.6	36.68	32.42	33.95	34.02
WDR46	28.84	29.55	29.08	26.56	32.22	32.43	27.71	28.27
SRXN1	22.12	23.01	24.13	23.62	34.51	32.44	31.89	31.87
TCFL5	30.67	28.37	29.7	28.57	28.81	32.46	25.52	27.48
ZNF581	33.64	34.56	34.2	31.55	34.49	32.46	26.06	28.02
CNOT3	33.07	35.41	32.98	30.94	32.22	32.49	26.57	29.12
MRPL9	29.32	32.8	32.17	31.28	35.14	32.5	31.02	29.44
TBC1D10B	34.59	34.44	33.38	30.73	31.52	32.52	26.52	27.36
BICD2	31.17	31.24	31.68	30.92	31.12	32.52	29.38	30.18
CYFIP2	26.27	26.09	26.66	25.25	34.54	32.53	31.46	32.22
PARL	27.47	30.03	28.6	29.96	32.23	32.54	31.78	31.68
TST	28.53	29.37	25.57	23.61	32.23	32.56	22.41	26.76
EML3	32.56	32.26	30.29	31	29.85	32.57	26.27	29.56
ARL6IP5	29.63	29.25	30.93	32.23	33.47	32.57	38.04	36.51
ZDHHC16	30.81	31.32	29.93	29.87	31.95	32.59	29.49	31.75
C11orf58	32.52	31.16	34.1	36.95	34.67	32.59	43.09	40.11
PAK4	34	36.19	33.29	30.86	30.99	32.6	21.55	25.88
ATP13A2	30.88	31.49	29.77	27.22	30.47	32.64	23.27	26.41
TUBGCP2	29.6	30.04	27.45	28.46	32.2	32.65	25.72	26.32
ALDH2	32.23	33.99	29.98	28.71	31.77	32.65	27.85	30.13
ELAC2	29.75	27.78	27.93	25.87	31.65	32.66	28.07	28.28
HMGB2	31.07	30.35	32.55	36.66	33.71	32.67	43.6	36.32
NRP1	32.09	33.84	34.31	36.58	29.84	32.7	33.38	35.18
PRPF31	33.67	31.87	32.07	31.54	33.49	32.72	28.99	29.18
ABR	37.27	37.16	37.3	36.22	32.24	32.72	29.7	32.55
SRSF1	35.05	31.45	36.82	37.77	35.87	32.75	42.94	36.7
C11orf84	34.24	34.44	33.82	31.89	31.53	32.76	25.89	30.29
TMEM129	32.91	33.28	30.43	30.39	31.26	32.77	25.45	29.69
NUDCD2	27.95	28.85	29.93	28.85	31.31	32.77	32.34	31.33
XAB2	35.88	33.28	33.63	29.99	32.58	32.78	28.35	28.04
THAP4	31.36	30.16	29.3	26.53	32.29	32.79	27.92	27.74
NT5E	36.38	34.28	42.5	44.65	34.62	32.79	47.75	42.08
DHX40	30.43	27.1	33.81	35.07	32.36	32.8	45.35	37.83
CMC2	29.04	27.68	28.42	29.04	30.98	32.82	33.11	31.7
FTSJ3	30.98	31.01	32.06	29.37	35.22	32.82	30.8	32.04
CHP1	30.1	30.38	31.3	31.94	31.88	32.83	30.82	32.37
GTF3C5	34.62	32.78	31.4	30.99	33.42	32.87	29.6	30.34
HMGCL	33.53	34.74	33.15	30.48	31.66	32.89	30.05	28.65
MRPS23	29.59	30.78	29.83	30.27	33.6	32.89	30.27	29.95
CKAP5	30.6	27.9	31.66	31.95	33.45	32.89	42.25	39.45
TRAF3IP2	34.87	32.51	35.2	34.51	32.35	32.91	31.68	30.46
RPP25L	28.69	33.59	31.04	29.3	31.76	32.92	26.05	25.58
CORO2A	31.98	34.7	33.64	33.52	31.57	32.92	31.36	31.39
ZNF622	36.18	36.77	34.04	35.48	32	32.97	26.5	30.38
EXOC4	31.52	32.13	31.67	31.3	33.32	32.99	36.26	32.28
DDX1	32.61	32.32	37.19	37.68	35.21	33	44.94	41.1
B4GALT3	28.09	31.27	31.95	27.27	33.37	33.01	28.14	27.8
NUP85	32.1	32.1	31.39	31.38	31.93	33.02	34.22	30.29
PCYOX1L	31.71	32.83	32.13	30.93	35.18	33.06	32.51	32.75
IL1RAP	31.92	29.94	36.09	42.19	35.65	33.06	49.97	45.37
BRAT1	33.6	31.89	32.13	28.66	30.68	33.1	25.59	27.71
MRPS27	31.72	32.47	34.21	32.31	35.49	33.11	36.8	36.25
GNL3	28.04	26.79	30.61	34.09	34.09	33.13	45.98	41.39
CCDC86	28.87	26.56	27.65	26.07	33.94	33.16	26.91	26.98
GTF3C1	34.69	35.29	32.81	31.67	32.8	33.16	29.23	32.05
SH3BP1	35.43	35.77	33.74	31.12	32.34	33.17	25.63	30.38
SRPRA	31.62	31.97	33.54	31.23	35.97	33.2	30.39	33.95
FAM168B	30.52	29.5	31.17	32.49	34.67	33.2	34.94	35.15

HMGCR	28.17	29.03	30.36	32.2	31.94	33.21	40.52	39.24
GET4	29.93	28.06	29.94	27.98	29.28	33.22	27.01	28.74
GAS2L1	25.55	26.88	23.79	21.75	30.25	33.24	22.45	26.28
SERPINA1	25.84	27.97	25.96	25.57	33.06	33.25	30.53	33.31
IFNGR2	38.39	37.93	39.92	40.2	36.51	33.27	36.47	35.04
ZMAT2	29.27	30.95	32.98	32.66	35.23	33.29	38.19	38.01
CMPK1	28.26	28	31.38	35.09	35.1	33.31	46.49	41.63
WDR13	33.27	33.74	29.49	29.45	33.48	33.32	27.75	29.2
SLC39A3	27.59	29.23	25.37	26.37	29.48	33.35	21.12	25.42
C11orf31	38.35	37.15	38.91	36.83	36.34	33.35	28.23	28.55
RNF114	35.29	36.16	34.23	35.37	33.91	33.35	34.39	32.81
CDCA7L	35.63	34.25	35.74	35.51	36.54	33.35	38.56	33.13
RANBP3	34.3	34.59	32.5	32.43	32.71	33.36	27.52	29.46
WRAP73	27.38	24.45	25.42	22.95	29.06	33.38	28.35	26.86
MELTF	32.98	35.37	32.71	31.16	31.39	33.39	27.3	30.11
PKIG	37.63	38.98	37.38	34.46	33.47	33.39	26.27	31.28
SPHK1	31.25	29.17	29.05	27.66	31.48	33.41	23.05	28.48
PNPO	31.67	35.05	33.5	31.76	33.59	33.41	34.08	34.79
MGME1	30.03	27.04	28.68	31.5	34.41	33.41	41.22	35.61
FLAD1	30.07	30.43	29.82	27.36	30.83	33.43	27.67	28.78
SMPD4	31.99	32.28	31.55	29.67	31.45	33.45	30.26	30.27
AKIRIN1	31.7	33.46	33.11	34.64	33.88	33.45	36.25	35.47
SON	32.78	30.65	34.66	35.99	34	33.45	43.5	39.31
ZDHHC24	26.53	25.02	25.35	22.48	25.13	33.47	19.07	21.84
IDH2	35.39	35.65	32.77	30.87	33.49	33.47	26.45	26.92
TMEM203	30.09	29.78	29.35	28.96	30.02	33.48	26.84	28.23
WBP11	31.69	32.82	32.37	32.03	35.39	33.48	35.22	32.89
MYBL2	40.56	37.2	38.78	35.79	33.17	33.5	26.69	24.87
C5orf15	34.53	32.42	35.64	37.47	36.14	33.5	45.51	42.91
NDRG3	32.12	33.71	32.83	32.93	37.03	33.53	35.32	35.1
SH3TC1	41.04	40.77	36.84	35.41	31.2	33.54	26.11	27.43
TFAP2A	35.4	37.03	36.68	38.28	31.71	33.54	32.03	34.85
FZD5	32.86	30.22	33.68	34.91	34.28	33.55	33.83	34.5
PACSIN3	36.6	37.75	34.51	31.6	31.69	33.56	26.84	30.94
CFAP20	33.52	36.63	35.34	32.36	36.73	33.56	34.79	31
ATP6V0E2	29.06	30.25	28.36	28.09	30.72	33.59	25.38	26.21
EDEM2	33	36	33.5	33.18	35.25	33.61	30.01	32.88
FAM207A	32.84	35.32	28.58	29.34	29.2	33.63	22.96	25.35
IGFBP6	49	45.07	42.05	39.65	29.28	33.65	24.44	24.25
ICMT	31.48	31.48	30.32	29.79	35.8	33.65	33.82	32.49
MARVELD1	40.51	39.67	39.38	37.48	29.71	33.67	27.02	29.69
MED25	35.27	37.79	35.88	32.58	33.73	33.67	27	30.19
44446	35.97	35.88	35.75	38.04	34.43	33.68	52.39	46.86
ADORA2B	30.06	28.71	30.15	27.29	34.49	33.69	34.78	31.86
UBL7	33.72	35.24	30.42	32.25	32.13	33.7	27.34	28.53
RRNAD1	31.94	32.44	32.39	31.11	31.52	33.72	28.39	28.09
HYAL2	33.91	35.22	33	30.85	30.12	33.73	24.59	28.18
AIP	35.09	33.8	34.64	29.78	34.05	33.76	28.8	27.63
PSPH	45.06	44.11	43.87	42.44	33.6	33.77	35.35	35.69
FOXK2	30.64	32.1	31.31	31.16	31.72	33.79	31.01	31.26
DNAJA2	32.79	32.49	32.45	32.05	35.99	33.82	36.76	34.25
PRKAG1	30.2	29.59	28.92	27.63	35.45	33.82	36	35.82
MANEAL	34.24	33.62	32.77	33.12	33.2	33.83	27	28.59
CKLF	34.43	36.15	34.96	37	35.17	33.86	37.01	34.3
WIZ	32.54	34.16	32.65	31.47	32.58	33.88	28.32	30.11
PTK2	33.81	31.8	35.08	37.49	34.41	33.9	41.73	41.58
THOC7	34.46	37.39	38.5	41.68	35.22	33.91	43.24	37.53
KIF22	34.75	30.74	29.65	26.88	32.86	33.92	28.15	27.88
CPSF1	30.66	30.37	28.77	27.24	31.56	33.93	26.2	26.89
ARL4C	35.05	34.44	33.87	32.39	33.28	33.93	32.14	33.56
DNTTIP1	33.7	36.26	35.81	32.32	36.36	33.94	32.19	31.57
STT3A	36.35	38	37.32	38.32	35.1	33.94	37.41	34.91
NOP16	27.42	25.31	25.79	25.64	35.38	34.03	29.48	32.6
RGL2	31.87	31.92	29.49	30.45	32.2	34.09	30.16	33.37
TMEM222	31.59	32.33	32.9	29.32	31.42	34.12	28.89	28.53
DHX30	33.41	33.55	31.69	30.49	32.19	34.13	28.84	29.75
ARAF	30.89	31.39	28.41	31.87	32.67	34.16	28.49	30.14
AMFR	32.43	33.39	31.11	31.22	31.2	34.16	28.58	31.4
PIH1D1	33.89	33.41	31.99	29.1	29.79	34.17	25.83	26.61
DDA1	31.29	32.51	30.01	30.03	33.75	34.19	30.47	30.92
MGP	26.75	27.63	29.01	28.78	33.83	34.19	38.03	34.37
CHST14	35.55	37.45	35.66	32.75	32.27	34.24	25.08	29.58
JMJD8	35.12	36.64	35.21	33.44	33.67	34.26	29.43	29.62
ETV5	31.57	34.02	31.74	31.84	33.37	34.26	32.04	35.65
HACD3	32.24	30.17	31.61	33.85	34.8	34.26	39.05	36.32
TPRG1L	31.32	33.07	34.24	28.41	34.69	34.29	31.11	33.68
CMBL	31.3	31.99	33.04	31.17	34.31	34.33	35.17	32.7
ITGA2	32.96	28.39	39.43	43.91	36.83	34.33	60.23	48.46
DRG2	30.49	31.4	30.43	28.64	33.87	34.34	29.85	30.11
WLS	36.1	37.38	34.03	34.89	34.39	34.34	35.85	34.26
FDX1L	30.2	30	30	29.11	32.74	34.4	27.95	32.74
KRI1	32.29	32.83	33.92	34.58	35.34	34.41	35.39	35.98
SLC39A10	24.33	23.94	28.55	33.61	33.79	34.45	55.54	47.92
IGBP1	35.52	31.56	33.43	32.32	36.48	34.46	35.72	34
PKD1	35.51	33.99	34.51	35.44	30.85	34.48	32.02	32.9
ZC3H18	35.3	34.31	35.72	35.04	34.99	34.51	28.83	30.83
C8orf59	31.53	36.2	33.73	37.01	37.45	34.52	48.42	44.61
CCNB1IP1	30.2	31.6	29.46	29.14	38.7	34.53	38.37	38.73

CYHR1	33.43	36.42	32.33	31.55	33.89	34.55	29.85	29.23
ANKRD13D	30.43	32.17	30.73	30.22	34.22	34.59	27.18	31.51
RIN1	32.02	31.39	28.26	26.82	31.44	34.67	24.31	26.6
IGSF8	35.35	37.25	33.72	32.13	33.49	34.67	24.08	27.88
FOXD1	34.2	32.89	35.34	31.98	29.54	34.68	26.45	27.48
FAM134A	31.28	32.5	30.14	30.4	33.51	34.68	29.82	30.81
TPCN1	23.08	23.55	23.17	24.88	34.89	34.69	32.44	33.14
PSMD6	30.28	26.79	32.27	33.66	34.52	34.74	43.35	39.32
SAP18	33.17	32.65	31.34	33	35.81	34.75	35.45	33.38
PIAS3	38.2	38.75	38.11	37.74	33.23	34.75	33.43	36.39
ADH5	33.2	34.14	35.04	33.3	36.61	34.77	39.14	35.07
ATP6V1H	29.03	29.08	29.18	28.85	36.17	34.78	39.15	35.75
SLC39A13	30.51	31.47	31.53	29.11	32.87	34.8	28.57	30.66
DNPEP	38.77	37.88	35.77	35.43	35.21	34.81	30.89	32.85
BNIP3	53.26	48.79	54.18	51.04	34.42	34.81	39.59	35.23
ZCRB1	34.96	34.44	33.82	39.67	38.13	34.83	42.9	41.28
SERPINF1	40.85	45.89	38.01	42.44	29.46	34.84	29.85	34.4
STIM1	35.82	36.21	34.7	34.01	32.51	34.86	32.3	33.26
PPP1R11	37.81	39.22	35.23	37.62	35.06	34.91	32.91	34.61
EXOSC10	35.16	34.52	34.74	34.36	36.17	34.91	38.46	35.97
POLR2C	34.4	35.06	32.28	31.96	36.49	34.93	32.18	33.71
MRPL49	32.59	35.11	33.6	30.07	36.9	34.96	31.74	32.31
B4GALT5	33.47	34.64	34.94	35.75	36	34.97	36.44	36.51
SPRY2	40.25	40.43	39.93	43.21	35.47	34.99	36.81	35.24
EIF2A	30.25	26.62	32.62	36.43	37.09	35	50.47	44.88
CYFIP1	34.5	33.94	33.61	34.3	33.66	35.01	34.27	33.7
TFDP1	31.63	31.88	32.9	34.15	35.38	35.01	36.35	34.79
YTHDF1	34.58	32.51	32.41	33.11	34.7	35.08	33.9	33.48
SLC7A8	30.55	32.87	29.17	31.63	33.55	35.08	29.21	35.24
ARL8A	36.96	37.92	34.4	35.4	37.2	35.09	31.49	32.5
SLC29A1	32.02	30.61	30.27	28.62	36.5	35.11	30.63	32.55
DNAJC4	39.87	41.49	37.05	38.6	37.81	35.12	29.6	32.72
LRP1	36.57	38.81	35.72	37.2	30.04	35.12	29.43	34.27
PPP2CB	34	34.06	34.09	36.22	36.84	35.12	42.81	37.96
SOX4	41.01	40.25	43.2	44.78	36.23	35.13	34.45	37.92
ACBD6	32.96	31.05	30.32	29.89	33.7	35.16	30.41	31.5
NSG1	33.93	34.83	33.83	32.15	32.56	35.16	31.3	32.28
ATP2C1	37.73	33.53	38.46	39.64	35.96	35.16	45.02	41.03
FOXP1	36.51	35.18	40.09	40.87	34.61	35.16	43.25	42.94
PSMD14	31.4	28.31	32.28	33.91	35.73	35.16	42.62	45.34
GALNS	34.46	35.85	33.86	32.6	32.49	35.18	29.36	33.65
SRSF4	36.04	38.33	35.85	38	36.25	35.18	34.83	38.04
CCNB1	32.06	31.26	32.01	27.6	41.22	35.21	39.27	39.19
UPK3BL	25.99	28.42	31.3	33.71	29.21	35.22	34.08	31.01
SMARCC2	39.49	39.54	38.46	36.9	34.71	35.24	34.75	35.62
COMTD1	31.05	31.23	28.69	26.28	36.03	35.27	26.78	27.42
LRRC47	37.72	36.12	34.64	33.52	33.97	35.27	29.78	33.41
IST1	36.37	39.13	37.86	36.63	37.6	35.27	37.22	37.27
LSM14B	37.99	37.33	36.93	37.22	34.97	35.36	31.22	32.39
GDE1	35.23	37.59	35.84	36.17	36.9	35.36	37.29	36.14
SLC39A4	35.6	36.61	37.1	32.51	38.09	35.5	27.18	31.71
PCYOX1	31.25	29.57	34.78	36.52	35.26	35.52	44.91	40.32
PEF1	34.75	34.67	32.03	30.02	34.61	35.55	28.31	28.55
MPZL1	36.15	37.27	35.41	38.22	36.51	35.57	37.83	37.06
FBRS	35.56	35.93	35.78	34.56	33.78	35.6	28.02	31.67
CNPPD1	33.85	35.24	31.13	31.35	38.13	35.62	28.42	30.99
ESYT2	32.43	29.69	33.42	34.16	36.37	35.63	40.98	37.47
POLE3	34.54	33.8	33.52	32.83	38.66	35.64	36.82	36.63
MAP1B	28.61	26.7	33.31	39.22	35.24	35.65	46.37	47.9
CHPF2	39.71	39.46	36.78	35.79	33.41	35.66	29.08	31.22
GNPDA1	31.56	32.46	33.4	32.53	39.85	35.66	39.04	38.37
SDHC	36.91	38.88	37.61	35.15	39.92	35.68	38.89	38.88
SSSCA1	36.99	38.28	37.7	30.1	36.54	35.73	28.84	31.27
DDX56	31.94	31.49	31.37	29.47	34.43	35.78	32.32	31.98
GNS	31.76	34.47	33.58	32.51	34.36	35.78	39.1	37.6
STT3B	32.68	30.82	34.69	38.11	35.45	35.8	48.01	43.66
AP1B1	34.19	34.74	33.06	29.88	35.35	35.81	29.16	31.12
DGKA	31.33	34.87	33.31	34.9	33.37	35.81	34.93	35.27
PEA15	37.55	38.69	34.42	36.19	38.63	35.82	34.01	37.41
KLF16	34.41	35.74	33.26	30.81	34.69	35.83	25.25	28.52
ABCA2	37.4	38.41	36.43	35.35	32.47	35.83	29.08	33.09
MIF4GD	36.06	37.78	35.87	32.46	34.8	35.84	32.14	32.54
MEX3D	37.81	34.54	35.18	32.7	31.09	35.88	24.02	25.97
CDKN2AIPNL	36.58	38.86	37.44	37.32	37.88	35.89	29.58	32.21
TMEM43	32.73	33.66	33.33	33.14	36.52	35.89	35.9	35.85
MSMO1	32.1	31.32	34.46	39.19	36.36	35.89	51.29	45.54
HARS	35.62	34.86	32.93	34.27	37.4	35.92	36.76	34.83
TERF2IP	36.63	37.29	38.88	37.97	39.77	35.92	37.2	36.79
ZNF282	36.38	36.67	34.92	32.42	35.47	35.93	27.67	28.99
PSENEN	40.48	39.16	35.84	34.7	37.48	35.94	31.14	29.21
MOB3A	42.67	46.95	43.83	41.3	33.92	35.94	30.94	35.29
CYB561A3	35.22	32.65	32.39	31.08	37.34	35.95	30.85	31.17
VGLL2	28.77	29.38	29.84	30.15	33.09	35.96	28.84	29.86
BRMS1	35.81	39.63	36.18	30.99	37.77	35.96	30.4	31.77
PAM	36.04	35.19	37.74	38.19	35.62	35.97	42.86	39.57
ACTL6A	40.79	39.13	43.18	41.62	40.67	36	42.41	40.27

PIDD1	31.59	28.15	30.2	29.3	37.72	36.05	36.76	34.7
PEX10	34.61	34.64	31.72	30.04	34.56	36.07	26.48	29.27
HEBP2	33.5	32.91	33.67	32.89	35.36	36.07	32.97	29.31
INO80B	33.85	31.46	30.29	28.39	32.86	36.07	26.95	29.68
CYSTM1	36.04	35.27	34.24	34.15	36.02	36.08	29.16	32.9
TOP1MT	33.47	35.34	32.49	32.73	36.86	36.11	32.41	36.24
SUPT16H	35.84	30.87	37.83	40.96	39.21	36.12	47.32	43.38
CLPP	33.01	30.18	32.17	27.22	36.09	36.15	23.71	27.07
B4GALNT4	41.68	42.03	38.52	39.54	34.3	36.17	29.95	34.07
NCAPH2	38.38	37.18	35.08	31.46	34.31	36.19	26.79	28.03
DVL3	36.41	34.74	34.39	35.3	36.92	36.2	34.26	36.7
CDC42SE1	34.01	36.25	36.58	35.37	38.33	36.25	39.01	39.57
CNOT1	35.5	32.96	37.71	37.92	35.99	36.25	44.82	40.38
GAGE12B	37.78	43.43	46.64	38.89	49.29	36.33	37.65	46.89
MRPL14	30.29	33.21	30.38	27.86	33.29	36.35	28.26	29.97
COL4A1	37.29	38.01	36.92	39.54	34.89	36.39	36.21	36.72
AGR2	36.48	37.06	35.64	40.91	33.28	36.41	41.81	41.65
RBM3	38.89	33.77	39.19	36.63	40.92	36.42	39.8	34.26
RRP9	34.18	34.5	33.91	30.31	40.11	36.43	28.8	33.51
USP10	36.62	37.57	38.06	37.89	36.32	36.43	40	37.93
CCZ1	34.75	32.57	31.06	38.05	36.75	36.47	42.19	37.73
CARS	41.66	41.82	40.35	39.48	43.47	36.49	38.91	41.1
HN1L	36.76	36.32	35.15	34.05	36.75	36.5	37.06	35.78
KNOP1	39.4	40.76	37.38	37.43	37.99	36.54	34.27	36.95
TOR4A	39.85	40.94	39.13	35.22	34.98	36.56	25.95	28.4
TOB1	30.06	30.36	33.45	33.82	38.48	36.56	43.57	43.58
ICAM4	42.78	44.77	39.43	35.27	32.59	36.58	25.95	31.22
SNRNP40	37.36	36.89	36.83	35.57	38.05	36.59	32.84	32.6
ZDHHC8	33.14	32.98	31.5	29.28	31.39	36.65	28.17	30.21
PCCB	31.72	31.21	29.93	30.3	37.51	36.65	33.2	35.3
LRFN4	32.13	31.08	29.25	28.02	33.56	36.66	26.82	28.66
SLITRK6	40.85	38.8	47.66	57.68	35.7	36.66	63.73	57.05
ERRFI1	39.26	38.05	40.94	43.45	36.9	36.67	43.9	39.17
ACP1	32.6	30.91	33.3	33.56	38.09	36.74	45.91	41.48
GCN1	35.16	34.97	33.38	33.83	35.69	36.77	32.65	35.11
GGT7	33.45	36.56	33.51	36.46	34.57	36.84	34.14	36.94
SLC41A3	30.18	29.38	29.81	28.5	35.53	36.89	32.93	33.51
C5orf38	42.24	39.62	42.09	37.53	36.68	36.9	25.79	29.1
HAS3	35.77	34.16	35.73	36.03	37.48	36.9	36.13	33.89
PSIP1	38.59	34.85	40.91	43.07	38.78	36.91	51.38	47.44
EVA1B	37.01	39.65	34.81	32.31	33.45	36.92	25.11	29.6
ARHGAP1	41.47	42.12	38.63	37.8	35.16	36.97	31.6	34.53
PANK2	39.2	37.42	40.38	35.17	33.72	36.98	37.13	38.9
GNA15	33.85	33.55	34.14	29.46	36.13	36.99	29.04	31.08
POLR2H	31.45	30.81	32.54	30.55	35.11	37	34	34.08
FAT1	34.42	34.55	37.06	41.02	36.99	37.01	46.68	43.25
TPBG	36.58	35.63	36.13	39.07	35.71	37.02	37.15	35.85
PMPCA	33.13	34.87	33.67	30.44	37.36	37.06	32.23	32.81
AKAP1	33.19	32.45	32.81	33.13	37.24	37.08	37.26	36.35
DEGS1	35.48	36.9	35.34	37.18	38.06	37.1	41.65	37.91
METTL23	34.16	35.23	36.22	34.57	35.7	37.16	35.87	33.09
BRD4	39.63	39.76	38.28	40.12	35.71	37.17	32.52	35.87
MRPL40	36.77	38.6	39.46	39.09	36.84	37.17	37.42	36.72
MYO9B	34.9	35.1	34.56	34.35	35.09	37.2	31.28	33.3
TRADD	39.86	38.15	33.43	34.26	32.21	37.21	26.33	29.58
LAP3	41.97	39.66	43.25	42.47	39.46	37.21	42.39	36.25
MRPS2	35.05	33.33	32.62	29.57	36.07	37.22	28.78	30.67
DYNLT1	38.37	40.01	38.16	42.19	42.76	37.23	38.4	35.84
NANS	41.3	42.52	36.38	36.18	38.41	37.25	33.16	33.07
GORASP2	37.79	41.82	39.75	39.93	39.62	37.26	38.32	38.97
SLC25A11	35.63	36.22	34.24	31.68	36.14	37.27	28.28	29.77
DCAF15	35.87	37.2	33.48	32.47	33.05	37.27	27.68	29.88
CHST2	45.34	41.95	43.47	39.9	33.66	37.28	29.07	29.45
RETSAT	28.25	29.1	28.18	28.79	37.73	37.29	33.91	36.51
H2AFV	46.68	47.23	45.7	46.62	39.55	37.3	38.99	38.01
ZDHHC4	38.27	38.33	36.61	36.68	39.82	37.31	36.44	35.93
DPYSL2	35.99	38.23	38.15	40.09	35.92	37.34	37.47	40.66
BECN1	36.61	33.12	37.42	39.27	42.65	37.34	45.18	42.37
E2F4	36.68	32.92	35.1	32.87	37.48	37.36	34.89	34.9
CD2BP2	39.36	39.97	39.98	37.88	39.12	37.36	34.29	37.24
EXT2	38.77	41.3	39.27	38.72	37.73	37.41	36.99	39.46
PRR7	39.36	37.27	34.39	31.36	32.33	37.42	22.35	26.36
FBXO44	33.01	35.95	34.72	33.48	40.95	37.47	34.89	37.89
SMARCD1	35.56	40.59	39.99	37.72	35.24	37.48	35.19	35.03
TMUB2	32.47	34.49	31.93	32.92	36.78	37.48	34.6	35.93
RSL1D1	32.22	30.92	35.01	37.28	39.28	37.48	47.51	42.66
BRD2	41.58	41.78	40.97	43.64	39.64	37.51	39.38	41.64
ATP8B2	36	34.85	35.46	36.26	36.47	37.54	34.47	37.45
DPP3	37.26	38.76	35.97	34.02	36.92	37.55	27.78	30.93
SLC35B1	34.13	35.51	35.12	35.39	39.03	37.55	35.99	37.32
SERP1	38.65	38.2	39.87	38.44	38.34	37.55	39.78	40.97
TMED10	36.68	35.11	39.59	43.15	37.56	37.55	47.1	42.47
ANXA6	46.57	51.82	46.99	44.75	39.38	37.59	36.05	34.84
MID1IP1	29.68	31.7	27.92	31.5	34.89	37.6	29.27	35.24
ATAD3A	32.36	31.65	30.54	28.18	37.07	37.63	28.49	30.93
PSMA3	37.17	35.98	36.72	41.09	38.88	37.64	49.27	39.81
CLIC4	34.24	30.17	38.12	42.83	38.99	37.64	55.15	46.87

PTPRA	39.07	36.59	37.86	39.31	38.66	37.67	39.98	38.22
ACSS2	31.33	35.47	30.33	32.45	36.19	37.67	35.55	39.34
STK17A	37.18	34.66	41.55	43.32	43.55	37.68	58.03	48.29
UBE2J2	35.29	35.52	32.61	32.06	37.87	37.73	30.53	32.64
CRELD2	35.67	36.64	33.15	31.56	34.13	37.75	29.45	30.44
SLC38A10	38.16	39.14	37.55	36.54	33.86	37.75	30.86	33.85
TMEM14B	38.43	40.38	35.09	36.04	40.03	37.75	35.84	34.2
BAIAP2L1	38.39	37.91	39.28	38.04	38.92	37.76	39.94	39.06
KEAP1	39.52	39.28	36.28	34.72	35.61	37.85	30.43	31.15
DPM3	44.48	40.25	41.49	35.57	42.69	37.88	22.99	27
ZNF580	44.73	45.24	40.7	39.87	33.62	37.91	27.86	29.26
SRPX	37.62	36.52	38.56	37.05	35.92	37.93	30.4	34.05
PLEKHB2	37.85	36.99	37.93	37.39	39.23	37.99	42.87	41.04
TEX264	38.29	37.96	38.88	35.63	37.97	38	29.48	32.67
TRAPPC3	38.37	37.63	37.3	37.76	40.21	38.02	36.29	36.55
STXBP2	41.12	41.49	37.81	36.87	37.59	38.03	32.13	37.76
PTK7	40.42	42.02	39.64	38.49	37.44	38.05	32.15	35.33
SF3B3	37.91	40.56	38.59	37.63	39.24	38.05	37.71	37.07
SCARA3	44.45	46.34	45.54	43.04	37.05	38.06	32.09	35.48
TCIRG1	29.44	30.15	29.9	31.1	36.41	38.09	35.37	38.17
WDR74	35.71	36.23	36.19	32.05	39.06	38.15	30.23	35.78
TCOF1	40.59	39	37.43	36.91	41.44	38.15	34.8	37.22
MED24	38.7	36.15	36.09	35.79	38.38	38.18	34.38	36.01
THRAP3	36.86	34.75	38.73	41.01	39.37	38.19	44.21	43.51
COQ9	36.54	35.51	33.72	33.62	37.39	38.2	35.07	37.15
DYNLL2	44.61	45.54	44.34	47.25	40.33	38.2	35.16	39.16
CHMP5	37.34	39.36	38.8	41.6	41.94	38.21	50.05	45.38
STYXL1	44.44	45.25	44.97	42.27	38.46	38.22	39.2	38.22
TNFAIP2	45.92	43.8	44.78	43.17	38.48	38.23	38.38	38.09
RCC1L	36.05	37.51	34.99	34.45	38.44	38.26	29.57	33.83
GALNT18	38.67	38.89	38.03	36.17	36.92	38.28	34.89	35.9
HSPB11	43.14	41.42	40.98	41.38	34.11	38.28	44.84	41.8
ATG9A	34.95	36.66	35	33.58	35.48	38.29	33.44	34.56
MRPL52	37.3	38.18	36.26	32.32	36.54	38.3	31.61	32.38
DTYMK	36.36	35.91	35.2	32.81	38.32	38.3	30.29	33.8
ACSL4	33.73	34.43	38.72	44.64	37.52	38.34	55.13	52.59
EID1	35.86	33.69	40	42.29	42.06	38.38	52.56	44.32
POLRMT	35.14	32.86	32.2	30.87	35.74	38.41	27.2	29.67
RRP36	35.65	38.05	36.91	37.63	41	38.41	38.26	38.86
ANXA8	34.59	36.45	36.94	30.4	45.35	38.41	39.69	40.8
SLC9A3R2	38.87	37.85	35.3	33.95	37.11	38.42	30.06	32.14
EXOC3	36.36	35.89	36.47	34.16	37.18	38.42	36.8	37.91
CNPY3	39.46	41.06	39.29	35.65	36.42	38.45	30.38	33.06
PLXNB1	40.52	39.73	39.03	38.33	34.78	38.46	34.36	37.12
UBE2D3	36.45	36.86	41.99	39.54	41.98	38.46	46.52	43.83
HUWE1	37.64	37.5	38.08	39.72	37.41	38.47	39.87	41.35
NDUFS4	43.93	46.63	44.34	43.5	43.98	38.47	57.29	50.95
CNDP2	40.62	41.11	38.12	40.19	40.01	38.48	37.49	36.82
SUCLG1	32.27	32.14	30.6	29.63	37.5	38.52	37.4	35.8
LPCAT3	36.3	39.48	35.16	36.74	37.35	38.52	37.04	39.4
DAB2IP	38.91	40.21	37.48	37.23	37.21	38.53	32.56	34.05
CDIP1	32.19	33.92	31.67	29.16	39.41	38.55	32.39	33.56
CLIP2	42.19	45.25	41.23	37.96	36.27	38.55	31.13	34.12
MAT2B	36.14	35.17	40.93	39.85	42.36	38.55	53.3	45.91
BCL7B	36.18	38.2	35.35	35.98	36.95	38.56	35.81	36.15
MPV17	33.76	34.95	32.74	33.13	38.51	38.6	35.42	35.85
AZIN1	36.07	35	37.88	40.5	39.44	38.6	47.7	44.96
CLN3	33.7	36.33	34.72	30.21	37.79	38.63	33.18	31.55
PACS1	40.24	40.98	37.95	40.55	38.25	38.64	33.78	38.17
CUX1	41.63	42.02	41.24	39.22	37.38	38.67	32.78	36.46
USB1	34.56	37.64	34.74	36.04	38.52	38.67	35.57	41.03
NPTN	37.76	36.19	39.84	42.35	39.98	38.7	46.1	45.8
XPO6	36.15	36.22	36.52	36.93	38.43	38.71	38.25	38.08
TOMM22	37.65	38.96	34.61	33.63	41.88	38.71	35.39	39.22
NAT9	30.64	31.25	30.45	29.15	37.86	38.74	36.66	34.05
TXN2	37.64	39.67	34.36	36.14	40.49	38.74	32.72	35.16
VPS72	43.51	45.71	42.47	43.33	42.07	38.74	37.3	35.65
PCMT1	34.93	37.35	38.28	36.56	39.68	38.77	45.73	41.45
RGS3	37.4	40.32	36.01	34.1	39.04	38.79	32.91	33.13
PPP1CC	37.21	33.95	39.34	39.84	39.91	38.83	50.03	43.21
IPO7	30.59	26.92	35.52	39.51	41.43	38.83	60.04	50.92
CDV3	34.85	33.58	36.63	42.06	40.82	38.84	47.65	43.9
LGMN	39.49	41.24	40.37	41.52	38.4	38.87	38.67	39.23
TMEM50A	43.56	43.27	44.56	48.26	43.22	38.91	45.76	43.46
NAT14	41.47	43.83	40.58	37.18	37.87	38.93	27.49	33.51
FUCA1	42.61	42.36	43.97	42.93	39.65	38.93	39.34	39.39
MRPL10	36.38	40.4	36.48	35.81	38.76	38.94	34.23	35.72
SRSF11	35.78	35.31	40.38	47.93	40.2	38.95	66.36	57.33
BCKDHA	42.32	41.44	39.5	36.25	39.36	38.96	30.06	34.11
CSK	36.55	35.68	35.69	35.55	37.96	38.97	30.71	35.5
DHX15	37.28	34.04	39.59	44.34	40.32	38.99	54.56	49.46
SERPINB5	27.87	25.98	30.19	31.51	40.88	39	47.36	43.02
RBX1	40.29	40.23	41.34	37.48	37.63	39.05	41.11	36.04
AP3S1	37.97	39.24	37.59	39.04	41.94	39.05	52.73	46.64
NOL7	32.74	35.4	40.42	38.61	40.63	39.09	41.49	37.59
KLHL21	38.96	39.28	36.56	34.78	38.75	39.1	31.61	34.64
ARHGDIB	32.85	33.05	34.84	36.01	41.02	39.1	37.03	37.14
GALNT10	37.46	39.73	39.18	40.4	41.01	39.1	41.24	39.73

UBA2	33.62	33.03	37.66	40.25	41.66	39.1	50.65	44.02
MTRNR2L1	35.15	37.01	38.74	41.32	37.18	39.14	41.33	44.46
NPPC	32.55	33.27	27.98	26.88	26.46	39.18	20.78	32.16
WDR18	40.87	38.44	36.22	31.65	35.66	39.21	24.86	28.26
P4HA2	44.56	48.3	42.25	41.31	38.33	39.21	38.07	38.64
DLST	39.72	40.78	40.52	38.4	40.82	39.21	40.14	41.24
NISCH	39.69	39.47	39.76	40.47	39.04	39.22	42.39	40.84
ZFPL1	45.15	44.67	42.49	39.8	38.78	39.24	33.55	34.06
PTP4A1	33.76	35.09	36.56	39.45	41.06	39.24	46.72	46.06
NUP62	40.98	41.02	40.89	40.58	38.75	39.25	33.08	36.01
BSCL2	41.6	39.49	41.05	39.8	36.68	39.26	32.41	34.81
YTHDF2	40.3	37.8	42.03	41.1	40.13	39.27	45.08	40.15
CNIH4	39.03	41.26	38.33	39.03	39.8	39.27	42.37	42.85
CARD10	40.05	41.28	39.24	37.2	38.53	39.28	33.36	32.73
TBCD	37.69	38.75	36.63	35.04	38.81	39.31	33.98	36.14
ACY1	32.07	34.41	32.73	27.88	36.5	39.32	27.41	30.06
FURIN	41.44	41.83	39.88	38.25	36.66	39.33	29.13	33.6
RPP21	43.4	43.59	39.65	36.39	37.67	39.34	30.12	31.88
MAP1S	42.03	44.32	40.33	36.88	37.59	39.4	27.03	31.58
STRN4	41.52	40.56	39.26	37.32	38.97	39.4	33.49	34.92
NDUFA12	39.28	43.46	37.05	42.03	40.63	39.4	43.15	43.44
RUVBL2	39.58	40.51	36.95	33.04	39.6	39.42	29.65	31.6
SCYL1	39.08	37.7	37.15	35.5	38.52	39.42	31.61	33.16
NCOA4	37.04	33.52	37.42	37.73	40.89	39.42	43.48	40.48
SUPT4H1	39.15	42.86	40.95	39.01	42.86	39.43	39.99	39.16
AATF	40.94	41.54	44.64	40.27	45.31	39.46	41.12	39.77
FUBP1	44.1	41.37	44.09	45.33	41.67	39.47	48.14	43.29
CTDSP1	45.15	46.11	43.28	45.62	39.86	39.48	32.77	36.1
FAM83G	36.85	36.78	35.47	33.17	37.18	39.49	29.02	32.18
SART1	41.86	41.45	40.06	37.36	40.53	39.52	31.34	33.14
SLC35A4	40.78	40.02	39.84	38.4	39.95	39.52	35.48	37.66
NUDT1	36.75	37.25	35.6	33.36	40.07	39.56	31.09	30.15
UNC93B1	43.42	41.93	41.08	38.49	37.62	39.56	26	32.51
RFC2	44.01	43.32	42.27	37.42	39.56	39.57	39.83	33.91
B4GALT7	39.11	38.67	37.45	35.66	37.26	39.57	29.28	33.95
GLUD1	35.76	34.57	35.48	33.46	40.42	39.57	39.04	37.4
LARS	33.55	32.13	36	36.64	41.79	39.69	51.06	44.89
CLTB	44	44.38	40.32	36.88	39.57	39.73	28.47	32.86
PSMB2	37.92	38.56	37.78	37.94	41.24	39.73	38.69	38.72
YWHAH	43.59	43.1	40.93	41.56	42.4	39.77	38.31	40.85
GSDMD	44.46	44	40.23	39.73	37	39.81	29.39	32.5
RNF40	41.41	42.76	40.75	38.25	39.31	39.81	33.58	35.8
LAGE3	41.61	40.51	37.66	33.81	38.6	39.83	29.16	29.1
RAP1B	40.37	39.03	44.05	45.98	44.23	39.87	58.41	51.89
POLD4	36.77	39.43	37.93	35.02	36.44	39.9	32.62	34.32
MTCH2	36.18	37.6	36.83	37.06	43.01	39.9	41.81	43.02
ZDHHC5	36.27	38.6	36.19	35.43	38.79	39.92	39.53	39.34
CHCHD3	31.2	31.7	34.73	35.94	41.38	39.93	42.36	42.72
PRMT2	41.43	40.9	40.7	41.73	38.44	39.96	37.59	39.1
SLC22A18	46.1	45.67	40.22	38.87	37.17	40	27.48	34.41
LMAN1	35.64	34.16	42.75	49.72	40.64	40.03	69.26	59.48
CKS2	32.04	34.74	29.41	28.56	38.11	40.07	45.25	43.2
POLR2I	39.08	42.25	34.69	36.65	37.13	40.1	30.02	28.81
EBPL	39.74	39.02	38.08	36.81	38.52	40.11	35.48	37.39
FGFR2	41.94	40.37	43.12	43.76	38.21	40.11	39.05	37.83
CORO1C	38.29	38.11	39.62	41.12	41.5	40.13	43.68	42.51
TKFC	45.33	43.32	39.33	37.9	38.15	40.22	31.45	33.65
ITPR3	36.91	36.01	36.82	35	38.98	40.22	34.96	37.93
NARF	44.85	45.32	44.97	40.82	39	40.23	37.38	38.77
TMEM134	41.13	42.89	43.89	41.19	35.97	40.29	30.43	33.84
ACSL3	36.44	34.75	40.98	48.55	41.33	40.3	63.46	54.2
NDST1	35.84	33.83	34.67	35.22	40.29	40.31	37.76	38.81
MRPL11	35.8	34.28	34.03	33.56	39.6	40.36	33.59	34.02
ACAA1	41.45	43.95	38.95	35.52	39.63	40.36	34.11	35.21
SMARCC1	39.75	39.11	40.97	41.81	41.99	40.38	42	42.74
ANAPC13	35.97	36.53	37.77	36.11	41.04	40.42	49.58	45.87
IGF2BP2	42.81	44.67	46.25	49.9	43.14	40.46	49.4	47.66
HNRNPR	42.45	39.5	44.41	49.02	42.19	40.5	53.99	48.99
CALM1	42.36	40.58	43.29	43.28	40.39	40.51	45.04	42.07
PFDN6	40	39.87	37.89	41.73	44.04	40.52	37.24	42.45
TMEM160	44.08	39.58	36.83	31.69	39.31	40.53	25.16	29.73
TUSC3	40.82	41.03	41.91	46.84	42.9	40.55	53.51	51.44
FBXO2	35.63	36.38	34.21	30.5	41.21	40.56	29.76	34.71
COX17	39.91	36.22	36.72	39.68	44.04	40.57	46.7	41.58
TYK2	40.26	37.34	37.31	35.85	36.84	40.58	32.94	36.43
BCL7C	39.59	43.36	38.31	36.59	39.96	40.65	30.91	29.13
UBQLN4	42.68	41.86	39.36	38.1	41.05	40.67	33.98	39.2
UBE2Q1	38.49	41.84	39.51	40.8	43.76	40.67	41.54	42.87
PLBD2	38.17	38.85	36.77	37.64	38.76	40.72	34.51	37.49
SNAPIN	41.39	44.58	43.54	39.66	38.41	40.74	34.46	35.99
SRRT	39.41	37.05	38.35	39.92	42.43	40.77	41.9	39.69
SQLE	36.61	35.56	38.11	37.82	39.86	40.78	44.87	43.19
ZFP36L1	51.39	51.96	50.96	52.56	41.52	40.84	38.98	46.17
NDUFB1	37.81	36.89	32.76	34.71	38.61	40.9	35.5	32.63
LRRC41	44.12	47.33	42.98	41.08	41.26	40.94	34.9	38.99
PRELID3B	36.41	34.5	39.01	44.82	42.85	40.94	66.95	58.28
NOLC1	34.66	32.95	34.56	37.5	43.11	40.98	43.94	43.72
MARS	41.38	41.58	39.52	38.03	40.66	40.99	36.98	37.44

PPIH	39.71	37.71	38.61	38.8	40.29	41.03	39.13	38.4
PODXL	45.51	47.79	47.55	49.81	42.26	41.04	46.01	45.58
MCM2	47.22	44.54	44.87	40.05	39.58	41.05	33.35	33.23
ABHD14B	38.67	41.3	37.67	36.06	41.97	41.06	35.23	36.51
EIF2D	39.99	39.57	38.42	36.25	43.19	41.06	42.01	41.6
GBAS	44.03	40.3	47.87	48.11	41.75	41.06	52.38	47.82
CIZ1	42.36	43.13	41.55	40.42	38.55	41.07	33.71	37.58
TMEM120A	34.72	39.44	37.89	37.8	41.34	41.09	35.05	39.45
TMEM179B	46.67	50.42	43.98	42.8	44.57	41.09	39.79	40.61
GNPTG	38.69	39.32	37.35	38.01	42.65	41.1	35.52	39.75
SNRPN	44.25	44.44	43.02	42.11	39.3	41.12	35.06	36.33
SNURF	44.25	44.44	43.02	42.76	39.98	41.12	35.06	37.23
OTUD5	40.41	43.21	41.46	40.14	42.41	41.13	36.74	38.05
VASP	41.68	42.18	38.8	36.77	40.52	41.14	32.68	36.22
KIAA0319L	43.92	45.7	42.41	44.31	40.16	41.15	41.34	42.93
BLVRA	40.88	41.38	42.02	39.74	39.82	41.16	40.21	43.22
COPS7A	41.07	43.06	37.44	37.85	44.11	41.18	37.23	39.22
NELFB	41.39	40.07	41.2	35.23	41.86	41.2	33.99	35.13
INTS1	40.77	39.15	38.74	35.67	38.71	41.26	31.6	34.02
SELO	34.95	34.8	34.05	32.64	37.15	41.29	27.61	32.17
HNRNPUL2	44.38	45.86	46.42	44.79	41.63	41.29	41.35	42.17
IMP3	38.33	38.69	39.37	34.49	40.58	41.3	34.16	36.79
CERCAM	43.37	44.84	42.71	39.99	34.86	41.33	30.33	33.03
ANP32A	42.6	40.68	43.4	44.55	43.47	41.33	48.06	47.22
GSR	39.9	39.33	39.06	37.61	43.31	41.34	44.68	43.18
ARAP1	38.8	40.96	38.57	36.91	40.74	41.35	35.51	37.75
MTHFD1	38.91	38.08	39.66	38.42	42.19	41.35	42.91	39.74
SHARPIN	42.51	42.15	38.28	36.54	37.95	41.41	31.08	35.18
ANXA7	40.36	40.17	41.39	40.09	44.98	41.41	48.51	44.52
PRRC2B	41.89	43.16	41.44	42.27	38.65	41.45	37.74	40.64
KIAA1217	40.43	39.55	42.47	46.05	41.05	41.49	46.26	45.91
COPS9	42.45	46.17	39.06	38.38	38.62	41.51	33.92	34.25
PRPSAP1	41.79	44.56	42.46	41.23	39.26	41.54	37.7	40.07
NIT1	38.46	38.57	37.83	36.29	42	41.55	36.51	35.76
ANAPC5	42.24	42.76	41.74	39.8	43.5	41.56	43.32	39.97
HP1BP3	38.4	36.6	41.31	45.21	44.73	41.58	60.32	53.23
ARFRP1	43.78	46.66	41.31	41	40.98	41.63	32.96	37.83
CFH	51.49	48.61	54.78	64	39.73	41.67	67.48	60.03
CD59	37.66	38.19	39.5	39.91	41.71	41.68	47.11	43.45
EIF4E2	37.87	42.81	39.39	36.21	41.01	41.71	35.86	40.24
EXOSC6	36.94	35.84	36.02	31.93	37.04	41.72	29.72	33.45
SAMD4B	41.51	41.31	40.95	41.55	40.86	41.73	37.83	38.03
UTP18	34.8	35.02	37.14	34.47	41.72	41.73	42.03	39.65
FGFR3	41.96	44.5	42.99	40.48	36.81	41.77	31.85	37.22
MXD4	40.08	42.16	36.58	34.22	39.8	41.78	28.13	34.18
FRMD6	48.16	42.81	48.47	51.24	45.69	41.78	51.91	50.56
SDCBP	39.1	39.61	43.42	45.9	42.12	41.79	58.08	51.48
RNF10	38.94	42.83	39.44	39.5	39.63	41.81	37.2	38.09
POR	41.67	44	40.13	38.71	41.2	41.89	33.65	37.81
AMPD2	44.27	44.9	42.49	39.68	42.45	41.93	35.35	38.06
MLX	40.11	40.82	38.98	38.38	44.55	41.97	42.9	42.83
FXYD3	35.09	32.19	31.86	32.21	38.8	41.98	35.88	40.48
NR1H2	46.65	45.13	40.36	39.46	39.93	42.01	31.83	35.55
UNC45A	44.01	45.18	42.6	39.82	44.05	42.01	33.95	38.33
ZNF593	40.73	41.39	40.51	39.08	41.9	42.04	29.42	33.81
CLNS1A	38.14	40.58	43.58	42.96	46.13	42.04	49.14	43.13
PEMT	35.59	35.91	32.46	32.37	38.39	42.09	32.07	34.33
DFNA5	44.01	47.4	46.58	42.24	40.05	42.1	42.72	44.31
CPNE2	51.19	52.51	47.71	46.33	38.1	42.11	31.97	36.47
TMEM183A	38.23	40.8	39.15	37.36	43.51	42.11	41.01	39.12
SMIM7	39.78	44.7	40.39	41.41	43.23	42.14	38.58	42.77
DDRGK1	43.49	43.76	42.39	39.14	42.71	42.15	36.66	39.11
ENG	41.5	41.64	37.04	37.19	40.73	42.18	33	37.02
COG4	41.63	41.77	40.61	39.71	41.4	42.2	38.89	40.32
TFPI	44.01	45.45	49.86	53.9	43.04	42.25	68.18	57.39
SNN	39.41	42.99	40.14	39.27	41.84	42.26	40.02	42.2
ATF6B	45.2	44.76	42.25	43.26	41.4	42.28	40.07	41.45
RPL7L1	40.52	39.96	43.08	41.39	44.59	42.32	46.53	44.54
LYPLA1	34.75	33.49	40.79	42.33	45.14	42.32	64.43	55.88
RAD21	39.51	35.96	43.82	48.54	45.73	42.32	65.48	56.52
CRTAP	42.13	41.3	41.85	40.07	43	42.41	40.03	42.51
DGCR2	44.46	46.53	41.37	42.49	39.98	42.42	32.08	37.04
PTGES2	51.94	48.91	46.79	44.43	43.83	42.42	32.69	37.06
CELSR1	46.95	45.01	44.92	46.29	40.31	42.42	38.64	41.99
PLEKHJ1	40.65	43.34	39.75	36.78	43.78	42.43	35.53	37.11
YDJC	36.73	37.57	35.86	33.25	40.64	42.46	31.05	36.61
PTDSS1	36.77	36.24	35.68	36	43.22	42.46	39.91	42.51
PFDN1	42.49	41.63	43.52	45.07	45.96	42.56	43.79	45.41
SNX7	44.44	44.51	48.46	49.61	46.9	42.56	52.75	49.36
NFIX	46.19	46.62	44.12	44.48	42.54	42.57	35.13	38.23
MYO1C	47.8	47.92	44.05	43.46	43.63	42.66	34.49	37.87
FH	42.27	40.53	39.57	39.09	43.32	42.69	45.03	40.96
TCEA1	41.16	41.48	46.12	47.82	45.79	42.69	56.63	52.12
CALD1	35.2	33.82	40.16	49.35	41.82	42.69	61.6	56.9

TMEM8A	42.3	43.57	40.84	39.29	40.28	42.71	31.09	35.55
PCYT2	35.46	37.67	35.11	33.59	36.42	42.73	30.64	35.89
INPP5D	33.18	33.23	33.54	30.86	42.5	42.74	38.95	40.01
EMC7	45.3	45.12	40.08	42.03	42.34	42.75	49.14	43.55
KAT2A	36.28	35.72	35.96	36.69	40.95	42.76	45.19	42.57
LONP1	44.61	45.23	41.39	39.64	40.58	42.77	32.65	36.16
PITRM1	36.89	38.52	36.95	35.86	43.23	42.77	43.33	42.37
ERCC1	43.26	45.4	44.68	43.36	41.43	42.78	36.69	36.82
CPT1A	45.58	45.25	43.52	41.18	48.24	42.79	42.41	42.65
M6PR	39.96	40.37	41.39	40.15	42.58	42.79	46.52	43.11
SNRPF	43.22	42.95	42.89	43.95	41.76	42.79	42.34	43.81
NPM3	36.39	38.54	35.55	31.62	46.4	42.81	34.13	39.21
MCRIP2	36.49	36.91	35.73	31.26	40.6	42.82	26.62	31.63
CPSF4	41.78	44.95	41.72	39.37	41.69	42.86	37.33	36.81
GLB1	42.42	45.62	42.53	41.65	43.05	42.9	41.36	41.4
C6orf62	44.18	41.52	47.89	52.04	44.42	42.92	58.24	53.02
MRPL57	43.17	41.07	43.77	38.23	45.18	42.93	37.23	35.44
NXT1	41.53	44.75	42.17	39.13	44.98	42.98	37.89	37.03
AP2A2	41.16	43.96	42.82	38.94	39.45	42.98	35.95	38.46
PTPN1	41.86	41.87	43.07	41.94	42.43	43.05	41.14	42.64
CITED4	48.87	51.41	45.83	41.47	38.96	43.06	29.52	32.05
RARS	39.03	35.31	41.35	42.27	45.25	43.13	54.92	50.05
LIMA1	43.68	40.04	50.36	54.52	49.23	43.19	66.07	57.65
PHC2	53.07	52.76	51.51	47.88	43.59	43.21	38.1	39.03
SAFB	47.68	48.41	46.32	44.76	45	43.21	40.81	41.83
STX16	37.09	36.9	39.56	47.5	42.91	43.23	59.23	51.71
NABP2	42.38	43.67	40.39	39.42	47.59	43.27	39.87	43.04
IDH3G	45.91	48.73	43.26	37.78	44.63	43.28	38.25	38.96
NAA38	43.01	46.22	44.28	35.66	41	43.29	33.85	34.43
CBX1	44.06	38.78	44.64	49.22	46.19	43.32	53.48	50.37
NPDC1	36.28	37.02	34.38	34.41	40.54	43.34	33.63	37.31
GARS	44.31	44.45	43.71	44.9	44.68	43.34	46.88	45.29
EIF3CL	29.4	32.2	37.03	31.52	37.03	43.38	27.12	24.27
PDZD11	39.59	42.4	39.64	40.98	41.6	43.39	45.97	41.43
SUMO1	41.8	42.59	45.17	49.65	44.94	43.41	63.06	53.15
SLC12A7	42.78	43.51	42.04	40.55	43.39	43.43	36.97	39.27
TNRC18	41.39	40.74	41.02	40.61	37.92	43.45	34.39	37.28
SH3GLB2	42.28	43.85	41.75	39.16	44.13	43.47	40	39.84
PIM3	44.85	43.34	44.7	37.9	40.43	43.5	32.32	35.13
IFRD2	38.26	37.47	36.44	34.92	43.69	43.54	33.99	36.05
TMEM94	41.09	42.28	40.53	39.49	41.59	43.54	37.56	39.44
ATG4D	42.42	41.06	40.42	35.81	40.88	43.61	28.79	34.98
ARSA	47.39	48.43	45.34	44.45	42.53	43.62	35.16	37.44
ANKH	40.81	38.24	40.19	40.24	45.12	43.63	42.94	39.7
SLC39A6	37.02	34.82	38.99	46.09	47.65	43.65	61.77	54.11
PLS3	42.4	39.91	46.34	48.89	46.6	43.68	61.09	52.3
KLC2	38.53	39.68	37.71	38.02	43.88	43.71	33.95	37.32
DRG1	43.3	42.21	40.43	40.58	46.43	43.77	44.35	44.2
ESRRA	38.19	39.83	36.17	33.82	45.5	43.79	34.64	37.72
SEC24C	47.1	48.96	47.24	46.32	45.83	43.81	43.08	44.12
FBXW5	41.68	41.21	39.88	37.2	40.5	43.82	30.35	34.64
POLR2J3	44.66	39.06	47.29	46.72	46.51	43.82	39.31	47.86
DEK	42.51	39.67	44.4	47.44	46.77	43.82	66.06	57.64
KLC1	44.39	44.23	46.78	45.37	45.9	43.85	46.05	46.49
NADSYN1	36.91	36.2	34.62	38.32	42.1	43.86	41.32	45.22
PAICS	42.38	38.2	43.52	44.16	47.08	43.89	56.68	47.89
SRSF6	39.46	37.3	41.02	47.3	46.31	43.92	64.8	54.89
LUC7L2	43.21	41.49	44.61	45.26	44.15	43.93	50.29	45.28
WIPI1	37.62	43.14	40.97	39.44	45.36	43.93	42.16	45.52
WBSCR27	39.69	44.32	37.47	38.64	42.11	43.94	34.67	35.23
RHOG	43.75	45.79	43.32	37.99	40.82	43.96	37.86	37.41
ARHGEF1	48.7	48.96	45.35	44.18	41.8	43.96	33.45	37.92
RPL26L1	39.26	38.21	39.31	37.62	44.49	43.98	42.91	41.98
DPM1	46.04	39.12	44.45	47.23	44.96	44.01	59.81	51.19
PPP2R2A	46.91	43.67	50.68	51.94	47.19	44.04	57.96	51.15
MRPS7	40.42	40.09	39.69	38.02	43.89	44.05	40.8	37.41
PCNP	35.44	38.99	37.9	43.04	45.06	44.06	61.81	55.97
NOL4L	51.16	50.97	48.12	52.04	43.42	44.07	41.13	44.15
SRSF5	39.5	38.55	39.63	47.13	44.37	44.09	64.3	57.98
LUC7L3	38.94	39.96	42.78	52.26	42.58	44.09	72.68	64.08
STAT1	79.62	76.06	80.49	84.67	42.03	44.1	55.07	44.65
DOCK6	45.78	45.85	45.1	43.4	43.12	44.17	37.35	41.33
HMG20B	45.89	45.85	44.65	41.48	40.1	44.2	33.97	37.78
HSPBP1	45.29	46.32	43.18	39.06	44.69	44.21	33.87	35.82
TMEM106C	40.89	42.47	44.16	40.65	47.35	44.25	47	44.5
FAM213A	39.81	38.19	42.53	43.39	42.08	44.27	53.25	47.87
CD164	44.18	42.28	46.99	51.68	43.16	44.28	68.44	59.16
TMEM184B	45.26	47.33	43.16	43.97	42.96	44.29	37.17	41.6
ORAI3	42.66	45.94	44.82	42.09	44.43	44.3	36.57	41.39
NFIC	48.83	51.77	50.45	48.31	41.82	44.31	35.28	42.37
SSB	36.88	38.7	42.19	49.75	47.53	44.36	73.44	62.62
METTL5	37.16	42.56	39.42	38.7	44.43	44.43	51.26	51.84
LAMP2	42.43	38.45	46.8	50.58	44.99	44.46	58.99	54.7
IQGAP1	41.72	37.28	46.8	48.67	47.63	44.47	58.46	54.05
MOSPD3	45.77	46.85	44.71	44.62	45.5	44.5	36.66	39.3
CHCHD5	44.67	46.46	43.9	42.31	45.99	44.5	31.33	40.21

MAP1LC3A	48.9	45.44	41.72	39.59	43.23	44.52	30.37	33.04
RNF220	42.18	41.32	42.42	41.37	42.94	44.52	36.25	37.49
GMPS	43.33	42.68	45.34	44.47	46.07	44.53	51.81	47.45
JOSD2	42.32	44.71	39.62	37.07	40.48	44.56	29.07	29.69
MBD3	42.81	43.76	38.84	36.79	42.48	44.6	30.95	33
STK25	44.11	44.01	42.81	42.68	42.38	44.6	37.86	40.12
SBDS	39.09	35.46	41.9	42.97	44.34	44.61	59.42	50.43
DDX27	42.88	39.96	42.78	43.21	48	44.65	46.04	47.05
FPGS	42.07	41.61	41.42	38.32	42.86	44.67	33.57	36.25
DUT	46.74	44.09	41.93	43.19	43.79	44.67	49.99	40.18
CUEDC1	50.76	51.46	48.11	47.31	42.6	44.69	34.92	38.82
RUSC1	45.66	46.82	44	44.64	46.92	44.7	40.87	40.51
ALDH18A1	47.04	48.17	45.3	45.41	46.22	44.71	42.3	42.64
PLCB3	45.29	45.72	43.05	41.56	44.41	44.71	39.3	43.1
USP5	46.49	44.83	44.84	42.27	45.86	44.72	39.42	40.01
DFFA	40.49	42.93	40.23	42.06	44.06	44.79	43.26	43.18
XPO1	39.12	34.72	45.54	53.1	45.48	44.81	78.17	60.59
UCKL1	47.41	44.86	42.66	42.18	45.73	44.88	39.2	43.53
SUPT5H	49.35	51.06	46.07	45.38	45.42	44.94	37.27	41.81
AHCYL1	42.84	46.12	43.72	44.17	47.62	44.95	51.56	49.36
ETF1	40.46	38.31	42.17	43.56	46.34	44.97	52.24	50.23
CELSR2	52.73	53.78	51.64	52.58	41.02	45	38.64	43.98
WDR6	41.9	43.43	40.74	39.89	45.1	45.03	42.15	43.18
NDUFB6	42.33	44.2	42.02	39.69	49.13	45.04	46.23	43.51
ATRN	46.45	42.93	46.94	46.66	46.85	45.08	52.49	49.37
SUPT6H	50.19	50.46	49.4	49.83	45.05	45.12	45.05	44.99
FLNB	52.74	50.97	50.33	47.38	44.04	45.13	41.3	42.93
TNPO2	40.02	40.83	39.65	40.15	44.36	45.17	43.74	44.36
VPS51	44.68	41.32	40.69	37.89	41.22	45.19	30.08	33.85
ACP6	32.77	35.59	32.55	32.43	42.84	45.2	40.88	42.74
EPAS1	49.64	52.91	51.44	51.69	45.99	45.21	45.22	46.31
MRPL2	43.12	41.73	44.08	38.4	46.99	45.22	39	38.52
NADK	43.46	44.58	41.9	42.15	47.52	45.27	40.06	43.73
SLC25A22	43.23	44.48	41.9	39.81	45.62	45.28	36.95	39.65
TSG101	44.04	45.44	44.44	47.2	51.03	45.29	54.84	49.09
TM7SF2	47.55	51.49	43.05	41.44	41.36	45.3	35.35	39.34
ID1	31.09	33.16	33.16	30.61	45	45.36	39.57	38.86
TSKU	47.56	46.88	44.55	43.68	43.82	45.36	35.14	40.26
MAPK1IP1L	41.71	42.07	42.26	44.85	45.31	45.42	40.88	44.03
FLYWCH1	46.03	44.08	42.37	41.32	43.52	45.5	34.55	40.39
GTF3A	43.84	40.76	43.44	40.12	45.07	45.5	42.93	42.98
RAE1	41.37	42.69	40.59	38.52	47.66	45.55	45.35	46.29
NDN	47.45	50.43	45.2	42.83	46.58	45.56	38.72	40.04
JAG2	47.77	46.1	44.16	43.62	41.29	45.64	34.4	38.82
KIAA2013	52.64	50.69	48.77	45.66	41.96	45.66	34.16	38.22
MMADHC	43.84	44.19	43.18	43.47	48.34	45.66	59.54	49.42
ST6GALNAC2	51.75	55	50.19	52.33	43.33	45.67	39.1	42.26
COPS3	43.43	40.14	44.16	41.38	48.93	45.67	53.75	47.2
CNN3	47.51	46.56	46.5	48.79	46.21	45.69	48.09	48.74
SRP68	48.97	49.4	47.04	47.89	47.87	45.7	45.93	45.02
RAC3	48.93	49.64	49.13	41.41	47.5	45.71	35.36	39.5
LTA4H	36.65	36.7	39.85	38.87	47.19	45.73	59.05	54.45
PPP6R1	44.7	44.97	43.93	40.43	45.17	45.77	37.25	38
MED15	51.82	50.4	47.29	47.91	44.19	45.78	38.11	41.6
EFEMP1	56.3	55.9	61.39	64.15	49.91	45.82	60.46	50.59
TRIM47	45.19	44.84	43.52	38.9	43.2	45.83	33.58	35.12
QTRT1	43.8	42.68	39.87	38.75	43.69	45.85	40.43	38.26
PLK1	33.22	33.84	33.6	28.99	42.31	45.93	35.22	37.6
WDR45B	45.19	46.36	46.33	45.23	47.95	45.96	48.78	45.84
LRIG3	46.29	43.25	49.55	53	47.42	45.99	58.21	51.41
LAMA1	41.52	41.28	40.5	44.11	43.51	46.02	49.94	48.67
THOP1	45.34	42.99	41.66	37.73	44.38	46.03	35.53	35.48
RNF126	43.15	39.4	39.81	39.85	42.96	46.08	32.31	36.92
IFRD1	40.31	38.68	44.31	48.65	47.51	46.1	66.52	56.53
PITPNM1	46.97	48.38	43.11	43.17	42.25	46.13	33.41	38.39
DDX42	44.35	42.08	46.16	50.34	47.9	46.15	59.7	55.6
NASP	44.75	41.48	49.74	51.15	47.69	46.2	59.03	52.4
PET100	52.44	49.73	51.73	48.92	51.02	46.25	42.31	49.65
CBX3	41.16	40.52	45.17	50.53	51.27	46.25	77.47	68.5
CACYBP	47.25	49.12	51.57	53.26	50.96	46.26	57.19	51.51
FNDC3B	48.16	44.06	52.13	59.68	45.1	46.26	58.73	56.11
EMC1	42.31	48.19	43.82	45.52	44.65	46.27	45.72	47.02
ILK	53.22	57.73	51.51	50.81	47.98	46.27	44.73	47.57
PPP1R18	55.86	57.91	54.71	51.92	46.4	46.31	41.33	44.48
ELOVL5	35.77	36.39	39.39	42.76	47.39	46.32	57.17	52.82
DBNL	52.16	53.66	47.6	48.61	45.14	46.36	39.36	42.19
MLST8	41.55	40.79	38.47	35.74	41.67	46.38	32.71	34.95
IER2	54.18	50.4	49.06	48.67	43.1	46.41	35.44	40.82
RELA	46.71	51.13	46.1	44.82	45.46	46.42	38.51	40.93
ASF1B	52.93	49.45	51.87	46.21	44.66	46.43	39.97	37.87
TBRG4	40.82	42.25	40.07	37.21	43.98	46.45	35.6	39.99
TPGS2	44.8	42.49	45.41	43.47	48.48	46.48	46.96	42.88
FAM32A	43.92	43.18	42.53	41.01	46.23	46.55	44.65	44.39
OTUB1	48.49	51.08	46.05	44.3	45.99	46.57	38.76	42.46
RXRA	51.56	52.13	49.23	48.16	44.72	46.58	37.56	42.52

TMEM141	49.86	52.16	48.77	43.73	50.45	46.63	40.89	41.65
KLHDC3	48.74	47.95	46.29	42.41	44.67	46.65	39.06	38.7
MCFD2	44.79	42.26	47.1	49.04	48.85	46.68	59.21	53.06
DSG2	44.8	41.62	52.25	61.71	45.36	46.7	72.05	64.68
ATXN7L3	46.94	49.24	46.03	45.18	47.44	46.71	40.93	44.8
TRIM8	44.62	49.11	45.34	43.59	43.35	46.73	37.35	43.31
CCDC47	42.6	38.18	47.48	52.84	50.29	46.74	68.12	59.83
EXOSC4	47.66	46.45	40.49	37.21	46.68	46.75	31.23	35.1
MICALL2	35.34	35.2	35.15	38.95	45.88	46.76	45.46	45.67
PPP1R9B	50.58	50.39	48.26	44.76	44.45	46.8	36.89	41.56
AHNAK2	45.45	45.76	46.31	47.59	43.01	46.83	45.2	46.29
RHEB	42.69	41.22	41.33	43.88	46.31	46.83	52.17	47.65
TBC1D9B	50.05	47.88	48.09	48.18	46.31	46.84	41.84	42.47
MAP1LC3B	41.67	41.99	39.67	42.48	47.57	46.87	53.66	46.1
SPG21	45.57	47.1	45.74	45.32	49.35	46.89	50.18	48.76
ROGDI	49.61	48.83	45.31	44.38	45.41	46.96	32.84	43
B4GAT1	45.05	47.23	43.46	41.05	47.29	46.99	37.84	41.06
SUN2	46.45	47.27	45.39	41.72	46.93	46.99	39.64	44.82
SLC16A1	49.81	44.57	50.97	55.23	50.54	47.01	62.79	55.71
HDAC2	47.52	46.27	51.15	56.08	50.65	47.07	66.2	62.26
TMED1	45.07	48.51	42.26	41.51	41.92	47.1	32.95	38.08
MRPS21	41.83	45.13	43.01	44.11	47.67	47.11	49.13	46.15
NSUN2	40.93	40.06	43.61	42.78	50.1	47.12	53.9	50.6
CHST12	45.49	48.49	45.34	40.55	45.21	47.15	32.52	36.77
LIMK1	54.35	57.81	52.17	49.07	45.95	47.15	36.89	41.94
NUDT14	47.34	48.36	42.54	40.13	43.82	47.17	34.27	36.74
PDGFA	54.28	53.92	55.91	56.43	47.25	47.19	43	45.13
TRMT1	38.93	37.16	36.23	35.16	48	47.21	41.64	41.28
SMO	49.62	49.16	48.34	45.29	44.73	47.23	42.17	44.76
MMP1	45.29	47.43	43.41	41.9	41.24	47.23	45.49	46.14
PDDC1	42.92	40.74	39.35	38.69	44.68	47.25	41.51	41.05
RAB34	51.55	53.15	51.97	44.79	48.14	47.25	40.13	43.98
DPY30	48.17	48.56	46.89	55.13	51.54	47.26	54.42	52.99
DGKZ	50.02	48.76	46.55	43.87	46.51	47.32	36.96	42.01
MPLKIP	46.09	43.83	42.26	44.57	46.75	47.37	39.55	42.18
LPCAT1	45.33	45.9	46.28	44.18	45.8	47.37	43.97	45.25
C12orf10	43.84	47.85	46.66	42.8	48.64	47.4	39.03	41.73
CHERP	47.29	46.98	44.28	43.43	45.39	47.43	39.03	42.55
SNRPE	49.09	48.42	47.48	51.79	52.84	47.48	59.74	56.01
SNRPA	49.71	48.16	49.29	47.13	47.04	47.49	40.98	42.39
HEXA	45.17	47.46	42.86	44.53	48.61	47.52	43.78	45.43
IGF2R	40.93	43.67	42.73	44.85	45.45	47.56	48.43	50.42
TMC6	43.1	43.46	40.86	40.93	43.66	47.57	36.66	41.74
ALKBH7	46.71	47.57	41.66	39.7	41.83	47.59	30.36	31.3
SDC3	54.78	58.8	54.53	54.63	47.5	47.59	40.52	45.74
HNRNPA3	49.35	44.82	51.05	56.69	50.74	47.69	61.81	56.98
MEPCE	44.11	44.93	43.45	42.26	45.45	47.7	40.48	40.02
NBR1	43.81	42.17	44.8	47.49	49.93	47.7	55.97	51.79
SMARCE1	47.6	43.89	52.68	56.35	53.4	47.82	63.44	59.1
PRMT5	46.38	47.77	46.33	46.5	51.71	47.85	46.04	48.55
CIC	49.45	50.71	47.64	47.52	45.41	47.86	39.54	42.37
HES1	50.04	50.1	50.24	44.29	44.2	47.89	38.44	44.27
CSRP1	70.38	76.8	70.06	65.64	44.69	47.9	41.32	46.79
SORBS3	57.64	59.17	55.49	54.55	47.04	47.93	37	43.51
ESYT1	48.15	50.27	45.71	44.81	48.49	47.93	43.14	44.15
PTPRF	56.36	56.18	54.29	53.27	44.12	47.93	39.9	44.22
SLC39A7	43.91	47.16	45.05	45.1	48.75	47.94	44.69	47.35
HYOU1	51.09	53.63	50.27	51.68	48.8	47.95	44.88	46.55
ASS1	39.67	41.02	36.49	34.41	48.37	47.97	38.84	42.22
EXOSC5	39.46	43.17	40.4	36.56	49.44	48.01	35.26	36.8
RCC1	43.58	44.84	43.39	41.23	51.24	48.04	45.72	44.07
WDR54	62.7	61.37	57.41	52.11	48.61	48.14	38.38	42.17
MRPS18B	46.17	47.77	48.77	44.13	52.81	48.18	45.56	48.1
PSMC2	44.79	41.91	47.74	50.04	48.62	48.19	54.16	52.46
UQCR10	44.7	46.15	44.4	43.58	47.64	48.22	45.59	45.19
TMEM161A	43.99	45.07	43.88	39.55	47.24	48.27	39.69	42.39
CES2	40.34	41.12	39.25	38.01	47.1	48.32	40.38	44.07
MRPL55	49.74	47.1	44.59	45.62	49.93	48.34	41.1	38.63
UBQLN1	46.78	45.62	49.41	49.14	48.26	48.41	56.48	50.75
DDX24	56.46	57.2	56.73	56.18	51.4	48.42	49.65	51.25
CMTM6	41.11	38.42	45.36	50.91	51.72	48.42	69.72	59.77
CD276	51.73	54.56	49.05	49.01	44.6	48.44	38.72	42.4
SYNCRIP	46.67	45.18	51.95	52.88	51.49	48.46	65.16	57.94
ABCF2	42.92	44.15	41.9	40.88	50.02	48.49	47.35	48.13
BZW1	45.22	39.94	51.61	52.93	48.22	48.49	68.39	58.59
MRPS16	44.41	44.14	42.79	42.77	47.02	48.54	41.61	41.88
IFI35	89.37	88.76	87.35	82.84	45.05	48.57	43.22	41.26
MIDN	59.75	59.07	56.1	54.85	45.25	48.61	35.67	43.43
REEP2	43.75	44.36	44.89	41.54	50.71	48.68	38.37	44.56
RNF145	45.27	43.6	48.77	52.37	51.1	48.71	58.24	54.21
AKT2	45.43	48.64	43.43	45.02	44.81	48.72	39.69	41.73
APOBEC3C	50.5	50.59	47.82	46.14	49.39	48.72	45.97	45.36
TRAPPC6A	52.29	48.24	52.11	45.48	45.98	48.74	36.37	40.08
DHX9	49.82	48.05	50.87	52.58	52.6	48.76	60.21	55.01
PLEKHH3	51.42	51.37	47.8	46.1	46.47	48.81	34.94	40.37

GART	46.77	45.17	45.92	44.16	51.57	48.81	52.81	50.35
YPEL3	43.09	42.51	42.05	36.54	47.72	48.83	39.4	41.01
TAF6	53.05	52.51	51.13	48.05	47.87	48.87	43.36	44.12
PIGS	54.38	57.7	53.97	53.72	48.11	49	41.9	48.57
DDX17	49.04	46.52	51.1	56.07	50.39	49.01	64.82	58.73
CCDC3	35.04	38.59	34.35	32.16	48.35	49.03	42.16	45.98
TMEM101	48.64	48.36	45.61	42.1	47.64	49.05	40.65	45.94
NDE1	50.05	55.25	52.17	48.63	50.62	49.14	48.9	51.25
H2AFX	56.09	58.1	49.29	46.55	45.11	49.15	33.29	37.33
PDLIM7	52.81	54.64	50.01	47.73	43.34	49.15	38.22	44.51
SCRN2	46.22	47.87	44.44	43.87	46.17	49.16	36.19	39.13
MAPKAPK2	48.8	52.06	48.92	48.21	45.51	49.16	39.59	44.62
ARFGAP1	46.77	47.37	45.85	44.39	50.58	49.17	41.64	44.33
RCN2	42.75	39.24	45.03	48.86	49.54	49.17	67.58	59.33
ADA	43.67	44.39	42.19	36.99	48	49.2	38.62	43.79
RFNG	43.61	44.31	41.46	40.01	49.59	49.27	40.1	44.28
MARCKS	53.07	49.65	54.28	56.11	48.57	49.34	47.72	48.99
SPNS1	40.56	42.81	40.18	39.06	45.23	49.35	36.6	41.82
TP53	50	52.05	46.76	45.78	49.98	49.4	42.09	46.07
ATP13A1	43.12	44.87	41.74	39.84	47.9	49.41	38.88	43.48
PMPCB	42.51	40.8	43.93	45.4	48.91	49.41	58.53	53.4
ETFA	45.83	43.18	47.86	48.99	49.96	49.42	58.44	51.31
CMIP	48.93	51.66	48.94	48	48.72	49.45	44.97	49.27
EIF2AK1	44.88	45.3	46.85	46.64	50.68	49.47	52.26	49.88
SYTL1	51.15	47.87	46.6	45.1	44.77	49.56	43.47	44.26
SF3B1	46.61	42.15	53.27	63.68	50.48	49.59	84.1	70.28
PAIP2	50.2	49.58	53.24	54.35	53.21	49.64	69.6	62.47
ILVBL	47.56	46.26	43.81	41.05	45.71	49.68	35.01	38.9
YKT6	46.65	49.23	46.79	44.32	46.71	49.68	44.01	44.29
TRA2B	53.3	49.68	56.13	56.04	52.58	49.7	60.52	54.65
POMP	45.07	50.63	49.23	45.62	50.56	49.73	58.77	51.99
BLOC1S1	44.63	50.85	46.85	43.87	48.51	49.75	42.05	39.63
NPLOC4	46.88	49.59	47.94	45.54	48.65	49.77	44.05	46.03
TOP1	45.96	41.84	51.63	55.69	53.48	49.86	66.34	61.26
GRK6	47.44	48.7	46.27	43.35	48.64	49.92	37.43	40.79
PLPP2	33.13	31.99	32.18	32.66	49.76	49.95	40.41	44.93
SCRN1	49.91	51.76	52.29	50.58	52.21	49.97	54.58	54.16
DNASE2	51.92	52.13	51.43	47.15	49.68	50.05	43.14	46.41
OXLD1	46.43	48.18	43.3	40.72	46.13	50.08	33.79	39.38
ISOC2	46.6	47.27	42.9	40.84	51.41	50.08	41.87	44.56
PPP2CA	50.7	47.46	54.2	53.2	50.51	50.08	61.16	55.96
ANXA11	52.99	53.77	50.43	48.47	45.32	50.13	39.88	41.08
HMGCS1	42.75	43.67	49.33	60.25	49.07	50.17	70.69	70.08
ICAM5	52.1	54.86	49.45	48.65	43.29	50.21	39.6	43.14
RBM39	49.84	46.24	53.4	62.93	51.64	50.23	79.73	66.96
ATP5G3	54.57	53.81	50.74	48.66	52.7	50.25	47.09	47.04
UBE2L3	47.62	51.24	50.98	53.36	50.07	50.25	51.03	51.36
TRPT1	52.37	53.19	51.54	47.4	50.86	50.27	40.55	42.88
SAE1	48.11	51.53	47.42	46.56	49.08	50.33	43.9	43.57
TMEM256	51.92	54.21	47	49.75	50.15	50.35	42.46	41.9
SSNA1	48.4	50.13	46.83	41.09	52.62	50.36	36.66	40.76
BCKDK	57.13	56.17	52.72	51.21	48.42	50.4	39.56	44.12
DEAF1	51.3	49.3	47.61	42.44	44.19	50.42	36.36	40.3
POP7	49.98	52.57	51.04	45.63	50.38	50.42	39.89	42.15
SLC4A11	48.09	46.9	47.7	49.99	46.92	50.47	47.33	47.08
CCAR2	56.11	54.32	51.92	51.96	51.37	50.52	45.41	48.24
TTLL12	41.79	41.85	41.78	37.53	50.13	50.53	37.77	41.23
CTDNEP1	46.37	49.47	49.54	45.4	52.41	50.56	44.48	44.86
PNKD	45.42	49.55	49.67	45.55	49.32	50.59	40.92	44.17
TMEM189	47.1	48.95	46.5	45.64	49.33	50.59	43.49	49.97
MRPL53	53.61	53.82	50.57	48.58	51.64	50.65	44.47	44.07
KCNN4	44.79	48.14	43.98	43.31	47.22	50.71	36.62	39.8
CLPTM1	47.09	48.49	44.25	44.29	46.81	50.71	39.68	42.93
FADS3	41.55	40.28	40.71	40.18	53.48	50.73	47.26	46.05
POM121C	48.39	48.32	47.19	49.27	48.27	50.74	44.52	48.1
SHKBP1	50.53	52.06	47.62	44.15	47.66	50.79	37.24	43.32
EXTL3	46.42	49.19	46.23	45.63	49.19	50.85	46.92	47.9
RAD23B	50.91	48.03	52.07	53.95	52.03	50.85	58.26	54.57
SPTBN1	53.13	52.22	53.36	54.12	50.81	50.87	52.75	52.89
SUN1	45.87	44.94	45.44	47.94	50.96	50.88	56.15	53.73
CDK16	51.82	54.4	50.64	49.89	49.14	50.92	42.14	48.41
PQBP1	43.42	41.05	42.77	37.98	47.73	51.04	34.72	37.77
NRDC	48.68	46.21	50.09	51.17	53.18	51.08	62.23	57.81
NELFE	54.13	55.75	49.35	49.21	54.94	51.09	46.09	46.63
SF3B6	48.85	55.36	50.2	54.44	54.9	51.12	64.62	62
LYPD6B	48.58	48.48	51.9	50.81	52.56	51.14	55.61	49.77
PLXNA2	50.81	50.07	50.13	50.1	49.15	51.17	45.01	47.92
RPS19BP1	48.35	51.01	50.21	43.75	49.26	51.19	41.47	41.8
NGRN	50.64	48.27	51.03	48.29	52.35	51.19	48.48	48.92
MEA1	51.98	57.28	52.96	47.85	51.53	51.21	40.91	45.9
SUMO3	47.71	53.46	46.84	49.24	49.75	51.22	45.84	48.16
SF3A3	48.97	48.66	49.92	49.68	56.34	51.22	55.91	52.74
CANT1	52.4	53.87	49.72	46.57	49.66	51.24	41.31	45.78
DIAPH1	55.71	57.12	55.66	55.34	53.24	51.28	52.08	52.35
SF3A1	51.72	53.03	52.73	50.1	54.64	51.29	45.91	49.03
SRSF3	50.49	50.19	53.01	52.42	53.58	51.29	63.35	52.53
FAM127B	51.72	51.35	51.24	50.34	48.84	51.39	41.08	44.31
PHLDA2	39.12	46.16	37.35	38.27	47.52	51.42	31.77	36.42

SPATA20	57.36	53.39	49.87	48.27	50.86	51.43	43.32	45.61
MFSD10	49.99	47.55	47.17	45.42	48.05	51.44	45.08	48.57
CTSZ	56.02	56.71	55.09	53.26	50.64	51.46	40.17	47.62
EI24	44.21	45.54	46.21	43.28	54.23	51.46	53.43	50.52
CDK5	47.9	51.48	46.28	46.24	51.78	51.49	44.6	47.56
LMNB2	56.79	54.81	53.34	49.82	51.52	51.5	40.61	45.23
RRM1	51.74	47.63	52.1	50.08	57	51.55	63.55	55.07
CDC123	40.9	40.18	42.43	45.24	53.53	51.56	60.11	52.04
P3H1	54.99	59.66	53.04	54.1	48.74	51.58	44.1	47.1
IK	53.73	49.7	53.12	56.6	54.48	51.62	60.93	60.67
CTNND1	56.03	55.97	57.99	59.19	54.41	51.67	60.1	58.18
NACC1	50.37	53.02	49.77	47.46	49.47	51.71	38.95	46.51
AGAP3	50.52	51.74	49.3	47.16	52.96	51.77	41.68	47.94
UBE2Z	60.15	59.87	58.5	57.54	53.3	51.8	49.23	49.27
CEND1	40.22	42.18	36.92	35.34	46.19	51.81	33.11	39.16
ALDOC	69.4	73.2	64.43	65.07	47.63	51.83	43.28	51.36
UPF1	56.97	56.25	54.83	51.61	49.28	51.89	42.86	45.48
TCF3	54.48	54.06	52.25	50.93	50.35	52.04	42.72	43.92
PKP3	54.27	51.79	52.56	44.79	48.23	52.1	37.34	40.2
EPRS	47.9	39.28	54.27	57.07	56.16	52.25	73.75	64.03
TAF7	50.6	50.51	50.77	50.96	52.4	52.26	58.49	55.66
EMC4	53.19	53.5	50.77	49.35	57.08	52.27	49.7	48.99
COPB2	53.61	50.26	55.93	58.08	55.31	52.29	66.35	60.27
NKIRAS2	53.51	54.83	53.22	50.27	53.11	52.38	47.83	49.17
TMEM54	63.22	63.33	56.44	52.2	50.21	52.39	38.39	44.97
C19orf48	53.21	48.41	48.91	45.41	48.1	52.42	40.92	41.37
MPC2	47.98	48.05	48.56	47.75	51.84	52.51	52.71	49.65
TGFBR2	52.4	51.03	54.02	59.15	51.45	52.52	61.08	56.19
LSM2	50.99	49.39	48.94	46.8	51.43	52.69	46.19	46.43
ADD1	55.88	59.1	54.16	54.11	50.87	52.72	48.41	50.3
GSK3A	51.33	53.61	52.37	51.1	54.57	52.72	42.78	50.76
PDCD5	52.95	51.76	48.88	54.62	53.41	52.77	54.68	55.23
DDX3X	49.48	43.29	55.21	61.54	55.21	52.79	76.07	68.52
ITGB5	63.41	64.54	59.08	61.48	48.94	52.85	43.95	47.83
FAM189B	45.23	45.78	45.81	42.47	50.18	52.86	42.29	47.43
TPX2	47.85	45.58	45.72	43.38	58.06	52.88	56.08	54.17
ALG3	48.94	49.89	46.99	44.82	56.23	52.9	45.56	47.92
IFI16	68.11	62.88	79.47	89.49	56.9	52.9	84.27	71.49
MRPL41	48.74	46.13	44.84	42.43	50.59	52.91	40.69	44.39
SCAP	52	49.98	46.81	45.83	48.84	52.95	40.19	42.24
SIVA1	61.55	55.54	51.61	49.86	50.31	52.95	38.32	42.5
NRBP1	61.21	60.92	57.74	55.4	53.27	53.03	48.02	51.11
SLC12A9	50.43	50.44	48.49	49.11	47.82	53.06	37.98	46.51
COPB1	53.58	49.21	60.63	62.15	56.92	53.14	76.98	68.83
B4GALT1	56.57	55.73	57.51	56.77	53.49	53.18	51.34	53.33
YIPF2	53.86	52.07	52.06	46.94	52.83	53.21	40.13	46.58
DPP9	50.85	50.32	50.12	47.94	49.74	53.22	43.09	46.35
RNF44	46.9	46.65	47.6	47.22	56.34	53.22	51.22	52.25
MRPS12	49.92	47.88	46.06	42.91	52.64	53.25	38.19	45.81
LDOC1	56.22	56.62	54.31	51.47	50.77	53.25	42.96	48.69
DCAF7	51.9	53.52	52.15	51.86	53.04	53.27	50.8	53.04
LAMC1	51.02	52.28	51.38	54.42	51.95	53.32	56.87	54.8
ATN1	53.38	54.57	52.54	54.28	49.92	53.33	43.79	50.22
PCDHGC3	50.35	55.09	50.4	49.65	51.46	53.37	43.63	49.85
MAPK3	52.9	56.31	49.31	48.99	49.6	53.41	41.28	45.08
USF2	51	52.71	49.55	46.33	53.92	53.42	40.45	43.53
DNMT1	59.26	56.7	57.63	53.75	54.69	53.45	48.55	49.42
POFUT1	54.32	53.88	53.4	53.6	53.3	53.45	51.56	51.03
SCAF1	58.88	58.24	54.07	51.35	49.87	53.46	35.97	41.47
TMBIM1	43.29	49.65	45.67	43.77	51.64	53.48	47.24	50.09
NUMA1	56.78	55.35	54.08	53.07	51.9	53.52	46.33	50.67
GABARAPL2	49.82	50.8	51.38	55.36	56.41	53.56	64.71	59.86
DDB2	48.75	49.12	48.17	46.52	56.85	53.58	51.32	50.02
LITAF	52.9	55.9	55.64	52.53	57.47	53.63	55.41	57.45
ETS2	61.85	61.42	61.36	60.89	58.47	53.7	54.48	55.9
COA3	52.14	50.51	50.89	47.85	52.24	53.73	47.05	49.52
SRC	53.53	55.05	49.04	49.15	51.33	53.74	42.06	46.56
CALM3	56.24	60.08	55.79	54.21	57.3	53.76	46.78	52.04
CD70	51.48	49.98	49.69	46.5	52.93	53.77	44.69	50.2
VPS4A	63.18	62.66	58.06	55.59	49.51	53.81	40.18	47.09
PSME2	56.97	55.3	56.74	52.93	55.58	53.84	57.28	54.13
FOXA2	63.08	63.79	59.23	56.68	49.84	53.89	42.8	45.37
HDAC3	54.72	58.91	55.59	51.96	54.86	53.99	49.54	51.45
PSMB6	57.32	57.5	53.52	50.6	60.67	54.09	47.25	48.67
MRPL18	50.75	51.13	50.18	47	52.41	54.09	51.6	50.55
IRF3	56.75	57.56	54.05	52.18	56.67	54.13	47.85	50.47
SNRPC	55.32	53.89	55.55	52.02	60.97	54.15	54.71	54.11
CDK5RAP3	54.06	51.94	53.48	53.65	56.02	54.16	58.58	59.19
TOP2A	44.97	36.8	52.12	56.86	59.23	54.16	91.24	78.26
KIAA0100	49.64	51.89	50.49	48.61	54.67	54.19	54.38	53.25
PRPF19	57.18	55.7	55.16	51.66	55.05	54.3	46.49	46.78
STK11	49.46	50.73	47.11	46.9	51.14	54.32	41.93	43.87
AMIGO2	47.17	44.27	51.4	59.42	56.16	54.33	74.1	60.98
TCF25	52.87	52.65	50.99	47.48	53.41	54.35	45.44	48.8
ZNF207	59.08	53.54	57.77	62.88	56.15	54.37	68.35	59.6
IMMT	50.62	50.72	52.78	52.23	55.99	54.38	57.65	57.89
FAM162A	63.97	61.29	61.59	63.23	52.36	54.53	61.22	55.49

FAM49B	50.27	46.12	53.45	59.18	58.03	54.55	73.79	62.21
TXNL4A	47.98	50.04	49.51	48.11	57.82	54.58	50.38	46.96
SEC23B	55.46	52.35	53.81	52.62	55.84	54.65	58.34	56.21
VMP1	48.67	51.48	53.6	55.42	56.64	54.75	73.39	62.27
RABL6	52.73	55.55	53.3	50.97	51.52	54.76	41.04	46.71
LZTS2	59.33	60.1	57.84	55.69	52.46	54.86	43.92	48.78
NT5C	49.31	53.05	50.88	46.28	51.58	54.89	41.24	45.77
MATR3	55.6	52.27	61.73	69.35	57	54.91	84.05	72.91
SLC35C2	48.85	54.64	47.21	49.3	55.24	54.92	50.82	49.55
ACADVL	62.31	63.48	60.3	54.33	55.95	54.99	51.56	53.22
RPS6KA1	50.81	52.29	48.73	44.27	57.1	55.01	47.98	51.52
BZW2	49.14	49.23	49.23	51.48	59.53	55.05	62.65	56.55
EMD	54.81	56.1	51.81	51.02	56.67	55.08	43.32	47.22
ARL2BP	51.54	56.36	55.98	56.78	57.18	55.09	60.51	56.57
NUTF2	53.19	53.28	51.7	48.38	58.67	55.11	49.85	50.83
RBM4	63.92	65.97	65.02	59.19	59.12	55.15	47.1	51.95
PRCC	57.59	58.72	57.12	56.52	55.08	55.24	47.93	50.75
IDH1	48.09	48.41	52.69	51.59	58.49	55.27	67.89	62.45
MYO10	52.84	51.63	54.15	57.5	55.09	55.31	57.5	59.19
CNPY2	55.01	52.82	55.26	53.44	54.86	55.33	48.11	48.68
NSMF	50.38	52.23	48.74	46.35	52.69	55.33	43.87	48.79
GLMP	57.01	57.79	55.97	57.48	56.48	55.4	47.25	48.63
MRPL38	54.1	52.24	52.43	45.56	53.08	55.44	43.37	45.68
CXXC5	76.67	73.68	73.05	69.97	54.33	55.46	47.9	50.03
LARP1	50.38	50.16	52.22	53.11	54.68	55.49	54.75	55.02
STAT3	56.39	59.4	55.26	53.2	56.96	55.54	52.97	55.27
PDHA1	52.38	54.21	52.07	52.32	58.79	55.56	57.99	58.2
OBSL1	74.87	72.66	69.03	66.09	48.12	55.69	39.83	44.9
IVNS1ABP	46.77	48.12	50.93	56.44	60.92	55.71	76.66	70.07
NOSIP	56.82	56.96	55.12	46.16	53.37	55.73	38.99	44.17
COMMD4	52.83	53.56	50.58	45.08	53.11	55.86	43.74	45.36
TNIP1	65.7	61.51	61.46	57.68	58.92	55.87	47.61	49.33
ACACA	43.6	48.37	44.18	47.97	54.42	55.87	56.73	59.34
VPS25	56.79	58.02	54.31	52.01	56.81	55.89	51.71	53.57
DCTN2	50.67	54.18	51.84	47.64	56.54	55.92	53.16	54.12
TXNL1	50.48	52.25	54.65	53.98	57.69	55.92	66.44	61.22
UBALD2	57.2	56.58	53.51	48.87	54.3	56	39.54	47.02
CAMTA1	53.87	58.12	57.83	60.15	58	56.03	56.77	54.49
RBM42	58.2	57.81	55.29	50.19	53.88	56.05	39.84	41.88
ETV4	61.91	62.3	59.98	57.43	56.72	56.06	47.57	50.95
TMEM63B	47.31	52.62	48.88	49.43	56.07	56.07	49.27	57.04
RUVBL1	54.39	56.49	54.34	53.6	61.66	56.08	53.17	57.05
OCIAD1	50.78	51.08	55.43	56.2	57.88	56.14	73.66	64.27
MKRN1	59.05	61.58	58.87	60.43	59.12	56.15	59.78	60.19
MKNK2	81.17	77.78	77.71	69.9	53.74	56.16	46.32	50.4
EPPK1	50.62	49.65	48.18	45.35	48	56.17	38.89	44.95
SBF1	53.61	52.8	50.42	47.61	54.07	56.17	43.02	48.15
ATP6AP2	52.47	48.87	54.72	59.84	58.74	56.26	72.67	65.33
THOC3	58.58	57.48	56.21	57.18	59.21	56.3	52.89	53.66
PLIN3	60.41	62.62	58.12	53.87	54	56.35	44.44	49.24
UBE3C	50.33	49.39	53.47	52.86	58.2	56.44	64.54	60.26
POLDIP2	50.39	51.1	49.43	46.46	58.75	56.45	48.2	53.71
C16orf58	59.67	60.09	55.49	54.16	57.6	56.47	46.88	54.19
CCND1	48.42	48.61	49.47	51.68	59.8	56.49	57.54	56.92
BOLA2-SMG1P6	46.93	47.94	48.85	51.71	61.39	56.52	69.24	61.56
MYOF	50.96	49.78	54.66	57.03	60.32	56.53	68.05	61.1
KIAA1522	54.4	53.69	52.08	50.9	51.1	56.67	44.52	50.26
MAN1B1	59.41	61.5	55.3	53.48	54.29	56.73	46.74	49.59
SPATS2L	64.35	62.63	68.92	74.42	58.4	56.83	64.94	57.89
PAK1	50.97	52.9	50.31	49.78	59.08	56.85	60.91	62.33
MRPS26	58.15	59.02	55.79	50.91	59.28	56.91	45.2	48.79
NUDT22	58.1	56.4	54.82	52.59	52.85	56.91	46.74	51.45
FBXL12	63.65	66.2	66.91	62.8	60.31	56.92	61.27	60.39
WDR34	56.74	54.79	52.47	48.68	58.65	56.94	45.64	43.56
UBE2D2	59.64	59.34	58.23	55.36	58.41	56.96	55.74	53.61
TM9SF4	59.07	63.12	58.84	62.51	56.25	56.99	55.12	58.59
CD24	50.51	51.12	56.84	65.11	61.98	56.99	80.79	70.65
TIMM50	51.05	54	50.78	47.71	55.93	57	42.06	48.99
LTBP4	55.8	56.19	52.79	52.92	52.67	57.02	47.12	50.73
IP6K2	58.94	57.74	52.32	56.48	56.68	57.03	54.15	53.11
DNAJC7	52.94	52.82	56.93	56.65	59.57	57.06	66.99	62.26
CHMP1A	58.44	57.95	55.08	52.62	53.58	57.11	44.46	51.4
PNPLA6	52.79	54.55	49.05	48.52	54.3	57.15	43.69	48.4
EXOC7	60.46	59.71	57.56	56.27	58.65	57.15	50.34	55.06
GLO1	57.28	50.8	59.95	60.19	62.41	57.2	75.44	64.36
LIMD2	62.17	64.34	60.21	58.1	58.17	57.23	47.19	54.91
BABAM1	56.63	56.46	55.39	53.33	60.64	57.25	50.25	53.39
NMT1	57.03	58.02	58.78	58.13	57.05	57.26	55.89	56.14
WDR77	54.58	55.43	54.91	53.7	58.04	57.26	57	57.46
C11orf98	55.93	53.79	53.2	54.28	57.76	57.27	52.13	57.11
SNX3	54.39	54.8	57.06	56.87	60.73	57.3	64.98	59.19
TIMM23	53.74	54.28	52.27	50.43	58.2	57.33	49.44	54.44
SEC13	56.17	56.85	51.87	52.37	58.26	57.35	46.89	47.76
RRAS	58.17	58.14	56.45	56.2	53.74	57.39	43.33	45.96
EBNA1BP2	51.87	50.52	50.73	48.92	60.92	57.4	58.9	57.19
NDUFV2	53.7	52.32	55.67	55.94	57.19	57.46	64.46	58.2
KIAA0930	53.47	54.63	52.14	50.09	54.7	57.48	47.11	49.93

CDC20	48.01	43	42.54	37.03	54.81	57.51	41.57	47.02
ABHD17A	60.31	58.19	55.86	50.8	53.47	57.62	38.19	43.51
POLR2E	55.55	56.14	54.68	49.8	55.77	57.75	44.52	49.29
USP11	62.27	67.46	59.73	57.63	54.75	57.75	47.01	50.09
DAG1	58.4	63.87	57.69	59.33	53.76	57.75	50.82	56.3
NSUN5	54.02	53.1	50.6	48.16	56.46	57.77	47.87	48.38
AP1M1	54.28	53.32	52.76	51.31	56.79	57.78	47.46	51.93
SLIRP	58.76	55.98	57.63	61.42	59.45	57.79	65.53	64.02
DDX49	53.35	52.37	50.24	45.52	57.12	57.86	46.49	46.71
KDM1A	63.6	67.13	66.16	62.11	60.44	57.89	57.56	59.66
RAVER1	62.4	61.1	58.93	55.14	53.41	57.91	42.34	48.83
PBXIP1	66.69	71.56	66.32	65.24	55.94	57.91	49.57	56.26
TIMMDC1	49.26	50.35	52.4	54.94	55.62	57.92	61.16	57.37
PES1	58.27	57.58	54.46	51.28	59.27	57.96	52.13	51.72
SHMT2	64.19	65.11	59.51	54	58.36	57.97	46.6	49.08
NCAPD2	55.87	53.3	54.2	50.66	58.26	57.98	54.7	52.75
TSTA3	52.9	57.01	49.28	46.4	54.46	57.99	44.13	49.27
HSBP1	57	58.15	58.28	57.58	59.44	58.05	63.93	60.05
CUEDC2	61.79	63.6	56.59	52.84	55.85	58.09	44.82	49.09
ATP6V1G1	56.89	55.89	57.82	60.69	65.57	58.1	75.32	71.06
PSMC4	59.57	61.4	58.2	54.64	59.6	58.12	50.88	53.92
ADSL	49.71	48.75	51.64	47.61	61.4	58.12	54.99	56.91
TRAM1	57.58	53.86	57.84	63.73	61.11	58.16	85.25	71.17
GUSB	60.74	61.73	57.38	58.78	59.35	58.17	57.95	55.14
MFN2	59.74	59.26	56.99	53.21	59.33	58.25	54.76	58.18
HADHB	59.86	60.58	61.98	55.9	59.45	58.27	62.61	58.31
RER1	56.8	55.56	57.29	55.65	58.86	58.29	56.62	55.5
ACAT2	56.55	60.04	58.61	54.93	59.7	58.29	59.78	61.6
SCPEP1	63.52	64.29	61.87	67.43	58	58.3	65.22	63.54
AAMP	55.12	57.88	54.59	51.74	61.17	58.34	51.23	52.16
RAB1A	54.22	56.06	60.76	62.22	62.6	58.36	73.32	67.64
UTP4	56.79	54.7	56.12	52.48	64.39	58.39	60.23	57.06
STRA13	54.55	57.1	54.23	52.02	59.78	58.4	49.03	49.54
NCK2	64.66	63.15	67.07	64.75	54.9	58.47	51.42	52.53
DNAJA1	55.04	53.2	61.75	65.41	63.57	58.48	74.49	70.65
ALDH7A1	58.88	61.03	59.9	58.88	61.85	58.49	66.93	62.86
ANXA8L1	54.83	50.07	52.16	55.2	59.07	58.54	58.18	56.79
DNAJB11	60.62	57.13	62.61	68.28	61.76	58.54	70.74	64.03
TFG	64.22	61.99	69.25	66.2	58.02	58.59	70.58	64.27
R3HDM4	61.02	56.56	54.94	50.55	57.41	58.6	48.09	53.7
COMMD6	63.62	59.53	56.79	61.17	61.88	58.82	72.46	59.77
CRACR2B	55.57	55.86	53.48	48.51	56.04	58.83	44.81	47.59
C4orf48	53.33	47.4	43.83	41.9	46.3	58.86	34.68	37.73
TMEM173	66.45	67.72	64.1	63.07	60.8	58.86	53.87	55.41
EFTUD2	55.94	57.66	55.39	51.66	57.3	58.88	53.42	53.69
BAD	63.74	65.92	60.2	55.13	56.22	58.9	43.55	46.09
SRRM2	58.69	57.01	58.28	61.52	55.99	58.97	65.99	66.07
PPP1R14C	67.35	67.54	68.33	67.24	59.44	58.98	59.2	62.04
ATP6V0D1	58.95	61.24	54.03	54.8	56.9	59.01	49.8	55.05
ZNF358	62.93	65.9	59.12	57.66	50.65	59.07	38.66	46.3
COL4A6	61.25	63.97	62.78	66.78	55.91	59.07	63.57	64.42
GRB2	58.25	60.32	57.14	58.65	60.31	59.08	53.6	56.86
REEP4	52.99	51.99	51.63	50.51	54.95	59.09	46.39	51.96
SGTA	56.09	60.52	56.32	50.66	61.08	59.13	47.72	49.59
HINT2	57.15	55.42	57.3	55.11	54.59	59.15	45.09	48.28
PSMD7	58.91	54.48	56.41	58.83	62.03	59.17	66.26	62.29
TMEM14C	64.46	63.5	65.76	60.89	62.88	59.18	59.88	56.58
YARS	58.99	59.4	60.89	54.56	60.8	59.2	56.7	54.95
MDFI	61.99	65.22	57.67	56.21	55.12	59.21	43.99	48.63
TMEM248	54.56	55.92	54.8	56.58	59.77	59.3	61.22	61.04
PPP2R4	61.19	61.6	58.64	54.98	57.75	59.31	49.54	50.35
CTBP1	66.19	64.17	64.65	63.04	56.62	59.34	48.93	53.29
SLC52A2	54.02	53.19	49.11	47.31	56.27	59.35	44.26	47.01
DNAJC8	61.97	61.04	58.01	57.93	62.99	59.36	64.51	61.84
HSPH1	53.37	50.05	60.48	66.66	57.92	59.38	80.65	74.25
PLCG1	58.91	60.1	57.69	58.35	60.81	59.42	57.68	59.34
TMEM219	64.83	69.63	62.17	61.48	61.26	59.46	55.7	54.03
VOPP1	68.78	70.45	69.27	65.93	60.38	59.47	57.02	57.68
TSC22D4	59.39	60.45	58.23	56.46	58.26	59.51	44.86	49.98
COASY	58.62	57.79	56.34	52	57.07	59.51	48.95	52.81
BLVRB	68.97	63.77	58.37	53.06	57.35	59.56	45.26	49.78
TMEM147	51.23	52.3	48.45	49.07	52.71	59.59	47.73	50.76
FAM120A	56.14	51.56	57.25	60.24	58.56	59.61	63.07	60.47
SSH3	60.4	59.29	57.91	55.49	55.97	59.64	48.11	56.55
METRN	60.68	59.54	57.61	50.28	54.33	59.65	41.27	46.99
LAMTOR1	58.59	59.32	52.94	53.83	58.57	59.69	52.63	56.91
ARF3	62.71	69.69	62.25	62.4	61.37	59.74	53.03	61.32
DNM2	63.58	65.98	61.26	58.61	59	59.81	49.93	53.49
SLC38A2	50.93	49.9	59.59	68.98	63.27	59.84	91.06	80.76
PRKDC	52.23	49.51	54.76	56.14	61.16	59.9	73.58	67.32
FLNC	51.09	50.34	49.25	46.25	57.15	59.94	46.79	51.57
UROD	57.81	56.73	53.32	52.05	64.95	59.99	49.12	54.17
DGUOK	60.36	66.56	58.38	57.37	62.23	59.99	58.49	55.18
MRPS6	57.01	61.05	60.1	57.18	61.18	59.99	56.55	56.67
NAGLU	54.41	56.9	52.2	51.32	55.29	60.02	43.98	47.41
SRSF2	57.81	54.56	55.98	57.66	59.99	60.04	64.83	64.14
POM121	55.1	56.99	55.24	56.29	60.05	60.23	58.58	61.36

BCAR1	70.73	67.77	64.36	61.19	57.84	60.25	43.67	47.96
CSNK2A1	57.01	59.59	63.44	61.56	62.17	60.25	66.65	63.83
NARS	50.71	47.33	55.7	62.58	63	60.28	85.34	73.76
HLA-E	71.87	75.75	69.56	66.35	61.65	60.33	53.3	55.58
FKBP2	60.74	66.56	66.18	63.56	64.66	60.43	58.26	63.57
CAPZA1	53.44	54.58	55.89	64.66	60.76	60.47	87.13	78.3
NETO2	53.13	50.56	59.28	56.95	61.22	60.48	75.02	73.7
ADAM9	48.65	50.16	56.29	70.36	63.66	60.49	105.88	89.77
MCL1	59.76	57.56	61.96	63.15	62.52	60.51	68.19	63.22
NCOR2	66.08	64.98	63.16	62.29	55.63	60.52	48.27	52.37
PSMA2	55.27	55.7	57.02	56.12	65.42	60.6	73.89	64.98
ETFB	66.27	67.17	66.73	58.82	60.03	60.61	46.37	51.61
MRPL21	56.64	56.89	54.78	50.5	61.35	60.67	49.96	56.01
MRPL27	53.16	52.11	48.78	48.77	60.33	60.73	51.55	52.33
DCAKD	57.29	60.48	54.16	54.24	59.27	60.75	50.67	55.12
RIC8A	60.33	61.82	57.75	56.14	59.06	60.78	51.91	53.69
CHMP4B	69.54	64.41	65.25	63.79	64.28	60.79	56.62	58.63
STARD7	56.09	54.8	57.28	55.15	64.07	60.81	64.28	66.14
PPA1	50.29	49.18	54.41	53.67	65.48	60.93	76.89	65.96
MRPL36	52.08	55.06	55.61	55.39	61.13	60.97	56.07	58.49
NECTIN1	78.71	77.22	74.31	73.59	57.78	61.04	50.67	57.89
COA4	53.95	53.5	52.31	49.92	62.93	61.05	57.32	58.93
G3BP1	67.85	66.39	71.67	70.99	64.73	61.15	73.48	67.88
PRPF8	60.52	60.3	59.33	58.1	61.08	61.25	59.32	58.21
RNF187	73.56	71.7	69.36	63.88	58.55	61.27	46.86	52
DGCR6L	57.96	56.78	58	49.21	59.93	61.28	41.68	42.53
AGPAT1	65.05	65.49	64.85	59.65	62.8	61.4	54.55	62.5
DTD1	64.38	64.94	64.47	60.23	62.01	61.41	56.76	57.39
SMS	61.02	59.75	60.27	62.39	67.07	61.49	68.96	64.4
LRPPRC	46.3	39.56	54.22	58.69	63.23	61.54	93.59	81.7
SLC44A2	66.8	74.03	66.5	65.51	60.35	61.55	56.06	62.67
GSTO1	64.26	68.91	61.24	62.09	67.1	61.56	63.58	63.62
CHTF8	69.58	68.67	69.49	63.58	61.08	61.66	56.87	57.65
PSME3	59.24	58.31	57.3	58.4	64.24	61.67	61.85	60.51
UBE2H	60.34	60.31	64.88	66.99	62.88	61.74	71	67.8
MGMT	60.43	60.66	57.17	51.41	62.61	61.75	48.16	49.97
HMGB1	64.24	61.26	66.06	72.16	66.2	61.8	78.85	70.75
BUB3	49.06	47.09	54.17	56.53	62.5	61.83	74.89	67.45
MYL6B	66.16	72.4	64.47	62.86	64.6	61.84	50.6	59.29
SLC7A5	59.73	59.8	57.02	56.07	58.82	61.89	47.61	52.32
MRPL3	60.22	55.87	60.26	60.02	68.04	61.9	73.37	64.37
MTA2	64.25	65.16	61.62	59.84	62.87	61.94	55.18	58.49
TP53I13	71.98	70.26	66.27	59.22	63.03	61.95	43.99	50.37
ATP5J	58.34	57.96	61.14	56.7	63.82	61.99	63.54	65.93
CNBP	60.52	57.8	65.24	67.79	64.2	62.13	74.26	69.87
SERPINH1	115.82	118.94	109.04	105.34	57.91	62.16	49.34	54.83
CD99L2	60.81	64.2	58.12	57.66	59.65	62.2	51.81	58.53
MAGEA2B	53.34	59.89	52.94	53.58	61.35	62.29	60.04	67.03
IER5	56.75	56.15	52.85	52.74	57.88	62.33	48.5	49.04
OGDH	57.47	58.89	55.4	53.84	60.05	62.35	52.38	57.39
C17orf62	66.43	63.97	64.63	59.46	58.45	62.39	53.77	54.19
DPYSL3	60.3	59.54	60.06	59.63	62.09	62.45	64.29	61.34
TOMM34	51.09	51.21	54.34	48.99	66.27	62.51	59.96	60.98
TIMM17B	60.14	62.49	57.17	57.43	60.19	62.52	46.07	47.64
PHLDA3	50.22	52.14	49.65	47.07	60.44	62.54	47.62	50.38
CCM2	64.14	68.12	66.91	62.53	59.34	62.56	54.2	56.26
ATP1B1	60.71	58.59	65.56	70.35	65.52	62.67	79.15	74.72
TADA3	70.83	72.68	65.58	63.39	66.13	62.73	52.95	57.48
COA1	56.87	60.43	57.8	53.66	57.74	62.78	58.14	60.18
CTNNBIP1	66.51	66.23	64	60.4	61.18	62.87	52.37	55.43
PARP1	68.96	70.85	69.14	66.46	64.3	62.88	59.37	62.49
NDUFS3	54.2	54.52	52.41	51.13	65.68	63.02	55.47	61.11
PSMA4	63.15	63.91	63.77	65.13	67.94	63.08	79.78	75.18
CA12	67.75	66.56	71.97	69.79	61.08	63.1	67.12	64.94
SZRD1	62.36	65.1	62.15	60.93	65.69	63.17	57.36	56.98
TSR3	59.5	57.55	54.99	50.04	57.34	63.2	42.34	44.87
NDUFAF3	61.69	63.76	60.21	55.38	61.6	63.24	48.28	53.6
GRAMD1A	61.91	64.63	61.64	56.79	61.19	63.27	49.78	55.07
SOX9	78.39	80.96	78.89	78.78	57.65	63.28	55.17	62.9
SPARC	86.39	93.95	84.83	85.47	58.83	63.29	57.77	60.1
ACIN1	61.63	58.95	63.99	66.79	65.47	63.34	70.21	71.9
OXA1L	60.9	63.01	63.86	57.55	66.18	63.36	61.97	62.77
ANKRD65	64.68	66.68	65.68	59.47	60.48	63.39	53.58	56.77
CDC34	60.49	61.59	57.42	53.88	62.33	63.5	45.81	48.54
EIF2S3	60.95	60.27	67.38	70.65	68.64	63.54	87.52	76.44
TMX2	57.75	59.61	56.18	59.19	68.78	63.56	68.26	69.49
SNU13	59.75	63.2	58.76	60.32	67.12	63.62	63.28	60.69
MOGS	67.38	67.7	64.11	61.88	60.66	63.68	55.36	59.21
LAPTM4A	66.17	69.01	68.57	69.77	63.18	63.74	67.04	62.79
DNPH1	72.37	67.03	61.96	59.41	62.72	63.78	48.35	54.68
MANF	65.72	64.23	68.06	67.12	64.17	63.83	64.6	63.98
MANBAL	60.95	68.09	61.07	58.62	67.5	63.95	61.44	59.73
HDGFRP2	66.87	68.35	65.63	60.86	63.05	63.99	51.65	58.28
PON2	68.64	68.47	70.64	78.75	66.6	64.16	91.02	80.54
AP3D1	64.8	65.24	61.98	61.01	62.79	64.17	53.06	59.3
MET	56.03	49.45	66.64	72.06	67.14	64.31	90.78	77.74
TARS	56.74	52.13	60.64	64.87	65.49	64.35	83.64	74.79

SEPN1	73.27	73.12	71.45	67.93	63.62	64.38	56.06	60.42
MBTPS1	61.77	62.6	62.26	62.43	63.59	64.44	64.53	63.52
PPT1	56.52	56.48	55.84	58.19	68.13	64.49	74.57	67.61
SCARB1	52.79	56.92	54.66	52.7	58.3	64.53	53.27	58.79
P3H4	68.31	70	64.28	63.01	55.14	64.66	49.63	54.19
XYLT2	68.58	72.02	63.15	64.07	58.79	64.68	49.54	51.77
AHSA1	61.98	67.13	60.54	59.26	64.2	64.72	65.09	64.3
CDH2	56.98	59.86	61.7	65.67	65.98	64.74	76.01	71.93
DVL1	57.04	58.12	56.72	51.7	62.41	64.85	47.86	54.73
RNF5	66.42	68.86	62.03	59.53	68.29	64.97	52.9	59.3
VPS35	58.63	54.13	65	66.42	68.39	65.09	85.51	75.94
44454	58.86	60.78	62.61	67.07	65.58	65.21	78.1	70.07
NCLN	62.31	59.36	58.07	54.35	60.99	65.22	47.88	51.38
CHID1	66.86	71.85	64.05	61.43	62.83	65.22	52.21	58.88
MOV10	74.88	74.16	71.2	70.94	63.83	65.22	61.66	60.75
SERBP1	57.02	52.46	62.04	66.25	70.39	65.26	82.39	77.92
SSRP1	77.78	79.81	81.71	77.27	67.53	65.28	62.23	64.41
VDAC3	60.34	60.55	62.38	62.35	69.02	65.34	75.41	65.24
TWF2	67.53	68.5	64.5	59.29	61.89	65.38	50.01	56.19
FASTK	66.53	64.19	63.47	60.85	61.83	65.39	56.61	57.62
SMARCD2	65.59	68.98	64.79	62.42	63.67	65.41	55.77	58.54
PCSK1N	70.05	73.2	64.14	57.02	59.48	65.46	36.11	43.14
PSMC1	64.14	61.33	64.56	66.05	69.03	65.46	70.38	66.5
ODC1	59.74	57.15	55.77	55.78	69.94	65.48	65.99	62.45
DUS1L	61.18	60.26	57.24	54.25	63.9	65.5	54.14	55.54
DDX39A	58.55	58.85	59.5	51.71	68.22	65.74	57.03	56.28
IDS	63.81	58.23	66.55	66.46	65.02	65.74	71.31	68.24
LOC102724023	52.55	58.37	55.26	52.78	63.53	65.77	52.28	55.95
TRABD	63.59	61.91	58.96	56.49	62.63	65.78	53.8	54.45
TSPAN4	63.42	63.02	59.56	54.17	69.14	65.81	58.35	57.14
ZDHHC12	68.93	68.11	62.44	56.84	61.53	65.82	46.41	52.02
EZR	62.57	64.1	62.24	60.5	68.95	65.96	60.93	64.43
C19orf60	70.49	68.17	67.48	61.3	61.88	65.99	51	50.47
UQCR11	64.51	61.73	61.92	58.37	68.59	66.11	56.1	59.35
FAM89B	69.88	68	65.23	58.98	60.67	66.14	43.77	49.13
TRAF4	74.93	74.85	70.83	69.14	63.08	66.18	52.94	59.23
PTPRU	67.26	67.58	65.25	63.1	65.72	66.29	54.33	59.13
CYB5A	63.84	65.95	64.68	64.96	72.78	66.39	66.69	63.84
TRPC4AP	67.25	65.55	64.31	63.48	65.38	66.4	63.05	61.43
IARS2	57.79	54.35	61.93	65.27	71.15	66.41	85.32	77.31
CDIPT	62.33	64.81	62.68	58.67	63.68	66.6	50.96	57.25
SPCS1	62.59	66.42	64.89	63.69	72.36	66.64	66.64	62.82
VAMP3	61.79	61.96	64.88	64.71	68.48	66.69	77.03	69.74
EFNB1	65.37	68.72	63.79	59.39	66.08	66.74	56.03	60.87
HLA-C	85.19	89.1	82.33	77.99	69.71	66.76	57.17	57.69
GLRX3	61.82	61.79	61.79	62.22	68.54	66.76	72.05	72.42
ALDH4A1	64.05	66.53	61.27	58.03	67.28	66.83	52.26	60.36
HIF1A	71.13	69.44	82.08	91.29	67.57	66.88	101.03	92.87
NUBP2	59.83	63.07	57.52	50.44	60.69	66.93	45.39	48.28
MED10	58.61	55.9	60	57.35	73.52	66.94	70.95	64.01
NOB1	61.39	62.38	60.39	59.92	70.28	67.02	58.36	61.8
LAMTOR2	59.36	54.17	60.32	59.13	64.6	67.03	56.06	57.66
TAF15	67.78	70.48	71.12	70.06	72.88	67.07	74.93	75.5
CS	59.22	63.98	58.2	57.8	67.72	67.1	67.01	68.4
FADS1	51.97	56.86	55.54	57.26	66.7	67.11	67.17	72.68
TOMM5	68.06	60.97	58.44	58.12	72.32	67.17	74.13	67.49
SDHB	59.1	56.65	57.72	56.35	74.98	67.19	71	66.83
PITX1	80.4	80.41	74.08	66.6	57.01	67.22	40.73	50.74
EPHX1	68.08	74.73	67.39	64.23	61.43	67.24	55.1	62.81
DYNC1H1	61.93	62.82	61.99	63.87	63.98	67.25	66.77	69.25
UBE2E3	73.8	71.86	77.44	74.59	71.4	67.28	76.22	69.23
NUCKS1	65.19	61.44	72.6	78.43	74.41	67.33	92.15	82.38
TLN1	70.52	71.38	68.69	65.14	63.82	67.34	57.01	61.97
TOMM40	60.64	58.05	59.24	55.51	65.7	67.35	51.46	56.42
AGTRAP	64.92	67.84	62.18	59.33	62.34	67.36	53.33	57.12
ACTR2	67.01	62.12	71.49	79.61	72.06	67.43	98	84.05
EWSR1	70.29	67.5	70.57	69.18	72.65	67.5	70.23	69.67
NSA2	65.76	65.56	68.98	70.14	81.04	67.55	88.94	82.25
TOMM7	71.56	73.48	69.81	73.65	71.16	67.61	65.73	68.34
FEZ1	69	73.9	68.13	65.21	69.99	67.64	62.74	62.3
ELOF1	68.44	65.21	63.32	63.84	67.78	67.71	58.08	59.89
RBM38	61.05	64.74	61.72	58.66	65.89	67.89	51.15	59.37
CSNK1G2	67.18	70.43	67.11	62.4	66.67	67.92	54.38	57.13
NOP10	57.42	63.18	54.88	60.36	68.22	67.96	61.73	62.25
MTA1	62.06	64.52	61.38	58.79	64.36	67.99	53.26	57.85
LTBP3	80.1	80.46	73.14	72.12	58.18	68.04	47.43	52.78
SNRNP200	68.52	66.66	67.42	65.5	68.69	68.04	66.81	66.64
STRAP	57.52	58.69	60.47	63	70.54	68.08	74.49	77.12
LRRC59	73.3	71.32	69.84	64.69	69	68.13	58.79	65.41
MICALL1	59.16	61.69	57.87	54.7	67.39	68.15	55.5	60.24
EBP	61.81	64.63	58.22	59.48	62.87	68.16	54.43	59.58
MVB12A	65.25	67.07	63.17	58.08	66.73	68.19	50.04	56.3
MRPL34	59.77	64.4	60.27	55.5	70.71	68.22	55.52	64.77
MYDGF	64.79	70.36	62.8	57.53	64.61	68.29	49.13	54.25
C20orf24	64.67	63.42	62.66	61.7	68.06	68.33	60.54	58.39
C7orf50	66.27	73.55	65.17	57.59	65.07	68.37	49.52	51.84
SLC27A1	71.03	74.73	67.99	65.54	63.59	68.42	53.61	59.95

CPSF3L	67.51	66.17	65.37	61.77	68.52	68.49	55.04	58.18
SSBP4	72.46	68.26	63.89	61.26	62.99	68.63	48.12	53.83
COTL1	64.04	64.81	59.17	58.89	72.47	68.81	59.44	62.09
FIBP	72.8	68.82	66.08	61.66	71.4	68.89	60.58	63.04
GHITM	57.15	57.77	58.08	58.34	72.17	68.95	75.06	70.55
HNRNPD	77.14	77.81	77.26	75.71	74.42	68.96	69.81	70.71
HNRNPH1	71.26	62.57	77.61	84.45	71.99	68.99	102.75	85.11
HCFC1R1	61.6	63.61	59.54	58.67	70.77	69.09	50.47	65.05
IGSF3	65.09	67.71	66.19	65.73	66.27	69.24	64.88	68.77
KHSRP	72.29	72.46	66.99	67.39	67.41	69.32	57.87	59.9
INSIG1	54.25	59.17	57.95	67.31	62.86	69.34	69.26	73.24
HTRA1	71.51	74.47	65.85	66.99	61.63	69.43	58.59	62.53
ATIC	64.62	64.73	62.37	59.46	70.94	69.53	69.16	67.68
PDAP1	68.88	68.61	66.94	66.07	71.94	69.56	62.38	65.68
ADAR	93.06	94.88	93.62	95.84	67.83	69.65	72.8	69.88
NAPA	73.98	72.95	67.38	67.23	63.77	69.67	52.85	56.64
XRN2	67.08	64.42	73.58	71.02	73.46	69.67	85.46	78.15
TRIB2	69.07	72.12	72.69	78.35	67.14	69.68	77.32	76.72
PXN	75.74	75.38	74.02	73.48	67.31	69.69	61.33	66.08
TMED3	63.48	64.53	63.27	55.06	69.7	69.7	54.89	59.91
SERPINE1	74.1	77.89	72.54	76.64	69.26	69.75	70.56	70.62
SLC25A10	64.03	65.34	61.78	56.85	67.18	69.76	49.06	58.36
KIAA1191	64.35	69.45	66.05	62.79	68.98	69.78	65.25	67.6
BCL2L1	59.78	59.24	57.71	55.24	73.63	69.82	64.82	60.83
EIF3A	60.75	54.87	68.63	78.84	74.65	69.82	97.88	90.89
MAD2L2	73.38	76.23	68.62	65.54	74.8	69.85	53.39	56.72
ARL6IP1	61.81	56.81	66.4	68.36	73.13	69.85	93.6	86.67
COX7A2L	65.19	66.62	62.66	59.36	74.41	69.88	66.1	69.37
SLC50A1	65.31	70.43	68.77	65.46	69.06	69.92	65.5	73.64
CYP51A1	61.84	63.48	69.18	74.35	69.16	69.99	84.53	81.42
YIF1A	64.58	65.46	59.61	57.1	71.51	70	56.12	56.36
SNX5	58.7	58.4	65.88	68.95	71.13	70.01	92.41	77.61
CYGB	53.77	51.44	52.69	46.47	75.11	70.12	63.78	62.55
FAM234A	74.77	76.57	72.03	68.44	68.16	70.35	55.16	62.39
RCC2	66.54	71.3	72.15	70.44	71.83	70.35	66.04	67.91
AKT1S1	58.34	58.23	52.45	53.02	65.3	70.43	54.25	56.87
GPI	77.47	78.13	72.21	70.49	67.33	70.54	59	64.04
LSS	55.86	64.84	54.72	56.58	65.46	70.54	55.99	64.79
FAM83H	64.29	63.42	60.65	56.68	66.66	70.61	52.98	58.85
AUP1	68.84	64.16	63.34	62.08	73.44	70.67	63.91	67.11
CD46	64.16	63.57	70.66	86.35	74.82	70.71	119.74	105.69
BAG3	82.6	82	77	76.68	70.97	70.74	58.71	63.05
CDK4	74.27	75.87	74.16	69.17	70.82	70.83	60.42	62.67
LMF2	66.36	63.46	64.47	62.12	66.01	70.95	57.12	55.81
CARM1	75.91	76.04	70.98	67.72	71.6	70.95	59.71	63.69
COL4A5	75.1	77.9	78.85	85.66	65.76	70.97	82.79	81.52
C12orf57	83.82	83.82	81.85	75.16	69.39	70.99	55.18	63.18
REPIN1	69.28	70.02	67.19	66.27	67.26	71.02	56.65	64.29
ID3	73.81	76.31	71.88	65.84	65.27	71.03	56.21	57
GRK2	69.58	70.17	70.57	64.7	68.7	71.1	55.6	61.28
PSMB5	68.2	67.25	64.31	66.72	72.89	71.15	62.63	65.75
CCNL2	55.21	54.26	55.3	64.85	68.24	71.24	87.76	78.56
44261	65.38	61.38	71.52	78.6	71.02	71.26	93.22	84.89
KDELR2	73.42	76.03	76.68	73.03	73.53	71.28	74.95	74.77
DCTN3	74.54	74.24	69.15	65.08	69.81	71.34	58.81	61.05
ACO2	69.26	71.34	64.17	59.76	68.9	71.47	57.18	63.54
FAM3C	71.03	70.72	80.91	91.07	80.46	71.47	111.43	95.69
MPG	70.76	69.33	68.91	62.57	63.79	71.51	46.55	55.33
NCSTN	73.48	78.89	73.55	73.84	72.09	71.53	71.63	76
FDXR	63.94	65.29	60.25	55.73	76.11	71.65	55.84	61.15
VARS	65.91	66.94	61.42	57.52	66.73	71.73	53.8	56.62
GSN	87.92	88.96	83.48	79.66	68.15	71.78	57.68	63.19
PRAF2	66.93	73.1	69.04	66.04	66.55	71.8	56.89	64.58
ECI1	74.64	72.6	70.32	60.26	70.14	71.93	48.59	55.39
MTSS1L	80.55	79.54	76.73	74.75	71.93	72	57.96	69.83
HLA-B	87.36	92.47	90.01	77.46	73.02	72.01	62.05	60.49
NDUFB8	66.31	65.94	66.42	65.5	68.37	72.07	61.02	61.49
YIPF3	71.93	74.47	71.3	66.66	72.77	72.08	60.42	67.17
STAU1	73.42	71.97	75.98	75.28	75.34	72.13	76.1	76.11
ATP6V1E1	63.03	59.69	65.39	68.92	74.29	72.14	84.04	79.71
ERGIC1	73.7	80.08	74.27	73.58	73.83	72.16	66.15	69.68
NDUFA4	66.84	66.1	65.83	64.65	78.58	72.28	78.04	73.83
SKP1	77.49	75.37	78.86	78.35	78.4	72.48	86.25	84.31
ESD	69.71	73.52	73.39	69.29	70.07	72.49	82.08	77.68
BOP1	62.64	60.62	58.87	53.92	71.67	72.56	50.47	55.58
ATXN2L	76.19	74.99	74.17	72.47	69.79	72.63	64.01	68.54
TK1	87.49	82.21	78.4	72.51	74.7	72.68	53.19	54.86
HSPA4	67.62	61.71	74.42	76.7	77.4	72.73	89.41	84.82
HSD17B10	71.71	71.22	66.84	63.88	75.49	72.79	61.86	62.99
UFD1L	67.88	67.69	68.13	64.05	72.46	72.8	66.52	64.5
HGS	77.06	69.7	71.72	67	71.25	72.85	58.05	62
CSNK1E	71.62	73.91	73.63	73.73	73.57	72.96	69.95	69.84
PFKL	85.92	85.24	80.48	73.67	69.04	73.01	54.48	59.47
TPP1	76.8	82.02	79.84	80.97	74.71	73.03	75.98	80.48
TYMS	73.08	68.2	68.19	67.37	75.59	73.14	69.56	68.61
THBS1	81.13	76.44	84.16	89.26	71.98	73.15	80.64	77.74
PSMD2	72.78	73.41	71.33	69.14	71.32	73.16	63.46	66.97

PPM1G	69.15	70.27	67.39	65.61	75.5	73.19	68.61	69.56
IARS	67.63	64.46	69.01	68.26	75.53	73.28	86.74	79.96
PSMD13	67.48	72.6	68.72	65.98	72.74	73.38	65.03	63.86
UQCRQ	70.43	73.43	70.48	67.7	78.18	73.38	64.67	67.2
NDUFA7	70.58	73.36	64.45	70.26	66.8	73.43	61.89	62.59
PSMB1	76.71	76.64	80.7	75.96	78.5	73.43	80.64	77.7
LAMTOR5	71.1	73.47	73.24	74.11	79.59	73.51	85.62	80.21
HEXB	73.31	65.72	73.94	74.76	74.17	73.65	81	77.37
PRDX3	62.39	59.4	65.4	71.85	76.44	73.7	98.35	88.19
UFC1	55.69	58.61	61.33	60.19	76.79	73.73	74.29	73.11
TRAF7	74.3	70.78	69.26	67.99	73.23	73.79	61.38	66.69
CSAG3	65.63	70.09	70.93	66.97	74.11	73.8	75.9	75.18
MCAM	71.96	73.17	74.97	74.83	73.97	73.89	66.51	68.46
ARF4	77.49	78.95	79.82	81.84	77.9	73.89	82.56	83.64
SNF8	69.95	75.75	70.28	66.01	71.73	73.95	66.28	66.33
NDUFAB1	75.02	74.66	76.57	68.75	79.05	74.08	74.24	73.29
PODXL2	78.04	75.83	74.75	69.08	74.31	74.16	59.29	65.3
C14orf166	68.7	70.94	71.88	69.42	75.11	74.2	88.95	78.06
SF3B4	87.72	87.48	82.54	75.97	78.05	74.3	62.39	69.07
MAF1	78.16	80.03	74.57	66.85	76.73	74.31	58.36	63.98
SNRPG	70.54	81.39	76.44	79.89	76.69	74.38	81.25	85.57
SNX17	73.53	75.36	71.06	66.67	72.69	74.48	60.34	64.49
NDUFB5	65.17	65.7	69.55	71.25	78.52	74.5	86.84	76.64
KHDRBS1	80.43	75.16	82.1	78.55	79.27	74.62	82.43	75.58
TMED4	73.76	72.41	71.01	68.53	73.73	74.65	76.22	75.28
HOMER3	68.74	72.43	68.69	64.81	72.78	74.66	56.19	61.93
ARPC4	77.85	81.57	73.44	71.72	77.79	74.78	64.93	69.33
SEMA4B	98.24	95.45	92.18	87.91	67.36	74.83	60.3	67.57
FXR1	64.3	57.99	74.82	85.73	77.27	74.89	112.43	98.74
CEBPB	75.96	72.43	67.88	62.92	66.7	74.99	50.2	55.56
NOTCH1	80.75	77.47	76.51	75.16	66.85	75.08	59.59	64.16
FLII	74.15	74.1	69.78	69.35	72.57	75.12	61.86	65.89
GALNT2	77.3	79.91	74.74	76.75	75.55	75.24	66.68	71.14
IDH3B	70.99	70.61	72.08	64.21	75.24	75.34	66.09	67.52
CAV1	62.79	59.79	70.01	72.51	79.57	75.34	100.92	86.87
MRPS15	71.22	73.11	69.54	67.61	78.59	75.52	76.51	73.64
SF1	84.54	84.94	81.78	80.5	75.16	75.53	67.18	70.89
ERP29	73.44	80.04	74.78	73.03	83.9	75.62	69.94	79.78
RHBDD2	79.31	81.37	74.34	71.08	72.25	75.76	56.3	64.72
SNRNP70	79.61	82.2	79.15	76.65	76.87	75.84	60.86	70.95
DCTN1	76.53	78.56	73.76	70.5	75.46	75.85	64.95	69.74
CD320	73.91	73.41	70.98	64.36	73.84	75.91	55.74	63.49
RHOT2	63.85	62.07	60.71	57.17	70.16	76.03	66.33	66.95
USP22	76.58	76.86	75.47	73.3	75.69	76.07	70.23	74.46
MIEN1	73.7	76.02	70.82	72.26	74.63	76.08	64.5	68.22
DCTPP1	76.18	72.4	69.49	65.13	76.69	76.17	62.52	65.77
YIF1B	76.79	82.22	75.35	66.1	73.3	76.31	54.33	62.06
ADGRG1	79.27	80.01	76.86	77.68	73.02	76.31	67.53	73.67
SERPINE2	70.28	71.42	70.72	72.55	74.59	76.33	80.11	81.52
UXT	77.81	84.6	76.67	76.65	76.67	76.41	68.96	65.51
ERI3	79.91	76.04	74.05	68.06	80.6	76.51	68.05	69.02
DHPS	72.98	76.43	70.63	63.43	74.8	76.53	66.02	66.76
C1orf122	69.48	66.45	62.38	60.65	73.75	76.55	52.12	55.83
LOC107986035	88.28	89.06	84.77	74.12	54.14	76.65	48.57	53.66
MAPRE1	75.15	73.75	73.96	72.07	79.73	76.75	81.37	78.31
LRP5	90.75	93.55	85.79	84.1	70.88	76.97	58.73	66.69
DNAJC5	77.61	79.46	75.33	71.97	74.19	77.07	63.46	69.21
AKR1A1	80.4	89.2	79.05	75.19	79.26	77.14	69.27	72.33
CTSH	72.1	73.22	68.33	70.98	74.92	77.55	68.16	66.25
LDB1	75.53	77.73	74.18	74.75	76.94	77.55	69.38	73.01
CNP	96.33	102.06	94.32	89.93	75.13	77.66	64.73	70.99
MMP2	123.62	128.32	116.18	123.36	68.69	77.69	63.08	73.52
PTTG1	76.64	70.75	74.59	67.44	79.63	77.75	89.37	81.96
MVD	66.23	69.49	62.61	61.24	72.65	77.93	55.41	68.67
DPP7	78.6	77.87	74.86	64.9	76.97	77.97	61.37	66.82
STX10	69.35	69.07	68.04	64.08	78.17	78.02	66.77	69.91
SCRIB	67.12	65.88	62.53	58.78	73.32	78.07	58.52	64.57
SLC20A1	73.37	78.37	77.14	78.92	78.69	78.07	88.72	83.39
TAPBP	83.49	88.52	77.59	80.27	68.91	78.09	64.52	72.99
FKBP9	100.28	103.33	96.61	101.41	74.56	78.1	77	79.26
SARAF	70.61	70.02	74.49	82.64	77.88	78.11	96.59	89.58
ATP2A2	70.6	71.54	71.83	72.75	74.42	78.13	76.56	75.12
PFDN2	72.71	73.43	76.44	82.26	81.4	78.17	82.3	81.75
SCAMP3	73.9	76.5	72.39	69.17	76.69	78.28	64.36	68.85
FBLIM1	91.13	95.47	90.31	87.53	76.97	78.28	69.77	75.21
MAT2A	68.3	69.6	71.78	75.42	81.69	78.32	97.13	88.57
SERF2	77.64	84.13	73.92	73.5	77.44	78.33	66.42	66.29
MCRIP1	76.88	77.7	73.45	69.28	76.38	78.35	57.6	60.83
PSMA1	79.96	79.76	82.48	83.74	85.21	78.53	99.92	94.18
TNFRSF10B	67.33	69.13	69.35	71.3	80.88	78.54	84.1	84.18
APEH	74.42	75.51	70.5	66.54	75.4	78.74	65.54	67.18
BTBD2	84.97	84.41	79.37	74.06	71.29	78.75	55.77	64.24
CHD4	95.85	96.2	97.02	94.17	77.51	78.8	75.8	79.76
KIAA0040	74.18	75.9	75.28	75.87	78.17	78.89	81.21	84.3
HNRNPA0	82.75	81.09	79.31	77.28	78.71	78.91	60.84	70.61

TNFRSF1A	78.96	84.25	76.26	76.98	75.21	78.96	66.13	72.75
PPP2R1A	85.99	90.07	80.47	75.58	73.93	79.03	64.75	68.71
ARPC5	81.24	81.78	83.8	82.64	84.21	79.2	87.52	82.99
SRI	77.1	76.93	73.5	76.28	79.74	79.3	80.38	77.66
NEDD8	80.43	86.08	76.56	78.26	87.85	79.33	79.11	83.46
NDUFA3	86.06	86.92	88.21	76.44	86.68	79.44	64.06	73.51
AARS	89.95	94.71	89.06	86.07	82.14	79.45	73.2	76.9
GSS	65.16	69.11	63.62	62.49	81.79	79.51	75.45	71.56
EIF4A3	85.09	78.34	78.14	78.77	75.64	79.61	68.86	67.76
CKAP4	95.37	92.21	87.52	86.89	77.06	79.77	70.4	75.97
COPZ1	78.3	82.63	80.9	80.99	83.84	79.77	84.08	82.13
INF2	76.21	79.59	73.34	69.29	73.74	79.92	59.97	69.56
ATOX1	76.12	77.56	74.54	68.91	82.66	79.95	66.12	77.42
SEC11A	83.12	78.85	85.44	83.32	89.66	79.96	92.89	92.29
GBA	68.91	73.24	71.88	71.17	77.61	80.13	74.76	72.33
CDC42	86.87	85.49	87.58	90.99	86.38	80.17	96.43	89.29
NT5C3B	73.17	73.42	68.79	69.59	82.97	80.2	73.04	73.54
BRK1	83.36	80.22	84.33	84.76	82.65	80.22	82.79	83.31
PRKACA	84.03	86.65	83.37	81.96	82.92	80.34	72.22	80.96
POLR1D	79.52	79.25	78.08	75.81	85.31	80.38	84.13	82.05
ITPA	74.31	73.14	71.59	68.69	80.93	80.47	71.95	70.71
C16orf13	82.17	79.08	78.01	64.36	75.95	80.55	63.98	69.15
NME3	88.7	83.7	76.35	70.48	72.1	80.67	58.4	62.48
TPD52L2	77.84	76.26	75.51	71.62	79.92	80.73	72.98	75.21
ATRAID	89.52	96.88	83.41	83.11	81.98	80.76	70.56	69.9
CIB1	80.36	81.55	80.86	74.74	84.36	80.95	69.44	67.73
HK1	78.66	80.44	76.77	74.49	80.53	81.01	70.16	75.59
RBCK1	83.5	82.78	84.58	80.24	79.34	81.05	67.92	72.42
RBMX	83.21	77.98	81.91	90.29	86.11	81.05	101.37	88.56
NME4	92.13	95.86	90.16	83.18	79.38	81.21	62	74.31
DAZAP2	82.61	82.63	84.51	83.12	86.71	81.27	88.14	85.37
MRPL43	78.02	76.21	77.54	74.96	85.18	81.45	60.73	74
CTDSP2	85.42	87.53	86.52	87.67	83.38	81.53	82.6	83.91
NOMO2	82.25	93.08	80.28	90.31	79.41	81.58	77.99	81.12
VAT1	77.41	88.14	79.42	74.6	81.66	81.6	62.59	75.63
DAZAP1	80.1	76.55	75.97	73.09	77.32	81.61	69.72	68.1
UBXN6	81.39	79.91	76	72	78.69	81.62	61.51	69.31
NDUFS2	80.29	86.33	80.06	79.26	82.91	81.73	78.51	82.61
IFI30	81.76	79.34	79.54	77.47	81.25	81.74	65.82	71.18
DDX39B	86.93	82.11	83.41	81.56	86.04	81.74	87.59	82.52
LUZP6	76.73	67.33	81.34	87.22	91.49	81.98	111.03	97.94
AP2S1	78.49	89.64	78.83	74.28	80.7	82.05	65.93	67.43
F2R	83.75	74	91.42	97.78	89.71	82.17	113.21	92.35
CCNI	84.94	90.86	86.18	83.69	85.34	82.26	86.15	87.1
JUNB	79.09	82.4	73.91	70.06	75.43	82.33	57.09	63.84
LRRC8A	72.96	72.68	69.13	70.15	81.06	82.33	69.46	74.06
WBP1	80.74	84.08	76.15	74.15	80.01	82.49	66.92	70.56
ZG16B	86.44	107.75	94.09	84.71	77.28	82.5	60.99	76.1
EIF2S2	74.98	71.39	80.69	89.3	87.85	82.66	111.54	102.92
SOX2	80.28	77.01	80.39	81.44	78.02	82.73	72.83	79.98
ELOVL1	74.78	79.07	70.72	68.56	83.46	82.77	72.92	75.73
PSME1	97.54	99.77	99.43	85.4	90.96	82.81	79.94	84.08
PRKAR1A	77.8	79.52	92.73	99.87	85.58	82.86	109.6	99.36
C14orf2	79.22	82.04	77.28	78.8	84.75	83.01	80.52	85.41
CCT8	72.73	69.13	79.62	84.32	89.45	83.04	112.84	98.69
NRG1	71.69	66.93	72.17	77.15	81.78	83.14	88.28	85.15
MRPL20	79.85	87.41	80.07	74.76	83.16	83.29	73.44	72.37
SRP9	76.22	78.6	78.59	82.59	88.68	83.29	121.84	101.71
FN1	87.84	91.62	85.51	94.45	73.11	83.46	83	82.24
RBBP7	91.4	86.78	91.64	95.48	86.98	83.49	96.65	88.94
CALU	84.25	79.73	89.35	95.65	93.02	83.6	109.29	102.53
SEC61G	99.43	106.42	89.92	97.39	90.13	83.68	99.92	93.58
SH3GL1	94.49	95.88	90.93	84.9	82.85	83.73	64.09	71.64
MRPL28	83.13	87.45	85.68	78.95	82.57	83.74	64.74	68.47
PYCR1	92.9	92.62	83.59	78.42	85.1	83.75	66.75	72.78
ERH	82.6	84.96	88.74	97.99	89.25	83.83	94.76	103.46
NTPCR	76.18	77.83	74.32	71.95	89.74	83.89	78.39	82.21
MAP4	91.63	93.25	89.34	89.3	85.82	83.91	78.49	83.97
FXYD5	87.2	88.82	82.8	81.59	85.22	84.02	71.7	73.1
NSMCE1	88.05	83.71	84.78	79.52	88.42	84.03	76.93	79.22
UBAP2L	89	90.35	87.5	90.78	87.85	84.07	86.21	86.44
TXNDC5	83.22	77.23	81.2	79.16	86.89	84.1	83.08	81.33
NOC2L	78.22	74.27	74	68.3	83.93	84.23	66.83	71.77
44441	77.61	72.05	81.47	89.71	86.2	84.25	109.09	97.29
WASH1	81.6	73.67	77.22	77.79	86.11	84.33	80.27	82.68
HADHA	83.63	83.73	83.93	76.8	88.09	84.52	83.65	83.4
IRF2BP2	87.03	84.1	86.38	83.65	78.58	84.53	76.17	76.74
UBXN1	83.64	82.83	77.15	72.58	91.43	84.54	68.11	80.79
SLC1A5	87.64	90.98	84.98	80.3	82.88	84.58	68.01	73.81
TM7SF3	71.63	66.7	74.69	72.57	87.52	84.58	93.72	84.75
GOT2	72.06	78.44	75.48	68.86	86.09	84.67	84.84	78.71
SRSF7	80.11	75.55	77.37	83.97	87.28	84.75	106.11	92.55
TRAPPC5	82.74	82.89	75.98	68.25	76.12	84.94	51.23	62.75
PSMC3	83.02	80.19	80.55	77.81	88.8	85.22	76.81	78.66
PA2G4	78.41	77.12	78.03	72.74	90.71	85.27	82.11	85.18
PMEPA1	90.21	90.82	88.6	89.46	84.58	85.58	74.87	75.56
MAGED2	100.51	108.72	98.65	96	84.65	85.58	76.37	79.88
OGFR	98.51	97.98	92.24	89.08	86.18	85.64	69.83	71.62

CDK2AP2	100.49	99.72	94.81	86.25	84.69	85.7	65.64	79.1
SLC35B2	82.62	88.92	79.48	77.61	82.44	85.79	70.73	77.93
NOP56	78.87	79.95	81.33	76.61	94.28	85.91	89.91	87.32
PRNP	84.29	77.97	93.23	101	92.51	85.91	119.95	103.73
U2AF2	86.85	86.41	83.25	80.1	85.98	85.97	70.68	74.34
HNRNPF	86.2	84.68	88.67	85.44	93.49	86.02	89.92	86.94
NENF	81.12	88.04	73.49	75.23	81.95	86.03	63.83	65.91
TRAP1	80.64	77.76	79.46	72.43	86.47	86.09	75.72	75.2
FARSA	79.84	79.77	76.26	75.23	87.13	86.29	63.77	74.48
GAGE12G	67.24	69.19	62.71	63.86	82.91	86.3	72.3	66.58
VKORC1	93.72	94.82	90.81	84.63	81.76	86.37	67.45	71.33
RTFDC1	85.01	84.66	86.41	86.24	90.9	86.4	83.74	85.81
ERF	100.53	100.18	97.05	93.64	86.63	86.8	64.58	74.34
WASF2	103.49	102.7	100.09	97.88	86.93	86.8	81.42	86.9
PTTG1IP	88.7	88.58	89.19	83.07	86.09	86.88	81.29	85.66
MGAT1	94.39	96.64	92.8	87.67	82.27	87.03	66.85	75.14
CSNK1A1	82.47	75.08	89.9	92.89	87.88	87.25	104.35	94.05
NDUFS7	84.25	79.68	75.46	69.14	80.36	87.33	57.51	65.35
JTB	84.15	83.94	81.77	74.87	84.86	87.47	78.02	76.57
NAA10	75.91	74.23	72.57	67.68	85.53	87.53	64.37	73.96
PRR13	92.31	91.86	87.93	89.88	97.11	87.54	86.76	98.21
BGN	122.04	128.65	115.4	113.29	84.16	87.59	71.04	80.84
CAPN2	71.52	70.76	73.36	75.15	86.5	87.61	93.13	86.96
PRRC2A	89.05	91.72	87.83	88.23	84.1	87.75	75.2	81.98
CCT2	82.16	78.31	83.23	79.77	93.53	87.77	96.38	90.11
ABHD12	88.1	89.54	87.3	84.65	80.71	87.78	70.04	75.74
KPNA2	69.39	67.41	69.43	67.57	90.73	87.92	100.24	97.01
CCDC85B	77.13	73.11	69.04	65.07	73.65	87.96	52.64	60.82
MGST3	89.47	94.72	96.48	87.01	91.47	88.03	89.26	87.94
PGF	105.45	99.24	95.17	86.43	86.15	88.11	70.54	75.78
NELFCD	85.15	85.96	83.5	82.3	91.33	88.17	88.09	86.65
MRPS24	89.31	90.77	82.32	74.36	83.31	88.25	68.59	75.09
MAP7D1	92.38	90.31	88.1	81.29	82.62	88.38	64.99	73.35
HNRNPU	98.28	92.09	104.65	105.6	93.78	88.49	102.15	96.34
COPG1	90.67	94.66	88.51	84.83	88.49	88.72	78.14	81.9
ECE1	107.09	112.43	102.13	101.63	84.96	88.76	76.03	84.28
KDELR1	91.58	102.29	92.76	91.19	91.36	88.86	74.63	85.18
SLC3A2	84.38	86.3	83.13	79.52	87.22	88.87	72.68	80.02
SLX1A	99.62	103.61	92.65	82.08	83.79	88.89	57.9	67.47
SLX1B	99.62	103.61	92.65	82.08	83.79	88.89	57.9	67.47
F11R	86.06	90.94	86.04	83.79	92.26	88.95	87.84	91.47
TOMM6	78.81	78.16	77.83	83.82	86.06	89.18	89.48	88.45
ACTR1A	83.42	89.87	82.52	79.88	92.58	89.27	78.46	87.6
MRPS34	85.81	86.69	77.04	74.41	84.07	89.38	62.79	73.63
GALE	93.12	93.8	88.69	81.74	85.45	89.45	69.75	75.45
MRPL51	78.02	80.91	82.98	83.64	93.85	89.45	82.78	88.01
SMARCA4	88.98	87.93	84.16	84.42	83.4	89.53	73.84	78.23
DST	75.83	72.14	93.04	112.12	82.32	89.79	168.88	145.3
DUSP6	95.3	86.78	100.68	108	93.12	89.8	107.1	95.17
EIF4EBP1	86.85	89.18	82.66	79.54	96.6	89.84	66.39	76.24
RNPS1	90.98	90.44	89.48	87.72	90.75	89.88	77.54	80.91
GADD45A	73.62	68.01	75.31	75.96	92.25	89.97	84.62	86.37
AK2	91.87	88.68	88.04	86.32	87.08	90.15	89.57	85.45
CSNK1D	93.92	96.47	91.02	91.93	89.81	90.25	75.46	82.34
INO80E	81.3	77.87	77.96	80.24	88.83	90.39	77.3	80.35
UQCRFS1	80.13	76.17	77.63	75.34	87.32	90.45	79.85	84.35
JUND	90.45	95.97	84.86	82.75	79.39	90.65	53.47	67.71
MPST	82.79	82.21	81.84	69.03	87.19	90.66	64.64	70.89
TMEM9	91.78	97.03	89.94	82.95	87.79	90.78	75.23	79.13
NUCB1	95.34	100.23	91.55	89	88.26	90.81	73.47	81.44
VPS28	94.15	88.41	82.9	82.16	90.04	90.84	69.46	78.4
ATP1B3	95.66	99.11	94.22	96.04	95.23	91.06	99.69	96.54
NDUFB11	98.15	105.4	88.31	85.43	91.04	91.19	74.76	75.58
MRPL23	92.75	88.15	89.19	80.8	88.54	91.3	70.35	68.73
PXDN	103.24	104.88	104.13	106.53	89.15	91.3	88.13	93.04
TUBA4A	96.07	91.76	87.46	82.12	88.27	91.32	71.08	78.38
CBR1	84.76	83.48	85.54	81.39	85.17	91.48	72.92	80.39
GDI2	91.77	85.2	93.13	93.41	100.25	91.66	107.56	102.2
MAN2B1	94.28	100.45	90.06	87.68	87.31	91.81	71.06	80.54
UBE2I	92.26	104.37	97.93	91.01	91.93	91.93	87.25	90.82
SIGMAR1	79.52	79.79	79.48	75.39	92.17	92.11	76.72	82.04
CENPB	86.92	84.72	81.09	75.96	85.73	92.3	65.37	74.31
MFGE8	98.42	103.32	97.44	99.41	88.22	92.33	79.23	84.03
STUB1	90.36	91.86	84.03	79.46	89.05	92.34	69.46	78.33
TUBG1	95.31	88.72	83.18	82.59	92.49	92.35	76.79	78.36
TOMM20	81.37	77.43	88.93	93.21	96.25	92.36	117.15	103.19
SERINC3	87.71	87.2	90.54	96.17	94.78	92.37	110.65	105.83
C9orf16	98.28	99.78	89.68	83.19	94.81	92.43	67.29	74.65
C19orf53	89.63	94.83	84.33	80.51	94.28	92.48	76.01	81.87
FAM50A	84.43	87.59	85.03	82.45	96.09	92.63	83	88.98
CTTN	87.33	86.46	86.99	93.48	90.31	92.67	98.19	103.66
MORF4L2	96.55	94.31	102.11	106.59	96.89	92.77	116.03	107.54
TRAPPC1	89.3	100.73	83.13	91.14	86.14	92.92	77.62	82.58
ANXA5	92.17	92.41	93.69	89.32	95.21	92.98	96.5	91.84
PTHLH	102.69	96.4	103.8	100.27	100.8	93.02	101.98	90.99
RNPEP	87.92	90.15	85.74	82.6	91.84	93.07	78.87	85.66
B4GALT2	81.68	82.09	78.54	71.02	88.61	93.13	70.67	72.53
KARS	86.84	88.86	85.8	79.83	102.05	93.15	96.33	98.02
ARL6IP4	103.28	104.38	99.95	91.87	94.05	93.2	74.3	83.68

HAX1	98.16	101.96	95.44	89.4	93.06	93.36	92.09	89.39
SURF4	98.56	104.14	97.92	90.05	95.02	93.37	82.42	88.92
SMARCB1	101.93	102.47	96.39	91.24	93.79	93.38	76.01	84.4
SARS	92.45	93.71	90.66	89.45	99.9	93.38	90.12	91.27
BAX	73.74	78.77	74.82	70.28	93.88	93.45	75.73	78.07
HSPE1	98.06	95.65	96.76	99.22	97.12	93.55	104.71	100.77
GPRC5B	111.07	115.86	110.79	108.58	89.19	93.68	83.9	94.21
IRAK1	85.02	84.99	82.32	75.51	91.29	93.81	73.99	76.87
RRAGA	103.41	102.1	100.77	91.43	91.33	93.88	88.11	86.89
ECHS1	81.84	82.67	83.52	76.98	93.31	94.08	75.67	87.65
FST	40.79	43.5	45.2	48.75	93.39	94.16	103.82	96.85
NME1-NME2	85.1	95.78	83.1	71.67	74.36	94.24	51.85	61.14
UQCRC2	82.2	85.21	93.24	90.52	96.48	94.39	109.5	106.5
CAPRIN1	101.13	90.49	103.07	102.62	98.67	94.47	112.15	102.47
POMGNT1	90	95.3	89.73	84.65	93.31	94.67	80.39	84.69
MAGEA12	97.41	89.27	90.08	89.11	102.79	94.67	87.49	94.15
SF3A2	107.56	102.2	95.38	91.87	95.67	94.68	71.7	77.74
ATP5C1	86.79	88.15	89.78	88.62	99.69	94.79	105.58	97.75
PRPF6	94.86	99.72	95.45	89.11	96.82	94.82	80.4	85.78
EIF3D	87.27	88.57	88.25	83.48	102.45	94.92	86.64	92.83
BCAM	135.61	127.91	122.91	117.7	95.53	95.14	74.65	82.31
ATP5F1	85.46	85.31	86.77	88.45	100.29	95.41	110.14	101.95
NR2F6	96.95	94.13	88.14	82.29	82.35	95.5	63.17	71.51
CARD19	95.87	100.27	92.76	81.07	89.48	95.52	68.68	75.66
ITM2C	103.61	110.44	100.91	95.07	94.17	95.53	78.66	87.83
CD99	99.87	106.16	97.61	96.17	96.06	95.58	79.48	86.9
NDUFA1	98.8	98.39	93.64	98.74	93.41	95.58	88.67	95.65
DBI	78.92	85.06	79.9	86.39	95.48	95.58	98.4	106.13
ZYX	108.81	111.94	102.99	97.24	93.43	95.69	71.98	87.25
ENSA	93.33	99.45	95.8	97.42	105.51	95.71	95.46	91.76
UBE2C	83.04	80.24	83.83	74.49	101.09	95.82	93.26	84.51
MT2A	234.38	201.38	198.06	193.22	108.3	95.94	86.7	82.04
AKT1	99.6	100.54	95.34	90.97	92.8	95.96	75.49	83.35
RNF181	103.79	113.91	103.91	99.01	98.49	96.01	88.15	86.8
PFN2	108.83	107.48	104.29	109.37	105.79	96.12	110.74	102.87
ADIPOR1	86.37	94.74	88.16	87.09	100.33	96.73	93.28	93.8
RNASEH2A	102.36	99.32	98.57	91.3	97.31	96.8	75.8	80.54
DCXR	96.89	98.13	89.12	79.43	91.66	96.83	72.82	71.96
LSM7	102.22	88.84	97.78	97.69	103.9	96.88	81.13	79
MRFAP1	95.21	96.66	98.91	99.89	101.15	96.89	96.42	100.16
APH1A	97.44	105.66	98.39	92.88	93.6	96.99	81.81	89.86
CTSA	96	103.46	95.95	97.73	98.86	97.1	85.42	92.73
TMEM259	90.82	90.32	87.56	86.11	92.39	97.11	80.45	84.01
MRPL24	92.99	95.97	87.77	81.81	101.43	97.2	83.34	83.36
EPN1	104.16	106.21	103.49	94.94	97.42	97.72	69	81.86
RFXANK	96.98	99.23	90.97	87.01	94.54	97.84	74.61	82.03
TMEM59	100.74	93.84	100.47	101.29	102.92	97.87	110.15	103.7
CIRBP	102.05	101.96	105.06	107.41	103.09	97.92	102.23	103.88
NDUFB10	96.52	101.75	95.95	93.71	97.61	97.93	84.13	90.32
KRTCAP2	92.64	102.28	102.73	96.77	97.46	98	83.99	90.6
PPP1R35	94.45	94.74	91.7	90.71	91.46	98.03	71.18	82.54
MAGEA3	93.87	91.66	93.52	92.25	100.38	98.09	89.36	95.09
COPA	102.46	100.9	100.72	101.34	99.31	98.2	101.59	99.61
POLR2J	94.94	96.9	86.61	91.71	97.83	98.21	87.29	95.58
MDH1	93.38	94.67	100.47	98.98	105.21	98.26	119.55	117.14
NAP1L4	95.07	93.18	99.27	97.42	100.49	98.38	97.23	97.08
C17orf89	93.82	86.76	87.4	80.38	102.4	98.46	78.84	86.98
RAB11B	116.25	109.53	108.18	103.82	92.65	98.52	74.88	80.53
MAGEA6	93.77	93.47	90.5	87.96	101.71	98.57	90.3	99.23
RANGAP1	88.96	87.92	84.16	78.74	99.26	98.59	79.51	86.73
ST13	98.18	95.45	101.01	101.69	104.32	98.64	116.53	109.1
FIS1	87.88	84.52	85.81	80.25	95.34	98.72	77.64	78.73
TUBB6	87.72	85.2	82.49	80.35	100.17	98.78	82.02	83.32
PHLDA1	98.14	97.66	99.49	101.56	95.74	99.08	93.65	103.05
TMEM230	92.89	94.02	93.74	93.45	102.33	99.09	108.37	104.04
ICAM1	98.83	106.86	98.62	95.61	98	99.4	91.36	96.36
CSE1L	84.67	74.24	89.69	95.58	104.08	99.78	145.83	124.52
EFHD2	107.41	110.3	103.48	92.85	100.54	99.79	78.45	82.23
AP2A1	95.96	95.35	90.21	83.94	96.91	99.79	74.82	84.7
CYB5R3	99.48	99.62	97.77	93.13	96.91	99.8	77.95	84.67
ILF2	101.49	102.02	102.24	98.41	103.68	100.37	101.24	97.58
MRPL37	92.56	96.9	86.42	86.11	95.45	100.39	84.27	88.33
TPM4	111.75	102.8	112.29	114.18	105.19	100.53	119.12	107.76
CLTC	97.4	87.64	106.69	108.51	105.55	100.53	127.85	116.46
U2AF1	102.77	107.19	108.18	106.81	102.91	100.56	90.61	90.1
AMZ2	87.49	89.13	85.86	90.97	101.19	100.7	103.72	99.89
SF3B2	103.78	104.74	102.2	95.2	104.26	101.04	89.93	95.87
CSNK2B	104.71	106.6	102.54	95.75	111.62	101.12	94.9	99.39
SDHA	95.67	92.27	94.45	91.41	100.48	101.53	96.32	97.03
FUS	117.74	111.3	116.01	112.18	110.47	101.57	100.38	96.02
SEC61B	98.22	98.25	97.72	95.43	105.43	101.67	97.97	90.72
ATP5I	108.09	105.51	102.8	103.41	108.71	101.77	96.21	101.07
PTOV1	115.84	119.47	107.58	100	96.11	101.85	79.53	83.25
PGD	92.26	100.15	90.67	87.18	98.94	101.95	87.44	95.77
POLR2G	103.36	105.78	103.9	97.59	112.39	101.96	99.01	97.3
SF3B5	95.21	97.76	91.29	91.16	100.49	101.98	81.7	87.19

PTRF	110	115.23	105.7	102.35	101.26	102.22	82.02	92.68
TMED2	100.92	97.91	103.56	107.13	108.01	102.22	119.88	108.82
LASP1	118.06	118.56	116.07	109.84	100.42	102.26	85.77	93.37
RAC2	77.82	87.09	81.87	76.85	108.52	102.31	90.62	96.13
UBE2V1	94.77	103.98	105.58	100.82	111.52	102.34	115.12	119.06
UBE2M	101.35	107.29	97.35	94.52	102.47	102.4	81.45	86.86
STMN3	111.37	107.59	101.31	95.05	102.48	102.51	80.12	88.88
IFI27L2	114.78	112.57	107.46	94.33	92.28	102.76	76.14	79.46
COL7A1	90.87	94.58	93.13	99.36	94.21	102.79	111.63	111.42
DAP3	92.24	90.45	89.5	83.42	111.89	103.05	109.09	102.02
MTCH1	99.67	99.66	98.66	90.87	92.32	103.24	80.35	90.06
DNAJB6	104.37	106.65	102.54	105.89	110.86	103.43	117.34	110.44
RAN	94.61	96.97	95.66	97.96	105.82	103.51	108.96	101.87
NIPSNAP1	98.26	106.59	98.63	95.24	105.23	103.76	93.88	101.65
CSAG1	96.85	103.57	96.78	96.49	106.02	103.83	91.56	107.57
PEBP1	95.09	91.12	92.27	87.69	107.71	103.9	95.19	94.83
SLC6A8	144.02	131.08	130.06	124.51	101.34	104.02	90.81	94.69
RAB1B	106.3	112.85	105.34	100.28	104.89	104.1	79.79	96.14
FAM127A	108.37	109.38	105.19	99.09	97.63	104.22	79.58	85.62
ATP6V0E1	114.1	119.34	114.13	111.73	116.07	104.34	114.15	114.45
HMGN1	107.95	110.16	114.56	108.92	105.87	104.46	123.31	116.46
ZNHIT1	109.5	104.59	100.11	97.56	103.03	104.5	83.4	85.6
PRDX4	110.51	109.29	103.06	100.26	103.6	104.59	99.1	98.73
GNG5	119.21	121.73	110.54	108.63	110.01	104.97	102.66	95.94
GPS1	96.12	92.89	89.81	82.92	102.25	105.01	80.78	84.07
PDCD6	108	99.76	99.7	102.26	106.8	105.01	105.57	103.46
EMC10	108.89	108.35	105.77	97.73	100.72	105.11	76.62	89.79
ATP6V0B	93.17	99.45	88.83	88.1	109.23	105.18	87.43	92.26
SET	102.15	91.59	100.89	102.17	110.17	105.22	116.1	110.72
SSU72	103.91	105.83	97.88	97.01	103.35	105.56	89.85	89.41
MAGED1	131.69	136.39	128.72	122.96	102.15	105.82	89.71	99.96
SFPQ	114.86	101.94	108.15	110.58	103.94	105.96	98.67	97.02
GPR153	77.74	77.98	75.29	71.23	96.69	106.09	79.29	89.73
CLDN1	113.45	106.35	123.74	129.51	120.74	106.1	140.69	126.01
PIN1	100.95	102.07	95.95	87.62	102.74	106.19	80.16	82.61
GSTK1	102.18	104.67	101	98.98	112.06	106.62	98.32	103.96
AHNAK	107.99	107.36	110.83	115.04	100.88	106.67	118.03	115.44
CTNNA1	117.27	119.06	118.61	114.6	109.34	106.69	110.47	105.38
XRCC5	107.11	103.7	111.78	109.34	115.37	106.72	126.72	114.56
CLPTM1L	101.04	104.26	95.98	95.56	106.79	106.77	96.48	103.71
C20orf27	89.61	96.23	90.05	84.38	104.12	107.04	82.41	89.87
SSBP1	103.37	110.46	103.14	106.81	116.01	107.04	116.94	117.99
SLC39A1	99.64	103.92	98.44	93.54	111.01	107.18	92.26	96.87
CORO1B	106.79	106.68	97.98	90.49	98.23	107.33	74.97	82.52
FAM210B	109.8	109.38	114.09	117.11	109.36	107.39	121.82	113.81
PAFAH1B3	114.86	108.98	107.11	99.95	100.24	107.64	79.62	89.96
SLC2A1	127.48	126.44	117.65	113.06	102.88	107.7	88.84	94.82
PHB	91.73	98.17	96.49	82.4	111.89	107.76	94.55	97.61
BUD31	105.75	107.59	114.59	102.85	109.04	107.76	107.44	103.87
PERP	97.16	96.22	107.87	109.91	112.72	108.4	128.61	119.7
ASNA1	99.73	104.57	96.3	91.26	108.44	108.48	83.21	92.58
KXD1	113.34	109.71	104.15	101.81	113.39	108.75	94.08	98.58
TUBA1A	112.72	123.81	112.69	108.32	106.33	108.81	92.17	107.17
TMEM258	117.92	114.66	113.04	104.72	105.47	108.98	90.98	105.03
GM2A	91.45	97.24	93.07	94.13	109.91	108.99	105.61	112.97
ATP6AP1	103.3	106.37	98.55	99.27	101.54	109.11	91.63	99.9
LAMP1	103.65	109.68	100.64	100.37	109.99	109.31	101.88	102.49
WBSCR22	106.28	112.19	106.76	103.74	114.46	109.49	100.58	105.58
ATP5L	107.57	116.19	106.87	103.82	111.36	109.5	98.52	107.12
ANP32B	112.96	106.94	109.11	115.17	118.92	109.56	113.53	117.61
NAA20	89.44	85.39	95.37	98.96	112.5	110.01	129.43	121.97
HSPA9	104.27	99.27	107.9	103.46	115.96	110.09	130.83	124.08
MAZ	108.07	108.05	105.58	96.78	106.93	110.17	82.32	92.67
PSMA6	114.79	119.88	118.14	113.22	120.64	110.56	121.94	115.16
LAMA5	108.82	102.3	103.8	104.25	100.7	110.71	94.57	97.75
CHMP2A	114.65	117.15	112.43	108.48	115.9	110.82	95.49	100.48
GLUL	91.58	103.15	92.73	89.19	111.91	110.87	100.81	113.04
CRABP2	160.54	184.75	156.38	153.39	111.96	111.1	82.7	111.47
NDUFV1	104.12	102.74	94.39	91.39	108.11	111.15	90.09	94.99
ALYREF	112.99	109.45	99.36	94.57	102.32	111.2	84.44	79.1
PSMC5	110.42	110.39	108.98	104.71	113.85	111.29	114.17	107.32
RRBP1	127.28	126.11	129.37	129.44	104.73	111.38	102.02	109.33
ZFP36L2	84.04	84.79	88.44	87.96	110.17	111.66	101.52	105.53
TIMP1	125.2	129.02	116.2	111.52	109.84	111.79	91.86	94.72
B3GAT3	108.53	107.33	106.17	97.62	104.84	112.25	83.75	95.02
CREG1	109.56	105.96	118.95	124.19	109.47	112.27	133.54	126.83
SRSF9	117.01	119.91	115.78	110.17	112.7	112.35	95.59	103.14
PSMB7	112.82	116.11	108.23	109.3	121.92	112.36	116.68	117.49
NDUFS8	105.77	110.1	106.79	85.8	105.88	112.39	77.84	85.52
IFI27	422.94	442.73	431.33	363.69	120.63	112.56	101.19	78.65
PCNA	114.62	104.46	115.79	120.85	118.67	112.62	124.58	112.09
ITGA6	109.33	102.14	116.73	130.64	118.15	113.12	150.46	137.97
PPP4C	113.99	114.17	106.92	101.62	107.95	113.25	83.68	88.68
RNASEK	103.68	109.74	102.96	101.72	116.49	113.62	102.77	104.26
SLC4A2	121.29	122.07	115.73	106.59	109.68	113.77	89.07	97.75
NFE2L1	115.53	119.34	115.24	114.14	114.3	113.81	108.97	118.02
MVP	126.65	133.36	120.49	114.04	108.54	113.82	88.37	99.15

PUF60	120.62	118.52	116.03	106.25	110.67	113.87	89.89	98.52
NT5DC2	97.25	94.39	90.77	87.5	105.88	114.08	80.76	93.14
STOML2	115.11	118.26	115.42	104.33	115.54	114.4	99.92	105.76
PSMD8	111.96	113.45	109.15	98.96	114.39	114.83	99.75	100.81
EIF4A2	120.6	122.36	128.49	138.75	115.3	114.88	156.46	145.14
LAPTM4B	112.13	109.77	114.23	110.73	116.11	115.28	120.29	120.74
QSOX1	106.18	108.99	106.34	103.69	114.69	115.8	98.82	103.88
CERS2	109.33	111.94	107.56	104.22	116.38	115.86	110.59	113.32
PHPT1	108.85	112.5	105.06	102.73	119.62	116.07	91.61	99.6
GADD45GIP1	123.29	111.59	111.08	106.67	107.46	116.25	84.35	86.65
DHCR7	91.93	106.62	91.41	92	108.97	116.28	92.64	107.47
C1QBP	106	105.54	102.39	98.65	123.81	116.45	111.3	109.92
FLOT2	128.81	131.35	122.41	116.48	115.71	116.47	100.81	108.18
CDK2AP1	113.1	121.25	117.12	121.52	113.07	116.49	121.2	121.05
RAB7A	109.09	115.07	119.13	120.82	121.77	116.76	121.4	131.72
PRELID1	112.27	113.27	104.84	100.29	117.18	116.83	90.16	98.46
TMUB1	115.81	116.01	106.22	100.28	115.43	117.1	81.01	92.1
COL18A1	121.96	123.13	117.2	113.28	109.51	117.3	92.83	98.11
HDAC1	126.91	129.2	127.94	120.19	120.75	117.53	114.67	112.2
GRHPR	114.09	123.82	109.95	104.67	119.3	117.62	91.23	99.6
USMG5	103.55	114.9	103.1	101.66	124.19	117.62	135.13	113.59
COL4A2	125.68	132.84	123.49	124.76	110.64	117.68	102	107.75
GPAA1	117.46	118.99	112.7	100.64	110.92	117.71	86.18	96.92
ANAPC11	114.72	113.52	108.33	99.33	109.92	117.77	85.16	94.14
PSMD3	115.44	115.8	110.79	101.81	112.87	117.77	92.51	99.62
TBCB	119.3	125.11	113.55	106.11	115.4	117.95	99.02	99.69
ASPH	98.42	92.74	110.1	136.08	121.97	117.99	198.26	172.93
SRP14	114.59	119.53	121.22	119.09	124.5	118.12	125.95	125.49
NINJ1	100.34	101.97	96.03	90.35	116.42	118.33	97.16	102.5
ARHGAP23	140.06	138.27	135.33	134.28	113	118.37	97.14	103.54
DDB1	105.05	108.94	103.56	98.94	121.17	118.45	108.5	111.08
NDUFA8	121.35	124.42	115.51	114.21	124.7	118.55	111.86	122.25
RPL36AL	125.76	130.94	130.55	136.45	125.37	118.62	141.31	148.27
BASP1	148.13	148.98	146.03	142.04	117.24	118.81	105.84	113.24
PLOD3	121.53	128.32	114.77	113.08	111.06	119.11	90.74	103.02
LIPA	106.6	107.92	106.75	108.94	128.12	119.14	139.88	125.7
PGLS	112.42	110.79	107.21	96.76	111.28	119.75	88.3	97.43
CAST	124.36	117.94	128.92	134.38	123.33	119.79	143.46	137.05
COMT	112.15	110.86	108.79	103.23	113.59	119.81	91.54	98
WDR83OS	123.92	118.52	112.67	106.54	116.51	120.02	105.54	101.56
TMEM132A	122.69	125.19	116.01	109.62	108.64	120.33	84.61	99.12
POLD2	127.16	129.95	115.35	113.52	112.55	120.4	89.06	101.46
IFITM1	396.81	417.22	373.63	390.13	124.03	120.43	115.63	106.84
TSPAN3	103.54	107.25	112.94	115.03	127.36	120.61	148.58	141.65
CAPZB	131.51	140.62	122.35	123.97	126.26	120.77	108.47	118.17
SYPL1	104.41	106.66	111.19	115.65	125.34	120.98	158.36	149.41
NOMO1	114.06	120.02	116.43	113.99	115.33	121.16	118.18	120.66
SNRPD2	127.58	134.81	118.26	112.79	126.98	121.4	112.61	111.89
CSTB	106.38	112.73	111.35	110.17	120.34	121.42	107.23	104.21
CKS1B	117.99	125.12	122.47	119.89	126.52	121.47	117.88	118.12
MYC	90.76	87.99	89.47	86.58	131.45	121.55	112.35	113.72
OS9	127.88	132.49	126.94	128.41	121.46	121.63	110.51	118.22
LYPLA2	122.87	125.55	118.38	112.59	126.49	121.71	103.42	111.84
LSM4	109.39	111.24	105.64	96.11	115.77	121.97	95.19	93.51
DBN1	130.07	132.83	125.36	117.98	119.09	122.1	94.25	112.32
VCP	122.41	124.73	117.08	115.57	124.59	122.22	115.5	119.31
ENTPD6	113.88	109.4	106.14	105.67	118.62	122.58	106.95	108.28
DDX5	112.55	106.37	121.35	133.56	128.69	122.84	178.05	153.31
LAMB3	131.54	130.84	127.63	124.58	117.97	123.32	103.67	108.04
WDR1	122.26	125.29	119.48	116.47	120.73	123.51	103.2	109.13
SLC9A3R1	122	132.58	119.69	115.33	115.93	123.95	89.56	104.87
GAMT	134.24	133.68	124.63	112.7	118.58	124.02	82.37	95.41
BLCAP	125.43	130.65	123.45	122.1	126.67	124.11	120.71	125.17
DAP	123.61	128.43	120.75	115.48	119.97	124.16	109.56	115.71
SREBF2	118.77	124	118.9	115.11	120.96	124.59	107.66	115.77
PLOD1	152.7	165.71	149.66	143.93	114.85	124.74	101.67	111.38
HRAS	129.1	127.53	121.89	112.26	132.5	124.87	97.28	103.49
NDUFA2	127.21	134.19	120.38	118.78	123.34	125.01	104.93	115.94
MRPL12	131.11	137.59	126.15	110.51	121.3	125.02	92.11	101.33
PFKP	157.49	154.12	146.01	133.4	121.02	125.07	104.71	105.72
YWHAG	125.17	126.88	125.75	125.93	131.86	125.12	132.16	129.46
LMAN2	130.69	132.59	123.11	117.04	113.73	125.27	95.37	100.19
APMAP	123.45	123.32	118.71	118.63	124.94	126.1	115.37	120.66
IMPDH1	121.46	116.69	114.48	107.96	122.26	126.2	100.84	108.51
CAPN1	120.46	121.63	113.84	106.91	123.71	126.48	97.97	107.52
CUTA	127.22	134.58	121.23	115.44	121.49	126.6	99.21	108.98
PRDX6	123.42	126.55	125.32	116.29	132.87	126.69	134	125.22
DAD1	132.59	136.1	138.24	129.94	131.32	126.84	123.13	124.98
EIF3M	109.86	108.89	118.76	113.93	132.66	126.86	156.08	137.7
DNAJB1	132.94	137.53	131.05	129.08	123.51	127	114.22	116.91
PLCD3	110.06	112.21	105.48	101.95	125.58	127.02	98.46	111.82
GIT1	114.11	112.8	112.69	107.19	121.16	127.46	100.55	109.19
SLC25A39	119.73	120.64	113.46	104.79	121.5	127.47	96.01	110.41
HN1	131.4	141.67	129.29	124.12	132.13	127.53	115.04	121.41
S100A16	128.49	130.91	130.5	120.41	128.14	127.99	107.97	109.41

RAC1	135.98	134.94	133.37	129.36	131.19	128.04	130.69	127.14
HNRNPM	140.4	127.15	136.3	134.37	133.23	128.06	120.03	119.86
CTSC	127.36	120.75	131.67	135.82	132.33	128.16	146.8	137.7
KPNB1	122.82	116.3	124.94	127.88	134.7	128.19	148.02	142.01
GDI1	131.23	135.96	130.32	126.07	129.2	128.35	117.13	124.66
RPN1	131.17	133.06	133.1	127.34	125.46	128.63	118.54	120.77
MBOAT7	126.22	134.24	121.67	121.31	116.36	129.15	94.54	109.12
ANXA1	111.84	113.72	121.31	130.74	131.6	129.23	175.97	154.67
HSF1	125.4	129.25	120.06	116.01	119.63	129.29	92.41	108.86
MXRA7	144.05	138.89	140.94	130.82	130.42	129.55	121.12	120.06
HNRNPAB	124.51	123.23	126.91	122.44	136.04	130.07	122.06	121.99
PLTP	134.33	137.14	126.1	123.18	126.29	130.12	106.98	117.9
CDC25B	85.98	89.99	84.14	83.25	125.53	130.19	114.54	125.15
NUDC	141.06	135.74	137.19	125.55	137.01	130.33	115.93	127.48
COX6C	121.78	121.24	124.71	136.76	134.8	130.47	149.98	151.12
RABAC1	137.09	140.13	134.38	114.6	127.49	131.6	92.86	112.16
G6PC3	130.71	133.44	120.81	123.51	138.65	131.61	110.09	121.27
HSPA5	162.95	160.01	160.4	165.75	136.46	132.47	148.01	141.81
MORF4L1	137.55	141.34	147.4	149.78	150.98	132.66	161.99	158.51
EIF3B	120.81	123.32	117.49	114.69	135.02	133.39	116.99	121.51
PROCR	115.63	122.3	109.07	106.85	128.66	133.6	113.17	117.22
SCAND1	136.17	135.17	128.35	111.25	120.12	134.05	88.66	97.55
CCDC124	124.16	127.36	121.81	110.96	135.42	134.41	102.56	109.21
SHFM1	144.32	136.54	142.15	155.74	136.12	135.43	158.85	156.05
TSEN34	137.49	136.38	133.44	119.19	130.93	135.54	112.65	117.55
FBLN1	159.08	160.18	150.5	142.1	127.56	135.6	106.41	116.45
COL6A2	152.95	156.1	141.09	141.95	124.94	136.25	104.15	111.32
COX7A2	129.82	141	131.84	119.25	139.77	136.45	124.73	128.02
OST4	140.11	141.48	127.59	136.32	135.22	136.68	116.26	135.48
YWHAB	139.61	132.43	140.67	134.66	139.98	137.57	144.03	145.8
GIPC1	133.58	136.58	125.25	116.96	132.05	137.7	94.14	119.84
SEC61A1	131.53	135.46	128.18	128.13	140.47	137.8	131.7	133.55
GAA	153.16	157.66	142.56	140.51	128.51	138.03	109.31	119.93
PDIA3	154.2	161.03	156.83	158	144.21	138.35	151.56	148.55
TCP1	121.22	116.06	127.01	126.18	145.59	138.35	158.29	148.7
ARPC3	131.84	128.98	134.86	138.31	142.11	138.72	162.16	150.23
QARS	132.54	127.72	127.49	121.53	146.16	139.28	127.89	132.08
PIGT	136.02	140.34	134.03	130.31	137.73	139.41	113.77	125.82
GABARAP	138.89	150.05	134.99	128.28	149.07	139.46	131.7	135.24
TRIM28	171.23	167.61	161.07	147.39	128.41	139.52	102.98	111.4
MRPL4	132.51	134.08	122.62	114.86	140.88	139.77	101.36	115.19
PFDN5	147.18	164.82	146.97	134.86	146.65	139.8	136.79	137.34
HM13	135.97	146.75	132.79	128.5	138.65	139.87	120.81	134.84
MYL12A	149.37	148.91	155.42	152.75	150.88	139.93	169.61	160.85
H2AFZ	144.04	144.94	143.37	130.67	148.95	140	139.25	140.31
LRP10	138.12	141.46	134.63	129.01	132.85	140.93	109.74	125.24
ECH1	152.22	159.09	145.68	133.58	135.94	140.96	108.47	114.92
PHGDH	179.68	181.66	164.86	150.56	146.36	141.1	118.33	125.3
COPS6	134.88	140.34	131.58	123.58	138.74	141.12	119.7	122.2
GTF2I	134.46	138.07	138.23	145.78	141.48	141.14	173.64	162.68
EPS8L2	128.96	130.31	123.45	117.07	138.51	142.3	121.82	128.27
COX7B	137.01	133.04	135.57	140.26	140.37	142.5	174.49	152.52
PTMS	168.51	178.7	167.02	166.58	133.01	143.09	93.13	130.41
PLXNA1	146.36	139.74	142.03	144.8	142.77	143.17	127.36	130.03
CCT4	139.94	140.23	139.68	138.69	149.12	143.49	149.22	149.2
RAB13	134.03	137.35	137.39	141.17	144.68	143.82	141.1	143.38
PTGES3	143.98	141.14	142.07	142.48	151.59	143.84	167.71	158.62
TALDO1	140.85	142.44	134.34	128.06	141.22	144.53	118.5	127.67
RANBP1	143.68	143.17	145.31	139.09	151.28	144.59	142.16	136.24
MYH9	159.53	158.08	153.64	148.57	146.82	144.82	125.5	130.52
PSMD4	145.83	146.26	140.11	137.79	148.21	144.99	131.91	138.64
PDIA6	162.67	165.67	164.99	170.71	153.35	145.52	167.61	163.05
FAM129B	141.38	146.18	134.09	130.1	141.66	145.58	109.91	123.23
TPM3	159.03	160.89	158.88	156.08	149.73	145.63	144.35	141.1
SHISA5	184.59	194.78	182.83	175.3	142.47	145.7	123.82	128.05
PIK3R2	139.94	141.92	131.2	126.54	134.11	146.08	105.75	121.16
FAM96B	138.4	146.56	135.6	126.31	140.72	146.09	114.9	124.43
ARL2	145.25	150.41	134.18	125.03	147.07	146.22	112.52	122.5
ATPIF1	138.53	141.69	133.22	136.1	153.77	146.29	132.05	141.21
CALM2	153.08	159.19	149.94	154.17	158.17	146.61	186.59	163.31
DRAP1	147.17	148.26	133.04	128.34	152.96	146.72	110.95	125.38
MAP2K2	136.11	137.02	131.3	128.47	139.47	146.78	110.35	122.16
ADAM15	139.56	139.97	135.26	122.88	139.72	147.2	114.66	125.27
CALML3	187.56	187.53	169.45	152.29	136.58	147.3	101.46	118.76
C19orf33	163.14	177.17	157.92	147.85	143.69	148.33	110.39	126.01
EIF4G1	146.9	152.6	146.89	144.07	147.23	148.44	134.28	140.49
CLTA	146.29	150.55	148.72	140.38	148.62	148.7	135.69	146.83
NDUFB2	142.49	144.08	139.2	128.17	152.89	148.71	126.51	128.24
TM4SF1	150.18	147.71	153.84	163.83	144.26	149.91	171.12	153.56
SOD1	156.51	157.91	158.48	158.27	154.26	150.55	152.92	159.4
HNRNPUL1	155.9	158.57	150.79	149.1	151.39	150.69	130.66	138.51
PLEC	152.44	145.94	143.47	137.51	134.98	150.72	112.46	121.82
PTBP1	166.61	160.66	158.77	150.57	153.05	150.84	126.35	133.18
CCT5	144.31	143.18	148.83	146.44	163.87	151.73	167.4	160.65
PPP1CA	164.22	168.64	155.27	142.04	151.85	152.43	117.5	126.71
RAD23A	149.91	154.71	145.77	136.89	155.48	152.54	128.99	139.55
MCM7	157.36	152.56	147.61	141.87	150.78	153.85	133.42	129.96

XRCC6	160.74	163.75	160.07	150.83	159.25	153.85	159.23	147.24
BAG6	156.76	157.93	149.62	139.59	151.79	154.11	124.99	134.97
MLF2	152.1	159.66	148.2	144.29	160.76	154.43	130.06	146.41
PPIB	173.52	174.22	159.21	151.11	154.19	154.58	130.29	142.74
PABPC4	146.59	144.66	144.84	142.81	155.37	154.65	136.41	147.1
MGAT4B	143.42	148.94	141.79	133.92	146.61	154.76	125.86	131.26
STMN1	162.59	163	164.36	158.57	168.09	155.32	178.64	164.65
ATP5O	146.3	152.71	148.7	144.53	162.53	155.46	164.61	164.05
FAAP20	152.6	148.48	144.62	132.52	135.33	155.5	101.11	112.11
SRM	146.73	141.54	139.68	127.24	148.62	155.63	117.39	125.04
APRT	148.83	142.06	143.41	122.75	156.49	155.72	119.06	131.24
SUMF2	164.73	177.54	167.22	158.49	145.86	156.1	133.56	142.36
ATF4	155.8	154.99	162.18	158.03	165.82	156.64	159.89	155.54
TMBIM6	165.43	169.89	165.34	164.78	159.52	157.23	158.57	159.45
AP1S1	138.73	144.35	137.3	130.84	160.06	157.35	139.36	145.18
ATP5G1	140.08	147.89	140.25	131.45	160.43	157.44	137.15	135.6
VDAC2	146.05	154.27	147.91	142.51	159.53	157.75	158.26	149.88
HNRNPL	154.45	165.09	153.74	145.78	160.69	157.79	147.1	141.41
HNRNPK	172.47	170.67	172.42	174.17	170.54	158.04	184.55	171.81
CCT6A	162.7	145.96	165.37	175.58	157.71	158.19	198.18	180.42
TMA7	153.52	163.55	155.93	158.77	171.96	158.58	159.06	164.01
HNRNPC	175.35	182.94	174.86	171.35	172.27	158.58	169.23	175
ISG15	411.86	404.1	379.05	350.12	151.59	158.9	125.12	112.77
ITGB1	150.15	150.27	171.52	188.99	169.56	158.99	233.6	211.96
BEX3	164.42	169.03	164.7	171.69	156.63	159.29	154.44	160.39
SLC14A1	208.86	206	209.24	216.29	164.55	159.75	175.43	169.94
C6orf48	148.59	150.95	153.78	151.25	165.36	160.16	152.53	158.37
AP2B1	155.42	162.61	160.62	153.94	166.12	160.35	175.63	166.5
YWHAQ	166.6	162.49	172.78	167.93	159.38	160.62	173.14	163.86
RNH1	165.38	163.67	155.03	143.37	157.78	161.28	117.27	128.84
MYL12B	159.63	161.29	162.81	164.81	170.32	161.3	199.6	182.25
EIF3C	156.59	151.45	143.67	141.97	165.82	162	150.47	157.5
TMED9	166.71	177.61	160.67	158.61	158.57	162.16	131.73	144.16
ISYNA1	203.35	199.06	188.84	166.85	150.8	162.51	123.8	136.7
C1orf43	147.46	148.05	149.73	149.75	170.16	164.14	160.97	156.42
STIP1	180.76	189.94	173.83	170.1	170.67	164.57	152.07	152.84
CTNNB1	167.46	154.78	172.58	180.99	171.3	164.83	199.41	184.31
UBE2S	155.11	151.3	146.47	129.44	153.23	164.9	112.21	123.03
COPE	173.06	171.03	158.19	149.4	159.61	165.21	124.43	132.71
CPNE1	178.13	183.63	174.22	164.44	165.41	165.25	148.21	157.26
SEMA3F	209.62	221.97	196.66	194.2	148.17	165.37	124.75	144.5
HIGD2A	167.04	159.26	155.91	145.79	159.78	165.92	136.71	137.81
TAF10	150.67	151.6	148.17	134.52	159.74	166.06	129.03	133.87
TSPO	156.16	147.81	136.46	130.43	152.54	166.41	112.97	120.85
PKN1	167.39	165.71	160.42	143.06	164.04	166.49	120.54	135.64
AURKAIP1	152.08	157.66	149.16	139.29	160.4	166.74	123.99	136.6
SERINC2	137.97	142.95	134.44	133.18	171.3	166.79	132.17	145.62
TRMT112	178.31	177.39	161.53	159.02	171.78	167.1	132.86	144.1
IER3	184.17	174.54	171.04	170.3	173.74	167.38	144.03	146.82
TIMP2	166.57	181	171.83	169.92	162.25	167.55	153.42	159.9
ARPC1A	171.66	177.98	164.68	161.45	165.09	167.8	156.03	157.43
PSMB3	167.53	169.28	158.7	145.4	163.16	167.83	129.83	136.99
LAPTM5	141.92	146.64	141.97	141.78	179.66	168.46	167.49	166.17
SSR4	182.31	186.36	168.26	164.76	177.4	168.55	141.04	150.97
TMEM205	172.72	171.95	165.72	148.44	160.29	168.63	133.29	143.41
CLU	307.06	336.65	285.3	295.42	148.96	168.79	123.29	162.1
EIF3H	170.76	162.6	176.21	183.01	185.46	170.43	207.01	194.18
HMGA2	190.11	164.7	194.07	208.08	170.68	170.77	179.31	181.72
LFNG	184.88	187.64	181.94	171.18	154.43	170.83	136.65	144.96
CAP1	168.73	166.76	171.35	169.75	178.91	170.88	183.17	178.24
EIF4B	148.12	149.1	152.39	154	179.69	171.53	182.42	179.79
LAMTOR4	179.1	177.25	166.3	145.16	170.78	172.02	133.72	139.81
SLC2A4RG	194.09	187.13	178.18	167.77	162.34	173.07	121.14	133.52
HSPD1	161.08	147.83	172.69	180.74	188.53	173.07	224.06	212.49
CANX	177.76	171.29	185.74	195.87	192.6	173.18	228.17	209.62
HNRNPA2B1	182.66	160.79	194.45	208.54	198.2	173.94	245.2	237.86
RTN4	169.28	162.89	169.69	178.7	172.13	174.12	168.51	170.41
SSR2	188.25	194.35	183.98	176.21	183.16	174.46	179.99	174.21
HDLBP	182	184.76	180.07	176.59	172.64	174.49	163.85	169.43
ACLY	133.04	145.54	131.88	136.41	164.19	174.77	150.75	168.74
HDGF	172.98	174.92	172	168.78	186.37	175.12	161.29	170.36
G6PD	159.85	163.86	148.76	142.64	160.55	175.45	126.43	147.04
MSN	181.81	191.17	180.5	176.83	181.98	175.7	168.74	177.38
NDUFB9	170.93	175.51	166.13	153.75	181.1	175.82	161.64	163.35
CDC37	163.34	165.46	163.22	161.41	173.18	176.29	156.24	160.26
ARPC1B	169.97	171.58	161.1	152.61	164.95	176.42	125.52	142.69
PCBP2	179	187.28	176.71	175.5	180.32	176.42	161.15	172.77
APEX1	171.85	173.86	165.72	162.55	193.54	177.46	164.85	170.65
AKR1B1	176.66	182.72	165.88	157.84	179.37	177.5	157.45	162.33
VDAC1	177.73	178.39	177.52	176.61	188.49	178.85	192.88	186.36
PLXNB2	187.12	187.47	178.85	175.5	169.95	180.68	145.41	154.28
EIF3I	172.23	175.19	173.78	161.1	191.62	181.42	170.53	175.08
PNPLA2	182.13	182.29	172.81	163.02	172.13	181.89	132.46	150.62
ATP5G2	212.96	210.56	205.59	193.97	201.53	181.9	161.82	182.31
SH3BGRL3	176.64	184.75	161.33	160.2	181.11	181.94	148.96	169.92
UQCRC1	181.62	189.29	176.12	161.04	182.03	182.15	154.92	161.3

IMPDH2	170.28	170.27	163.27	156.42	190.54	182.49	166.41	168.22
WBP2	174.59	182.19	164.86	169.2	180.76	183.04	140.08	168.16
HLA-A	190.16	205.91	192.18	180.89	185.45	183.07	150.98	160.31
PRMT1	187.03	184.83	174.27	165.27	178.73	183.08	145.38	154.62
EPHA2	221.72	221.57	203.99	203.39	174.28	183.31	146.59	161.51
CNN2	224.33	234.37	215.78	200.19	181.67	183.62	142.67	164.6
YWHAE	196.85	201.3	200.63	198.06	193.64	183.65	186.58	202.92
ILF3	188.92	190.55	185.67	184.02	188.74	184.1	178.52	177.66
TRIP6	198.68	189.6	185.32	175.23	186.37	184.75	143.93	157.34
GDF15	149.14	148.7	144.08	126.68	181.59	184.82	140.22	147.32
RAB5C	179.6	186.47	175.24	167.07	187.72	184.85	146.15	160.02
ACOT7	180.5	180.12	164.79	156.26	182.88	185.52	140.23	152.44
S100A10	186.92	198.41	189.1	182.34	200.37	185.6	195.59	187.71
SDF4	194.63	200.43	185.68	182.64	185.59	186.82	141.4	164.35
NDUFB4	185.29	201.84	180.18	181.26	178.98	187.68	174.82	179.02
COX5A	167.21	173.33	168.9	150.26	185.89	188.72	168.07	173.55
DYNLL1	209.71	207.94	203.9	205.69	193.32	189.56	195.64	185.58
NME1	198.21	203.39	188.01	184.58	217.88	189.8	179.42	187.08
UBA1	193.52	198.64	186.73	176.32	181.64	189.91	158.58	169.97
SUMO2	199.87	205.25	200.31	209.99	204.04	189.92	210.95	211.72
TBCA	185.67	197.76	190.76	190.42	202.99	190.12	197.81	205.16
ATP6V1F	190.22	201.42	174.72	171.94	193.25	190.42	150.13	171.57
RALY	208.39	201.26	197.21	184.05	183.52	190.48	143.61	152.88
H3F3B	214.93	205.23	212.37	211.91	192.09	190.59	188.77	188.29
SQSTM1	185.94	186.68	182.13	174.92	190.77	193.54	159.23	173.34
CX3CL1	215.08	226.5	209.74	209.45	191.83	194.22	165.82	186.51
NPC2	171.8	188.67	173.39	180.23	194.71	194.45	181.51	185.52
GNB1	205.12	208.01	210.8	204.06	199.96	195.41	195.08	193.32
GNAI2	217.26	223.48	202.85	199.19	192.04	195.62	157.94	173.75
EPHB4	215.22	217.32	204.05	200.5	184.18	195.72	150.43	172.16
NDUFS5	172.55	199.61	179.03	185.65	202.89	195.72	177.37	194.78
NDUFA11	199.45	214.19	190.25	177.18	185.66	195.76	139.59	154.91
NAXE	190.52	196.5	181.11	171.42	197.36	195.9	160.98	171.73
NCL	190.96	175.1	199.15	209.55	221.66	196.67	245.91	235.02
TXN	191.49	197.71	192.29	195.76	199.11	196.87	235.42	209.65
IGFBP7	222.06	213.87	207.92	210.31	201.78	198.11	161.45	162.31
CLSTN1	214.87	228.4	209.42	205.95	182.55	198.23	165.52	176.59
DSTN	177.46	175.1	188.49	186.17	219.57	198.3	250.23	223.12
EIF3E	183.33	187.48	184.95	189.72	213.41	198.49	263.6	227.09
SLC25A1	179.22	193.48	176.4	160.48	182.25	199.13	141.85	162.82
GRINA	222.15	226.11	207.02	199.98	200.27	199.28	159.02	183.78
SND1	199.06	203.87	195.62	178.59	198.92	199.65	173.81	181.46
COL6A1	172.18	179.43	171.84	169	178.45	199.77	157.37	173.65
GLTSCR2	208.84	207.46	199.45	182.84	199.58	199.85	147.63	166.69
ARF5	214.45	225.09	213.8	190.36	197.71	200.66	153.5	168.71
H1FX	243.24	243.9	226.48	201.88	182.48	200.9	130.83	150.88
LAMB1	186.91	182.52	202.52	220.8	201.5	201.76	248.14	236.28
APLP2	202.65	206.91	201.95	210.22	205.74	202.09	212.11	206.88
NONO	212.48	215.49	211.14	204.42	212.75	202.77	205.65	202.3
CYC1	180.8	181.46	172.46	153.66	188.53	203.46	138.95	156.99
EDF1	225.86	237.47	230.36	207.61	204.76	203.96	171.3	175.73
POLR2L	221.39	208.22	211.04	184.48	196.92	204.04	164.86	167
CSDE1	183.73	171.55	197.33	215.3	215.89	204.36	268.26	243.8
JAG1	192.19	185.14	199.64	204.35	215.74	204.82	229.68	205.12
IFI6	615.08	607.98	596.75	582.59	212.58	204.94	183.98	148.99
HSP90B1	220.85	216.07	248.69	285.95	217.73	205.03	323.43	291.01
DDIT4	259.57	267.1	247.48	232.5	200.8	205.05	179.7	194.23
ATP5D	188.73	190.78	173.74	157.31	199.19	206.03	143.96	158.01
EIF3K	183.18	193.88	185.33	174.35	211.21	206.11	175.9	186.72
C19orf43	208.94	211.14	201.94	194.03	215.56	206.4	157.27	181.46
DUSP4	217.85	221.02	214.67	217	192.72	206.56	184.38	200.08
RPN2	214.16	205.2	215.95	224.29	208.73	207.69	223.83	219.61
NHP2	188.22	185.22	177.35	176.95	217.97	208.63	191.52	182.5
MYL6	248.82	255.68	235.88	224.5	219.5	211.32	188.76	190.72
MDH2	197.75	201.62	192.75	180.79	217.26	213.27	180.73	198.35
RHOA	226.11	237.01	225.25	218.27	233.2	213.68	219.46	213.66
FBL	248.65	237.87	231.29	215.98	223.16	213.98	203.26	201.59
EIF3L	199.55	203.41	198.86	195.66	226.99	215.22	224.77	218.06
HSPG2	199.52	204.64	192.05	192.45	196.78	215.96	176.72	194.68
ROMO1	235.87	222.22	213.37	200.73	223.15	216.73	164.98	184.46
ITM2B	234.76	235.16	242.55	252.46	222.12	217.18	254.63	236.59
LDLR	189.62	210.73	188.68	200.54	204.88	217.36	187.37	210.1
PARK7	211.1	216.19	215.88	208.18	221.61	217.42	209.19	209.34
CMTM7	211.49	221.06	210.79	211.74	226.06	217.61	202.46	214.84
COX5B	219.39	228.94	210.46	198.55	221.88	217.95	196.24	194.15
DYNLRB1	230.5	243.05	223.13	222.64	232.01	218.25	192.88	218.83
BANF1	227.78	251.01	224.38	231.87	225.74	219.55	190.15	198.61
ACTN1	214.72	221.37	209.76	204.43	217.52	219.86	186.44	192.53
NAP1L1	203.89	193.07	215.21	223.19	234.31	220.17	281.94	261.52
DKK3	230.8	234.49	224.34	226.6	220.16	223.18	203.64	209.33
H2AFY	226.89	230.46	230.37	223.28	225.73	223.56	220.73	219.92
TXNIP	295.35	305.19	299.87	290.46	229.21	224.14	250.93	242.24
PLP2	251.32	257.37	247.92	240.9	242.48	225.13	204.66	215.33
PDIA4	251.91	262.37	241.93	242.36	221.82	225.29	211.84	217.44
COX6B1	222.52	210.44	208.97	204.09	234.43	225.38	198.83	188.82
HMGN2	231	223.63	233.1	216.15	232.73	225.88	225.79	210.01
NDUFB7	227.06	216.81	217.05	193.45	226.47	226.96	173.89	175.71
TECR	223.52	239.89	225.04	203.63	220.59	227.14	178.16	203.16
CCT7	240.53	248.06	236.75	222.48	227.96	227.39	211.48	216.78

MZT2A	214.88	208.61	206.03	186.49	214.27	227.44	157.6	170.6
PHB2	219.5	227.13	215.86	200.68	230.22	227.79	197.15	199.21
RPL27A	244.92	249.05	237.75	234.27	238.52	229.92	215.01	216.47
GANAB	239.79	247	237.65	237.05	230.16	230.44	222.67	226.16
GPC1	229.97	230.14	217.64	205	223.03	230.72	174.28	191.27
BRI3	221.1	220.86	210.17	198.08	215.04	231.49	178.9	196.49
GUK1	255.48	255.27	234.85	218.56	223.5	231.54	167.17	183.31
TTYH3	242.95	243.31	229.09	216.83	212.2	232.06	168.65	195.06
TP63	246.19	236.75	262.25	271.85	242.22	232.18	249.79	261.14
PYCR2	249.76	261.15	252.15	238.29	239.34	232.81	208.03	225.05
YWHAZ	229.2	226.36	238.45	245.57	240.78	236.56	285.73	268.55
HMGA1	230.23	227.47	224.6	212.64	248.9	237.01	194.52	212.63
JUP	278.26	281.88	260.64	242.18	221.4	237.08	179.47	200.2
KRT75	183.23	170.22	185.86	166.83	253.18	237.22	205.49	193.25
TUFM	217.39	218.22	203.07	190.06	240.34	237.35	187.34	204.64
CLIC1	263.18	272.97	259.97	241.41	236.38	237.42	207.13	206.06
CD44	206.17	205.46	206.7	207.55	235.06	237.8	239.4	240.56
EIF6	236.34	243.58	229.37	215.96	239.74	239.11	193.7	206.46
RPL22	239.85	226.29	237.74	247.89	260.37	243.65	280.78	275.59
COLGALT1	225.52	239.03	220.98	218.18	232.74	243.82	204.42	220.91
TUBB4B	248.17	247.73	229.35	207.64	233.61	243.94	189.62	201.45
ARF1	252.1	253.75	245.3	224.43	239.47	244.09	212.62	223.46
TGFB1	239.66	252.53	230.49	225.06	228.51	245.08	179.43	208.33
ATP5J2	259.44	265.36	233.56	237.87	262.03	245.6	218.23	233.17
CTSB	250.38	259.51	250.77	238.64	255.17	247.44	234.49	231.99
LY6E	361.02	360.37	337.61	305.64	241.8	247.9	183.93	192.51
SYNGR2	223.72	235.55	225.26	213.18	253.17	248.19	199.27	229.76
CAPS	287.72	289.97	273.96	247.66	228.54	249.32	172.99	201.41
ERGIC3	274.94	273.49	260.03	251.6	244.85	250.31	211.33	212.43
EIF4G2	236.63	221.77	249.3	251.81	261.34	250.93	304.9	285.49
FDPS	217.97	251.18	221.22	221.59	255.78	251.05	221	252.74
RPS26	246.26	254.34	238.57	223.55	246.94	251.54	220.75	213.98
COL9A3	255.4	265.28	246.68	257.25	213.69	253.24	207.25	231.56
ITGB4	237.9	237.88	230.63	223.19	241.26	254.28	202.91	221.74
RPS23	281.83	282.69	271.61	265.08	277.74	254.55	258.66	254.46
SAT1	184.64	199.32	201	216.97	256.79	255.89	298.03	282.5
PGAM1	341.99	336.34	324.53	300.76	255.54	256.98	232.09	228.38
ATP6V0C	235.03	255.39	224.18	213.45	253.27	258.36	178.22	209.65
SLC25A3	228.16	240.47	223.28	218.24	264.62	258.9	241.2	250.5
UQCRH	249.64	244.18	264.6	237.68	275.76	259.99	277.86	273.91
FLOT1	327.24	349.74	334.07	310.24	274.44	260.32	226.94	255.63
ARPC2	259.35	263.11	268.39	269.23	271.14	261.12	273.2	263.41
HINT1	257.95	264.19	243.29	241.71	268.33	264.41	260.66	256.43
SREBF1	171.43	203.71	172.69	189.42	215.83	265.07	172.16	215.6
B2M	294.06	296.37	308.67	321.1	281.18	268.32	346.1	296.99
EEF1D	263.9	260.57	251.55	233.17	267.82	270.28	205.31	233.66
ATP5A1	256.81	262.65	254.04	256.34	280.86	270.51	267.47	273.06
BCAP31	276.96	294.1	266.17	259.34	278.34	271.1	233.76	252.25
EIF3F	268.1	268.14	255.99	249.3	272.74	271.82	238.71	245.81
TNS4	255.68	270.15	260.09	255.16	277.95	272.05	261.25	283.02
CTAG2	256.12	251.66	237.7	213.93	266.21	272.55	183.26	202.28
KRT5	319.04	311.33	303.47	286.42	268.55	273.46	217.43	235.87
CCT3	265.93	273.59	262.87	245.63	286.57	273.69	259.14	254.43
TGFBI	356.41	368.62	348.21	345.04	258.21	273.92	251.18	257.61
PLD3	324.52	329.76	298.39	294.84	257.9	273.97	193.84	223.48
EIF3G	264.85	263.04	254.08	238.33	272.91	274.62	208.97	228.18
DDOST	278.98	292.08	277.57	268.57	283.03	275.06	259.65	268.69
MZT2B	281.34	272.75	266.45	234.24	266.8	275.78	184.04	205.45
HSP90AA1	284.27	249.81	310.69	327.86	301.24	276.16	389.62	342.55
CD81	298.7	301.76	283.66	267.59	269.7	276.94	224.53	241.72
PRDX1	264.78	259.32	280.91	260.19	287.41	277.02	300.87	291.09
FKBP10	285.25	299.66	268.94	270.44	273.8	277.89	229.57	251.81
FKBP1A	273.36	289.27	271.23	265.92	296.26	278.01	276.88	288.32
CAPNS1	288.71	303.87	274.14	266.75	277.07	278.17	237.56	261
PRDX5	284.75	306.14	271.5	265.98	283.45	282.43	205.15	253.55
SDC1	273.47	292.33	263.83	257.91	256.98	282.78	213.61	242.91
ATP5H	278.76	280.82	273.47	269.86	293.85	284.55	282.58	291.86
LDHA	447.35	431.06	452.87	414.4	280.7	285.06	312.53	281.18
PGK1	378.77	380.72	374.16	348.28	276.48	285.2	280.72	276.7
DHCR24	256.61	276.51	251.98	251.35	267.78	285.26	243.39	264.72
PSMB4	310.62	296.41	300.98	270.04	270.72	286.88	237.42	257.61
NDUFS6	273.48	287.27	251.87	250.96	271.91	287.27	214.7	224.61
RHOC	292.29	313.17	279.36	260.97	286.34	287.55	233.78	257.28
44448	323.64	323.44	309.75	298.91	285.87	288.28	227.99	251.29
AP2M1	289.14	298.67	281.41	275.5	288.85	288.66	248.92	262.77
SNRPB	302.85	305.56	291.6	265.04	293.44	289.46	225.27	237.6
KRT18	319.11	336.79	311.61	287.11	284.88	292.12	246.45	270.06
EIF4A1	284.04	290.66	277.93	276.78	309.76	292.7	290.09	293.79
NDUFA13	300.15	304.01	278.33	267.27	308.58	295.68	241.57	257.69
YBX3	302	288.49	286.9	280.84	293.37	296.69	248.44	262.65
AES	325.85	346.92	318.13	314.74	295.68	297.41	240.28	269.33
ACTN4	313.62	328.53	301.3	294.19	298.18	301.96	239.56	268.13
DDT	273.19	283.5	278.1	252.88	311.33	306.23	251.85	264.69
CD63	329.49	342.15	309.07	316.87	324.83	310.04	278.12	293.57
KRT15	337.5	327.88	343.16	336.04	291.34	312.39	297.45	300.98
ADRM1	312.43	305.22	286.13	269.21	297.23	313.42	224.59	253.19
EIF5A	310.86	320.29	296.8	293.39	331.53	314.75	265.56	285.74

PPP1R14B	297.11	293.92	279.24	251.17	312.63	315.47	231.41	258.06
TUBB3	290.04	293.37	270.49	252.18	293.34	317.2	222.32	246.07
PCBP1	312.14	314.24	304.27	295.99	309.76	317.84	255.97	277.54
FDFT1	253.69	301.45	260.78	265	306.06	320.41	285.89	320.45
CD151	343.98	343.11	327.67	312.08	308.82	320.64	257.47	270.3
MMP14	346.91	368.76	342.58	344.95	312	323.58	277.68	298.77
UCHL1	303.43	315.54	307.42	295.52	345.13	323.85	305.47	307.62
SHC1	360.75	359.1	342.96	339.19	317.16	325.49	287.47	296.2
KRT17	382.21	387.48	383.18	346.29	358.53	328.38	273.84	275.36
FKBP8	354.74	357.22	334.86	311.44	320.86	330.52	234.11	272.12
EIF4H	345.01	348.13	341.14	335.53	341.15	331.5	307.41	308.33
PRDX2	307.59	312.17	293.56	277.9	336.22	331.92	269.41	278.49
CHCHD2	339.72	336.7	326.94	292.5	344.39	332	291.15	298.39
CHCHD10	315.09	312.74	300.86	256.04	314.81	332.89	229.07	261.63
PDLIM1	416.85	414.39	395.36	383.43	335.51	333.3	308.55	298.75
CD9	274.19	294.5	267.97	277.96	329.05	336.8	304.06	322.78
MARCKSL1	356.35	382.02	359.65	351.88	340.94	337.42	278.38	309.52
GPX1	360.99	370.56	339.7	299.9	333.99	340.26	242.24	269.41
CRIP2	384.92	380.75	354.87	343.02	326.54	346.53	231.86	271.83
PRKCSH	398.9	401.25	371.62	358.61	339.8	348.92	289.77	307.37
RPL37	419.46	408.61	382.34	389.13	360.5	350.56	344.24	329.84
ATP5E	357.3	372.76	338.26	345.94	374.32	352.43	360.14	340.23
TUBA1C	340.32	343.04	326.79	301.98	342.9	353.07	297.22	320.88
CDKN1A	247.08	273.47	252.43	243.01	367.02	361.71	302.03	321.99
KRT8	361.67	387.15	348.52	323.64	345.9	363	275.89	310.84
PPDPF	417.69	406.55	389.55	367.55	356.15	364.3	249.01	304.23
SLC25A5	334.98	350.63	338.5	314.16	376.84	366.71	333.96	340.37
PYGB	323.02	324.94	309.24	296.47	368.11	369.98	310.57	326.09
SLC16A3	448.36	454.97	420.53	397.34	349.32	373.7	274.01	308.78
CHPF	445.61	462.36	413.39	391.67	330.9	378.1	252.89	293.54
TINAGL1	359.56	382.11	341.67	336.15	340.84	379.77	277.54	334.24
DDR1	412.18	439.52	397.6	403.56	361.31	380.24	306.58	342.71
TAGLN2	453.2	460.76	432.26	406.89	403.4	382.57	331.04	331.77
CTSD	397.03	404.76	374.19	348.96	367.66	382.74	286.07	307.14
RPL32	431.97	424.16	413.28	400.9	404.24	386.21	375.21	371.33
COX6A1	380.73	380.91	368.14	360.83	382.84	389.61	310.23	360.17
GNB2	402.11	415.83	381.02	351.66	374.27	389.91	286.63	318.06
COX4I1	383.52	390.45	376.88	360.72	411.57	391.01	353.21	362.66
UBL5	385.09	414.26	369.64	367.21	388.31	391.06	324.69	355.78
HSPA1B	453.22	447.16	425	397.39	383.56	391.77	297.17	341.08
TKT	346.69	365.49	334.07	311.29	392.49	392.36	307.21	333.93
GNAS	390.53	421.19	389.87	379.31	385.6	392.56	367.7	368.36
PSMA7	397.82	396.76	369.96	353.72	396.39	395.66	321.09	337.83
NACA	394.98	399.51	408.85	423.26	402.4	395.73	474.25	462.52
MIR205HG	444.93	466.33	458.03	496.63	408.18	396.95	418.2	459.14
AHCY	343.76	339.52	318.92	300.44	393.17	398.51	314.68	336.49
AGRN	471.23	466.99	441.47	432.84	365.99	401.37	302.34	332.48
TPT1	431.55	426.72	440.8	426.38	429.95	403.57	429.16	425.4
EEF1B2	418.52	407.14	397.95	373.48	448.02	411.13	396.28	428.35
TPI1	517.43	528.78	497.65	460.55	399.66	411.17	359.98	373.72
TCEB2	405.74	402.29	388.36	369.7	394.07	413.08	325.27	343.64
ATP5B	380.83	391.76	374.34	342.73	421.9	416.1	378.36	390.01
LDHB	382.47	377.39	391.28	400.32	439.31	417.01	477.65	465.94
APP	415.55	419.34	428.63	449.32	426.43	417.09	453.64	447.22
LMNA	450.85	434.55	426.05	381.77	396.45	419.38	312.46	346.28
GPX4	446.74	447.9	428.69	396.96	430.43	422.04	315.04	344.29
EIF1	418.88	445.93	428.19	403.09	445.4	423.37	415.02	413.28
RPL36A	487.92	508.71	474.63	489.05	486.85	427.41	453.56	452.28
PSAP	427.81	447.46	425.83	424.81	421.83	428.62	397.57	404.28
RPL34	488.17	473.77	482.59	453.42	461.76	429.19	479.21	445.41
IGFBP4	457.29	476.43	440.4	410.16	415.69	432.3	331.41	375.28
RPL5	419.58	400.65	426.74	412.55	461.86	433.8	469.21	468.18
IGFBP2	469.99	473.13	442.49	399.09	406.23	434.4	292.5	325.91
OAZ1	434.62	461.45	407.35	399.17	409.27	435.62	342.79	358.21
ARHGDIA	449.78	454.26	416.74	396.1	442.82	435.83	328.07	359.95
SFN	409.1	426.61	378.79	360.95	437.95	443.93	320.2	377.82
COX8A	462.41	461.55	441.52	404.82	462.54	455.09	359.34	386.15
RPL13	519.07	510.57	484.07	437.89	458.9	471.4	347.19	383.04
ATP1A1	466.34	495.13	462.01	457.12	467.81	477.32	439.12	477.45
RPS29	605.2	586.02	513.35	495.93	503.93	486.81	395.17	445.72
PFN1	523.43	540.9	495.93	462.22	507.79	514.27	409.5	443.5
FLNA	543.6	526.35	509.72	477.87	492.58	514.75	410.86	432.4
HSPA8	537.99	508.32	534.38	520.36	551.98	522.36	557.33	519.53
GSTP1	504.29	524.43	488.64	448.18	528.91	523.68	414.26	445.87
SCD	364.43	454.42	391.34	452.65	473.89	527.5	494.25	547.63
GRN	508.21	506.24	478.76	460.55	501.47	528.96	408.88	438.22
FSCN1	603.31	591.19	566.84	512.2	496.3	529.15	381.86	423.67
H3F3A	525.45	535.67	547.03	550.19	549.43	537.81	603.04	554.33
NPM1	514.09	498.03	539.53	571.53	592.02	541.93	710.03	661.6
RPL38	534.32	586.72	509.02	513.93	519.07	550.52	451.65	458.78
YBX1	557.78	564.05	548.28	536.49	553.57	553.71	487.66	496.84
P4HB	655.15	664.78	613.35	600.96	559.03	576	491.9	512.98
LGALS3BP	745.79	778.39	711.93	687.6	554.62	579.04	479.4	504.12
COX7C	593.02	592.88	577.14	580.08	616.27	593.7	589.85	602.03
RPL15	624.65	635.81	609.89	571.62	632.11	594.17	563.69	556.4
FTL	599.78	613	588.04	555.51	595.69	594.59	491.3	548.71
SLC25A6	587.46	574.84	560.79	517.16	573.33	596.54	435.88	496.21

RPL21	590.85	646.74	638.93	638.8	656.66	597.86	657.38	640.73
RPS27A	658.2	660.63	651.94	656.43	663.17	603.08	711.19	702.95
HNRNPA1	637.46	619.61	655.44	655.86	667.28	605.49	707.04	703.32
PTMA	660.15	653.39	646.1	638.71	661.89	607.91	575.57	613.2
TUBA1B	691.67	692.63	672.82	611	633.69	613.76	540.8	563.58
PPIA	582.51	589.56	579.96	573.92	617.74	614.14	612.6	612.37
MDK	659.52	694.35	636.2	621.51	593.01	617.12	459.1	529.42
RPS24	667.61	664.59	650.88	652.25	660.41	619.41	694.92	689.33
BTF3	627.88	590.45	640.81	643.28	669.07	623.77	722.58	681.38
UBC	633.98	663.3	644.47	608.88	679.48	651.41	628.75	607.47
ITGA3	745.25	746.1	702.99	686.68	620.16	656.48	537.05	572.96
RPS25	727.73	687.71	695.87	685.5	709.82	675.71	692.78	674.08
BSG	730.46	743.87	690.47	647.67	670.24	679.38	505.26	557.28
FAU	707.68	711.69	680.25	619.42	683.92	679.38	543.42	586.15
CFL1	707.74	742.96	689.64	657.69	700.32	689.06	570.95	624.55
TMSB4X	773.85	764.35	803.39	829.28	729.97	694.82	781.93	771.95
ANXA2	692.81	705.42	701.18	676.17	721.37	705.06	718.83	655.98
CST3	653.1	667.11	627.92	575.26	679.41	713.59	505.08	572.86
CKB	851.04	828.79	788.96	724.62	651.05	717.21	496.34	561.03
S100A6	610.07	611.17	579.94	545.26	731.6	731.6	570.58	640.64
RPL14	684.39	688.87	706.9	702.21	768.17	737.29	764.84	727.22
RPL24	801.03	822.11	796.39	753.51	796.3	747.95	743.13	722.27
RPL10	932.07	931.11	900.25	831.81	746.22	749.03	647.26	697.77
RPL26	804.2	804.03	807.83	851.31	840.04	779.63	865.67	808.05
TUBB	923.33	934.28	886.26	827.16	784.58	785.43	700.19	703.82
RPS3A	867.33	887.39	854.59	820.75	876.57	816.6	862.91	848.29
RPS13	924.33	927.54	942.45	877.63	891.49	824.86	903.91	863.5
S100A11	1006.89	1069.99	994.93	941.14	848.75	830.47	775.31	817.29
HSP90AB1	857.3	854.87	848.43	834.51	867.45	831.73	833.84	854.58
PKM	1035.24	1044.38	985.32	913.65	861.39	872.43	737.86	776.88
CALR	932.93	1035.95	917.23	920.35	889.24	894.84	791.02	885.03
FASN	518.87	626.09	524.59	558.92	736.14	898.97	588.81	744.9
S100A2	784.9	799.62	792.35	734.9	970.69	903.39	806.05	844.01
RPL39	994.31	1004.66	994.79	940.49	947.68	906.17	903.27	925.36
RPL35A	998.81	973.9	948.33	932.25	961.35	922.62	877.04	861.1
NME2	895.31	901.1	833.72	798.42	974.71	935.39	828.36	816.7
RPS7	962.4	982.73	951.21	923.53	982.32	936.37	922.17	942.56
LGALS1	992.16	968.79	899.41	841.79	938.57	939.84	706.49	769.98
RPL10A	989.47	976.29	979.57	940.7	980.68	943.06	932.16	912.87
PABPC1	934.21	961.61	950.8	948.37	971.12	950.01	934.51	943.82
RPL18	1062.73	1081.93	1007.84	907.6	940.27	960.23	743.98	796.92
RPL28	1069.03	1005.36	1003.28	922.45	936.4	966.27	734.66	787.92
RPSA	953.56	985.28	936.78	855.69	1008.6	974.47	883.67	897.78
VIM	991.73	957.95	969.19	923.5	975.13	988.06	911.52	902.65
FTH1	1002.71	1041.29	979.44	931.06	985.14	996.19	843.24	862.72
RPS3	1068.42	1118.6	1021.98	998.95	1059.93	1029.85	871.63	963.38
RPL36	1176.61	1184.76	1144.11	1027.98	1056.32	1030.76	816.71	903.27
RPL30	1163.86	1149.99	1084.88	1045.11	1115.44	1033.87	1035.01	1014.54
RPS15	1127.4	1144.7	1078.97	973.48	1009.08	1060.66	783.29	821.92
RPL6	1039.06	1035.69	1105.69	1112.42	1126.34	1092.99	1222.77	1220.33
RPL7	1116.72	1163.08	1124.82	1144.59	1155.5	1105.56	1218.11	1237.49
RPS4X	1149.98	1145.43	1172.55	1140.38	1122.07	1105.96	1163.66	1160.42
RPL31	1143.63	1159.75	1094.83	1101.64	1168.91	1119.61	1139.61	1063.33
RPS10	1204.2	1207.72	1182.15	1060.21	1181.99	1151.88	1027.08	1014.25
RPL23A	1208.51	1237.11	1178.94	1133.59	1179.37	1155.53	1117.92	1160.31
RPL9	1248.58	1264.22	1210.71	1147.99	1243.75	1155.99	1215.43	1189.85
IFITM3	1925.83	1994.36	1843.25	1737.6	1177.42	1169.56	924.63	972.33
RPS20	1191.05	1236.58	1196.83	1118.29	1225.42	1173.19	1054.17	1106.48
RPS17	1298.68	1277.84	1242.62	1185.08	1184.42	1179.95	1084.5	1060.43
RPS5	1230.72	1256.51	1141.45	1091.93	1157.55	1200.73	972.47	963.53
EEF2	1197.16	1163.58	1115.6	1044.07	1203.23	1222.23	933.5	1016.32
HSPB1	1599.94	1604.03	1485.26	1402.54	1168.08	1286.19	844.6	991.58
RPL17	1340.54	1295.31	1309.79	1358.8	1309.16	1290.2	1371.03	1355.96
RPL35	1445.29	1472.19	1376.27	1235.47	1347.37	1292.4	1077.82	1120.36
UBB	1367.25	1454.99	1363.14	1258.3	1319.61	1320.98	1149.88	1196.62
RPS9	1520.92	1538.81	1443.75	1320.51	1392.64	1353.19	1045.84	1144.17
RPL12	1498.6	1506.77	1438.94	1394.49	1475.26	1375.84	1273.27	1285.42
MIF	1575.9	1558.54	1450.3	1306.46	1314.16	1399.42	936.27	1045.06
RPL29	1525.37	1597.52	1486.35	1432.17	1480.47	1413.78	1208.01	1315.43
RPS16	1586.14	1604.63	1506.79	1400.21	1429.11	1422.01	1151.53	1171.21
RPS15A	1585.04	1563.78	1582.91	1489.37	1514.96	1439.92	1463.32	1470.69
ALDOA	1833.43	1850.59	1706.81	1548.66	1375.18	1451.73	1123.15	1212.11
UBA52	1515.78	1545.58	1469.47	1438.91	1496.75	1452.21	1294.45	1383.15
RPL11	1594.23	1508.87	1590.99	1610.07	1509.18	1493.04	1581.42	1540.65
RPL23	1540.97	1550	1552.6	1539.67	1625.89	1504.23	1568.1	1572.16
RPS12	1599.01	1628.44	1601.04	1529.3	1535.11	1508.08	1421.37	1557.63
RPL41	1790.23	1757.48	1694.66	1656.88	1653.33	1573.74	1457.33	1412.78
RACK1	1666.57	1649.13	1640.61	1540.08	1666.42	1611.07	1477.6	1542.92
RPL7A	1682.21	1691.02	1604.99	1501.87	1716.49	1637.24	1517.41	1518.03
TMSB10	1813.07	1950.91	1822.72	1822.26	1641.06	1667.71	1394.35	1508.65
RPL4	1708.71	1715.52	1675.75	1602.91	1744.56	1684.32	1614.14	1652.12
EEF1G	1766.54	1856.73	1741.79	1612.83	1709.87	1691	1482.14	1584.2
RPL3	1758.3	1757.83	1689.84	1574.02	1783.78	1709.6	1491.5	1596.52
RPLP2	1922.94	1936.41	1842.53	1732.64	1820.31	1766.01	1434.37	1523.27
RPS11	2014.24	2080.58	1878.23	1872.3	1838	1776.08	1548.14	1580.48
RPL27	2053.47	2135.63	1976.96	1906.85	1930.14	1858.14	1625.51	1794.07
ACTB	2170.37	2229.31	2073.94	1947.39	1851.13	1874	1557.96	1677.2
RPS21	2065.33	2073	1976.4	1917.55	1917.78	1908.43	1589	1642.51

ENO1	2482.51	2512.83	2362.07	2203.76	1926.07	1965.7	1635.21	1683.57
RPS8	2177.57	2218.15	2210.15	2076.33	2096.69	2031.47	1873.71	1986.06
RPS19	2224.7	2264.36	2166.69	1935.75	2090.3	2043.61	1690.06	1741.43
RPL37A	2420.2	2423.71	2276.31	2243.51	2087.9	2101.32	1802.27	1849.88
RPS27	2605.97	2639.96	2520.76	2464.63	2423.6	2158.8	2143.89	2286.12
RPL13A	2369.19	2382.31	2274.8	2089.98	2336.05	2244.98	1789.02	2024.22
RPL19	2463.45	2526.14	2449.2	2311.36	2430.55	2296.15	2186.07	2259.83
RPS14	2466.54	2474.01	2408.26	2239.13	2283.74	2296.4	1976.75	1928.39
RPS28	2539.51	2675.22	2386.3	2290.19	2234.93	2317.38	1815.65	1866.36
RPL8	2615.29	2693.99	2435.2	2298.37	2454.01	2439.5	1852.54	2097.69
RPS6	2539.53	2562.85	2524.27	2475.62	2570	2447.63	2556.58	2585.43
ACTG1	2863.07	2862.99	2716.47	2533.69	2588.4	2586.36	2110.35	2278.18
RPLP0	2576.34	2685.34	2523.69	2380.35	2731.56	2694.15	2340.2	2444.88
RPS18	3040.13	3119.2	3033.11	2885.69	2993.14	2863.61	2670.75	2698.28
RPL18A	3251.81	3163.58	3034.86	2836.97	2958.13	2976.28	2229.41	2375.96
RPLP1	3483.96	3435.55	3284.86	2978.52	3259.9	3112.07	2455.31	2813.18
RPS2	3610.49	3540.32	3347.66	3062.35	3645.59	3568.76	2643.8	3019.88
GAPDH	4615.46	4626.32	4393.29	4118.53	3907.97	4045.97	3418	3511.21
EEF1A1	5060.78	4927.76	5044.02	4968.78	5293.12	5069.77	5152.95	5181.59
